# Ligand-controlled stereodivergent alkenylation of alkynes to access functionalized *trans*- and *cis*-1,3-dienes

**DOI:** 10.1038/s41467-022-35688-2

**Published:** 2023-01-04

**Authors:** Tianyu Long, Chen Zhu, Ling Li, Liang Shao, Shengqing Zhu, Magnus Rueping, Lingling Chu

**Affiliations:** 1 State Key Laboratory for Modification of Chemical Fibers and Polymer Materials, Center for Advanced Low-Dimension Materials, Donghua University, College of Chemistry and Chemical Engineering, Shanghai, 201620 China; 2 King Abdullah University of Science and Technology (KAUST), KAUST Catalysis Center (KCC), Thuwal, 23955-6900 Saudi Arabia

**Keywords:** Photochemistry, Synthetic chemistry methodology, Photocatalysis, Homogeneous catalysis

## Abstract

Precise stereocontrol of functionalized alkenes represents a long-standing research topic in organic synthesis. Nevertheless, the development of a catalytic, easily tunable synthetic approach for the stereodivergent synthesis of both *E*-selective and even more challenging *Z*-selective highly substituted 1,3-dienes from common substrates remains underexploited. Here, we report a photoredox and nickel dual catalytic strategy for the stereodivergent sulfonylalkenylation of terminal alkynes with vinyl triflates and sodium sulfinates under mild conditions. With a judicious choice of simple nickel catalyst and ligand, this method enables efficient and divergent access to both *Z-* and *E-*sulfonyl-1,3-dienes from the same set of simple starting materials. This method features broad substrate scope, good functional compatibility, and excellent chemo-, regio-, and stereoselectivity. Experimental and DFT mechanistic studies offer insights into the observed divergent stereoselectivity controlled by ligands.

## Abstract (web summary)

The stereoselective synthesis of 1,3-dienes represents a challenge in organic synthesis. Here, the authors report a ligand-controlled stereodivergent access to both *Z*- and *E*-sulfonyl-1,3-dienes via photoredox and nickel catalysis.

## Introduction

1,3-Dienes are one of the most important structural motifs frequently found in many natural products and biologically active compounds^1,2^, as well as serve as valuable building blocks for diverse transformations in organic synthesis^3,4^. Accordingly, the stereoselective synthesis of 1,3-dienes has received paramount attention in organic synthesis^5–7^. Olefinations of carbonyls are widely embraced methods to access 1,3-dienes, while typically leading to *E*/*Z* isomers^8,9^. Alternatively, transition-metal-catalyzed cross-coupling reactions, including Heck couplings^10^, ene-yne couplings^11^, hydrovinylation of alkynes^12^, alkenyl-alkenyl couplings^13–15^, and boroalkenylation of alkynes^16–19^, have emerged as a powerful strategy for the stereoselective synthesis of 1,3-dienes. Despite enabling, these methods generally lead to thermodynamically more stable *E*-isomers, or rely on the use of stereochemically well-defined organometallic agents or electrophiles. To the best of our knowledge, catalytic stereodivergent protocol that would enable both *E*-selective and even more challenging *Z*-selective access of highly substituted 1,3-dienes from one set of substrates remains underexploited^20,21^.

**Fig. 1:**
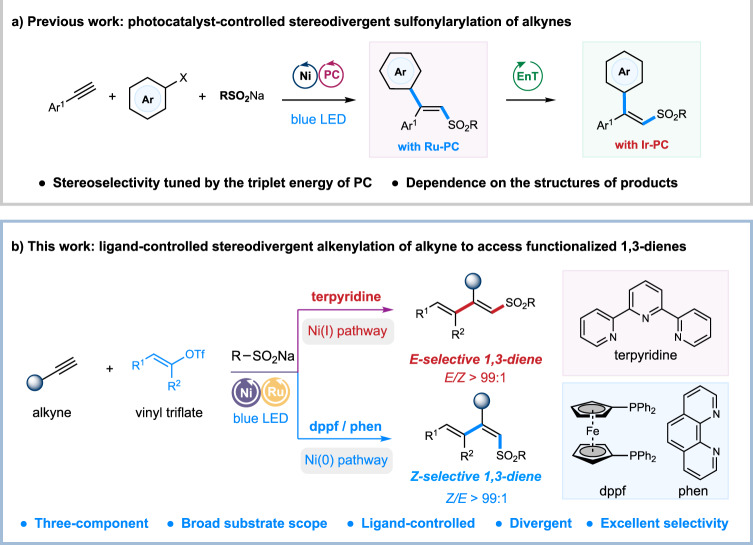
Background and recent work of carbosulfonylation of alkynes. **a** Previous work: photocatalyst-controlled stereodivergent sulfonylarylation of alkynes. **b** This work: ligand-controlled stereodivergent alkenylation of alkynes to access functionalized 1,3-dienes. PC photocatalyst, EnT energy transfer, dppf 1,1-bis(diphenylphosphino)ferrocene, phen 1,10-phenanthroline.

Ni-catalyzed vicinal difunctionalization of alkynes via metalation followed by subsequent functionalization of alkenyl nickel species represents an attractive strategy for the synthesis of substituted alkenes from simple starting materials^22–26^. The highly stereoselective metalation step ensures the selective construction of *syn*-addition products, with a few exceptions of *anti*-selective examples that proceed via substrate-driving *E/Z* isomerization of alkenyl nickel species^27–34^. Recently, radical-involved catalytic 1,2-difunctionalization of alkynes has been disclosed to furnish trisubstituted alkenes with complementary *anti*-selectivity^35–42^. Particularly, utilizing dual nickel/photoredox catalysis^43–47^ has further exploited a number of selective difunctionalization of alkynes with diverse coupling partners under mild conditions^48–56^. Nevertheless, rare examples for stereodivergent synthesis of both *trans*- and *cis*-substituted alkenes from alkynes are reported. Recently, we reported a photoredox/nickel dual catalyzed stereodivergent difunctionalization of alkynes, furnishing both *syn*- and *anti*-selective aryl-substituted alkenes via the judicious choice of photocatalysts with different triplet state energies (Fig. 1a)^57^. Despite attractive, such a *contra*-thermodynamic alkene isomerization strategy relies on the structure of alkenes or photocatalysts. Thus, the development of photoredox/nickel dual-catalyzed divergent method with a complementary stereoselective tuning strategy to access more diverse types of alkenes, such as functionalized *cis- and trans*-1,3-dienes, under mild and operationally simple conditions would be of particular interest.

Here, we show a ligand-controlled stereodivergent alkenyl-functionalization of alkynes with vinyl triflates and sodium sulfinates via photoredox and nickel dual catalysis (Fig. 1b). This strategy furnishes a wide array of synthetically valuable *cis*- and *trans*-sulfonyl-1,3-dienes^58,59^ in one pot from simple starting materials. Detailed mechanistic experiments and computational investigations offer insights into the origins of the observed stereoselectivity and the role of dynamic ligand exchange in the dual ligand system.

## Results and discussion

### Reaction optimizations

Our investigations were started with phenylacetylene **1**, vinyl triflate **2**, and sodium *p*-tolylsulfinate **3** as model substrates (Table 1). After some experimentations, we found that in the presence of Ru(dtbbpy)_3_(PF_6_)_2_ as the photocatalyst, Ni(OAc)_2_•4H_2_O as the nickel catalyst, and 1,10-phenanthroline (1,10-phen) as the ligand, irradiation of the reaction mixture in DMF with blue LEDs gave the sulfonylated diene product **4b** in 45% yield with excellent regio- and *anti*-selectivity (entry 1). Pleasingly, employing terpyridine (tpy) as the ligand dramatically improved the yield of product **4b** to 86% without observation of *syn*-selective isomer (entry 2). Further evaluation of nickel catalysts or pre-catalysts disclosed that Ni(OAc)_2_•4H_2_O was the optimal catalyst for this *anti-*selective transformation (entries 3-6). During this process, we noticed that the nature of nickel salts played an intriguing effect on the *trans/cis* selectivity. Switching to phosphine-ligated nickel pre-catalysts resulted in the formation of a mixture of *trans/cis* isomers **4a** and **4b** in varied yields and *Z/E* ratios (entries 4–6). Interestingly, the use of NiCl_2_•dppf in the absence of terpyridine led to the exclusive formation of *syn*-selective product **4a**, albeit in low yield (entry 7). The addition of 1,10-phen as an exogenous ligand turned out to be beneficial to the yield of **4b**, together with the formation of isomer **4a** (entry 8)^60^. Careful examination of the ratio of nickel and ligand revealed that the use of 20 mol% NiCl_2_•dppf with 10 mol% 1,10-phen furnished **4a** in 80% yield with excellent *cis/trans* selectivity (entries 8–11). Finally, control experiments disclosed that photocatalyst, nickel catalyst, and light were all essential for this stereodivergent reaction (entries 12–14) (see Supplementary Information for more optimization details).

**Table 1:** Optimization of reaction conditions^a^


entry	nickel catalyst	ligand	yield of 4b	yield of 4a
1	Ni(OAc)_2_•4H_2_O	1,10-phen	45%	0%
2	Ni(OAc)_2_•4H_2_O	tpy	86%	0%
3	NiCl_2_	tpy	68%	0%
4	NiCl_2_•dppe	tpy	10%	2%
5	NiCl_2_(PPh_3_)_2_	tpy	31%	16%
6	NiCl_2_•dppf	tpy	28%	10%
7	NiCl_2_•dppf	–	0%	32%
8	NiCl_2_•dppf	1,10-phen	15%	51%
9	NiCl_2_•dppf	1,10-phen (15 mol%)	13%	46%
10	NiCl_2_•dppf (15 mol%)	1,10-phen	5%	53%
11	NiCl_2_•dppf (20 mol%)	1,10-phen	0%	80%
12	w/o PC		0%	0%
13	w/o light		0%	0%
14	w/o nickel		0%	0%

^a^Reactions conditions: Ru(dtbbpy)_3_(PF_6_)_2_ (1 mol%), [Ni] (10 mol%), ligand (10 mol%), alkyne **1** (1.5 equiv.), vinyl triflate **2** (0.1 mmol), TsNa **3** (1.5 equiv.), DMF [0.04 M], 35 ^o^C, blue LED, 6 h. Yields were determined by ^1^H-NMR using 1,3-benzodioxole as an internal standard. *Z/E* ratios were determined by ^1^H-NMR. tpy = terpyridine; 1,10-phen = 1,10-phenanthroline; dppf = 1,1’-bis(diphenyphosphino)ferrocene.

### Substrate scope studies

**Fig. 2:**
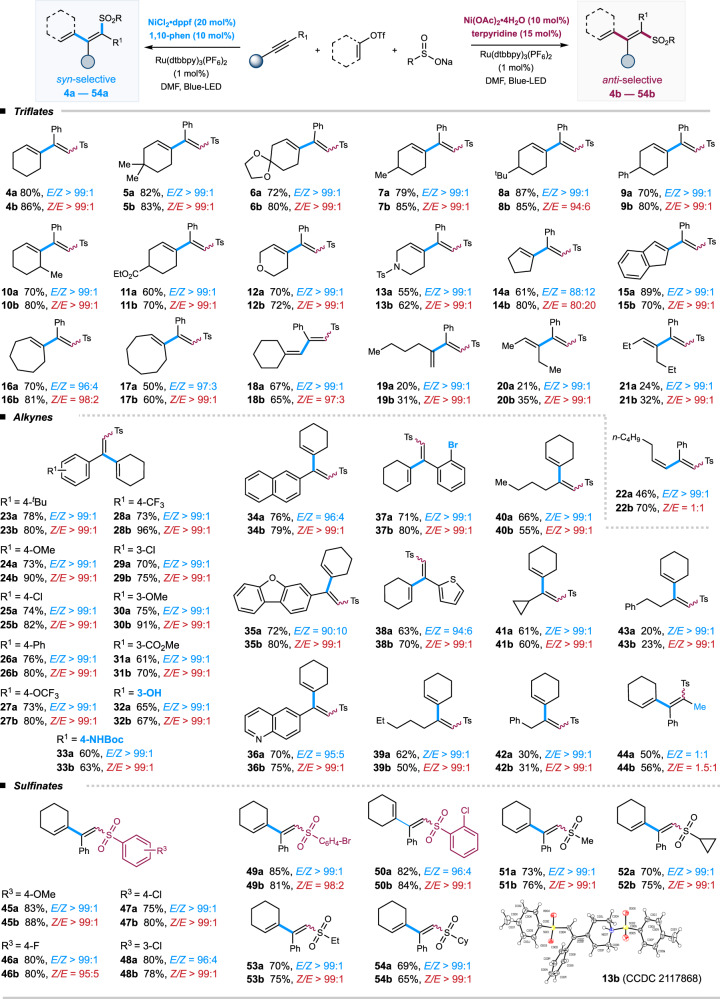
Substrate Scope. Reaction conditions: Ru(dtbbpy)_3_(PF_6_)_2_ (1 mol%), Ni(OAc)_2_•4H_2_O/tpy (for *anti*-selective), NiCl_2_•dppf/1,10-phen (for *syn*-selective), alkyne (1.5 equiv.), vinyl triflate (0.1 mmol), sodium sulfinate (1.5 equiv.), DMF [0.04 M], 35 ^o^C, blue LED, 6 h. Isolated yields. Ts = toluenesulfonyl.

With the optimal reaction conditions in hand, we began to investigate the substrate compatibility of this stereodivergent protocol in the presence of Ru(dtbbpy)_3_(PF_6_)_2_ with two sets of nickel catalysts (entries 2 and 11, respectively). As shown in Fig. 2, a series of cyclic vinyl triflates smoothly underwent cross-couplings with phenyl acetylene **1** and TsNa **3**, furnishing 1,3-dienyl sulfones with high efficiency and excellent *Z* or *E*-selectivity (**4a**–**18a** & **4b**–**18b**). Cyclohexenyl triflates with substituents on 4-position, including alkyl, aryl, ketal, and ester, were well compatible; interestingly, *ortho*-substituents turned out to be well-tolerated with any deleterious effect to the coupling yield and *cis/trans* selectivity (**10a** & **10b**). Moreover, vinyl triflates derived from heteroatom-incorporated cyclic ketones, represented by tetrahydro-4*H*-pyran-4-one and 4-piperidinone, were competent coupling partners, affording corresponding dienes with excellent selectivity (**12a**–**13a** & **12b**–**13b**). The reaction of cyclic vinyl triflates that were prepared from 5-, 7- and 8-membered cyclic ketones proceeded smoothly, albeit with slightly decreased *E/Z* selectivity in the case of 5-membered vinyl triflate (**14a**–**17a** & **14b**–**17b**). Pleasingly, acyclic vinyl triflates that derived from aliphatic ketones were suitable substrates, furnishing the diene products with excellent selectivity yet decreased yields (**18a-21a** & **18b-21b**). The reactions with 1,2-disubstituted vinyl triflates produced the desired coupling products with excellent *syn-*selectivity yet low *anti-*selectivity (**22a** & **22b**). It should be noted that the structures of products **13a** and **13b** are validated by single-crystal X-ray diffraction and the stereoselectivity of the two isomers of products **4a** & **4b**, **13** & **13b**, **22a** & **22b**, **25a** & **25b**, and **53a** & **53b** is further confirmed by COSEY and NOSEY. The stereoselectivity of other products is assigned by analogy.

Next, we turned our attention to the scope of the alkyne component (Fig. 2). A wide range of aryl alkynes, bearing electron-donating or withdrawing substituents on the *ortho-*, *meta*-, or *para*-positions of aromatic rings, all underwent efficient couplings to furnish sulfonated 1,3-dienes with excellent stereoselectivity (**23a**–**37a** & **23b**–**37b**). The mild conditions were compatible with diverse functional groups, including esters, halides, phenols, and amines, leaving synthetic handles for further potential manipulations. Acetylenes incorporated with a heteroarene, represented by quinoline, thiophene, and dibenzo[*b,d*]furan, all worked well with high efficiency (**35a**–**36a, 38a** & **35b**–**36b, 38b**). Besides, aliphatic terminal alkynes were suitable substrates, affording functionalized 1,3-dienes in decreased yields yet with excellent *Z*/*E* selectivity (**39a**–**43a** & **39b**–**43b**). Nevertheless, the reaction of internal alkynes only proceeded with moderate *trans/cis* selectivity under these two reaction conditions (**44a** & **44b**). Last, a number of sodium alkyl sulfinates and substituted aryl sulfinates all participated in the dual catalytic divergent protocol smoothly, delivering the desired sulfonyl-1,3-diene products with high yields and excellent regio- and *syn/anti*-selectivity (**45a**–**54a** & **45b**–**54b**).

**Fig. 3:**
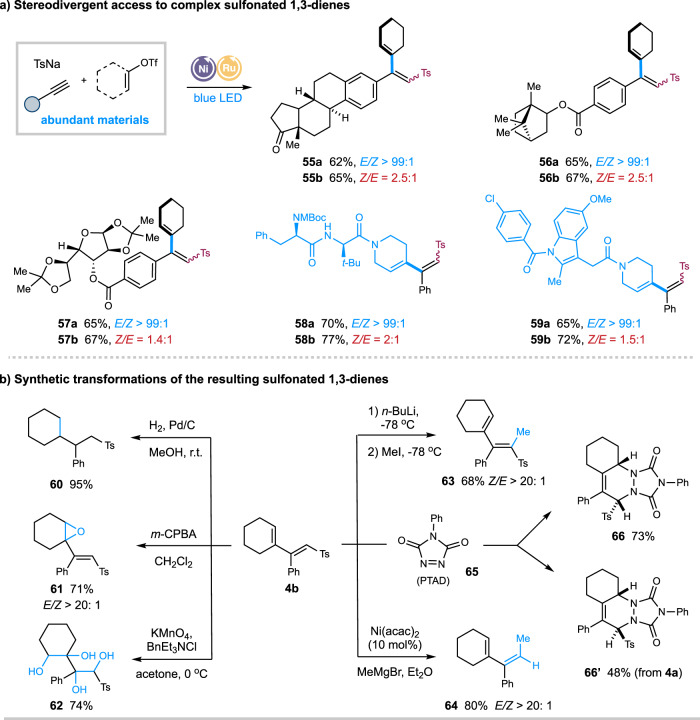
Synthetic applications. **a** Stereodivergent access to complex sulfonyl 1,3-dienes; **b** Synthetic transformations of sulfonyl 1,3-dienes. *m*-CPBA = *m*-chloroperoxybenzoic acid.

To further demonstrate the synthetic applicability of this dual-protocol, late-stage modifications of complex molecules have been evaluated (Fig. 3a). Under the *syn*-selective conditions (entry 11, Table 1), the reaction of complex terminal alkynes or vinyl triflates, derived from estrone, indomethacin (anti-inflammatory), glucose, borneol, and amino acid, proceeded smoothly to afford the desired *E*-1,3-dienes with high yields and excellent stereoselectivity (**55a**–**59a**). Under the *anti*-selective condition (entry 2, Table 1), nevertheless, reactions with these complex substrates proceeded in comparable yields yet moderate stereoselectivity, probably due to the significant steric hindrance of these complex substrates (**55b**–**59b**). Moreover, the resulting sulfonyl 1,3-dienes are useful building blocks in organic synthesis (Fig. 3b). Hydrogenation of **4b** in the presence of palladium on carbon (Pd/C) and H_2_ gave alkyl sulfone **60** in 95% yield. Oxidation of **4b** with *m*-CPBA or KMnO_4_ yielded epoxide **61** and polyol **62**, respectively. Furthermore, treatment of **4b** with *n*-butyllithium, followed by the addition of MeI, afforded 68% yield of (*Z*)-tetra-substituted sulfonyl alkene **63**, the stereoselective synthesis of which remains challenging. Cross-coupling of **4b** with methylmagnesium bromide in the presence of catalytic Ni(acac)_2_ furnished (*E*)-tri-substituted 1,3-dienes **64** in 78% yield and excellent stereoselectivity. Finally, cycloaddition reactions of **4a** or **4b** with 4-phenyl-1,2,4-triazoline-3,5-dione (PTAD) **65** resulted in the formation of the two stereoisomers of products **66** and **66’** in moderate yields.

### Mechanistic studies

**Fig. 4:**
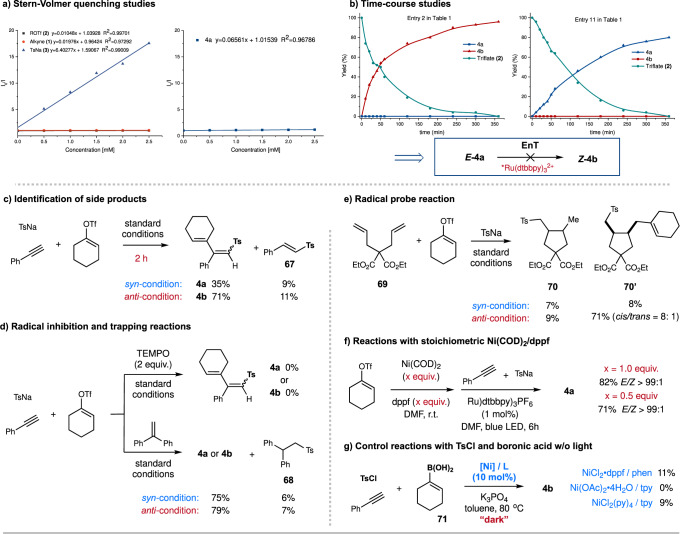
Mechanistic studies. **a** Stern-Volmer quenching studies; **b** Time-course studies; **c** Identification of side products; **d** Radical inhibition and trapping reactions; **e** Radical probe reaction; **f** Reaction with stoichiometric Ni(COD)_2_/dppf; **g** Control reactions with TsCl and boronic acid w/o light. EnT = Energy transfer.

To gain insights into the potential mechanism, we have performed some preliminary mechanistic experiments (Fig. 4). On the one hand, we conducted a number of experiments regarding the photocatalytic part. Stern-Volmer fluorescence quenching studies indicated that the photoexcited *Ru(dtbbpy)_3_^2+^ was quenched by TsNa, other than alkyne **1** or vinyl triflate **2** (Fig. 4a). Light on/off experiments under both *anti*- and *syn-*selective conditions were performed, which showed that the desired couplings ceased in the dark (See Supplementary Figure 6–7); additionally, quantum yields (ϕ) of both *anti*- and *syn*-selective reactions were determined to be less than 1 (See Supplementary Information)^61^. These results ruled out the possibility of a radical chain pathway in this photochemical process. Furthermore, time-course studies of template reactions (phenylacetylene **1**, vinyl triflate **2**, and TsNa **3**) showed that *anti/syn*-selectivity of products **4a** and **4b** remained steady under the conditions shown in entries 2 or 11 (Fig. 4b). Additionally, no significant fluorescence quenching effect was observed between *E*-**4a** and photoexcited *Ru(dtbbpy)_3_^2+^ (E_T_ ≈ 46 kcal/mol)^57^ (Fig. 4a). These results precluded the involvement of photocatalytic *E*→*Z* isomerization of sulfonyl 1,3-dienes, thus further supporting ligand control of stereoselectivity in this catalytic system^57^. On the other hand, we detected a small amount of side product ***E*-51** with careful monitoring of this reaction, implying that this reaction could proceed via sulfonyl addition followed by alkenylation (Fig. 4c). Then, radical inhibition and probe reactions were performed (Figs. 4d-4e). The addition of 2,2,6,6-tetramethyl-1-piperidinyloxy (TEMPO) into the standard systems (entries 1 and 11, Table 1) completely shut down the desired reactions, while the addition of 1,1-diphenylethylene gave the desired products **4a**/**4b** in slightly decreased yields, together with a small amount of alkyl sulfone adduct **68** (Fig. 4d). Reaction of vinyl triflate **2** and TsNa with 1,5-diene **69**, instead of alkyne **1**, gave a small amount of tosylation/cyclization product **70** and tosylation/cyclization/alkenylation product **70’** under the *syn*-selective condition; interestingly, the parallel reaction under the *anti*-selective condition resulted in the formation of 9% yield of **70**, together with 71% yield of **70’** (Fig. 4e). These results implied that different reaction pathways could be involved in *syn-* and *anti-*selective systems. Additionally, control reactions with stoichiometric Ni(cod)_2_ and dppf in the absence of 1,10-phen gave good yields of product **4a** (*Z/E* > 99:1), in contrast to the results with catalytic Ni(cod)_2_/dppf (entry 7, Table 1), suggesting the importance of a synergistic effect with the two ligands (Fig. 4e)^62^. Furthermore, the reaction of alkyne **1**, alkenyl boronic acid **71**, and TsCl with catalytic NiCl_2_(py)_4_/tpy or NiCl_2_•dppf/phen gave a small amount of *anti*-selective diene **4b** under thermal conditions^35^, demonstrating the intriguing role of light irradiation in this stereodivergent alkenylation and further highlighting the synthetic advantage of this photochemical dual-protocol.

**Fig. 5:**
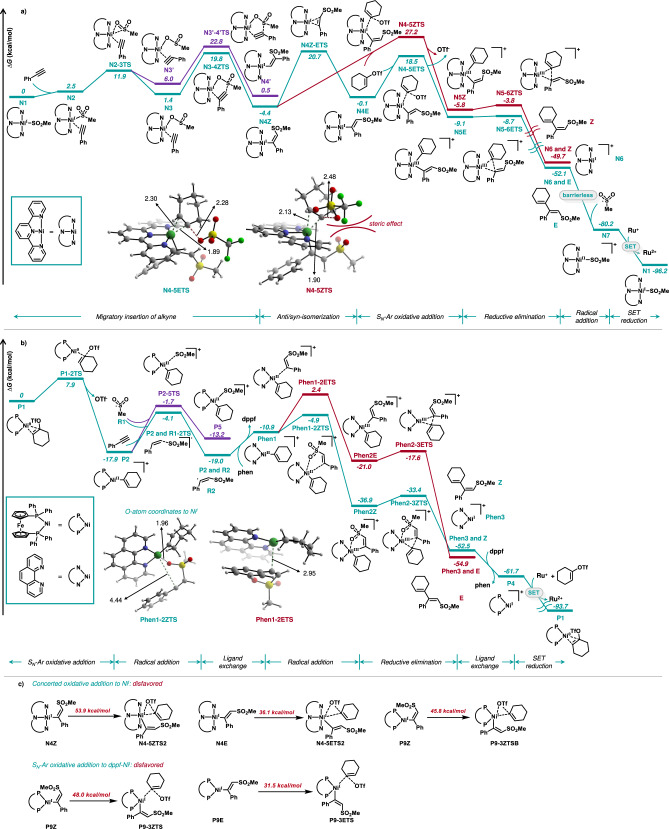
DFT calculations. **a** DFT-computed energy profiles for the sulfonylalkenylation with terpyridine ligand. **b** DFT-computed energy profiles for the sulfonylalkenylation with dppf and phenanthroline ligand. **c** Energy barriers of concerted oxidative addition to Ni^I^ and S_N_-Ar oxidative addition to Ni^I^ with dppf ligand. Free energies in solution (in kcal·mol^-1^) were calculated at SMD (DMF)-M06/Def2-TZVPP//PBE-D3(BJ)/Def2-TZVP (Ni, Fe, Ru)/Def2-SVP (other atoms). DFT-optimized geometries of selected transition states or intermediates are shown. Bond lengths are in Å.

Next, we performed density functional theory (DFT) calculations to gain a deeper understanding of the mechanism and catalytic cycle, particularly to rationalize the stereoselectivity controlled by the different ligands (Computational details are given in the supporting information) (Fig. 5). We chose **51a**/**b** as the model substrate with terpyridine and dppf/phenanthroline as the model ligands for the *anti*/*syn* selective conditions, respectively (Table 1, entries 3 and 11). In Fig. 5a, the Ni-catalytic cycle with terpyridine as the ligand begins with the Ni^I^-SO_2_Me intermediate **N1**^57^. First, phenylacetylene **1** coordinates to the intermediate **N1** and produces **N2**, followed by the rearrangement of the SO_2_Me moiety from a Ni-S to Ni-O bonding mode with an energy barrier of 11.9 kcal/mol (**N2-3TS**). Two intermediates are formed in which the sulfonyl group is in a suitable orientation to either attack the terminal (**N3**) or internal (**N3’**) carbon of the alkyne. The sulfonyl group then migrates to the terminal carbon of the alkyne via a five-membered ring transition state (**N3-4ZTS**) with an energy barrier of 18.4 kcal/mol. In contrast, the migratory insertion into the internal carbon is disfavored by 3.0 kcal/mol (**N3’-4’TS**), which is consistent with the observed regioselectivity of this reaction. In the intermediate **N4Z**, the phenyl and sulfonyl moieties are *anti*-oriented after migratory insertion. Thus, it can either undergo direct S_N_-Ar type of oxidative addition of vinyl triflate **2** via transition state **N4-5ZTS**, with an energy barrier of 31.6 kcal/mol, resulting in the *syn*-selective product **51b**, or undergo *anti*/*syn* isomerization followed by oxidative addition. The C-C bond rotates during the *anti*/*syn* isomerization with an activation energy of 25.1 kcal/mol, leading to the intermediate **N4E**. The S_N_-Ar-type oxidative addition of vinyl triflate to **N4E** has an energy barrier of only 18.6 kcal/mol (**N4-5ETS**), which lies 8.7 kcal/mol below the transition state **N4-5ZTS**. The proximity of the sulfonyl and OTf groups in the transition state **N4-5ZTS** may account for the high oxidative addition energy barrier. A comparison of the two oxidative addition routes implies that the formation of *anti*-selective product **E** is preferred since the transition state **N4Z-ETS** is favored by 6.5 kcal/mol over **N4-5ZTS**, which explains the *anti*-stereoselectivity when terpyridine was used as the ligand. After the reductive elimination, the cross-coupling product is formed, together with the generation of the Ni^I^ intermediate **N6**. The reduction of **N6** to Ni^0^ intermediate **N8** by the Ru-photocatalyst is calculated to be thermodynamically disfavored (+2.2 kcal/mol, see Supplementary Figure 19). In contrast, the radical addition of sulfonyl radical to **N6** is barrierless, with an energy gain of 28.1 kcal/mol^63,64^. The generated Ni^II^ intermediate **N7** can then be reduced by the Ru-photocatalyst and start the next catalytic cycle.

Subsequently, the reaction with a dual ligand system (dppf and phenanthroline) was explored, and the catalytic cycle is depicted in Fig. 5b. Based on a recent report^62^, we proposed that the dynamic ligand exchanges on Ni intermediates promote different steps in the *syn*-selective catalytic cycle. The phosphine ligand dppf facilitates the Ni^I^ reduction and Ni^0^ oxidative addition steps, while the phenanthroline ligand facilitates the radical addition step. The binding of vinyl triflate **2** to dppf-Ni^0^ forms the intermediate **P1**, which undergoes S_N_-Ar-type oxidative addition to afford the Ni^II^ cation **P2** with an energy barrier of only 7.9 kcal/mol^65,66^. Next, the sulfonyl radical **R1** can either add to **P2** via the transition state **P2-5TS** with a 16.2 kcal/mol barrier, leading to **P5**, or add to the alkyne, forming the vinyl radical **R2**, with a barrier of 13.8 kcal/mol. The radical addition to an alkyne is kinetically and thermodynamically favored. The Ni^II^ cation **P2** then undergoes ligand exchange with phenanthroline to afford Ni^II^ intermediate **Phen1**. The vinyl radical **R2** undergoes radical addition with two distinct orientations to the Ni^II^ cation **Phen1**, resulting in an *anti-*or *syn-*selective Ni^III^ intermediate (**Phen2E** or **Phen2Z**). The transition state of the *syn*-selective radical addition is 14.1 kcal/mol **(Phen1-2ZTS**), favored by 7.3 kcal/mol over the *anti*-selective radical addition (**Phen1-2ETS**), which may be due to the coordination of the oxygen atom from the sulfonyl group to the Ni^II^ metal center. Also, the *syn*-selective Ni^III^ intermediate **Phen2Z** is more stable than the *anti*-selective Ni^III^ intermediate **Phen2E** (15.9 kcal/mol difference in energy). With a barrier of only 3.5 kcal/mol, the reductive elimination of **Phen2Z** is rapid, yielding the *syn*-selective product. The resulting Ni^I^ cation **Phen3** again undergoes ligand exchange with dppf to afford the dppf-Ni^I^ intermediate **P4**. In contrast to the tpy-Ni^I^ intermediate **N6**, the intermediate **P4** can easily be reduced by the Ru-photocatalyst, leading to the formation of Ni^0^ intermediate **P1** with an energy gain of 32.0 kcal/mol. The concerted oxidative addition to Ni^I^ intermediate via a three-membered ring transition state was calculated to be disfavored. In addition, the S_N_-Ar type oxidative addition to the dppf-Ni^I^ intermediate is not feasible at room temperature for both *anti*- and *syn*-selective pathways (Fig. 5c). Given that 32% *syn*-selective product was obtained when only dppf was used as the ligand (see Table 1, entry 7), the *syn*-selective catalytic cycle with only dppf as the ligand was also explored (see Supplementary Figure 15 for the energy profile). The stereoselectivity can also be explained by the 19.1 kcal/mol energy difference between the *anti/syn* radical addition step (**P2-3ZTS** *vs*. **P2-3ETS**). Alternatively, another catalytic pathway involving the migratory insertion of alkyne to dppf-ligated vinyl-Ni species was investigated, in which the regio- and stereoselectivity can also be explained (See Supplementary Figure 16 for the energy profile). However, this pathway was not chosen as the main catalytic pathway due to the observed hydrosulfonylation by-product **51** (Fig. 4c), which cannot be generated by this pathway.

**Fig. 6:**
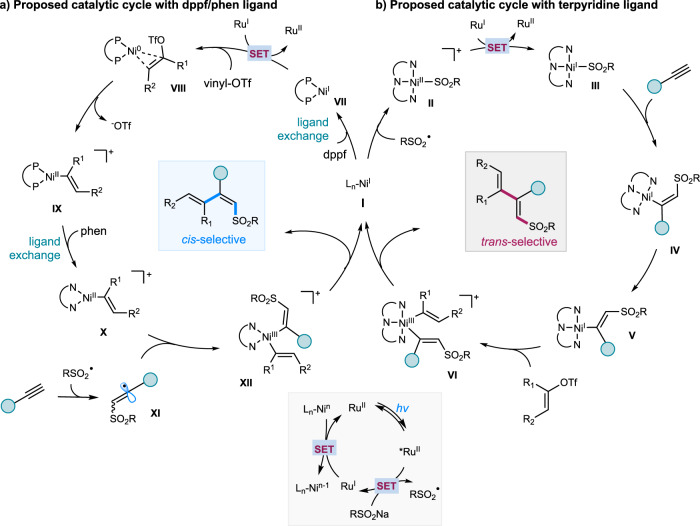
Proposed reaction pathways. **a** Proposed catalytic cycle with dppf/phen ligand. **b** Proposed catalytic cycle with terpydine ligand. dppf = 1,1-bis(diphenylphosphino)ferrocene; phen = 1,10-phenanthroline. *hv* = visible light. SET = single electron transfer.

Based on these experimental and computational results, we propose a plausible reaction pathway as depicted in Fig. 6. Upon light excitation, photoexcited *Ru(dtbbpy)_3_^2+^ (*E*_1/2_^*ox^ = +0.81 V vs. SCE)^67,68^ interacts with RSO_2_Na (TsNa, *E*^red^ = + 0.45 V versus SCE in CH_3_CN)^69^ to release sulfonyl radical and the reducing Ru(I). In the case of terpyridine as ligand, the sulfonyl radical is trapped by Ni(I) **I** to give RSO_2_-Ni(II) **II**^57^, which is then single-electron reduced by Ru(I) to generate RSO_2_-Ni(I) **II**. Migratory insertion of **II** into alkyne **III** regioselectively delivers *cis*-alkenylNi(I) species **IV**, followed by isomerization, to afford the *trans*-alkenylNi(I) **V**. S_N_-Ar type oxidative addition of **V** with vinyl triflate affords *trans*-Ni(III) **VI**, followed by reductive elimination, to furnish *anti*-addition dienes. In the case of NiCl_2_•dppf/phen, which is more electron-rich and less sterically hindered compared to NiCl_2_(py)_4_/tpy, dppf-ligated Ni(I) **VII** is more prone to a SET reduction by Ru(I) to generate Ni(0) species, followed by binding and facile S_N_-Ar type oxidative addition with vinyl triflate to form dppf-ligated alkenylNi(II) **IX**. Then, **IX** undergoes ligand exchange with 1,10-phen to form phen-ligated alkenylNi(II) **X**. At the same time, the sulfonyl radical adds to alkyne to form vinyl radical **XI**, which is subsequently captured by phen-ligated alkenylNi(II) **X** to generate the more stable *cis*-Ni(III) species **XII**. Reductive elimination of Ni(III) **XII** furnishes *syn*-selective product as well as Ni(I) **I**, the latter of which undergoes ligand exchange with dppf to yield (dppf)Ni(I) **VII**. Finally, Ru(I) (Ru(dtbbpy)_3_^+^, *E*_1/2_^II/I^ = −1.45 V vs SCE)^67^ is feasible to reduce (dppf)Ni(I) **VII** or (terpy)Ni(II) **II** [*E*_1/2_ (Ni^II^/Ni^0^) = −1.2 V versus SCE in DMF]^70^ to regenerate the ground state Ru(dtbbpy)_3_^2+^ and close the two catalytic cycles.

In summary, we have developed a dual photoredox and nickel catalyzed stereodivergent three-component alkenyl-functionalization of alkynes with vinyl triflates and sodium sulfinates under mild and operationally simple conditions. Such a photochemical dual strategy enables divergent and straightforward access to *syn-* and *anti*-selective sulfonyl-1,3-dienes by a simple choice of nickel catalyst and ligand without the reliance on the structures of photocatalyst and alkene products. This method demonstrates broad substrate scope and excellent chemo-, regio-, and stereo-selectivity, with potential applications in late-stage functionalizations. A series of mechanistic experiments, including Stern-Volmer fluorescence quenching studies, light on/off experiments, determination of quantum yields, radical probe reactions, and time course studies, as well as detailed computational investigations, offer insights into the origins of the observed stereoselectivity controlled by simple ligands.

## Methods

### General procedure for the cis-selective alkenylation

To a flame-dried 8 mL reaction vial equipped with a magnetic stir bar was charged with Ru(dtbbpy)_3_(PF_6_)_2_ (1 mol %), NiCl_2_•dppf (20 mol %), 1,10-phenanthrane (10 mol %), sulfinate (1.5 equiv.), and DMF [0.04 M]. The reaction mixture was degassed by nitrogen sparging for 30 min, followed by the addition of vinyl triflate (0.10 mmol) and alkyne (1.5 equiv). The reaction mixture was irradiated with blue LEDs for 6 h (around 35 °C, with a cooling fan placed on the top of the vial). The reaction mixture was quenched with water and extracted with ethyl acetate. The combined organic layers were dried with MgSO_4_, filtered and concentrated in vacuo. The crude material was purified by flash chromatography (silica gel, petroleum ether/ ethyl acetate) to afford the products.

### General procedures for the trans-selective alkenylation

To a flame-dried 8 mL reaction vial equipped with a magnetic stir bar was charged with Ru(dtbbpy)_3_(PF_6_)_2_ (1 mol %), Ni(OAc)_2_• 4H_2_O (10 mol %), terpyridine (10 mol %), sulfinate (1.5 equiv.) and DMF [0.04 M]. The reaction mixture was degassed by nitrogen sparging for 30 min, followed by the addition of vinyl triflate (0.10 mmol) and alkyne (1.5 equiv.). Then the reaction mixture was irradiated with blue LEDs for 6 h (around 35 °C). The reaction mixture was quenched with water and extracted with ethyl acetate. The combined organic layers were dried with MgSO_4_, filtered and concentrated in vacuo. The crude material was purified by flash chromatography (silica gel, petroleum ether/ ethyl acetate) to afford the products.

## Supplementary information

Supplementary Information

Description of Additional Supplementary Files

Supplementary Data 1

## Data Availability

The data supporting the findings of this study are available within the paper and its Supplementary Information. Supplementary Data file 1 contains the cartesian coordinates of the calculated structures. The crystallographic data for the structures reported in this Article have been deposited at the Cambridge Crystallographic Data Centre, under deposition numbers CCDC 2117868 and 2117869. Copies of the data can be obtained free of charge via https://www.ccdc.cam.ac.uk/structures/.
