# Supplementary material for: Ligand-controlled stereodivergent alkenylation of alkynes to access functionalized trans- and cis-1,3-dienes
Source: Nat Commun. 2023 Jan 4;14:55. doi: 10.1038/s41467-022-35688-2 (PMC9813127; doi:10.1038/s41467-022-35688-2)
Supplement: Supplementary file 1 — Supplementary Information [file 41467_2022_35688_MOESM1_ESM.pdf]

Supplementary Information for

Ligand-Controlled Stereodivergent Alkenylation of Alkynes to Access Functionalized *trans*- and *cis*-1,3-Dienes

Tianyu Long,[1,†] Chen Zhu,[2,†] Ling Li,[1] Liang Shao,[1] Shengqing Zhu,[1] Magnus Rueping,[2,*] and Lingling Chu[1,*]

[1] State Key Laboratory for Modification of Chemical Fibers and Polymer Materials, Center for Advanced Low- Dimension Materials, Donghua University, College of Chemistry and Chemical Engineering, Shanghai 201620, China.
[2] King Abdullah University of Science and Technology (KAUST), KAUST Catalysis Center (KCC), Thuwal, 23955-6900 Saudi Arabia.
[†] These authors contributed equally.
[*] E-mail: lingling.chu1@dhu.edu.cn; magnus.rueping@kaust.edu.sa.

Contents

Supplementary Methods 1
- 1.1 General Information 1
- 1.2 General Procedures for Sulfonylalkenylation of Alkynes 2

Supplementary Discussion 3
- 2. 1 Reaction Optimizations 3
- 2. 2 Characterization of Products 9
- 2. 3 Synthetic applicability 74
- 2. 4 Mechanistic Studies 79

Supplementary Notes 99
- 3.1 Crystal structure and X-ray refinement data 99
- 3.2 Computational Methods and Details 117
- 3.3 NMR Spectra 123

Supplementary References 253

Supplementary Methods

1.1 General Information

Commercial reagents were purchased from Aldrich, TCI, Energy Chemical and J&K chemical, and were used as received. All reactions were carried out in oven-dried glassware under an atmosphere of nitrogen unless otherwise noted. Chromatographic purification of products was accomplished by flash chromatography using silica gel. Thin-layer chromatography (TLC) was performed on Silicycle 250 mm silica gel F-254 plates.^{1}H, ^{19}F NMR, and ^{13}C NMR spectra were recorded on Bruker 400 (400, 376, and 100 MHz) and Bruker 600 (600, 564, and 150 MHz), and are internally referenced to residual solvent signals (for $CDCl_3$, 7.26 and 77.0 ppm). Data for ^{1}H NMR and ^{19}F NMR are reported as follows: chemical shift (δ ppm), multiplicity (s = singlet, d = doublet, t = triplet, q = quartet, m = multiplet, br = broad), integration, coupling constant (Hz). ^{13}C spectra were reported as chemical shifts in ppm and multiplicity where appropriate. High resolution mass spectra were obtained at Shanghai Institute of Organic Chemistry mass spectrometry facilities. X–Ray diffraction data was obtained by X–Ray spectrometry facilities at Shanghai Institute of Organic Chemistry. Photochemical experiments have been performed using 90 W LEDs light (λmax = 467 nm, commercialized from WATTCASTM). All known alkynes[1], vinyl triflates[2], and sulfinates were prepared according to the previously reported procedures.

1.2 General Procedures for Sulfonylalkenylation of Alkynes

General procedures A for *cis*-selective alkenylation:

To a flame-dried 8 mL reaction vial equipped with a magnetic stir bar was charged with $Ru(dtbbpy)_3(PF_6)_2$ (0.001 mmol, 1.2 mg, 1 mol %), $NiCl_2$•dppf (0.02 mmol, 13.6 mg, 20 mol %), 1,10-phenanthrane (0.01 mmol, 1.8 mg, 10 mol %), sulfinate (0.15 mmol, 1.5 equiv.), and DMF (2.5 mL, 0.04 M). The reaction mixture was degassed by nitrogen sparging for 30 min, followed by the addition of vinyl triflate (0.10 mmol, 1.0 equiv.) and alkyne (0.15 mmol, 1.5 equiv.). The vial was sealed with Parafilm. Then the reaction mixture was irradiated with blue LEDs for 6 h (around 35 °C, with a cooling fan placed on the top of the vial). The reaction mixture was quenched with water and extracted with ethyl acetate three times. The combined organic layers were dried with $MgSO_4$, filtered and concentrated in vacuo. The crude material was purified by flash chromatography (silica gel, petroleum ether/ ethyl acetate) to afford the products.

General procedures B for *trans*-selective alkenylation:

To a flame-dried 8 mL reaction vial equipped with a magnetic stir bar was charged with $Ru(dtbbpy)_3(PF_6)_2$ (0.001 mmol, 1.2 mg, 1 mol %), $Ni(OAc)_2$• $4H_2O$ (0.01 mmol, 2.5 mg, 10 mol %), terpyridine (0.01 mmol, 2.3 mg, 10 mol %), sulfinate (0.15 mmol, 1.5 equiv.) and DMF (2.5 mL, 0.04 M). The reaction mixture was degassed by nitrogen sparging for 30 min, followed by the addition of vinyl triflate (0.10 mmol, 1.0 equiv.) and alkyne (0.15 mmol, 1.5 equiv.). The vial was sealed with Parafilm. Then the reaction mixture was irradiated with blue LEDs for 6 h (around 35 °C). The reaction mixture was quenched with water and extracted with ethyl acetate three times. The combined organic layers were dried with $MgSO_4$, filtered and concentrated in vacuo. The crude material was purified by flash chromatography (silica gel, petroleum ether/ ethyl acetate) to afford the products.

Supplementary Discussion

2.1 Reaction Optimizations.

Supplementary Table 1. Photocatalyst effect.

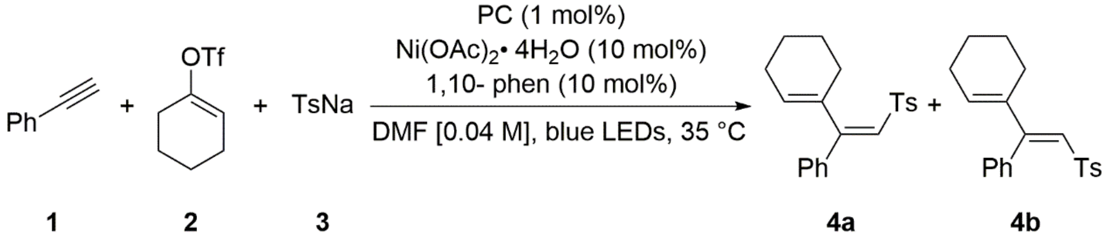

Entry	PC	Yield of 4a (%)	Yield of 4b (%)
1	$Ru(bpy)_3(PF_6)_2$	3	40
2	$Ru(dtbbpy)_3(PF_6)_2$	0	45
3	Eosin Y	0	10
4	4-CzIPN	7	24
5	$Ir[(dFCF_3ppy)_2(dtbbpy)]PF_6$	30	22
6	$Ir[(ppy)_2(dtbbpy)]PF_6$	9	21
7	$Ir(ppy)_3$	16	3
8	$Ru(bpm)_3(Cl)_2$	0	0

Supplementary Table 2. Solvent effect.

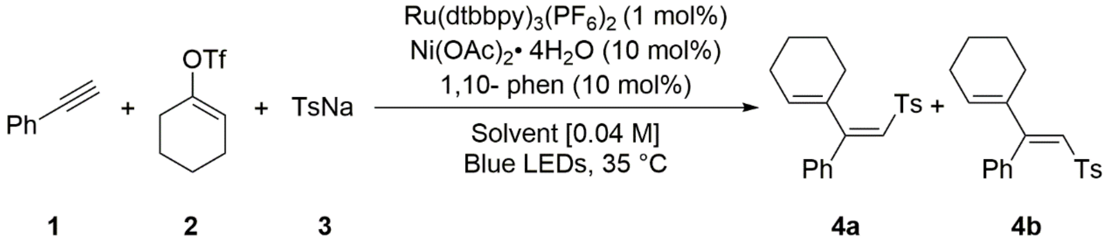

Entry	Solvent	Yield of 4a (%)	Yield of 4b (%)
1	DMF	0	45
2	DMA	0	28
3	NMP	0	32
4	CH_3CN	0	0
5	DCE	0	0
6	DCM	0	0
7	EA	0	0
8	Toluene	0	0

Supplementary Table 3. Nickel Catalyst and ligand effect.

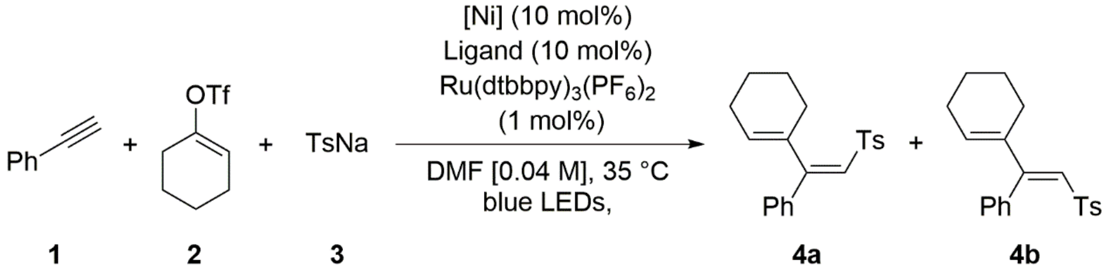

Entry	Nickel Catalyst	ligand	Yield of **4a** (%)	Yield of **4b** (%)
1	$Ni(OAc)_2$• $4H_2O$	1,10-phen	0	45
2	$Ni(OAc)_2$• $4H_2O$	Terpyridine	0	86
3	$NiCl_2$	Terpyridine	0	68
4	$NiCl_2$• dppe	Terpyridine	2	10
5	$NiCl_2(PPh_3)_2$	Terpyridine	16	31
6	$NiCl_2$• dppf	Terpyridine	10	28
7	$NiCl_2$• dppf	--	32	0
8	$NiCl_2$• dppf	1,10-phen	51	15
9	$NiCl_2$• dppf	1,10-phen (15 mol%)	46	13
10	$NiCl_2$• dppf (15 mol%)	1,10-phen	53	5
11	$NiCl_2$• dppf (20 mol%)	1,10-phen	80	0

Supplementary Table 4. Control Experiment.

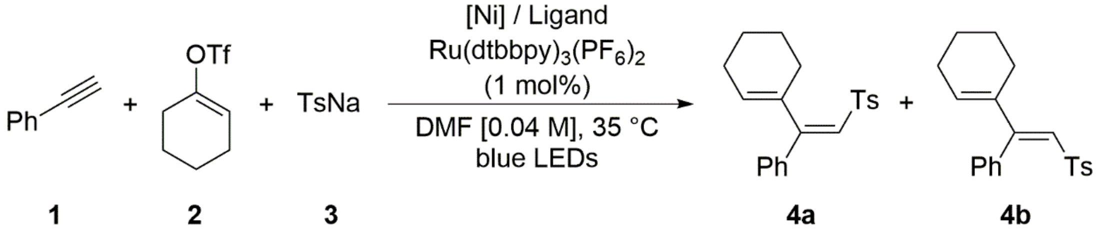

Entry	ligand	Yield of 4a (%)	Yield of 4b (%)
1	$NiCl_2$• dppf / 1,10-phen	80	0
2	w/o $NiCl_2$• dppf	0	0
3	w/o 1,10-phen	35	0
4	w/o PC	0	0
5	w/o light	0	0
6	$Ni(OAc)_2$•$4H_2O$ /terpyridine	0	86
7	w/o $Ni(OAc)_2$• $4H_2O$	0	0
8	w/o Terpyridine	0	0
9	w/o PC	0	0
10	w/o light	0	0

Supplementary Table 5. Photocatalyst screening for *cis*-selective alkenylation.

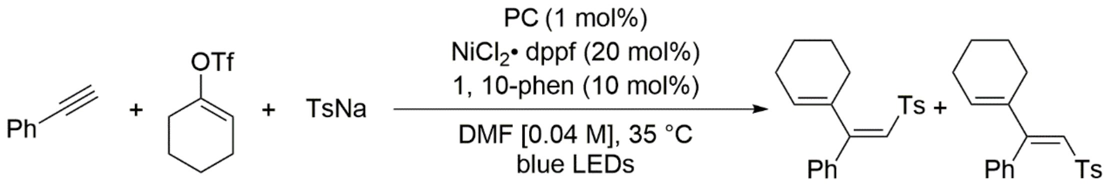

Entry	PC	E_T(kcal/mol)	Yield of 4a (%)	Yield of 4b (%)	Retain of 2 (%)
1	$Ir[(dFCF_3ppy)_2(d\text{-}OMe\text{-}bpy)]PF_6$	60.6	7	0	80
2	$Ir[(dFCF_3ppy)_2dtbbpy]PF_6$	59.4	7	0	80
3	$Ir[(dFppy)_2(phen)]PF_6$	54.0	9	0	75
4	$Ir(ppy)_3$	53.6	7	0	80
5	$Ru(dtbbpy)_3(PF_6)_2$	49.8	80	0	0
6	$Ru(phen)_3Cl_2$• x H_2O	47.0	65	0	0
7	$Ru(bpy)_3(PF_6)_2$	46.8	65	0	0
8	$Ru(phen)_3(PF_6)_2$	46.6	73	0	23
9	$Ir[(ppy)_2(dCO_2Etbpy)]PF_6$	39.7	13	0	75

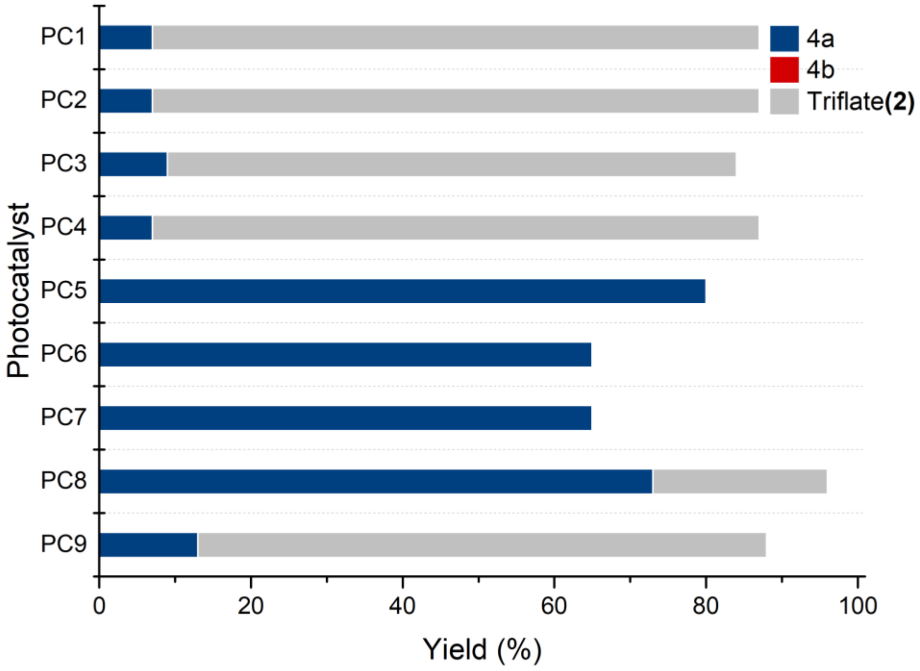

Supplementary Figure 1. Photocatalyst screening for *cis*-selective alkenylation. The bar chart in the figure represents the composition of the reaction mixture using different photocatalyst, the blue part represent the generation of **4a**, the red part represent the generation of **4b**, The grey part represents the remainder of triflate **(2)**.

Supplementary Table 6. Photocatalyst screening for *trans*-selective alkenylation.

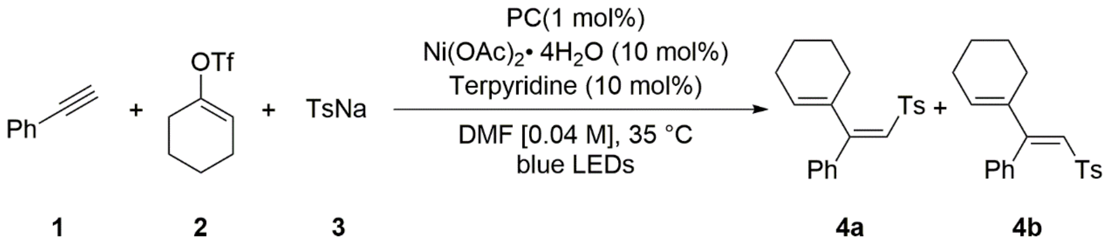

Entry	PC	E_T(kcal/mol)	Yield of 4a (%)	Yield of 4b (%)	Recovery of 2 (%)
1	Ir[(dFCF$_3$ppy)$_2$(d-OMe-bpy)]PF$_6$	60.6	48	17	10
2	Ir[(dFCF$_3$ppy)$_2$dtbbpy]PF$_6$	59.4	50	22	5
3	Ir[(dFppy)$_2$(phen)]PF$_6$	54.0	40	24	10
4	Ir(ppy)$_3$	53.6	45	14	14
5	Ru(dtbbpy)$_3$(PF$_6$)$_2$	49.8	0	86	0
6	Ru(phen)$_3$Cl$_2$•xH$_2$O	47.0	4	68	0
7	Ru(bpy)$_3$(PF$_6$)$_2$	46.8	0	78	0
8	Ru(phen)$_3$(PF$_6$)$_2$	46.6	5	77	0
9	Ir[(ppy)$_2$(dCO$_2$Etbpy)]PF$_6$	39.7	0	20	60

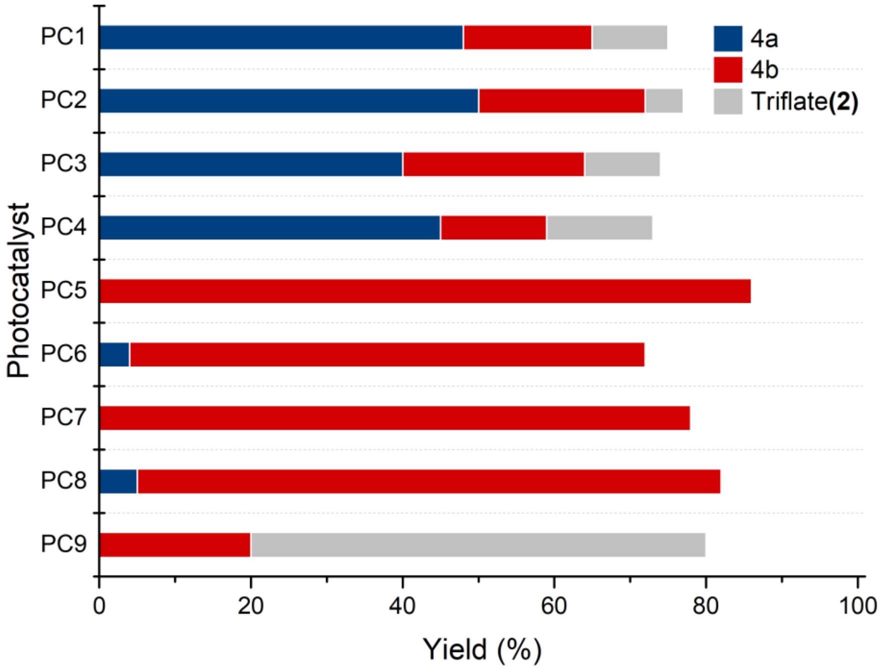

Supplementary Figure 2. Photocatalyst screening for *trans*-selective alkenylation. The bar chart in the figure represents the composition of the reaction mixture using different photocatalyst, the blue part represent the generation of **4a**, the red part represent the generation of **4b**, The grey part represents the remainder of triflate **(2)**.

Supplementary Table 7. Concentration effect for *cis*-selective alkenylation.

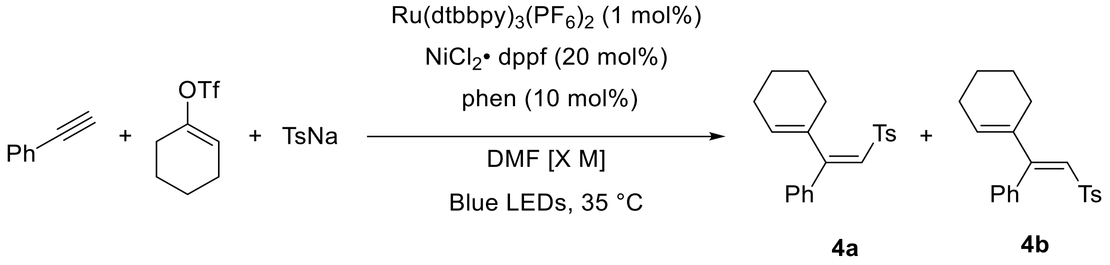

Entry	Concentration	Yield of 4a (%)	Yield of 4b (%)
1	0.04 M	80	0
2	0.05 M	72	0
3	0.1 M	54	0
4	0.2 M	27	0

Supplementary Table 8. Concentration effect for *trans*-selective alkenylation.

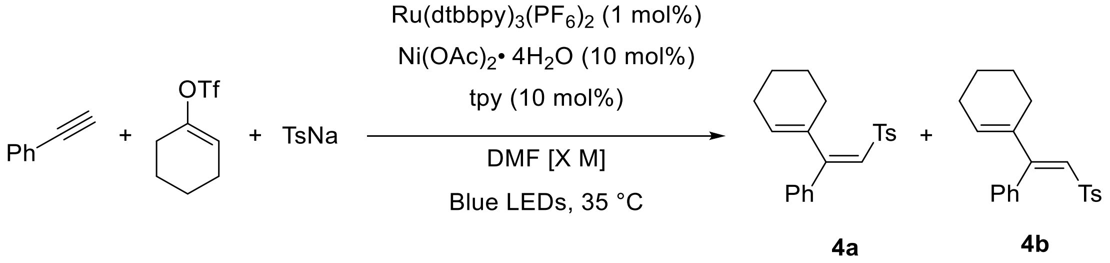

Entry	Concentration	Yield of 4a (%)	Yield of 4b (%)
1	0.04 M	0	86
2	0.05 M	0	82
3	0.1 M	0	81
4	0.2 M	0	70

2.2 Characterization of Products.

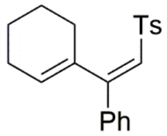

(*E*)-1-((2-(cyclohex-1-en-1-yl)-2-phenylvinyl)sulfonyl)-4-methylbenzene (4a).

According to the general procedure **A**, the crude material was purified by flash chromatography (silica gel, petroleum ether/ ethyl acetate = 5:1) as yellow oil (27.0 mg, 80%) (*E/Z* > 99: 1).

^{1}H NMR (400 MHz, $CDCl_3$) δ 7.71 (d, *J* = 8.2 Hz, 2H), 7.34 – 7.25 (m, 5H), 7.23 (d, *J* = 8.2 Hz, 2H), 6.61 (s, 1H), 5.63 (m, 1H), 2.35 (s, 3H), 2.05 (m, 2H), 1.57 (m, 2H), 1.51 – 1.45 (m, 2H), 1.42 – 1.36 (m, 2H).

^{13}C NMR (100 MHz, $CDCl_3$) δ 158.15, 143.77, 139.72, 136.99, 133.27, 130.93, 130.13, 129.43, 128.75, 127.92, 127.68, 127.51, 27.54, 25.25, 22.07, 21.63, 21.43.

HRMS (ESI) Calcd for $C_{21}H_{22}NaO_2S^+$ $[M+Na]^+$: 361.1233, found: 361.1234.

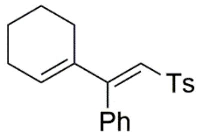

(Z)-1-((2-(cyclohex-1-en-1-yl)-2-phenylvinyl)sulfonyl)-4-methylbenzene (4b).

According to the general procedure **B**, the crude material was purified by flash chromatography (silica gel, petroleum ether/ ethyl acetate = 5:1) as yellow oil (29.1 mg, 86%) (*Z/E* > 99: 1).

^{1}H NMR (400 MHz, $CDCl_3$) δ 7.24 – 7.17 (m, 3H), 7.11 (m, 2H), 7.03 – 6.97 (d, *J* = 8.2 Hz, 2H), 6.84 – 6.75 (d, *J* = 8.2 Hz, 2H), 6.49 (s, 1H), 5.51 (m, 1H), 2.26 (s, 3H), 2.11 (s, 2H), 1.94 (m, 2H), 1.59 (m, 2H), 1.44 (m, 2H).

^{13}C NMR (100 MHz, $CDCl_3$) δ 155.62, 143.37, 139.16, 138.89, 136.79, 134.60, 129.55, 129.18, 127.86, 127.58, 127.33, 126.01, 26.58, 25.55, 22.42, 21.55, 21.54.

HRMS (ESI) Calcd for $C_{21}H_{22}NaO_2S^+$ $[M+Na]^+$: 361.1233, found: 361.1235.

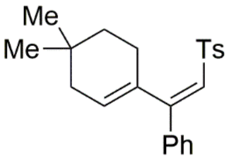

(*E*)-1-((2-(4,4-dimethylcyclohex-1-en-1-yl)-2-phenylvinyl)sulfonyl)-4-methylbenzene (5a).

According to the general procedure **A**, the crude material was purified by flash chromatography (silica gel, petroleum ether/ ethyl acetate = 5:1) as yellow oil (30.0 mg, 82%) (*E/Z* > 99: 1).

^{1}H NMR (400 MHz, $CDCl_3$) δ 7.79 (d, *J* = 8.2 Hz, 2H), 7.40 – 7.33 (m, 5H), 7.31 (d, *J* = 8.2 Hz, 2H), 6.65 (s, 1H), 5.58 – 5.52 (m, 1H), 2.43 (s, 3H), 1.93 (m, 2H), 1.87 – 1.82 (m, 2H), 1.26 (m, 2H), 0.99 (s, 6H).

^{13}C NMR (100 MHz, $CDCl_3$) δ 158.38, 143.82, 140.01, 137.25, 132.16, 130.13, 129.52, 129.46, 128.77, 127.63, 127.45, 127.36, 39.24, 34.99, 28.56, 28.20, 25.73, 21.63.

HRMS (ESI) Calcd for $C_{23}H_{26}NaO_2S^+$ $[M+Na]^+$: 389.1546, found: 389.1546.

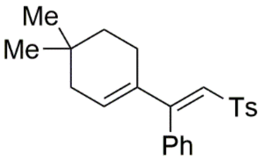

(*Z*)-1-((2-(4,4-dimethylcyclohex-1-en-1-yl)-2-phenylvinyl)sulfonyl)-4-methylbenzene (5b).

According to the general procedure **B**, the crude material was purified by flash chromatography (silica gel, petroleum ether/ ethyl acetate = 5:1) as yellow oil (30.4 mg, 83%) (*Z/E* > 99: 1).

^{1}H NMR (400 MHz, $CDCl_3$) δ 7.32 (d, *J* = 8.2 Hz, 2H), 7.30 – 7.26 (m, 1H), 7.21 (m, 2H), 7.10 (d, *J* = 8.2 Hz, 2H), 6.94 – 6.85 (d, *J* = 8.2 Hz, 2H), 6.59 (s, 1H), 5.52 (m, 1H), 2.37 (s, 3H), 2.22 (m, 2H), 1.84 (m, 2H), 1.46 (m, 2H), 0.88 (s, 6H).

^{13}C NMR (100 MHz, $CDCl_3$) δ 155.27, 143.35, 139.10, 138.04, 135.55, 134.68, 129.52, 129.18, 127.88, 127.63, 127.35, 126.17, 40.46, 35.22, 28.35, 27.98, 23.36, 21.58.

HRMS (ESI) Calcd for $C_{23}H_{26}NaO_2S^+$ $[M+Na]^+$: 389.1546, found: 389.1550.

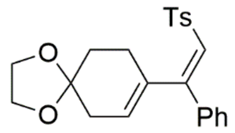

(*E*)-8-(1-phenyl-2-tosylvinyl)-1,4-dioxaspiro[4.5]dec-7-ene (6a).

According to the general procedure **A**, the crude material was purified by flash chromatography (silica gel, petroleum ether/ ethyl acetate = 5:1) as yellow oil (28.5 mg, 72%) (*E/Z* > 99: 1).

^{1}H NMR (400 MHz, $CDCl_3$) δ 7.81 (d, *J* = 8.2 Hz, 2H), 7.46 – 7.34 (m, 5H), 7.31 (d, *J* = 8.2 Hz, 2H), 6.74 (s, 1H), 5.55 (m, 1H), 3.99 (m, 4H), 2.43 (s, 3H), 2.38 (m, 2H), 1.99 (m, 2H), 1.62 (t, *J* = 6.4 Hz, 2H).

^{13}C NMR (100 MHz, $CDCl_3$) δ 157.35, 143.90, 139.72, 136.44, 132.60, 130.31, 129.49, 128.82, 128.01, 127.85, 127.63, 127.51, 107.18, 64.47, 35.92, 30.55, 27.55, 21.61.

HRMS (ESI) Calcd for $C_{23}H_{24}NaO_4S^+$ $[M+Na]^+$: 419.1288, found: 419.1290.

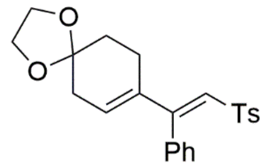

(*Z*)-8-(1-phenyl-2-tosylvinyl)-1,4-dioxaspiro[4.5]dec-7-ene (6b).

According to the general procedure **B**, the crude material was purified by flash chromatography (silica gel, petroleum ether/ ethyl acetate = 5:1) as yellow oil (31.7 mg, 80%) (*Z/E* > 99: 1).

^{1}H NMR (400 MHz, $CDCl_3$) δ 7.30 (m, 3H), 7.21 (m, 2H), 7.10 (d, *J* = 8.2 Hz, 2H), 6.91 (m, 2H), 6.61 (s, 1H), 5.48 (m, 1H), 3.96 (m, 4H), 2.47 (m, 2H), 2.37 (s, 3H), 2.30 (s, 2H), 1.84 (t, *J* = 6.4 Hz, 2H).

^{13}C NMR (100 MHz, $CDCl_3$) δ 154.34, 143.47, 138.97, 136.21, 135.29, 134.32, 129.52, 129.18, 128.00, 127.65, 127.41, 127.23, 107.12, 64.52, 36.78, 30.81, 24.92, 21.54.

HRMS (ESI) Calcd for $C_{23}H_{24}NaO_4S^+$ $[M+Na]^+$: 419.1288, found: 419.1289.

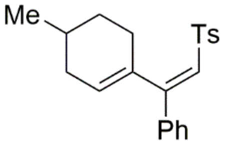

(*E*)-1-methyl-4-((2-(4-methylcyclohex-1-en-1-yl)-2-phenylvinyl)sulfonyl) benzene (7a).

According to the general procedure **A**, the crude material was purified by flash chromatography (silica gel, petroleum ether/ ethyl acetate = 5:1) as yellow oil (27.8 mg, 79%) (*E/Z* > 99: 1).

^{1}H NMR (400 MHz, $CDCl_3$) δ 7.79 (d, *J* = 8.2 Hz, 2H), 7.40 – 7.33 (m, 5H), 7.31 (d, *J* = 8.2 Hz, 2H), 6.68 (s, 1H), 5.69 – 5.61 (m, 1H), 2.43 (s, 3H), 2.27 – 2.18 (m, 1H), 1.80 – 1.63 (m, 4H), 1.60 – 1.52 (m, 1H), 1.13 – 1.04 (m, 1H), 0.98 (d, *J* = 6.4 Hz, 3H).

^{13}C NMR (100 MHz, $CDCl_3$) δ 158.11, 143.80, 139.77, 137.00, 132.99, 130.39, 130.14, 129.45, 128.75, 127.85, 127.58, 127.48, 33.79, 30.33, 27.75, 27.52, 21.79, 21.63.

HRMS (ESI) Calcd for $C_{22}H_{24}NaO_2S^+$ $[M+Na]^+$: 375.1389, found: 375.1387.

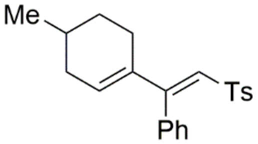

(*Z*)-1-methyl-4-((2-(4-methylcyclohex-1-en-1-yl)-2-phenylvinyl)sulfonyl)benzene (7b).

According to the general procedure **B**, the crude material was purified by flash chromatography (silica gel, petroleum ether/ ethyl acetate = 5:1) as yellow oil (29.9 mg, 85%) (*Z/E* > 99: 1).

^{1}H NMR (400 MHz, $CDCl_3$) δ 7.30 (m, 3H), 7.21 (m, 2H), 7.10 (d, *J* = 8.2 Hz, 2H), 6.92 – 6.86 (m, 2H), 6.57 (s, 1H), 5.58 – 5.53 (m, 1H), 2.36 (s, 3H), 2.29 – 2.14 (m, 2H), 1.88 – 1.77 (m, 1H), 1.63 (m, 2H), 1.26 (m, 2H), 0.93 (d, *J* = 6.4 Hz, 3H).

^{13}C NMR (100 MHz, $CDCl_3$) δ 155.37, 143.33, 139.15, 138.51, 136.52, 134.66, 129.53, 129.16, 127.85, 127.61, 127.34, 126.13, 34.98, 30.62, 27.69, 25.55, 21.56, 21.39.

HRMS (ESI) Calcd for $C_{22}H_{24}NaO_2S^+$ $[M+Na]^+$: 375.1389, found: 375.1388.

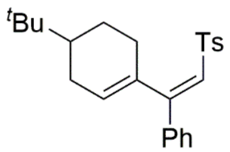

(*E*)-1-((2-(4-(*tert*-butyl)cyclohex-1-en-1-yl)-2-phenylvinyl)sulfonyl)-4-methylbenzene (8a).

According to the general procedure **A**, the crude material was purified by flash chromatography (silica gel, petroleum ether/ ethyl acetate = 5:1) as yellow oil (34.3 mg, 87%) (*E/Z* > 99: 1).

^{1}H NMR (400 MHz, $CDCl_3$) δ 7.79 (d, *J* = 8.2 Hz, 2H), 7.42 – 7.33 (m, 5H), 7.31 (d, *J* = 8.2 Hz, 2H), 6.70 (s, 1H), 5.80 – 5.72 (m, 1H), 2.43 (s, 3H), 2.15 (m, 1H), 1.86 (m, 1H), 1.71 – 1.57 (m, 3H), 1.27 – 1.13 (m, 2H), 0.87 (s, 9H).

^{13}C NMR (100 MHz, $CDCl_3$) δ 157.63, 143.72, 139.51, 137.00, 132.99, 131.67, 130.11, 129.43, 128.74, 127.93, 127.77, 127.55, 43.01, 32.15, 29.12, 27.14, 26.94, 23.38, 21.62.

HRMS (ESI) Calcd for $C_{25}H_{30}NaO_2S^+$ $[M+Na]^+$: 417.1859, found: 417.1859.

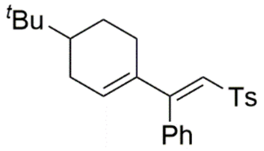

(*Z*)-1-((2-(4-(*tert*-butyl)cyclohex-1-en-1-yl)-2-phenylvinyl)sulfonyl)-4-methylbenzene (8b).

According to the general procedure **B**, the crude material was purified by flash chromatography (silica gel, petroleum ether/ ethyl acetate = 5:1) as yellow oil (35.4 mg, 85%) (*Z/E* = 94: 6). The products are mixtures of *Z/E* isomers and the radio was determined by ^{1}H NMR. The spectrum of *E* isomer is in accord with **8a**, the spectrum of *Z* isomer is as follows.

^{1}H NMR (400 MHz, $CDCl_3$) δ 7.31 (m, 3H), 7.21 (m, 2H), 7.09 (d, *J* = 8.2 Hz, 2H), 6.92 – 6.83 (m, 2H), 6.57 (s, 1H), 5.63 – 5.56 (m, 1H), 2.37 (m, 4H), 2.22 – 2.04 (m, 2H), 1.98 – 1.91 (m, 1H), 1.81 (m, 1H), 1.24 – 1.16 (m, 2H), 0.83 (s, 9H).

^{13}C NMR (100 MHz, $CDCl_3$) δ 155.27, 143.31, 139.42, 139.15, 136.71, 134.65, 129.50, 129.15, 127.84, 127.61, 127.33, 126.03, 43.37, 32.09, 28.26, 27.08, 26.96, 23.86, 21.57.

HRMS (ESI) Calcd for $C_{25}H_{30}NaO_2S^+$ $[M+Na]^+$: 417.1859, found: 417.1859.

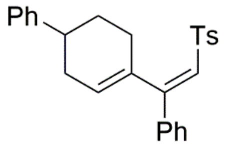

(*E*)-4-(1-phenyl-2-tosylvinyl)-1,2,3,6-tetrahydro-1,1'-biphenyl (9a).

According to the general procedure **A**, the crude material was purified by flash chromatography (silica gel, petroleum ether/ ethyl acetate = 5:1) as yellow oil (29.0 mg, 70%) (*E/Z* > 99: 1).

^{1}H NMR (400 MHz, $CDCl_3$) δ 7.84 (d, *J* = 8.2 Hz, 2H), 7.44 – 7.32 (m, 9H), 7.24 (m, 3H), 6.72 (s, 1H), 5.83 – 5.75 (m, 1H), 2.80 – 2.70 (m, 1H), 2.48 – 2.40 (m, 4H), 2.32 – 2.22 (m, 1H), 1.97 (m, 1H), 1.83 (m, 2H), 1.64 – 1.57 (m, 1H).

^{13}C NMR (100 MHz, $CDCl_3$) δ 157.59, 146.53, 143.93, 139.73, 136.85, 133.24, 130.29, 130.25, 129.55, 128.83, 128.53, 127.87, 127.82, 127.53, 126.85, 126.28, 39.20, 33.55, 29.32, 28.62, 21.67.

HRMS (ESI) Calcd for $C_{27}H_{26}NaO_2S^+$ $[M+Na]^+$: 437.1546, found: 437.1547.

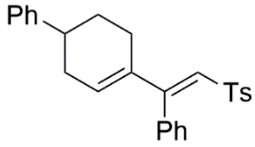

(*Z*)-4-(1-phenyl-2-tosylvinyl)-1,2,3,6-tetrahydro-1,1'-biphenyl (9b).

According to the general procedure **B**, the crude material was purified by flash chromatography (silica gel, petroleum ether/ ethyl acetate = 5:1) as yellow oil (33.1 mg, 80%) (*Z/E* > 99: 1).

^{1}H NMR (400 MHz, $CDCl_3$) δ 7.36 – 7.26 (m, 6H), 7.23 – 7.15 (m, 4H), 7.11 (d, *J* = 8.2 Hz, 2H), 6.93 (m, 2H), 6.62 (s, 1H), 5.68 (m, 1H), 2.81 – 2.70 (m, 1H), 2.47 – 2.30 (m, 6H), 2.23 – 2.15 (m, 1H), 2.11 – 2.04 (m, 1H), 1.85 – 1.75 (m, 1H).

^{13}C NMR (100 MHz, $CDCl_3$) δ 155.01, 145.83, 143.45, 139.05, 138.12, 136.73, 134.53, 129.55, 129.23, 128.55, 128.00, 127.66, 127.46, 126.76, 126.63, 126.39, 39.27, 34.59, 29.62, 26.22, 21.60.

HRMS (ESI) Calcd for $C_{27}H_{26}NaO_2S^+$ $[M+Na]^+$: 437.1546, found: 437.1549.

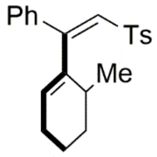

(*E*)-1-methyl-4-((2-(6-methylcyclohex-1-en-1-yl)-2-phenylvinyl)sulfonyl)benzene (10a).

According to the general procedure **A**, the crude material was purified by flash chromatography (silica gel, petroleum ether/ ethyl acetate = 5:1) as yellow oil (24.6 mg, 70%) (*E/Z* > 99: 1).

^{1}H NMR (400 MHz, $CDCl_3$) δ 7.80 (d, *J* = 8.2 Hz, 2H), 7.38 – 7.31 (m, 7H), 6.56 (s, 1H), 5.85 (m, 1H), 2.44 (s, 3H), 2.19 – 2.13 (m, 2H), 1.78 – 1.68 (m, 2H), 1.63 – 1.54 (m, 1H), 1.40 – 1.22 (m, 2H), 0.61 (d, *J* = 7.0 Hz, 3H).

^{13}C NMR (100 MHz, $CDCl_3$) δ 157.89, 143.93, 139.84, 137.92, 137.23, 131.63, 129.89, 129.55, 128.64, 128.47, 128.02, 127.67, 31.68, 31.04, 25.82, 21.64, 20.55, 18.89.

HRMS (ESI) Calcd for $C_{22}H_{24}NaO_2S^+$ $[M+Na]^+$: 375.1389, found: 375.1396.

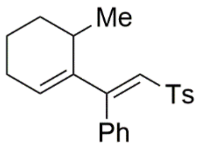

(*Z*)-1-methyl-4-((2-(6-methylcyclohex-1-en-1-yl)-2-phenylvinyl)sulfonyl)benzene (10b).

According to the general procedure **B**, the crude material was purified by flash chromatography (silica gel, petroleum ether/ ethyl acetate = 5:1) as yellow oil (28.1 mg, 80%) (*Z/E* > 99: 1).

^{1}H NMR (400 MHz, $CDCl_3$) δ 7.33 (d, *J* = 8.2 Hz, 2H), 7.27 (m, 1H), 7.21 (m, 2H), 7.09 (d, *J* = 8.2 Hz, 2H), 6.98 – 6.89 (m, 2H), 6.62 (s, 1H), 5.58 (m, 1H), 2.46 (s, 1H), 2.36 (s, 3H), 2.04 (m, 2H), 1.71 – 1.58 (m, 2H), 1.58 – 1.52 (m, 2H), 1.06 (d, *J* = 7.0 Hz, 3H).

^{13}**C NMR** (100 MHz, $CDCl_3$) δ 155.71, 143.31, 142.86, 139.09, 136.44, 135.08, 129.53, 129.16, 128.09, 127.48, 127.40, 126.75, 29.94, 28.37, 26.51, 21.55, 19.77, 17.30.

HRMS (ESI) Calcd for $C_{22}H_{24}NaO_2S^+$ $[M+Na]^+$: 375.1389, found: 375.1394.

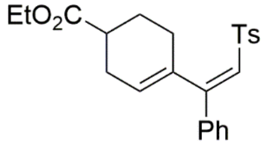

Ethyl (*E*)-4-(1-phenyl-2-tosylvinyl)cyclohex-3-ene-1-carboxylate (11a).

According to the general procedure **A**, the crude material was purified by flash chromatography (silica gel, petroleum ether/ ethyl acetate = 5:1) as yellow oil (24.6 mg, 60%) (*E/Z* > 99: 1).

^{1}**H NMR** (400 MHz, $CDCl_3$) δ 7.79 (d, *J* = 8.2 Hz, 2H), 7.41 – 7.34 (m, 5H), 7.33 (d, *J* = 8.2 Hz, 2H), 6.70 (s, 1H), 5.65 (m, 1H), 4.17 (q, *J* = 7.1 Hz, 2H), 2.60 – 2.51 (m, 1H), 2.44 (s, 3H), 2.40 (m, 2H), 1.95 – 1.83 (m, 3H), 1.66 – 1.58 (m, 1H), 1.28 (t, *J* = 7.1 Hz, 3H).

^{13}**C NMR** (100 MHz, $CDCl_3$) δ 175.42, 157.17, 143.97, 139.74, 136.57, 133.09, 130.29, 129.55, 128.81, 128.38, 127.87, 127.78, 127.43, 60.48, 38.39, 27.54, 27.14, 24.77, 21.61, 14.26.

HRMS (ESI) Calcd for $C_{24}H_{26}NaO_4S^+$ $[M+Na]^+$: 433.1444, found: 433.1445.

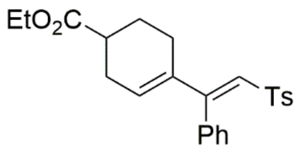

Ethyl (*Z*)-4-(1-phenyl-2-tosylvinyl)cyclohex-3-ene-1-carboxylate (11b).

According to the general procedure **B**, the crude material was purified by flash chromatography (silica gel, petroleum ether/ ethyl acetate = 5:1) as yellow oil (28.7 mg, 70%) (*Z/E* > 99: 1).

^{1}**H NMR** (400 MHz, $CDCl_3$) δ 7.34 – 7.27 (m, 3H), 7.22 (m, 2H), 7.10 (d, *J* = 8.2 Hz, 2H), 6.97 – 6.84 (m, 2H), 6.58 (s, 1H), 5.58 (s, 1H), 4.13 (q, *J* = 7.1 Hz, 2H), 2.50 (m,

1H), 2.37 (s, 3H), 2.35 – 2.28 (m, 3H), 2.29 – 2.20 (m, 1H), 2.16 – 2.09 (m, 1H), 1.81 – 1.68 (m, 1H), 1.24 (t, J = 7.1 Hz, 3H).

^{13}C NMR (100 MHz, $CDCl_3$) δ 174.94, 154.69, 143.46, 138.96, 136.41, 136.21, 134.29, 129.51, 129.20, 128.03, 127.64, 127.44, 126.85, 60.55, 38.44, 28.68, 25.06, 24.86, 21.54, 14.21.

HRMS (ESI) Calcd for $C_{24}H_{26}NaO_4S^+$ [M+Na]$^+$: 433.1444, found: 433.1443.

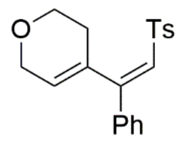

(*E*)-4-(1-phenyl-2-tosylvinyl)-3,6-dihydro-2H-pyran (12a).

According to the general procedure **A**, the crude material was purified by flash chromatography (silica gel, petroleum ether/ ethyl acetate = 5:1) as yellow oil (23.8 mg, 70%) (*E/Z* > 99: 1).

^{1}H NMR (400 MHz, $CDCl_3$) δ 7.82 (d, J = 8.2 Hz, 2H), 7.43 – 7.37 (m, 5H), 7.34 (m, 2H), 6.76 (s, 1H), 5.78 – 5.73 (m, 1H), 4.27 (m, 2H), 3.70 (t, J = 5.5 Hz, 2H), 2.44 (s, 3H), 1.88 – 1.83 (m, 2H).

^{13}C NMR (100 MHz, $CDCl_3$) δ 155.74, 144.14, 139.43, 136.03, 130.83, 130.49, 129.65, 128.91, 128.86, 128.17, 127.83, 127.56, 65.07, 63.74, 27.82, 21.65.

HRMS (ESI) Calcd for $C_{20}H_{20}NaO_3S^+$ [M+Na]$^+$: 363.1025, found: 363.1021.

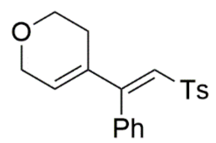

(*Z*)-4-(1-phenyl-2-tosylvinyl)-3,6-dihydro-2H-pyran (12b).

According to the general procedure **B**, the crude material was purified by flash chromatography (silica gel, petroleum ether/ ethyl acetate = 5:1) as yellow oil (24.5 mg, 72%) (*Z/E* > 99: 1).

^{1}H NMR (400 MHz, $CDCl_3$) δ 7.34 – 7.30 (m, 3H), 7.26 – 7.22 (m, 2H), 7.11 (d, *J* = 8.2 Hz, 2H), 6.94 – 6.89 (m, 2H), 6.60 (s, 1H), 5.55 (m, 1H), 4.17 – 4.13 (m, 2H), 3.84 (t, *J* = 5.5 Hz, 2H), 2.38 (s, 3H), 2.33 (m, 2H).

^{13}C NMR (100 MHz, $CDCl_3$) δ 153.44, 143.67, 138.70, 135.59, 134.45, 133.59, 129.49, 129.27, 128.21, 127.68, 127.56, 127.32, 66.02, 63.88, 25.54, 21.58.

HRMS (ESI) Calcd for $C_{20}H_{20}NaO_3S^+$ $[M+Na]^+$: 363.1025, found: 363.1025.

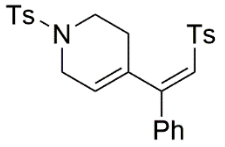

(*E*)-4-(1-phenyl-2-tosylvinyl)-1-tosyl-1,2,3,6-tetrahydropyridine (13a).

According to the general procedure **A**, the crude material was purified by flash chromatography (silica gel, petroleum ether/ ethyl acetate = 2:1) as yellow oil (27.1 mg, 55%) (*E/Z* > 99: 1).

^{1}H NMR (600 MHz, $CDCl_3$) δ 7.72 (dd, *J* = 7.9, 6.7 Hz, 4H), 7.40 (t, *J* = 8.7 Hz, 3H), 7.36 (t, *J* = 7.6 Hz, 2H), 7.32 (d, *J* = 7.5 Hz, 2H), 7.28 (d, *J* = 6.9 Hz, 2H), 6.73 (s, 1H), 5.73 (s, 1H), 3.73 (d, *J* = 2.6 Hz, 2H), 3.07 (t, *J* = 5.5 Hz, 2H), 2.50 (s, 3H), 2.44 (s, 3H), 1.95 (s, 2H).

^{13}C NMR (150 MHz, $CDCl_3$) δ 154.85, 144.31, 143.76, 138.86, 135.63, 133.15, 131.73, 130.60, 129.82, 129.73, 128.95, 128.41, 127.76, 127.68, 127.42, 125.29, 44.69, 42.28, 31.45, 28.33, 21.60.

HRMS (ESI) Calcd for $C_{27}H_{27}NNaO_4S_2^+$ $[M+Na]^+$: 516.1274, found: 516.1278.

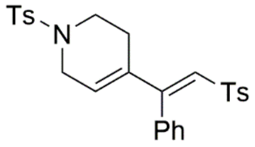

(*Z*)-4-(1-phenyl-2-tosylvinyl)-1-tosyl-1,2,3,6-tetrahydropyridine (13b).

According to the general procedure **B**, the crude material was purified by flash chromatography (silica gel, petroleum ether/ ethyl acetate = 5:1) as yellow oil (30.6 mg, 62%) (*Z/E* > 99: 1).

^{1}H NMR (600 MHz, $CDCl_3$) δ 7.64 (d, *J* = 7.9 Hz, 2H), 7.31 (m, 5H), 7.22 (m, 2H), 7.12 (d, *J* = 7.9 Hz, 2H), 6.85 (m, 2H), 6.58 (s, 1H), 5.45 (m, 1H), 3.60 (s, 2H), 3.24 (t, *J* = 5.6 Hz, 2H), 2.42 (s, 5H), 2.38 (s, 3H).

^{13}C NMR (150 MHz, $CDCl_3$) δ 152.94, 143.95, 143.80, 138.47, 135.32, 133.42, 132.76, 131.53, 129.77, 129.35, 129.30, 128.35, 128.17, 127.70, 127.64, 127.63, 45.60, 42.53, 31.45, 25.75, 21.54.

HRMS (ESI) Calcd for $C_{27}H_{27}NNaO_4S_2^+$ $[M+Na]^+$: 516.1274, found: 516.1277.

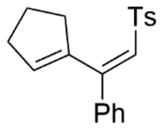

(*E*)-1-((2-(cyclopent-1-en-1-yl)-2-phenylvinyl)sulfonyl)-4-methylbenzene (14a).

According to the general procedure **A**, the crude material was purified by flash chromatography (silica gel, petroleum ether/ ethyl acetate = 5:1) as yellow oil (19.8 mg, 61%) (*E/Z* = 88: 12). The two isomers are separable via silica gel chromatography.

^{1}H NMR (400 MHz, $CDCl_3$) δ 7.76 (d, *J* = 8.2 Hz, 2H), 7.35 (m, 5H), 7.31 (m, 2H), 6.75 (s, 1H), 5.90 (m, 1H), 2.51 – 2.46 (m, 2H), 2.43 (s, 3H), 2.03 – 1.99 (m, 2H), 1.81 – 1.74 (m, 2H).

^{13}C NMR (100 MHz, $CDCl_3$) δ 153.41, 143.83, 142.24, 139.30, 137.72, 136.10, 130.13, 129.38, 128.72, 128.46, 127.93, 127.41, 35.64, 33.51, 23.43, 21.63.

HRMS (ESI) Calcd for $C_{20}H_{20}NaO_2S^+$ $[M+Na]^+$: 347.1076, found: 347.1077.

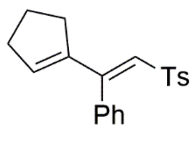

(*Z*)-1-((2-(cyclopent-1-en-1-yl)-2-phenylvinyl)sulfonyl)-4-methylbenzene (14b).

According to the general procedure **B**, the crude material was purified by flash chromatography (silica gel, petroleum ether/ ethyl acetate = 5:1) as yellow oil (25.9 mg, 80%) (*Z/E* = 80: 20). The two isomers are separable via silica gel chromatography.

^{1}H NMR (400 MHz, $CDCl_3$) δ 7.34 (d, *J* = 8.2 Hz, 2H), 7.29 (m, 1H), 7.25 (m, 1H),

7.22 (m, 1H), 7.10 (d, J = 8.2 Hz, 2H), 6.99 – 6.91 (m, 2H), 6.47 (s, 1H), 5.58 (m, 1H), 2.56 – 2.50 (m, 2H), 2.38 (m, 5H), 2.02 – 1.96 (m, 2H).

^{13}C NMR (100 MHz, $CDCl_3$) δ 150.83, 144.21, 143.46, 142.23, 138.98, 135.09, 129.19, 128.95, 127.93, 127.92, 127.69, 127.39, 33.81, 31.96, 23.20, 21.56.

HRMS (ESI) Calcd for $C_{20}H_{20}NaO_2S^+$ $[M+Na]^+$: 347.1076, found: 347.1079.

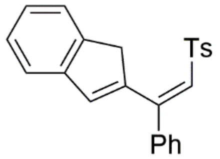

(*E*)-2-(1-phenyl-2-tosylvinyl)-1*H*-indene (15a).

According to the general procedure **A**, the crude material was purified by flash chromatography (silica gel, petroleum ether/ ethyl acetate = 5:1) as yellow oil (33.1 mg, 89%) (*E/Z* > 99: 1).

^{1}H NMR (400 MHz, $CDCl_3$) δ 7.64 (d, J = 8.2 Hz, 2H), 7.47 – 7.44 (m, 1H), 7.38 (m, 1H), 7.35 – 7.30 (m, 6H), 7.22 (m, 1H), 7.10 (d, J = 8.2 Hz, 2H), 7.04 (s, 1H), 6.86 (m, 1H), 3.14 (s, 2H), 2.34 (s, 3H).

^{13}C NMR (100 MHz, $CDCl_3$) δ 151.66, 144.42, 143.88, 143.52, 140.87, 138.73, 138.37, 137.53, 130.17, 129.65, 129.34, 128.72, 128.00, 127.93, 126.69, 125.88, 123.60, 122.07, 41.46, 21.56.

HRMS (ESI) Calcd for $C_{24}H_{20}NaO_2S^+$ $[M+Na]^+$: 395.1076, found: 395.1078.

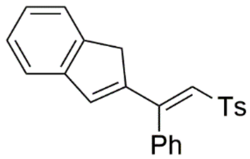

(*Z*)-2-(1-phenyl-2-tosylvinyl)-1*H*-indene (15b).

According to the general procedure **B**, the crude material was purified by flash chromatography (silica gel, petroleum ether/ ethyl acetate = 5:1) as yellow oil (26.1 mg, 70%) (*Z/E* > 99: 1).

^{1}H NMR (400 MHz, $CDCl_3$) δ 8.06 (s, 1H), 7.49 (d, J = 8.3 Hz, 2H), 7.41 – 7.28 (m, 6H), 7.22 – 7.17 (m, 6H), 2.38 (s, 2H), 2.37 (s, 3H).

^{13}C NMR (100 MHz, $CDCl_3$) δ 144.42, 143.95, 138.98, 136.43, 135.72, 131.49, 130.77, 130.32, 130.26, 129.54, 129.46, 129.34, 129.28, 129.15, 128.64, 128.43, 128.15, 128.22, 21.63, 21.20.

HRMS (ESI) Calcd for $C_{24}H_{20}NaO_2S^+$ $[M+Na]^+$: 395.1076, found: 395.1076.

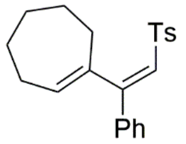

(*E*)-1-(1-phenyl-2-tosylvinyl)cyclohept-1-ene (16a).

According to the general procedure **A**, the crude material was purified by flash chromatography (silica gel, petroleum ether/ ethyl acetate = 5:1) as yellow oil (24.6 mg, 70%) (*E/Z* = 96: 4). The two isomers are separable via silica gel chromatography.

^{1}H NMR (400 MHz, $CDCl_3$) δ 7.79 (d, *J* = 8.2 Hz, 2H), 7.41 – 7.34 (m, 5H), 7.32 (d, *J* = 8.2 Hz, 2H), 6.56 (s, 1H), 5.80 (m, 1H), 2.44 (s, 3H), 2.24 (m, 2H), 1.91 – 1.87 (m, 2H), 1.72 – 1.63 (m, 4H), 1.45 (m, 2H).

^{13}C NMR (100 MHz, $CDCl_3$) δ 159.90, 143.75, 140.08, 138.50, 137.22, 135.58, 130.13, 129.51, 128.65, 127.97, 127.80, 126.43, 32.52, 31.80, 29.19, 26.41, 26.37, 21.63.

HRMS (ESI) Calcd for $C_{22}H_{24}NaO_2S^+$ $[M+Na]^+$: 375.1389, found: 375.1388.

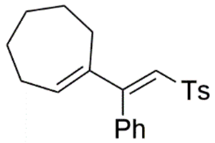

(*Z*)-1-(1-phenyl-2-tosylvinyl)cyclohept-1-ene (16b).

According to the general procedure **B**, the crude material was purified by flash chromatography (silica gel, petroleum ether/ ethyl acetate = 5:1) as yellow oil (28.5 mg, 81%) (*Z/E* = 98: 2). The two isomers are separable via silica gel chromatography.

^{1}H NMR (400 MHz, $CDCl_3$) δ 7.34 (d, *J* = 8.2 Hz, 2H), 7.31 – 7.27 (m, 1H), 7.22 (m, 2H), 7.10 (d, *J* = 8.2 Hz, 2H), 6.96 (m, 2H), 6.63 (s, 1H), 5.80 (m, 1H), 2.36 (s, 3H), 2.33 – 2.26 (m, 2H), 2.15 (m, 2H), 1.78 – 1.69 (m, 2H), 1.46 (m, 4H).

^{13}C NMR (100 MHz, $CDCl_3$) δ 157.31, 144.91, 143.34, 141.14, 139.01, 135.02, 129.63, 129.17, 128.18, 127.58, 127.43, 126.27, 31.90, 29.61, 28.88, 25.99, 25.95, 21.55.

HRMS (ESI) Calcd for $C_{22}H_{24}NaO_2S^+$ $[M+Na]^+$: 375.1389, found: 375.1387.

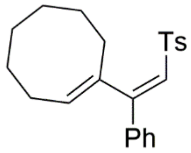

(*E*)-1-((*E*)-1-phenyl-2-tosylvinyl)cyclooct-1-ene (17a).

According to the general procedure **A**, the crude material was purified by flash chromatography (silica gel, petroleum ether/ ethyl acetate = 5:1) as yellow oil (18.3 mg, 50%) (*E/Z* = 97: 3). The two isomers are separable via silica gel chromatography.

^{1}H NMR (400 MHz, $CDCl_3$) δ 7.77 (d, *J* = 8.2 Hz, 2H), 7.35 (m, 5H), 7.31 (d, *J* = 8.2 Hz, 2H), 6.48 (s, 1H), 5.73 (m, 1H), 2.44 (s, 3H), 2.25 – 2.19 (m, 2H), 1.96 – 1.90 (m, 2H), 1.65 – 1.59 (m, 4H), 1.49 (s, 2H), 1.22 (m, 2H).

^{13}C NMR (100 MHz, $CDCl_3$) δ 160.27, 143.75, 139.82, 138.27, 135.94, 133.78, 129.76, 129.46, 129.14, 128.43, 127.98, 127.88, 29.79, 28.81, 28.78, 26.68, 26.08, 25.85, 21.65.

HRMS (ESI) Calcd for $C_{23}H_{26}NaO_2S^+$ $[M+Na]^+$: 389.1546, found: 389.1545.

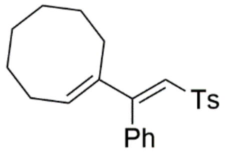

(*E*)-1-((*Z*)-1-phenyl-2-tosylvinyl)cyclooct-1-ene (17b).

According to the general procedure **B**, the crude material was purified by flash chromatography (silica gel, petroleum ether/ ethyl acetate = 5:1) as yellow oil (22.0 mg, 60%) (*Z/E* > 99: 1).

^{1}H NMR (400 MHz, $CDCl_3$) δ 7.21 (m, 3H), 7.13 (m, 2H), 7.01 (d, *J* = 8.2 Hz, 2H), 6.80 (m, 2H), 6.63 (s, 1H), 5.51 (m, 1H), 2.41 – 2.36 (m, 2H), 2.29 (s, 3H), 2.07 (s, 2H), 1.52 (s, 2H), 1.37 (s, 6H).

^{13}C NMR (100 MHz, $CDCl_3$) δ 155.49, 143.28, 141.15, 140.52, 139.09, 134.78, 129.62, 129.14, 127.90, 127.57, 127.39, 126.93, 29.72, 28.56, 28.05, 26.96, 25.93, 25.54, 21.55.

HRMS (ESI) Calcd for $C_{23}H_{26}NaO_2S^+$ $[M+Na]^+$: 389.1546, found: 389.1548.

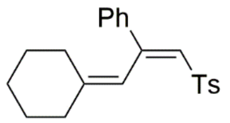

(*E*)-1-((3-cyclohexylidene-2-phenylprop-1-en-1-yl)sulfonyl)-4-methylbenzene (18a).

According to the general procedure **A**, the crude material was purified by flash chromatography (silica gel, petroleum ether/ ethyl acetate = 5:1) as yellow oil (23.6 mg, 67%) (*E/Z* > 99: 1).

^{1}H NMR (400 MHz, $CDCl_3$) δ 7.82 (d, *J* = 8.2 Hz, 2H), 7.40 – 7.34 (m, 3H), 7.34 – 7.28 (m, 4H), 6.57 (m, 1H), 6.36 (s, 1H), 2.43 (s, 3H), 2.24 – 2.20 (m, 2H), 1.58 (m, 2H), 1.49 – 1.45 (m, 2H), 1.44 – 1.39 (m, 2H), 1.12 (m, 2H).

^{13}C NMR (100 MHz, $CDCl_3$) δ 152.68, 151.43, 143.80, 139.59, 139.30, 129.83, 129.44, 128.64, 127.75, 127.59, 127.30, 116.59, 37.33, 31.30, 28.09, 26.86, 26.01, 21.62.

HRMS (ESI) Calcd for $C_{22}H_{24}NaO_2S^+$ $[M+Na]^+$: 375.1389, found: 375.1389.

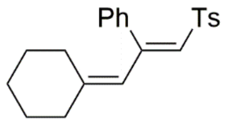

(*Z*)-1-((3-cyclohexylidene-2-phenylprop-1-en-1-yl)sulfonyl)-4-methylbenzene (18b).

According to the general procedure **B**, the crude material was purified by flash chromatography (silica gel, petroleum ether/ ethyl acetate = 5:1) as yellow oil (22.9 mg, 65%) (*Z/E* = 97: 3). The two isomers are separable via silica gel chromatography.

^{1}H NMR (400 MHz, $CDCl_3$) δ 7.40 (d, *J* = 8.2 Hz, 2H), 7.29 (m, 1H), 7.27 – 7.22 (m, 2H), 7.13 – 7.08 (m, 4H), 6.46 (s, 1H), 5.77 (s, 1H), 2.36 (s, 3H), 2.15 – 2.10 (m, 2H), 1.94 – 1.90 (m, 2H), 1.57 (dd, *J* = 8.2, 4.0 Hz, 2H), 1.51 (m, 2H), 1.39 – 1.34 (m, 2H).

^{13}C NMR (100 MHz, $CDCl_3$) δ 152.45, 151.62, 143.46, 139.02, 136.37, 129.67, 129.22, 128.70, 128.54, 127.77, 127.51, 122.80, 38.58, 29.94, 28.70, 27.90, 26.30, 21.56.

HRMS (ESI) Calcd for $C_{22}H_{24}NaO_2S^+$ $[M+Na]^+$: 375.1389, found: 375.1394.

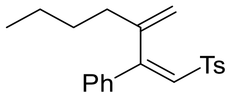

(E)-1-methyl-4-((3-methylene-2-phenylhept-1-en-1-yl)sulfonyl)benzene (19a).

According to the general procedure **A**, the crude material was purified by flash chromatography (silica gel, petroleum ether/ ethyl acetate = 5:1) as yellow oil (6.8 mg, 20%) (*Z/E* > 99: 1).

^{1}H NMR (400 MHz, $CDCl_3$) δ 7.81 (d, *J* = 8.3 Hz, 2H), 7.39 – 7.34 (m, 5H), 7.32 (d, *J* = 8.3 Hz, 2H), 6.62 (s, 1H), 5.33 (d, *J* = 1.5 Hz, 1H), 5.08 (d, *J* = 1.2 Hz, 1H), 2.43 (s, 3H), 1.94 – 1.86 (m, 2H), 1.33 – 1.28 (m, 2H), 1.18 (m, 2H), 0.80 (t, *J* = 7.3 Hz, 3H).

^{13}C NMR (101 MHz, $CDCl_3$) δ 157.49, 144.03, 139.37, 136.81, 131.48, 130.31, 130.16, 129.58, 128.74, 128.08, 127.40, 116.50, 34.71, 29.03, 22.46, 21.61, 13.92.

HRMS (ESI) Calcd for $C_{21}H_{24}NaO_2S^+$ $[M+Na]^+$: 363.1389, found: 363.1390.

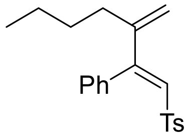

(Z)-1-methyl-4-((3-methylene-2-phenylhept-1-en-1-yl)sulfonyl)benzene (19b).

According to the general procedure **B**, the crude material was purified by flash chromatography (silica gel, petroleum ether/ ethyl acetate = 5:1) as yellow oil (10.1 mg, 31%) (*Z/E* > 99: 1).

^{1}H NMR (400 MHz, $CDCl_3$) δ 7.75 (d, *J* = 8.3 Hz, 2H), 7.29 – 7.23 (m, 5H), 7.12 – 7.10 (m, 2H), 6.27 (s, 1H), 5.38 – 5.23 (m, 2H), 2.64 (dd, *J* = 9.0, 6.0 Hz, 2H), 2.38 (s, 3H), 2.31 (dd, *J* = 7.0, 3.6 Hz, 2H), 1.20 – 1.17 (m, 2H), 0.77 – 0.73 (m, 3H).

^{13}C NMR (101 MHz, $CDCl_3$) δ 158.35, 149.13, 144.14, 139.50, 138.76, 129.85, 129.77, 128.54, 128.25, 127.69, 127.27, 117.29, 30.51, 29.02, 22.69, 21.64, 13.84.

HRMS (ESI) Calcd for $C_{21}H_{24}NaO_2S^+$ $[M+Na]^+$: 363.1389, found: 363.1391.

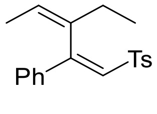

1-(((1E,3Z)-3-ethyl-2-phenylpenta-1,3-dien-1-yl)sulfonyl)-4-methylbenzene (20a).

According to the general procedure **A**, the crude material was purified by flash chromatography (silica gel, petroleum ether/ ethyl acetate = 5:1) as yellow oil (6.8 mg, 21%) (*Z/E* > 99: 1).

^{1}H NMR (400 MHz, $CDCl_3$) δ 7.78 (d, *J* = 8.3 Hz, 2H), 7.37 – 7.30 (m, 7H), 6.57 (s, 1H), 5.58 (q, *J* = 6.8 Hz, 1H), 2.44 (s, 3H), 2.03 (q, *J* = 7.6 Hz, 2H), 1.79 (d, *J* = 7.0 Hz, 3H), 0.69 (t, *J* = 7.6 Hz, 3H).

^{13}C NMR (101 MHz, $CDCl_3$) δ 158.58, 143.87, 139.90, 137.91, 136.38, 129.93, 129.53, 128.59, 128.42, 128.39, 127.89, 127.75, 22.77, 21.61, 13.60, 11.98.

HRMS (ESI) Calcd for $C_{20}H_{22}NaO_2S^+$ $[M+Na]^+$: 349.1233, found: 349.1235.

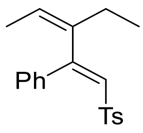

1-(((1Z,3Z)-3-ethyl-2-phenylpenta-1,3-dien-1-yl)sulfonyl)-4-methylbenzene (20b).

According to the general procedure **B**, the crude material was purified by flash chromatography (silica gel, petroleum ether/ ethyl acetate = 5:1) as yellow oil (11.4 mg, 35%) (*Z/E* > 99: 1).

^{1}H NMR (400 MHz, $CDCl_3$) δ 7.26 – 7.19 (m, 3H), 7.16 – 7.10 (m, 2H), 7.02 (d, *J* = 8.0 Hz, 2H), 6.82 (dd, *J* = 8.2, 1.2 Hz, 2H), 6.58 (s, 1H), 5.32 (q, *J* = 7.1 Hz, 1H), 2.29 (s, 3H), 2.20 (q, *J* = 7.5 Hz, 2H), 1.60 (d, *J* = 7.0 Hz, 3H), 0.94 (t, *J* = 7.6 Hz, 3H).

^{13}C NMR (101 MHz, $CDCl_3$) δ 155.82, 143.30, 142.56, 139.09, 134.89, 134.39, 129.58, 129.15, 128.01, 127.53, 127.39, 126.97, 21.54, 20.57, 14.48, 12.93.

HRMS (ESI) Calcd for $C_{20}H_{22}NaO_2S^+$ $[M+Na]^+$: 349.1233, found: 349.1232.

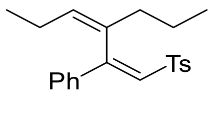

1-methyl-4-(((1E,3Z)-2-phenyl-3-propylhexa-1,3-dien-1-yl)sulfonyl)benzene (21a).

According to the general procedure **A**, the crude material was purified by flash chromatography (silica gel, petroleum ether/ ethyl acetate = 5:1) as yellow oil (8.5 mg,

24%) (*Z/E* > 99: 1).

^{1}H NMR (400 MHz, $CDCl_3$) δ 7.80 (d, *J* = 8.3 Hz, 2H), 7.39 – 7.30 (m, 7H), 6.51 (s, 1H), 5.50 (t, *J* = 7.2 Hz, 1H), 2.44 (s, 3H), 2.23 (p, *J* = 7.5 Hz, 2H), 1.99 (m, 2H), 1.20 – 1.12 (m, 2H), 1.10 (t, *J* = 7.3 Hz, 3H), 0.75 (t, *J* = 7.3 Hz, 3H).

^{13}C NMR (101 MHz, $CDCl_3$) δ 158.68, 143.83, 139.97, 138.10, 135.82, 134.08, 129.88, 129.59, 128.58, 127.87, 127.76, 127.70, 32.53, 21.60, 21.52, 21.32, 14.46, 13.82.

HRMS (ESI) Calcd for $C_{22}H_{26}NaO_2S^+$ $[M+Na]^+$: 377.1546, found: 377.1547.

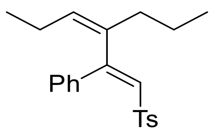

1-methyl-4-(((1Z,3Z)-2-phenyl-3-propylhexa-1,3-dien-1-yl)sulfonyl)benzene (21b).

According to the general procedure **B**, the crude material was purified by flash chromatography (silica gel, petroleum ether/ ethyl acetate = 5:1) as yellow oil (11.3 mg, 32%) (*Z/E* > 99: 1).

^{1}H NMR (400 MHz, $CDCl_3$) δ 7.35 – 7.29 (m, 3H), 7.26 – 7.19 (m, 2H), 7.11 (d, *J* = 8.3 Hz, 2H), 6.93 (m, 2H), 6.68 (s, 1H), 5.38 (t, *J* = 7.2 Hz, 1H), 2.39 (s, 3H), 2.25 – 2.18 (m, 2H), 2.11 (p, *J* = 7.5 Hz, 2H), 1.49 – 1.39 (m, 2H), 0.92 (m, 6H).

^{13}C NMR (101 MHz, $CDCl_3$) δ 156.61, 143.26, 141.71, 139.65, 139.07, 134.95, 129.56, 129.12, 128.07, 127.52, 127.41, 127.36, 29.59, 22.29, 21.88, 21.54, 14.00, 13.60.

HRMS (ESI) Calcd for $C_{22}H_{26}NaO_2S^+$ $[M+Na]^+$: 377.1546, found: 377.1548.

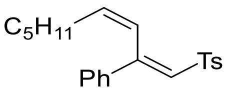

1-methyl-4-(((1E,3Z)-2-phenylnona-1,3-dien-1-yl)sulfonyl)benzene (22a).

According to the general procedure **A**, the crude material was purified by flash chromatography (silica gel, petroleum ether/ ethyl acetate = 4:1) as yellow oil (16.3 mg, 46%) (*E/Z* > 99: 1).

^{1}H NMR (400 MHz, $CDCl_3$) δ 7.89 – 7.80 (d, *J* = 8.2 Hz, 2H), 7.47 (m, 1H), 7.35 – 7.30 (m, 5H), 7.23 – 7.19 (m, 2H), 6.12 (s, 1H), 5.82 (dt, *J* = 15.4, 7.0 Hz, 1H), 2.42 (s, 3H), 2.20 (m, 2H), 1.40 – 1.34 (m, 2H), 1.29 – 1.23 (m, 4H), 0.88 (t, *J* = 7.0 Hz, 3H).
^{13}C NMR (101 MHz, $CDCl_3$) δ 153.12, 146.55, 144.01, 139.51, 138.62, 129.82, 129.03, 128.84, 128.27, 127.26, 125.41, 124.89, 33.48, 31.43, 28.26, 22.46, 21.62, 14.02.
HRMS (ESI) Calcd for $C_{22}H_{26}NaO_2S^+$ $[M+Na]^+$: 377.1546, found: 377.1546.

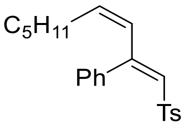

1-methyl-4-(((1Z,3Z)-2-phenylnona-1,3-dien-1-yl)sulfonyl)benzene (22b).

According to the general procedure **B**, the crude material was purified by flash chromatography (silica gel, petroleum ether/ ethyl acetate = 4:1) as yellow oil (24.8 mg, 70%) (*Z*/*E* = 1: 1).

^{1}H NMR (400 MHz, $CDCl_3$) δ 7.29 – 7.23 (m, 3H), 7.21 – 7.16 (m, 2H), 7.03 (d, *J* = 8.2 Hz, 2H), 6.93 – 6.82 (m, 2H), 6.42 (s, 1H), 6.13 (dt, *J* = 15.4, 7.0 Hz, 1H), 5.53 – 5.38 (m, 1H), 2.28 (s, 3H), 1.98 (td, *J* = 8.0, 1.0 Hz, 2H), 1.21 (m, 2H), 1.17 – 1.06 (m, 4H), 0.76 (t, *J* = 7.0 Hz, 3H).
^{13}C NMR (101 MHz, $CDCl_3$) δ 153.28, 144.46, 143.54, 138.96, 133.71, 131.79, 129.24, 129.14, 129.08, 128.17, 127.63, 127.61, 32.97, 31.34, 28.20, 22.38, 21.55, 13.96.
HRMS (ESI) Calcd for $C_{22}H_{26}NaO_2S^+$ $[M+Na]^+$: 377.1546, found: 377.1541.

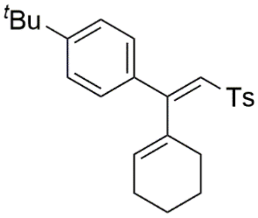

(*E*)-1-(*tert*-butyl)-4-(1-(cyclohex-1-en-1-yl)-2-tosylvinyl)benzene (23a).

According to the general procedure **A**, the crude material was purified by flash chromatography (silica gel, petroleum ether/ ethyl acetate = 5:1) as yellow oil (30.7 mg, 78%) (*E/Z* > 99: 1).

^{1}**H NMR** (400 MHz, $CDCl_3$) δ 7.78 (d, *J* = 8.2 Hz, 2H), 7.35 (m, 4H), 7.30 (d, *J* = 8.2 Hz, 2H), 6.70 (s, 1H), 5.62 (m, 1H), 2.42 (s, 3H), 2.17 – 2.08 (m, 2H), 1.71 (m, 2H), 1.61 – 1.54 (m, 2H), 1.50 (m, 2H), 1.30 (s, 9H).

^{13}**C NMR** (100 MHz, $CDCl_3$) δ 158.05, 153.68, 143.62, 140.00, 133.90, 133.39, 130.42, 129.37, 127.85, 127.27, 126.72, 125.71, 34.78, 31.19, 27.73, 25.24, 22.13, 21.61, 21.48.

HRMS (ESI) Calcd for $C_{25}H_{30}NaO_2S^+$ $[M+Na]^+$: 417.1859, found: 417.1858.

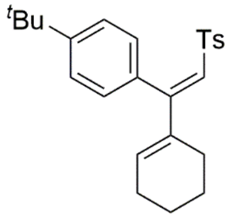

(*Z*)-1-(*tert*-butyl)-4-(1-(cyclohex-1-en-1-yl)-2-tosylvinyl)benzene (23b).

According to the general procedure **B**, the crude material was purified by flash chromatography (silica gel, petroleum ether/ ethyl acetate = 5:1) as yellow oil (31.5 mg, 80%) (*Z/E* > 99: 1).

^{1}**H NMR** (400 MHz, $CDCl_3$) δ 7.22 (d, *J* = 8.2 Hz, 2H), 7.19 – 7.13 (m, 2H), 7.01 (d, *J* = 8.2 Hz, 2H), 6.79 – 6.71 (m, 2H), 6.61 (s, 1H), 5.62 (m, 1H), 2.34 (s, 3H), 2.25 – 2.18 (m, 2H), 2.05 (m, 2H), 1.74 – 1.66 (m, 2H), 1.59 – 1.51 (m, 2H), 1.33 (s, 9H).

^{13}**C NMR** (100 MHz, $CDCl_3$) δ 155.99, 150.78, 142.93, 138.98, 138.48, 136.81, 131.38, 129.36, 128.91, 127.64, 126.51, 124.17, 34.55, 31.41, 26.55, 25.58, 22.47, 21.58, 21.51.

HRMS (ESI) Calcd for $C_{25}H_{30}NaO_2S^+$ $[M+Na]^+$: 417.1859, found: 417.1860.

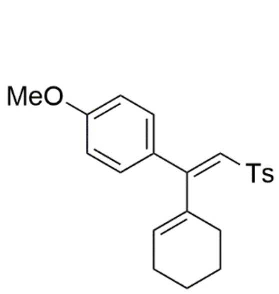

(*E*)-1-((2-(cyclohex-1-en-1-yl)-2-(4-methoxyphenyl)vinyl)sulfonyl)-4-methylbenzene (24a).

According to the general procedure **A**, the crude material was purified by flash

chromatography (silica gel, petroleum ether/ ethyl acetate = 5:1) as yellow oil (26.9 mg, 73%) (*E/Z* > 99: 1).

^{1}H NMR (400 MHz, $CDCl_3$) δ 7.78 (d, *J* = 8.2 Hz, 2H), 7.32 (m, 4H), 6.86 (d, *J* = 8.2 Hz, 2H), 6.63 (s, 1H), 5.61 (m, 1H), 3.81 (s, 3H), 2.42 (s, 3H), 2.13 – 2.10 (m, 2H), 1.73 – 1.68 (m, 2H), 1.62 – 1.55 (m, 2H), 1.53 – 1.44 (m, 2H).

^{13}C NMR (100 MHz, $CDCl_3$) δ 161.36, 157.83, 143.57, 140.10, 133.44, 130.40, 129.37, 129.06, 129.04, 127.82, 125.51, 114.13, 55.40, 27.79, 25.22, 22.13, 21.60, 21.48.

HRMS (ESI) Calcd for $C_{22}H_{24}NaO_3S^+$ $[M+Na]^+$: 391.1338, found: 391.1339.

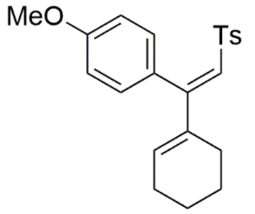

(*Z*)-1-((2-(cyclohex-1-en-1-yl)-2-(4-methoxyphenyl)vinyl)sulfonyl)-4-methylbenzene (24b).

According to the general procedure **B**, the crude material was purified by flash chromatography (silica gel, petroleum ether/ ethyl acetate = 5:1) as yellow oil (33.1 mg, 90%) (*Z/E* > 99: 1).

^{1}H NMR (400 MHz, $CDCl_3$) δ 7.34 (d, *J* = 8.2 Hz, 2H), 7.11 (d, *J* = 8.2 Hz, 2H), 6.82 (m, 2H), 6.74 (m, 2H), 6.56 (s, 1H), 5.65 (m, 1H), 3.82 (s, 3H), 2.37 (s, 3H), 2.21 – 2.17 (m, 2H), 2.06 (m, 2H), 1.72 – 1.66 (m, 2H), 1.57 – 1.53 (m, 2H).

^{13}C NMR (100 MHz, $CDCl_3$) δ 159.40, 155.66, 143.23, 139.28, 138.40, 137.09, 130.91, 129.08, 127.58, 126.75, 126.11, 112.81, 55.24, 26.55, 25.69, 22.45, 21.58, 21.54.

HRMS (ESI) Calcd for $C_{22}H_{24}NaO_3S^+$ $[M+Na]^+$: 391.1338, found: 391.1333.

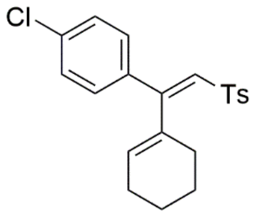

(*E*)-1-chloro-4-(1-(cyclohex-1-en-1-yl)-2-tosylvinyl)benzene (25a).

According to the general procedure **A**, the crude material was purified by flash chromatography (silica gel, petroleum ether/ ethyl acetate = 5:1) as yellow oil (27.5 mg, 74%) (*E/Z* > 99: 1).

^{1}H NMR (600 MHz, $CDCl_3$) δ 7.80 (d, *J* = 8.2 Hz, 2H), 7.35 – 7.31 (m, 6H), 6.68 (s, 1H), 5.73 (m, 1H), 2.45 (s, 3H), 2.17 – 2.13 (m, 2H), 1.67 – 1.64 (m, 2H), 1.59 – 1.57 (m, 2H), 1.51 – 1.48 (m, 2H).

^{13}C NMR (150 MHz, $CDCl_3$) δ 156.74, 143.92, 139.45, 136.24, 135.47, 132.96, 131.31, 129.47, 128.99, 128.81, 128.06, 127.93, 27.50, 25.22, 22.02, 21.63, 21.36.

HRMS (ESI) Calcd for $C_{21}H_{21}ClNaO_2S^+$ $[M+Na]^+$: 395.0843, found: 395.0841.

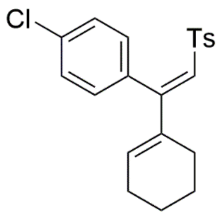

(*Z*)-1-chloro-4-(1-(cyclohex-1-en-1-yl)-2-tosylvinyl)benzene (25b).

According to the general procedure **B**, the crude material was purified by flash chromatography (silica gel, petroleum ether/ ethyl acetate = 5:1) as yellow oil (30.5 mg, 82%) (*Z/E* > 99: 1).

^{1}H NMR (600 MHz, $CDCl_3$) δ 7.37 (d, *J* = 8.2 Hz, 2H), 7.19 (m, 4H), 6.85 (d, *J* = 8.2 Hz, 2H), 6.59 (s, 1H), 5.60 (m, 1H), 2.41 (s, 3H), 2.21 (m, 2H), 2.08 (m, 2H), 1.74 – 1.69 (m, 2H), 1.59 – 1.54 (m, 2H).

^{13}C NMR (150 MHz, $CDCl_3$) δ 154.12, 143.67, 139.11, 138.98, 136.58, 134.04, 133.08, 130.92, 129.25, 127.62, 127.56, 126.47, 26.60, 25.52, 22.36, 21.58, 21.48.

HRMS (ESI) Calcd for $C_{21}H_{21}ClNaO_2S^+$ $[M+Na]^+$: 395.0843, found: 395.0843.

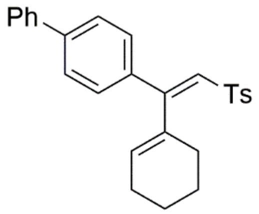

(*E*)-4-(1-(cyclohex-1-en-1-yl)-2-tosylvinyl)-1,1'-biphenyl (26a).

According to the general procedure **A**, the crude material was purified by flash chromatography (silica gel, petroleum ether/ ethyl acetate = 5:1) as yellow oil (31.5 mg, 76%) (*E/Z* > 99: 1).

^{1}H NMR (400 MHz, $CDCl_3$) δ 7.81 (d, *J* = 8.2 Hz, 2H), 7.57 (m, 4H), 7.49 – 7.42 (m, 4H), 7.37 – 7.29 (m, 3H), 6.75 (s, 1H), 5.73 – 5.69 (m, 1H), 2.43 (s, 3H), 2.15 (m, 2H), 1.72 (m, 2H), 1.62 – 1.56 (m, 2H), 1.54 – 1.47 (m, 2H).

^{13}C NMR (100 MHz, $CDCl_3$) δ 157.68, 143.79, 143.00, 140.04, 139.80, 135.80, 133.28, 130.89, 129.45, 128.93, 128.02, 127.93, 127.88, 127.41, 127.40, 127.08, 27.71, 25.28, 22.13, 21.64, 21.47.

HRMS (ESI) Calcd for $C_{27}H_{26}NaO_2S^+$ $[M+Na]^+$: 437.1546, found: 437.1547.

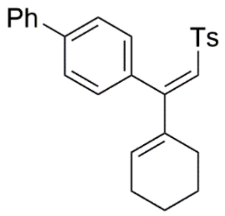

(*Z*)-4-(1-(cyclohex-1-en-1-yl)-2-tosylvinyl)-1,1'-biphenyl (26b).

According to the general procedure **B**, the crude material was purified by flash chromatography (silica gel, petroleum ether/ ethyl acetate = 5:1) as yellow oil (33.1 mg, 80%) (*Z/E* > 99: 1).

^{1}H NMR (400 MHz, $CDCl_3$) δ 7.62 – 7.58 (m, 2H), 7.48 – 7.33 (m, 7H), 7.07 (d, *J* = 8.0 Hz, 2H), 6.99 – 6.92 (m, 2H), 6.62 (s, 1H), 5.68 (s, 1H), 2.35 (s, 3H), 2.27 – 2.20 (m, 2H), 2.11 – 2.04 (m, 2H), 1.75 – 1.68 (m, 2H), 1.56 (m, 2H).

^{13}C NMR (100 MHz, $CDCl_3$) δ 155.32, 143.31, 140.67, 140.64, 139.09, 138.86, 136.76, 133.64, 130.09, 129.09, 128.86, 127.70, 127.52, 127.04, 126.44, 125.97, 26.61, 25.61, 22.46, 21.57, 21.56.

HRMS (ESI) Calcd for $C_{27}H_{26}NaO_2S^+$ $[M+Na]^+$: 437.1546, found: 437.1548.

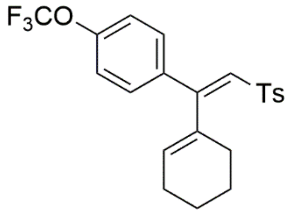

(*E*)-1-((2-(cyclohex-1-en-1-yl)-2-(4-(trifluoromethoxy)phenyl)vinyl)sulfonyl)-4-methylbenzene (27a).

According to the general procedure **A**, the crude material was purified by flash chromatography (silica gel, petroleum ether/ ethyl acetate = 5:1) as yellow oil (30.8 mg, 73%) (*E/Z* > 99: 1).

^{1}H NMR (400 MHz, $CDCl_3$) δ 7.79 (d, *J* = 8.2 Hz, 2H), 7.42 (m, 2H), 7.32 (d, *J* = 8.2 Hz, 2H), 7.19 (m, 2H), 6.68 (s, 1H), 5.72 (s, 1H), 2.44 (s, 3H), 2.16 – 2.12 (m, 2H), 1.66 (m, 2H), 1.60 – 1.55 (m, 2H), 1.52 – 1.46 (m, 2H).

^{13}C NMR (100 MHz, $CDCl_3$) δ 156.44, 150.50 (q, *J* = 1.8 Hz), 143.98, 139.45, 135.56, 132.99, 131.35, 129.50, 129.10, 128.47, 127.93, 120.94, 120.37 (q, *J* = 258.0 Hz), 27.49, 25.22, 22.02, 21.60, 21.35.

^{19}F NMR (377 MHz, $CDCl_3$) δ -57.78 (s, 3F).

HRMS (ESI) Calcd for $C_{22}H_{21}F_3NaO_3S^+$ $[M+Na]^+$: 445.1056, found: 445.1063.

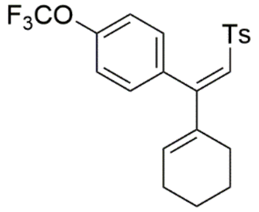

(*Z*)-1-((2-(cyclohex-1-en-1-yl)-2-(4-(trifluoromethoxy)phenyl)vinyl)sulfonyl)-4-methylbenzene (27b).

According to the general procedure **B**, the crude material was purified by flash chromatography (silica gel, petroleum ether/ ethyl acetate = 5:1) as yellow oil (33.8 mg, 80%) (*Z/E* > 99: 1).

^{1}H NMR (400 MHz, $CDCl_3$) δ 7.28 (m, 2H), 7.10 (d, *J* = 8.2 Hz, 2H), 7.03 (d, *J* = 8.2 Hz, 2H), 6.90 (m, 2H), 6.63 (s, 1H), 5.56 (m, 1H), 2.37 (s, 3H), 2.24 – 2.20 (m, 2H), 2.09 – 2.03 (m, 2H), 1.74 – 1.69 (m, 2H), 1.59 – 1.53 (m, 2H).

^{13}C NMR (100 MHz, $CDCl_3$) δ 154.06, 148.87 (q, *J* = 1.8 Hz), 143.64, 139.09, 138.84, 136.61, 133.16, 131.19, 129.18, 127.52, 127.08, 120.45 (q, *J* = 258.0 Hz), 119.84, 26.60, 25.52, 22.35, 21.49, 21.48.

^{19}F NMR (377 MHz, $CDCl_3$) δ -57.71 (s, 3F).

HRMS (ESI) Calcd for $C_{22}H_{21}F_3NaO_3S^+$ $[M+Na]^+$: 445.1056, found: 445.1052.

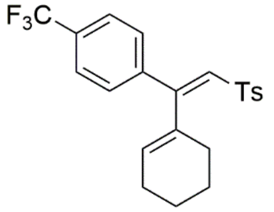

(*E*)-1-((2-(cyclohex-1-en-1-yl)-2-(4-(trifluoromethyl)phenyl)vinyl)sulfonyl)-4-methylbenzene (28a).

According to the general procedure **A**, the crude material was purified by flash chromatography (silica gel, petroleum ether/ ethyl acetate = 5:1) as yellow oil (29.6 mg, 73%) (*E/Z* > 99: 1).

^{1}H NMR (400 MHz, $CDCl_3$) δ 7.80 (d, *J* = 8.2 Hz, 2H), 7.61 (d, *J* = 8.2 Hz, 2H), 7.49 (d, *J* = 8.2 Hz, 2H), 7.33 (d, *J* = 8.2 Hz, 2H), 6.71 (s, 1H), 5.79 – 5.78 (m, 1H), 2.45 (m, 3H), 2.17 – 2.14 (m, 2H), 1.63 – 1.60 (m, 2H), 1.58 – 1.56 (m, 2H), 1.51 – 1.46 (m, 2H).

^{19}F NMR (377 MHz, $CDCl_3$) δ -62.81 (s, 3F).

^{13}C NMR (100 MHz, $CDCl_3$) δ 156.34, 144.11, 140.74, 139.22, 132.81, 131.84 (q, *J* = 32.7 Hz), 131.76, 129.66, 129.54, 128.00, 127.87, 125.71 (q, *J* = 3.6 Hz), 123.72 (q, *J* = 270.3 Hz), 27.40, 25.24, 21.98, 21.63, 21.32.

HRMS (ESI) Calcd for $C_{22}H_{21}F_3NaO_2S^+$ $[M+Na]^+$: 429.1107, found: 429.1108.

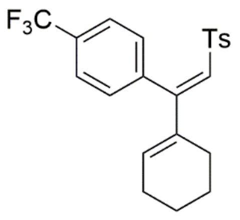

(*Z*)-1-((2-(cyclohex-1-en-1-yl)-2-(4-(trifluoromethyl)phenyl)vinyl)sulfonyl)-4-methylbenzene (28b).

According to the general procedure **B**, the crude material was purified by flash chromatography (silica gel, petroleum ether/ ethyl acetate = 5:1) as yellow oil (39.0 mg, 96%) (*Z/E* > 99: 1).

^{1}H NMR (400 MHz, $CDCl_3$) δ 7.45 (d, *J* = 8.0 Hz, 2H), 7.31 (d, *J* = 8.3 Hz, 2H), 7.10 (d, *J* = 8.0 Hz, 2H), 7.02 (d, *J* = 8.0 Hz, 2H), 6.62 (s, 1H), 5.52 (t, *J* = 4.1 Hz, 1H), 2.38 (s, 3H), 2.26 – 2.20 (m, 2H), 2.09 – 2.04 (m, 2H), 1.75 – 1.69 (m, 2H), 1.59 – 1.52 (m, 2H).

^{19}F NMR (377 MHz, $CDCl_3$) δ -62.66 (s, 3F).

^{13}C NMR (100 MHz, $CDCl_3$) δ 153.58, 143.76, 139.41, 138.73, 138.53, 136.39, 130.01 (q, *J* = 32.5 Hz), 129.95, 129.31, 127.55, 126.85, 124.29 (q, *J* = 3.7 Hz), 124.06 (q, *J* = 270.3 Hz), 26.61, 25.46, 22.32, 21.49, 21.44.

HRMS (ESI) Calcd for $C_{22}H_{21}F_3NaO_2S^+$ $[M+Na]^+$: 429.1107, found: 429.1106.

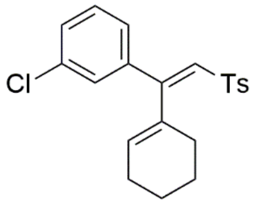

(*E*)-1-chloro-3-(1-(cyclohex-1-en-1-yl)-2-tosylvinyl)benzene (29a).

According to the general procedure **A**, the crude material was purified by flash chromatography (silica gel, petroleum ether/ ethyl acetate = 5:1) as yellow oil (26.0 mg, 70%) (*E/Z* > 99: 1).

^{1}H NMR (400 MHz, $CDCl_3$) δ 7.79 (d, *J* = 8.2 Hz, 2H), 7.36 – 7.25 (m, 6H), 6.66 (s, 1H), 5.80 – 5.70 (m, 1H), 2.44 (s, 3H), 2.15 (m, 2H), 1.64 (m, 2H), 1.59 – 1.54 (m, 2H), 1.52 – 1.46 (m, 2H).

^{13}C NMR (100 MHz, $CDCl_3$) δ 156.50, 144.00, 139.34, 139.05, 134.79, 132.83, 131.59, 130.03, 130.00, 129.51, 128.86, 127.97, 127.51, 125.69, 27.42, 25.23, 22.00, 21.64, 21.34.

HRMS (ESI) Calcd for $C_{21}H_{21}ClNaO_2S^+$ $[M+Na]^+$: 395.0843, found: 395.0839.

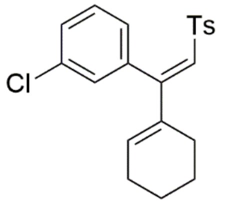

(*Z*)-1-chloro-3-(1-(cyclohex-1-en-1-yl)-2-tosylvinyl)benzene (29b).

According to the general procedure **B**, the crude material was purified by flash chromatography (silica gel, petroleum ether/ ethyl acetate = 5:1) as yellow oil (27.9 mg, 75%) (*Z/E* > 99: 1).

^{1}H NMR (400 MHz, $CDCl_3$) δ 7.32 (d, *J* = 8.2 Hz, 2H), 7.27 – 7.21 (m, 2H), 7.14 (d, *J* = 8.2 Hz, 2H), 6.95 (m, 1H), 6.59 (d, *J* = 8.0 Hz, 2H), 5.58 (m, 1H), 2.40 (s, 3H), 2.21 (m, 2H), 2.07 (m, 2H), 1.74 – 1.68 (m, 2H), 1.58 – 1.52 (m, 2H).

^{13}C NMR (100 MHz, $CDCl_3$) δ 153.78, 143.82, 139.27, 138.76, 136.38, 136.30, 133.40, 129.30, 129.04, 128.79, 128.21, 127.97, 127.56, 126.86, 26.61, 25.43, 22.34, 21.57, 21.47.

HRMS (ESI) Calcd for $C_{21}H_{21}ClNaO_2S^+$ $[M+Na]^+$: 395.0843, found: 395.0845.

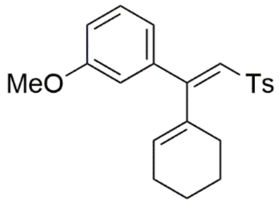

(*E*)-1-(1-(cyclohex-1-en-1-yl)-2-tosylvinyl)-3-methoxybenzene (30a).

According to the general procedure **A**, the crude material was purified by flash chromatography (silica gel, petroleum ether/ ethyl acetate = 5:1) as yellow oil (27.6 mg, 75%) (*E/Z* > 99: 1).

^{1}H NMR (400 MHz, $CDCl_3$) δ 7.79 (d, *J* = 8.2 Hz, 2H), 7.31 (d, *J* = 8.2 Hz, 2H), 7.26 (m, 1H), 6.97 (m, 1H), 6.94 – 6.88 (m, 2H), 6.70 (s, 1H), 5.69 (m, 1H), 3.80 (s, 3H), 2.43 (s, 3H), 2.15 – 2.09 (m, 2H), 1.69 – 1.63 (m, 2H), 1.59 – 1.52 (m, 2H), 1.50 – 1.43 (m, 2H).

^{13}C NMR (100 MHz, $CDCl_3$) δ 159.79, 157.99, 143.78, 139.69, 138.49, 133.30, 130.91, 129.75, 129.42, 127.92, 127.85, 119.93, 115.44, 113.14, 55.37, 27.49, 25.23, 22.08, 21.62, 21.42.

HRMS (ESI) Calcd for $C_{22}H_{24}NaO_3S^+$ $[M+Na]^+$: 391.1338, found: 391.1339.

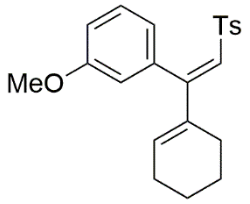

(*Z*)-1-(1-(cyclohex-1-en-1-yl)-2-tosylvinyl)-3-methoxybenzene (30b).

According to the general procedure **B**, the crude material was purified by flash chromatography (silica gel, petroleum ether/ ethyl acetate = 5:1) as yellow oil (33.5 mg, 91%) (*Z/E* > 99: 1).

^{1}H NMR (400 MHz, $CDCl_3$) δ 7.32 (d, *J* = 8.2 Hz, 2H), 7.14 (m, 1H), 7.09 (d, *J* = 8.2 Hz, 2H), 6.84 – 6.79 (m, 1H), 6.57 (s, 1H), 6.54 (m, 1H), 6.27 (s, 1H), 5.65 (m, 1H), 3.68 (s, 3H), 2.36 (s, 3H), 2.24 – 2.18 (m, 2H), 2.08 – 2.02 (m, 2H), 1.74 – 1.66 (m, 2H), 1.58 – 1.51 (m, 2H).

^{13}C NMR (100 MHz, $CDCl_3$) δ 158.60, 155.27, 143.26, 139.02, 138.79, 136.46, 135.73, 129.06, 128.39, 127.70, 126.19, 122.28, 114.56, 113.89, 54.97, 26.57, 25.47, 22.42, 21.54, 21.49.

HRMS (ESI) Calcd for $C_{22}H_{24}NaO_3S^+$ $[M+Na]^+$: 391.1338, found: 391.1338.

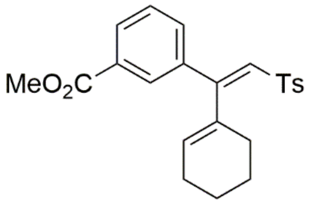

Methyl (*E*)-3-(1-(cyclohex-1-en-1-yl)-2-tosylvinyl)benzoate (31a).

According to the general procedure **A**, the crude material was purified by flash chromatography (silica gel, petroleum ether/ ethyl acetate = 5:1) as yellow oil (24.2 mg, 61%) (*E/Z* > 99: 1).

^{1}H NMR (400 MHz, $CDCl_3$) δ 8.07 – 8.03 (m, 2H), 7.80 (d, *J* = 8.2 Hz, 2H), 7.57 – 7.54 (m, 1H), 7.44 (m, 1H), 7.33 (d, *J* = 8.2 Hz, 2H), 6.74 (s, 1H), 5.82 – 5.76 (m, 1H), 3.93 (s, 3H), 2.44 (s, 3H), 2.16 (m, 2H), 1.62 (m, 2H), 1.59 – 1.53 (m, 2H), 1.50 – 1.44 (m, 2H).

^{13}C NMR (100 MHz, $CDCl_3$) δ 166.47, 156.88, 143.96, 139.38, 137.53, 132.89, 131.84, 131.67, 130.99, 130.85, 129.48, 128.89, 128.81, 128.55, 128.00, 52.36, 27.44, 25.24, 22.00, 21.62, 21.35.

HRMS (ESI) Calcd for $C_{23}H_{24}NaO_4S^+$ $[M+Na]^+$: 419.1288, found: 419.1291.

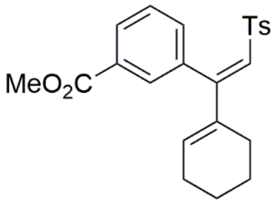

Methyl (*Z*)-3-(1-(cyclohex-1-en-1-yl)-2-tosylvinyl)benzoate (31b).

According to the general procedure **B**, the crude material was purified by flash chromatography (silica gel, petroleum ether/ ethyl acetate = 5:1) as yellow oil (27.7 mg, 70%) (*Z/E* > 99: 1).

^{1}H NMR (400 MHz, $CDCl_3$) δ 7.96 (m, 1H), 7.37 (m, 1H), 7.33 (s, 1H), 7.29 (d, *J* = 8.2 Hz, 2H), 7.27 – 7.23 (m, 1H), 7.09 (d, *J* = 8.2 Hz, 2H), 6.62 (s, 1H), 5.53 (m, 1H), 3.89 (s, 3H), 2.34 (s, 3H), 2.26 – 2.20 (m, 2H), 2.08 – 2.02 (m, 2H), 1.75 – 1.68 (m, 2H), 1.59 – 1.51 (m, 2H).

^{13}C NMR (100 MHz, $CDCl_3$) δ 166.52, 154.27, 143.55, 139.36, 138.96, 136.57, 134.93, 134.36, 130.26, 129.35, 129.26, 129.02, 127.62, 127.49, 126.70, 52.12, 26.61, 25.47, 22.35, 21.48, 21.46.

HRMS (ESI) Calcd for $C_{23}H_{24}NaO_4S^+$ $[M+Na]^+$: 419.1288, found: 419.1290.

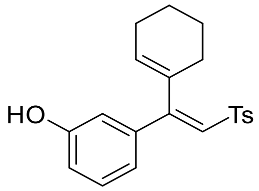

(E)-3-(1-(cyclohex-1-en-1-yl)-2-tosylvinyl)phenol (32a).

According to the general procedure **A**, the crude material was purified by flash chromatography (silica gel, petroleum ether/ ethyl acetate = 2:1) as yellow oil (23.0 mg, 65%) (*E/Z* > 99: 1).

^{1}H NMR (400 MHz, $CDCl_3$) δ 7.77 (d, *J* = 8.3 Hz, 2H), 7.30 (d, *J* = 8.2 Hz, 2H), 7.18 (m, 1H), 6.96 – 6.82 (m, 3H), 6.68 (s, 1H), 5.69 – 5.63 (m, 1H), 2.42 (s, 3H), 2.09 (m, 2H), 1.63 (m, 2H), 1.57 – 1.49 (m, 2H), 1.48 – 1.38 (m, 2H).

^{13}C NMR (101 MHz, $CDCl_3$) δ 158.37, 156.31, 143.94, 139.45, 138.48, 133.24, 131.00, 129.88, 129.48, 127.88, 127.41, 119.70, 117.36, 114.41, 27.47, 25.20, 22.01, 21.63, 21.37.

HRMS (ESI) Calcd for $C_{21}H_{23}O_3S^+$ $[M+H]^+$: 355.1362, found: 355.1362.

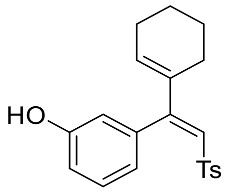

(Z)-3-(1-(cyclohex-1-en-1-yl)-2-tosylvinyl)phenol (32b).

According to the general procedure **B**, the crude material was purified by flash chromatography (silica gel, petroleum ether/ ethyl acetate = 2:1) as yellow oil (23.6 mg, 67%) (*E/Z* > 99: 1).

^{1}H NMR (400 MHz, $CDCl_3$) δ 7.38 – 7.32 (m, 2H), 7.11 (d, J = 8.2 Hz, 2H), 7.03 (m, 1H), 6.77 (m, 1H), 6.53 (s, 1H), 6.41 – 6.33 (m, 2H), 6.02 (br, 1H), 5.68 (m, 1H), 2.36 (s, 3H), 2.18 (m, 2H), 2.05 (s, 2H), 1.72 – 1.64 (m, 2H), 1.57 – 1.50 (m, 2H).
^{13}C NMR (101 MHz, $CDCl_3$) δ 155.51, 155.18, 143.59, 139.14, 138.73, 136.35, 135.76, 129.23, 128.59, 127.64, 125.60, 121.68, 116.88, 115.17, 26.58, 25.49, 22.41, 21.55, 21.52.
HRMS (ESI) Calcd for $C_{21}H_{23}O_3S^+$ $[M+H]^+$: 355.1362, found: 355.1365.

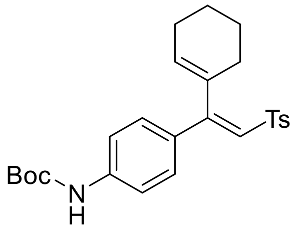

tert-butyl (E)-(4-(1-(cyclohex-1-en-1-yl)-2-tosylvinyl)phenyl)carbamate (33a).

According to the general procedure **A**, the crude material was purified by flash chromatography (silica gel, petroleum ether/ ethyl acetate = 5:1) as yellow oil (27.2 mg, 60%) (E/Z > 99: 1).
^{1}H NMR (400 MHz, $CDCl_3$) δ 7.70 (d, J = 8.3 Hz, 2H), 7.29 (d, J = 8.3 Hz, 2H), 7.25 – 7.20 (m, 4H), 6.71 (m, 1H), 6.56 (s, 1H), 5.61 – 5.49 (m, 1H), 2.35 (s, 3H), 2.04 (m, 2H), 1.58 (s, 2H), 1.51 – 1.46 (m, 2H), 1.44 – 1.37 (m, 11H).
^{13}C NMR (101 MHz, $CDCl_3$) δ 157.69, 152.46, 143.66, 140.44, 139.91, 133.31, 131.06, 130.66, 129.39, 128.43, 127.86, 126.01, 118.23, 80.98, 28.29, 27.69, 25.21, 22.08, 21.61, 21.44.
HRMS (ESI) Calcd for $C_{26}H_{32}NO_4S^+$ $[M+H]^+$: 454.2047, found: 454.2048.

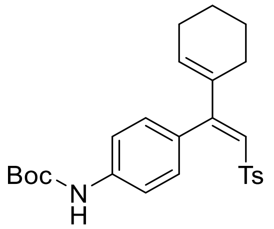

tert-butyl (Z)-(4-(1-(cyclohex-1-en-1-yl)-2-tosylvinyl)phenyl)carbamate (33b).

According to the general procedure **B**, the crude material was purified by flash chromatography (silica gel, petroleum ether/ ethyl acetate = 2:1) as yellow oil (28.6 mg, 63%) (*E/Z* > 99: 1).

^{1}H NMR (400 MHz, $CDCl_3$) δ 7.35 (d, *J* = 8.3 Hz, 2H), 7.23 (d, *J* = 8.3 Hz, 2H), 7.11 (d, *J* = 8.2 Hz, 2H), 6.86 – 6.78 (m, 2H), 6.65 (s, 1H), 6.53 (s, 1H), 5.64 (t, *J* = 4.0 Hz, 1H), 2.36 (s, 3H), 2.17 (m, 2H), 2.03 m, 2H), 1.70 – 1.65 (m, 2H), 1.55 – 1.49 (m, 11H).

^{13}C NMR (101 MHz, $CDCl_3$) δ 155.42, 152.67, 143.45, 139.21, 138.79, 138.28, 136.88, 130.30, 129.19, 129.07, 127.58, 125.94, 117.19, 80.63, 28.35, 26.54, 25.62, 22.43, 21.55.

HRMS (ESI) Calcd for $C_{26}H_{32}NO_4S^+$ $[M+H]^+$: 454.2047, found: 454.2043.

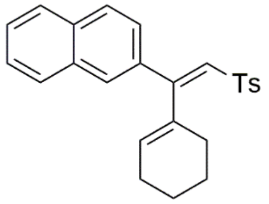

(*E*)-2-(1-(cyclohex-1-en-1-yl)-2-tosylvinyl)naphthalene (34a).

According to the general procedure **A**, the crude material was purified by flash chromatography (silica gel, petroleum ether/ ethyl acetate = 5:1) as yellow oil (29.5 mg, 76%) (*E/Z* = 96: 4). The two isomers are separable via silica gel chromatography.

^{1}H NMR (400 MHz, $CDCl_3$) δ 7.87 – 7.78 (m, 6H), 7.52 – 7.44 (m, 3H), 7.32 (d, *J* = 8.2 Hz, 2H), 6.82 (s, 1H), 5.79 (s, 1H), 2.43 (s, 3H), 2.19 (m, 2H), 1.70 (s, 2H), 1.60 (m, 2H), 1.54 – 1.46 (m, 2H).

^{13}C NMR (100 MHz, $CDCl_3$) δ 158.12, 143.80, 139.75, 134.39, 134.02, 133.35, 133.07, 131.15, 129.46, 128.64, 128.52, 127.97, 127.74, 127.67, 127.62, 127.23, 126.69, 124.44, 27.72, 25.31, 22.12, 21.64, 21.47.

HRMS (ESI) Calcd for $C_{25}H_{24}NaO_2S^+$ $[M+Na]^+$: 411.1389, found: 411.1387.

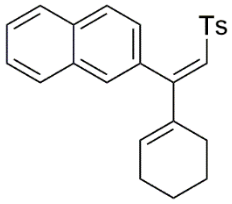

(*Z*)-2-(1-(cyclohex-1-en-1-yl)-2-tosylvinyl)naphthalene (34b).

According to the general procedure **B**, the crude material was purified by flash chromatography (silica gel, petroleum ether/ ethyl acetate = 5:1) as yellow oil (30.7 mg, 79%) (*Z/E* > 99: 1).

^{1}H NMR (400 MHz, $CDCl_3$) δ 7.84 – 7.80 (m, 1H), 7.67 (d, *J* = 8.4 Hz, 1H), 7.64 – 7.61 (m, 1H), 7.52 – 7.44 (m, 2H), 7.23 (s, 1H), 7.18 (d, *J* = 8.2 Hz, 2H), 7.02 (m, 1H), 6.87 (d, *J* = 8.1 Hz, 2H), 6.69 (s, 1H), 5.61 (m, 1H), 2.29 – 2.23 (m, 5H), 2.05 – 1.99 (m, 2H), 1.76 – 1.68 (m, 2H), 1.59 – 1.51 (m, 2H).

^{13}C NMR (100 MHz, $CDCl_3$) δ 155.50, 143.33, 139.14, 139.01, 136.78, 132.70, 132.37, 132.13, 128.94, 128.70, 128.06, 127.68, 127.62, 127.43, 126.86, 126.80, 126.36, 126.09, 26.59, 25.58, 22.45, 21.56, 21.45.

HRMS (ESI) Calcd for $C_{25}H_{24}NaO_2S^+$ $[M+Na]^+$: 411.1389, found: 411.1388.

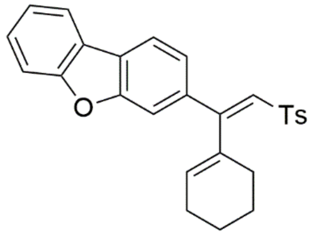

(*E*)-3-(1-(cyclohex-1-en-1-yl)-2-tosylvinyl)dibenzo[*b,d*]furan (35a).

According to the general procedure **A**, the crude material was purified by flash chromatography (silica gel, petroleum ether/ ethyl acetate = 2:1) as yellow oil (30.8 mg, 72%) (*E/Z* = 90:10). The two isomers are separable via silica gel chromatography.

^{1}H NMR (400 MHz, $CDCl_3$) δ 7.84 (m, 2H), 7.74 (d, *J* = 8.2 Hz, 2H), 7.51 – 7.46 (m, 2H), 7.40 (m, 1H), 7.32 (m, 1H), 7.28 – 7.22 (m, 3H), 6.72 (s, 1H), 5.69 (m, 1H), 2.35 (s, 3H), 2.10 (m, 2H), 1.63 (m, 2H), 1.52 (m, 2H), 1.43 (m, 2H).

^{13}**C NMR** (100 MHz, $CDCl_3$) δ 157.91, 156.94, 156.26, 143.86, 139.69, 136.20, 133.37, 131.24, 129.49, 128.00, 127.96, 126.03, 123.52, 123.11, 122.26, 121.03, 120.76, 111.84, 110.87, 27.68, 25.30, 22.11, 21.64, 21.44.

HRMS (ESI) Calcd for $C_{27}H_{24}NaO_3S^+$ $[M+Na]^+$: 451.1338, found: 451.1334.

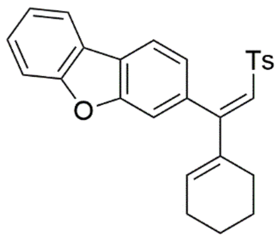

(*Z*)-3-(1-(cyclohex-1-en-1-yl)-2-tosylvinyl)dibenzo[*b*,*d*]furan (35b).

According to the general procedure **B**, the crude material was purified by flash chromatography (silica gel, petroleum ether/ ethyl acetate = 2:1) as yellow oil (34.2 mg, 80%) (*Z/E* > 99: 1).

^{1}**H NMR** (400 MHz, $CDCl_3$) δ 7.88 (m, 1H), 7.73 (m, 1H), 7.47 (d, *J* = 8.2 Hz, 1H), 7.40 (m, 1H), 7.28 (m, 1H), 7.19 (m, 2H), 6.90 (m, 1H), 6.84 (m, 3H), 6.57 (s, 1H), 5.55 (m, 1H), 2.17 (s, 5H), 1.96 (s, 2H), 1.67 – 1.59 (m, 2H), 1.47 (m, 2H).

^{13}**C NMR** (100 MHz, $CDCl_3$) δ 156.50, 155.29, 154.74, 143.56, 139.19, 138.78, 136.82, 133.72, 129.01, 127.61, 127.50, 126.86, 124.62, 123.92, 123.90, 122.92, 120.80, 119.50, 112.77, 111.65, 26.62, 25.59, 22.44, 21.55, 21.44.

HRMS (ESI) Calcd for $C_{27}H_{24}NaO_3S^+$ $[M+Na]^+$: 451.1338, found: 451.1340.

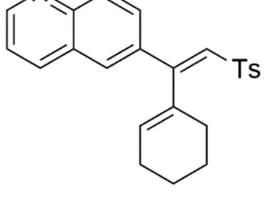

(*E*)-6-(1-(cyclohex-1-en-1-yl)-2-tosylvinyl)quinoline (36a).

According to the general procedure **A**, the crude material was purified by flash chromatography (silica gel, petroleum ether/ ethyl acetate = 1:1) as yellow oil (27.2 mg, 70%) (*E/Z* = 95: 5). The two isomers are separable via silica gel chromatography.

^{1}H NMR (400 MHz, $CDCl_3$) δ 8.94 (m, 1H), 8.18 (m, 1H), 8.09 (m, 1H), 7.85 (m, 3H), 7.70 (m, 1H), 7.45 (m, 1H), 7.34 (d, *J* = 8.2 Hz, 2H), 6.83 (s, 1H), 5.83 (m, 1H), 2.45 (s, 3H), 2.23 – 2.17 (m, 2H), 1.70 (m, 2H), 1.61 (m, 2H), 1.55 – 1.47 (m, 2H).
^{13}C NMR (100 MHz, $CDCl_3$) δ 157.12, 151.39, 148.68, 143.99, 139.45, 136.75, 135.32, 133.09, 131.55, 129.93, 129.52, 128.91, 128.22, 127.99, 127.55, 127.43, 121.84, 27.64, 25.30, 22.06, 21.64, 21.40.
HRMS (ESI) Calcd for $C_{24}H_{23}NNaO_2S^+$ $[M+Na]^+$: 412.1342, found: 412.1343.

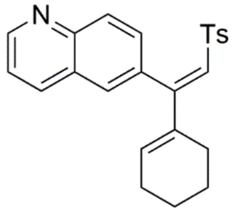

(*Z*)-6-(1-(cyclohex-1-en-1-yl)-2-tosylvinyl)quinoline (36b).

According to the general procedure **B**, the crude material was purified by flash chromatography (silica gel, petroleum ether/ ethyl acetate = 1:1) as yellow oil (29.2 mg, 75%) (*Z/E* > 99: 1).
^{1}H NMR (400 MHz, $CDCl_3$) δ 8.95 (m, 1H), 8.05 (d, *J* = 8.2 Hz, 1H), 7.91 (d, *J* = 8.6 Hz, 1H), 7.43 (m, 1H), 7.41 (s, 1H), 7.21 (d, *J* = 8.2 Hz, 2H), 7.17 (m, 1H), 6.94 (d, *J* = 8.2 Hz, 2H), 6.71 (s, 1H), 5.58 (m, 1H), 2.31 – 2.25 (m, 5H), 2.08 – 2.01 (m, 2H), 1.77 – 1.69 (m, 2H), 1.61 – 1.53 (m, 2H).
^{13}C NMR (100 MHz, $CDCl_3$) δ 154.50, 150.79, 147.43, 143.60, 139.45, 138.88, 136.65, 136.37, 133.04, 130.84, 129.08, 128.94, 128.25, 127.55, 127.28, 127.12, 121.39, 26.61, 25.56, 22.38, 21.49, 21.48.
HRMS (ESI) Calcd for $C_{24}H_{23}NNaO_2S^+$ $[M+Na]^+$: 412.1342, found: 412.1342.

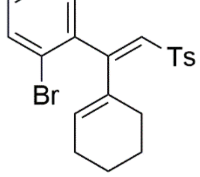

(*E*)-1-bromo-2-(1-(cyclohex-1-en-1-yl)-2-tosylvinyl)benzene (37a).

According to the general procedure **A**, the crude material was purified by flash chromatography (silica gel, petroleum ether/ ethyl acetate = 5:1) as yellow oil (29.5 mg, 71%) (*E/Z* > 99: 1).

^{1}H NMR (400 MHz, $CDCl_3$) δ 7.77 (d, *J* = 8.2 Hz, 2H), 7.49 (m, 1H), 7.26 (d, *J* = 8.2 Hz, 2H), 7.20 – 7.18 (m, 1H), 7.10 – 7.06 (m, 2H), 6.35 (s, 1H), 6.07 (m, 1H), 2.37 (s, 3H), 2.03 (m, 2H), 1.44 – 1.40 (m, 2H), 1.35 – 1.30 (m, 2H), 1.22 (m, 2H).

^{13}C NMR (100 MHz, $CDCl_3$) δ 155.75, 143.91, 139.02, 138.75, 133.53, 133.45, 133.35, 132.25, 129.96, 129.80, 129.34, 128.12, 127.24, 122.23, 26.22, 25.39, 21.80, 21.63, 21.19.

HRMS (ESI) Calcd for $C_{21}H_{21}BrNaO_2S^+$ $[M+Na]^+$: 439.0338, found: 439.0345.

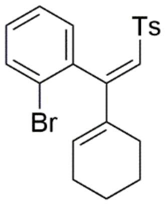

(*Z*)-1-bromo-2-(1-(cyclohex-1-en-1-yl)-2-tosylvinyl)benzene (37b).

According to the general procedure **B**, the crude material was purified by flash chromatography (silica gel, petroleum ether/ ethyl acetate = 5:1) as yellow oil (33.3 mg, 80%) (*Z/E* > 99: 1).

^{1}H NMR (400 MHz, $CDCl_3$) δ 7.37 – 7.32 (m, 3H), 7.24 (m, 1H), 7.14 – 7.12 (m, 1H), 7.10 – 7.07 (m, 3H), 6.46 (s, 1H), 5.46 (m, 1H), 2.32 (s, 3H), 2.15 (m, 2H), 1.98 (s, 2H), 1.68 – 1.60 (m, 2H), 1.53 – 1.44 (m, 2H).

^{13}C NMR (100 MHz, $CDCl_3$) δ 152.56, 142.75, 137.24, 136.75, 134.80, 133.84, 131.19, 130.80, 128.51, 128.30, 126.82, 125.40, 124.63, 122.28, 25.56, 24.40, 21.34, 20.56, 20.50.

HRMS (ESI) Calcd for $C_{21}H_{21}BrNaO_2S^+$ $[M+Na]^+$: 439.0338, found: 439.0340.

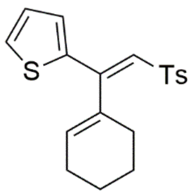

(*E*)-2-(1-(cyclohex-1-en-1-yl)-2-tosylvinyl)thiophene (38a).

According to the general procedure **A**, the crude material was purified by flash chromatography (silica gel, petroleum ether/ ethyl acetate = 5:1) as yellow oil (21.7 mg, 63%) (*E/Z* = 94:6). The two isomers are separable via silica gel chromatography.

^{1}H NMR (400 MHz, $CDCl_3$) δ 7.78 (d, *J* = 8.2 Hz, 2H), 7.37 (m, 1H), 7.30 (d, *J* = 8.2 Hz, 2H), 7.18 (m, 1H), 7.01 (m, 1H), 6.70 (s, 1H), 5.51 – 5.40 (m, 1H), 2.43 (s, 3H), 2.12 – 2.08 (m, 2H), 2.04 (m, 2H), 1.64 (m, 4H).

^{13}C NMR (100 MHz, $CDCl_3$) δ 151.19, 143.83, 141.75, 139.95, 133.57, 129.47, 129.16, 129.12, 128.80, 128.20, 127.79, 124.22, 28.55, 25.11, 22.30, 21.62, 21.48.

HRMS (ESI) Calcd for $C_{19}H_{20}NaO_2S_2^+$ $[M+Na]^+$: 367.0797, found: 367.0797.

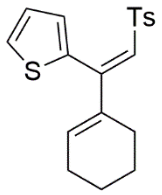

(*Z*)-2-(1-(cyclohex-1-en-1-yl)-2-tosylvinyl)thiophene (38b).

According to the general procedure **B**, the crude material was purified by flash chromatography (silica gel, petroleum ether/ ethyl acetate = 5:1) as yellow oil (24.1 mg, 70%) (*Z/E* > 99: 1).

^{1}H NMR (400 MHz, $CDCl_3$) δ 7.43 (d, *J* = 8.2 Hz, 2H), 7.33 (m, 1H), 7.14 (d, *J* = 8.2 Hz, 2H), 6.99 – 6.95 (m, 2H), 6.62 (s, 1H), 5.87 (m, 1H), 2.37 (s, 3H), 2.20 – 2.15 (m, 2H), 2.12 – 2.08 (m, 2H), 1.72 – 1.65 (m, 2H), 1.59 – 1.52 (m, 2H).

^{13}C NMR (100 MHz, $CDCl_3$) δ 148.81, 143.53, 138.58, 137.78, 137.28, 134.09, 130.81, 129.23, 128.29, 127.72, 127.59, 126.29, 26.41, 26.07, 22.39, 21.58, 21.51.

HRMS (ESI) Calcd for $C_{19}H_{20}NaO_2S_2^+$ $[M+Na]^+$: 367.0797, found: 367.0799.

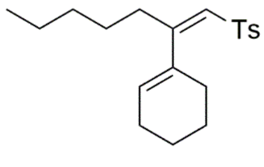

(*Z*)-1-((2-(cyclohex-1-en-1-yl)hept-1-en-1-yl)sulfonyl)-4-methylbenzene (39a).

According to the general procedure **A**, the crude material was purified by flash chromatography (silica gel, petroleum ether/ ethyl acetate = 5:1) as yellow oil (20.6 mg, 62%) (*Z/E* > 99: 1).

^{1}H NMR (400 MHz, $CDCl_3$) δ 7.65 (d, *J* = 8.2 Hz, 2H), 7.21 (d, *J* = 8.2 Hz, 2H), 6.15 (s, 1H), 5.41 – 5.33 (m, 1H), 2.35 (s, 3H), 2.05 (m, 2H), 1.94 – 1.89 (m, 2H), 1.69 (m, 2H), 1.40 – 1.37 (m, 4H), 1.28 (m, 2H), 1.18 (m, 4H), 0.79 (s, 3H).

^{13}C NMR (100 MHz, $CDCl_3$) δ 161.70, 143.49, 139.77, 134.15, 129.27, 127.90, 127.79, 126.96, 36.89, 31.15, 26.93, 26.79, 24.92, 22.37, 22.04, 21.59, 21.45, 13.95.

HRMS (ESI) Calcd for $C_{20}H_{28}NaO_2S^+$ $[M+Na]^+$: 355.1702, found: 355.1703.

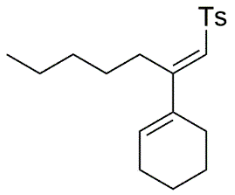

(*E*)-1-((2-(cyclohex-1-en-1-yl)hept-1-en-1-yl)sulfonyl)-4-methylbenzene (39b).

According to the general procedure **B**, the crude material was purified by flash chromatography (silica gel, petroleum ether/ ethyl acetate = 5:1) as yellow oil (16.6 mg, 50%) (*E/Z* > 99: 1).

^{1}H NMR (600 MHz, $CDCl_3$) δ 7.81 (d, *J* = 8.2 Hz, 2H), 7.32 (d, *J* = 8.2 Hz, 2H), 6.24 (s, 1H), 6.17 (m, 1H), 2.73 – 2.66 (m, 2H), 2.44 – 2.42 (m, 3H), 2.21 – 2.14 (m, 2H), 2.07 – 2.04 (m, 2H), 1.68 – 1.63 (m, 2H), 1.58 – 1.54 (m, 2H), 1.34 – 1.20 (m, 9H).

^{13}C NMR (150 MHz, $CDCl_3$) δ 158.19, 143.72, 140.12, 135.12, 131.61, 129.68, 127.16, 124.07, 32.11, 29.42, 27.81, 26.20, 26.08, 22.50, 22.44, 21.62, 21.56, 14.00.

HRMS (ESI) Calcd for $C_{20}H_{28}NaO_2S^+$ $[M+Na]^+$: 355.1702, found: 355.1702.

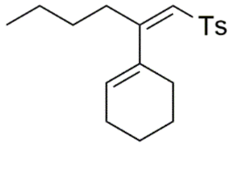

(*Z*)-1-((2-(cyclohex-1-en-1-yl)hex-1-en-1-yl)sulfonyl)-4-methylbenzene (40a).

According to the general procedure **A**, the crude material was purified by flash chromatography (silica gel, petroleum ether/ ethyl acetate = 5:1) as yellow oil (21.0 mg, 66%) (*Z/E* > 99: 1).

^{1}H NMR (400 MHz, $CDCl_3$) δ 7.71 (d, *J* = 8.2 Hz, 2H), 7.28 (m, 2H), 6.22 (s, 1H), 5.43 (m, 1H), 2.42 (s, 3H), 2.13 (m, 2H), 2.02 – 1.95 (m, 2H), 1.81 – 1.75 (m, 2H), 1.51 – 1.43 (m, 4H), 1.38 – 1.21 (m, 4H), 0.87 (m, 3H).

^{13}C NMR (100 MHz, $CDCl_3$) δ 161.66, 143.49, 139.77, 134.16, 129.26, 127.84, 127.76, 126.96, 36.65, 29.27, 26.93, 24.91, 22.11, 22.03, 21.56, 21.44, 13.81.

HRMS (ESI) Calcd for $C_{19}H_{26}NaO_2S^+$ $[M+Na]^+$: 341.1546, found: 341.1547.

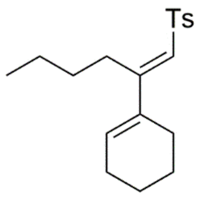

(*E*)-1-((2-(cyclohex-1-en-1-yl)hex-1-en-1-yl)sulfonyl)-4-methylbenzene (40b).

According to the general procedure **B**, the crude material was purified by flash chromatography (silica gel, petroleum ether/ ethyl acetate = 5:1) as yellow oil (17.5 mg, 55%) (*E/Z* > 99: 1).

^{1}H NMR (400 MHz, $CDCl_3$) δ 7.82 – 7.78 (m, 2H), 7.32 (d, *J* = 8.2 Hz, 2H), 6.23 (s, 1H), 6.17 (m, 1H), 2.71 – 2.67 (m, 2H), 2.43 (s, 3H), 2.19 – 2.14 (m, 2H), 2.07 – 2.03 (m, 2H), 1.67 – 1.62 (m, 2H), 1.58 – 1.53 (m, 2H), 1.33 – 1.25 (m, 4H), 0.86 (m, 3H).

^{13}C NMR (100 MHz, $CDCl_3$) δ 158.17, 143.72, 140.13, 135.16, 131.60, 129.69, 127.20, 124.08, 31.84, 27.64, 26.21, 26.08, 23.07, 22.50, 21.62, 21.57, 13.87.

HRMS (ESI) Calcd for $C_{19}H_{26}NaO_2S^+$ $[M+Na]^+$: 341.1546, found: 341.1547.

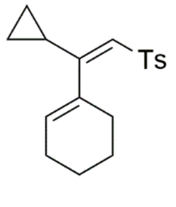

(*Z*)-1-((2-(cyclohex-1-en-1-yl)-2-cyclopropylvinyl)sulfonyl)-4-methylbenzene(41a).

According to the general procedure **A**, the crude material was purified by flash chromatography (silica gel, petroleum ether/ ethyl acetate = 5:1) as yellow oil (18.4 mg, 61%) (*Z/E* > 99: 1).

^{1}H NMR (400 MHz, $CDCl_3$) δ 7.71 (d, *J* = 8.2 Hz, 2H), 7.28 (d, *J* = 8.2 Hz, 2H), 6.14 (s, 1H), 5.16 (m, 1H), 2.42 (s, 3H), 1.97 (s, 4H), 1.57 – 1.52 (m, 4H), 1.43 (m, 1H), 0.82 – 0.76 (m, 2H), 0.59 – 0.55 (m, 2H).

^{13}C NMR (100 MHz, $CDCl_3$) δ 163.64, 143.46, 140.08, 133.19, 129.30, 127.62, 126.83, 124.23, 28.43, 24.85, 22.11, 21.57, 21.49, 17.53, 7.67.

HRMS (ESI) Calcd for $C_{18}H_{22}NaO_2S^+$ $[M+Na]^+$: 325.1233, found: 325.1229.

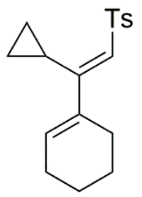

(*E*)-1-((2-(cyclohex-1-en-1-yl)-2-cyclopropylvinyl)sulfonyl)-4-methylbenzene(41b).

According to the general procedure **B**, the crude material was purified by flash chromatography (silica gel, petroleum ether/ ethyl acetate = 5:1) as yellow oil (18.1 mg, 60%) (*E/Z* > 99: 1).

^{1}H NMR (400 MHz, $CDCl_3$) δ 7.75 (d, *J* = 8.2 Hz, 2H), 7.25 (d, *J* = 8.2 Hz, 2H), 6.08 (s, 1H), 5.39 (m, 1H), 2.37 (s, 3H), 1.93 – 1.89 (m, 4H), 1.52 – 1.43 (m, 5H), 0.78 – 0.74 (m, 2H), 0.43 (m, 2H).

^{13}C NMR (100 MHz, $CDCl_3$) δ 161.22, 142.64, 138.84, 134.12, 128.58, 126.44, 126.11, 125.46, 27.70, 23.91, 21.26, 20.68, 20.58, 10.00, 6.82.

HRMS (ESI) Calcd for $C_{18}H_{22}NaO_2S^+$ $[M+Na]^+$: 325.1233, found: 325.1227.

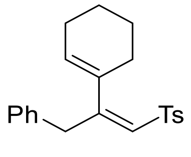

(Z)-1-((2-(cyclohex-1-en-1-yl)-3-phenylprop-1-en-1-yl)sulfonyl)-4-methylbenzene (42a).

According to the general procedure **A**, the crude material was purified by flash chromatography (silica gel, petroleum ether/ ethyl acetate = 4:1) as yellow oil (10.6 mg, 30%) (*E/Z* > 99: 1).

^{1}H NMR (400 MHz, $CDCl_3$) δ 7.62 (d, *J* = 8.3 Hz, 2H), 7.22 – 7.15 (m, 5H), 7.03 – 6.99 (m, 2H), 6.03 (m, 1H), 5.29 – 5.21 (m, 1H), 3.36 (s, 2H), 2.34 (s, 3H), 1.87 – 1.80 (m, 2H), 1.72 (m, 2H), 1.36 (m, 4H).

^{13}C NMR (101 MHz, $CDCl_3$) δ 160.48, 143.67, 139.55, 136.30, 134.21, 129.39, 129.33, 128.61, 128.53, 128.18, 127.77, 126.98, 43.77, 27.29, 24.90, 22.02, 21.60, 21.37.

HRMS (ESI) Calcd for $C_{22}H_{24}NaO_2S^+$ $[M+Na]^+$: 375.1389, found: 375.1391.

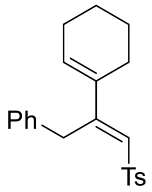

(E)-1-((2-(cyclohex-1-en-1-yl)-3-phenylprop-1-en-1-yl)sulfonyl)-4-methylbenzene (42b).

According to the general procedure **B**, the crude material was purified by flash chromatography (silica gel, petroleum ether/ ethyl acetate = 2:1) as yellow oil (10.9 mg, 31%) (*E/Z* > 99: 1).

^{1}H NMR (400 MHz, $CDCl_3$) δ 7.72 (d, *J* = 8.3 Hz, 2H), 7.26 – 7.09 (m, 7H), 6.20 (m, 2H), 3.67 (m, 2H), 2.34 (s, 3H), 2.12 – 2.10 (m, 2H), 2.01 – 1.99 (m, 2H), 1.60 – 1.57 (m, 2H), 1.50 – 1.49 (m, 2H).

^{13}C NMR (101 MHz, $CDCl_3$) δ 156.03, 143.90, 139.91, 134.94, 132.22, 130.02, 129.83, 128.46, 127.18, 126.14, 124.43, 41.45, 26.36, 26.16, 22.52, 21.63.

HRMS (ESI) Calcd for $C_{22}H_{24}NaO_2S^+$ $[M+Na]^+$: 375.1389, found: 375.1390.

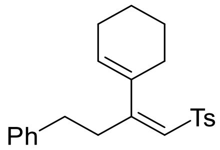

(Z)-1-((2-(cyclohex-1-en-1-yl)-4-phenylbut-1-en-1-yl)sulfonyl)-4-methylbenzene

(43a).

According to the general procedure **A**, the crude material was purified by flash chromatography (silica gel, petroleum ether/ ethyl acetate = 2:1) as yellow oil (7.3 mg, 20%) (*E/Z* > 99: 1).

^{1}H NMR (400 MHz, $CDCl_3$) δ 7.60 (d, *J* = 8.3 Hz, 2H), 7.20 (m, 3H), 7.14 – 7.09 (m, 2H), 7.04 – 7.00 (m, 2H), 6.13 (s, 1H), 5.42 – 5.34 (m, 1H), 2.60 (m, 2H), 2.38 – 2.34 (m, 5H), 1.95 – 1.90 (m, 2H), 1.74 (m, 2H), 1.40 (m, 4H).

^{13}C NMR (101 MHz, $CDCl_3$) δ 160.25, 143.59, 140.43, 139.68, 133.96, 129.30, 128.51, 128.41, 128.33, 127.76, 127.66, 126.26, 38.43, 33.51, 27.02, 24.96, 22.04, 21.61, 21.45.

HRMS (ESI) Calcd for $C_{23}H_{26}NaO_2S^+$ $[M+Na]^+$: 389.1546, found: 389.1547.

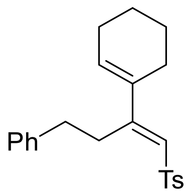

(E)-1-((2-(cyclohex-1-en-1-yl)-4-phenylbut-1-en-1-yl)sulfonyl)-4-methylbenzene (43b).

According to the general procedure **B**, the crude material was purified by flash chromatography (silica gel, petroleum ether/ ethyl acetate = 2:1) as yellow oil (8.4 mg, 23%) (*E/Z* > 99: 1).

^{1}H NMR (400 MHz, $CDCl_3$) δ 7.74 – 7.68 (m, 2H), 7.24 – 7.13 (m, 7H), 6.19 (m, 2H), 3.02 – 2.85 (m, 2H), 2.66 – 2.60 (m, 2H), 2.34 (d, *J* = 6.4 Hz, 3H), 2.16 – 2.06 (m, 2H), 2.02 – 1.96 (m, 2H), 1.63 – 1.54 (m, 2H), 1.53 – 1.45 (m, 2H).

^{13}C NMR (101 MHz, $CDCl_3$) δ 156.61, 143.88, 141.50, 139.89, 134.92, 132.20, 129.81, 128.44, 127.16, 126.12, 124.41, 36.11, 29.90, 26.34, 26.14, 22.50, 21.61.

HRMS (ESI) Calcd for $C_{23}H_{26}NaO_2S^+$ $[M+Na]^+$: 389.1546, found: 389.1549.

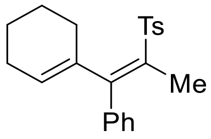

(E)-1-((1-(cyclohex-1-en-1-yl)-1-phenylprop-1-en-2-yl)sulfonyl)-4-methylbenzene (44a).

According to the general procedure **A**, the crude material was purified by flash chromatography (silica gel, petroleum ether/ ethyl acetate = 5:1) as yellow oil (17.6 mg, 50%) (*E/Z* = 1: 1).

^{1}H NMR (400 MHz, $CDCl_3$) δ 7.77 (d, J = 8.3 Hz, 1H), 7.38 (d, J = 8.3 Hz, 1.5H), 7.35 – 7.28 (m, 1.5H), 7.20 (m, 1H), 7.17 – 7.14 (m, 1H), 7.10 (d, J = 8.2 Hz, 1.5H), 7.04 – 6.95 (m, 1.5H), 5.74 – 5.66 (m, 0.5H), 5.62 (m, 0.5H), 2.43 (s, 1.5H), 2.36 (s, 1.5H), 2.20 (s, 1.5H), 2.09 – 2.06 (m, 1.5H), 2.01 (s, 1.5H), 1.77 (m, 1.5H), 1.54 – 1.50 (m, 3H), 1.47 – 1.39 (m, 1H), 1.35 – 1.28 (m, 1H).

^{13}C NMR (101 MHz, $CDCl_3$) δ (154.19, 153.68), (143.47, 143.16), 139.42, (138.79, 138.74), (138.44, 137.95), 137.17, (135.25, 134.95), (129.31, 129.10), (128.65, 128.50), (128.33, 128.26), (128.22, 127.98), (127.62, 127.55), (127.38, 126.76), (27.02, 26.94), (25.31, 25.12), (22.44, 22.05), (21.71, 21.59), (21.51, 21.35), (17.53, 17.43).

HRMS (ESI) Calcd for $C_{22}H_{24}NaO_2S^+$ $[M+Na]^+$: 375.1389, found: 375.1391.

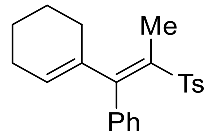

(Z)-1-((1-(cyclohex-1-en-1-yl)-1-phenylprop-1-en-2-yl)sulfonyl)-4-methylbenzene (44b).

According to the general procedure **B**, the crude material was purified by flash chromatography (silica gel, petroleum ether/ ethyl acetate = 5:1) as yellow oil (19.7 mg, 56%) (*Z/E* = 1.5: 1).

^{1}H NMR (400 MHz, $CDCl_3$) δ 7.77 (d, J = 8.3 Hz, 1.2H), 7.40 – 7.28 (m, 4.8H), 7.23 – 7.18 (m, 0.6H), 7.16 – 7.12 (m, 1.6H), 7.10 (d, J = 8.1 Hz, 0.4H), 7.01 (dd, J = 8.0, 1.5 Hz, 0.4H), 5.71 – 5.69 (m, 0.6H), 5.67 – 5.58 (m, 0.4H), 2.44 (s, 1.8H), 2.36 (s, 1.2H), 2.20 (s, 1.2H), 2.07 (dt, J = 6.1, 3.6 Hz, 2H), 2.01 (s, 1.8H), 1.60 – 1.51 (m, 3H), 1.44 – 1.31 (m, 3H).

^{13}C NMR (101 MHz, $CDCl_3$) δ (154.19, 153.68), (143.47, 143.16), 139.42, (138.79, 138.74), (138.44, 137.95), 137.17, (135.25, 134.95), (129.31, 129.10), (128.65, 128.50), (128.33, 128.26), (128.22, 127.98), (127.62, 127.55), (127.38, 126.76), (27.02, 26.94), (25.31, 25.12), (22.44, 22.05), (21.71, 21.59), (21.51, 21.35), (17.53, 17.43).

HRMS (ESI) Calcd for $C_{22}H_{24}NaO_2S^+$ $[M+Na]^+$: 375.1389, found: 375.1391.

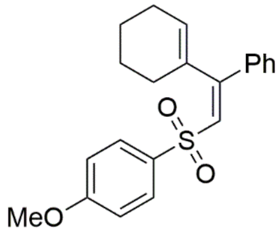

(*E*)-1-((2-(cyclohex-1-en-1-yl)-2-phenylvinyl)sulfonyl)-4-methoxybenzene (45a).

According to the general procedure **A**, the crude material was purified by flash chromatography (silica gel, petroleum ether/ ethyl acetate = 5:1) as yellow oil (29.4 mg, 83%) (*E/Z* > 99: 1).

^{1}H NMR (400 MHz, $CDCl_3$) δ 7.88 – 7.80 (m, 2H), 7.36 (m, 5H), 7.02 – 6.95 (m, 2H), 6.69 (s, 1H), 5.74 – 5.70 (m, 1H), 3.86 (s, 3H), 2.20 – 2.13 (m, 2H), 1.67 (m, 2H), 1.61 – 1.55 (m, 2H), 1.50 (m, 2H).

^{13}C NMR (100 MHz, $CDCl_3$) δ 163.21, 157.75, 137.03, 134.28, 133.31, 130.71, 130.08, 130.05, 128.73, 127.96, 127.47, 114.04, 55.71, 27.57, 25.27, 22.13, 21.46.

HRMS (ESI) Calcd for $C_{21}H_{22}NaO_3S^+$ $[M+Na]^+$: 377.1182, found: 377.1185.

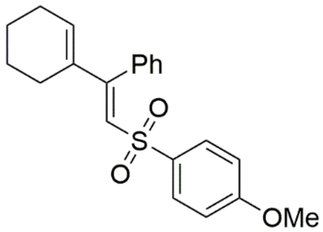

(*Z*)-1-((2-(cyclohex-1-en-1-yl)-2-phenylvinyl)sulfonyl)-4-methoxybenzene (45b).

According to the general procedure **B**, the crude material was purified by flash chromatography (silica gel, petroleum ether/ ethyl acetate = 5:1) as yellow oil (31.2 mg, 88%) (*Z/E* > 99: 1).

^{1}H NMR (400 MHz, $CDCl_3$) δ 7.36 – 7.26 (m, 3H), 7.25 – 7.19 (m, 2H), 6.89 (m, 2H), 6.81 – 6.71 (m, 2H), 6.58 (s, 1H), 5.59 (m, 1H), 3.82 (s, 3H), 2.24 – 2.18 (m, 2H), 2.05 (m, 2H), 1.73 – 1.66 (m, 2H), 1.58 – 1.51 (m, 2H).

^{13}C NMR (100 MHz, $CDCl_3$) δ 162.87, 155.25, 138.64, 136.81, 134.66, 133.80, 129.72, 129.59, 127.83, 127.36, 126.39, 113.75, 55.60, 26.57, 25.55, 22.44, 21.55.

HRMS (ESI) Calcd for $C_{21}H_{22}NaO_3S^+$ $[M+Na]^+$: 377.1182, found: 377.1183.

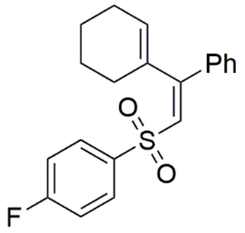

(*E*)-1-((2-(cyclohex-1-en-1-yl)-2-phenylvinyl)sulfonyl)-4-fluorobenzene (46a).

According to the general procedure **A**, the crude material was purified by flash chromatography (silica gel, petroleum ether/ ethyl acetate = 5:1) as yellow oil (27.4 mg, 80%) (*E/Z* > 99: 1).

^{1}H NMR (400 MHz, $CDCl_3$) δ 7.97 – 7.88 (m, 2H), 7.41 – 7.34 (m, 5H), 7.22 – 7.17 (m, 2H), 6.69 (s, 1H), 5.73 – 5.67 (m, 1H), 2.14 (m, 2H), 1.70 – 1.65 (m, 2H), 1.61 – 1.55 (m, 2H), 1.54 – 1.47 (m, 2H).

^{19}F NMR (377 MHz, $CDCl_3$) δ-104.73 (s, 1F).

^{13}C NMR (100 MHz, $CDCl_3$) δ 165.31 (d, J = 255.2 Hz), 158.88, 138.79 (d, J = 3.2 Hz), 136.74, 133.39, 131.00, 130.64 (d, J = 9.4 Hz), 130.33, 128.81, 127.51, 127.17, 116.06 (d, J = 22.6 Hz), 27.60, 25.25, 22.08, 21.42.

HRMS (ESI) Calcd for $C_{20}H_{19}FNaO_2S^+$ $[M+Na]^+$: 365.0982, found: 365.0981.

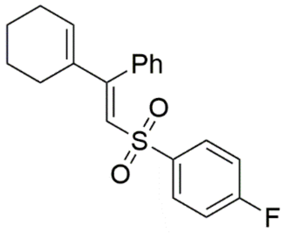

(*Z*)-1-((2-(cyclohex-1-en-1-yl)-2-phenylvinyl)sulfonyl)-4-fluorobenzene (46b).

According to the general procedure **B**, the crude material was purified by flash chromatography (silica gel, petroleum ether/ ethyl acetate = 5:1) as yellow oil (27.4 mg, 80%) (*Z/E* = 95: 5). The two isomers are separable via silica gel chromatography.

^{1}H NMR (400 MHz, $CDCl_3$) δ 7.30 (m, 2H), 7.23 – 7.18 (m, 1H), 7.12 (m, 2H), 6.86 (m, 2H), 6.80 – 6.74 (m, 2H), 6.53 (s, 1H), 5.53 (m, 1H), 2.17 – 2.10 (m, 2H), 1.96 (m, 2H), 1.67 – 1.58 (m, 2H), 1.51 – 1.42 (m, 2H).

^{13}C NMR (100 MHz, $CDCl_3$) δ 165.01 (d, *J* = 254.6 Hz), 156.27, 139.44, 138.01 (d, *J* = 3.0 Hz), 136.66, 134.39, 130.36 (d, *J* = 9.5 Hz), 129.59, 128.02, 127.45, 125.89, 115.66 (d, *J* = 22.6 Hz), 26.61, 25.50, 22.38, 21.49.

^{19}F NMR (377 MHz, $CDCl_3$) δ -105.38 (s, 1F).

HRMS (ESI) Calcd for $C_{20}H_{19}FNaO_2S^+$ $[M+Na]^+$: 365.0982, found: 365.0981.

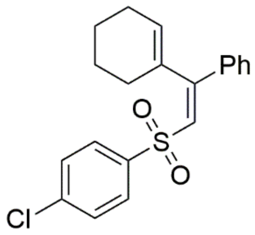

(*E*)-1-chloro-4-((2-(cyclohex-1-en-1-yl)-2-phenylvinyl)sulfonyl)benzene (47a).

According to the general procedure **A**, the crude material was purified by flash chromatography (silica gel, petroleum ether/ ethyl acetate = 5:1) as yellow oil (26.9 mg, 75%) (*E/Z* > 99: 1).

^{1}H NMR (400 MHz, $CDCl_3$) δ 7.85 (m, 2H), 7.54 – 7.47 (m, 2H), 7.41 – 7.34 (m, 5H), 6.67 (s, 1H), 5.71 – 5.66 (m, 1H), 2.17 – 2.11 (m, 2H), 1.70 (m, 2H), 1.62 – 1.55 (m, 2H), 1.54 – 1.48 (m, 2H).

^{13}C NMR (100 MHz, $CDCl_3$) δ 159.19, 141.26, 139.50, 136.68, 133.43, 131.04, 130.38, 129.33, 129.11, 128.82, 127.53, 126.89, 27.65, 25.26, 22.05, 21.41.

HRMS (ESI) Calcd for $C_{20}H_{19}ClNaO_2S^+$ $[M+Na]^+$: 381.0686, found: 381.0693.

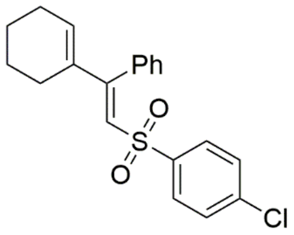

(*Z*)-1-chloro-4-((2-(cyclohex-1-en-1-yl)-2-phenylvinyl)sulfonyl)benzene (47b).

According to the general procedure **B**, the crude material was purified by flash chromatography (silica gel, petroleum ether/ ethyl acetate = 5:1) as yellow oil (28.6 mg, 80%) (*Z/E* > 99: 1).

^{1}H NMR (400 MHz, $CDCl_3$) δ 7.31 (m, 3H), 7.22 (m, 4H), 6.91 – 6.81 (m, 2H), 6.59 (s, 1H), 5.62 (m, 1H), 2.24 – 2.19 (m, 2H), 2.06 (m, 2H), 1.74 – 1.68 (m, 2H), 1.59 – 1.52 (m, 2H).

^{13}C NMR (100 MHz, $CDCl_3$) δ 156.51, 140.47, 139.61, 139.05, 136.66, 134.36, 129.59, 129.06, 128.71, 128.05, 127.45, 125.66, 26.63, 25.51, 22.38, 21.48.

HRMS (ESI) Calcd for $C_{20}H_{19}ClNaO_2S^+$ $[M+Na]^+$: 381.0686, found: 381.0687.

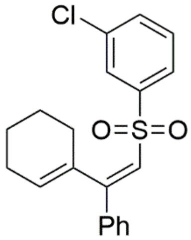

(*E*)-1-chloro-3-((2-(cyclohex-1-en-1-yl)-2-phenylvinyl)sulfonyl)benzene (48a).

According to the general procedure **A**, the crude material was purified by flash chromatography (silica gel, petroleum ether/ ethyl acetate = 5:1) as yellow oil (28.6 mg, 80%) (*E/Z* = 96: 4). The two isomers are separable via silica gel chromatography.

^{1}H NMR (400 MHz, $CDCl_3$) δ 7.91 (m, 1H), 7.83 – 7.76 (m, 1H), 7.56 (m, 1H), 7.47 (m, 1H), 7.41 – 7.35 (m, 5H), 6.69 (s, 1H), 5.72 – 5.66 (m, 1H), 2.15 (m, 2H), 1.66 (m, 2H), 1.61 – 1.56 (m, 2H), 1.52 – 1.47 (m, 2H).

^{13}C NMR (100 MHz, $CDCl_3$) δ 159.53, 144.41, 136.61, 134.95, 133.31, 133.03, 131.40, 130.44, 130.17, 128.83, 128.21, 127.56, 126.78, 125.94, 27.58, 25.19, 22.04, 21.37.

HRMS (ESI) Calcd for $C_{20}H_{19}ClNaO_2S^+$ $[M+Na]^+$: 381.0686, found: 381.0681.

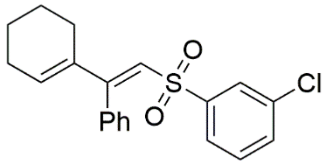

(*Z*)-1-chloro-3-((2-(cyclohex-1-en-1-yl)-2-phenylvinyl)sulfonyl)benzene (48b).

According to the general procedure **B**, the crude material was purified by flash chromatography (silica gel, petroleum ether/ ethyl acetate = 5:1) as yellow oil (27.9 mg, 78%) (*Z/E* > 99: 1).

^{1}H NMR (400 MHz, $CDCl_3$) δ 7.40 (m, 1H), 7.35 – 7.29 (m, 2H), 7.27 – 7.19 (m, 4H), 6.84 (m, 2H), 6.60 (s, 1H), 5.65 (m, 1H), 2.23 (m, 2H), 2.09 – 2.04 (m, 2H), 1.72 (m, 2H), 1.56 (m, 2H).

^{13}C NMR (100 MHz, $CDCl_3$) δ 156.93, 143.59, 139.84, 136.64, 134.57, 134.05, 132.62, 129.77, 129.56, 128.23, 127.92, 127.43, 125.68, 125.56, 26.65, 25.47, 22.37, 21.47.

HRMS (ESI) Calcd for $C_{20}H_{19}ClNaO_2S^+$ $[M+Na]^+$: 381.0686, found: 381.0684.

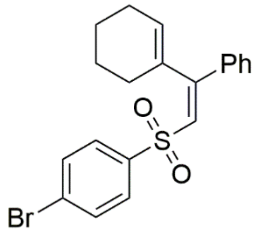

(*E*)-1-bromo-4-((2-(cyclohex-1-en-1-yl)-2-phenylvinyl)sulfonyl)benzene (49a).

According to the general procedure **A**, the crude material was purified by flash chromatography (silica gel, petroleum ether/ ethyl acetate = 5:1) as yellow oil (34.2 mg, 85%) (*E/Z* > 99: 1).

^{1}H NMR (400 MHz, $CDCl_3$) δ 7.80 – 7.75 (m, 2H), 7.68 – 7.64 (m, 2H), 7.41 – 7.35 (m, 5H), 6.67 (s, 1H), 5.70 – 5.66 (m, 1H), 2.16 – 2.11 (m, 2H), 1.70 (m, 2H), 1.61 – 1.56 (m, 2H), 1.54 – 1.48 (m, 2H).

^{13}C NMR (100 MHz, $CDCl_3$) δ 159.23, 141.80, 136.68, 133.45, 132.09, 131.04, 130.39, 129.41, 128.82, 128.01, 127.53, 126.83, 27.67, 25.26, 22.05, 21.42.

HRMS (ESI) Calcd for $C_{20}H_{19}BrNaO_2S^+$ $[M+Na]^+$: 425.0181, found: 425.0183.

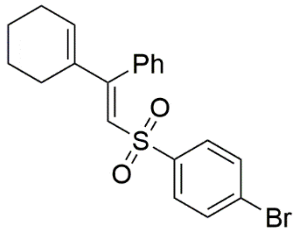

(*Z*)-1-bromo-4-((2-(cyclohex-1-en-1-yl)-2-phenylvinyl)sulfonyl)benzene (49b).

According to the general procedure **B**, the crude material was purified by flash chromatography (silica gel, petroleum ether/ ethyl acetate = 5:1) as yellow oil (32.6 mg, 81%) (*Z/E* = 98: 2). The two isomers are separable via silica gel chromatography.

^{1}H NMR (400 MHz, $CDCl_3$) δ 7.43 – 7.38 (m, 2H), 7.29 (m, 1H), 7.26 – 7.19 (m, 4H), 6.85 (m, 2H), 6.58 (s, 1H), 5.62 (m, 1H), 2.22 (m, 2H), 2.06 (m, 2H), 1.71 (m, 2H), 1.55 (m, 2H).

^{13}C NMR (100 MHz, $CDCl_3$) δ 156.56, 141.00, 139.64, 136.67, 134.35, 131.71, 129.59, 129.16, 128.06, 127.62, 127.45, 125.61, 26.63, 25.51, 22.38, 21.48.

HRMS (ESI) Calcd for $C_{20}H_{19}BrNaO_2S^+$ $[M+Na]^+$: 425.0181, found: 425.0182.

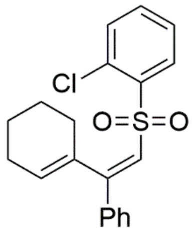

(*E*)-1-chloro-2-((2-(cyclohex-1-en-1-yl)-2-phenylvinyl)sulfonyl)benzene (50a).

According to the general procedure **A**, the crude material was purified by flash chromatography (silica gel, petroleum ether/ ethyl acetate = 5:1) as yellow oil (29.4 mg, 82%) (*E/Z* = 96: 4). The two isomers are separable via silica gel chromatography.

^{1}H NMR (400 MHz, $CDCl_3$) δ 7.39 – 7.30 (m, 2H), 7.23 (d, *J* = 8.0 Hz, 1H), 7.15 (m, 1H), 7.02 (m, 3H), 6.77 (m, 3H), 5.62 (m, 1H), 2.29 (m, 2H), 2.06 (m, 2H), 1.73 (m, 2H), 1.58 (m, 2H).

^{13}C NMR (100 MHz, $CDCl_3$) δ 156.22, 139.52, 139.29, 136.81, 134.10, 133.39, 131.87, 130.91, 130.58, 129.26, 127.88, 127.16, 126.57, 125.26, 26.61, 25.50, 22.41, 21.50.

HRMS (ESI) Calcd for $C_{20}H_{19}ClNaO_2S^+$ $[M+Na]^+$: 381.0686, found: 381.0683.

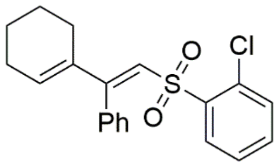

(*Z*)-1-chloro-2-((2-(cyclohex-1-en-1-yl)-2-phenylvinyl)sulfonyl)benzene (50b).

According to the general procedure **B**, the crude material was purified by flash chromatography (silica gel, petroleum ether/ ethyl acetate = 5:1) as yellow oil (30.1 mg, 84%) (*Z/E* > 99: 1).

^{1}H NMR (400 MHz, $CDCl_3$) δ 7.98 – 7.86 (m, 2H), 7.58 (m, 1H), 7.52 (m, 2H), 7.39 – 7.36 (m, 4H), 6.71 (s, 1H), 5.74 – 5.68 (m, 1H), 2.13 (m, 2H), 1.64 – 1.61 (m, 2H), 1.57 – 1.53 (m, 2H), 1.48 – 1.42 (m, 2H).

^{13}C NMR (100 MHz, $CDCl_3$) δ 158.61, 142.66, 136.91, 133.26, 132.88, 131.19, 130.20, 128.80, 128.76, 127.88, 127.53, 127.45, 27.48, 25.23, 22.04, 21.38.

HRMS (ESI) Calcd for $C_{20}H_{19}ClNaO_2S^+$ $[M+Na]^+$: 381.0686, found: 381.0682.

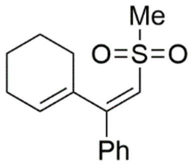

(*E*)-(1-(cyclohex-1-en-1-yl)-2-(methylsulfonyl)vinyl)benzene (51a).

According to the general procedure **A**, the crude material was purified by flash chromatography (silica gel, petroleum ether/ ethyl acetate = 2:1) as yellow oil (19.1 mg, 73%) (*E/Z* > 99: 1).

^{1}H NMR (400 MHz, $CDCl_3$) δ 7.46 – 7.38 (m, 5H), 6.53 (s, 1H), 6.01 – 5.96 (m, 1H), 3.07 (s, 3H), 2.27 (m, 2H), 2.02 (m, 2H), 1.70 (m, 4H).

^{13}C NMR (100 MHz, $CDCl_3$) δ 159.00, 136.71, 134.01, 130.32, 130.29, 128.85, 127.52, 126.19, 44.43, 28.17, 25.35, 22.31, 21.58.

HRMS (ESI) Calcd for $C_{15}H_{18}NaO_2S^+$ $[M+Na]^+$: 285.0920, found: 285.0913.

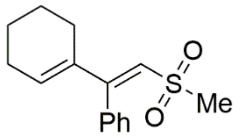

(*Z*)-(1-(cyclohex-1-en-1-yl)-2-(methylsulfonyl)vinyl)benzene (51b).

According to the general procedure **B**, the crude material was purified by flash chromatography (silica gel, petroleum ether/ ethyl acetate = 2:1) as yellow oil (19.9 mg, 76%) (*Z/E* > 99: 1).

^{1}H NMR (400 MHz, $CDCl_3$) δ 7.43 – 7.37 (m, 3H), 7.23 (m, 2H), 6.44 (s, 1H), 5.74 (m, 1H), 2.54 (s, 3H), 2.27 – 2.22 (m, 2H), 2.12 (m, 2H), 1.78 – 1.72 (m, 2H), 1.63 – 1.56 (m, 2H).

^{13}C NMR (100 MHz, $CDCl_3$) δ 155.69, 139.50, 136.57, 134.73, 129.62, 128.61, 127.87, 125.15, 43.72, 26.64, 25.56, 22.43, 21.55.

HRMS (ESI) Calcd for $C_{15}H_{18}NaO_2S^+$ $[M+Na]^+$: 285.0920, found: 285.0921.

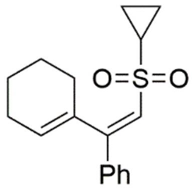

(*E*)-(1-(cyclohex-1-en-1-yl)-2-(cyclopropylsulfonyl)vinyl)benzene (52a).

According to the general procedure **A**, the crude material was purified by flash chromatography (silica gel, petroleum ether/ ethyl acetate = 2:1) as yellow oil (20.2 mg, 70%) (*E/Z* > 99: 1).

^{1}H NMR (400 MHz, $CDCl_3$) δ 7.49 – 7.43 (m, 2H), 7.41 – 7.36 (m, 3H), 6.52 (s, 1H), 6.00 – 5.95 (m, 1H), 2.57 (m, 1H), 2.28 – 2.23 (m, 2H), 2.04 – 2.00 (m, 2H), 1.33 – 1.19 (m, 6H), 1.03 (m, 2H).

^{13}C NMR (100 MHz, $CDCl_3$) δ 158.32, 137.03, 134.22, 130.11, 129.86, 128.78, 127.54, 125.62, 32.81, 28.16, 25.37, 22.37, 21.64, 5.35.

HRMS (ESI) Calcd for $C_{17}H_{20}NaO_2S^+$ $[M+Na]^+$: 311.1076, found: 311.1076.

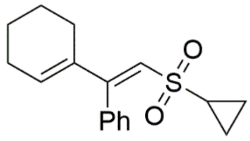

(*Z*)-(1-(cyclohex-1-en-1-yl)-2-(cyclopropylsulfonyl)vinyl)benzene (52b).

According to the general procedure **B**, the crude material was purified by flash chromatography (silica gel, petroleum ether/ ethyl acetate = 2:1) as yellow oil (21.6 mg, 75%) (*Z/E* > 99: 1).

^{1}H NMR (400 MHz, $CDCl_3$) δ 7.40 – 7.33 (m, 3H), 7.26 – 7.21 (m, 2H), 6.43 (s, 1H), 5.71 (m, 1H), 2.25 (m, 2H), 2.15 – 2.08 (m, 2H), 1.93 (m, 1H), 1.78 – 1.71 (m, 2H), 1.60 (m, 2H), 1.02 (m, 2H), 0.78 (m, 2H).

^{13}C NMR (100 MHz, $CDCl_3$) δ 155.14, 138.91, 136.73, 135.35, 129.70, 128.32, 127.59, 124.47, 32.27, 26.61, 25.63, 22.48, 21.59, 5.30.

HRMS (ESI) Calcd for $C_{17}H_{20}NaO_2S^+$ $[M+Na]^+$: 311.1076, found: 311.1078.

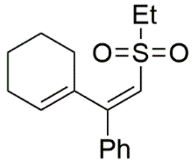

(*E*)-(1-(cyclohex-1-en-1-yl)-2-(ethylsulfonyl)vinyl)benzene (53a).

According to the general procedure **A**, the crude material was purified by flash chromatography (silica gel, petroleum ether/ ethyl acetate = 2:1) as yellow oil (19.3 mg, 70%) (*E/Z* > 99: 1).

^{1}H NMR (400 MHz, $CDCl_3$) δ 7.48 – 7.43 (m, 2H), 7.42 – 7.36 (m, 3H), 6.42 (s, 1H), 5.98 – 5.90 (m, 1H), 3.14 (q, *J* = 7.5 Hz, 2H), 2.28 – 2.22 (m, 2H), 2.05 – 1.99 (m, 2H), 1.70 (m, 4H), 1.41 (t, *J* = 7.5 Hz, 3H).

^{13}C NMR (100 MHz, $CDCl_3$) δ 159.72, 136.94, 133.98, 130.27, 129.89, 128.83, 127.53, 123.94, 50.43, 28.21, 25.34, 22.33, 21.61, 7.25.

HRMS (ESI) Calcd for $C_{16}H_{20}NaO_2S^+$ $[M+Na]^+$: 299.1076, found: 299.1077.

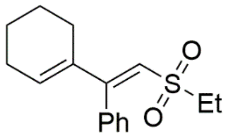

(*Z*)-(1-(cyclohex-1-en-1-yl)-2-(ethylsulfonyl)vinyl)benzene (53b).

According to the general procedure **B**, the crude material was purified by flash chromatography (silica gel, petroleum ether/ ethyl acetate = 2:1) as yellow oil (20.7 mg, 75%) (*Z/E* > 99: 1).

^{1}H NMR (400 MHz, $CDCl_3$) δ 7.38 (m, 3H), 7.25 – 7.18 (m, 2H), 6.31 (s, 1H), 5.73 (m, 1H), 2.57 (q, *J* = 7.5 Hz, 2H), 2.25 (m, 2H), 2.16 – 2.08 (m, 2H), 1.78 – 1.71 (m, 2H), 1.59 (m, 2H), 1.20 (t, *J* = 7.5 Hz, 3H).

^{13}C NMR (100 MHz, $CDCl_3$) δ 156.48, 139.28, 136.75, 134.79, 129.43, 128.50, 127.71, 122.60, 49.39, 26.62, 25.67, 22.45, 21.55, 7.11.

HRMS (ESI) Calcd for $C_{16}H_{20}NaO_2S^+$ $[M+Na]^+$: 299.1076, found: 299.1076.

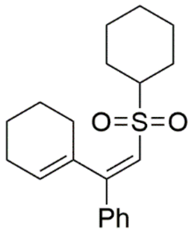

(*E*)-(1-(cyclohex-1-en-1-yl)-2-(cyclohexylsulfonyl)vinyl)benzene (54a).

According to the general procedure **A**, the crude material was purified by flash chromatography (silica gel, petroleum ether/ ethyl acetate = 2:1) as yellow oil (22.8 mg, 69%) (*E/Z* > 99: 1).

^{1}H NMR (400 MHz, $CDCl_3$) δ 7.48 – 7.42 (m, 2H), 7.39 (m, 3H), 6.36 (s, 1H), 5.94 – 5.87 (m, 1H), 2.98 (m, 1H), 2.29 – 2.17 (m, 4H), 2.01 (m, 2H), 1.93 (m, 2H), 1.71 (m, 5H), 1.63 – 1.52 (m, 2H), 1.32 – 1.20 (m, 3H).

^{13}C NMR (100 MHz, $CDCl_3$) δ 159.38, 137.27, 133.74, 130.13, 129.68, 128.78, 127.51, 122.85, 63.17, 28.25, 25.35, 25.31, 25.24, 22.41, 21.66.

HRMS (ESI) Calcd for $C_{20}H_{26}NaO_2S^+$ $[M+Na]^+$: 353.1546, found: 353.1549.

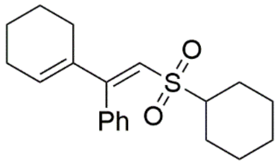

(*Z*)-(1-(cyclohex-1-en-1-yl)-2-(cyclohexylsulfonyl)vinyl)benzene (54b).

According to the general procedure **B**, the crude material was purified by flash chromatography (silica gel, petroleum ether/ ethyl acetate = 2:1) as yellow oil (21.5 mg, 65%) (*Z/E* > 99: 1).

^{1}H NMR (400 MHz, $CDCl_3$) δ 7.38 – 7.37 (m, 3H), 7.21 – 7.18 (m, 2H), 6.24 (s, 1H), 5.72 (m, 1H), 2.24 (m, 2H), 2.12 (m, 2H), 1.96 (s, 2H), 1.80 (s, 2H), 1.75 (m, 2H), 1.63 – 1.57 (m, 5H), 1.42 (m, 2H), 1.10 (m, 2H).

^{13}C NMR (100 MHz, $CDCl_3$) δ 156.32, 138.87, 136.88, 135.09, 129.37, 128.29, 127.55, 121.32, 61.99, 26.58, 25.74, 25.18, 25.10, 24.89, 22.48, 21.57.

HRMS (ESI) Calcd for $C_{20}H_{26}NaO_2S^+$ $[M+Na]^+$: 353.1546, found: 353.1547.

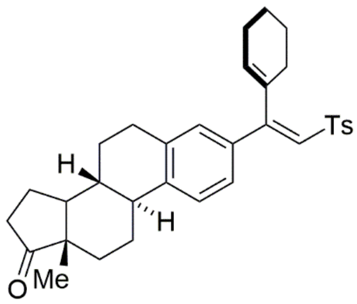

(8*R*,9*S*,13*S*)-3-((*E*)-1-(cyclohex-1-en-1-yl)-2-tosylvinyl)-13-methyl-6,7,8,9,11,12,13,14,15,16-decahydro-17*H*-cyclopenta[*a*]phenanthren-17-one (55a).

According to the general procedure **A**, the crude material was purified by flash chromatography (silica gel, petroleum ether/ ethyl acetate = 2:1) as yellow oil (31.9 mg, 62%) (*E/Z* > 99: 1).

^{1}H NMR (400 MHz, $CDCl_3$) δ 7.78 (d, *J* = 8.2 Hz, 2H), 7.31 (d, *J* = 8.2 Hz, 2H), 7.27 (m, 1H), 7.15 (m, 2H), 6.67 (s, 1H), 5.65 (m, 1H), 2.96 – 2.86 (m, 2H), 2.50 (m, 1H), 2.43 (s, 3H), 2.40 – 2.36 (m, 1H), 2.29 (m, 1H), 2.14 – 2.10 (m, 2H), 2.08 – 2.00 (m, 2H), 1.96 (m, 1H), 1.71 – 1.62 (m, 3H), 1.62 – 1.51 (m, 5H), 1.52 – 1.42 (m, 5H), 0.90 (s, 3H).

^{13}C NMR (100 MHz, $CDCl_3$) δ 158.15, 143.67, 142.29, 139.87, 137.05, 134.40, 133.29, 130.60, 129.38, 127.97, 127.86, 126.88, 125.77, 124.95, 50.47, 47.91, 44.47, 37.95, 35.83, 31.56, 29.40, 27.66, 26.34, 25.62, 25.21, 22.08, 21.62, 21.58, 21.45, 13.83.

HRMS (ESI) Calcd for $C_{33}H_{38}NaO_3S^+$ $[M+Na]^+$: 537.2434, found: 537.2437.

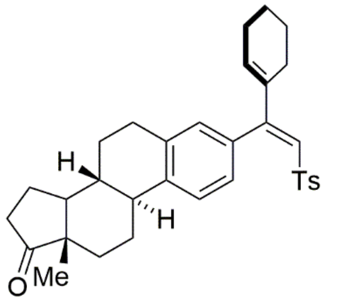

(8*R*,9*S*,13*S*)-3-((*Z*)-1-(cyclohex-1-en-1-yl)-2-tosylvinyl)-13-methyl-6,7,8,9,11,12,13,14,15,16-decahydro-17*H*-cyclopenta[*a*]phenanthren-17-one (55b).

According to the general procedure **B**, the crude material was purified by flash chromatography (silica gel, petroleum ether/ ethyl acetate = 2:1) as yellow oil (33.4 mg, 65%) (*Z/E* = 2.5: 1). The two isomers are separable via silica gel chromatography.

^{1}H NMR (400 MHz, $CDCl_3$) δ 7.31 – 7.27 (m, 2H), 7.12 (m, 1H), 7.06 (d, J = 8.2 Hz, 2H), 6.74 (m, 1H), 6.57 (s, 1H), 6.41 (s, 1H), 5.67 (m, 1H), 2.69 (m, 2H), 2.52 (m, 1H), 2.42 (m, 1H), 2.37 (s, 3H), 2.31 (m, 1H), 2.18 (m, 3H), 2.09 – 1.94 (m, 5H), 1.69 (m, 3H), 1.55 (m, 6H), 1.42 (m, 1H), 0.97 (s, 3H).

^{13}C NMR (100 MHz, $CDCl_3$) δ 155.76, 142.94, 139.29, 139.15, 138.58, 136.67, 135.16, 131.89, 129.80, 128.85, 127.63, 127.22, 126.07, 124.18, 50.50, 47.95, 44.40, 38.14, 35.85, 31.60, 29.14, 26.54, 26.48, 25.69, 25.53, 22.44, 21.62, 21.56, 21.55, 13.93.

HRMS (ESI) Calcd for $C_{33}H_{38}NaO_3S^+$ $[M+Na]^+$: 537.2434, found: 537.2440.

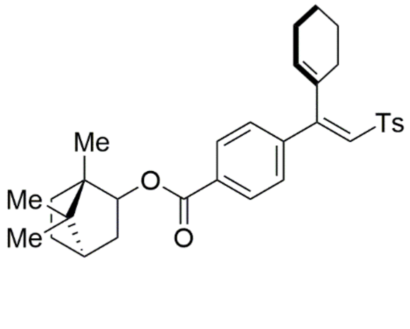

(1*S*,4*R*)-1,7,7-trimethylbicyclo[2.2.1]heptan-2-yl 4-((*E*)-1-(cyclohex-1-en-1-yl)-2-tosylvinyl)benzoate (56a).

According to the general procedure **A**, the crude material was purified by flash chromatography (silica gel, petroleum ether/ ethyl acetate = 2:1) as yellow oil (33.7 mg, 65%) (*E/Z* > 99: 1).

^{1}H NMR (400 MHz, $CDCl_3$) δ 7.96 (d, *J* = 8.2 Hz, 2H), 7.73 (d, *J* = 8.2 Hz, 2H), 7.38 (d, *J* = 8.2 Hz, 2H), 7.26 (d, *J* = 8.2 Hz, 2H), 6.66 (s, 1H), 5.70 (m, 1H), 5.06 – 5.01 (m, 1H), 2.44 – 2.35 (m, 4H), 2.07 (m, 2H), 2.05 – 1.99 (m, 1H), 1.77 – 1.70 (m, 1H), 1.67 (m, 1H), 1.55 (m, 2H), 1.50 (m, 2H), 1.41 (m, 2H), 1.26 – 1.19 (m, 2H), 1.03 (m, 1H), 0.89 (s, 3H), 0.84 (m, 6H).

^{13}C NMR (100 MHz, $CDCl_3$) δ 166.14, 156.85, 144.03, 141.36, 139.32, 132.94, 132.17, 131.54, 129.87, 129.52, 129.26, 128.00, 127.52, 80.91, 49.13, 47.92, 44.98, 36.90, 28.09, 27.47, 27.40, 25.26, 22.01, 21.64, 21.35, 19.73, 18.92, 13.62.

HRMS (ESI) Calcd for $C_{32}H_{38}NaO_4S^+$ $[M+Na]^+$: 541.2383, found: 541.2385.

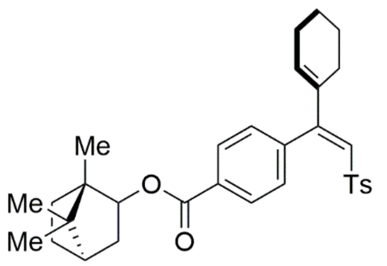

(1*S*,4*R*)-1,7,7-trimethylbicyclo[2.2.1]heptan-2-yl 4-((*Z*)-1-(cyclohex-1-en-1-yl)-2-tosylvinyl)benzoate (56b)

According to the general procedure **B**, the crude material was purified by flash chromatography (silica gel, petroleum ether/ ethyl acetate = 2:1) as yellow oil (34.7 mg, 67%) (*Z/E* = 2.5: 1). The two isomers are separable via silica gel chromatography.

^{1}H NMR (400 MHz, $CDCl_3$) δ 7.86 (d, *J* = 8.2 Hz, 2H), 7.31 (d, *J* = 8.2 Hz, 2H), 7.06 (d, *J* = 8.2 Hz, 2H), 6.94 (d, *J* = 8.2 Hz, 2H), 6.49 (s, 1H), 5.48 (m, 1H), 5.11 – 5.02 (m, 1H), 2.48 – 2.39 (m, 1H), 2.32 (s, 3H), 2.14 (m, 2H), 2.11 – 2.04 (m, 1H), 1.98 (m, 2H), 1.80 – 1.71 (m, 1H), 1.69 (m, 1H), 1.64 (m, 2H), 1.52 – 1.45 (m, 2H), 1.40 – 1.32 (m, 1H), 1.30 – 1.23 (m, 1H), 1.07 (m, 1H), 0.92 (s, 3H), 0.87 (m, 6H).

^{13}C NMR (100 MHz, $CDCl_3$) δ 166.50, 154.22, 143.75, 139.61, 139.40, 138.99, 136.44, 130.30, 129.53, 129.36, 128.54, 127.59, 126.05, 80.69, 49.14, 47.94, 45.02, 36.96, 28.14, 27.44, 26.62, 25.50, 22.36, 21.58, 21.47, 19.76, 18.95, 13.68.

HRMS (ESI) Calcd for $C_{32}H_{38}NaO_4S^+$ $[M+Na]^+$: 541.2383, found: 541.2382.

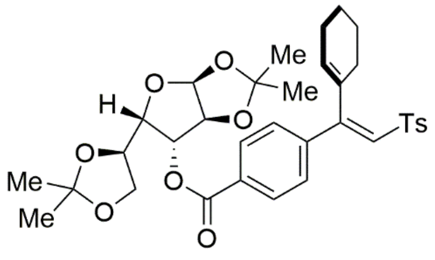

(3a*S*,5*S*,6*R*,6a*S*)-5-((*R*)-2,2-dimethyl-1,3-dioxolan-4-yl)-2,2-dimethyltetrahydrofuro[2,3-*d*][1,3]dioxol-6-yl 4-((*E*)-1-(cyclohex-1-en-1-yl)-2-tosylvinyl)benzoate (57a).

According to the general procedure **A**, the crude material was purified by flash chromatography (silica gel, petroleum ether/ ethyl acetate = 2:1) as yellow oil (40.6 mg, 65%) (*E/Z* > 99: 1).

^{1}H NMR (400 MHz, $CDCl_3$) δ 7.92 (d, *J* = 8.2 Hz, 2H), 7.73 (d, *J* = 8.2 Hz, 2H), 7.39 (d, *J* = 8.2 Hz, 2H), 7.26 (d, *J* = 8.2 Hz, 2H), 6.66 (s, 1H), 5.87 (m, 1H), 5.71 (s, 1H), 5.42 (m, 1H), 4.55 (m, 1H), 4.27 – 4.24 (m, 2H), 4.02 (m, 2H), 2.38 (s, 3H), 2.09 (m, 2H), 1.55 (s, 4H), 1.48 (s, 3H), 1.43 – 1.38 (m, 2H), 1.34 (s, 3H), 1.25 (s, 3H), 1.19 (s, 3H).

^{13}C NMR (100 MHz, $CDCl_3$) δ 164.57, 156.48, 144.12, 142.13, 139.19, 132.79, 131.74, 130.74, 130.07, 129.68, 129.55, 128.02, 127.69, 112.45, 109.47, 105.13, 83.35, 79.92, 72.56, 67.31, 27.46, 26.86, 26.73, 26.22, 25.24, 21.99, 21.65, 21.32.

HRMS (ESI) Calcd for $C_{34}H_{40}NaO_9S^+$ $[M+Na]^+$: 647.2285, found: 647.2289.

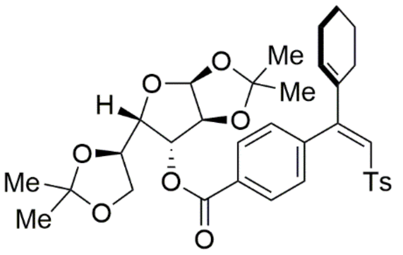

(3a*S*,5*S*,6*R*,6a*S*)-5-((*R*)-2,2-dimethyl-1,3-dioxolan-4-yl)-2,2-dimethyltetrahydrofuro[2,3-*d*][1,3]dioxol-6-yl 4-((*Z*)-1-(cyclohex-1-en-1-yl)-2-tosylvinyl)benzoate (57b).

According to the general procedure **B**, the crude material was purified by flash chromatography (silica gel, petroleum ether/ ethyl acetate = 2:1) as yellow oil (41.8 mg, 67%) (*Z/E* = 1.4: 1). The two isomers are inseparable via silica gel chromatography.

^{1}H NMR (400 MHz, $CDCl_3$) mixtures of *Z*/*E* isomers: δ 7.88 (m, 2H), 7.73 – 7.60 (m, 1H), 7.39 – 7.31 (m, 2H), 7.26 (d, *J* = 8.2 Hz, 1H), 7.10 (d, *J* = 8.2 Hz, 1H), 6.99 (d, *J* = 8.2 Hz, 1H), 6.66 (s, 0.42H), 6.47 (s, 0.58H), 5.87 (m, 1.42H), 5.71 (m, 0.58H), 5.45 – 5.41 (m, 1H), 4.56 (m, 1H), 4.36 – 4.29 (m, 0.84H), 4.28 – 4.24 (m, 1.16H), 4.07 – 4.00 (m, 2H), 2.37 (s, 1.26H), 2.33 (s, 1.74H), 2.15 – 2.06 (m, 2H), 1.63 (m, 2.12H), 1.55 (m, 1.68H), 1.50 (s, 1.26H), 1.48 (s, 1.74H), 1.43 – 1.33 (m, 5H), 1.27 (s, 1.25H), 1.24 (s, 1.73H), 1.21 – 1.15 (m, 3H).

^{13}C NMR (100 MHz, $CDCl_3$) mixtures of *Z*/*E* isomers: δ 164.94 (164.56), (156.47) 153.83, (144.12) 143.91, (142.12) 140.55, 139.52 (139.18), 139.04, 136.40 (132.78), (131.73) 131.64, (130.74) 130.35, (130.06), (129.70), (129.55) 129.47, 128.99, 128.76 (128.01), (127.69) 127.54, 125.86, (112.43) 112.41, (109.49), (109.46), 105.14, 83.40 (83.34), 79.90, 72.60 (72.56), (67.30) 67.27, (27.45) 26.95, (26.86), (26.74), 26.62, (26.22), 25.50, 25.33, (25.24), 22.32, (21.99), 21.64, 21.59, 21.43 (21.32).

HRMS (ESI) Calcd for $C_{34}H_{40}NaO_9S^+$ $[M+Na]^+$: 647.2285, found: 647.2282.

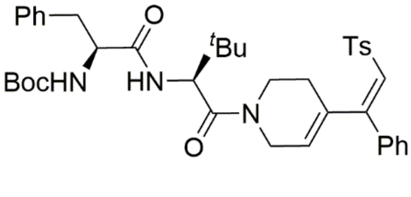

***Tert*-butyl ((*S*)-1-(((*S*)-3,3-dimethyl-1-oxo-1-(4-((*E*)-1-phenyl-2-tosylvinyl)-3,6-dihydropyridin-1(2*H*)-yl)butan-2-yl)amino)-1-oxo-3-phenylpropan-2-yl)carbamate (58a).**

According to the general procedure **A**, the crude material was purified by flash chromatography (silica gel, petroleum ether/ ethyl acetate = 1:1) as yellow oil (48.9 mg, 70%) (*E*/*Z* > 99: 1).

^{1}H NMR (400 MHz, $CDCl_3$) (Note: Two isomeric rotamers of amide exist, This phenomenon is seen with many tertiary amides[3]) δ 7.78 (m, 2H), 7.35 (m, 7H), 7.27 (m, 2H), 7.19 (m, 3H), 6.79 (m, 0.64H), 6.73 (m, 1.36H), 5.63 (m, 0.68H), 5.58 (m, 0.32H), 5.14 – 4.98 (m, 1H), 4.89 (m, 0.68H), 4.86 – 4.83 (m, 0.32H), 4.31 (m, 2.36H), 4.11 – 3.99 (m, 0.64H), 3.85 – 3.41 (m, 2H), 3.08 (m, 2H), 2.50 – 2.39 (m, 3H), 2.16 – 1.99 (m, 2H), 1.42 – 1.36 (m, 9H), 0.95 (m, 9H).

^{13}C NMR (100 MHz, $CDCl_3$) (Note: Two isomeric rotamers of amide exist, This phenomenon is seen with many tertiary amides[3]) δ (171.07, 170.94, 170.84), (170.21, 169.95, 169.76, 169.54), (155.38, 155.18), (144.35, 144.32, 144.29), (139.60, 139.54, 139.50), (136.72, 136.65), (135.82, 135.75), 133.39, (131.92, 131.75), (130.64, 130.58), (129.81, 129.76), (129.30, 129.21, 129.17), (128.99, 128.97, 128.70, 128.58), (128.17, 128.00), (127.60, 127.54), (127.42, 127.40, 127.38), (126.93, 126.83), (125.90, 125.81), 124.65, 80.13, 55.97, 54.95, (54.43, 54.35), (45.65, 45.61), (43.25, 43.07, 42.06), (38.45, 37.91, 35.97, 35.77), (28.95, 28.89, 28.27, 28.23), (26.57, 26.46), 21.63.

HRMS (ESI) Calcd for $C_{40}H_{49}N_3NaO_6S^+$ $[M+Na]^+$: 722.3234, found: 722.3237.

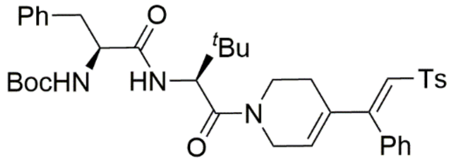

***Tert*-butyl ((*S*)-1-(((*S*)-3,3-dimethyl-1-oxo-1-(4-((*Z*)-1-phenyl-2-tosylvinyl)-3,6-dihydropyridin-1(2*H*)-yl)butan-2-yl)amino)-1-oxo-3-phenylpropan-2-yl)carbamate (58b).**

According to the general procedure **B**, the crude material was purified by flash

chromatography (silica gel, petroleum ether/ ethyl acetate = 1:1) as yellow oil (53.8 mg, 77%) (*Z/E* = 2: 1). The two isomers are inseparable via silica gel chromatography.

^{1}H NMR (400 MHz, $CDCl_3$) (mixtures of *Z*/*E* isomers and isomeric rotamers of amide, note: Two isomeric rotamers of amide exist, This phenomenon is seen with many tertiary amides[3]) δ 7.65 – 7.59 (m, 2H), 7.24 – 7.16 (m, 10H), 7.08 – 7.00 (m, 2H), 6.99 – 6.81 (m, 1H), 6.71 (brs, 1H), 5.90 – 5.46 [5.9 (m, 0.2H), 5.63(m, 0.26H), 5.59(m, 0.12H), 5.48(m, 0.45H)], 5.04 (m, 1H), 4.96 – 4.81 (m, 1H), 4.48 – 4.38 (m, 1H), 4.36 – 4.25 (m, 2H), 4.20 – 3.89 (m, 1H), 3.70 (m, 0.38H), 3.26 (m, 0.66 H), 3.06 (m, 2H), 2.75 – 2.39 (m, 2H), 2.37 (m, 3H), 1.40 (m, 9H), 0.90 (m, 9H).

^{13}C NMR (100 MHz, $CDCl_3$) (mixtures of *Z*/*E* isomers and isomeric rotamers of amide, note: Two isomeric rotamers of amide exist, This phenomenon is seen with many tertiary amides[3]) δ (171.19, 171.11, 171.09, 171.06), (169.22, 169.00, 168.86, 168.77, 168.57, 168.47), (155.35, 155.18), (144.61, 144.50), (139.62, 139.45), (136.59, 136.53), (136.36, 136.26), (134.50, 134.38), (134.19, 134.13), (129.68, 129.65, 129.63, 129.59), (129.22, 129.15), (128.76, 128.62), (128.32, 128.25), (128.09, 127.94), 127.15, (127.02, 126.86), (122.24, 121.80), (109.17, 109.01), (107.09, 106.93), (80.20, 71.81), (61.30, 60.90, 59.06, 56.00), (54.76, 54.72), (44.35, 44.13), (40.56, 40.33), (38.30, 37.99), (36.16, 36.08, 35.99, 35.91), (28.26, 28.24, 27.33), (26.62, 26.56, 26.53, 26.45, 26.42, 26.35, 26.31, 26.26), 21.58.

HRMS (ESI) Calcd for $C_{40}H_{49}N_3NaO_6S^+$ $[M+Na]^+$: 722.3234, found: 722.3233.

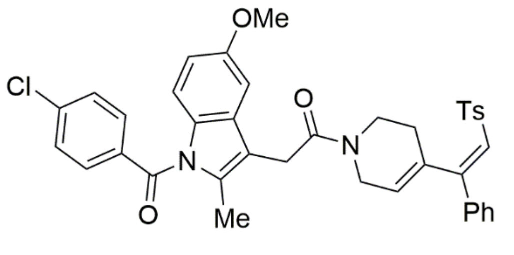

(*E*)-2-(1-(4-chlorobenzoyl)-5-methoxy-2-methyl-1*H*-indol-3-yl)-1-(4-(1-phenyl-2-tosylvinyl)-3,6-dihydropyridin-1(2*H*)-yl)ethan-1-one (59a).

According to the general procedure A, the crude material was purified by flash chromatography (silica gel, petroleum ether/ ethyl acetate = 1:1) as yellow oil (44.1 mg,

65%) (*E/Z* > 99: 1).

^{1}H NMR (400 MHz, $CDCl_3$) (Note: Two isomeric rotamers of amide exist) δ 7.73 (m, 2H), 7.66 (m, 0.7H), 7.59 (m, 1.3H), 7.46 – 7.35 (m, 3H), 7.32 (m, 3H), 7.26 (m, 3H), 7.09 (m, 0.35H), 7.04 (m, 0.65H), 6.88 (m, 0.35H), 6.78 (m, 0.65H), 6.75 – 6.63 (m, 2H), 5.79 (m, 0.35H), 5.47 (m, 0.65H), 4.28 – 4.14 (m, 2H), 3.84 – 3.75 (m, 5H), 3.65 (m, 0.7H), 3.59 (m, 1.3H), 2.46 – 2.37 (m, 6H), 1.92 (m, 2H).

^{13}C NMR (100 MHz, $CDCl_3$) (Note: Two isomeric rotamers of amide exist) δ (169.23, 169.07), (168.34, 168.24), (156.10, 156.05, 155.47, 154.95), (144.41, 144.27), 139.62, (139.22, 139.13), (135.78, 135.59), (135.26, 135.18), (133.93, 133.83), 132.90, 131.78, (131.22, 131.20), 130.93, (130.80, 130.77), (130.65, 130.58), 129.77, 129.15, (129.11, 129.02), (128.27, 127.96), (127.60, 127.56), (127.38, 127.29), (125.99, 125.85), 114.96, (113.03, 112.99), (111.79, 111.73), (101.57, 101.32), 55.75, (44.97, 42.56, 42.17), 38.44, (31.11, 30.87), (28.83, 27.65), 21.64, (13.57, 13.38).

HRMS (ESI) Calcd for $C_{39}H_{35}ClN_2NaO_5S^+$ [M+Na]$^+$: 701.1847, found: 701.1849.

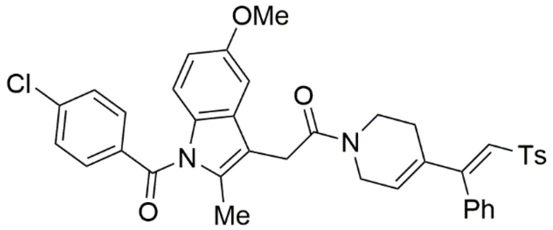

(*Z*)-2-(1-(4-chlorobenzoyl)-5-methoxy-2-methyl-1*H*-indol-3-yl)-1-(4-(1-phenyl-2-tosylvinyl)-3,6-dihydropyridin-1(2*H*)-yl)ethan-1-one (59b).

According to the general procedure **B**, the crude material was purified by flash chromatography (silica gel, petroleum ether/ ethyl acetate = 1:1) as yellow oil (48.8 mg, 72%) (*Z/E* = 1.5: 1). The two isomers are inseparable via silica gel chromatography.

^{1}H NMR (400 MHz, $CDCl_3$) (mixtures of *Z*/*E* isomers and isomeric rotamers of amide) δ 7.55 (m, 6H), 7.29 – 7.04 (m, 7H), 6.99 – 6.81 (m, 3H), 6.66 (m, 1H), 5.94 (m, 0.27H), 5.81 – 5.73 (m, 0.16H), 5.60 – 5.45 (m, 0.21H), 5.39 (m, 0.33H), 4.42 – 4.04 (m, 2H), 3.92 – 3.45 (m, 7H), 2.61 (m, 1H), 2.46 – 2.10 (m, 7H).

^{13}**C NMR** (100 MHz, $CDCl_3$) (mixtures of *Z*/*E* isomers and isomeric rotamers of amide) δ (169.01, 168.28, 168.24), 167.76, 156.09, (145.71, 144.67, 144.46), (139.65, 139.57, 139.30), (136.62, 136.44, 135.57, 135.43), (134.62, 134.58), (133.93, 133.87), 133.07, 132.35, 131.22, 130.87, (130.63, 130.55), 130.03, (129.76, 129.69, 129.63, 129.55), (129.17, 129.13, 129.09, 129.03), (128.20, 128.18, 128.16, 128.02), 127.77, (127.17, 127.01, 126.85, 126.55), (122.15, 121.50), (115.02, 114.95), 112.54, (111.71, 111.66), (109.10, 108.51), 106.82, (100.37, 100.29), (61.25, 60.96), 55.74, (43.50, 40.42, 40.16), (30.69, 30.12, 29.95), (27.10, 26.43), (21.73, 21.60, 21.56, 21.15), (13.57, 13.50).

HRMS (ESI) Calcd for $C_{39}H_{35}ClN_2NaO_5S^+$ [M+Na]$^+$: 701.1847, found: 701.1848.

2.3 Synthetic applicability.

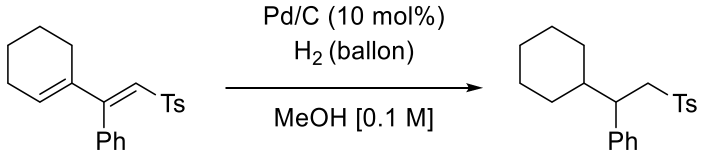

The preparation for compound **60** is based on previously reported procedure[4]. To a stirred solution of **4b** (0.1 mmol, 1.0 equiv.) in MeOH (1.0 ml) was added 10% Pd on carbon (0.01 mmol, 10 mol%). The reaction mixture was stirred vigorously under an atmosphere of hydrogen balloon at room temperature overnight. Upon completion of the reaction, the mixture was filtered through a short pad of celite with ethyl acetate as the eluent, and the filtrate was concentrated under reduced pressure. The product was purified by flash column chromatography on silica gel to afford product **60** as a colorless liquid (32.4 mg, 95% yield).

^{1}H NMR (400 MHz, $CDCl_3$) δ 7.41 (d, J = 8.2 Hz, 2H), 7.03 (m, 5H), 6.82 (d, J = 8.2 Hz, 2H), 3.56 – 3.38 (m, 2H), 2.99 – 2.88 (m, 1H), 2.28 (s, 3H), 1.67 (m, 1H), 1.53 (m, 2H), 1.40 – 1.29 (m, 2H), 1.23 – 1.14 (m, 2H), 1.03 – 0.91 (m, 2H), 0.82 – 0.75 (m, 2H).

^{13}C NMR (101 MHz, $CDCl_3$) δ 144.23, 141.50, 136.60, 129.54, 128.72, 128.02, 127.62, 126.86, 60.58, 32.81, 31.95, 25.53, 24.24, 24.20, 21.48.

HRMS (ESI) Calcd for $C_{21}H_{26}NaO_2S^+$ $[M+Na]^+$: 365.1546, found: 365.1547.

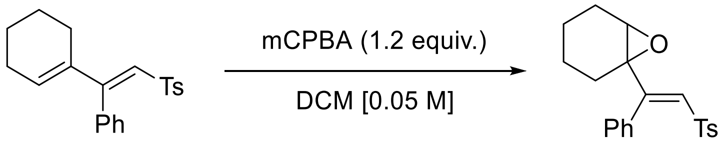

The preparation for compound **61** is based on previously reported procedure[5]. To a stirred solution of **4b** (0.1 mrnol, 1.0 equiv.) and m-chloroperbenzoic acid (0.12 mmol, 1.2 equiv.), DCM (2.0 ml) was refluxed for 48 h, then concentrated at room temperature. The product was purified by flash column chromatography on silica gel to afford product **61** as a colorless liquid (25.1 mg, 71% yield).

^{1}H NMR (400 MHz, $CDCl_3$) δ 7.43 (d, *J* = 8.2 Hz, 2H), 7.33 (m, 3H), 7.18 – 7.11 (m, 4H), 6.75 (s, 1H), 3.06 (m, 1H), 2.37 (s, 3H), 1.99 – 1.86 (m, 2H), 1.71 – 1.62 (m, 2H), 1.42 – 1.31 (m, 2H), 1.17 – 1.02 (m, 2H).

^{13}C NMR (101 MHz, $CDCl_3$) δ 156.67, 143.87, 138.48, 133.88, 129.40, 129.01, 128.44, 128.13, 128.08, 127.58, 61.91, 61.62, 26.35, 24.49, 21.57, 19.72, 18.84.

HRMS (ESI) Calcd for $C_{21}H_{22}NaO_3S^+$ $[M+Na]^+$: 377.1182, found: 377.1185.

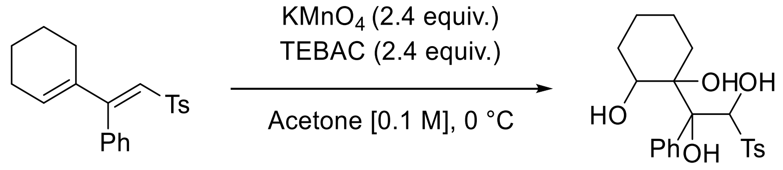

The preparation for compound **62** is based on previously reported procedure[6]. TEBAC (0.24 mmol, 2.4 equiv.) and $KMnO_4$ (0.24 mmol, 2.4 equiv.) were mixed in acetone (0.5 mL), and the mixture was stirred at r.t. for 3 h then cooled to 0 °C. A solution of the compound **4b** (0.1 mmol, 1.0 equiv.) in acetone (0.5 mL) was added dropwise over 5 min while the internal temperature was kept at 5 °C or below. After completion of the addition, the mixture was stirred for a further 30 min at 0 °C until the compound **4b** was completely consumed (TLC). Sat. aq $NaHSO_3$ (5 mL) was added in one portion to quench the reaction, and the mixture was filtered through Celite, which was washed with acetone. The filtrate was concentrated and the residue was extracted with EtOAc. The organic phase was dried (Na_2SO_4), filtered, and evaporated to dryness. The product was purified by flash column chromatography on silica gel to afford product **62** as a colorless liquid (30.1 mg, 74% yield).

^{1}H NMR (400 MHz, $CDCl_3$) δ 7.34 – 7.26 (m, 2H), 7.27 – 7.07 (m, 5H), 7.07 (d, *J* = 8.0 Hz, 2H), 6.89 (d, *J* = 13.0 Hz, 1H), 3.42 (dd, *J* = 11.0, 4.8 Hz, 1H), 2.32 (s, 3H), 1.66 – 1.33 (m, 7H), 0.99 – 0.85 (m, 1H).

^{13}C NMR (101 MHz, $CDCl_3$) δ 161.95, 143.68, 138.66, 133.55, 131.14, 129.34, 128.75, 128.03, 127.63, 69.93, 34.08, 30.11, 23.66, 21.58, 20.12.

HRMS (ESI) Calcd for $C_{21}H_{27}O_6S^+$ $[M+H]^+$: 407.1523, found: 407.1527.

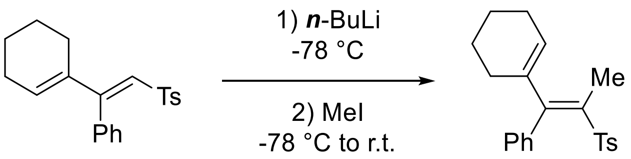

The preparation for compound **63** is based on previously reported procedure[7]. Compound **4b** (0.1 mmol, 1.0 equiv.) and THF (3 mL) were placed under N_2 in a Schlenk tube containing a magnetic stirring bar, the mixture was cooled to –78 °C, and nBuLi (0.11 mL, 1.1 equiv.) was added. After the mixture had been stirred for 30 min at this temperature, MeI (0.12 mmol, 1.2 equiv.) was added, and the reaction mixture was stirred for a further hour at the same temperature. The cool bath was removed, saturated aq. NH_4Cl (5 mL) was added, and the reaction mixture was extracted with ethyl acetate. The combined organic layer was washed with brine and dried with $MgSO_4$. After removal of solvent under reduced pressure, The product was purified by flash column chromatography on silica gel to afford product **63** as a colorless liquid (23.9 mg, 68% yield).

^{1}H NMR (400 MHz, $CDCl_3$) δ 7.77 (d, *J* = 8.3 Hz, 2H), 7.32 (m, 5H), 7.14 (m, 2H), 5.75 – 5.66 (m, 1H), 2.43 (s, 3H), 2.09 – 2.04 (m, 2H), 2.01 (s, 3H), 1.53 (td, *J* = 6.0, 1.9 Hz, 2H), 1.43 (m, 2H), 1.32 (m, 2H).

^{13}C NMR (101 MHz, $CDCl_3$) δ 154.19, 143.47, 139.41, 138.79, 137.16, 135.25, 129.31, 128.52, 128.33, 128.23, 127.99, 27.01, 25.31, 22.05, 21.60, 21.34, 17.44.

HRMS (ESI) Calcd for $C_{22}H_{24}NaO_2S^+$ $[M+Na]^+$: 375.1389, found: 375.1391.

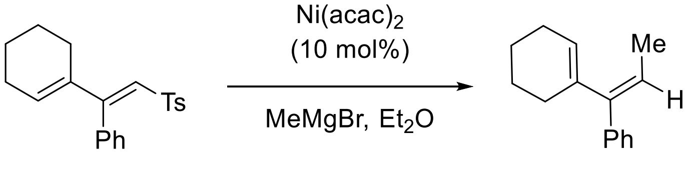

The preparation for compound **64** is based on previously reported procedure[8].

A flame-dried round bottom flask under nitrogen was charged with compound **4b** (0.1 mmol, 1.0 equiv.) and $Ni(acac)_2$ (0.01 mmol, 10 mol%). Dry Et_2O (3.0 mL) was added and the mixture was cooled down to -78°C. The mixture was sparged with N_2 for 5 minutes, and methylmagnesium bromide (0.3 mmol, 3.0 equiv.) was added slowly, and the reaction was stirred at room temperature for 2 h. The product was purified by flash

column chromatography on silica gel to afford product **64** as a colorless liquid (15.9 mg, 80% yield).

^{1}H NMR (400 MHz, $CDCl_3$) δ 7.35 (m, 2H), 7.29 (m, 2H), 7.20 (m, 1H), 5.86 (m, 1H), 5.61 (m, 1H), 2.22 – 2.16 (m, 2H), 1.94 – 1.87 (m, 2H), 1.81 (d, J = 6.9 Hz, 3H), 1.67 (m, 4H).

^{13}C NMR (101 MHz, $CDCl_3$) δ 144.65, 141.35, 135.73, 128.12, 127.12, 126.54, 126.40, 121.93, 28.41, 25.42, 23.00, 22.40, 15.37.

HRMS (ESI) Calcd for $C_{15}H_{19}^+$ $[M+Na]^+$: 199.1481, found: 199.1480.

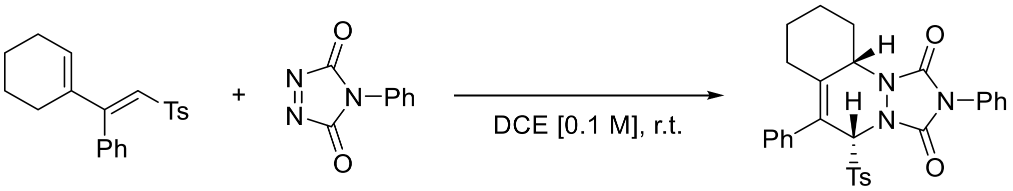

The preparation for compound **66** is based on previously reported procedure[9]. In a glovebox, compound **4b** (0.1 mmol, 1.0 equiv.), 4-phenyl-1,2,4-triazoline-3,5-dione (PTAD) (0.12 mmol, 1.2 equiv.), and 1,2-dichloroethane (1.0 mL) were added to a 4 mL vial with a Teflon-sealed screwcap. The mixture was stirred under dark at room temperature for 3 h. Then, this mixture was then evaporated to dryness, and the residue was purified by flash column chromatography (petroleum ether : ethyl acetate= 3:1) to give product **66** as a colorless oil (37.6 mg, 73%)

^{1}H NMR (400 MHz, $CDCl_3$) δ 7.41 (m, 4H), 7.34 – 7.29 (m, 1H), 7.26 (m, 5H), 7.18 (m, 2H), 7.00 (m, 2H), 5.84 (s, 1H), 4.25 (m, 1H), 3.18 – 3.02 (m, 1H), 2.66 (m, 1H), 2.23 (s, 3H), 1.92 (m, 3H), 1.62 (m, 3H).

^{13}C NMR (101 MHz, $CDCl_3$) δ 152.88, 150.02, 144.89, 139.63, 136.12, 135.89, 131.05, 129.86, 129.60, 129.20, 128.59, 128.44, 128.37, 125.89, 120.66, 76.24, 59.56, 34.33, 29.85, 27.88, 24.03, 21.59.

HRMS (ESI) Calcd for $C_{29}H_{27}N_3NaO_4S^+$ $[M+Na]^+$: 536.1614, found: 536.1612.

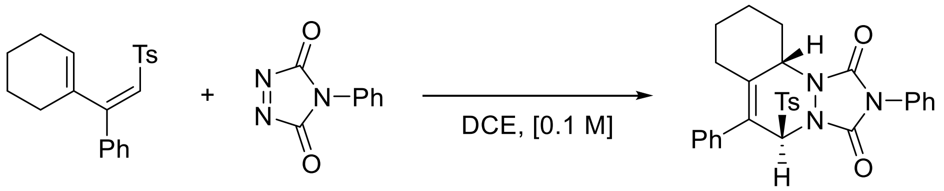

The preparation for compound **66’** is based on previously reported procedure[9]. In a glovebox, compound **4a** (0.1 mmol, 1.0 equiv.), 4-phenyl-1,2,4-triazoline-3,5-dione (PTAD) (0.12 mmol, 1.2 equiv.), and 1,2-dichloroethane (1.0 mL) were added to a 4 mL vial with a Teflon-sealed screwcap. The mixture was stirred under dark at room temperature for 3 days. Then, this mixture was then evaporated to dryness, and the residue was purified by flash column chromatography (petroleum ether : ethyl acetate= 3:1) to give product **66’** as a colorless oil (24.6 mg, 48%)

^{1}H NMR (400 MHz, $CDCl_3$) δ 7.91 – 7.71 (m, 4H), 7.53 – 7.44 (m, 4H), 7.43 – 7.37 (m, 5H), 7.24 (d, *J* = 8.2 Hz, 2H), 6.98 (s, 1H), 4.96 (m, 1H), 2.74 (m, 1H), 2.35 (s, 3H), 2.03 – 1.94 (m, 2H), 1.72 (m, 1H), 1.66 – 1.58 (m, 1H), 1.45 (m, 2H).

^{13}C NMR (101 MHz, $CDCl_3$) δ 146.39, 145.28, 144.30, 135.20, 133.11, 130.71, 130.30, 129.92, 129.20, 129.07, 128.73, 128.32, 124.96, 120.82, 113.25, 70.11, 52.48, 27.97, 26.59, 23.47, 21.66, 20.98.

HRMS (ESI) Calcd for $C_{29}H_{27}N_3NaO_4S^+$ $[M+Na]^+$: 536.1614, found: 536.1611.

2.4 Mechanistic Studies.

2.4.1 Stern-Volmer quenching experiments.

Stern-Volmer quenching experiments with substrates.

Stern-Volmer quenching experiments were carried by Edinburgh Fluorescence Spectrometer FS5, using a 0.01 mM solution of $Ru(dtbbpy)_3(PF_6)_2$ with variable concentrations (0.5, 1.0, 1.5, 2.0, 2.5 mM) of cyclohex-1-en-1-yl trifluoromethanesulfonate **(2)**, ethynylbenzene **(1)** and TsNa **(3)** in DMF. The samples were prepared in 4 mL quartz cuvettes, equipped with PTFE stoppers, and sealed with parafilm inside nitrogen filled glove-box. The intensity of the emission peak at 460 nm (λ_{ex}= 378 nm) expressed as the ratio I_0 /I, where I_0 is the emission intensity of photocatalyst at 460 nm in the absence of a quencher and I is the observed intensity, as a function of the quencher concentration was measured. Stern-Volmer plots for each component are given below.

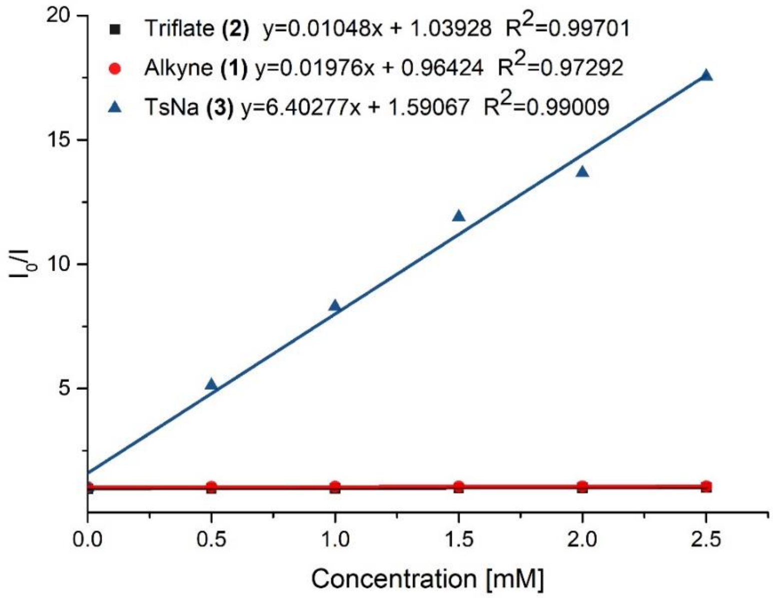

Supplementary Figure 3. Stern-Volmer fluorescence quenching studies with substrates. Stern-Volmer fluorescence quenching studies of Ru(dtbbpy)$_3$(PF$_6$)$_2$ (0.1 mM) with triflate (black line). Stern-Volmer fluorescence quenching studies of Ru(dtbbpy)$_3$(PF$_6$)$_2$ (0.1 mM) with alkyne (red line). Stern-Volmer fluorescence quenching studies of Ru(dtbbpy)$_3$(PF$_6$)$_2$ (0.1 mM) with TsNa (black line).

Stern-Volmer quenching experiments with products.

Stern-Volmer quenching experiments were carried by Edinburgh Fluorescence Spectrometer FS5, using a 0.01 mM solution of photocatalyst Ru(dtbbpy)$_3$(PF$_6$)$_2$ and variable concentrations (0.5, 1.0, 1.5, 2.0, 2.5 mM) of (***E***)-1-((2-(cyclohex-1-en-1-yl)-2-phenylvinyl)sulfonyl)-4-methylbenzene **(4a)** and (***Z***)-1-((2-(cyclohex-1-en-1-yl)-2-phenylvinyl)sulfonyl)-4-methylbenzene **(4b)** in DMF. The samples were prepared in 4 mL quartz cuvettes, equipped with PTFE stoppers, and sealed with parafilm inside nitrogen filled glove-box. The intensity of the emission peak at 460 nm (λ_{ex}= 378 nm) expressed as the ratio I_0 /I, where I_0 is the emission intensity of photocatalyst at 460 nm in the absence of a quencher and I is the observed intensity, as a function of the quencher concentration was measured. Stern-Volmer plots for each component are given in the Supplementary Figures below.

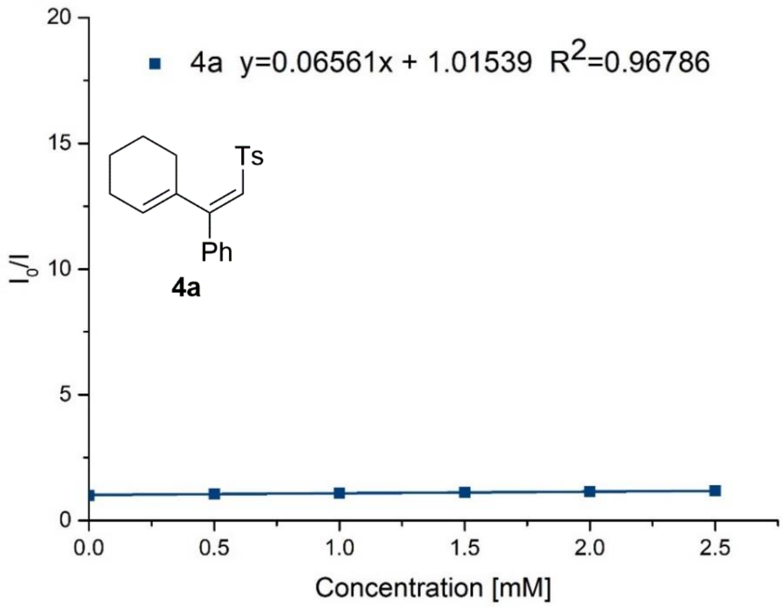

Supplementary Figure 4. Stern-Volmer fluorescence quenching studies with 4a. Stern-Volmer fluorescence quenching studies of Ru(dtbbpy)$_3$(PF$_6$)$_2$ (0.1 mM) with different concentrations of **4a** (blue line)**.**

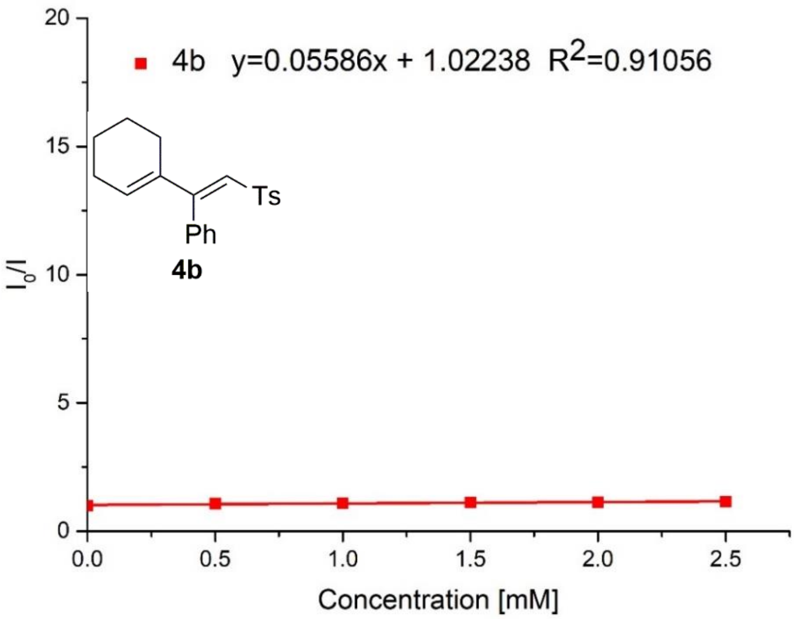

Supplementary Figure 5. Stern-Volmer fluorescence quenching studies with 4b. Stern-Volmer fluorescence quenching studies of Ru(dtbbpy)$_3$(PF$_6$)$_2$ (0.1 mM) with different concentrations of **4b** (red line)**.**

2.4.2 Light ON/OFF experiments.

Light ON/OFF experiments for *cis*-selectivity alkenylation.

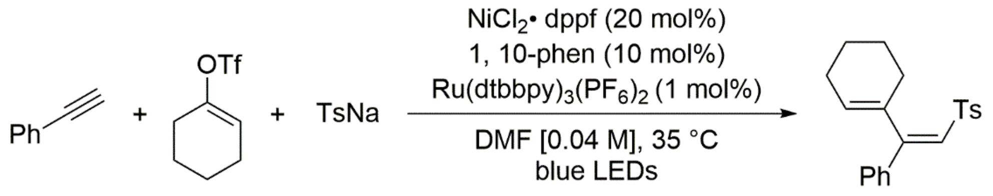

1 **2** **3** **4a**

According to general procedures A. To a flame-dried 8 mL reaction vial equipped with a magnetic stir bar was charged with $Ru(dtbbpy)_3(PF_6)_2$ (0.001 mmol, 1.2 mg, 1 mol %), $NiCl_2$• dppf (0.02 mmol, 13.6 mg, 20 mol %), 1,10-phen (0.01 mmol, 1.8 mg, 10 mol %), TsNa **(3)** (0.15 mmol, 1.5 equiv.) and DMF (2.5 mL, 0.04 M). The reaction mixture was degassed by nitrogen sparging for 30 min, followed by the addition of cyclohex-1-en-1-yl trifluoromethanesulfonate **(2)** (0.10 mmol, 1.0 equiv.) and ethynylbenzene **(1)** (0.15 mmol, 1.5 equiv.). The vial was sealed with Parafilm. Then the reaction mixture was irradiated with blue LEDs, The reaction mixtures were analyzed by GC with an internal standard. (Note: From the same reaction vial take 100 μL reaction mixture for analysis in every response time).

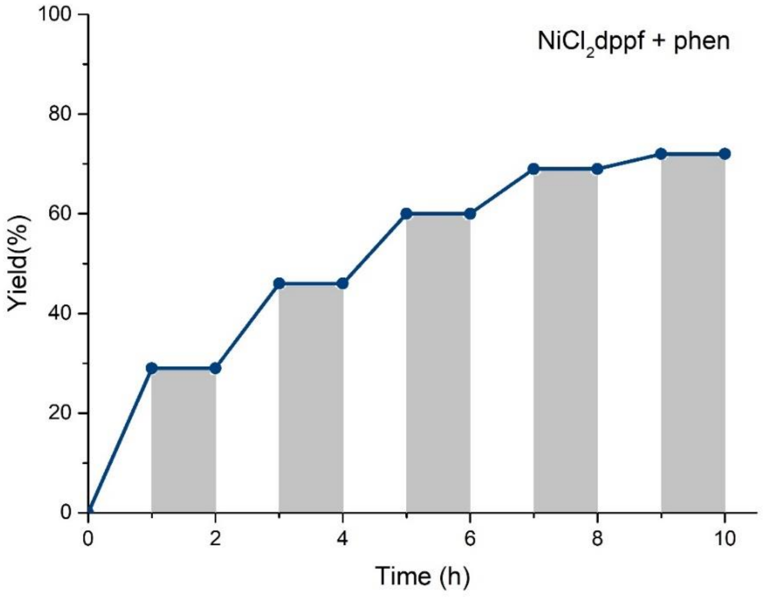

Supplementary Figure 6. Light on/off experiments for *cis*-selective alkenylation. Time course of the yield of **4a** for during which the light was periodically turned on and off. The white peaks represent light on experiments. The shaded peaks represent light off experiments. The blue line in the figure represents the percent product yield of **4a**.

Light ON/OFF experiments for *trans*-selectivity alkenylation.

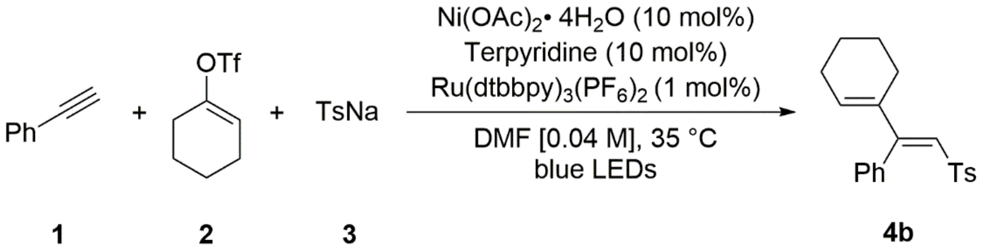

According to general procedures B. To a flame-dried 8 mL reaction vial equipped with a magnetic stir bar was charged with $Ru(dtbbpy)_3(PF_6)_2$ (0.001 mmol, 1.2 mg, 1 mol %), $Ni(OAc)_2$• $4H_2O$ (0.01 mmol, 2.5 mg, 10 mol %), Terpyridine (0.01 mmol, 2.3 mg, 10 mol %), TsNa **(3)** (0.15 mmol, 1.5 equiv.) and DMF (2.5 mL, 0.04 M). The reaction mixture was degassed by nitrogen sparging for 30 min, followed by the addition of cyclohex-1-en-1-yl trifluoromethanesulfonate **(2)** (0.10 mmol, 1.0 equiv.)

and ethynylbenzene **(1)** (0.15 mmol, 1.5 equiv.). The vial was sealed with Parafilm. Then the reaction mixture was irradiated with blue LEDs, The reaction mixtures were analyzed by GC with an internal standard. (Note: From the same reaction vial take 100 μL reaction mixture for analysis in every response time).

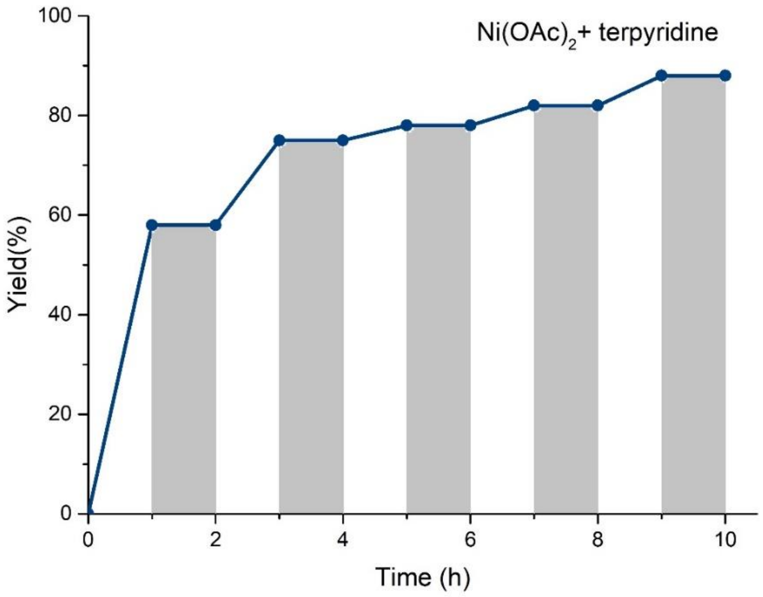

Supplementary Figure 7. Light on/off experiments for *trans*-selective alkenylation. Time course of the yield of **4b** for during which the light was periodically turned on and off. The white peaks represent light on experiments. The shaded peaks represent light off experiments. The blue line in the figure represents the percent product yield of **4b**.

2.4.3 Determination of fluorescence quantum yield.

We utilized protocol reported by Shunsuke and co-workers to determine the photon flux of blue LEDs. [10] All solutions were stored in the black vial and stored in the dark when not in use. Measurements were performed with the lights off to protect the samples from ambient light as much as possible.

Preparation of stock solutions.

A 0.15 M solution of ferrioxalate was obtained by dissolving potassium ferrioxalate

trihydrate([$K_3Fe^{III}(C_2O_4)_3$]•$3H_2O$; 1.11 g, 2.26 mmol) in 0.05 M H_2SO_4 (prepared by fresh deionized water) (15 mL total volume).

A buffered phenanthroline solution was obtained by dissolving 1,10-phenanthroline (10.0mg) and sodium acetate (2.25g) in 0.5M H_2SO_4 (prepared by fresh deionized water) (10 mL total volume).

Determination of background Fe^{2+} concentration.

3 mL of the ferrioxalate solution was added to a 8 mL vial. Next, 0.53 mL of the phenanthroline solution was added and the mixture was stored in the dark for 1 hour. Then the solution was transferred to a cuvette and a UV–vis spectrum was measured using UV–vis absorption spectrometer (lambda 950). The absorbance value at 510 nm was recorded. This process was repeated twice. Average value: 0.51377.

Determination of photon flux.

3 mL of the ferrioxalate solution was added to a 8 mL vial. The vial was immediately irradiated with blue LED (λ max= 469 nm) for 10 seconds and removed from the blue LED. Then, 0.53 mL of the phenanthroline solution was added to the ferrioxalate solution, and the resulting mixture was stored in the dark for 1 hour. Then the solution was transferred to a cuvette and the UV–vis spectrum was measured. The absorbance value at 510 nm was recorded. This process was repeated twice. Average value: 1.897.

Calculations.

The amount of Fe^{2+} formed was calculated according to the following equation:

$$mol\ Fe^{2+} = \frac{V \cdot \Delta A}{l \cdot \varepsilon} \qquad (1)$$

where V is the volume of the sample analyzed (3.53 mL), ΔA is the difference in average absorbances (between irradiated and unirradiated ferrioxalate solutions) at 510 nm, l is the path length, and ε is the molar absorptivity at 510 nm. [11]

$$mol\ Fe^{2+} = \frac{V \cdot \Delta A}{l \cdot \varepsilon} = \frac{(0.00353\ L)(1.38323)}{(1\ cm)(11100\ L\ /\ mol \cdot cm)} = 4.3989 \cdot 10^{-7} \qquad (2)$$

The fraction of light absorbed by the ferrioxalate actinometer was calculated by the

following equation:

$$f = 1 - 10^{-A} \qquad (3)$$

where A is the absorbance at 468 nm of the ferrioxalate actinometer solution prior to irradiation and addition of phenanthroline (Supplementary Figure 8).

$$f = 1 - 10^{-A} = 1 - 10^{-0.42545} = 0.6245518 \qquad (4)$$

The photon flux was calculated using the following equation:

$$photon\ flux = \frac{mol\ Fe^{2+}}{\Phi \cdot t \cdot f} \qquad (5)$$

Where Φ is the quantum yield for the ferrioxalate actinometer at 468 nm, t is the time and f is the fraction of light absorbed by the ferrioxalate actinometer solution.

$$photon\ flux = \frac{mol\ Fe^{2+}}{\Phi \cdot t \cdot f} = \frac{4.3989 \cdot 10^{-7}\text{mol}}{(0.92) \cdot (20\ s) \cdot (0.6245518)} \qquad (6)$$

$$= 3.8279 \cdot 10^{-8}\ einsterin\ /\ s$$

Determination of fraction of light for the ferrioxalate solution.

The absorbance at 468 nm of the ferrioxalate actinometer solution prior to irradiation and addition of phenanthroline was measured to be 0.42545.

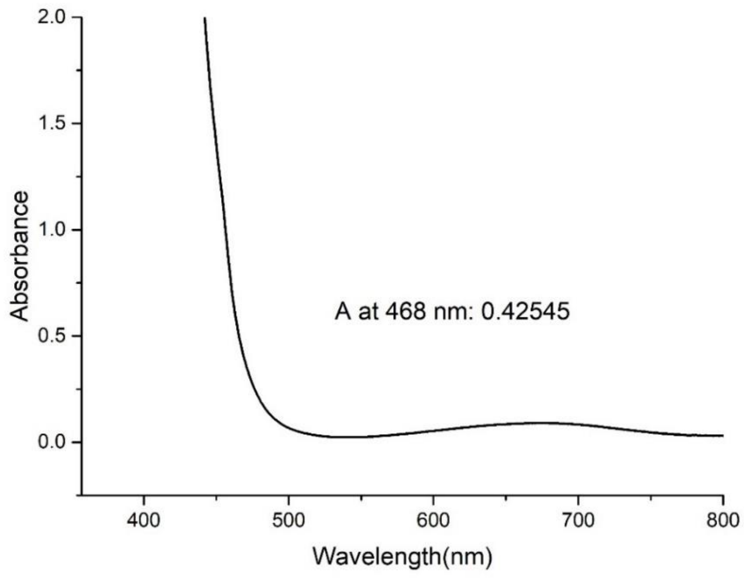

Supplementary Figure 8. UV-vis absorbance spectra of ferrioxalate solution. The black curve in the figure represents the UV-vis absorbance of ferrioxalate solution.

Absorbance of photocatalyst Ru(dtbbpy)$_3$(PF$_6$)$_2$.

The absorbance of Ru(dtbbpy)$_3$(PF$_6$)$_2$ in DMF was measured at the reaction concentration of 1 mM or a dilute concentration of 25 μM (Supplementary Figure 9). The absorbance at 468 nm for a 1 mM is 4.0371.

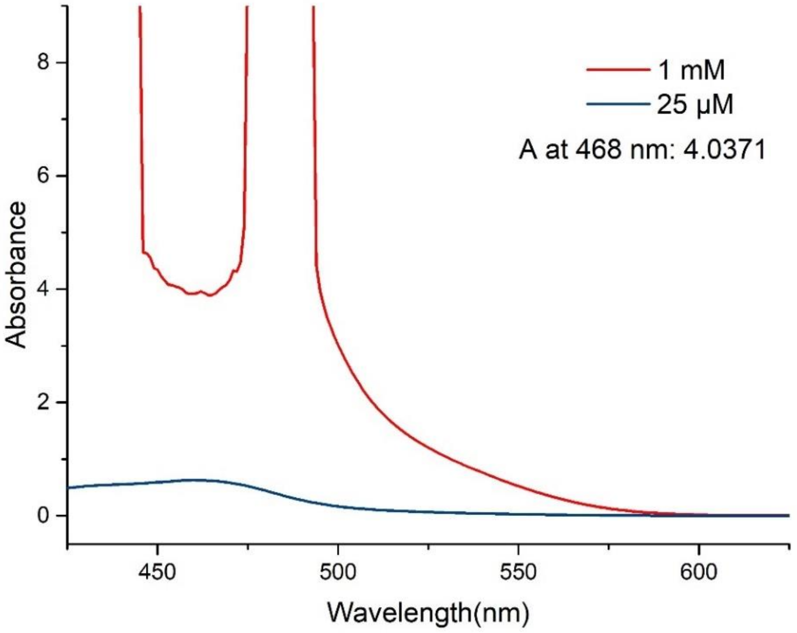

Supplementary Figure 9. UV-vis absorbance spectra of Ru(dtbbpy)$_3$(PF$_6$)$_2$. The red curve in the figure represents the UV-vis absorbance of 1.0 mM Ru(dtbbpy)$_3$(PF$_6$)$_2$ solution. The blue curve in the figure represents the UV-vis absorbance of 25.0 μM Ru(dtbbpy)$_3$(PF$_6$)$_2$ solution.

Determination of quantum yield.

For *cis*-selective alkenylation.

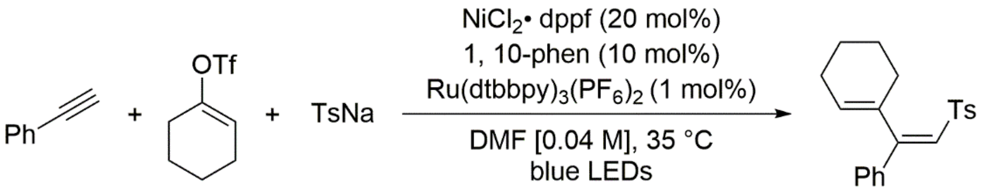

1 **2** **3** **4a**

According to the general procedure **A**.

To a flame-dried 8 mL reaction vial equipped with a magnetic stir bar was charged with $Ru(dtbbpy)_3(PF_6)_2$ (0.001 mmol, 1.2 mg, 1 mol %), $NiCl_2$• dppf (0.02 mmol, 13.6 mg, 20 mol %), 1,10-phen (0.01 mmol, 1.8 mg, 10 mol %), TsNa **(3)** (0.15 mmol, 1.5 equiv.) and DMF (2.5 mL, 0.04 M). The reaction mixture was degassed by nitrogen sparging for 30 min, followed by the addition of cyclohex-1-en-1-yl trifluoromethanesulfonate **(2)** (0.10 mmol, 1.0 equiv.) and ethynylbenzene **(1)** (0.15 mmol, 1.5 equiv.). The vial was sealed with Parafilm. Then the reaction mixture was irradiated with blue LEDs for 3600 s (around 35 °C). After irradiation, the reaction mixtures were analyzed by GC with an internal standard. Provide the desired product (29 % GC yield).

The quantum yield (Φ) was calculated using the following equation:

$$\Phi = \frac{mol\ \text{product}}{\text{photon}\, flux \cdot t \cdot f} \quad (7)$$

Where t is the reaction time and f is the fraction of light absorbed by photocatalyst that was calculated using the following equation:

$$f = 1 - 10^{-A} = 1 - 10^{-4.0371} = 0.9999 \quad (8)$$

Where A is the absorbance at 468 nm of the photocatalyst solution (1 mM in DMF) (Supplementary Figure 9).

$$\Phi = \frac{mol\ \text{product}}{\text{photon}\, flux \cdot t \cdot f} = \frac{0.0000029\, mol}{(3.8279 \cdot 10^{-8}\, einsterin\ /\ s) \cdot (3600s) \cdot (0.9999)} \quad (9)$$
$$= 0.2105$$

For *trans*-selective alkenylation.

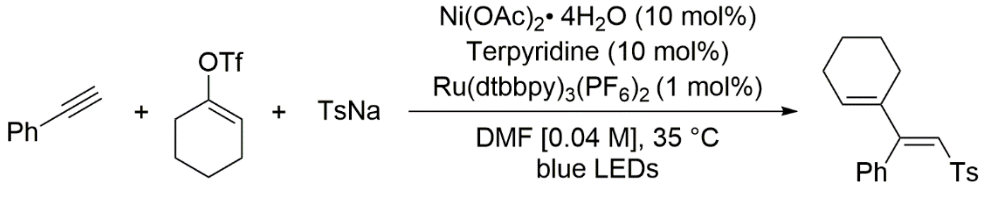

1 **2** **3** **4b**

According to the general procedures B.

To a flame-dried 8 mL reaction vial equipped with a magnetic stir bar was charged with $Ru(dtbbpy)_3(PF_6)_2$ (0.001 mmol, 1.2 mg, 1 mol %), $Ni(OAc)_2$• $4H_2O$ (0.01 mmol, 2.5 mg, 10 mol %), Terpyridine (0.01 mmol, 2.3 mg, 10 mol %), TsNa **(3)** (0.15 mmol, 1.5

equiv.) and DMF (2.5 mL, 0.04 M). The reaction mixture was degassed by nitrogen sparging for 30 min, followed by the addition of cyclohex-1-en-1-yl trifluoromethanesulfonate **(2)** (0.10 mmol, 1.0 equiv.) and ethynylbenzene **(1)** (0.15 mmol, 1.5 equiv.). The vial was sealed with Parafilm. Then the reaction mixture was irradiated with blue LEDs for 3600 s (around 35 °C). After irradiation, the reaction mixtures were analyzed by GC with an internal standard. Provide the desired product (58 % GC yield).

The quantum yield (Φ) was calculated using the following equation:

$$\Phi = \frac{mol\ \text{product}}{\text{photon}\, flux \cdot t \cdot f} \qquad (10)$$

Where t is the reaction time and f is the fraction of light absorbed by photocatalyst that was calculated using the following equation:

$$f = 1 - 10^{-A} = 1 - 10^{-4.0371} = 0.9999 \qquad (11)$$

Where A is the absorbance at 468 nm of the photocatalyst solution (1 mM in DMF) (Supplementary Figure 9).

$$\Phi = \frac{mol\ \text{product}}{\text{photon}\, flux \cdot t \cdot f} = \frac{0.0000058\, mol}{(3.8279 \cdot 10^{-8}\ einsterin\ /\ s) \cdot (3600s) \cdot (0.9999)} = 0.4209 \qquad (12)$$

2.4.4 Time-course studies.

Time-course studies for *cis*-selectivity alkenylation.

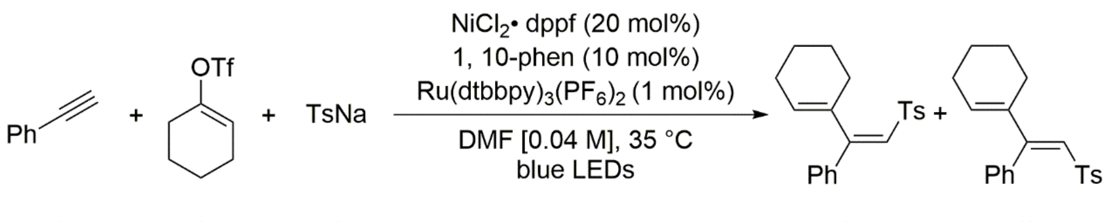

1 **2** **3** **4a** **4b**

According to General procedures A, to a flame-dried 8 mL reaction vial equipped with a magnetic stir bar was charged with $Ru(dtbbpy)_3(PF_6)_2$ (0.001 mmol, 1.2 mg, 1 mol %), $NiCl_2$• dppf (0.02 mmol, 13.6 mg, 20 mol %), 1,10-phen (0.01 mmol, 1.8 mg, 10 mol %), TsNa **(3)** (0.15 mmol, 1.5 equiv.) and DMF (2.5 mL, 0.04 M). The reaction mixture was degassed by nitrogen sparging for 30 min, followed by the addition of cyclohex-1-en-1-yl trifluoromethanesulfonate **(2)** (0.10 mmol, 1.0 equiv.) and ethynylbenzene **(1)**

(0.15 mmol, 1.5 equiv.). The vial was sealed with Parafilm. Then the reaction mixture was irradiated with blue LEDs, 100 μL of the reaction mixture was taken out at 10, 20, 30, 40, 50, 60, 120, 180, 240, 300 minutes, and immediately quenched with water and ethyl acetate, and the percent product yield was monitored by GC.

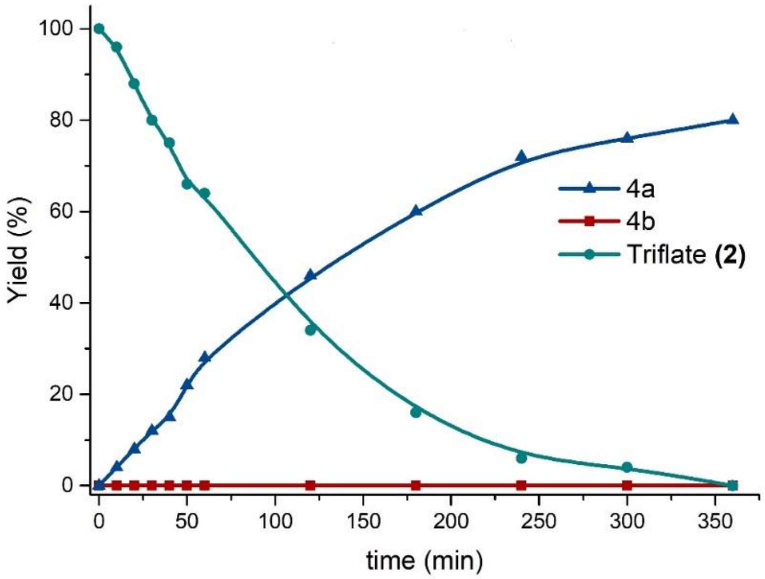

Supplementary Figure 10. Time-course studies of *cis*-selectivity alkenylation. The curves in the figure represent the composition of the reaction mixture at different time. The blue line represent the generation of **4a**, the red part represent the generation of **4b**, The green part represents the remainder of triflate **(2)**.

Time-course studies for *trans*-selectivity alkenylation.

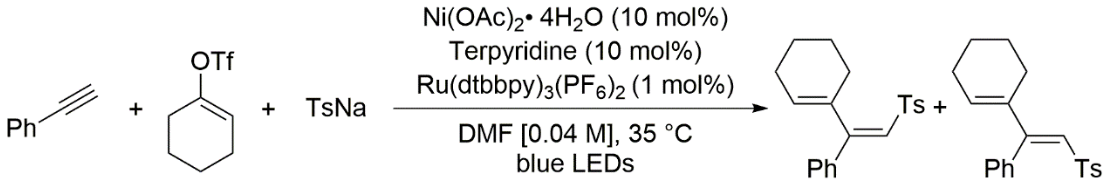

1 **2** **3** **4a** **4b**

According to general procedures B, To a flame-dried 8 mL reaction vial equipped with a magnetic stir bar was charged with $Ru(dtbbpy)_3(PF_6)_2$ (0.001 mmol, 1.2 mg, 1 mol %), $Ni(OAc)_2$• $4H_2O$ (0.01 mmol, 2.5 mg, 10 mol %), Terpyridine (0.01 mmol, 2.3 mg, 10 mol %), TsNa **(3)** (0.15 mmol, 1.5 equiv.) and DMF (2.5 mL, 0.04 M). The reaction

mixture was degassed by nitrogen sparging for 30 min, followed by the addition of cyclohex-1-en-1-yl trifluoromethanesulfonate **(2)** (0.10 mmol, 1.0 equiv.) and ethynylbenzene **(1)** (0.15 mmol, 1.5 equiv.). The vial was sealed with Parafilm. Then the reaction mixture was irradiated with blue LEDs, 100 μL of the reaction mixture was taken out at 10, 20, 30, 40, 50, 60, 120, 180, 240, 300 minutes, and immediately quenched with water and ethyl acetate, and the percent product yield was monitored by GC.

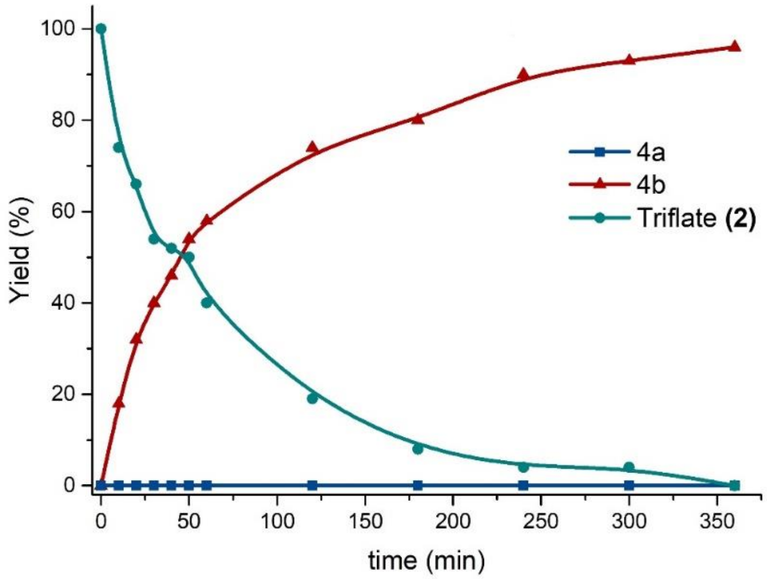

Supplementary Figure 11. Time-course studies for *trans*-selectivity alkenylation. The curves in the figure represent the composition of the reaction mixture at different time. The blue line represent the generation of **4a**, the red part represent the generation of **4b**, The green part represents the remainder of triflate **(2)**.

2.4.5 Identification of side products.

Supplementary Table 9. Identification of side products.

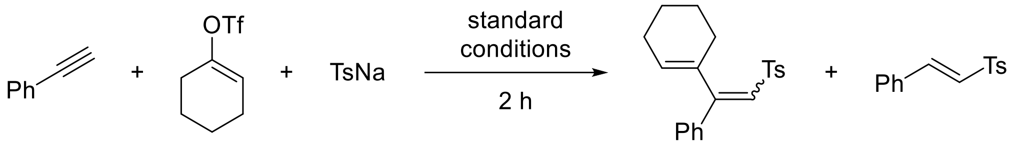

4a or 4b **67**

Entry	Conditions	Yield of 4a (%)	Yield of 4b (%)	Yield of 67(%)
1	*cis*-condition	35	0	9
2	*trans*-condition	0	71	11

To a flame-dried 8 mL reaction vial equipped with a magnetic stir bar was charged with $Ru(dtbbpy)_3(PF_6)_2$ (0.001 mmol, 1 mol %), nickel catalyst, ligand, sulfinate (0.15 mmol, 1.5 equiv.) and DMF (2.5 mL, 0.04 M). The reaction mixture was degassed by nitrogen sparging for 30 min, followed by the addition of vinyl triflate (0.10 mmol, 1.0 equiv.) and alkyne (0.15 mmol, 1.5 equiv.). The vial was sealed with Parafilm. Then the reaction mixture was irradiated with blue LEDs for 2 h (around 35 °C, with a cooling fan placed on the top of the vial). The reaction mixture was quenched with water and extracted with ethyl acetate three times. The combined organic layers were dried with $MgSO_4$, filtered and concentrated in vacuo. The crude material was purified by flash chromatography (silica gel, petroleum ether/ ethyl acetate) to afford the products **4a/ 4b** and **67**.

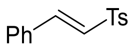

(*E*)-1-methyl-4-(styrylsulfonyl)benzene (67).

^{1}H NMR (400 MHz, $CDCl_3$) δ 7.83 (d, *J* = 8.2 Hz, 2H), 7.66 (d, *J* = 15.4 Hz, 1H), 7.50 – 7.45 (m, 2H), 7.37 (dd, *J* = 18.7, 7.4 Hz, 5H), 6.85 (d, *J* = 15.4 Hz, 1H), 2.44 (s, 3H).

^{13}C NMR (100 MHz, $CDCl_3$) δ 144.42, 141.96, 137.77, 132.48, 131.13, 129.99, 129.09, 128.55, 127.74, 127.65, 21.64.

HRMS (ESI) Calcd for $C_{15}H_{15}O_2S^+$ $[M+H]^+$: 259.0787, found: 259.0789.

2.4.6 Radical inhibition and trapping reactions.

Supplementary Table 10. Radical inhibition reactions.

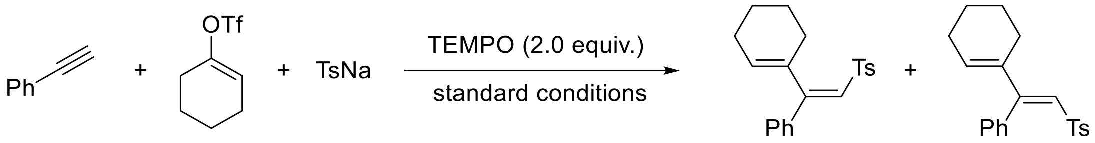

4a **4b**

**Entry**	**Conditions**	**Yield of 4a** (%)	**Yield of 4b** (%)
1	*cis*-condition	0	0
2	*trans*-condition	0	0

To a flame-dried 8 mL reaction vial equipped with a magnetic stir bar was charged with Ru(dtbbpy)$_3$(PF$_6$)$_2$ (0.001 mmol, 1 mol %), nickel catalyst, ligand, sulfinate (0.15 mmol, 1.5 equiv.), TEMPO (0.20 mmol, 2.0 equiv.) and DMF (2.5 mL, 0.04 M). The reaction mixture was degassed by nitrogen sparging for 30 min, followed by the addition of vinyl triflate (0.10 mmol, 1.0 equiv.) and alkyne (0.15 mmol, 1.5 equiv.). The vial was sealed with Parafilm. Then the reaction mixture was irradiated with blue LEDs for 6 h (around 35 °C, with a cooling fan placed on the top of the vial). The product **4a**/ **4b** not detected by GC-MS.

Supplementary Table 11. Radical trapping reactions with 1,1- diphenylethylene.

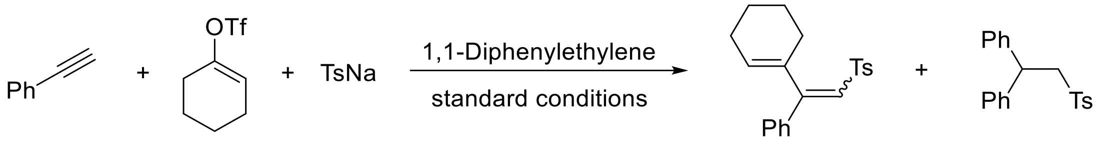

Entry	Conditions	Yield of 4a (%)	Yield of 4b (%)	Yield of 68(%)
1	*cis*-condition	75	0	6
2	*trans*-condition	0	79	7

To a flame-dried 8 mL reaction vial equipped with a magnetic stir bar was charged with $Ru(dtbbpy)_3(PF_6)_2$ (0.001 mmol, 1 mol %), nickel catalyst, ligand, sulfinate (0.15 mmol, 1.5 equiv.) and DMF (2.5 mL, 0.04 M). The reaction mixture was degassed by nitrogen sparging for 30 min, followed by the addition of vinyl triflate (0.10 mmol, 1.0 equiv.) 1,1-diphenylethylene (0.20 mmol, 2.0 equiv.) and alkyne (0.15 mmol, 1.5 equiv.). The vial was sealed with Parafilm. Then the reaction mixture was irradiated with blue LEDs for 6 h (around 35 °C, with a cooling fan placed on the top of the vial). The reaction mixture was quenched with water and extracted with ethyl acetate three times. The combined organic layers were dried with $MgSO_4$, filtered and concentrated in vacuo. The crude material was purified by flash chromatography (silica gel, petroleum ether/ ethyl acetate) to afford the products **4a/ 4b** and **68**.

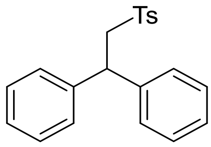

(2-tosylethane-1, 1-diyl) dibenzene (68).

^{1}H NMR (400 MHz, $CDCl_3$) δ 7.45 (d, J = 8.3 Hz, 2H), 7.14 – 7.08 (m, 4H), 7.06 (dd, J = 9.5, 4.6 Hz, 8H), 4.53 (s, 1H), 3.82 (d, J = 7.1 Hz, 2H), 2.29 (s, 3H).

^{13}C NMR (101 MHz, $CDCl_3$) δ 144.23, 141.50, 136.60, 129.54, 128.72, 128.02, 127.62, 126.86, 61.56, 46.20, 21.56.

HRMS (ESI) Calcd for $C_{21}H_{21}O_2S^+$ $[M+H]^+$: 337.1257, found: 337.1255.

Supplementary Table 12. Radical trapping reactions with 1,5-diene.

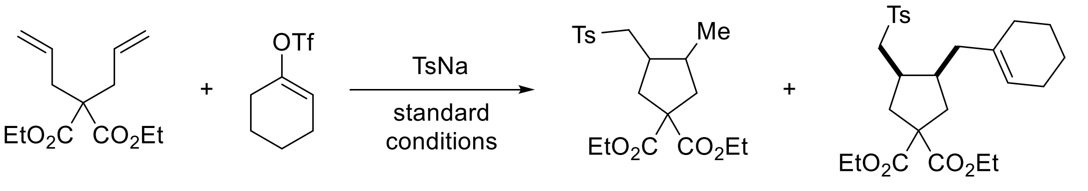

Entry	Conditions	Yield of 70 (%)	Yield of 70’(%)
1	*cis*-condition	7	0
2	*trans*-condition	9	0

To a flame-dried 8 mL reaction vial equipped with a magnetic stir bar was charged with Ru(dtbbpy)$_3$(PF$_6$)$_2$ (0.001 mmol, 1 mol %), nickel catalyst, ligand, sulfinate (0.15 mmol, 1.5 equiv.) and DMF (2.5 mL, 0.04 M). The reaction mixture was degassed by nitrogen sparging for 30 min, followed by the addition of vinyl triflate (0.10 mmol, 1.0 equiv.) and 1,5-diene (0.20 mmol, 2.0 equiv.). The vial was sealed with Parafilm. Then the reaction mixture was irradiated with blue LEDs for 6 h (around 35 °C, with a cooling fan placed on the top of the vial). The reaction mixture was quenched with water and extracted with ethyl acetate three times. The combined organic layers were dried with $MgSO_4$, filtered and concentrated in vacuo. The crude material was purified by flash chromatography (silica gel, petroleum ether/ ethyl acetate) to afford the products **70** and **70’**.

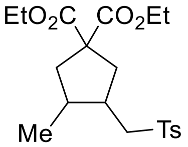

Diethyl 3-methyl-4-(tosylmethyl)cyclopentane-1,1-dicarboxylate (70).

^{1}H NMR (400 MHz, $CDCl_3$) δ 7.72 (d, J = 7.7 Hz, 2H), 7.29 (d, J = 8.1 Hz, 2H), 4.12 – 4.06 (m, 4H), 3.07 (dd, J = 14.1, 4.8 Hz, 1H), 2.97 (dd, J = 14.1, 8.1 Hz, 1H), 2.42 – 2.32 (m, 6H), 2.25 – 2.17 (m, 1H), 2.11 (td, J = 12.1, 4.6 Hz, 1H), 1.89 (dd, J = 13.8, 4.9 Hz, 1H), 1.16 (td, J = 7.1, 0.7 Hz, 6H), 0.76 (d, J = 7.0 Hz, 3H).

^{13}C NMR (101 MHz, $CDCl_3$) δ 172.45, 172.31, 144.71, 136.68, 129.95, 128.00, 61.59,

61.56, 58.59, 56.86, 40.98, 37.80, 37.11, 36.27, 21.64, 14.96, 14.01.

HRMS (ESI) Calcd for $C_{20}H_{29}O_6S^+$ [M+H]$^+$: 397.1679, found: 397.1682.

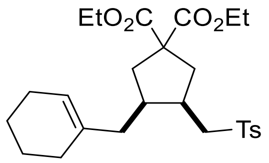

Diethyl (3*S*,4*R*)-3-(cyclohex-1-en-1-ylmethyl)-4-(tosylmethyl)cyclopentane-1,1-dicarboxylate (70').

^{1}H NMR (400 MHz, $CDCl_3$) δ 7.69 (d, *J* = 8.2 Hz, 2H), 7.28 (d, *J* = 8.2 Hz, 2H), 5.22 (s, 1H), 4.09 (m, 4H), 3.22 (m, 0.11H), 3.12 (m, 0.88H), 3.02 – 2.91 (m, 1H), 2.49 – 2.32 (m, 5H), 2.31 (m, 1H), 2.27 – 2.13 (m, 2H), 1.91 (m, 1H), 1.83 (s, 2H), 1.70 (m, 4H), 1.51 – 1.34 (m, 4H), 1.15 (m, 6H).

^{13}C NMR (101 MHz, $CDCl_3$) δ 172.45, 172.30, 144.61, 136.65, 135.01, 129.88, 127.97, 123.22, 61.49, 58.35, 56.01, 39.42, 38.05, 37.78, 37.34, 36.37, 27.95, 25.18, 22.77, 22.32, 21.55, 13.97.

HRMS (ESI) Calcd for $C_{26}H_{36}NaO_6S^+$ [M+Na]$^+$: 499.2125, found: 499.2126.

Supplementary Table 13. Reactions with Ni(COD)$_2$/dppf.

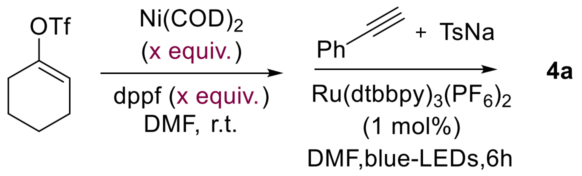

Entry	Conditions	Yield of 4a (%)	Yield of 4b (%)
1	x = 1.0	82	0
2	x = 0.5	71	0

In a glovebox, nickel catalyst, ligand, vinyl triflate (0.10 mmol, 1.0 equiv.) and DMF (2.5 mL) were added to a 8 mL vial with a Teflon-sealed screwcap. The mixture was stirred for 2 h, followed by the addition of $Ru(dtbbpy)_3(PF_6)_2$ (0.001 mmol, 1 mol %), sulfinate (0.15 mmol, 1.5 equiv.) and alkyne (0.15 mmol, 1.5 equiv.), The vial was sealed with Parafilm. Then the reaction mixture was irradiated with blue LEDs for

6 h (around 35 °C, with a cooling fan placed on the top of the vial). The reaction mixture was quenched with water and extracted with ethyl acetate three times. The combined organic layers were dried with $MgSO_4$, filtered and concentrated in vacuo. The crude material was purified by flash chromatography (silica gel, petroleum ether/ ethyl acetate) to afford the products.

2.4.7 Control reactions with TsCl and boronic acid without light

Supplementary Table 14. Control reaction.

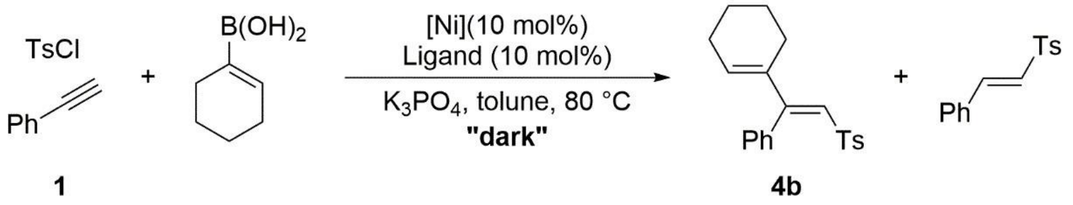

Entry	[Ni]/ ligand	Yield of 4b (%)	Yield of 67 (%)
1[a]	$NiCl_2$• dppf/ phen	11	0
2	$Ni(OAc)_2$• $4H_2O$/ tpy	0	0
3	$NiCl_2$• py_4/ tpy	9	0

[a]Reaction condition: $NiCl_2$• dppf (20 mol%), 1,10-phenanthroline (10 mol%).

Experimental Procedure: nickel catalyst (0.015 mmol, 10 mol%), ligand(0.015 mmol, 10 mol%), cyclohex-1-en-1-ylboronic acid (37.5 mg, 0.30 mmol, 2.0 equiv), phenylsulfonyl chloride (57.0 mg, 0.30 mmol, 2.0 equiv.) and K_3PO_4 (84.9 mg, 0.40 mmol, 2.7 equiv.) were placed in an oven-dried Schlenk-tube. The reaction vessel was evacuated and filled with N_2 three times. Toluene (4.0 mL) and ethynylbenzene **(1)** (15.3 mg, 0.15 mmol, 1.0 equiv) were sequentially added at 25 °C. The reaction mixture was placed in a preheated oil bath at 80 °C and stirred at 1400 rpm for 16 h. The resulting mixture was cooled to 25 °C, filtered through a short pad of Celite and washed with EtOAc (30 mL). After removal of the solvent under reduced pressure, The crude material was purified by flash chromatography (silica gel, petroleum ether/ ethyl acetate = 5: 1) to afford the products (*Z*)-1-((2-(cyclohex-1-en-1-yl)-2-phenylvinyl)sulfonyl)-

4-methylbenzene **(4b)**.

2.4.8 NMR titration experiments:

Solutions containing equal molar concentrations of the $NiCl_2$• dppf (0.01 M inDMSO-*d*6) and 1,10-phenanthroline (0.1 M in DMSO-*d*6) were prepared and mixed to cover Nickel /Ligand ratio from 1: 2, 1: 1, to 2: 1, a coaxial insert containing a solution of H_3PO_4 in DMSO-*d*6 was used for locking and calibration.

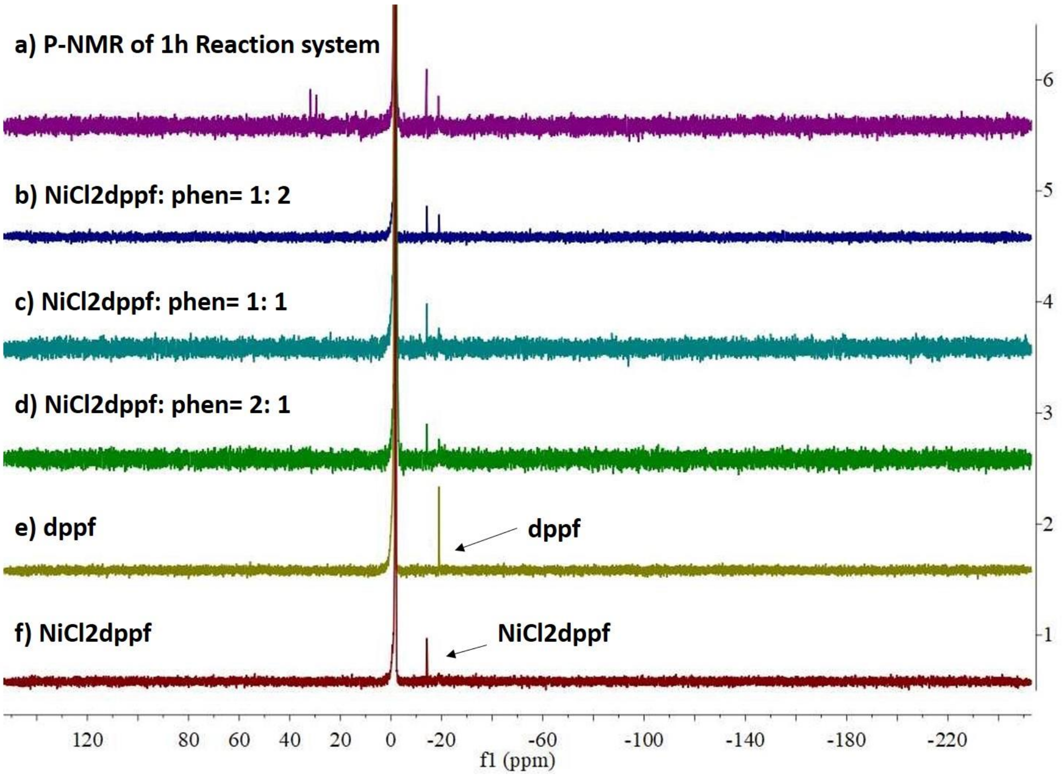

Supplementary Figure 12. NMR titration. No significant new signals are observed in the P-NMR of 1h reaction system. a) P-NMR of 1h reaction system. b) P-NMR of mixture with $NiCl_2$• dppf and phen molar ratio of 1: 2. c) P-NMR of mixture with $NiCl_2$• dppf and phen molar ratio of 1: 1. d) P-NMR of mixture with $NiCl_2$• dppf and phen molar ratio of 2: 1. e) P-NMR of dppf. f) P-NMR of mixture with $NiCl_2$• dppf.

Supplementary Notes

3.1 Crystal structure and X-ray refinement data

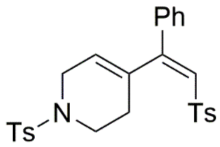

(CCDC 2117869)

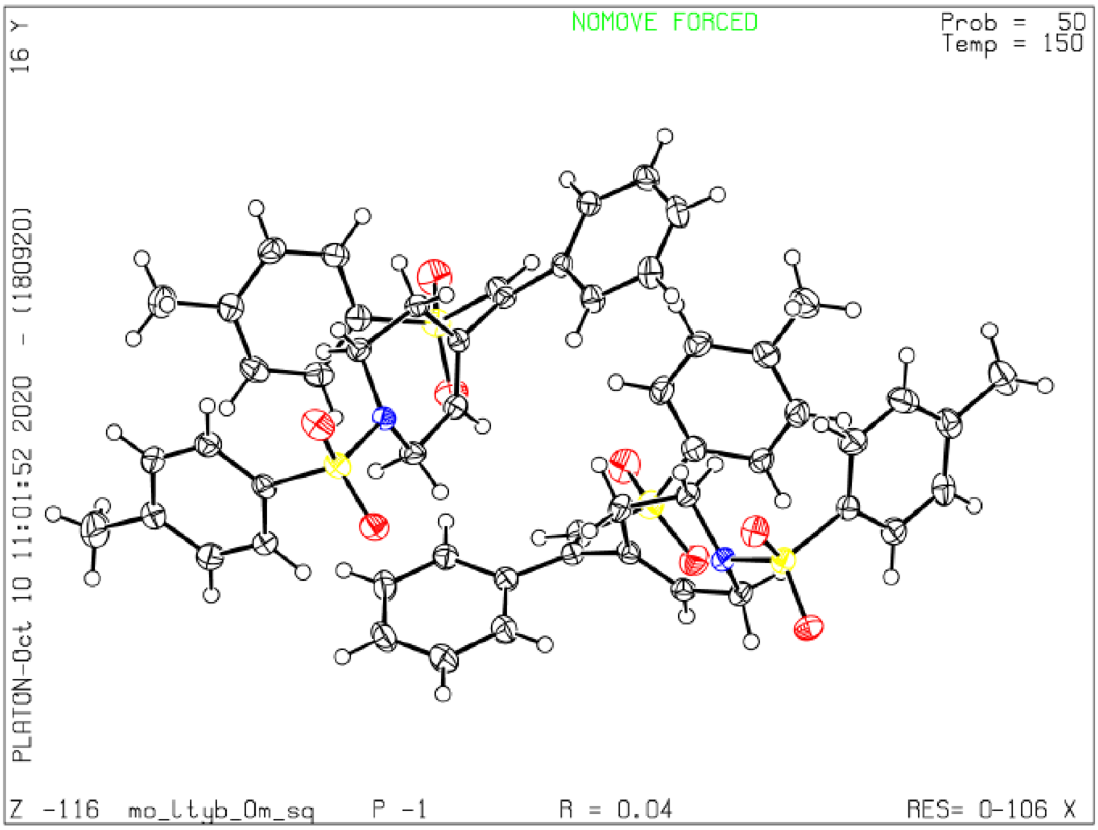

Supplementary Figure 13. X-ray structure of compound 13a. Single crystals for X-ray studies were grown by slow evaporation of a solution of compound **13a** in a mixture of THF/ n-heptane in NMR tube at room temperature. The X-ray data of **13a** is deposited in the Cambridge Crystallographic Data Centre with a number of CCDC: 2117869.

Supplementary Table 15 Crystal data and structure refinement for 13a.

Identification code	**13a**
Empirical formula	$C_{27}H_{27}NO_4S_2$
Formula weight	493.61
Temperature/K	150.0
Crystal system	triclinic
Space group	P-1
a/Å	10.5898(5)
b/Å	14.9050(7)
c/Å	17.2403(8)
α/°	67.528(2)
β/°	88.548(2)
γ/°	82.184(2)
Volume/Å^3	2490.2(2)
Z	4
ρ_{calc}g/cm^3	1.317
μ/mm^{-1}	0.248
F(000)	1040
Crystal size/mm^3	0.2 × 0.15 × 0.10
Radiation	MoKα (λ = 0.71073)
2Θ range for data collection/°	3.884 to 52.772
Index ranges	-13 ≤ h ≤ 13, -18 ≤ k ≤ 18, -21 ≤ l ≤ 21
Reflections collected	59861
Independent reflections	10201 [R_{int} = 0.0657, R_{sigma} = 0.0410]
Data/restraints/parameters	10201/0/613
Goodness-of-fit on F^2	1.017
Final R indexes [I>=2σ (I)]	R_1 = 0.0402, wR_2 = 0.0935
Final R indexes [all data]	R_1 = 0.0622, wR_2 = 0.1054
Largest diff. peak/hole / e Å^{-3}	0.41/-0.37

Supplementary Table 16. Fractional Atomic Coordinates (×10^4) and Equivalent Isotropic Displacement Parameters (Å^2×10^3) for 13a. U_{eq} is defined as 1/3 of of the trace of the orthogonalised U_{IJ} tensor.

Atom	*x*	*y*	*z*	U(eq)
S001	10208.3(11)	4694.6(7)	3873.7(7)	23.6(3)
S002	4800.3(10)	4736.1(8)	8022.9(7)	23.2(3)
S003	5975.9(11)	29.0(8)	8046.4(7)	26.6(3)
S004	10952.2(11)	-76.9(8)	6764.6(7)	26.7(3)
O005	5685(3)	5432(2)	7709(2)	30.0(7)
O006	3462(3)	5056(2)	7871(2)	31.4(7)
O007	11209(3)	5296(2)	3734(2)	32.7(8)
O008	8903(3)	5111(2)	3846(2)	30.7(7)
O009	9614(3)	49(3)	6534(2)	35.8(8)
O00A	11574(4)	-1059(2)	7205(2)	39.8(9)
O00B	6695(4)	-917(2)	8197(2)	37.9(8)
O00C	4640(3)	64(3)	8215(2)	35.9(8)
N00D	10479(3)	3823(3)	4805(2)	21.1(7)
N00E	5159(3)	3918(3)	7601(2)	21.3(7)
C00F	10917(4)	2245(3)	6351(3)	18.6(8)
C00G	9801(4)	2408(3)	5946(3)	21.2(8)
C00H	5794(4)	2409(3)	7012(3)	21.2(8)
C00I	11804(4)	3319(3)	5007(3)	22.8(9)
C00J	4679(4)	2467(3)	7378(3)	23.3(9)
C00K	10345(4)	4126(3)	3146(3)	21.8(8)
C00L	11990(4)	2823(3)	5961(3)	22.0(8)
C00M	11132(4)	1503(3)	7228(3)	21.2(8)
C00N	6695(4)	546(3)	8659(3)	25.5(9)
C00O	4271(4)	3187(3)	7782(3)	24.8(9)
C00P	6295(4)	2125(3)	5669(3)	23.5(9)
C00Q	9493(4)	3172(3)	5079(3)	26.0(9)
C00R	6106(4)	1700(3)	6591(3)	22.6(9)
C00S	6194(4)	723(3)	6981(3)	28.0(9)
C00T	11293(4)	1870(3)	7901(3)	21.6(8)
C00U	7104(4)	1615(3)	5270(3)	25.6(9)
C00V	11141(4)	540(3)	7431(3)	25.8(9)
C00W	12046(4)	1305(4)	8623(3)	26.8(9)
C00X	11530(4)	3954(4)	2814(3)	29.7(10)
C00Y	9275(4)	3800(4)	2941(3)	28.6(10)

Supplementary Table 16. Fractional Atomic Coordinates (×10⁴) and Equivalent Isotropic Displacement Parameters (Å²×10³) for 13a. U_{eq} is defined as 1/3 of of the trace of the orthogonalised U_{IJ} tensor (continuation).

C00Z	5073(4)	4129(3)	9111(3)	23.7(9)
C010	5970(4)	821(3)	9238(3)	26.7(9)
C011	6521(4)	3491(3)	7663(3)	23.7(9)
C012	11900(5)	1121(3)	4347(3)	28.6(10)
C013	11195(4)	781(3)	5069(3)	27.9(10)
C014	10616(5)	2767(3)	7832(3)	28.2(10)
C015	13109(4)	479(3)	5925(3)	28.5(10)
C016	13206(4)	1166(3)	4399(3)	27.2(9)
C017	6768(4)	3071(3)	6984(3)	27.9(10)
C018	6207(5)	4161(4)	9482(3)	33.4(11)
C019	11808(4)	454(3)	5847(3)	24.8(9)
C01A	8006(4)	589(4)	8606(3)	30.0(10)
C01B	5504(5)	3094(3)	10835(3)	29.9(10)
C01C	5590(5)	3019(4)	5180(3)	32.1(10)
C01D	7174(5)	1991(4)	4404(3)	30.5(10)
C01E	6570(4)	1138(4)	9783(3)	30.5(10)
C01F	13799(4)	842(3)	5197(3)	29.0(10)
C01G	7879(4)	1193(3)	9734(3)	27.8(10)
C01H	8584(4)	921(4)	9143(3)	30.6(10)
C01I	4379(5)	3079(4)	10450(3)	33.4(11)
C01J	4150(5)	3590(4)	9597(3)	30.7(10)
C01K	11375(5)	2510(4)	9223(3)	34.3(11)
C01L	12073(5)	1623(4)	9280(3)	30.8(10)
C01M	11619(5)	3444(4)	2277(3)	32.0(10)
C01N	9396(5)	3277(4)	2427(3)	36.2(11)
C01O	13956(5)	1576(4)	3620(3)	36.7(11)
C01P	10653(5)	3087(4)	8498(3)	33.8(11)
C01Q	10566(5)	3082(4)	2089(3)	33.6(11)
C01R	6456(5)	2875(4)	3922(3)	35.1(11)
C01S	8534(5)	1552(4)	10309(3)	37.5(11)
C01T	5738(6)	2527(4)	11766(3)	44.1(13)
C01U	6408(5)	3648(4)	10347(3)	40.4(12)
C01V	5656(5)	3398(4)	4310(3)	39.6(12)
C01W	10687(6)	2459(6)	1561(4)	55.0(17)

Supplementary Table 17. Anisotropic Displacement Parameters ($Å^2×10^3$) for 13a. The Anisotropic displacement factor exponent takes the form: -$2\pi^2[h^2a^{*2}U_{11}+2hka^*b^*U_{12}+…]$.

Atom	U_{11}	U_{22}	U_{33}	U_{23}	U_{13}	U_{12}
S001	29.7(6)	16.3(5)	22.1(5)	-4.1(4)	-2.1(4)	-2.9(4)
S002	23.6(5)	19.9(5)	23.1(5)	-5.7(4)	0.9(4)	-0.5(4)
S003	28.1(6)	22.4(5)	25.5(6)	-4.3(4)	5.0(4)	-6.5(4)
S004	30.0(6)	19.1(5)	30.3(6)	-8.2(4)	-0.7(5)	-4.4(4)
O005	35.1(18)	22.7(16)	31.0(17)	-7.6(13)	7.7(14)	-8.8(13)
O006	24.2(16)	32.7(18)	34.0(18)	-11.7(15)	-3.0(13)	5.2(13)
O007	43(2)	22.6(16)	29.7(18)	-3.5(14)	-1.3(15)	-14.0(14)
O008	36.3(18)	21.9(16)	30.1(17)	-8.8(13)	-5.6(14)	6.8(13)
O009	30.7(18)	36.0(19)	43(2)	-15.4(16)	1.7(15)	-13.3(15)
O00A	51(2)	20.0(17)	46(2)	-10.0(15)	1.9(17)	-3.4(15)
O00B	46(2)	23.1(17)	39(2)	-6.4(15)	5.8(16)	-3.5(15)
O00C	28.8(18)	40(2)	38(2)	-12.1(16)	7.5(15)	-12.4(15)
N00D	21.7(17)	18.0(17)	20.8(18)	-4.9(14)	0.5(14)	-1.3(14)
N00E	17.3(17)	22.2(18)	23.4(18)	-7.8(14)	1.0(14)	-2.5(14)
C00F	22(2)	16.4(18)	17.7(19)	-7.3(16)	1.9(15)	-1.3(15)
C00G	23(2)	20(2)	21(2)	-7.6(17)	0.6(16)	-3.8(16)
C00H	21(2)	23(2)	18.1(19)	-5.3(16)	1.5(16)	-6.7(16)
C00I	21(2)	25(2)	20(2)	-5.8(17)	1.4(16)	-1.5(16)
C00J	20(2)	28(2)	21(2)	-8.3(17)	0.5(16)	-4.1(17)
C00K	24(2)	21(2)	17.2(19)	-3.1(16)	0.2(16)	-2.0(16)
C00L	22(2)	22(2)	20(2)	-4.4(16)	1.4(16)	-3.4(16)
C00M	18.6(19)	23(2)	20(2)	-5.9(17)	1.5(15)	-2.9(16)
C00N	26(2)	25(2)	20(2)	-1.6(17)	4.0(17)	-4.6(17)
C00O	19(2)	29(2)	28(2)	-12.8(19)	7.2(17)	-7.8(17)
C00P	22(2)	27(2)	21(2)	-8.1(18)	2.2(16)	-6.5(17)
C00Q	22(2)	28(2)	22(2)	-1.3(18)	-1.4(17)	-8.0(17)
C00R	21(2)	26(2)	20(2)	-8.5(17)	1.2(16)	-4.4(16)
C00S	32(2)	26(2)	26(2)	-9.1(18)	2.9(18)	-6.3(18)
C00T	21(2)	24(2)	18(2)	-5.3(16)	3.5(16)	-5.4(16)
C00U	26(2)	30(2)	26(2)	-15.1(19)	5.1(17)	-10.2(18)
C00V	26(2)	24(2)	27(2)	-9.1(18)	1.1(18)	-3.0(17)
C00W	25(2)	34(2)	17(2)	-4.2(18)	2.3(17)	-8.4(18)
C00X	27(2)	35(3)	25(2)	-9(2)	2.3(18)	-5.2(19)
C00Y	23(2)	39(3)	25(2)	-14(2)	-3.6(17)	-2.2(19)

Supplementary Table 17. Anisotropic Displacement Parameters (Å^2×10^3) for 13a. The Anisotropic displacement factor exponent takes the form: -2π^2[h^{2}a*^{2}U$_{11}$+2hka*b*U$_{12}$+…] (continuation).

C00Z	23(2)	25(2)	25(2)	-10.8(18)	2.7(17)	-3.5(17)
C010	24(2)	27(2)	21(2)	-1.7(18)	4.8(17)	-0.4(17)
C011	16.8(19)	28(2)	28(2)	-12.5(18)	4.4(16)	-4.9(17)
C012	34(2)	25(2)	30(2)	-13.9(19)	-4.5(19)	-4.2(19)
C013	30(2)	23(2)	34(2)	-14.1(19)	-3.8(19)	-4.3(18)
C014	33(2)	28(2)	25(2)	-11.6(19)	5.8(18)	-6.0(19)
C015	27(2)	29(2)	32(2)	-15(2)	-1.9(18)	0.8(18)
C016	31(2)	24(2)	30(2)	-15.4(19)	0.9(19)	-1.4(18)
C017	27(2)	30(2)	32(2)	-17(2)	11.1(19)	-10.7(18)
C018	28(2)	41(3)	31(3)	-11(2)	-1.4(19)	-10(2)
C019	27(2)	21(2)	28(2)	-11.8(18)	-0.3(18)	-1.5(17)
C01A	26(2)	32(2)	30(2)	-10(2)	5.8(18)	-5.7(19)
C01B	38(3)	26(2)	26(2)	-11.1(19)	0.6(19)	-0.5(19)
C01C	35(3)	34(3)	23(2)	-8(2)	-0.8(19)	-1(2)
C01D	33(2)	39(3)	27(2)	-17(2)	5.8(19)	-15(2)
C01E	29(2)	31(2)	25(2)	-4.6(19)	0.9(18)	-1.0(19)
C01F	26(2)	28(2)	36(3)	-17(2)	-2.0(19)	0.5(18)
C01G	29(2)	26(2)	21(2)	-1.0(18)	-0.5(17)	-3.4(18)
C01H	25(2)	32(2)	29(2)	-5(2)	4.0(18)	-4.3(19)
C01I	40(3)	36(3)	29(2)	-17(2)	8(2)	-10(2)
C01J	30(2)	38(3)	26(2)	-12(2)	6.0(19)	-11(2)
C01K	39(3)	45(3)	25(2)	-17(2)	9(2)	-18(2)
C01L	29(2)	39(3)	22(2)	-7(2)	2.2(18)	-11(2)
C01M	28(2)	41(3)	24(2)	-10(2)	3.4(18)	-3(2)
C01N	26(2)	54(3)	36(3)	-25(2)	2(2)	-5(2)
C01O	38(3)	38(3)	35(3)	-16(2)	7(2)	-3(2)
C01P	42(3)	35(3)	28(2)	-16(2)	5(2)	-6(2)
C01Q	31(2)	45(3)	27(2)	-18(2)	-1.9(19)	0(2)
C01R	38(3)	45(3)	23(2)	-11(2)	1(2)	-12(2)
C01S	40(3)	40(3)	31(3)	-12(2)	-2(2)	-7(2)
C01T	61(4)	43(3)	26(3)	-11(2)	-3(2)	-3(3)
C01U	37(3)	52(3)	31(3)	-13(2)	-4(2)	-10(2)
C01V	44(3)	44(3)	23(2)	-6(2)	-2(2)	4(2)
C01W	55(4)	75(5)	52(4)	-45(4)	9(3)	-7(3)

Supplementary Table 18. Bond Lengths for 13a.

Atom	Atom	Length/Å	Atom	Atom	Length/Å
S001	O007	1.437(3)	C00P	C01C	1.395(7)
S001	O008	1.431(3)	C00R	C00S	1.341(6)
S001	N00D	1.636(4)	C00T	C00W	1.400(6)
S001	C00K	1.756(4)	C00T	C014	1.392(6)
S002	O005	1.434(3)	C00U	C01D	1.384(6)
S002	O006	1.429(3)	C00W	C01L	1.387(7)
S002	N00E	1.642(4)	C00X	C01M	1.400(7)
S002	C00Z	1.755(5)	C00Y	C01N	1.382(7)
S003	O00B	1.440(4)	C00Z	C018	1.390(6)
S003	O00C	1.435(3)	C00Z	C01J	1.400(6)
S003	C00N	1.763(5)	C010	C01E	1.402(7)
S003	C00S	1.758(5)	C011	C017	1.528(6)
S004	O009	1.450(4)	C012	C013	1.391(7)
S004	O00A	1.437(4)	C012	C016	1.401(7)
S004	C00V	1.754(5)	C013	C019	1.385(6)
S004	C019	1.764(5)	C014	C01P	1.405(7)
N00D	C00I	1.481(5)	C015	C019	1.395(6)
N00D	C00Q	1.468(5)	C015	C01F	1.394(7)
N00E	C00O	1.474(5)	C016	C01F	1.405(7)
N00E	C011	1.485(5)	C016	C01O	1.502(7)
C00F	C00G	1.333(6)	C018	C01U	1.398(7)
C00F	C00L	1.507(6)	C01A	C01H	1.392(7)
C00F	C00M	1.495(6)	C01B	C01I	1.386(7)
C00G	C00Q	1.503(6)	C01B	C01T	1.508(7)
C00H	C00J	1.333(6)	C01B	C01U	1.396(7)
C00H	C00R	1.494(6)	C01C	C01V	1.389(7)
C00H	C017	1.509(6)	C01D	C01R	1.387(7)
C00I	C00L	1.529(6)	C01E	C01G	1.398(6)
C00J	C00O	1.502(6)	C01G	C01H	1.398(7)
C00K	C00X	1.399(6)	C01G	C01S	1.512(7)
C00K	C00Y	1.395(6)	C01I	C01J	1.383(7)
C00M	C00T	1.482(6)	C01K	C01L	1.394(7)
C00M	C00V	1.339(6)	C01K	C01P	1.390(7)
C00N	C010	1.394(6)	C01M	C01Q	1.399(7)
C00N	C01A	1.398(6)	C01N	C01Q	1.397(7)
C00P	C00R	1.490(6)	C01Q	C01W	1.521(7)
C00P	C00U	1.405(6)	C01R	C01V	1.402(8)

Supplementary Table 19. Bond Angles for 13a.

Atom	Atom	Atom	Angle/°	Atom	Atom	Atom	Angle/°
O007	S001	N00D	107.01(19)	C00P	C00R	C00H	116.7(4)
O007	S001	C00K	108.4(2)	C00S	C00R	C00H	124.7(4)
O008	S001	O007	120.5(2)	C00S	C00R	C00P	118.6(4)
O008	S001	N00D	106.42(19)	C00R	C00S	S003	128.4(4)
O008	S001	C00K	107.0(2)	C00W	C00T	C00M	121.1(4)
N00D	S001	C00K	106.85(19)	C014	C00T	C00M	118.9(4)
O005	S002	N00E	106.70(19)	C014	C00T	C00W	119.9(4)
O005	S002	C00Z	108.2(2)	C01D	C00U	C00P	120.1(4)
O006	S002	O005	119.8(2)	C00M	C00V	S004	128.4(4)
O006	S002	N00E	106.24(19)	C01L	C00W	C00T	119.8(4)
O006	S002	C00Z	108.0(2)	C00K	C00X	C01M	118.5(4)
N00E	S002	C00Z	107.27(19)	C01N	C00Y	C00K	119.6(4)
O00B	S003	C00N	107.5(2)	C018	C00Z	S002	120.4(3)
O00B	S003	C00S	104.6(2)	C018	C00Z	C01J	120.4(4)
O00C	S003	O00B	117.5(2)	C01J	C00Z	S002	119.2(3)
O00C	S003	C00N	108.4(2)	C00N	C010	C01E	119.3(4)
O00C	S003	C00S	109.9(2)	N00E	C011	C017	108.6(4)
C00S	S003	C00N	108.6(2)	C013	C012	C016	120.9(4)
O009	S004	C00V	109.3(2)	C019	C013	C012	119.2(4)
O009	S004	C019	108.8(2)	C00T	C014	C01P	120.0(4)
O00A	S004	O009	117.7(2)	C01F	C015	C019	118.6(4)
O00A	S004	C00V	105.0(2)	C012	C016	C01F	118.6(4)
O00A	S004	C019	107.6(2)	C012	C016	C01O	121.0(4)
C00V	S004	C019	108.1(2)	C01F	C016	C01O	120.4(4)
C00I	N00D	S001	116.6(3)	C00H	C017	C011	110.5(4)
C00Q	N00D	S001	114.5(3)	C00Z	C018	C01U	118.9(5)
C00Q	N00D	C00I	114.7(3)	C013	C019	S004	119.5(4)
C00O	N00E	S002	115.0(3)	C013	C019	C015	121.6(4)
C00O	N00E	C011	113.5(3)	C015	C019	S004	118.8(4)
C011	N00E	S002	116.2(3)	C01H	C01A	C00N	118.7(4)
C00G	C00F	C00L	121.8(4)	C01I	C01B	C01T	120.4(5)
C00G	C00F	C00M	120.9(4)	C01I	C01B	C01U	118.8(5)
C00M	C00F	C00L	117.3(3)	C01U	C01B	C01T	120.9(5)
C00F	C00G	C00Q	124.0(4)	C01V	C01C	C00P	121.0(5)

Supplementary Table 19. Bond Angles for 13a (continuation).

C00J	C00H	C00R	120.8(4)	C00U	C01D	C01R	120.5(5)
C00J	C00H	C017	121.6(4)	C01G	C01E	C010	120.1(4)
C00R	C00H	C017	117.6(4)	C015	C01F	C016	121.1(4)
N00D	C00I	C00L	108.9(3)	C01E	C01G	C01S	120.6(4)
C00H	C00J	C00O	124.0(4)	C01H	C01G	C01E	119.5(4)
C00X	C00K	S001	120.1(3)	C01H	C01G	C01S	119.9(4)
C00Y	C00K	S001	119.2(3)	C01A	C01H	C01G	121.1(4)
C00Y	C00K	C00X	120.6(4)	C01J	C01I	C01B	121.2(5)
C00F	C00L	C00I	111.2(3)	C01I	C01J	C00Z	119.6(4)
C00T	C00M	C00F	117.3(4)	C01P	C01K	C01L	119.7(4)
C00V	C00M	C00F	123.3(4)	C00W	C01L	C01K	120.7(4)
C00V	C00M	C00T	119.4(4)	C01Q	C01M	C00X	121.6(4)
C010	C00N	S003	119.3(3)	C00Y	C01N	C01Q	121.5(5)
C010	C00N	C01A	121.3(4)	C01K	C01P	C014	119.9(5)
C01A	C00N	S003	119.1(4)	C01M	C01Q	C01W	121.5(5)
N00E	C00O	C00J	111.2(3)	C01N	C01Q	C01M	118.1(5)
C00U	C00P	C00R	121.5(4)	C01N	C01Q	C01W	120.4(5)
C01C	C00P	C00R	119.3(4)	C01D	C01R	C01V	120.2(4)
C01C	C00P	C00U	119.1(4)	C01B	C01U	C018	121.1(5)
N00D	C00Q	C00G	111.1(3)	C01C	C01V	C01R	119.2(5)

Supplementary Table 20. Torsion Angles for 13a.

A	B	C	D	Angle/°	A	B	C	D	Angle/°
S001	N00D	C00I	C00L	159.5(3)	C00N	C010	C01E	C01G	1.1(7)
S001	N00D	C00Q	C00G	-178.9(3)	C00N	C01A	C01H	C01G	1.0(7)
S001	C00K	C00X	C01M	176.0(4)	C00O	N00E	C011	C017	64.5(4)
S001	C00K	C00Y	C01N	-174.4(4)	C00P	C00R	C00S	S003	-178.1(3)
S002	N00E	C00O	C00J	-179.6(3)	C00P	C00U	C01D	C01R	0.5(7)
S002	N00E	C011	C017	-158.9(3)	C00P	C01C	C01V	C01R	-0.7(8)
S002	C00Z	C018	C01U	-178.3(4)	C00Q	N00D	C00I	C00L	-62.7(5)
S002	C00Z	C01J	C01I	177.6(4)	C00R	C00H	C00J	C00O	-177.4(4)
S003	C00N	C010	C01E	173.6(3)	C00R	C00H	C017	C011	-161.3(4)
S003	C00N	C01A	C01H	-174.6(4)	C00R	C00P	C00U	C01D	174.3(4)
O005	S002	N00E	C00O	-174.2(3)	C00R	C00P	C01C	C01V	-174.3(5)
O005	S002	N00E	C011	49.7(3)	C00S	S003	C00N	C010	124.7(4)
O005	S002	C00Z	C018	-17.2(4)	C00S	S003	C00N	C01A	-61.0(4)
O005	S002	C00Z	C01J	164.6(4)	C00T	C00M	C00V	S004	179.1(3)
O006	S002	N00E	C00O	-45.3(3)	C00T	C00W	C01L	C01K	-1.0(7)
O006	S002	N00E	C011	178.6(3)	C00T	C014	C01P	C01K	0.5(7)
O006	S002	C00Z	C018	-148.2(4)	C00U	C00P	C00R	C00H	151.2(4)
O006	S002	C00Z	C01J	33.5(4)	C00U	C00P	C00R	C00S	-30.5(6)
O007	S001	N00D	C00I	-46.2(4)	C00U	C00P	C01C	C01V	1.5(7)
O007	S001	N00D	C00Q	176.0(3)	C00U	C01D	C01R	C01V	0.3(8)
O007	S001	C00K	C00X	26.5(4)	C00V	S004	C019	C013	-126.8(4)
O007	S001	C00K	C00Y	-157.1(4)	C00V	S004	C019	C015	57.1(4)
O008	S001	N00D	C00I	-176.2(3)	C00V	C00M	C00T	C00W	32.2(6)
O008	S001	N00D	C00Q	45.9(3)	C00V	C00M	C00T	C014	-143.6(4)
O008	S001	C00K	C00X	157.8(4)	C00W	C00T	C014	C01P	-2.2(7)
O008	S001	C00K	C00Y	-25.8(4)	C00X	C00K	C00Y	C01N	2.0(7)
O009	S004	C00V	C00M	-77.4(5)	C00X	C01M	C01Q	C01N	2.5(8)
O009	S004	C019	C013	-8.2(4)	C00X	C01M	C01Q	C01W	-175.2(5)
O009	S004	C019	C015	175.7(3)	C00Y	C00K	C00X	C01M	-0.4(7)
O00A	S004	C00V	C00M	155.5(4)	C00Y	C01N	C01Q	C01M	-0.8(8)
O00A	S004	C019	C013	120.3(4)	C00Y	C01N	C01Q	C01W	176.8(5)
O00A	S004	C019	C015	-55.8(4)	C00Z	S002	N00E	C00O	70.0(3)
O00B	S003	C00N	C010	-122.6(4)	C00Z	S002	N00E	C011	-66.0(3)
O00B	S003	C00N	C01A	51.7(4)	C00Z	C018	C01U	C01B	1.2(8)

Supplementary Table 20. Torsion Angles for 13a (continuation).

O00B	S003	C00S	C00R	-155.7(4)	C010	C00N	C01A	C01H	-0.4(7)
O00C	S003	C00N	C010	5.3(4)	C010	C01E	C01G	C01H	-0.6(7)
O00C	S003	C00N	C01A	179.6(4)	C010	C01E	C01G	C01S	179.0(4)
O00C	S003	C00S	C00R	77.4(5)	C011	N00E	C00O	C00J	-42.4(5)
N00D	S001	C00K	C00X	-88.5(4)	C012	C013	C019	S004	-174.5(3)
N00D	S001	C00K	C00Y	87.9(4)	C012	C013	C019	C015	1.4(7)
N00D	C00I	C00L	C00F	48.1(5)	C012	C016	C01F	C015	0.3(7)
N00E	S002	C00Z	C018	97.6(4)	C013	C012	C016	C01F	1.2(7)
N00E	S002	C00Z	C01J	-80.6(4)	C013	C012	C016	C01O	-177.2(4)
N00E	C011	C017	C00H	-50.6(5)	C014	C00T	C00W	C01L	2.5(6)
C00F	C00G	C00Q	N00D	-10.1(6)	C016	C012	C013	C019	-2.0(7)
C00F	C00M	C00T	C00W	-150.0(4)	C017	C00H	C00J	C00O	0.8(7)
C00F	C00M	C00T	C014	34.1(5)	C017	C00H	C00R	C00P	-61.9(5)
C00F	C00M	C00V	S004	1.5(7)	C017	C00H	C00R	C00S	119.8(5)
C00G	C00F	C00L	C00I	-19.1(6)	C018	C00Z	C01J	C01I	-0.7(7)
C00G	C00F	C00M	C00T	-112.4(5)	C019	S004	C00V	C00M	40.8(5)
C00G	C00F	C00M	C00V	65.3(6)	C019	C015	C01F	C016	-0.8(7)
C00H	C00J	C00O	N00E	9.3(6)	C01A	C00N	C010	C01E	-0.6(7)
C00H	C00R	C00S	S003	0.1(7)	C01B	C01I	C01J	C00Z	0.3(7)
C00I	N00D	C00Q	C00G	42.4(5)	C01C	C00P	C00R	C00H	-33.2(6)
C00J	C00H	C00R	C00P	116.4(5)	C01C	C00P	C00R	C00S	145.2(5)
C00J	C00H	C00R	C00S	-61.9(6)	C01C	C00P	C00U	C01D	-1.4(7)
C00J	C00H	C017	C011	20.4(6)	C01D	C01R	C01V	C01C	-0.2(8)
C00K	S001	N00D	C00I	69.7(3)	C01E	C01G	C01H	C01A	-0.5(7)
C00K	S001	N00D	C00Q	-68.1(3)	C01F	C015	C019	S004	176.0(3)
C00K	C00X	C01M	C01Q	-1.9(7)	C01F	C015	C019	C013	0.0(7)
C00K	C00Y	C01N	C01Q	-1.3(8)	C01I	C01B	C01U	C018	-1.6(8)
C00L	C00F	C00G	C00Q	-0.5(7)	C01J	C00Z	C018	C01U	-0.1(8)
C00L	C00F	C00M	C00T	65.4(5)	C01L	C01K	C01P	C014	1.0(7)
C00L	C00F	C00M	C00V	-116.9(5)	C01O	C016	C01F	C015	178.7(4)
C00M	C00F	C00G	C00Q	177.1(4)	C01P	C01K	C01L	C00W	-0.7(7)
C00M	C00F	C00L	C00I	163.2(4)	C01S	C01G	C01H	C01A	180.0(5)
C00M	C00T	C00W	C01L	-173.4(4)	C01T	C01B	C01I	C01J	-179.6(5)
C00M	C00T	C014	C01P	173.7(4)	C01T	C01B	C01U	C018	178.8(5)
C00N	S003	C00S	C00R	-41.1(5)	C01U	C01B	C01I	C01J	0.8(8)

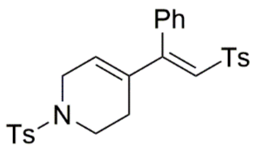

(CCDC 2117868)

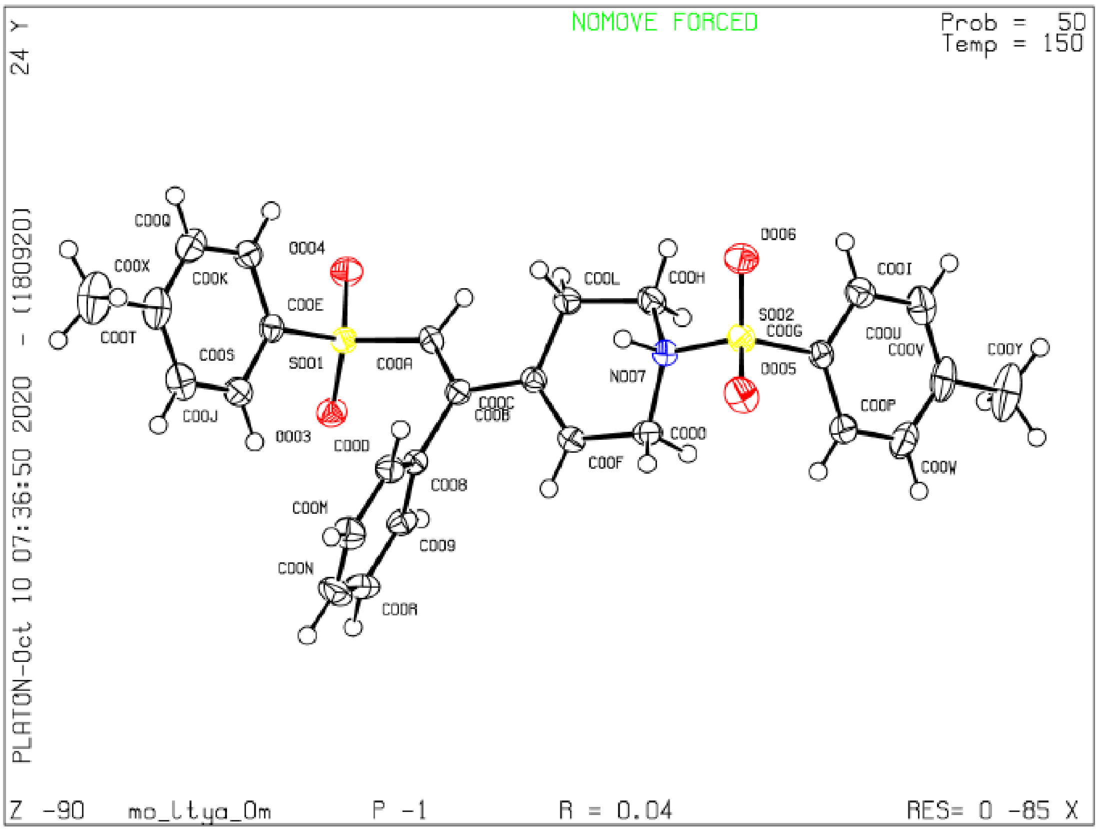

Supplementary Figure 14. X-ray structure of compound 13b. Single crystals for X-ray studies were grown by slow evaporation of a solution of compound **13b** in a mixture of THF/ n-heptane in NMR tube at room temperature. The X-ray data of **13b** is deposited in the Cambridge Crystallographic Data Centre with a number of CCDC: 2117868.

Supplementary Table 21. Crystal data and structure refinement for 13b.

Identification code	**13b**
Empirical formula	$C_{27}H_{28}NO_4S_2$
Formula weight	494.62
Temperature/K	150.0
Crystal system	triclinic
Space group	P-1
a/Å	6.3244(3)
b/Å	12.6335(5)
c/Å	15.2978(6)
α/°	84.593(2)
β/°	88.318(2)
γ/°	80.920(2)
Volume/Å^3	1201.47(9)
Z	2
ρ_{calc}g/cm^3	1.367
μ/mm^{-1}	0.257
F(000)	522.0
Crystal size/mm^3	0.2 × 0.2 × 0.1
Radiation	MoKα (λ = 0.71073)
2Θ range for data collection/°	4.038 to 52.928
Index ranges	-7 ≤ h ≤ 7, -15 ≤ k ≤ 15, -19 ≤ l ≤ 19
Reflections collected	31124
Independent reflections	4935 [R_{int} = 0.0462, R_{sigma} = 0.0271]
Data/restraints/parameters	4935/0/309
Goodness-of-fit on F^2	1.049
Final R indexes [I>=2σ (I)]	R_1 = 0.0368, wR_2 = 0.0909
Final R indexes [all data]	R_1 = 0.0497, wR_2 = 0.0995
Largest diff. peak/hole / e Å^{-3}	0.34/-0.74

Supplementary Table 22. Fractional Atomic Coordinates (×10^4) and Equivalent Isotropic Displacement Parameters (Å^2×10^3) for 13b. U_{eq} is defined as 1/3 of of the trace of the orthogonalised U_{IJ} tensor.

**Atom**	***x***	***y***	***z***	**U(eq)**
S001	6020.6(7)	5765.9(3)	3708.2(3)	21.75(12)
S002	8045.3(7)	8291.7(4)	8498.4(3)	23.83(12)
O003	3791(2)	5740.5(10)	3559.1(8)	27.1(3)
O004	7521(2)	4782.9(10)	3673.0(9)	29.3(3)
O005	6942(2)	9359.6(11)	8568.8(9)	35.2(3)
O006	10338(2)	8087.6(11)	8489.8(9)	32.6(3)
N007	7276(2)	7906.9(12)	7582.5(10)	22.5(3)
C008	3629(3)	7861.8(13)	4672.7(11)	20.3(4)
C009	1642(3)	7599.4(14)	4494.6(12)	24.6(4)
C00A	6375(3)	6190.8(14)	4751.7(11)	23.9(4)
C00B	5346(3)	7058.9(14)	5119.0(11)	21.0(4)
C00C	5927(3)	7271.1(13)	6011.7(11)	20.6(4)
C00D	4010(3)	8915.5(14)	4449.3(12)	24.8(4)
C00E	6868(3)	6740.3(14)	2935.2(11)	21.5(4)
C00F	4540(3)	7907.3(15)	6492.4(12)	26.4(4)
C00G	7144(3)	7443.4(14)	9365.7(11)	22.8(4)
C00H	8202(3)	6821.8(15)	7355.8(12)	26.9(4)
C00I	8572(3)	6607.2(15)	9763.3(13)	28.0(4)
C00J	5411(3)	7618.4(14)	2603.7(12)	26.3(4)
C00K	9006(3)	6619.3(16)	2659.3(12)	27.1(4)
C00L	8063(3)	6764.6(15)	6374.7(12)	25.9(4)
C00M	2465(3)	9673.9(15)	4026.4(13)	31.7(4)
C00N	507(3)	9397.2(16)	3839.4(13)	34.0(5)
C00O	4982(3)	8175.7(18)	7393.2(13)	32.2(4)
C00P	5002(3)	7604.5(17)	9633.0(12)	31.5(4)
C00Q	9652(3)	7389.4(17)	2049.3(13)	33.1(5)
C00R	92(3)	8369.6(16)	4078.2(13)	31.0(4)
C00S	6098(4)	8375.4(15)	1993.3(13)	33.0(5)
C00T	8224(4)	8271.7(16)	1706.6(13)	34.0(5)
C00U	7856(4)	5919.0(16)	10428.2(13)	35.0(5)
C00V	5715(4)	6036.8(17)	10684.8(13)	36.7(5)
C00W	4317(3)	6893.3(19)	10282.3(13)	37.7(5)
C00X	8961(5)	9109(2)	1045.1(16)	52.1(7)
C00Y	4924(5)	5244(2)	11366.5(15)	55.7(7)

Supplementary Table 23. Anisotropic Displacement Parameters (Å^2×10^3) for 13b. The Anisotropic displacement factor exponent takes the form: -2π^2[h^{2}a*^{2}U$_{11}$+2hka*b*U$_{12}$+…].

Atom	U_{11}	U_{22}	U_{33}	U_{23}	U_{13}	U_{12}
S001	22.5(2)	20.0(2)	22.1(2)	-3.42(16)	-0.06(17)	-0.49(17)
S002	23.9(2)	23.8(2)	24.4(2)	-2.46(17)	-2.36(17)	-4.97(17)
O003	24.2(7)	29.7(7)	28.5(7)	-4.7(5)	0.3(5)	-6.1(5)
O004	31.6(7)	21.5(6)	32.2(7)	-4.1(5)	1.0(6)	4.2(5)
O005	45.7(9)	23.5(7)	36.0(8)	-5.8(6)	-6.6(6)	-1.1(6)
O006	23.0(7)	41.8(8)	35.5(8)	-4.3(6)	-2.1(6)	-11.9(6)
N007	21.5(8)	25.3(8)	20.3(7)	-0.6(6)	-0.1(6)	-2.8(6)
C008	21.0(9)	21.3(8)	18.4(8)	-3.2(7)	0.9(7)	-1.4(7)
C009	22.6(9)	24.2(9)	27.7(9)	-7.2(7)	2.8(7)	-3.8(7)
C00A	24.7(9)	26.1(9)	19.6(9)	0.3(7)	-3.5(7)	-0.7(7)
C00B	19.7(9)	22.1(8)	21.3(9)	0.4(7)	1.2(7)	-5.0(7)
C00C	20.1(9)	20.0(8)	21.4(9)	0.5(7)	-1.3(7)	-3.4(7)
C00D	23.6(9)	24.7(9)	26.5(9)	-3.1(7)	-1.2(7)	-4.5(7)
C00E	24.0(9)	22.3(8)	19.3(8)	-4.5(7)	-1.2(7)	-4.9(7)
C00F	21.5(9)	33.2(10)	23.6(9)	-3.1(8)	-3.2(7)	-0.3(8)
C00G	24.1(9)	26.6(9)	18.9(8)	-5.6(7)	-3.3(7)	-5.4(7)
C00H	28.1(10)	25.4(9)	25.7(9)	-2.6(7)	-4.6(8)	1.4(7)
C00I	29.0(10)	27.5(9)	27.9(10)	-5.1(8)	-2.7(8)	-3.5(8)
C00J	28.0(10)	24.8(9)	25.9(9)	-5.2(7)	-2.8(7)	-1.1(7)
C00K	23.7(9)	33.5(10)	24.8(9)	-6.9(8)	-2.1(7)	-3.4(8)
C00L	24.7(9)	27.1(9)	23.8(9)	-3.9(7)	-1.5(7)	3.0(7)
C00M	38.3(11)	21.7(9)	33.1(11)	-0.6(8)	-1.4(9)	0.9(8)
C00N	32.6(11)	31.5(10)	34.4(11)	-7.2(9)	-10.4(9)	10.3(8)
C00O	20.8(9)	47.8(12)	26.5(10)	-10.9(9)	-2.0(8)	4.2(8)
C00P	25.6(10)	46.3(12)	22.4(9)	-5.9(8)	-1.8(8)	-3.4(9)
C00Q	29.7(10)	46.1(12)	27.9(10)	-10.5(9)	3.3(8)	-15.8(9)
C00R	20.0(9)	35.8(11)	37.9(11)	-15.7(9)	-3.9(8)	1.5(8)
C00S	46.3(12)	23.3(9)	29.0(10)	-2.9(8)	-5.7(9)	-3.0(9)
C00T	50.7(13)	31.1(10)	25.1(10)	-5.8(8)	-0.3(9)	-19.6(9)
C00U	52.1(13)	25.8(10)	26.7(10)	-2.7(8)	-3.9(9)	-3.8(9)
C00V	59.0(14)	37.9(11)	19.8(9)	-10.1(8)	4.1(9)	-24.7(10)
C00W	33.5(11)	60.7(14)	23.7(10)	-11.7(10)	6.0(8)	-19.0(10)
C00X	80.3(19)	43.1(13)	39.2(13)	-0.7(10)	6.0(12)	-32.3(13)
C00Y	97(2)	53.3(15)	27.4(11)	-11.3(10)	12.7(12)	-42.7(15)

Supplementary Table 24. Bond Lengths for 13b.

Atom	Atom	Length/Å	Atom	Atom	Length/Å
S001	O003	1.4411(13)	C00E	C00J	1.391(2)
S001	O004	1.4435(13)	C00E	C00K	1.394(3)
S001	C00A	1.7626(18)	C00F	C00O	1.494(3)
S001	C00E	1.7599(18)	C00G	C00I	1.383(3)
S002	O005	1.4298(14)	C00G	C00P	1.393(3)
S002	O006	1.4322(14)	C00H	C00L	1.515(2)
S002	N007	1.6369(15)	C00I	C00U	1.388(3)
S002	C00G	1.7646(19)	C00J	C00S	1.384(3)
N007	C00H	1.472(2)	C00K	C00Q	1.384(3)
N007	C00O	1.467(2)	C00M	C00N	1.383(3)
C008	C009	1.390(2)	C00N	C00R	1.379(3)
C008	C00B	1.496(2)	C00P	C00W	1.382(3)
C008	C00D	1.399(2)	C00Q	C00T	1.390(3)
C009	C00R	1.390(3)	C00S	C00T	1.392(3)
C00A	C00B	1.346(2)	C00T	C00X	1.512(3)
C00B	C00C	1.484(2)	C00U	C00V	1.388(3)
C00C	C00F	1.344(2)	C00V	C00W	1.392(3)
C00C	C00L	1.499(2)	C00V	C00Y	1.508(3)
C00D	C00M	1.384(3)			

Supplementary Table 25. Bond Angles for 13b.

Atom	Atom	Atom	Angle/°	Atom	Atom	Atom	Angle/°
O003	S001	O004	118.49(8)	C00J	C00E	S001	120.10(14)
O003	S001	C00A	110.79(8)	C00J	C00E	C00K	120.63(17)
O003	S001	C00E	107.90(8)	C00K	C00E	S001	119.27(14)
O004	S001	C00A	104.78(8)	C00C	C00F	C00O	124.13(17)
O004	S001	C00E	107.86(8)	C00I	C00G	S002	119.41(14)
C00E	S001	C00A	106.39(8)	C00I	C00G	C00P	120.50(18)
O005	S002	O006	119.98(9)	C00P	C00G	S002	120.08(14)
O005	S002	N007	106.71(8)	N007	C00H	C00L	109.70(15)
O005	S002	C00G	108.01(9)	C00G	C00I	C00U	119.58(19)
O006	S002	N007	106.82(8)	C00S	C00J	C00E	119.45(18)
O006	S002	C00G	107.59(9)	C00Q	C00K	C00E	118.75(18)
N007	S002	C00G	107.09(8)	C00C	C00L	C00H	112.97(15)
C00H	N007	S002	117.31(12)	C00N	C00M	C00D	119.88(18)
C00O	N007	S002	116.30(12)	C00R	C00N	C00M	120.04(18)
C00O	N007	C00H	112.69(15)	N007	C00O	C00F	110.81(15)
C009	C008	C00B	121.89(16)	C00W	C00P	C00G	118.82(19)
C009	C008	C00D	118.92(16)	C00K	C00Q	C00T	121.66(19)
C00D	C008	C00B	119.17(16)	C00N	C00R	C009	120.48(18)
C008	C009	C00R	120.05(17)	C00J	C00S	C00T	121.00(19)
C00B	C00A	S001	128.92(14)	C00Q	C00T	C00S	118.51(18)
C00A	C00B	C008	123.83(16)	C00Q	C00T	C00X	121.0(2)
C00A	C00B	C00C	119.85(16)	C00S	C00T	C00X	120.5(2)
C00C	C00B	C008	116.31(15)	C00V	C00U	C00I	121.1(2)
C00B	C00C	C00L	119.50(15)	C00U	C00V	C00W	118.13(19)
C00F	C00C	C00B	120.14(16)	C00U	C00V	C00Y	120.8(2)
C00F	C00C	C00L	120.36(16)	C00W	C00V	C00Y	121.1(2)
C00M	C00D	C008	120.59(17)	C00P	C00W	C00V	121.8(2)

Supplementary Table 26. Hydrogen Atom Coordinates (Å×10^4) and Isotropic Displacement Parameters (Å^2×10^3) for 13b.

**Atom**	***x***	***y***	***z***	**U(eq)**
H007	7925.92	8382.24	7125.81	27
H009	1343.21	6893.13	4657.66	29
H00A	7437.81	5745.73	5103.23	29
H00D	5340.75	9112.24	4589.12	30
H00F	3193	8205.04	6243.78	32
H00B	7412.48	6282.15	7673	32
H00C	9717.27	6656.16	7536.18	32
H00I	10034.73	6504.46	9582.61	34
H00J	3955.99	7697.49	2794.87	32
H00K	10000.34	6019.59	2885.63	33
H00E	9190.18	7131.61	6073.6	31
H00G	8344.85	6000.74	6247.37	31
H00M	2748.29	10383.2	3864.93	38
H00N	-552.04	9915.61	3546.3	41
H00H	4505.25	8953.4	7440.29	39
H00L	4162.74	7769.5	7831.36	39
H00P	4029.01	8192.67	9373.75	38
H00Q	11107.89	7313.04	1860.17	40
H00R	-1263.46	8186.39	3957.22	37
H00S	5103.52	8974.96	1766.34	40
H00U	8847.63	5357.79	10712.27	42
H00W	2851.47	6992	10458.55	45
H00O	9344.44	9697.08	1352.2	78
H00T	7802.26	9393.75	635.24	78
H00V	10212.37	8779.78	719.33	78
H00X	4314.47	4705.73	11074.21	83
H00Y	3821.32	5625.21	11736.21	83
H	6120.69	4884.74	11732.16	83

3.2 Computational Methods and Details.

3.2.1 Computational Methods.

All DFT-calculations were performed using Gaussian 16, Revision B.01. [12] The geometry optimization and frequency analysis were performed using the PBE-D3(BJ) functional.[13] The triple-ζ basis set Def2-TZVP was used for nickel, iron, and ruthenium atoms and the split-valence plus one polarization function Def2-SVP basis set was used for other atoms.[14] In all cases, the default integral grid (Ultrafine Grid) was employed. Frequency calculations were performed in order to obtain thermal corrections (298 K) and to confirm the nature of the stationary points (minima with no imaginary frequency or transition states with one imaginary frequency). All transition states were optimized using the default Berny algorithm implemented in the Gaussian 16 code. [12] For transition state structures, IRC calculations were undertaken to confirm that the transition states were connected to the correct minima. For further validation of energetics, single-point calculations were performed on the PBE/Def2-SVP optimized geometries using meta-hybrid GGA functional M06 [15] employing a valence triple-ζ-type of basis set Def2-TZVPP [14] for all atoms. The solvent effects (*N,N*-dimethylformamide (DMF): ε=37.219) were evaluated implicitly by a self-consistent reaction field (SCRF) approach for all the intermediates and transition states, using the SMD continuum solvation model.[16] Unless specified otherwise, ΔG was used throughout the text. The ΔG value was obtained by augmenting the E_{el} energy terms at M06 (SMD-DMF)/Def2-TZVPP with the respective free energy corrections at the PBE-D3(BJ)/Def2-TZVP (Ni, Fe, Ru)/Def2-SVP (other atoms) level in gas phase. The geometries were realized using CYLview20, Build 0001.[17]

3.2.2 Alternative catalytic cycle calculation.

Because 32% *syn*-selective product was obtained when only dppf was used as ligand (see Table 1, entry 7), the *syn*-selective catalytic cycle with only dppf as ligand was also explored (Supplementary Figure 15). The stereoselectivity (**P2-3ZTS** *vs.* **P2-3ETS**)

can be explained by the DFT calculation.

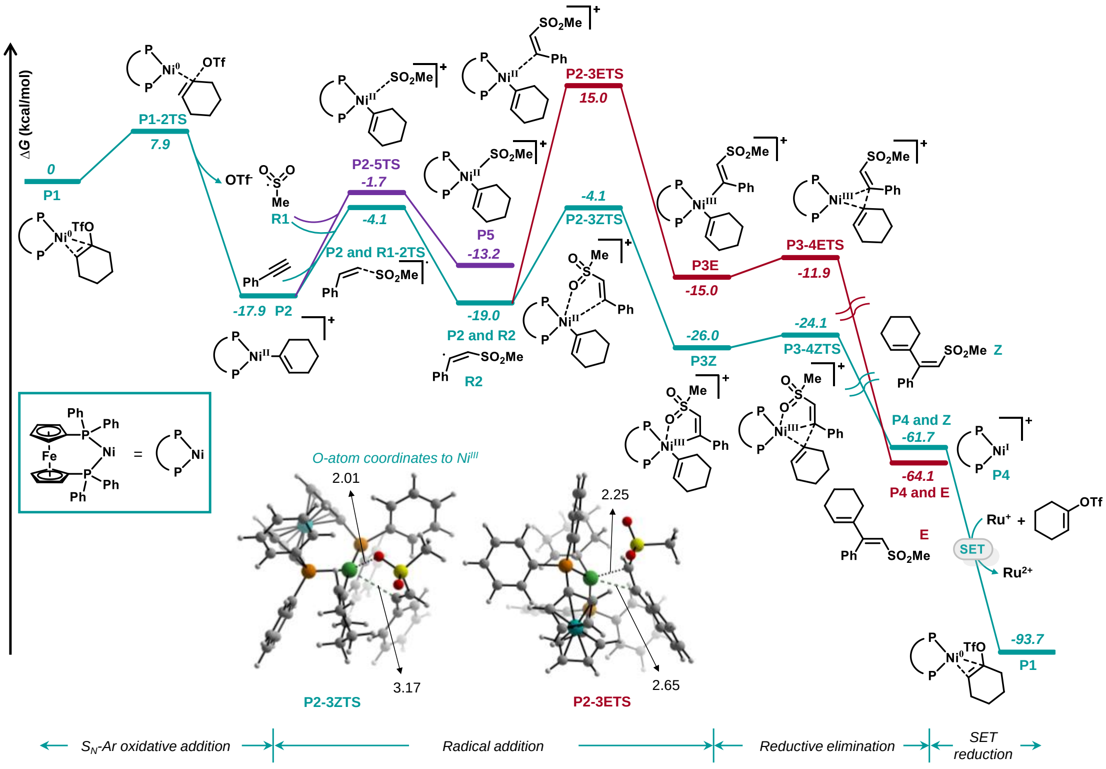

Supplementary Figure 15. Energy profile of the catalytic cycle with only dppf ligand. the *syn*-selective catalytic cycle with only dppf as ligand was explored. The stereoselectivity can be explained by the DFT calculation. The green pathway is the most likely reaction way. Red and purple way are disfavored because of higher energy barrier.

The alternative catalytic cycle with dppf was also explored (Supplementary Figure 16). This catalytic cycle involves S_N-Ar oxidative addition to Ni^0, vinyl group migratory insertion into internal carbon of alkyne, radical addition, reductive elimination, and SET reduction. Both regioselectivity (**P2-6ZTS** *vs.* **P2-8TS**) and stereoselectivity (**P6-7ZTS** *vs.* **P6Z-ETS**) can be explained by the DFT calculation. However, this pathway was not chosen as the main catalytic pathway due to the observed hydro-sulfonylation by-product **51** (Scheme 3C), which cannot be generated by this pathway.

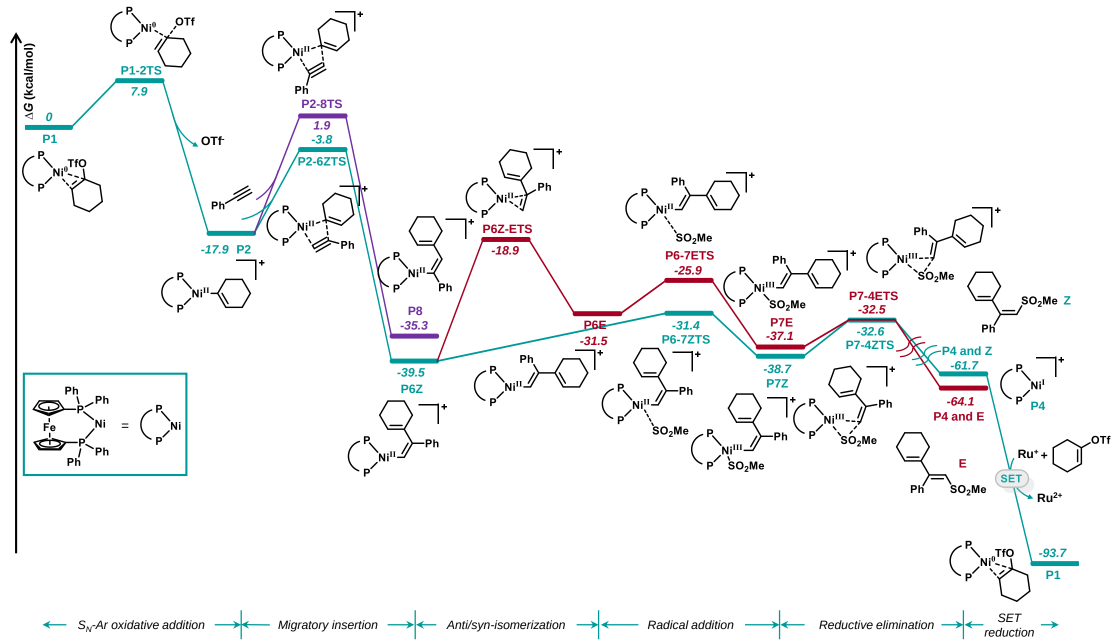

Supplementary Figure 16. Energy profile of the alternative catalytic cycle with dppf ligand. This catalytic cycle involves S_N-Ar oxidative addition to Ni^0, vinyl group migratory insertion into internal carbon of alkyne, radical addition, reductive elimination, and SET reduction. The green pathway is the most likely reaction way. Red and purple way are disfavored because of higher energy barrier.

The S_N-Ar oxidative addition energy barrier is lower than three-membered ring concerted oxidative addition energy barrier. The reason is that the electrically neutral Ni^{III} intermediate is too crowded. The calculation showed that the cationic Ni^{III} intermediate is more stable than electrically neutral Ni^{III} intermediate (Supplementary Figure 17).

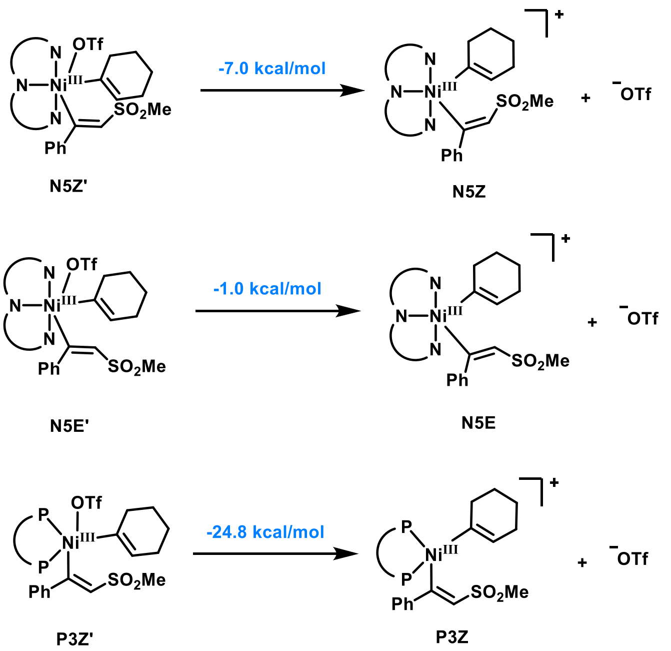

Supplementary Figure 17. Nickel intermediates comparison. Ni^{III} intermediates in cationic form and electrically neutral form.

The reduction of **N6** to Ni^0 intermediate **N8** by the Ru-photocatalyst is calculated to be thermodynamically disfavored (+2.2 kcal/mol). In contrast, the phen-Ni^I intermediate **Phen3** can undergo ligand exchange with dppf followed by SET reduction by the Ru-photocatalyst with an energy gain of 41.2 kcal/mol (Supplementary Figure 18).

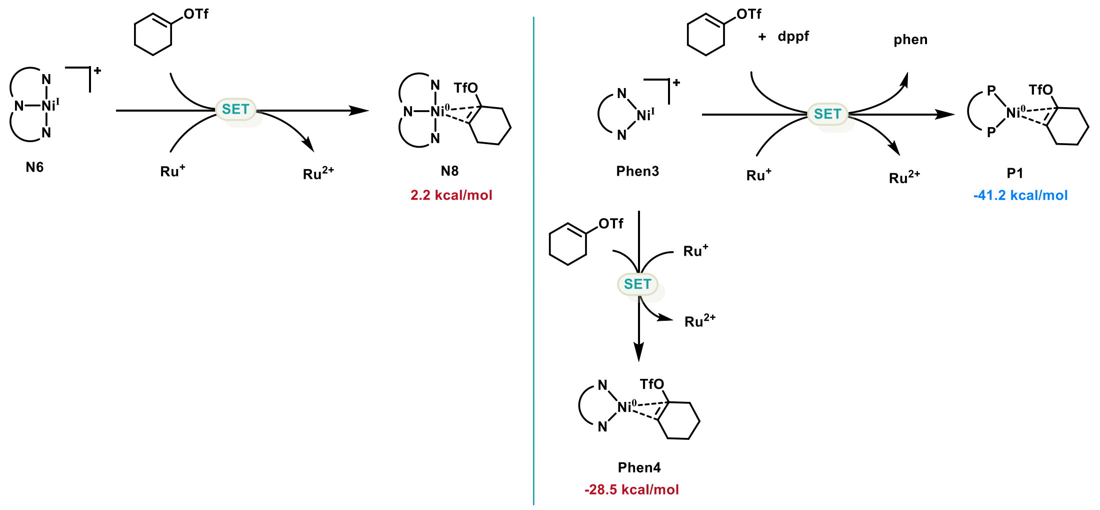

Supplementary Figure 18. Ni^I reduction pathways calculation. Comparison between Ni^I reduction with terpyridine ligand and dppf ligand. The left panel shows Ni^I reduction with terpyridine ligand, The right panel shows Ni^I reduction with dppf ligand.

The calculation showed that the tpy-Ni^{I} intermediate was easier to undergo radical addition, while dppf-Ni^{I} intermediate was easier to undergo SET reduction by Ru-photocatalyst (Supplementary Figure 19).

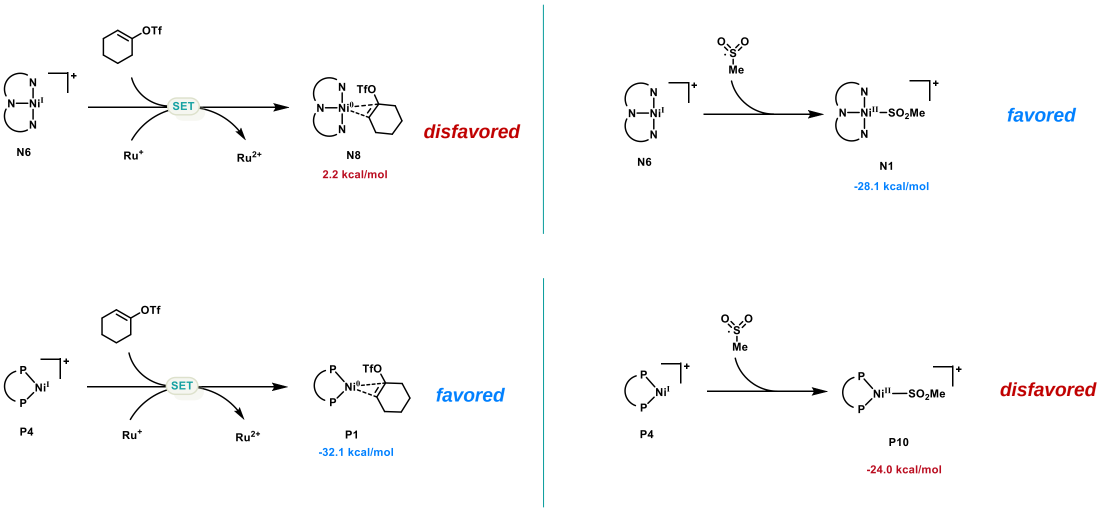

Supplementary Figure 19. Radical addition and SET reduction pathways comparison. Radical addition of sulfonyl radical to Ni^{I} versus SET reduction of Ni^{I} with terpyridine or dppf ligand. The upper panel shows the Radical addition to Ni^{I}, the lower panel shows the SET reduction of Ni^{I}.

We also investigated the benzene sulfinate radical converting to benzene radical pathway (Supplementary Figure 20). The result showed that this process is thermodynamically disfavored (from **R3** to **R4** + **SO₂**, $\Delta G^{o} = +13.5$ kcal/mol).

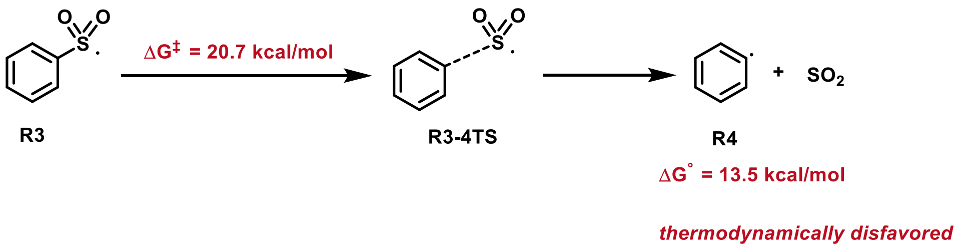

Supplementary Figure 20. Benzene radical generation. Benzene sulfinate radical converts to benzene radical.

3.2.3 IRC analysis of P2-3ZTS and P2-3ETS

In order to confirm the radical addition transition states for both *syn* and *anti* pathways

(**P2-3ZTS** and **P2-3ETS**), we conducted the IRC calculations for both TS (Supplementary Figure 21). The results were summarized below. The IRC calculations revealed that the calculated transition states (**P2-3ZTS** and **P2-3ETS**) are the corrected transition states leading to the corresponding Ni^{III} intermediates. The bond length of Ni-C decreases over the course of the IRC and eventually forms a covalent bond.

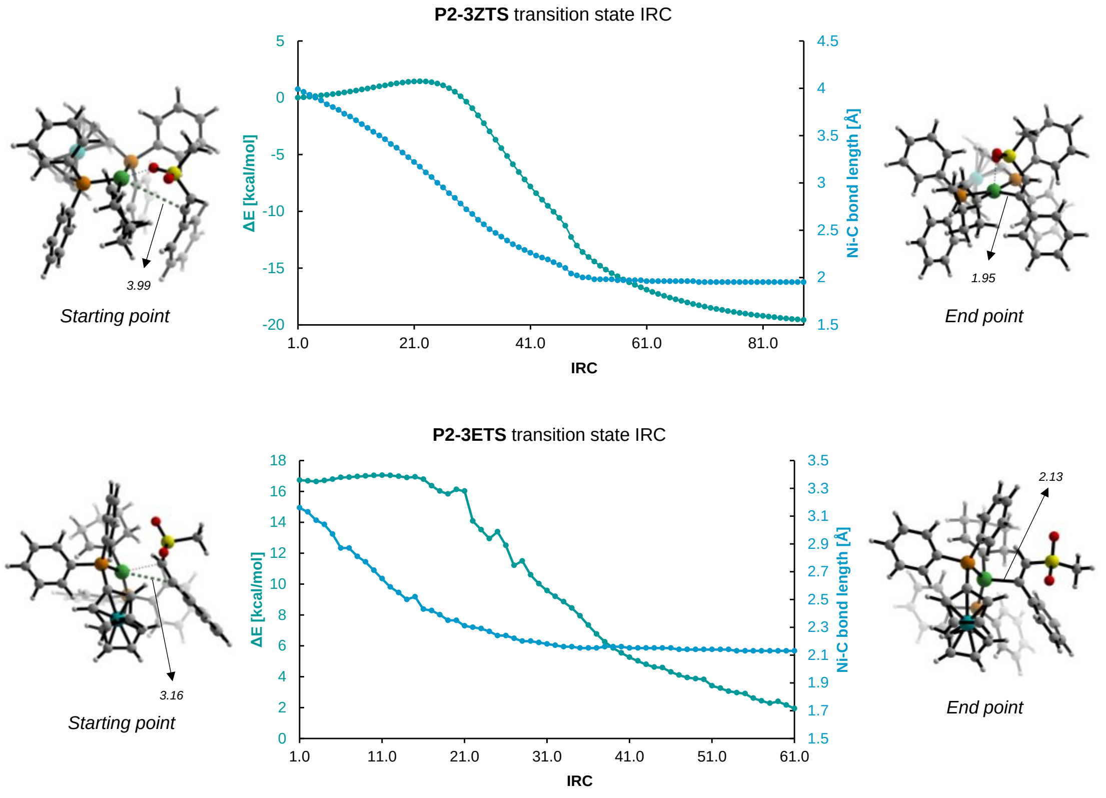

Supplementary Figure 21. IRC analysis. IRC calculations of radical addition transition states for both *syn*- and *anti*-pathways. The upper panel shows IRC calculations of the *syn* pathway, the lower panel shows IRC calculations of the *anti* pathway.

3.3 NMR Spectra.

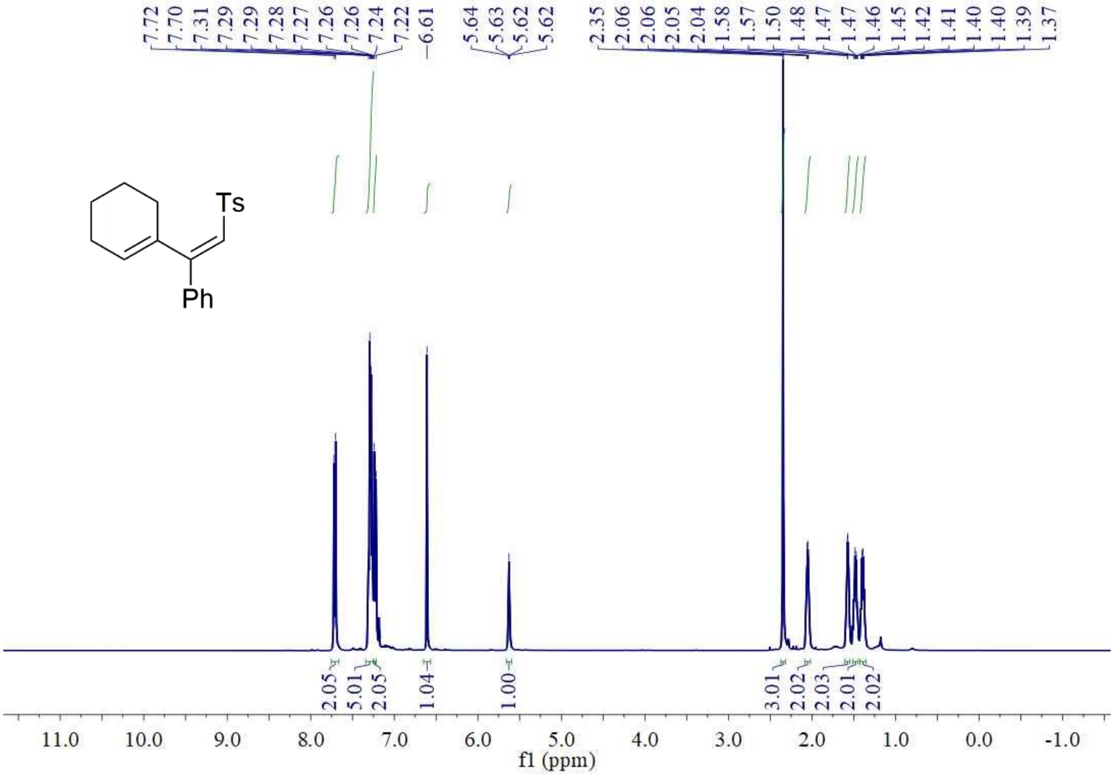

Supplementary Figure 22: ^{1}H NMR of 4a (400 MHz, $CDCl_3$).

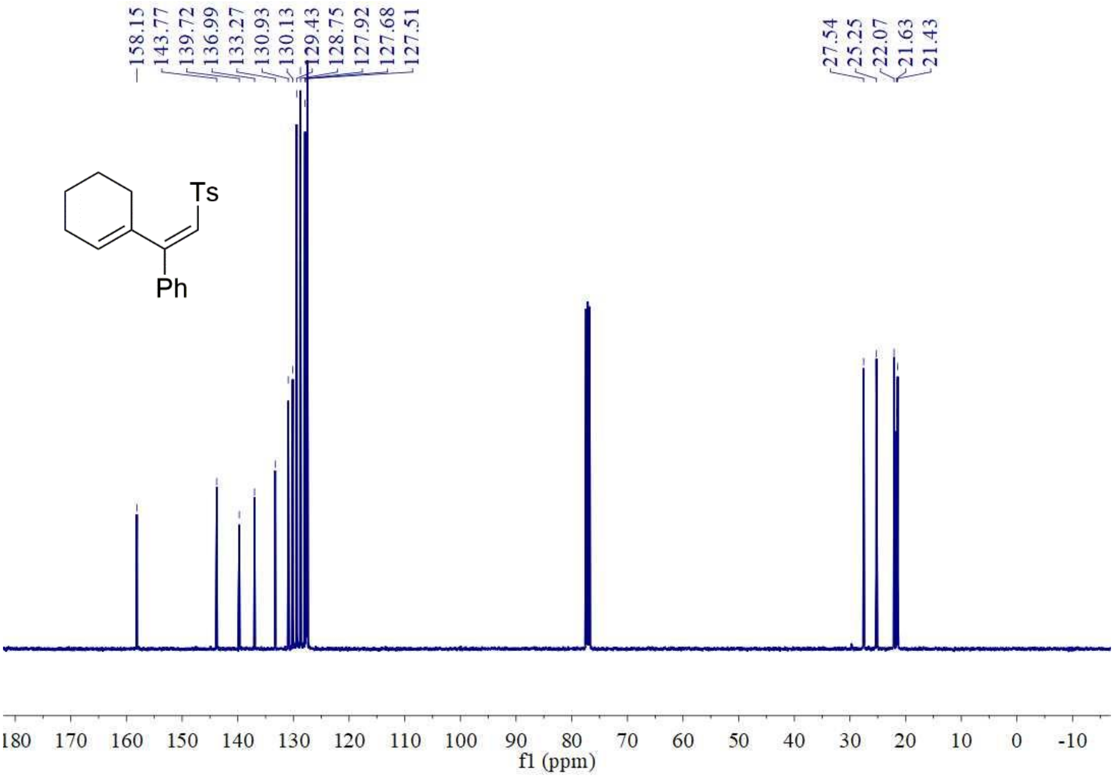

Supplementary Figure 23: ^{13}C NMR of 4a (100 MHz, $CDCl_3$).

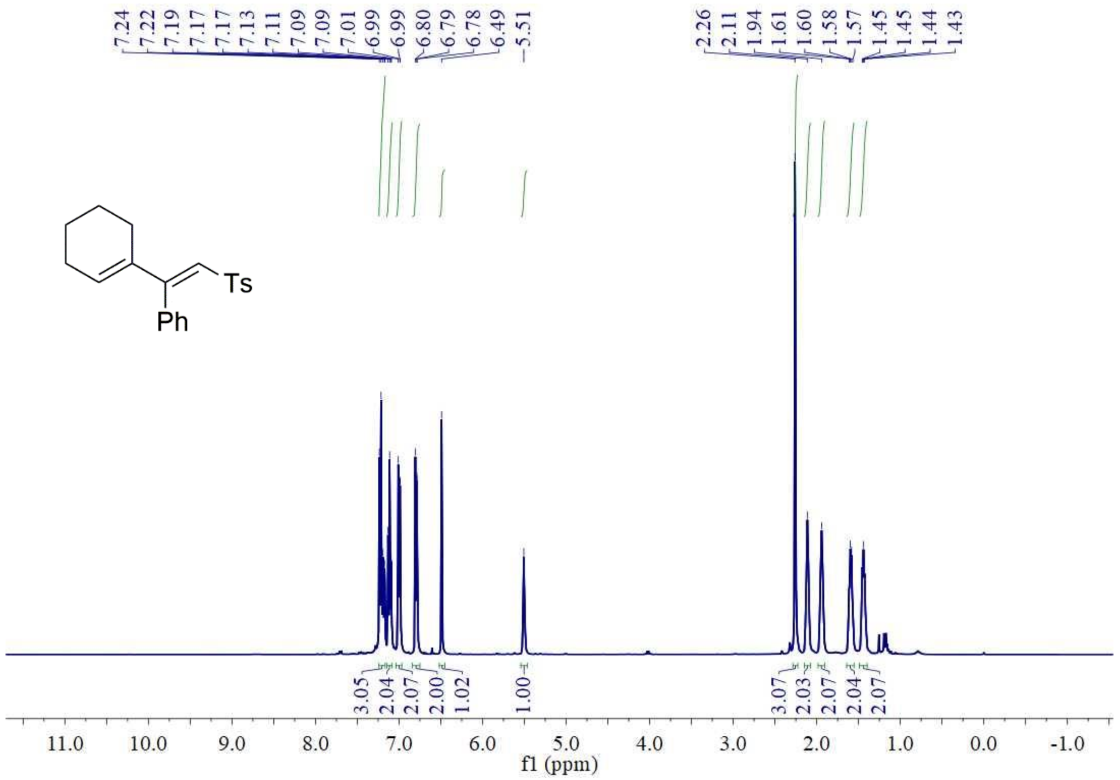

Supplementary Figure 24: ^{1}H NMR of 4b (400 MHz, $CDCl_3$).

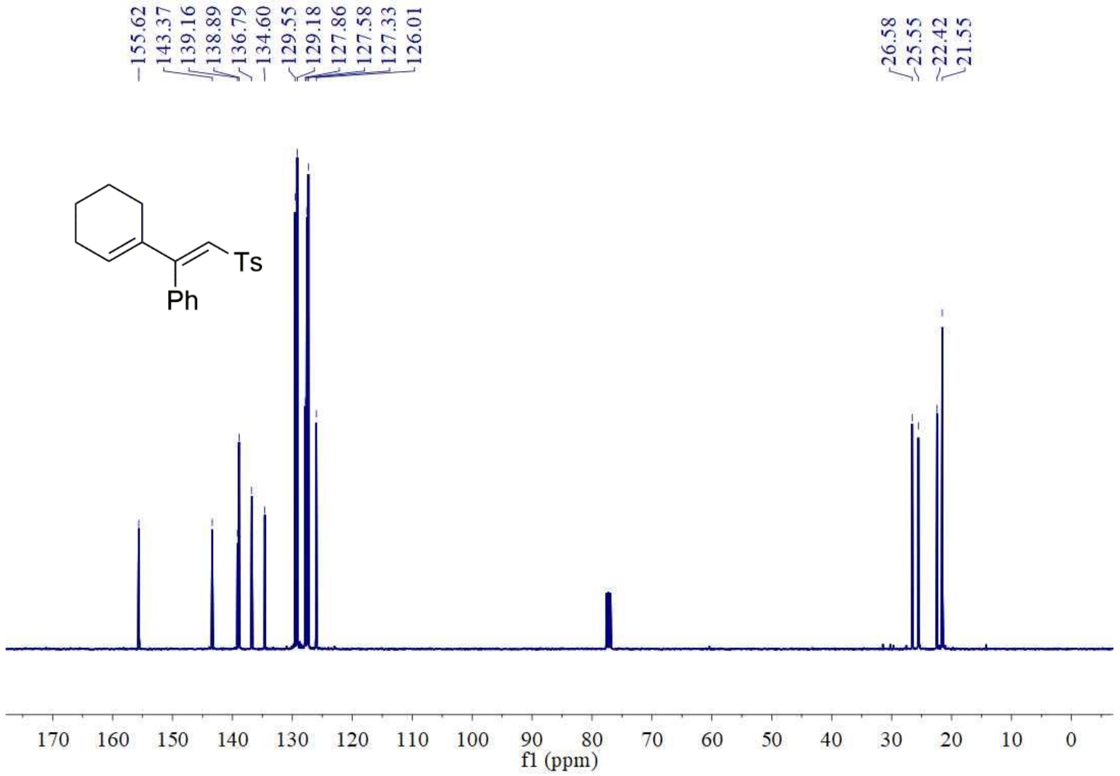

Supplementary Figure 25: ^{13}C NMR of 4b (100 MHz, $CDCl_3$).

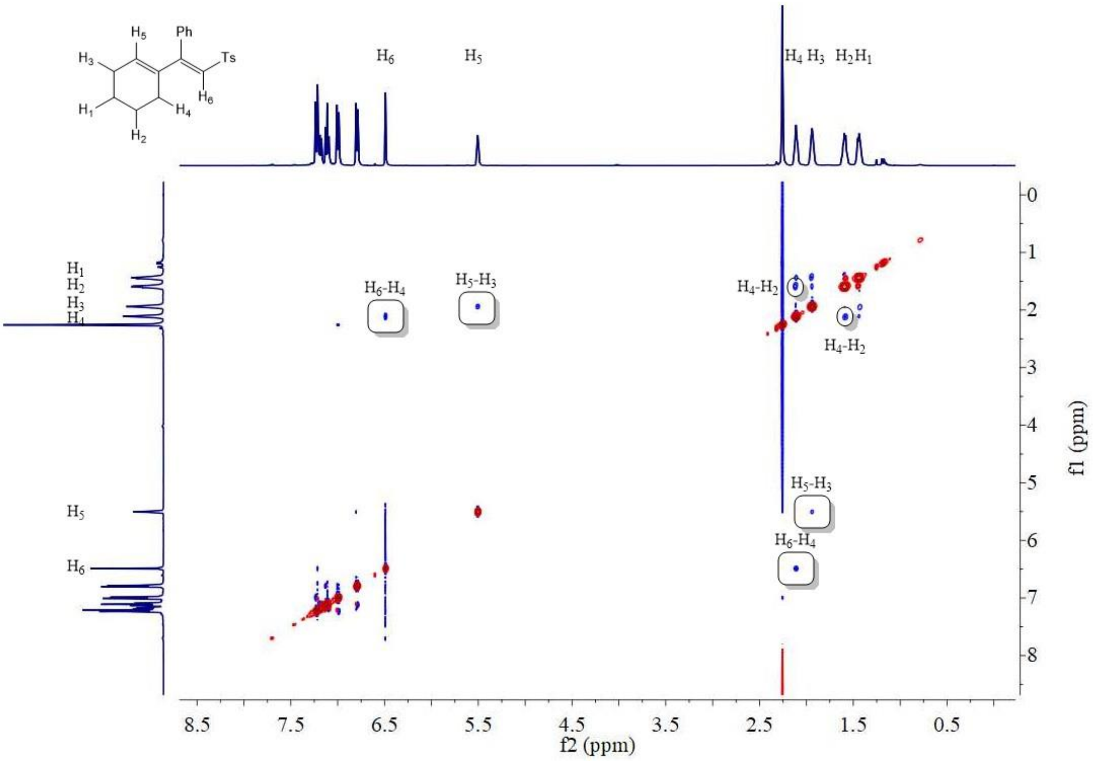

Supplementary Figure 26: NOESY of 4b.

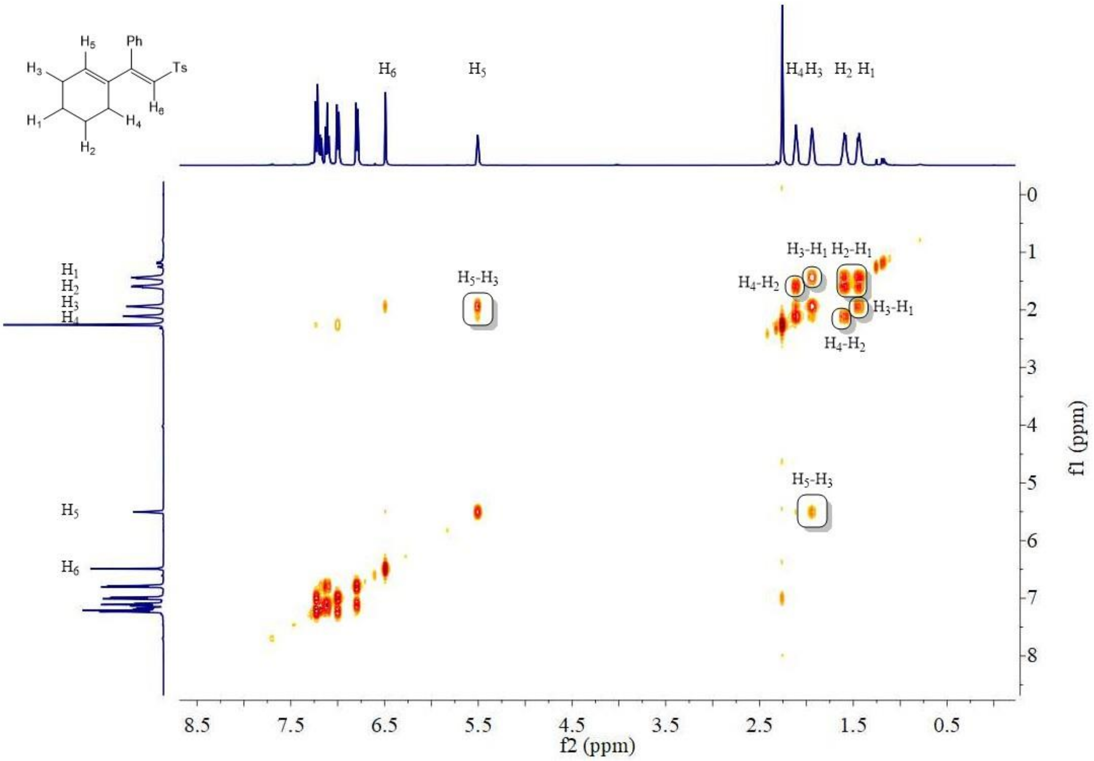

Supplementary Figure 27: COSY of 4b.

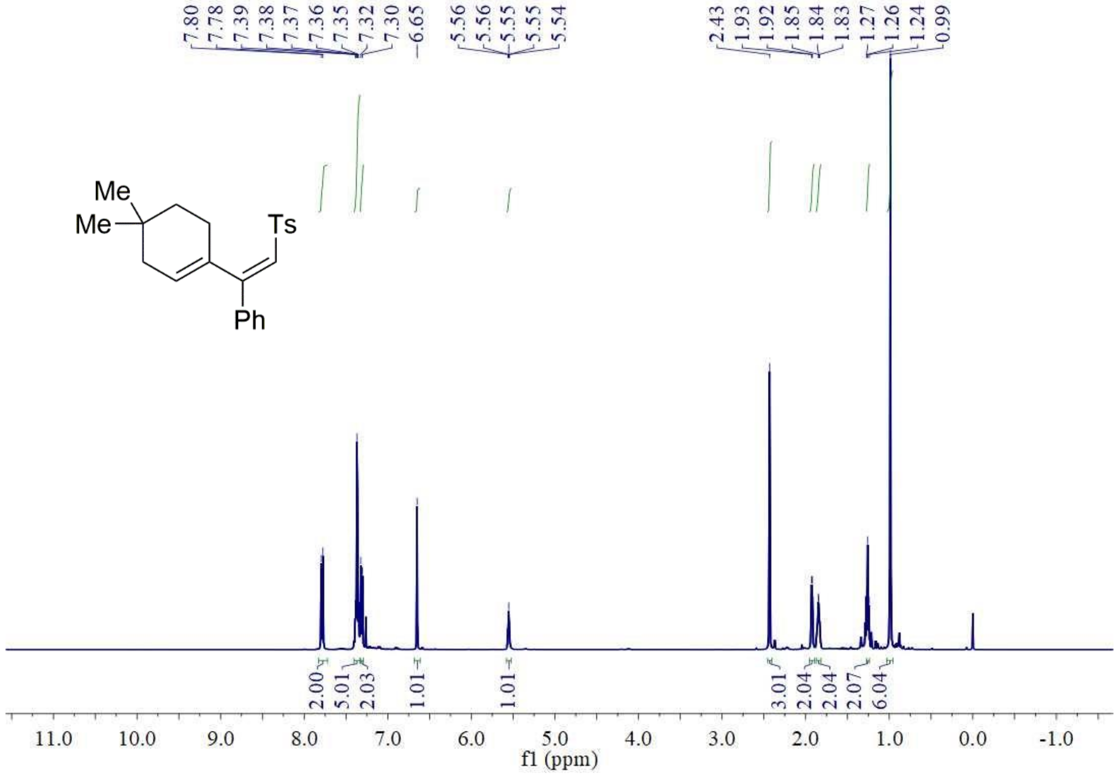

Supplementary Figure 28: ^{1}H NMR of 5a (400 MHz, $CDCl_3$).

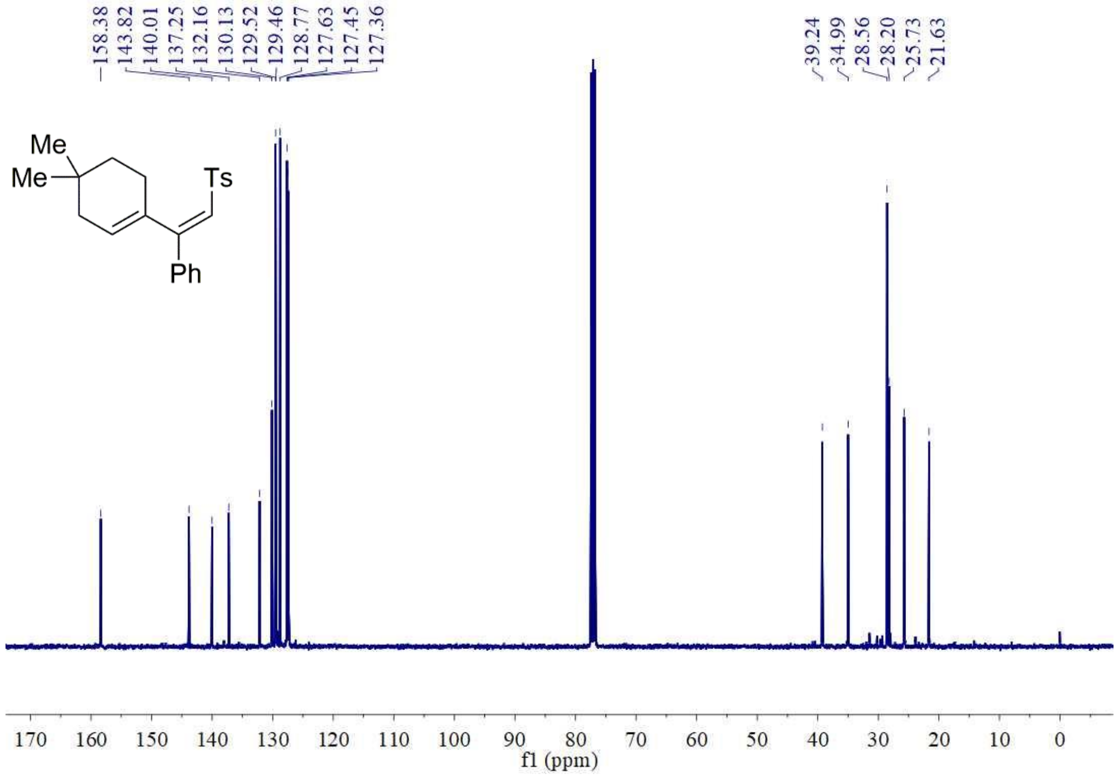

Supplementary Figure 29: ^{13}C NMR of 5a (100 MHz, $CDCl_3$).

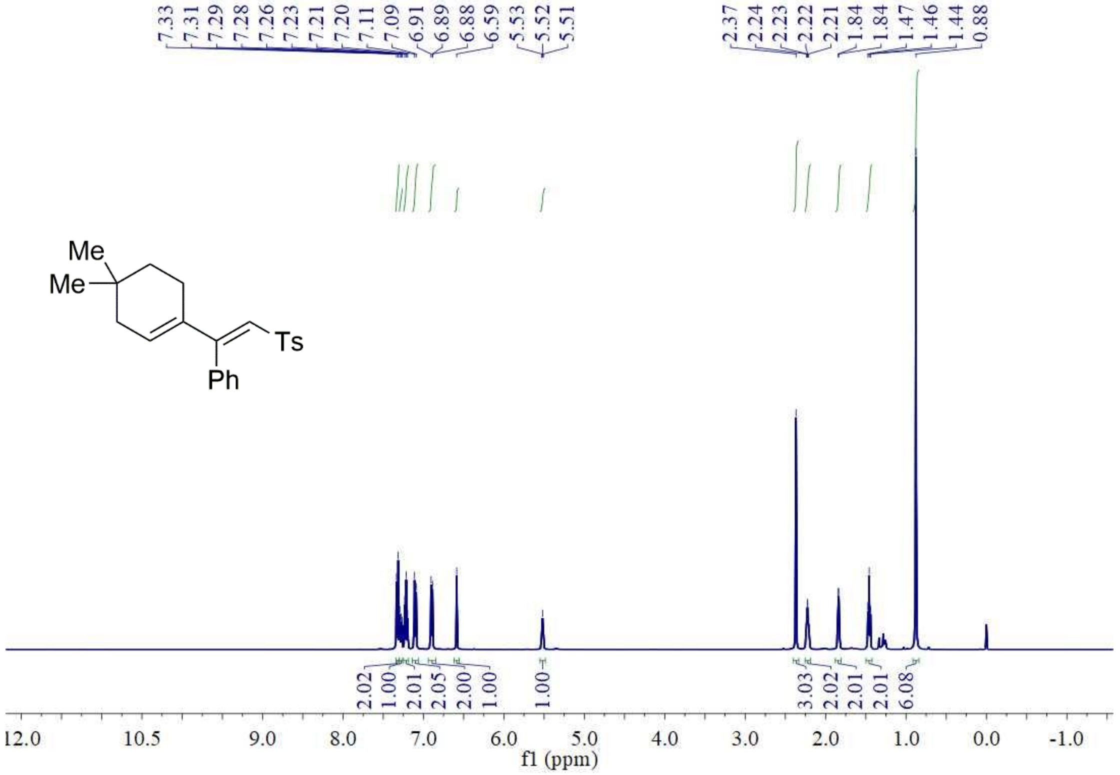

Supplementary Figure 30: ¹H NMR of 5b (400 MHz, $CDCl_3$).

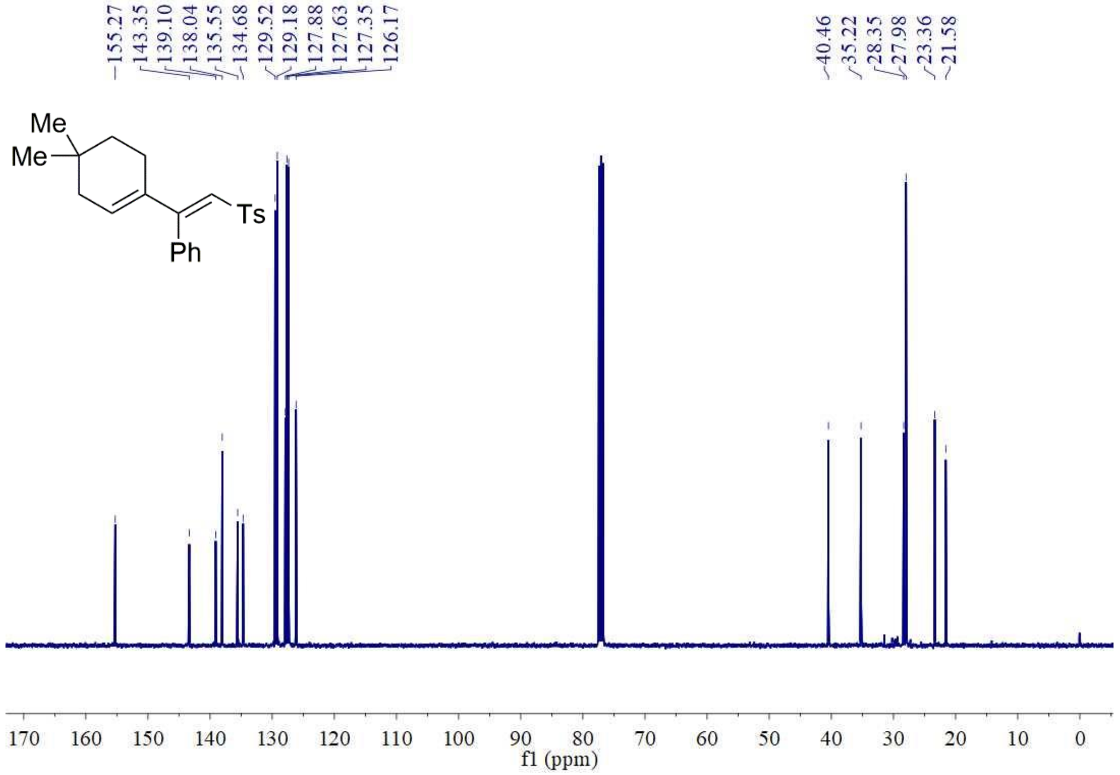

Supplementary Figure 31: ¹³C NMR of 5b (100 MHz, $CDCl_3$).

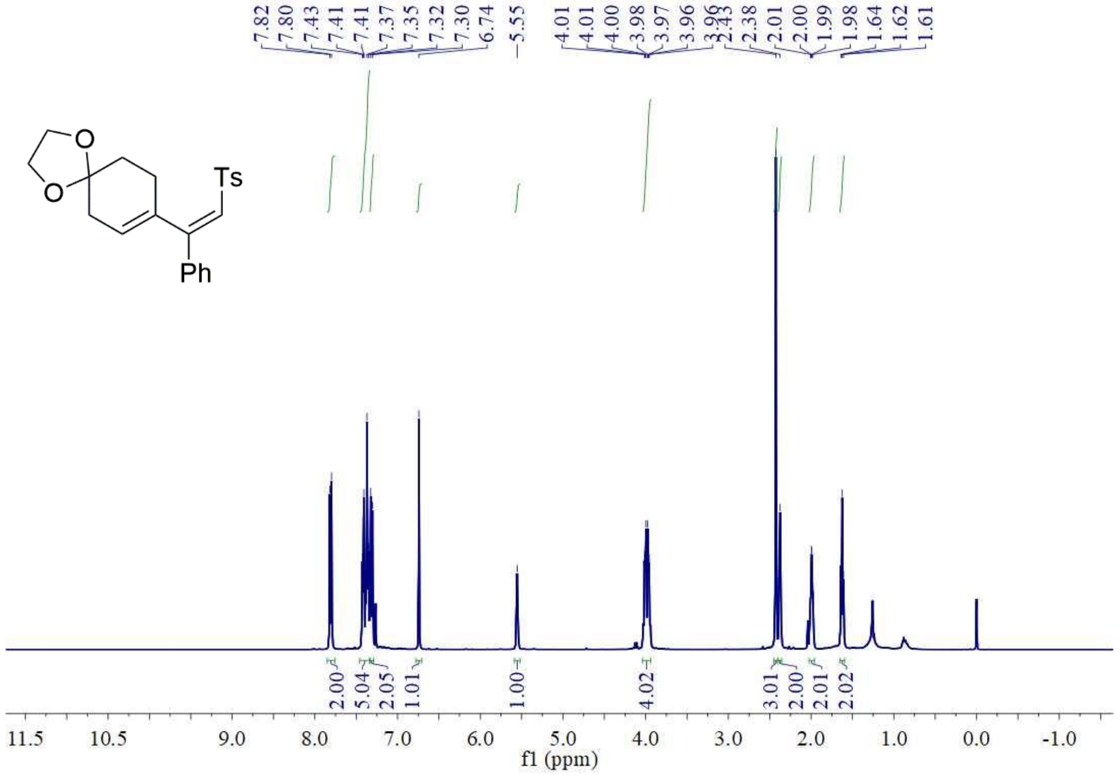

Supplementary Figure 32: ^{1}H NMR of 6a (400 MHz, $CDCl_3$).

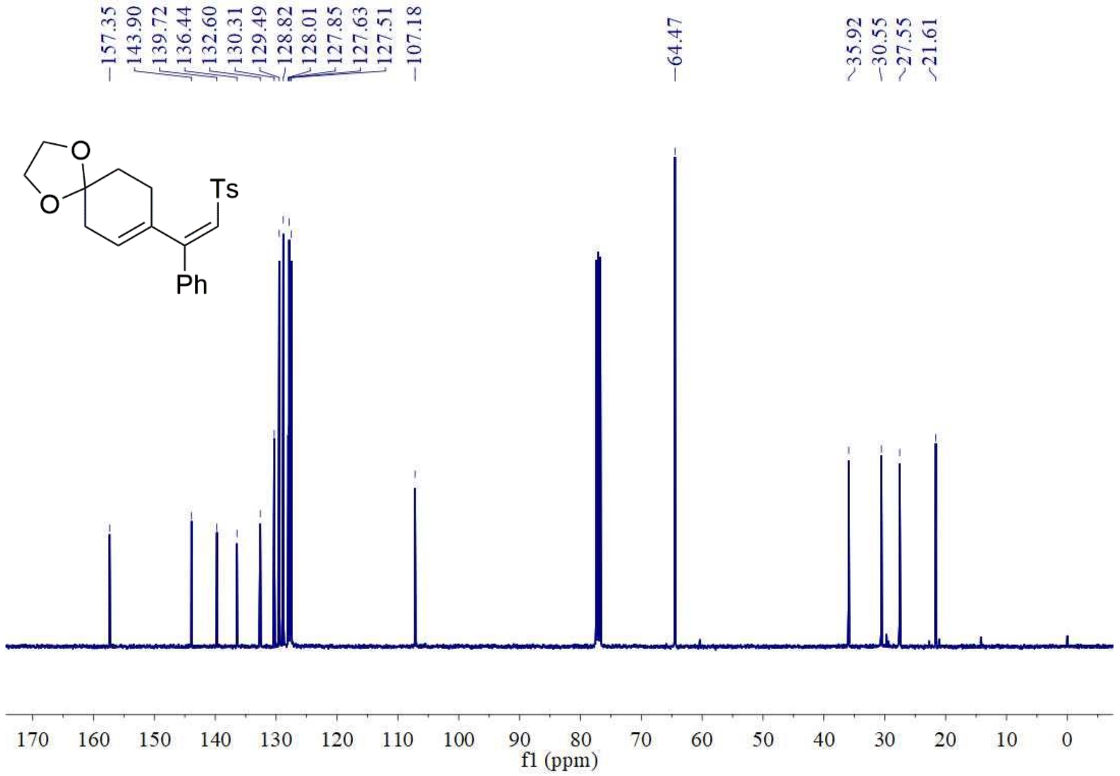

Supplementary Figure 33: ^{13}C NMR of 6a (100 MHz, $CDCl_3$).

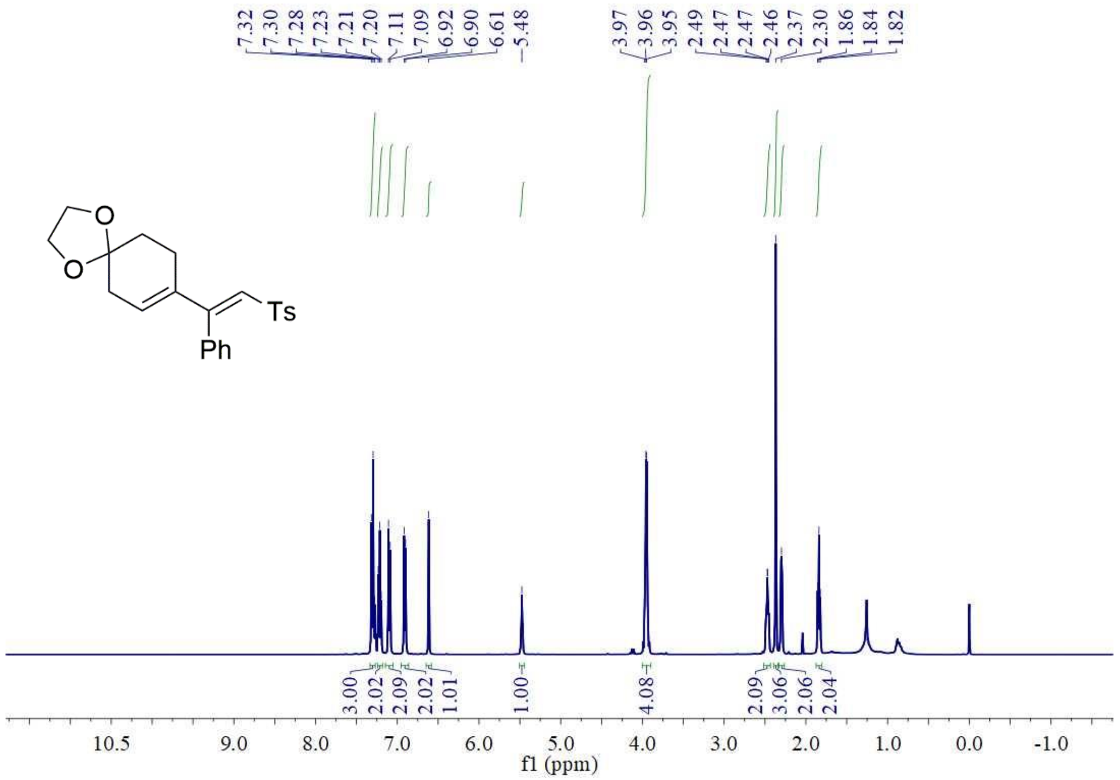

Supplementary Figure 34: ^{1}H NMR of 6b (400 MHz, $CDCl_3$).

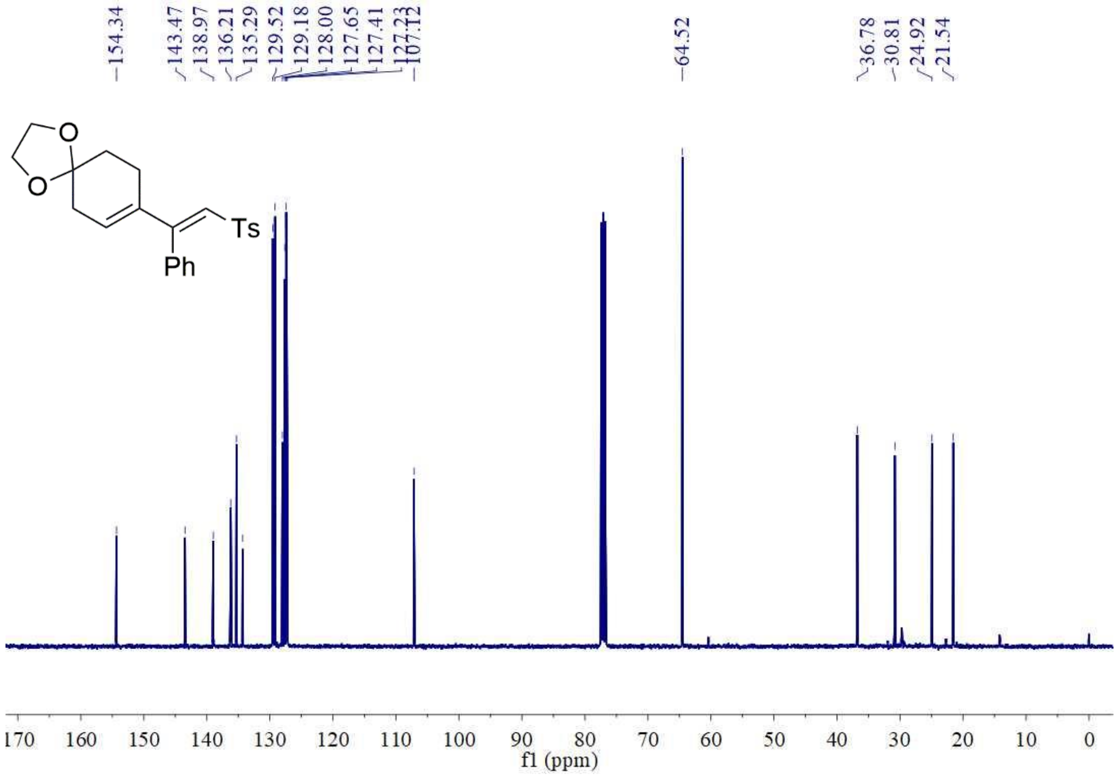

Supplementary Figure 35: ^{13}C NMR of 6b (100 MHz, $CDCl_3$).

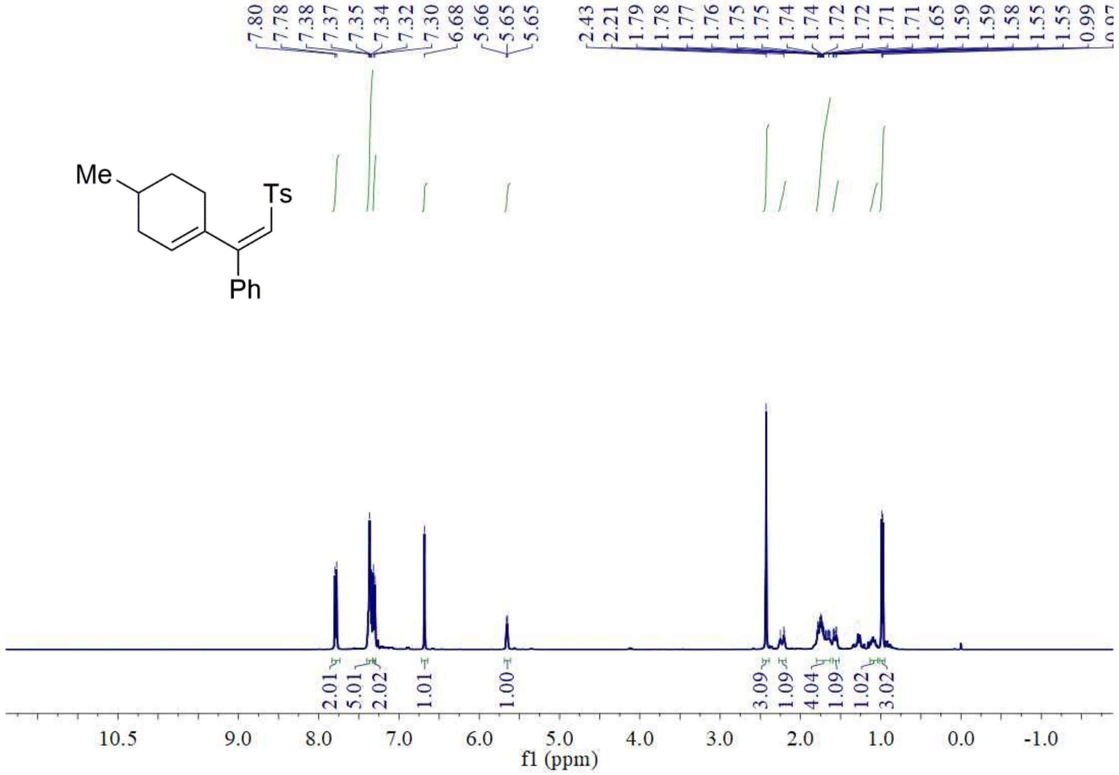

Supplementary Figure 36: ^{1}H NMR of 7a (400 MHz, $CDCl_3$).

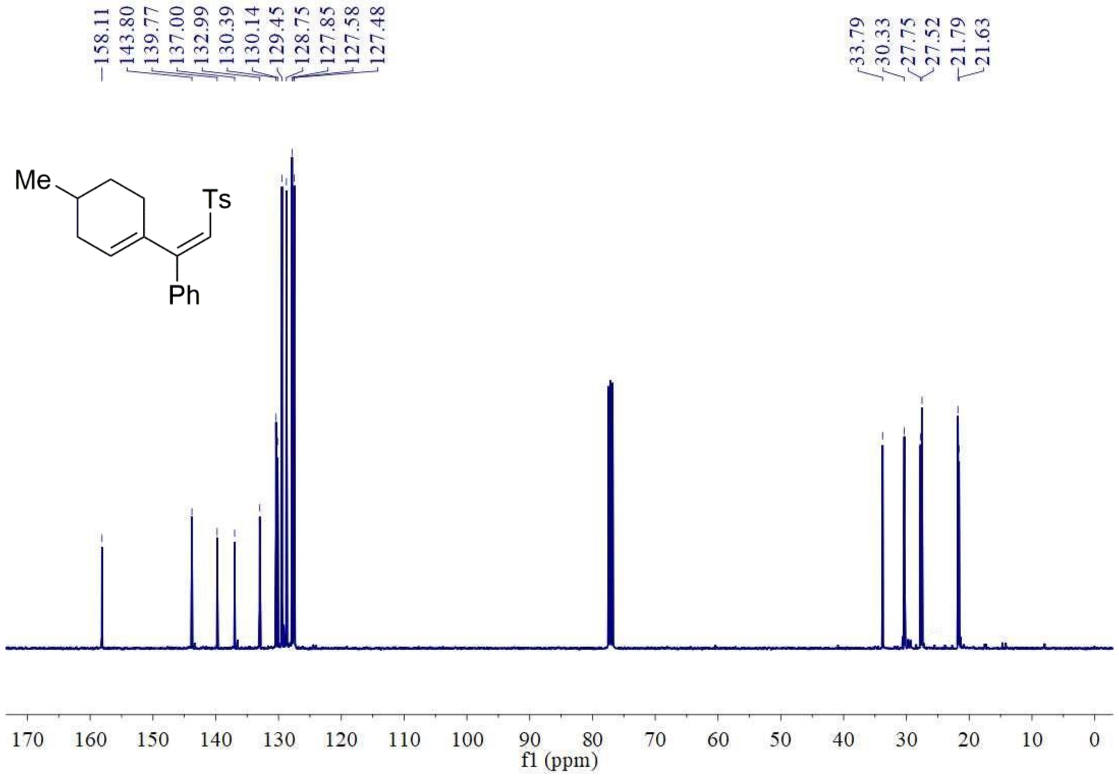

Supplementary Figure 37: ^{13}C NMR of 7a (100 MHz, $CDCl_3$).

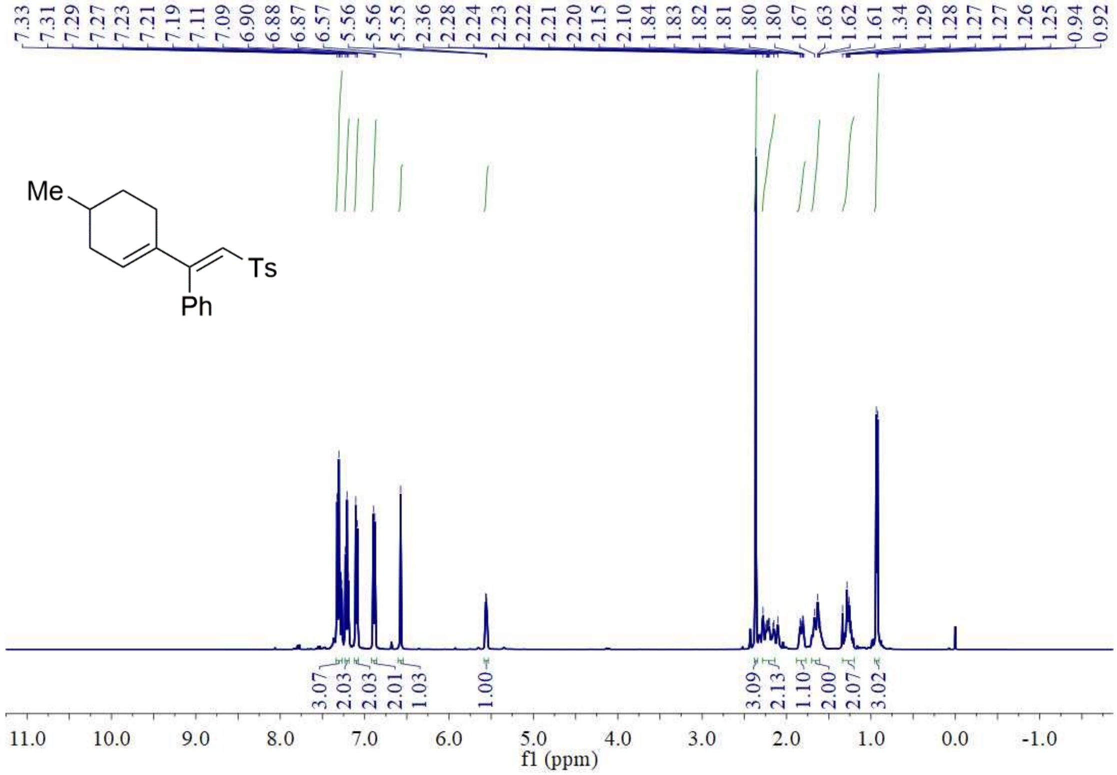

**Supplementary Figure 38: **

Supplementary Figure 38: ^{1}H NMR of 7b (400 MHz, $CDCl_3$).

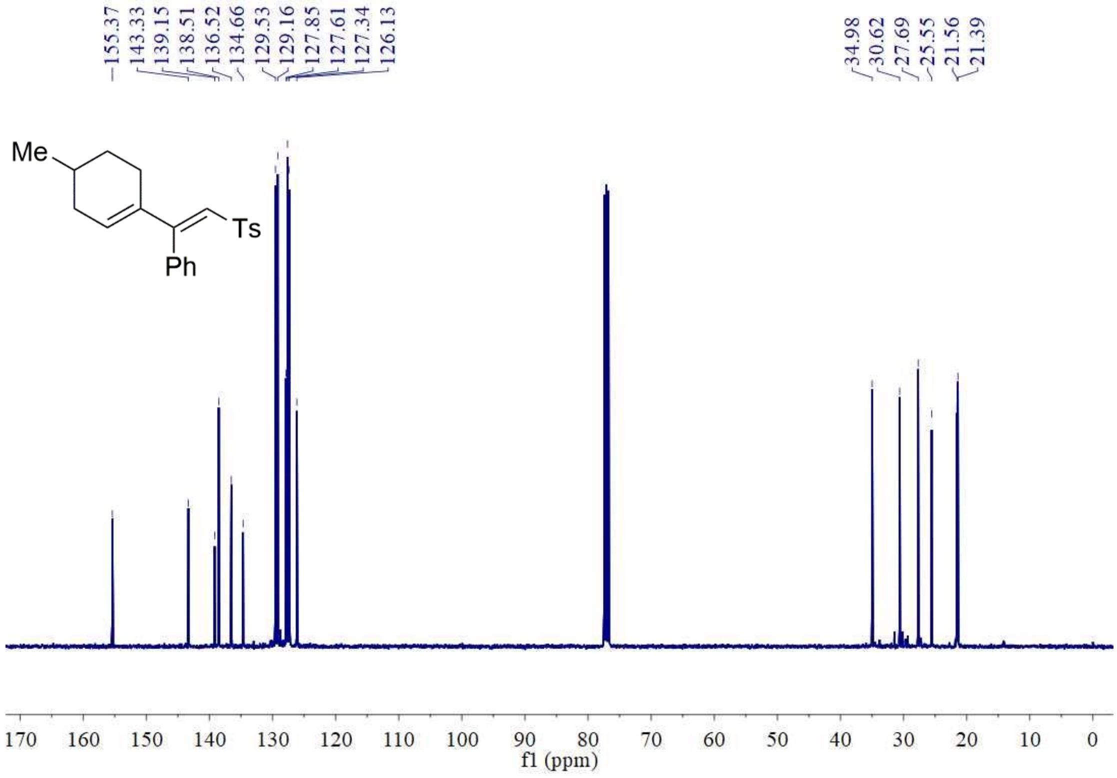

Supplementary Figure 39: ^{13}C NMR of 7b (100 MHz, $CDCl_3$).

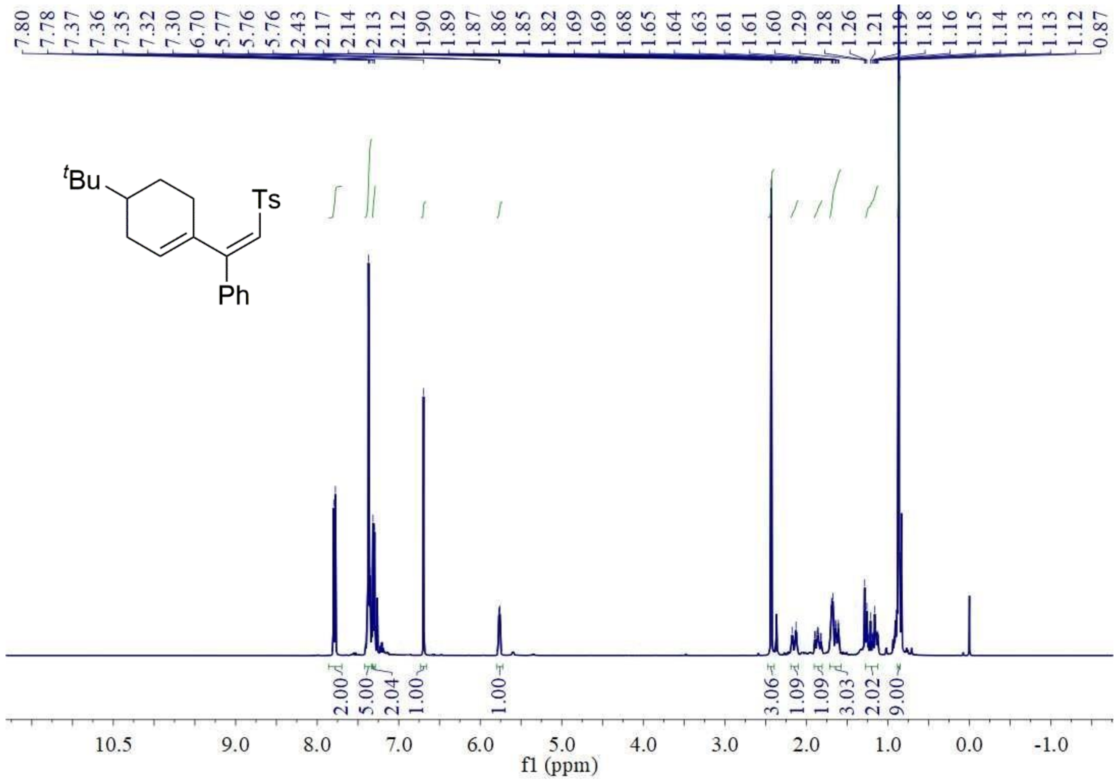

Supplementary Figure 40: ^{1}H NMR of 8a (400 MHz, $CDCl_3$).

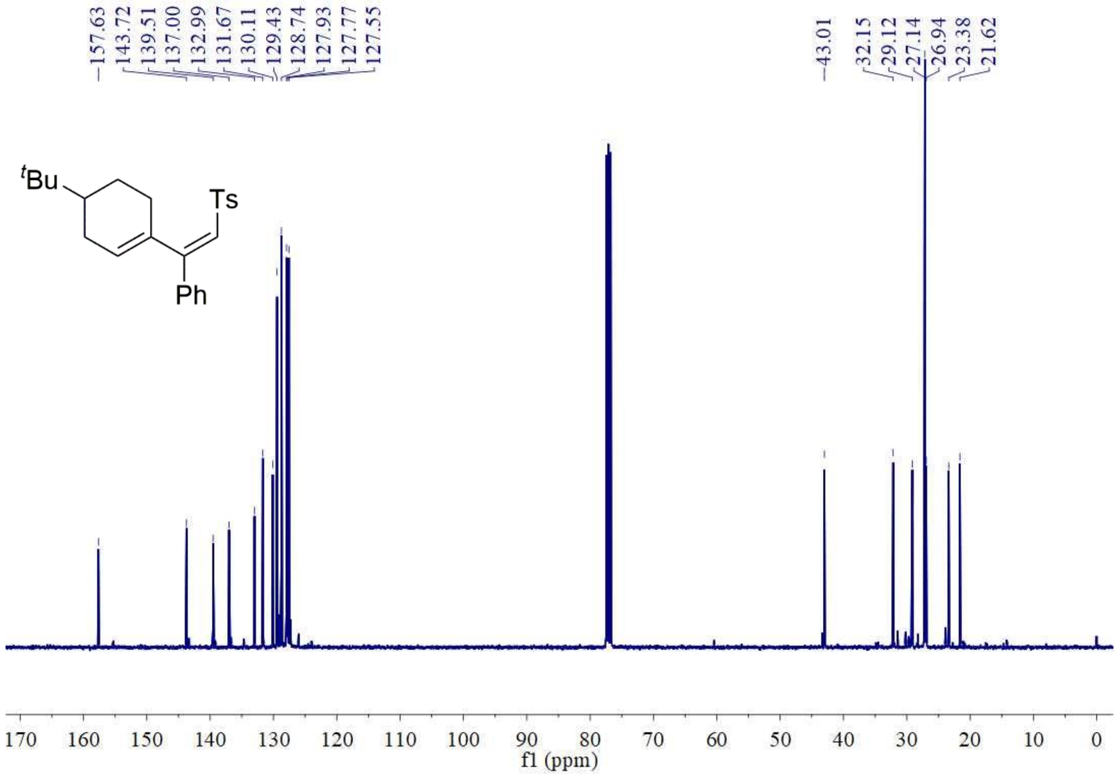

Supplementary Figure 41: ^{13}C NMR of 8a (100 MHz, $CDCl_3$).

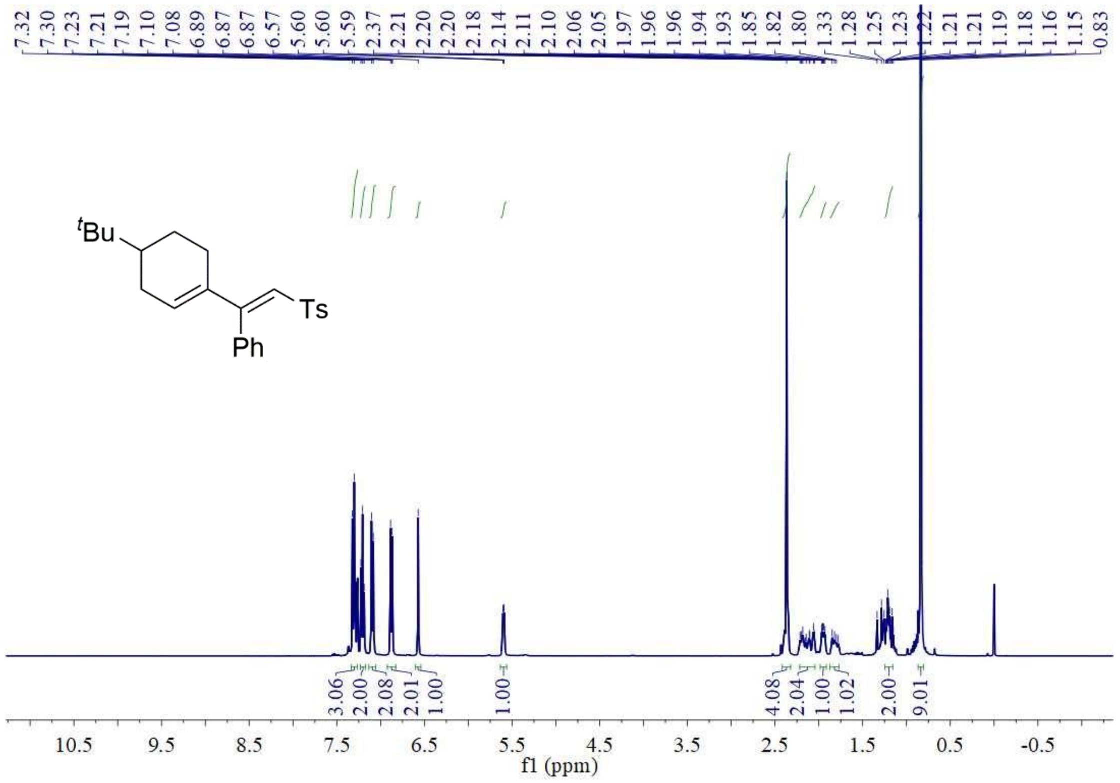

Supplementary Figure 42: ^{1}H NMR of 8b (400 MHz, $CDCl_3$).

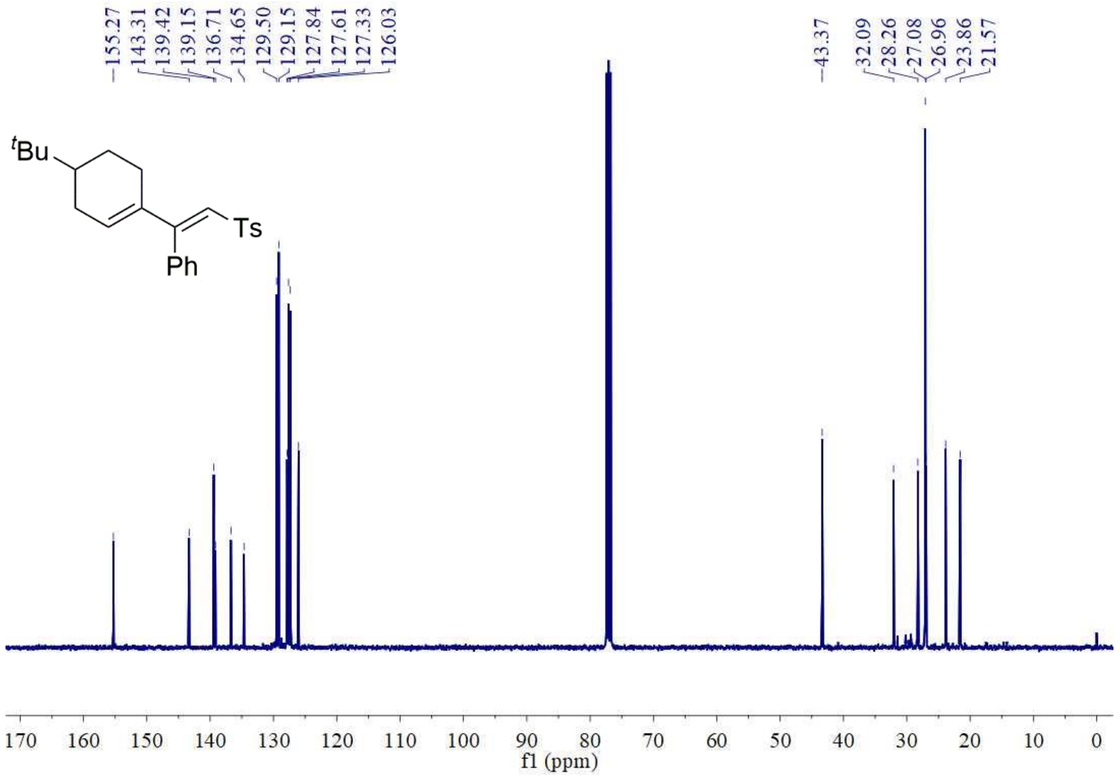

Supplementary Figure 43: ^{13}C NMR of 8b (100 MHz, $CDCl_3$).

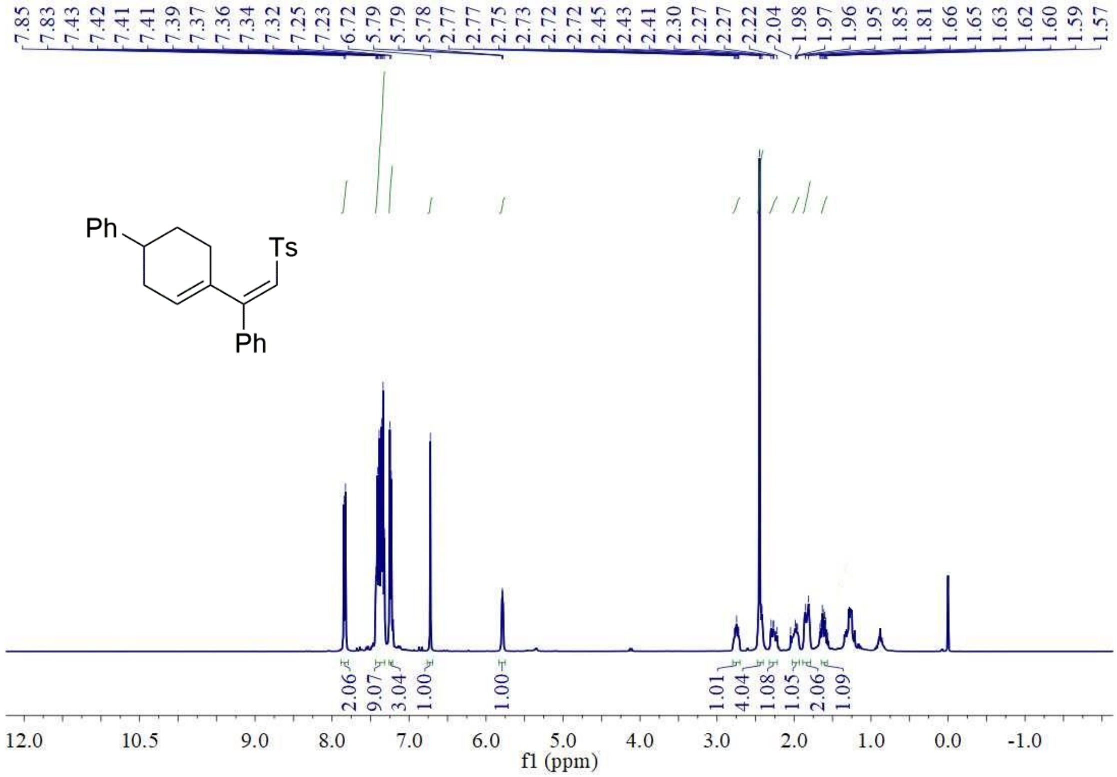

Supplementary Figure 44: ¹H NMR of 9a (400 MHz, $CDCl_3$).

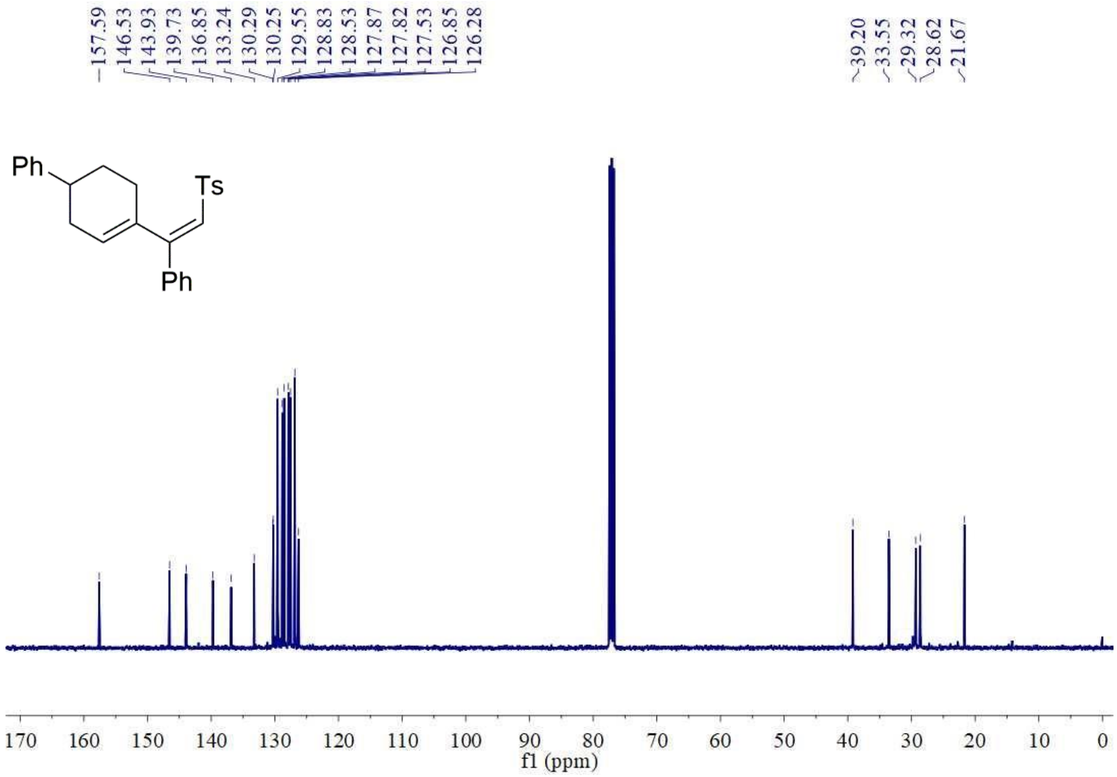

Supplementary Figure 45: ¹³C NMR of 9a (100 MHz, $CDCl_3$).

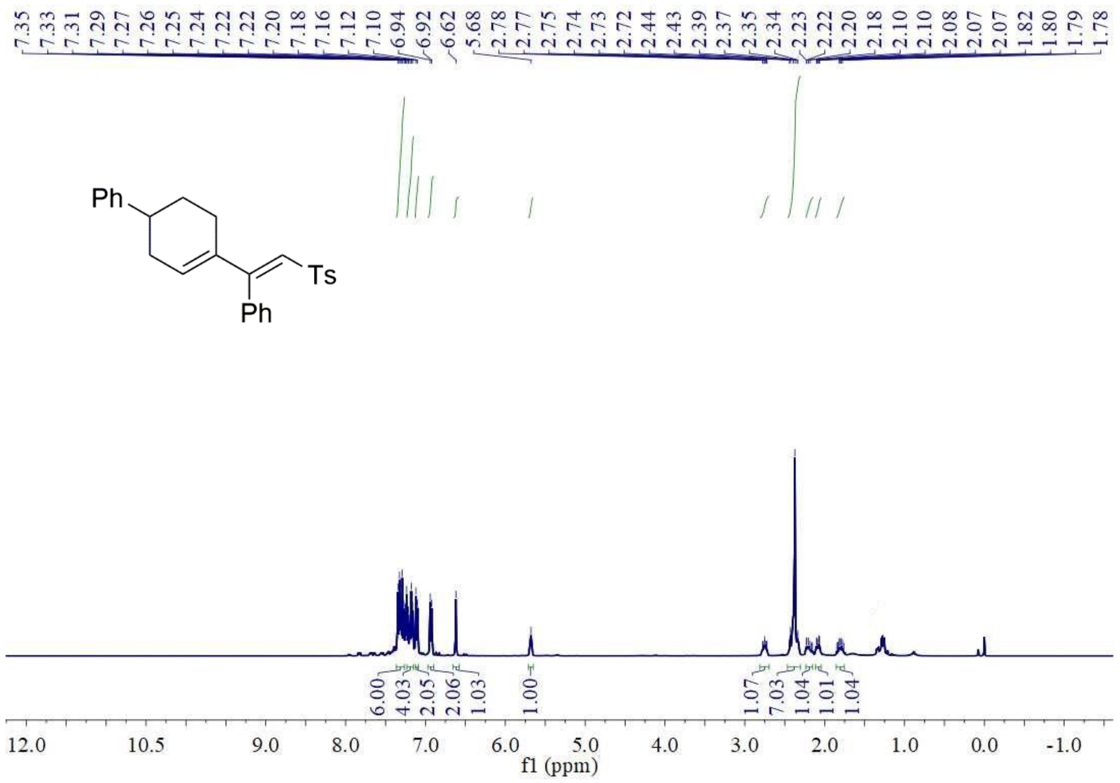

Supplementary Figure 46: ^{1}H NMR of 9b (400 MHz, $CDCl_3$).

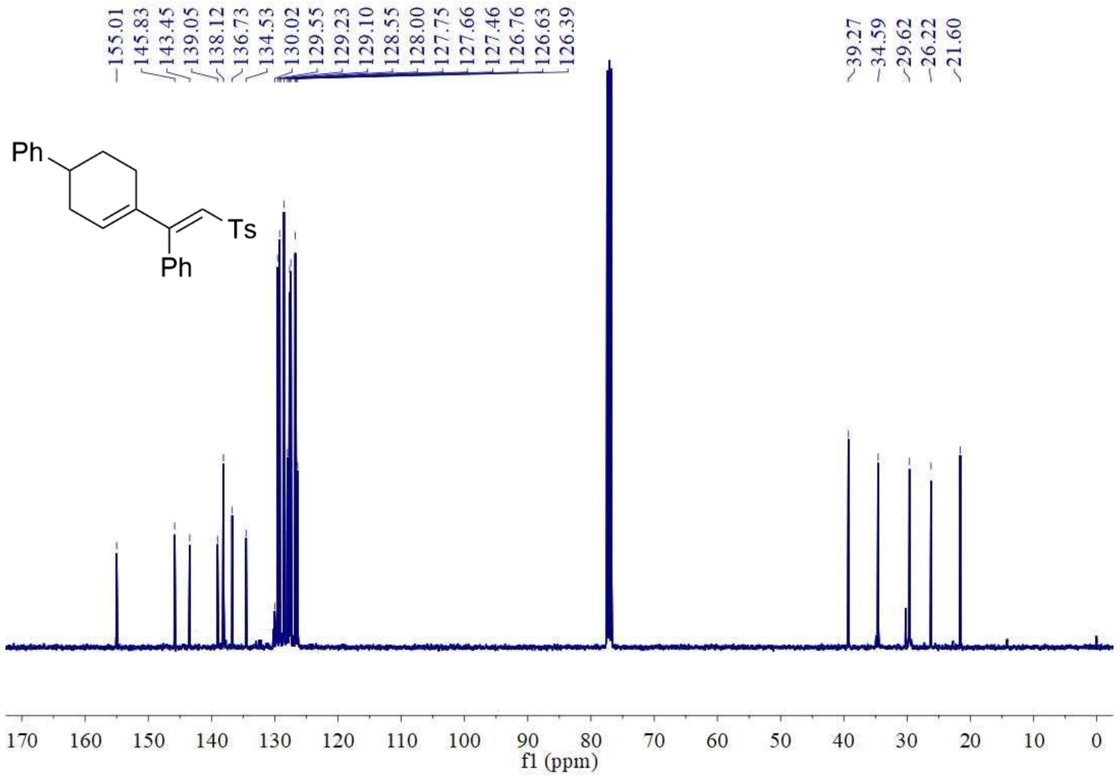

Supplementary Figure 47: ^{13}C NMR of 9b (100 MHz, $CDCl_3$).

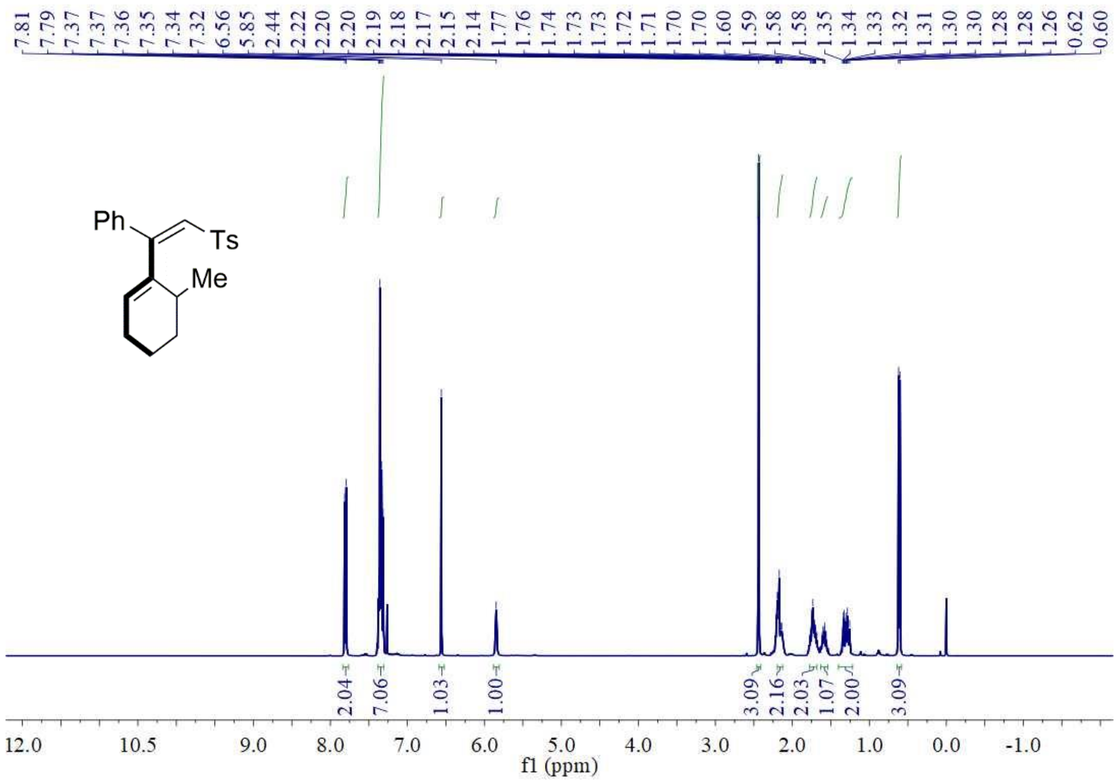

Supplementary Figure 48: ^{1}H NMR of 10a (400 MHz, $CDCl_3$).

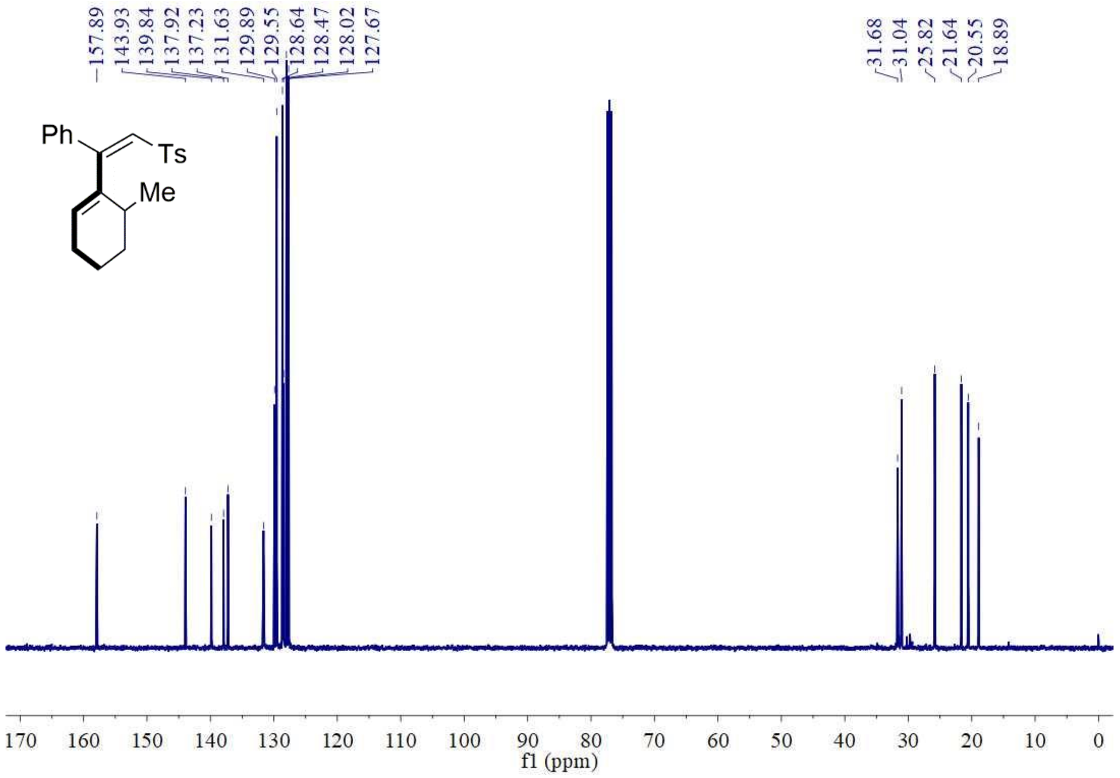

Supplementary Figure 49: ^{13}C NMR of 10a (100 MHz, $CDCl_3$).

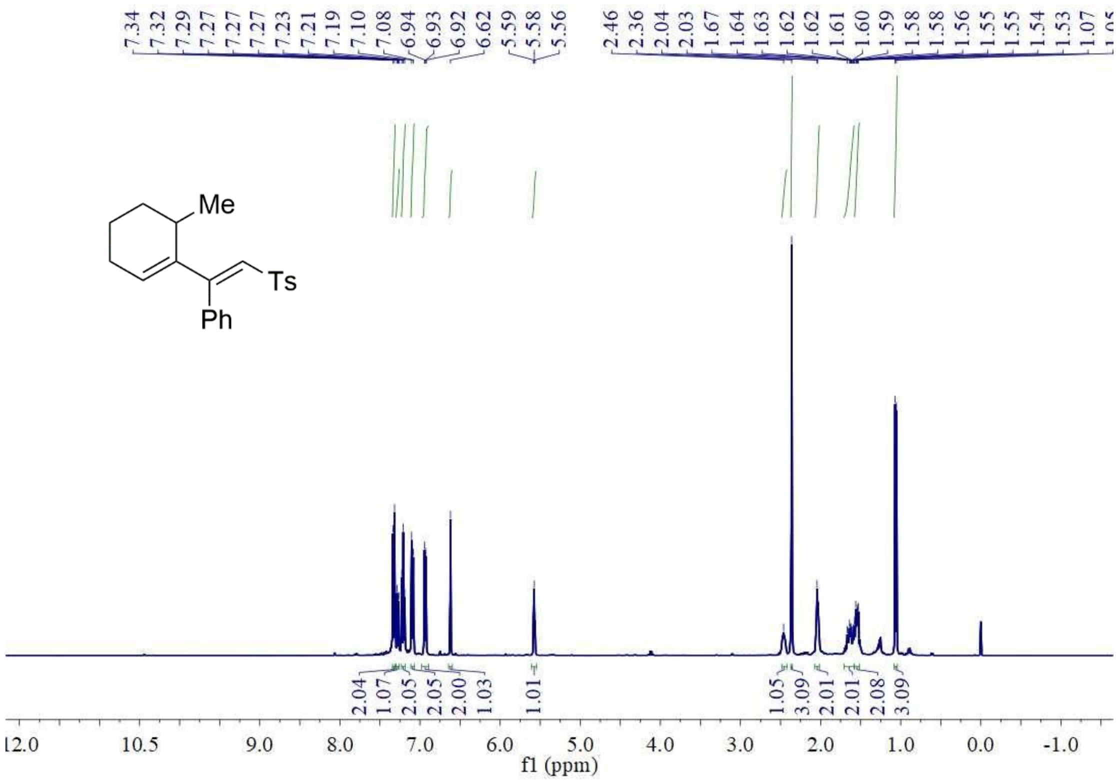

Supplementary Figure 50: ^{1}H NMR of 10b (400 MHz, $CDCl_3$).

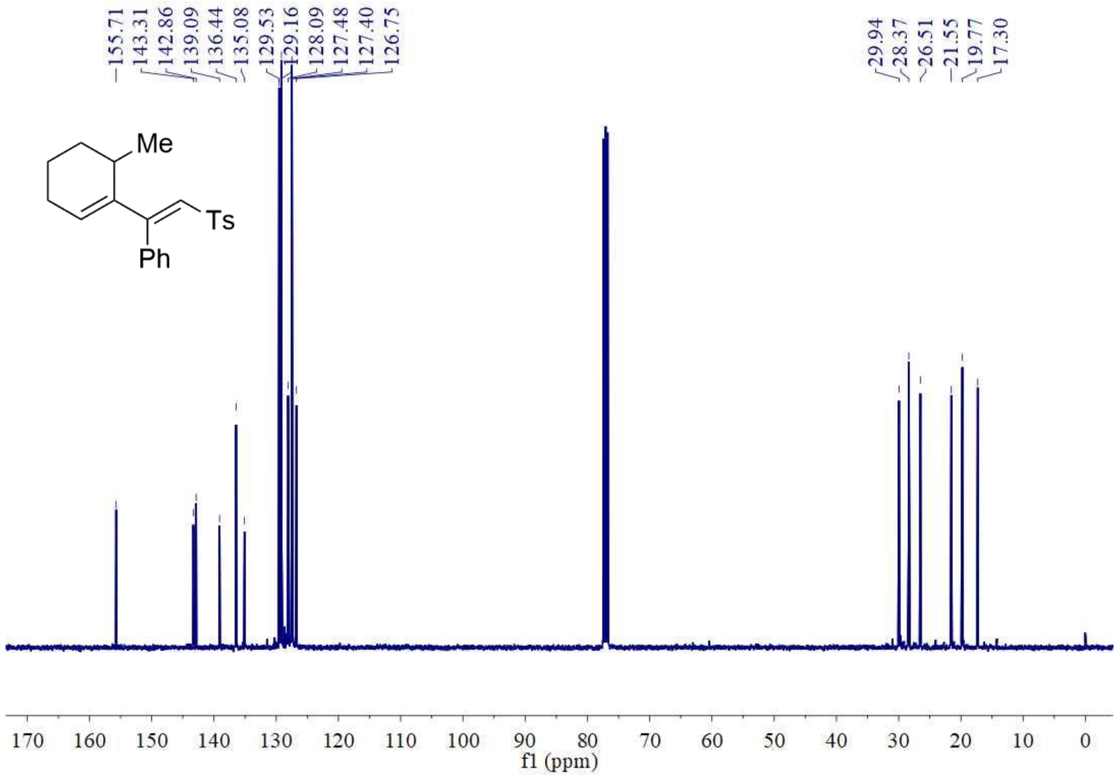

Supplementary Figure 51: ^{13}C NMR of 10b (100 MHz, $CDCl_3$).

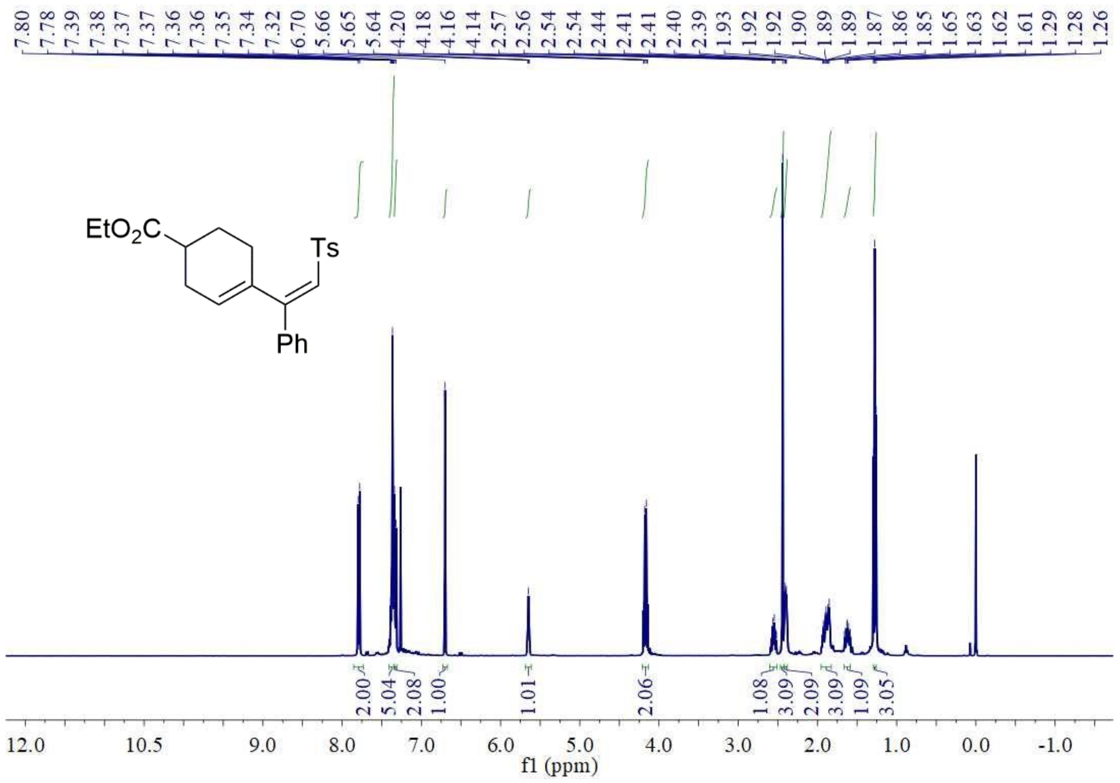

Supplementary Figure 52: ^{1}H NMR of 11a (400 MHz, $CDCl_3$).

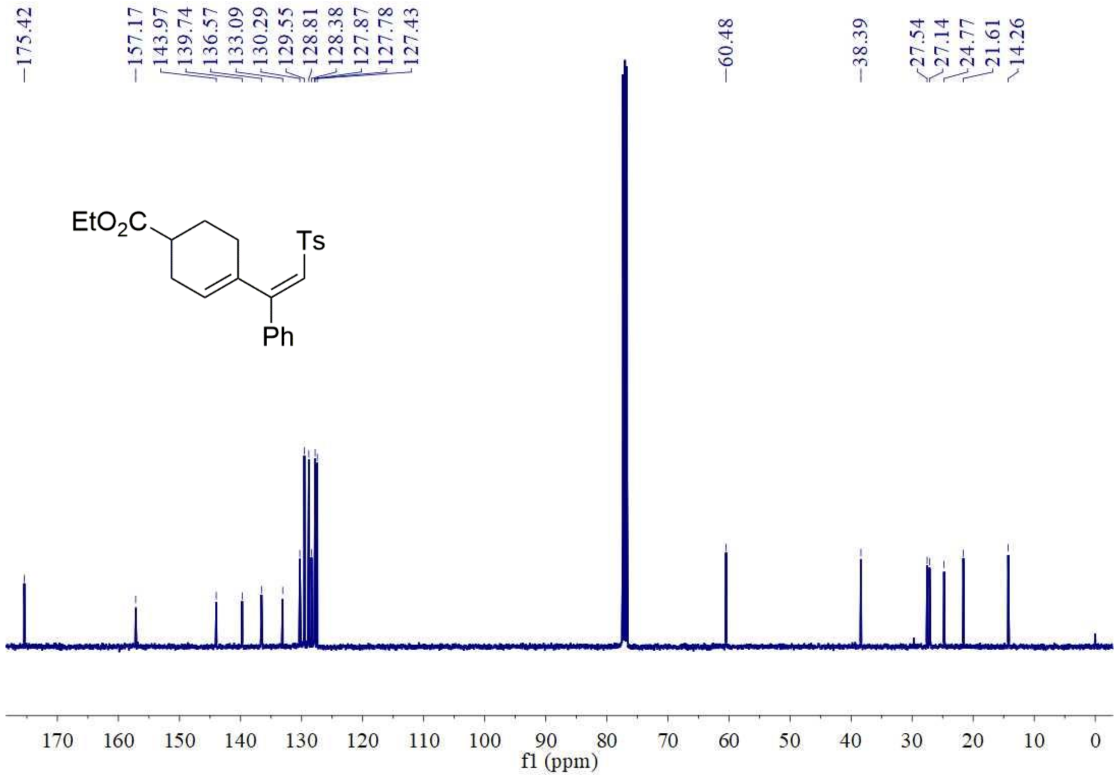

Supplementary Figure 53: ^{13}C NMR of 11a (100 MHz, $CDCl_3$).

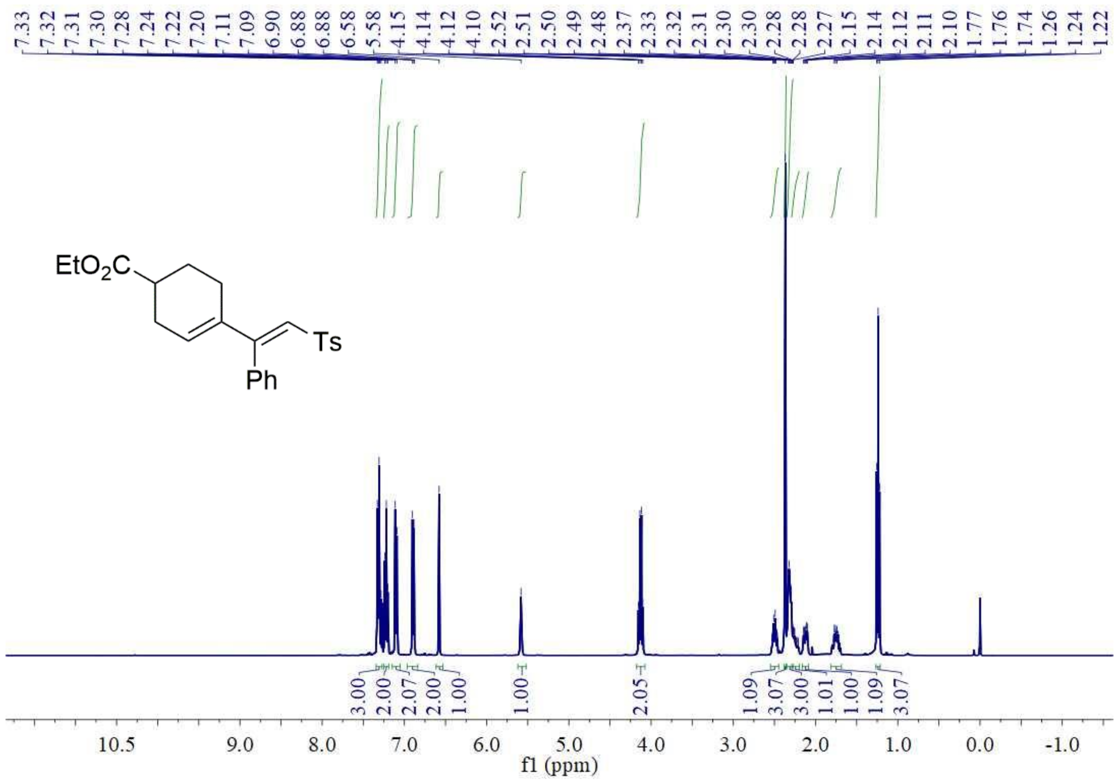

Supplementary Figure 54: ^{1}H NMR of 11b (400 MHz, $CDCl_3$).

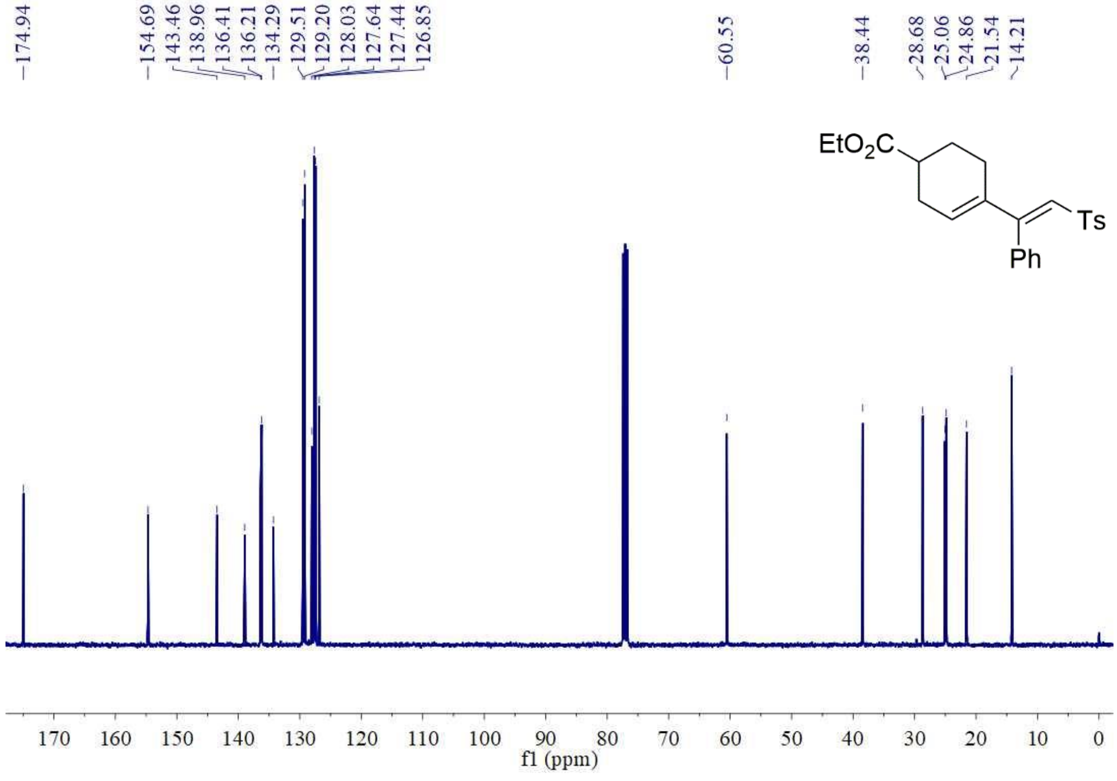

Supplementary Figure 55: ^{13}C NMR of 11b (100 MHz, $CDCl_3$).

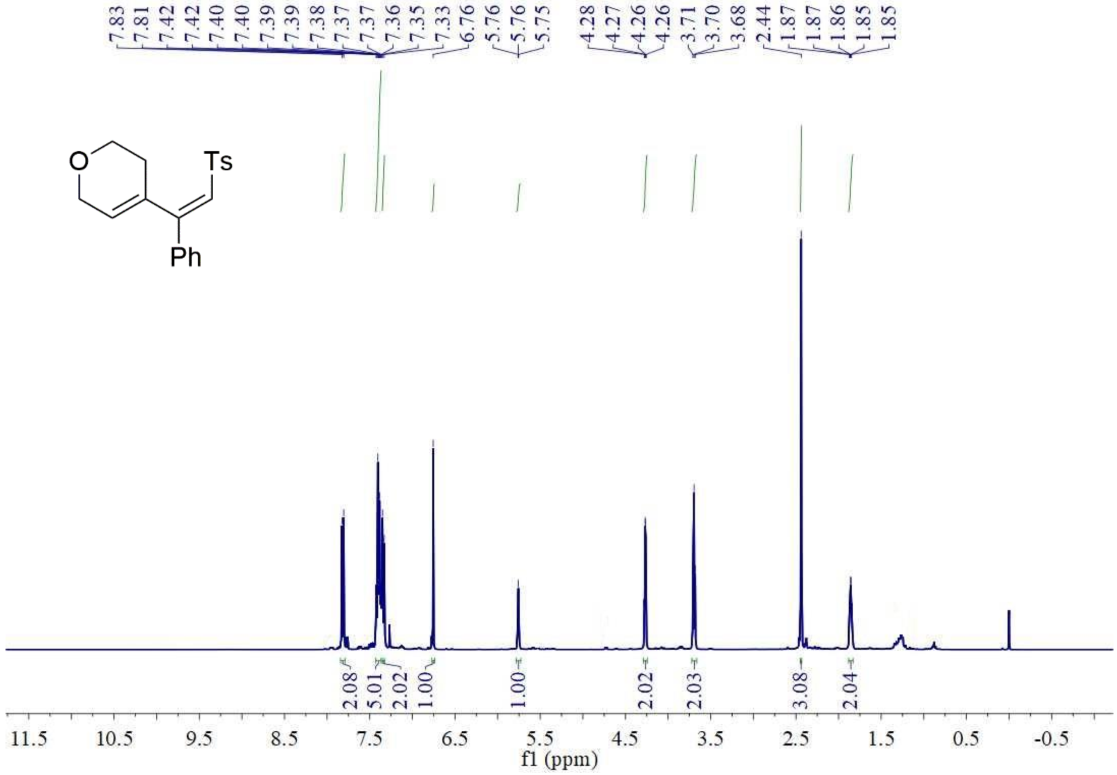

Supplementary Figure 56: ^{1}H NMR of 12a (400 MHz, $CDCl_3$).

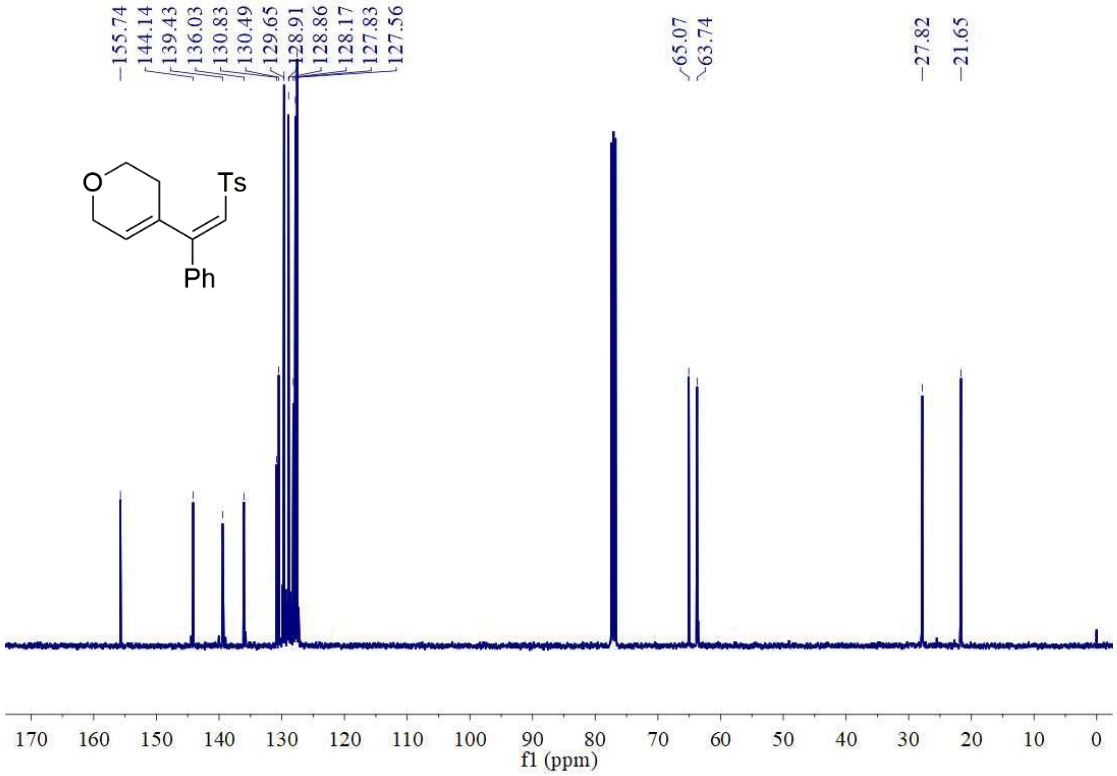

Supplementary Figure 57: ^{13}C NMR of 12a (100 MHz, $CDCl_3$).

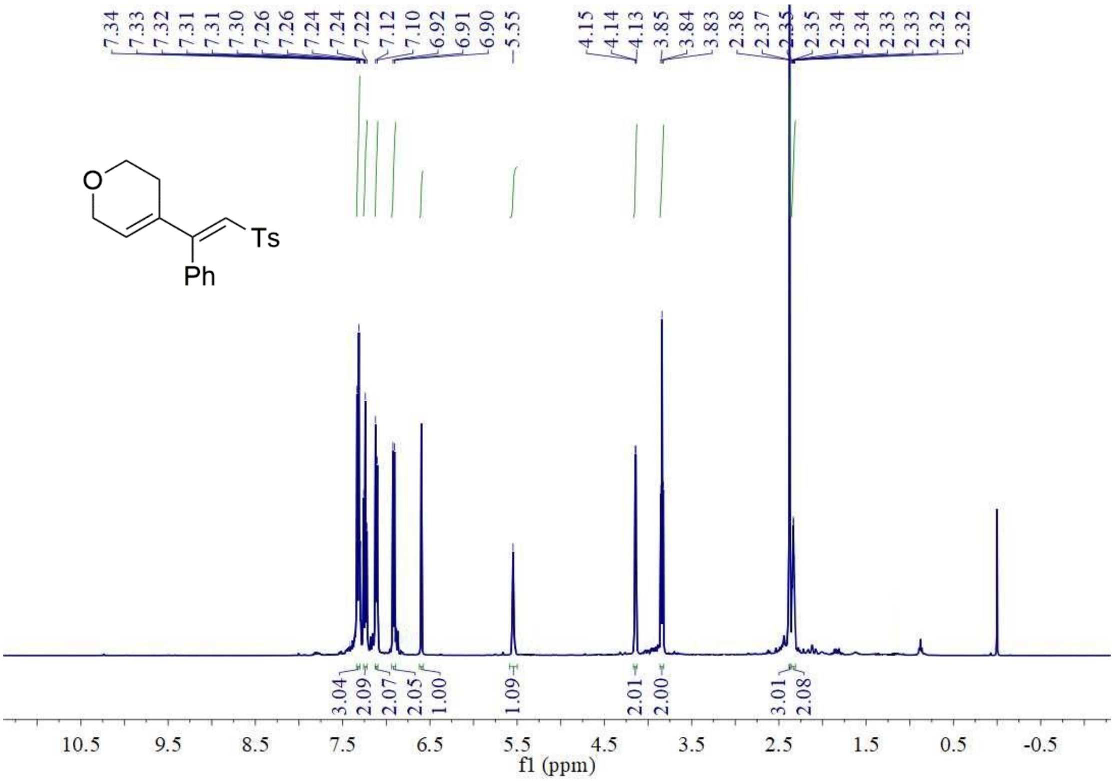

Supplementary Figure 58: ^{1}H NMR of 12b (400 MHz, $CDCl_3$).

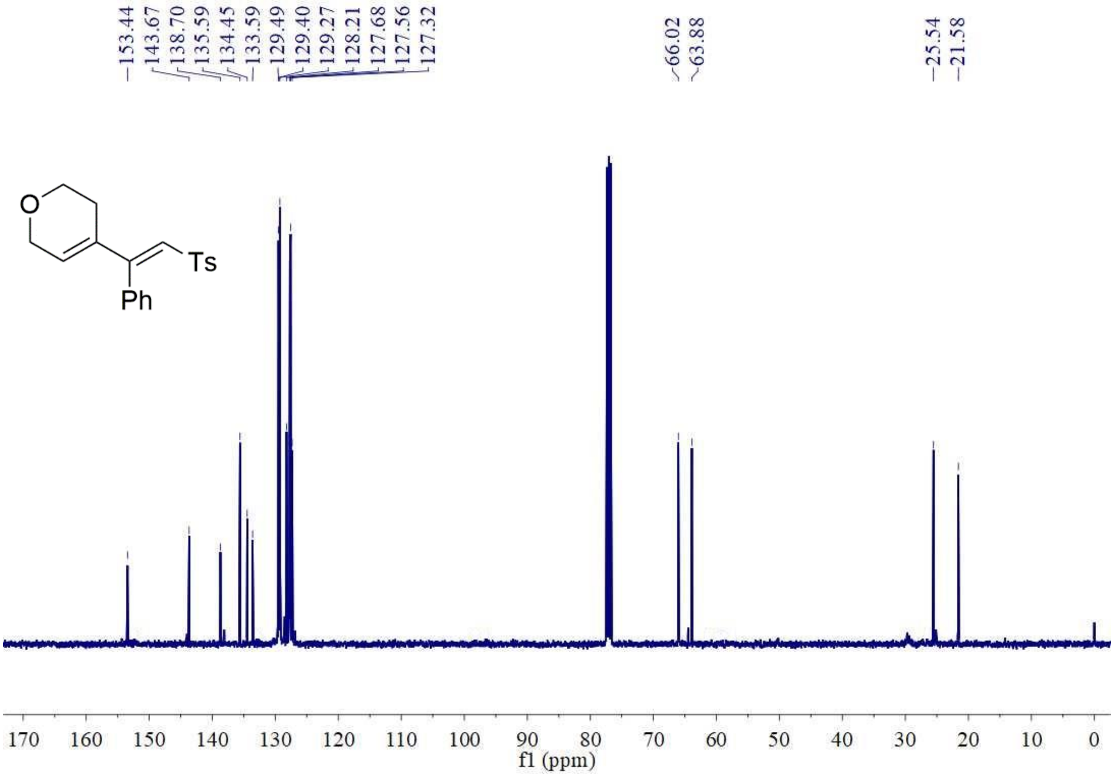

Supplementary Figure 59: ^{13}C NMR of 12b (100 MHz, $CDCl_3$).

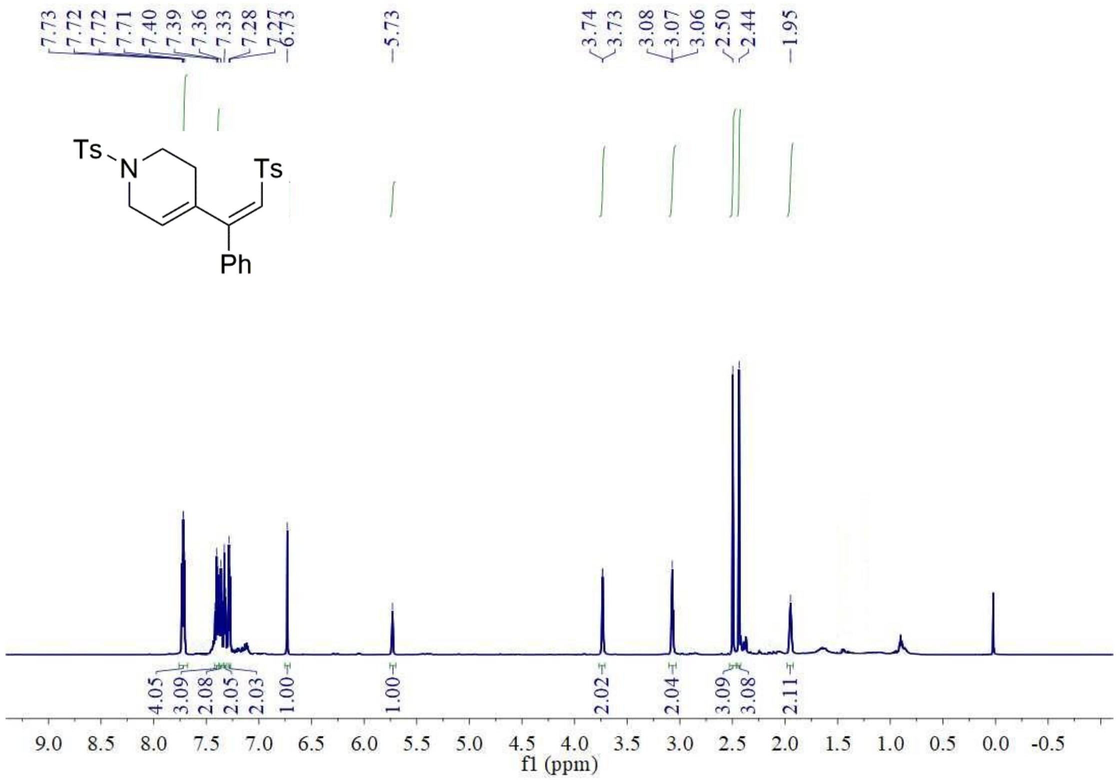

Supplementary Figure 60: ^{1}H NMR of 13a (600 MHz, $CDCl_3$).

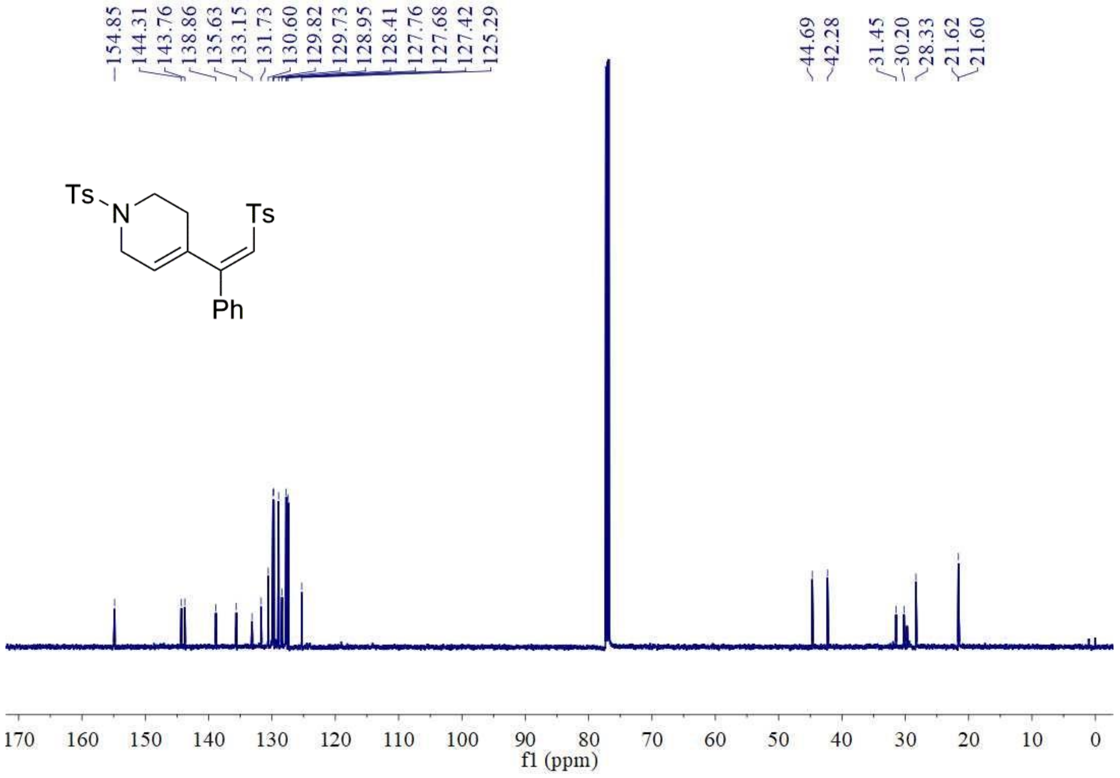

Supplementary Figure 61: ^{13}C NMR of 13a (150 MHz, $CDCl_3$).

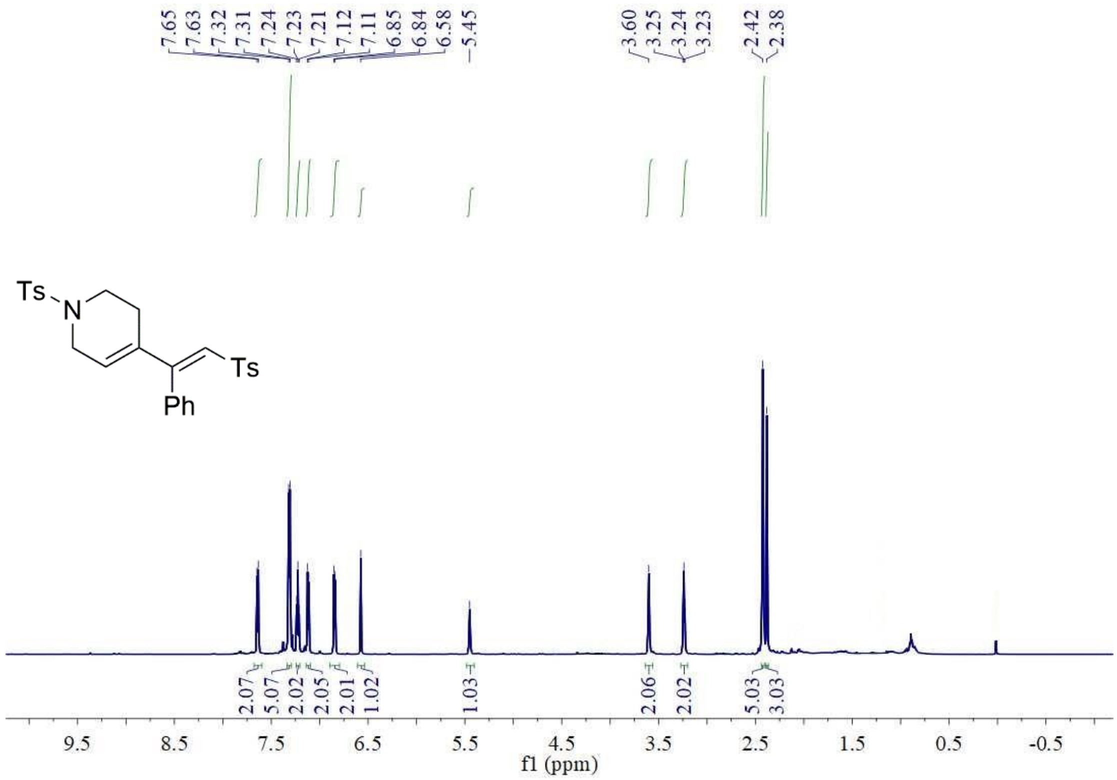

Supplementary Figure 62: ^{1}H NMR of 13b (600 MHz, $CDCl_3$).

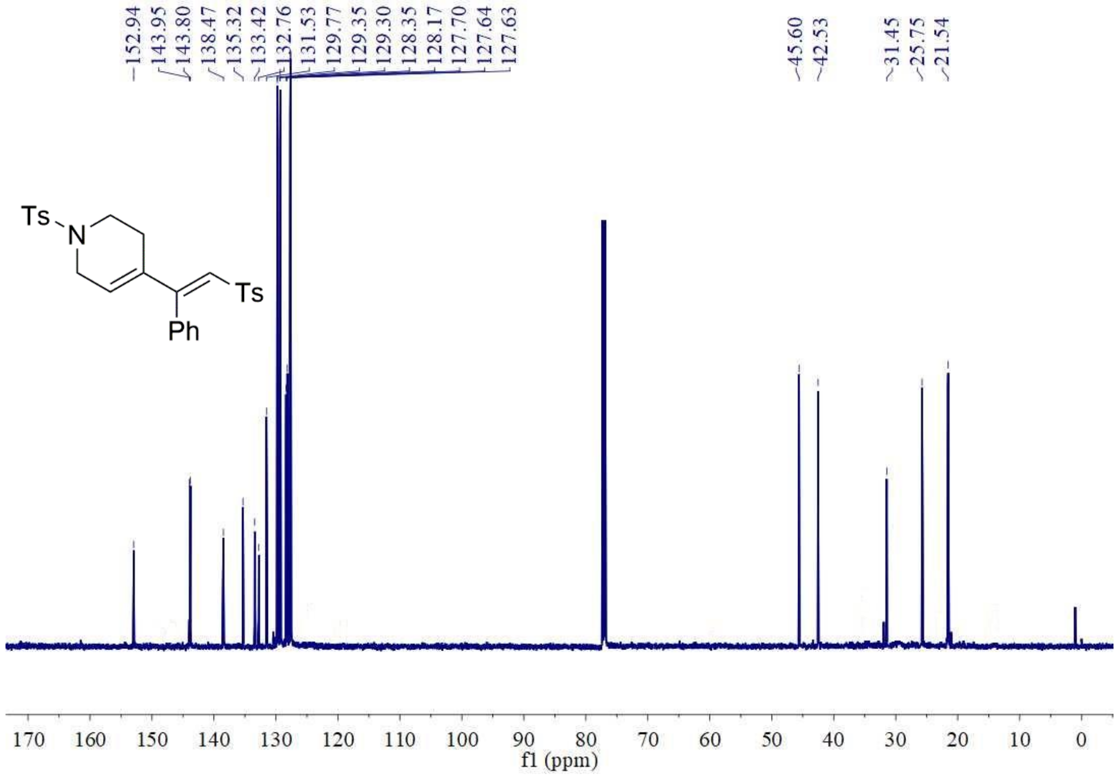

Supplementary Figure 63: ^{13}C NMR of 13b (150 MHz, $CDCl_3$).

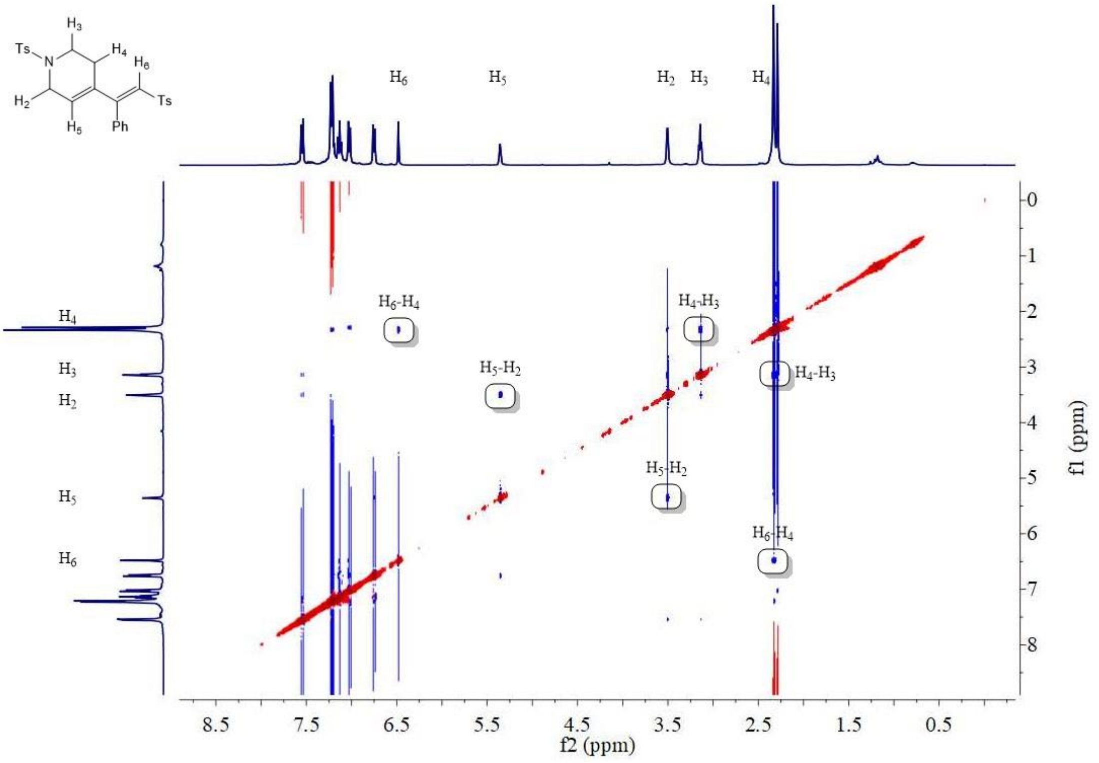

Supplementary Figure 64: NOESY of 13b.

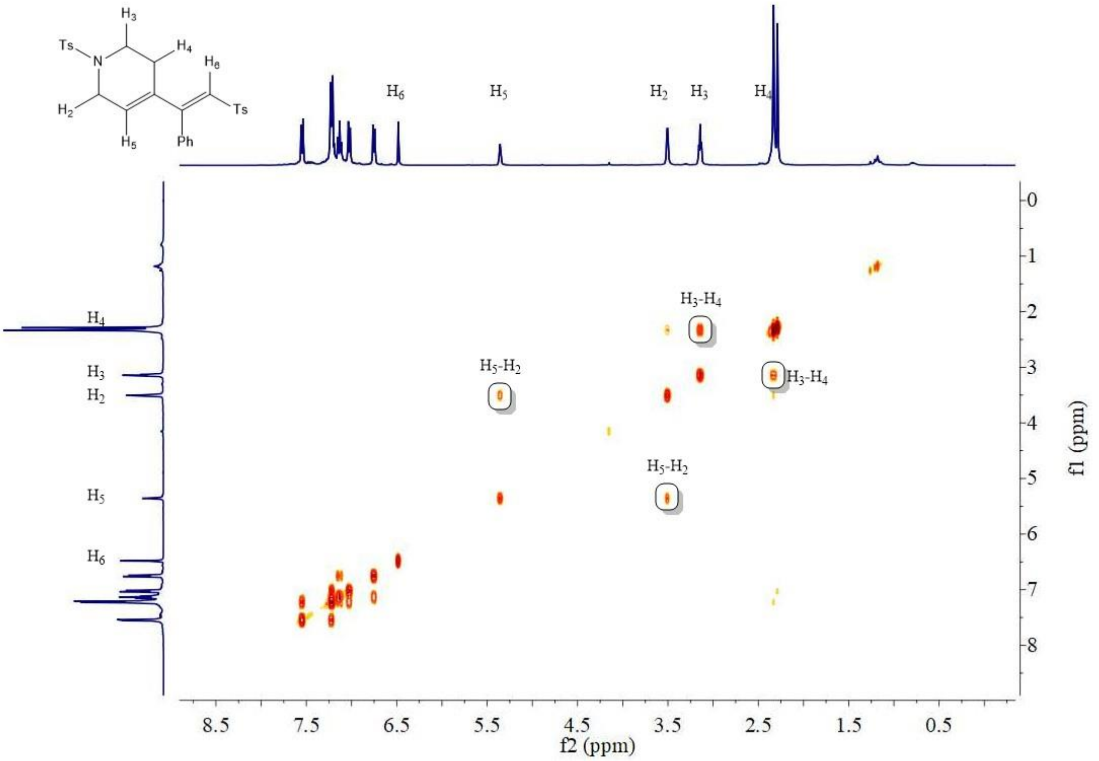

Supplementary Figure 65: COSY of 13b.

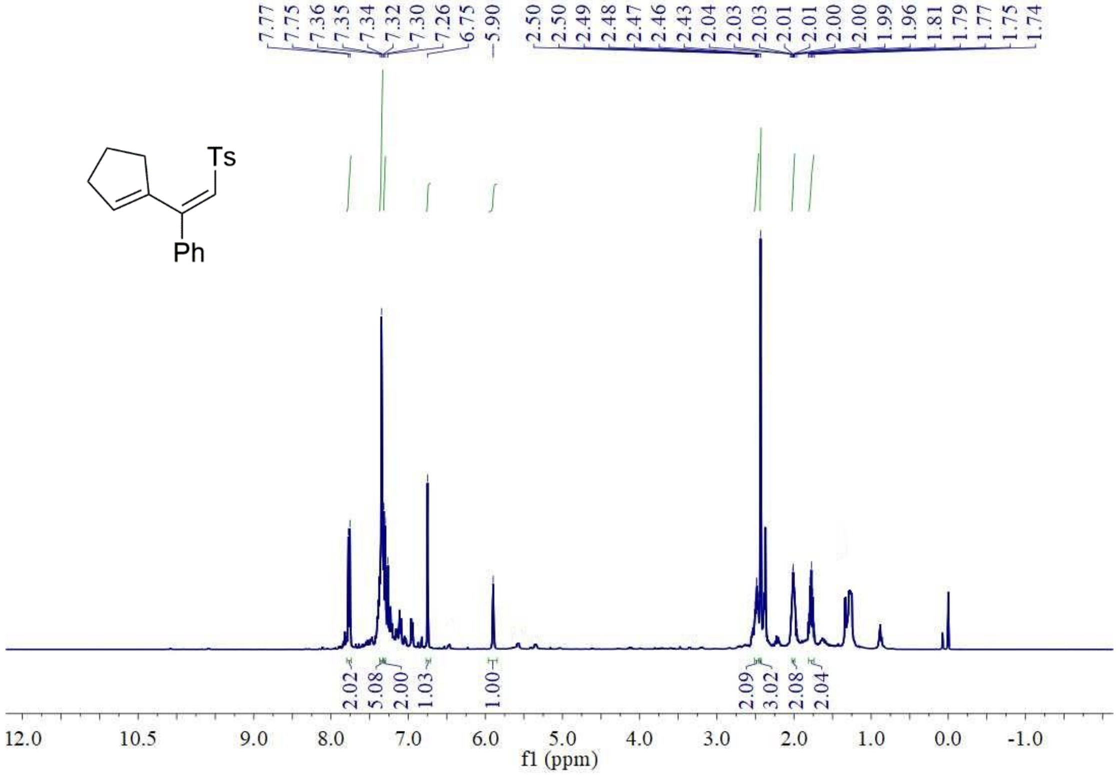

Supplementary Figure 66: ^{1}H NMR of 14a (400 MHz, $CDCl_3$).

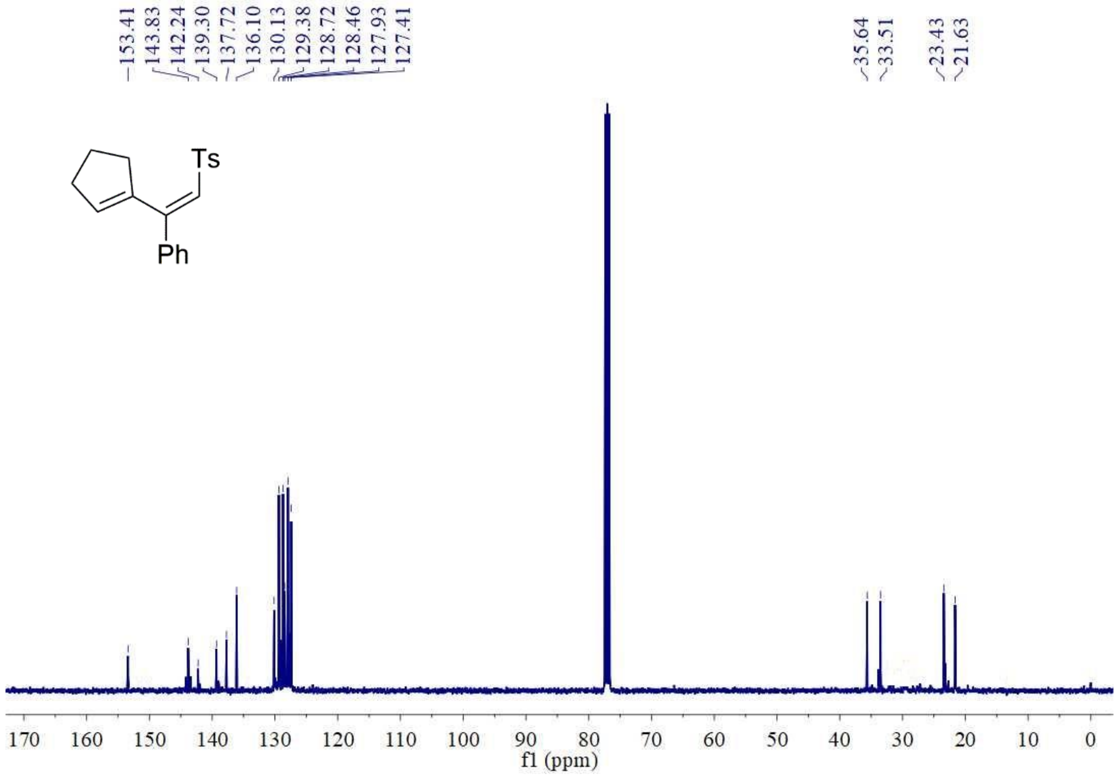

Supplementary Figure 67: ^{13}C NMR of 14a (100 MHz, $CDCl_3$).

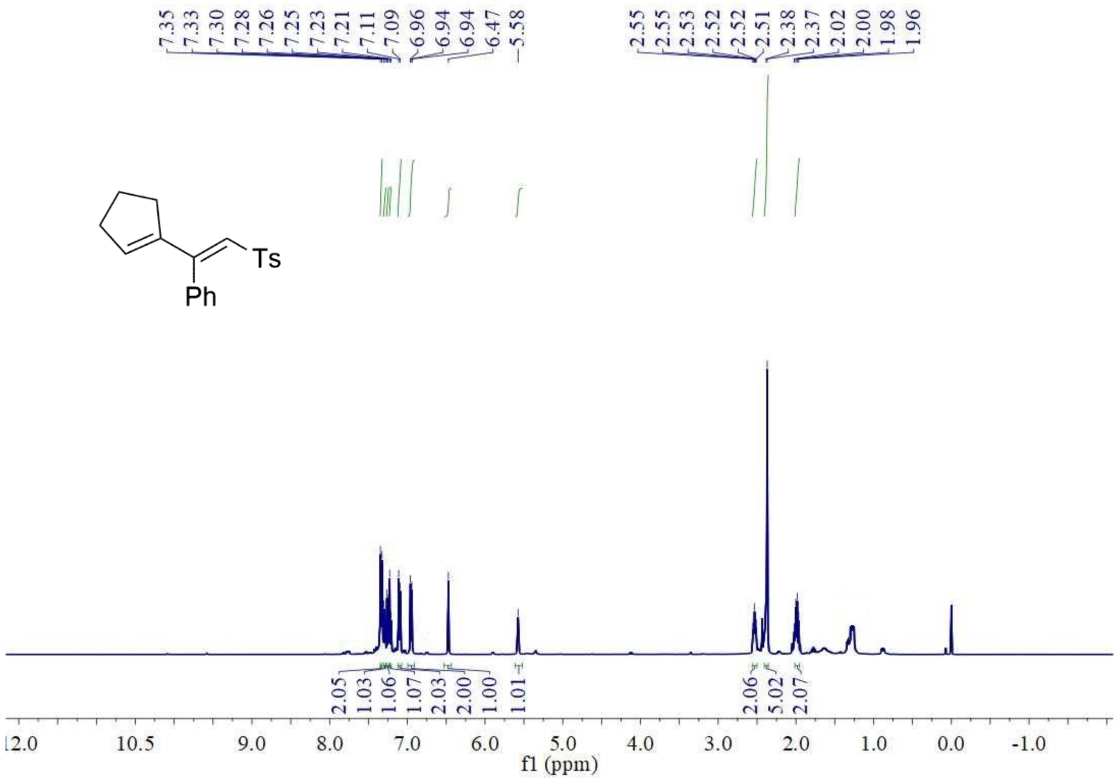

Supplementary Figure 68: ^{1}H NMR of 14b (400 MHz, $CDCl_3$).

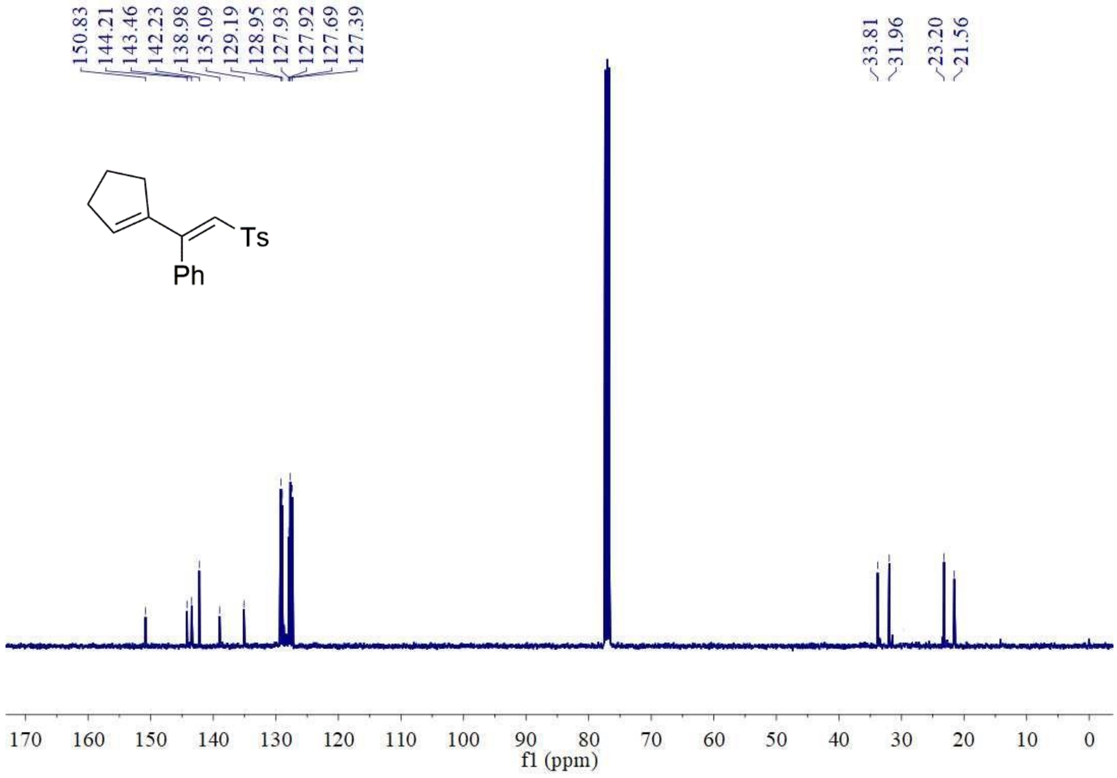

Supplementary Figure 69: ^{13}C NMR of 14b (100 MHz, $CDCl_3$).

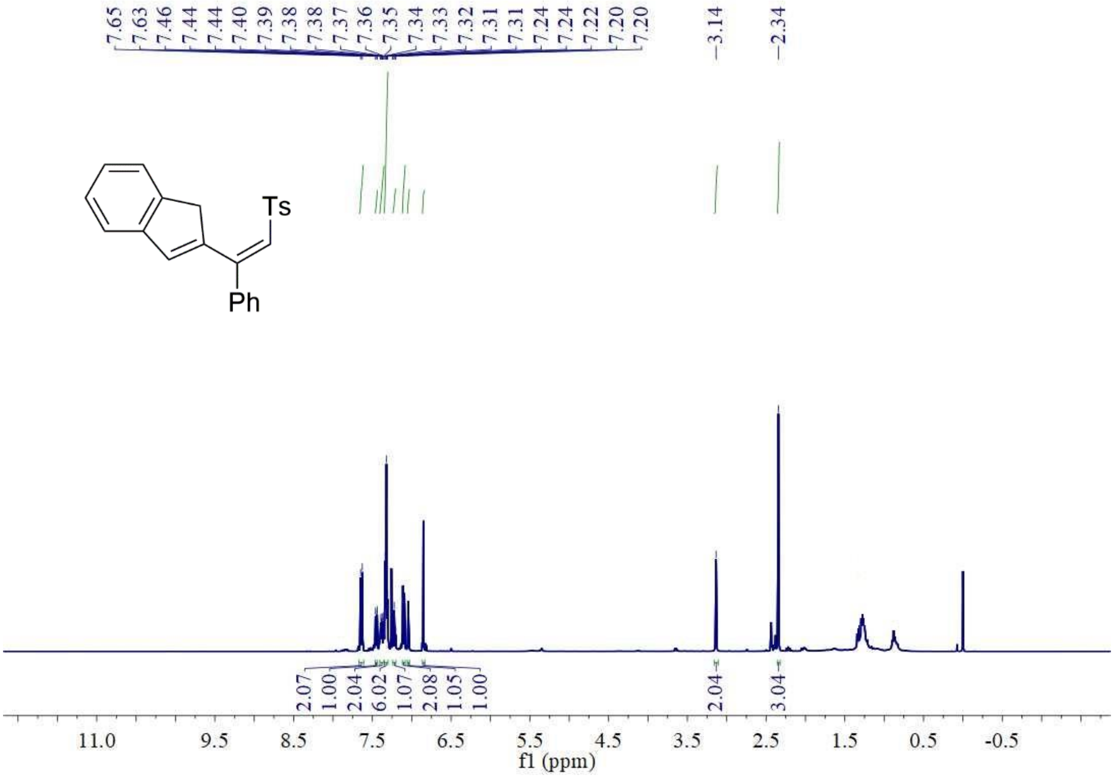

Supplementary Figure 70: ^{1}H NMR of 15a (400 MHz, $CDCl_3$).

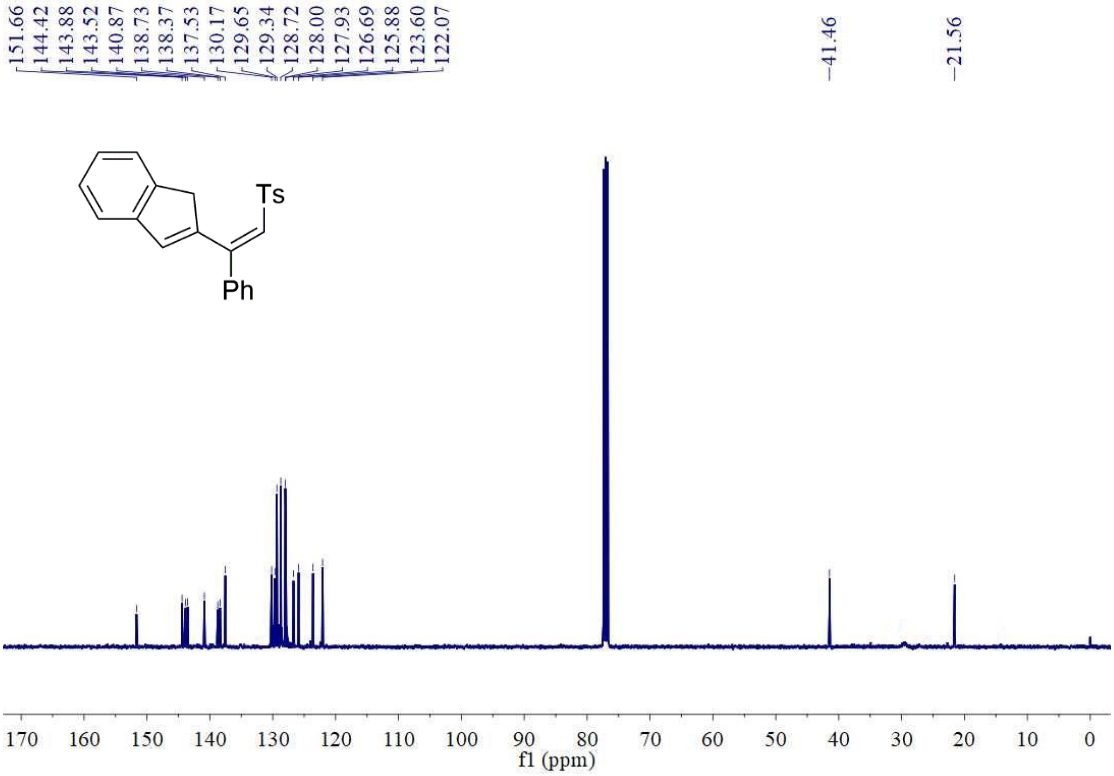

Supplementary Figure 71: ^{13}C NMR of 15a (100 MHz, $CDCl_3$).

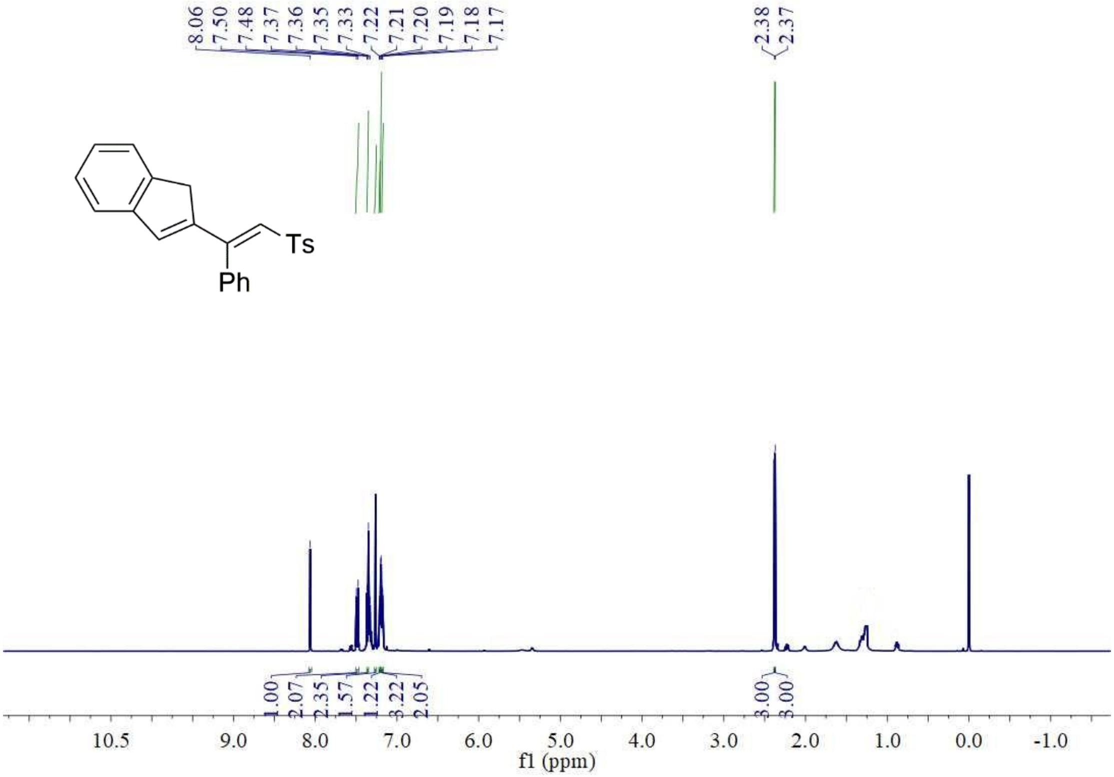

Supplementary Figure 72: ^{1}H NMR of 15b (400 MHz, $CDCl_3$).

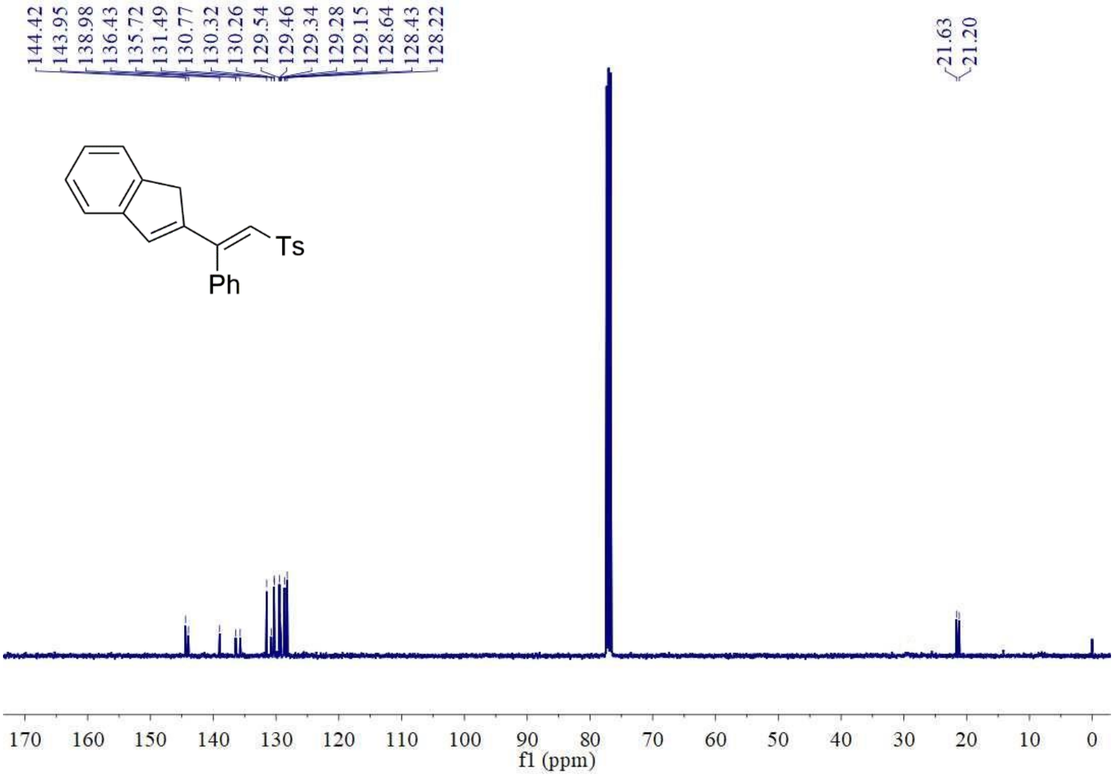

Supplementary Figure 73: ^{13}C NMR of 15b (100 MHz, $CDCl_3$).

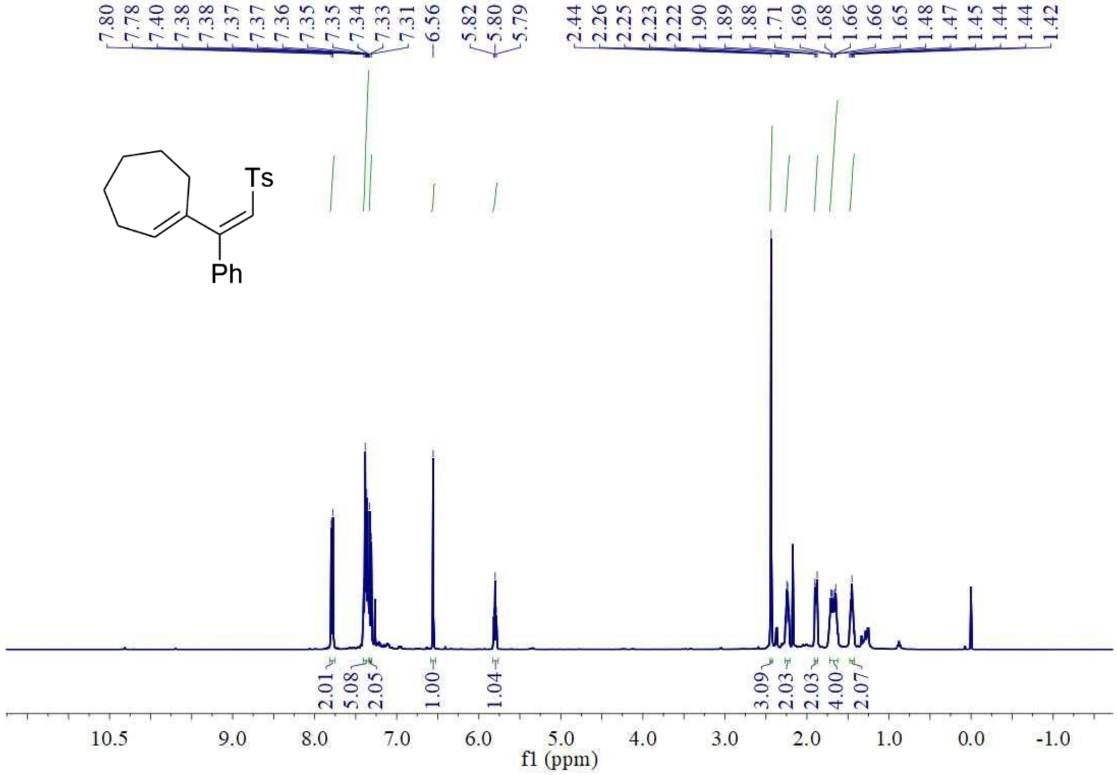

Supplementary Figure 74: ^{1}H NMR of 16a (400 MHz, $CDCl_3$).

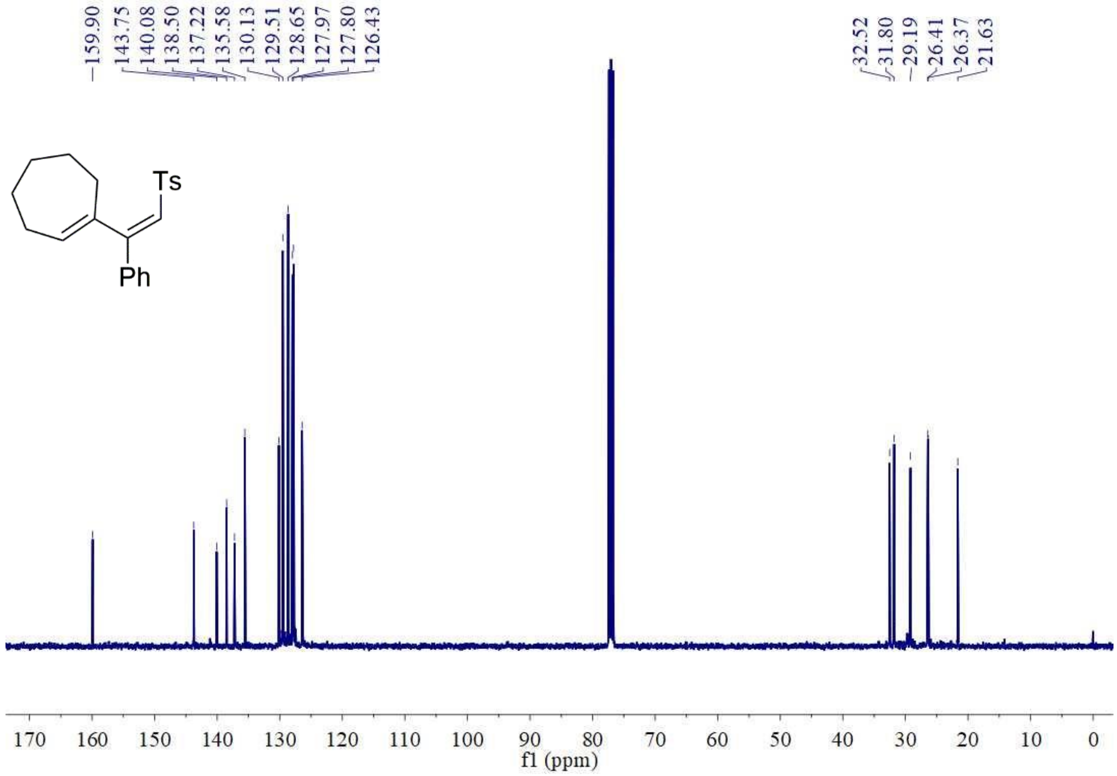

Supplementary Figure 75: ^{13}C NMR of 16a (100 MHz, $CDCl_3$).

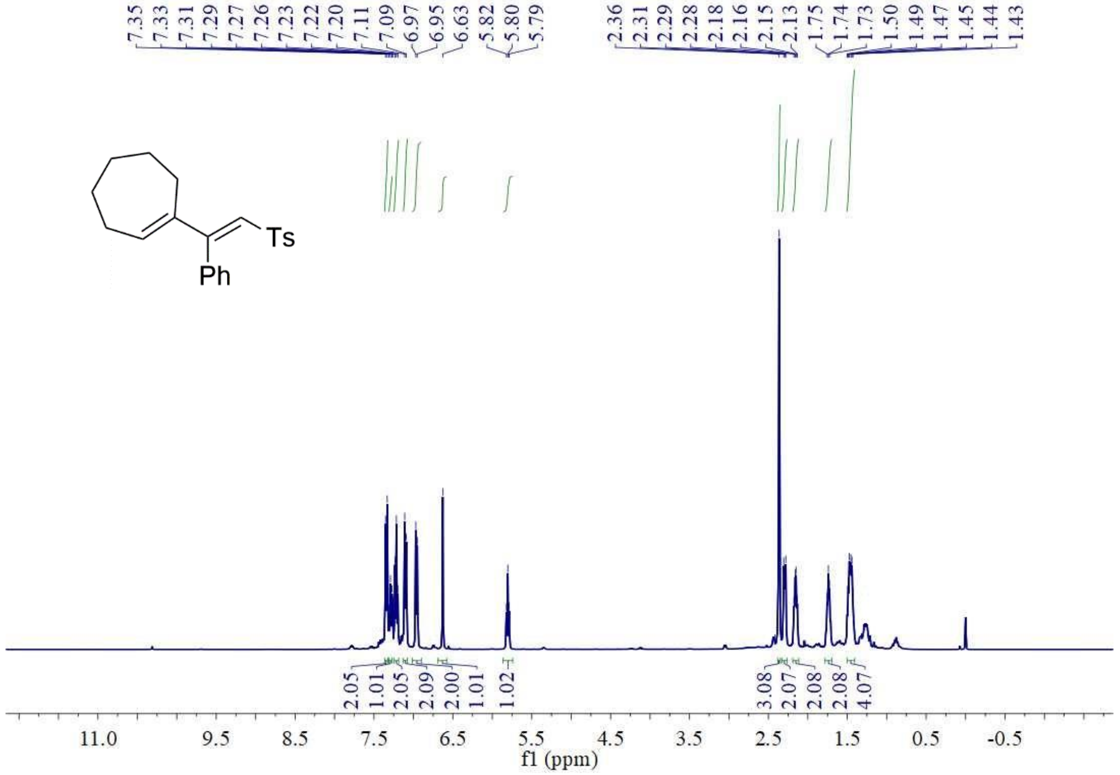

Supplementary Figure 76: ¹H NMR of 16b (400 MHz, $CDCl_3$).

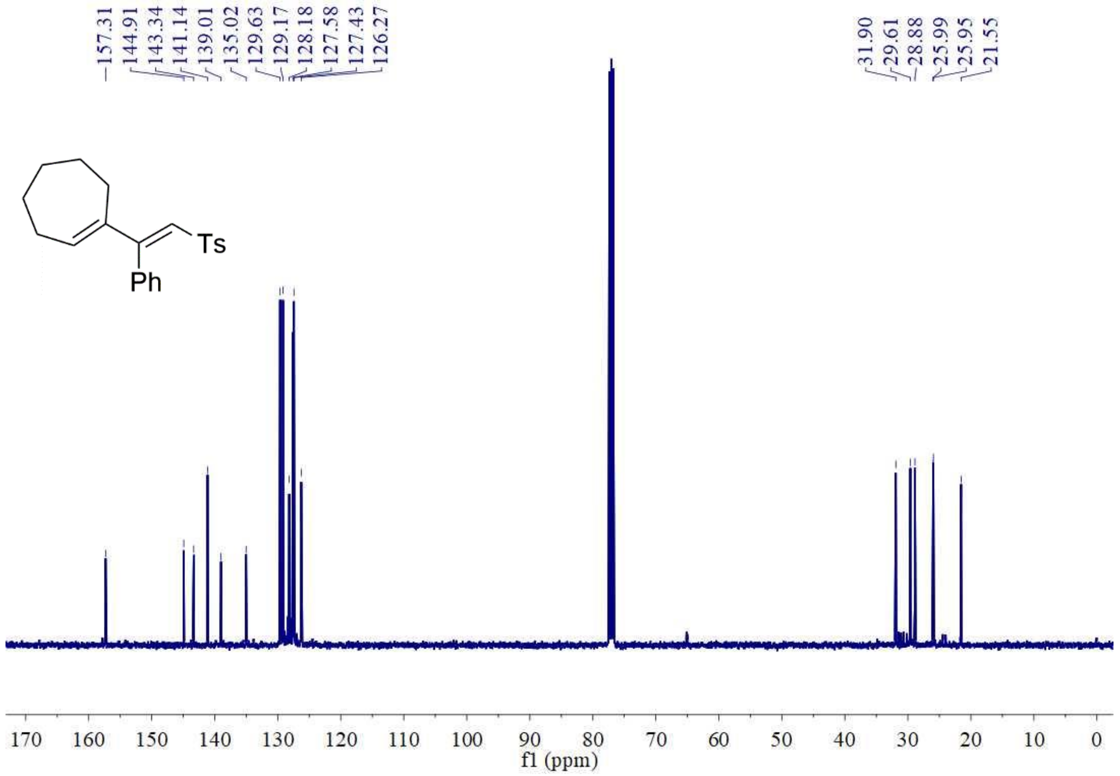

Supplementary Figure 77: ¹³C NMR of 16b (100 MHz, $CDCl_3$).

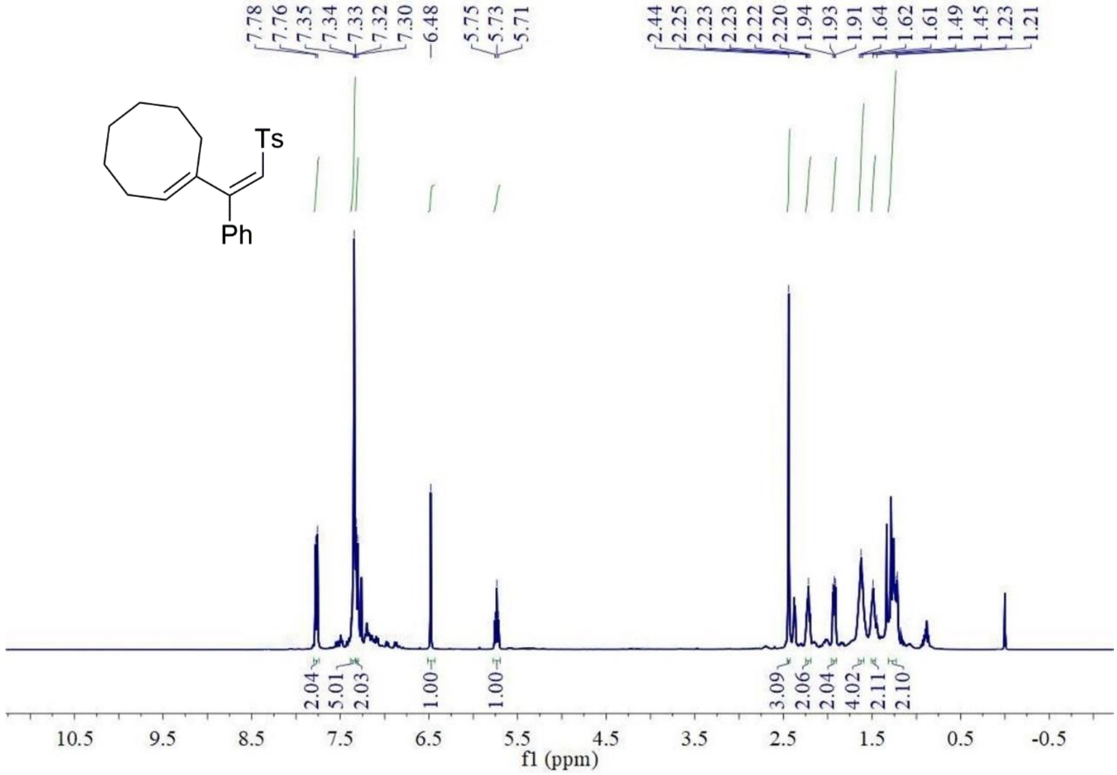

Supplementary Figure 78: ^{1}H NMR of 17a (400 MHz, $CDCl_3$).

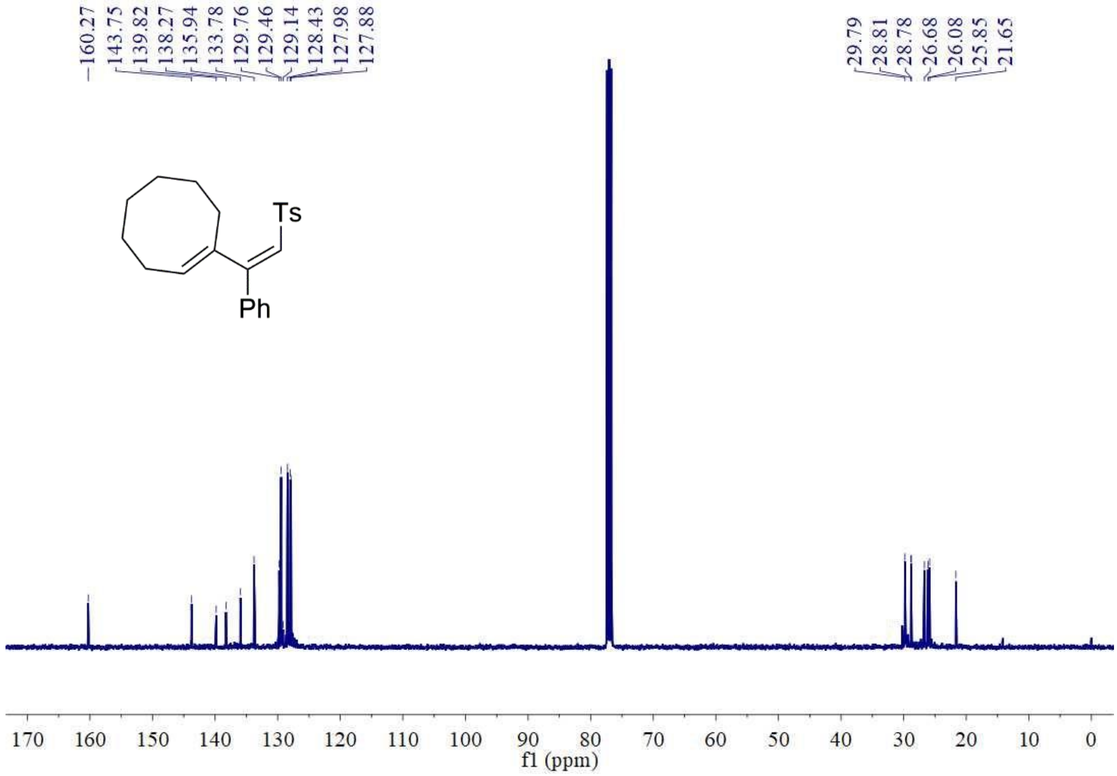

Supplementary Figure 79: ^{13}C NMR of 17a (100 MHz, $CDCl_3$).

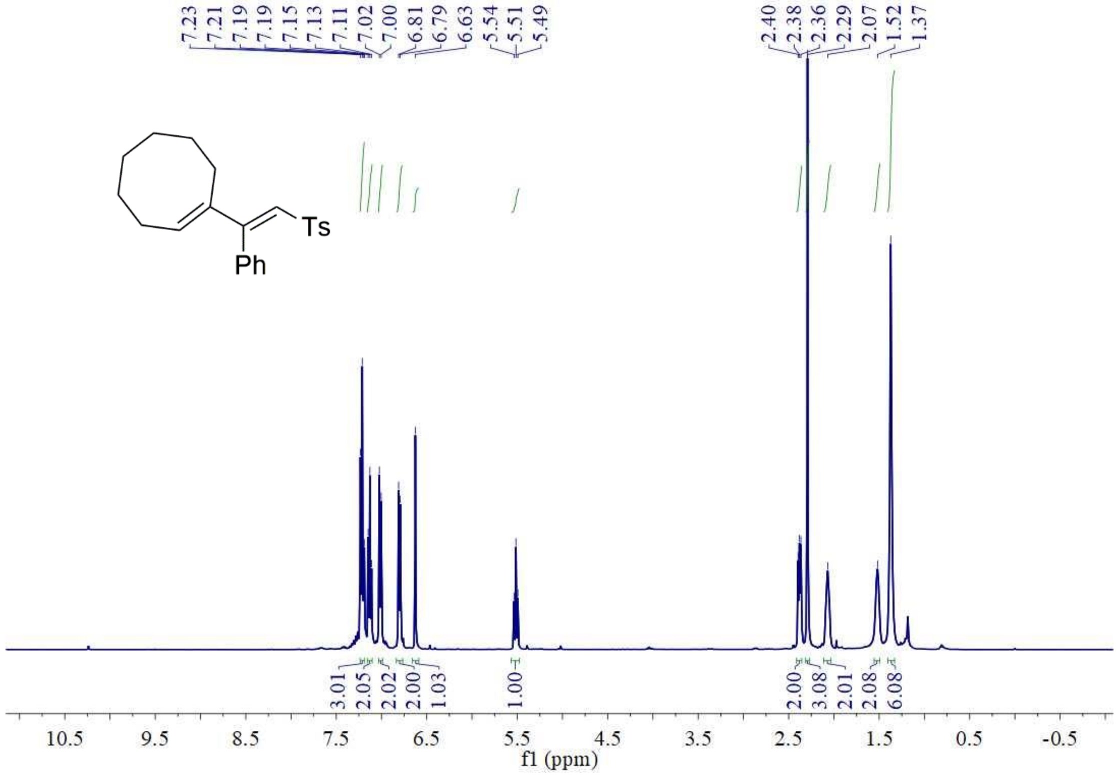

Supplementary Figure 80: ^{1}H NMR of 17b (400 MHz, $CDCl_3$).

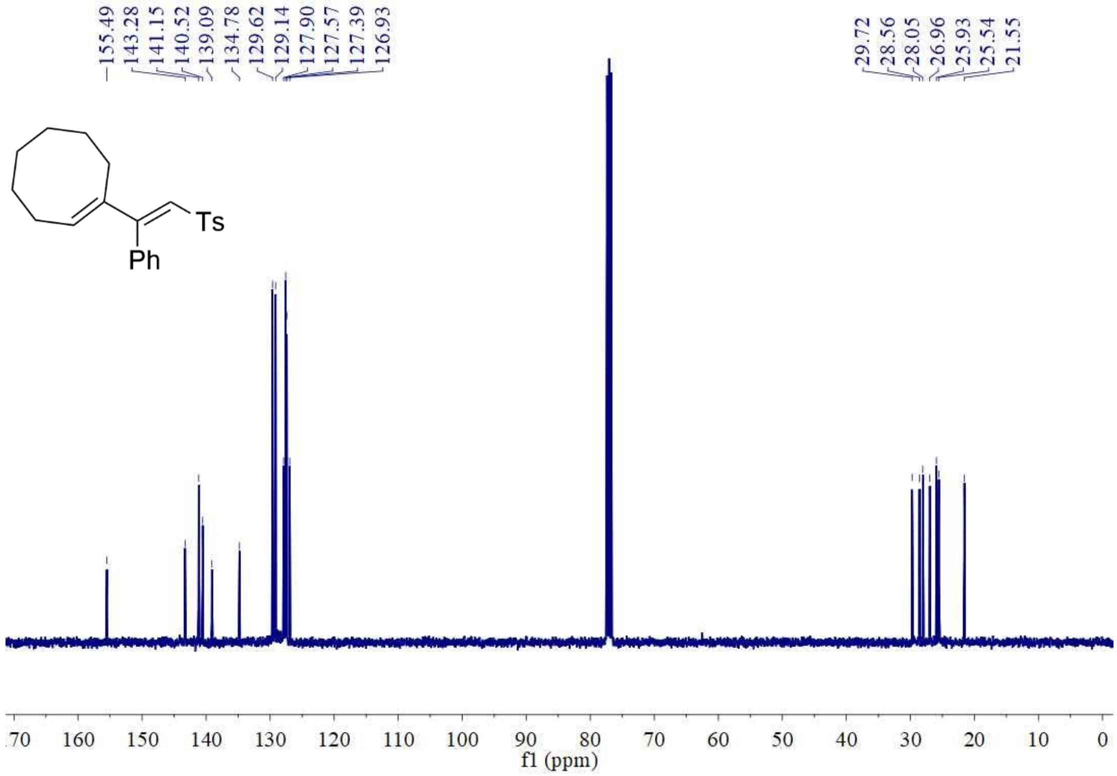

Supplementary Figure 81: ^{13}C NMR of 17b (100 MHz, $CDCl_3$).

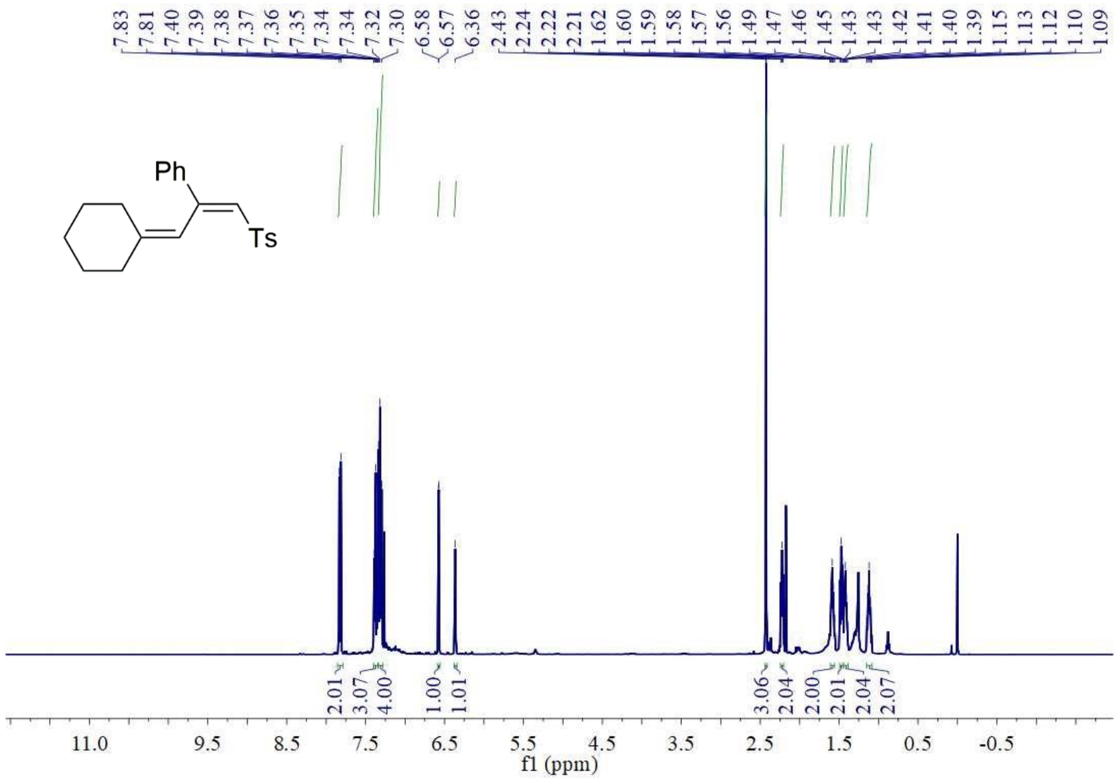

Supplementary Figure 82: ¹H NMR of 18a (400 MHz, $CDCl_3$).

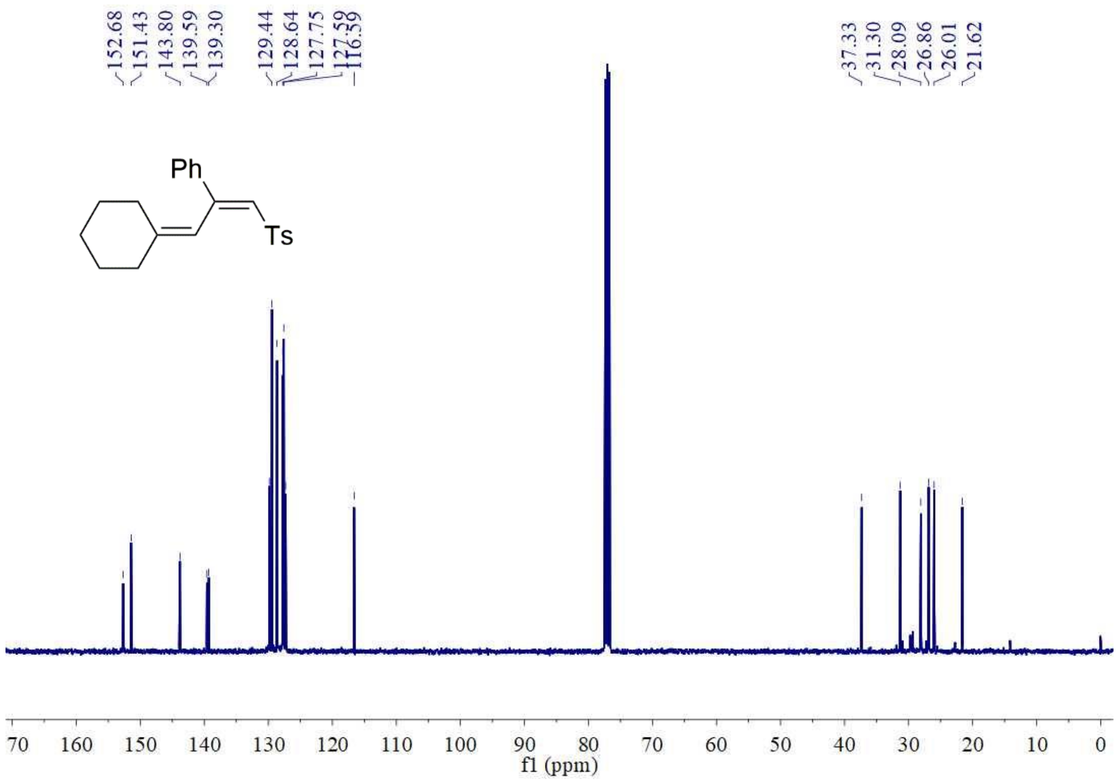

Supplementary Figure 83: ¹³C NMR of 18a (100 MHz, $CDCl_3$).

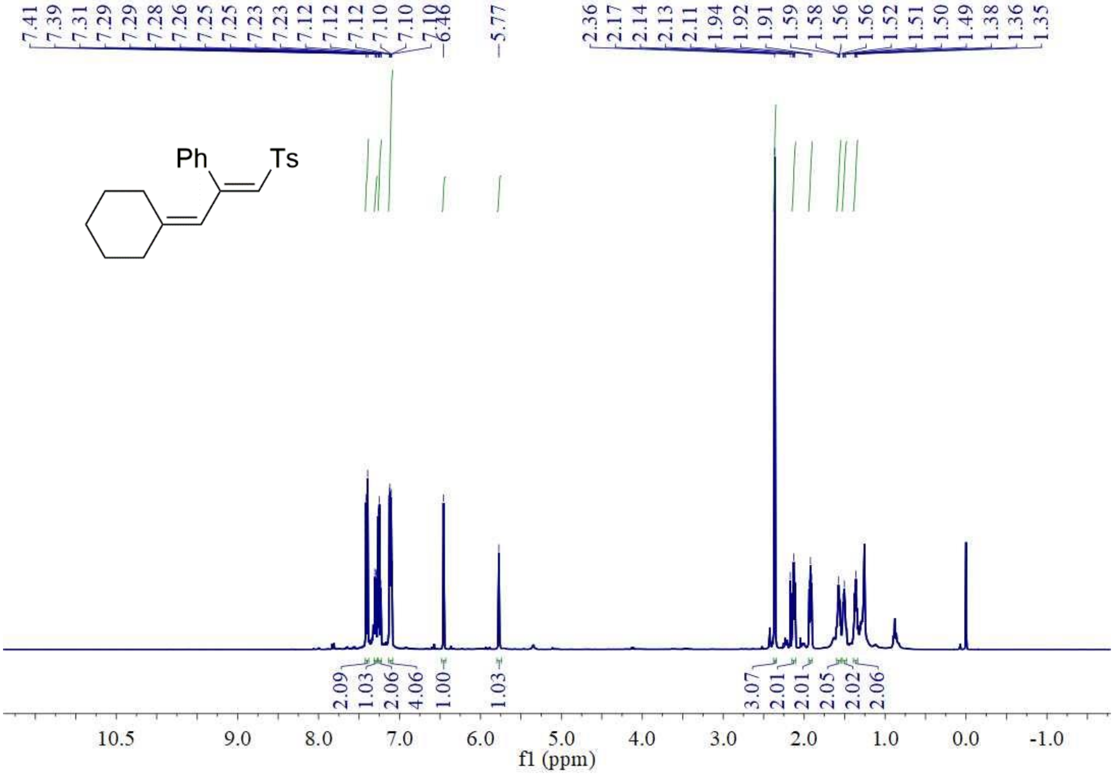

Supplementary Figure 84: ^{1}H NMR of 18b (400 MHz, $CDCl_3$).

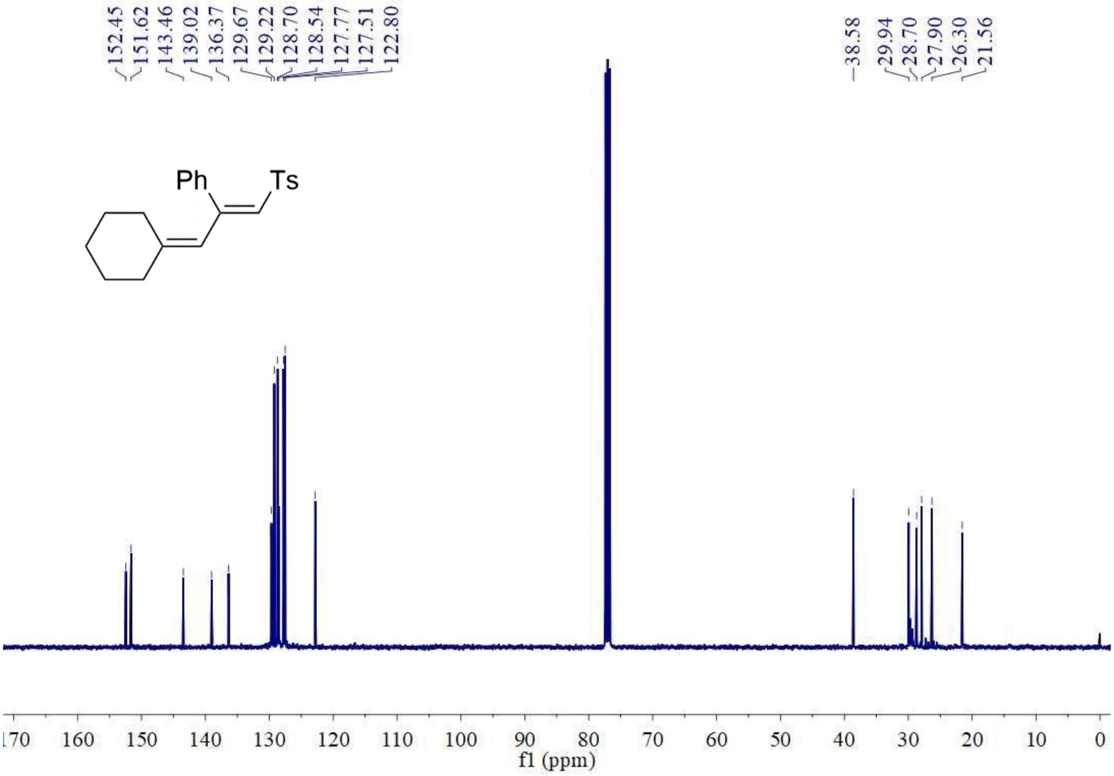

Supplementary Figure 85: ^{13}C NMR of 18b (100 MHz, $CDCl_3$).

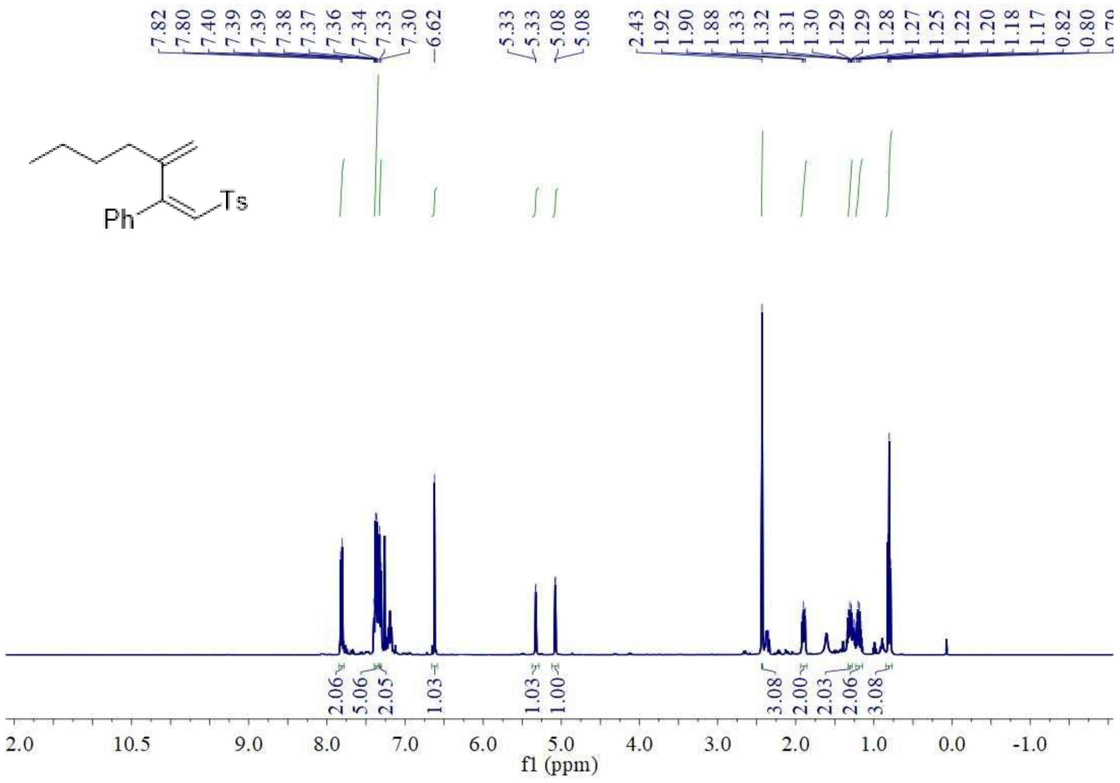

Supplementary Figure 86: **^{1}H NMR of 19a** (400 MHz, $CDCl_3$).

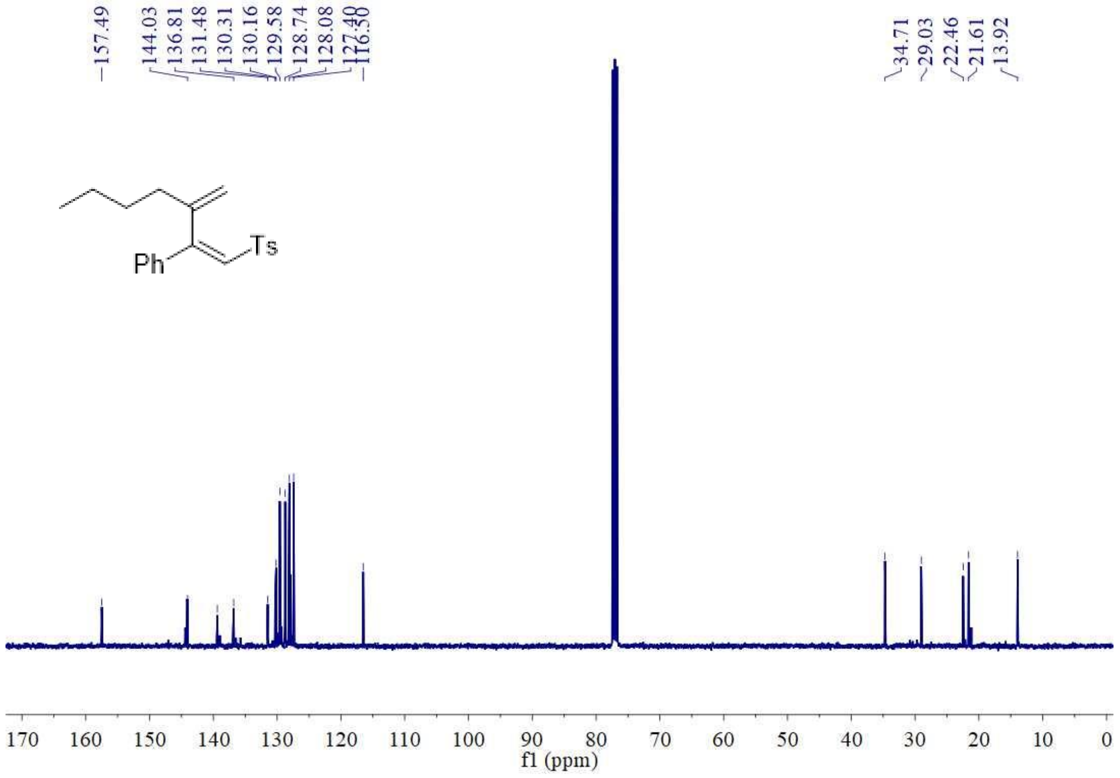

Supplementary Figure 87: **^{13}C NMR of 19a** (100 MHz, $CDCl_3$).

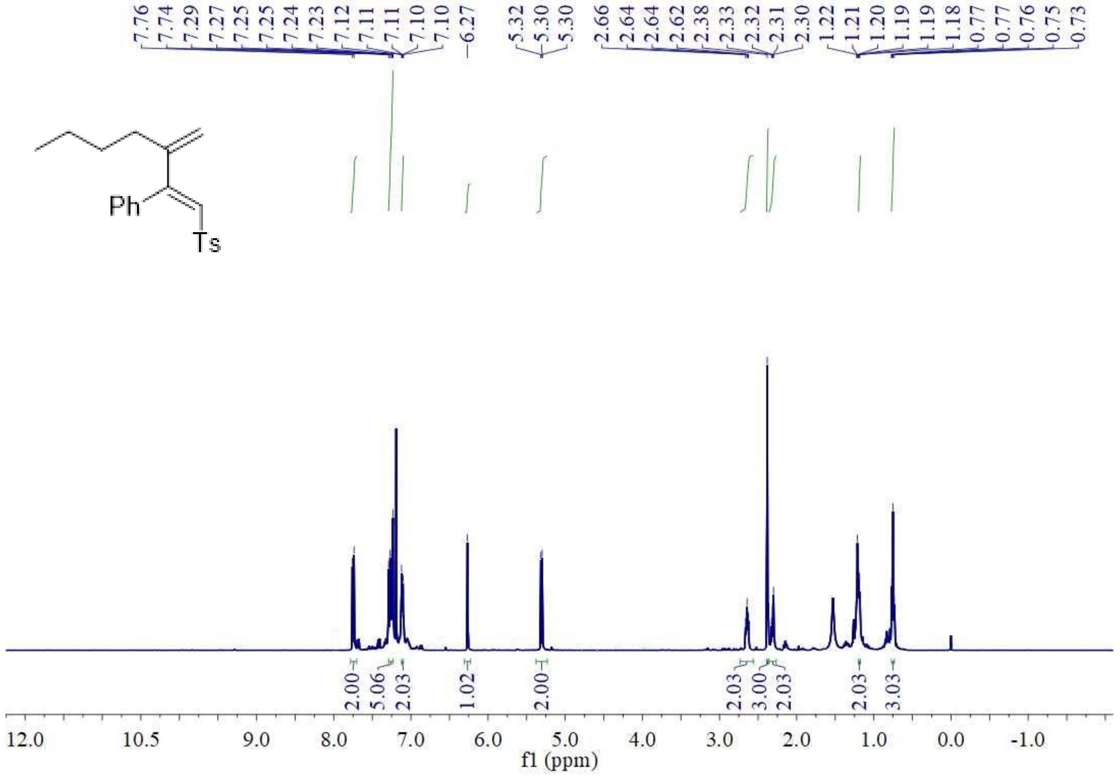

Supplementary Figure 88: ^{1}H NMR of 19b (400 MHz, $CDCl_3$).

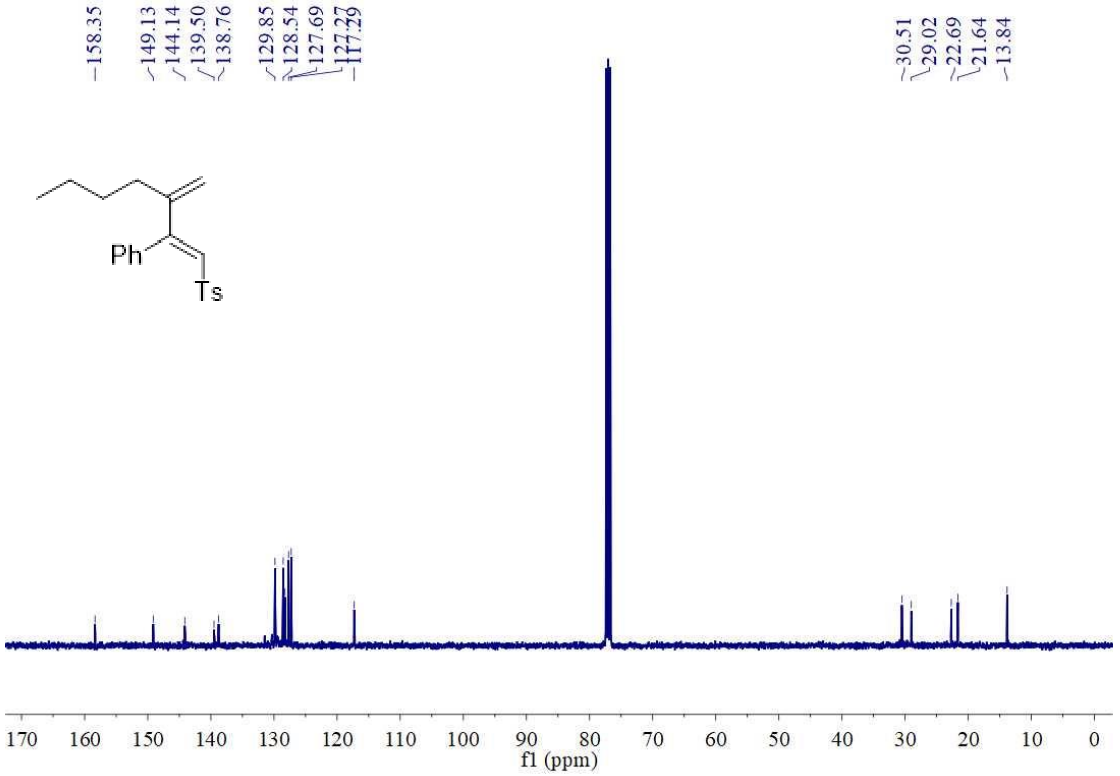

Supplementary Figure 89: ^{13}C NMR of 19b (100 MHz, $CDCl_3$).

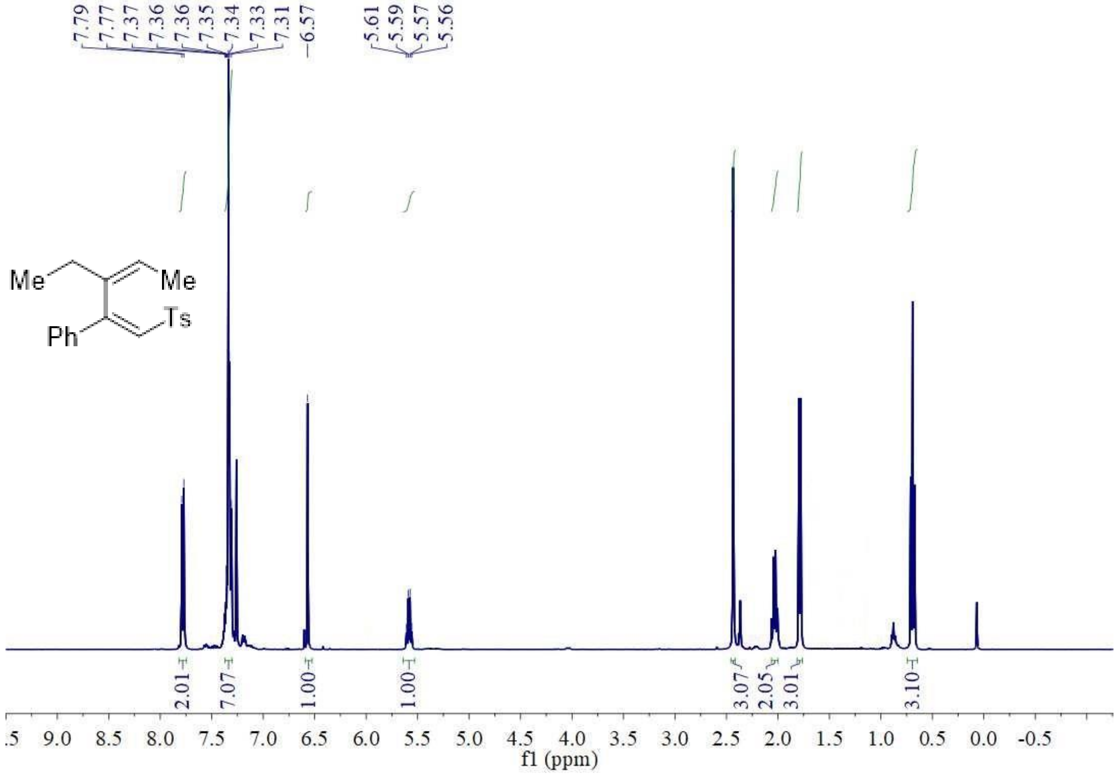

Supplementary Figure 90: ^{1}H NMR of 20a (400 MHz, $CDCl_3$).

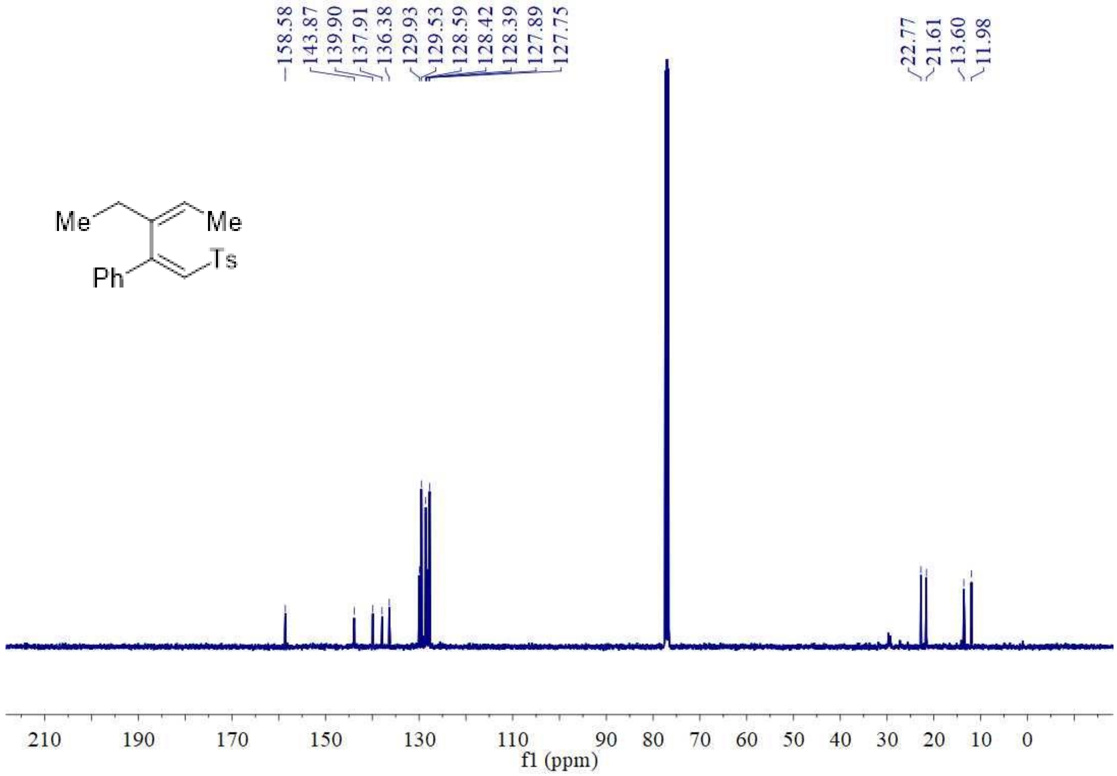

Supplementary Figure 91: ^{13}C NMR of 20a (100 MHz, $CDCl_3$).

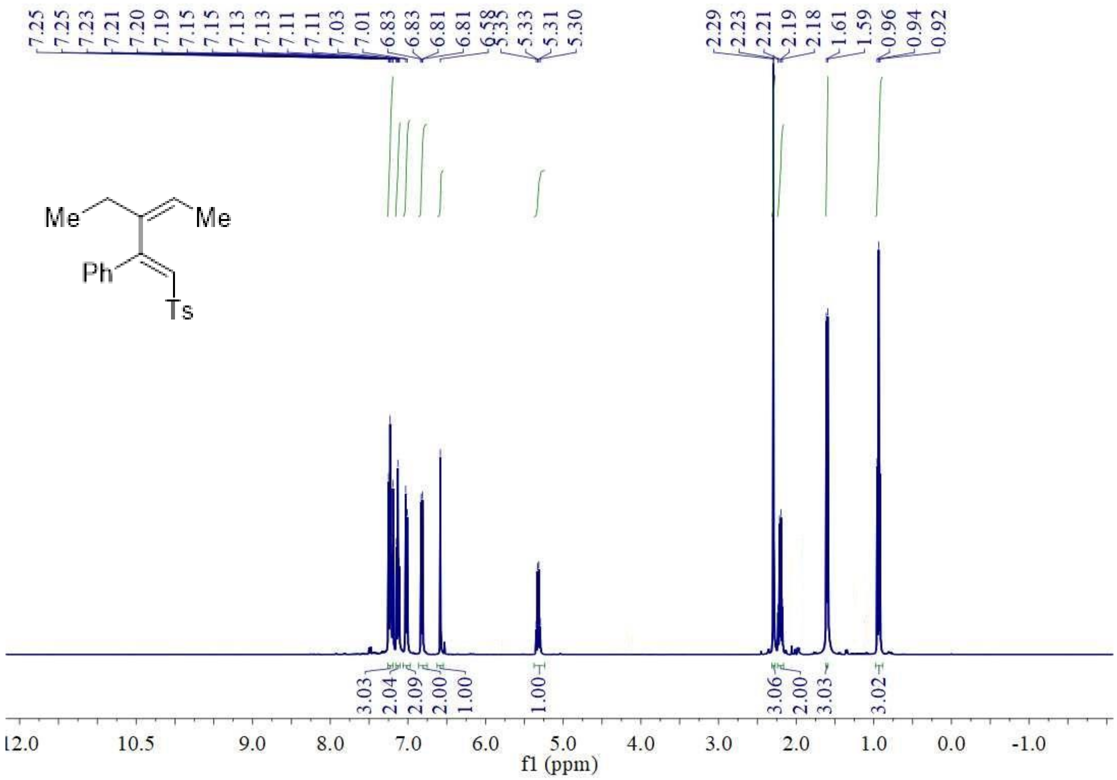

Supplementary Figure 92: ^{1}H NMR of 20b (400 MHz, $CDCl_3$).

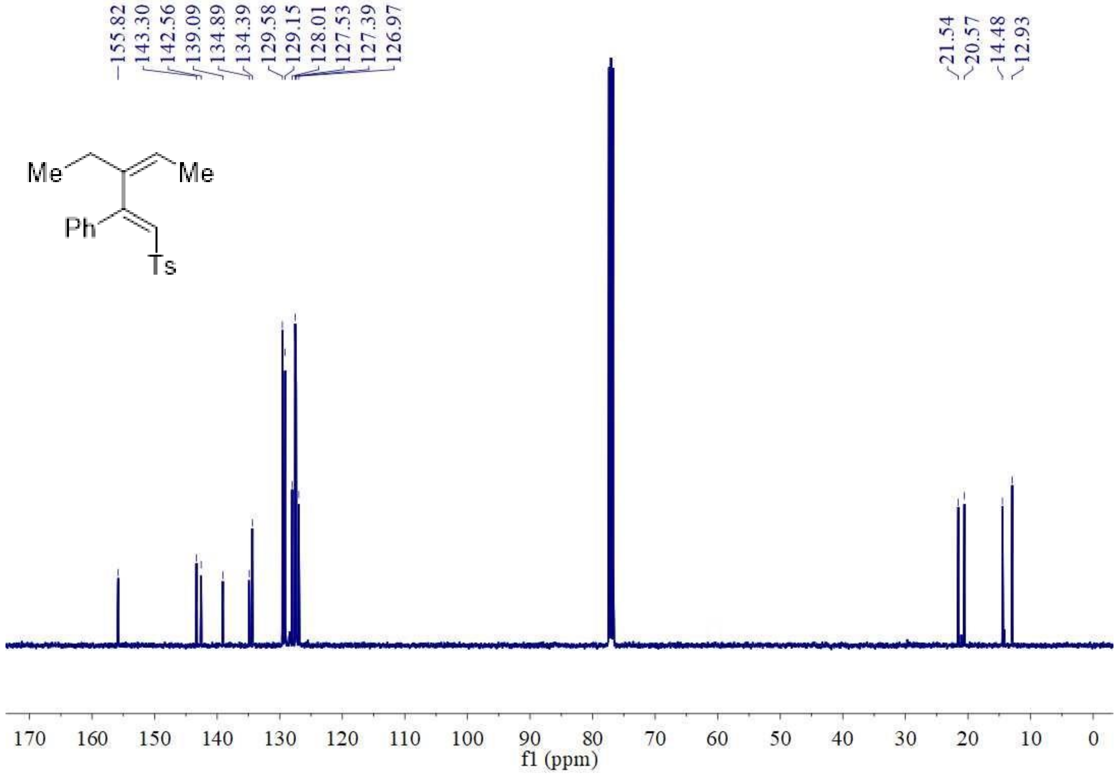

Supplementary Figure 93: ^{13}C NMR of 20b (100 MHz, $CDCl_3$).

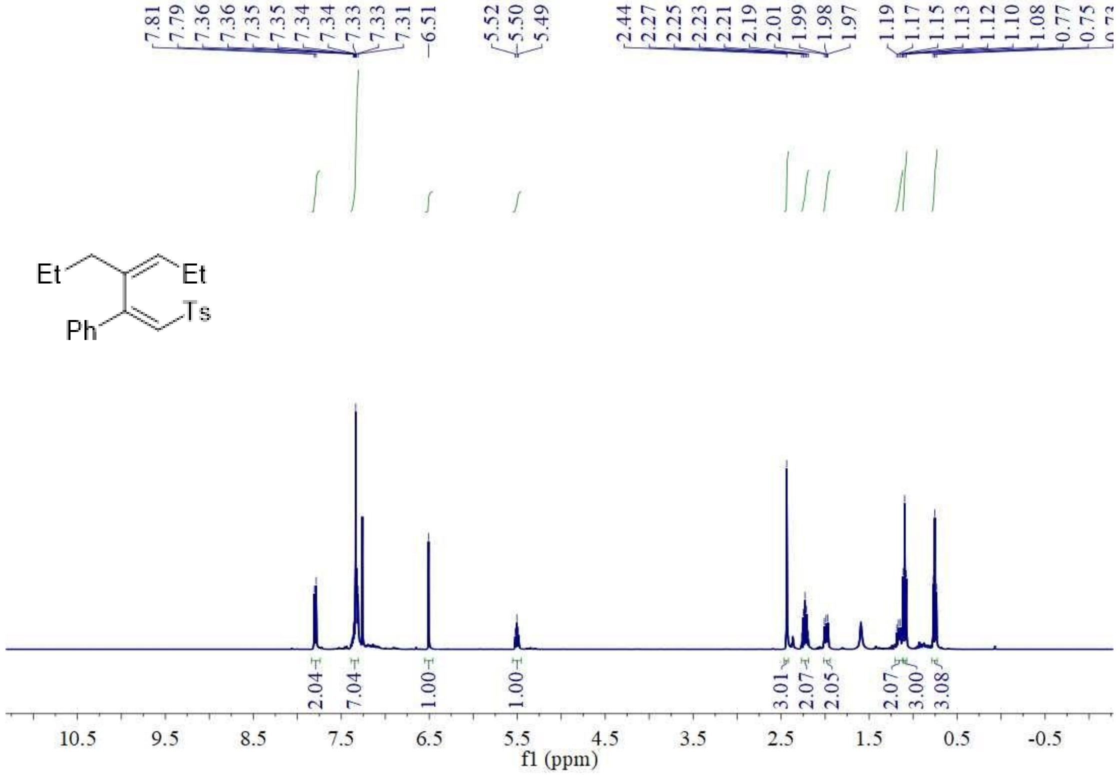

Supplementary Figure 94: ^{1}H NMR of 21a (400 MHz, $CDCl_3$).

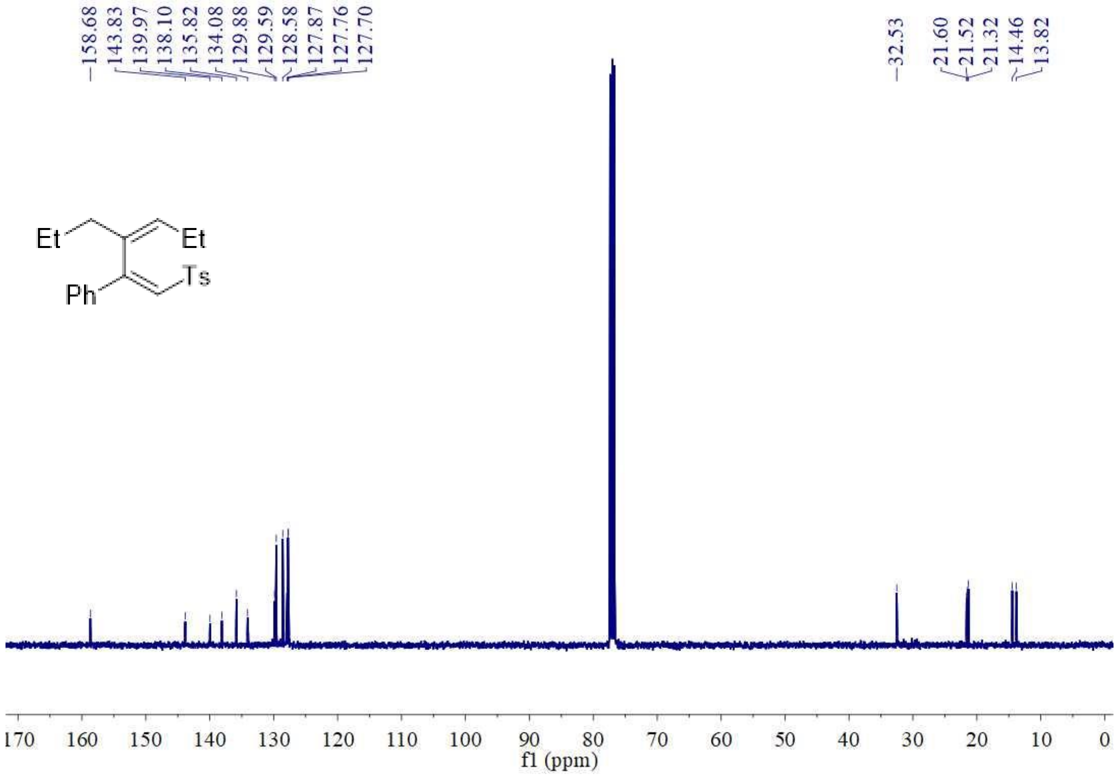

Supplementary Figure 95: ^{13}C NMR of 21a (100 MHz, $CDCl_3$).

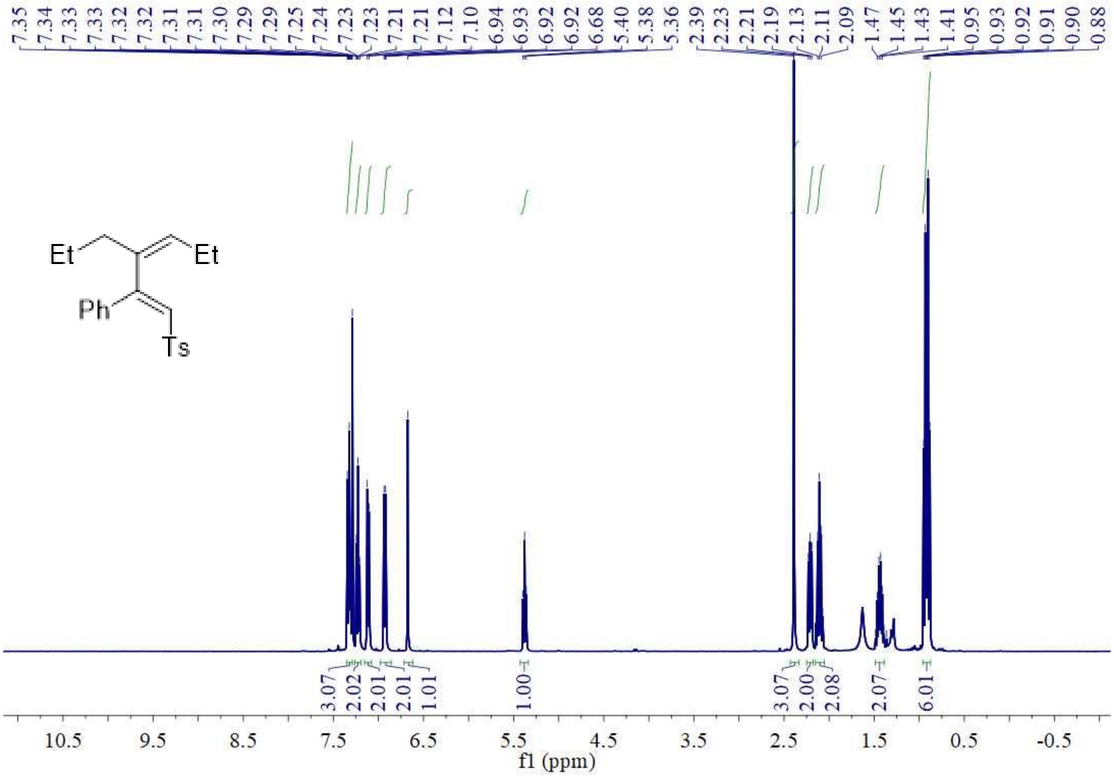

Supplementary Figure 96: ^{1}H NMR of 21b (400 MHz, $CDCl_3$).

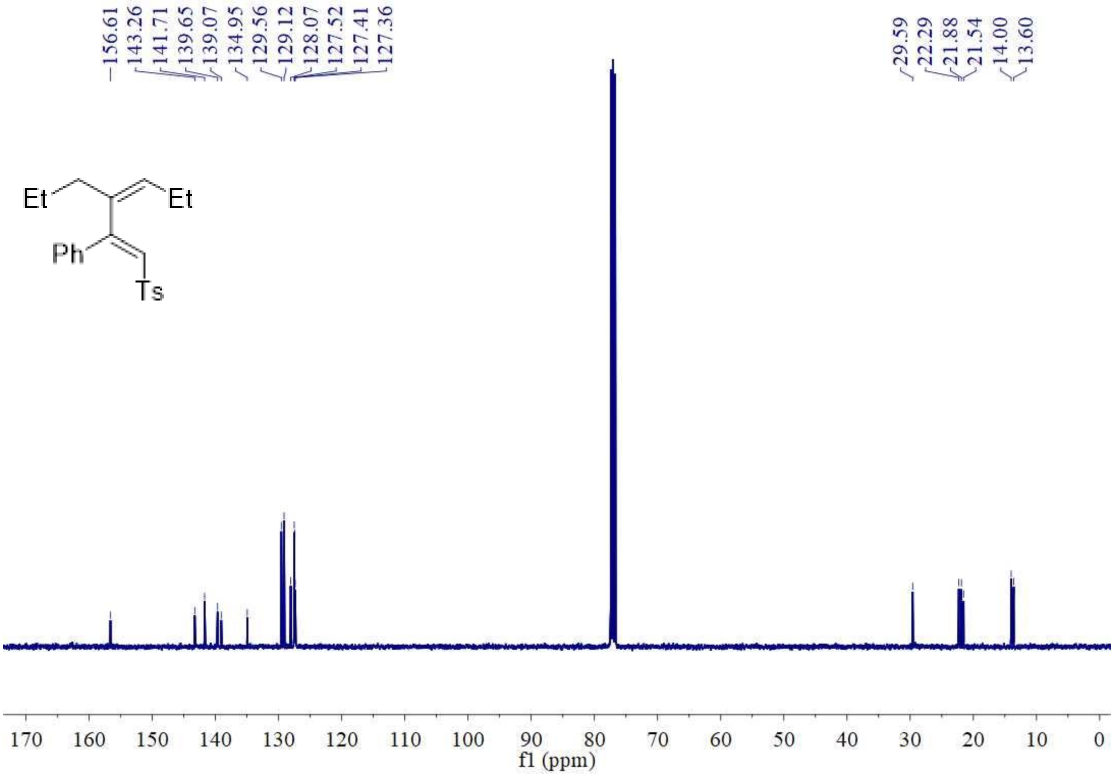

Supplementary Figure 97: ^{13}C NMR of 21b (100 MHz, $CDCl_3$).

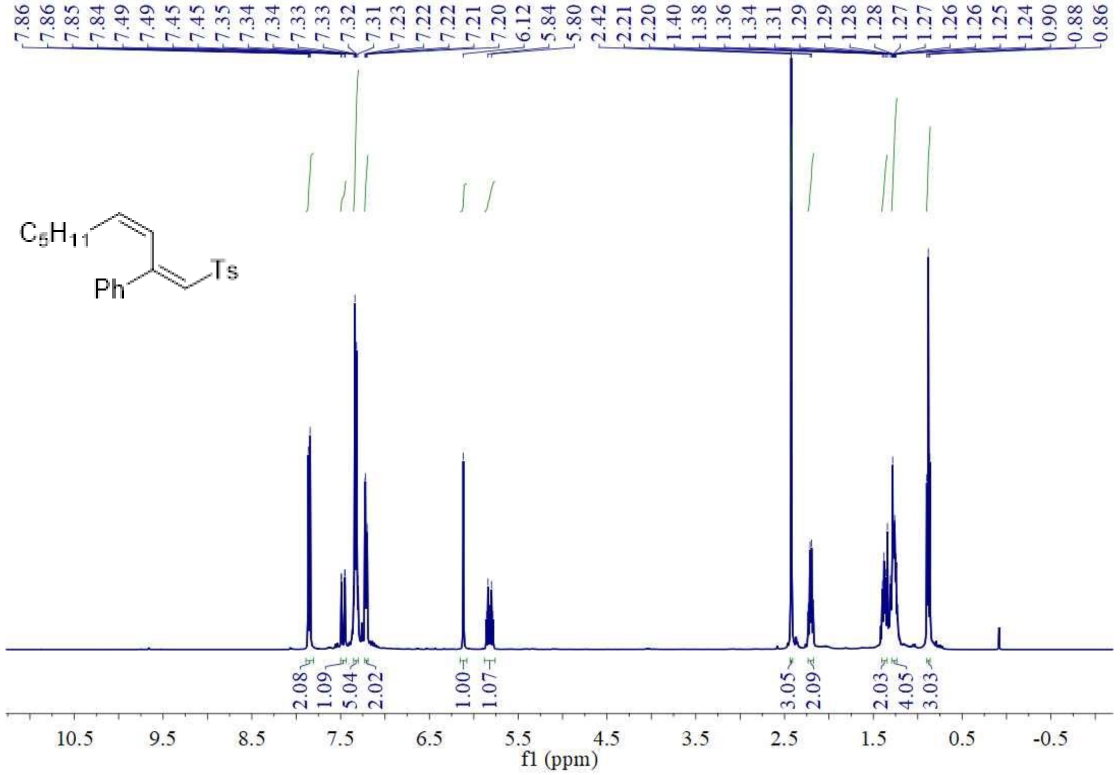

Supplementary Figure 98: ^{1}H NMR of 22a (400 MHz, $CDCl_3$).

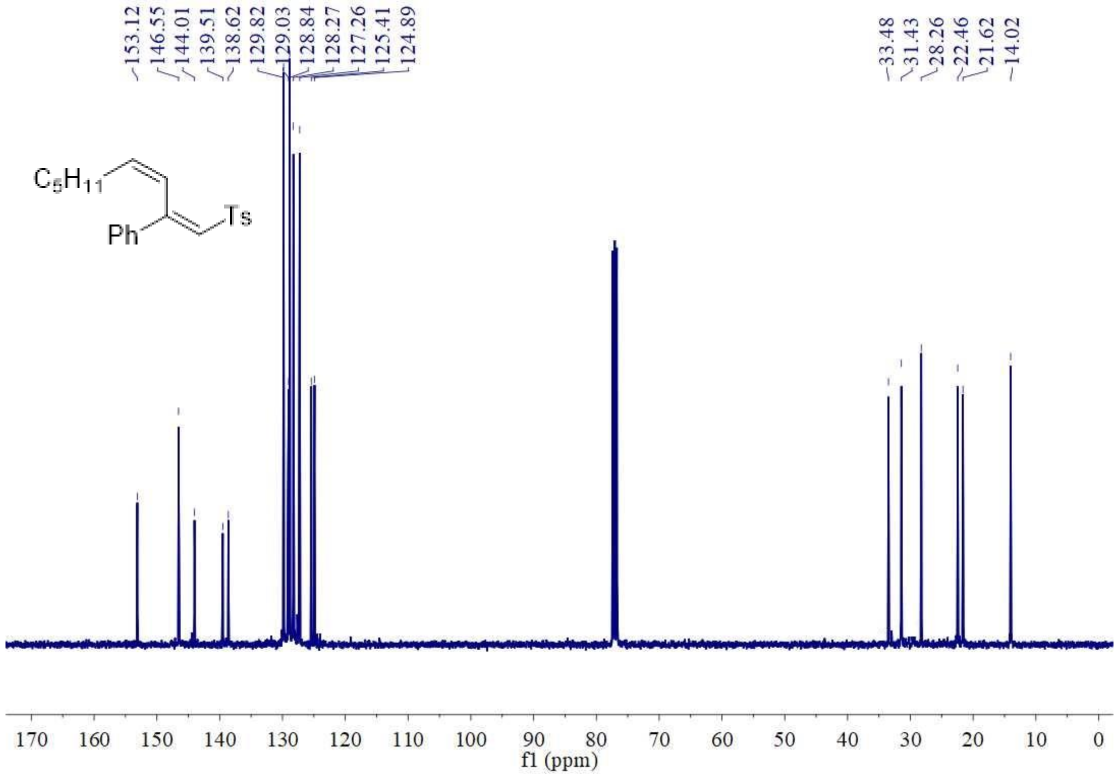

Supplementary Figure 99: ^{13}C NMR of 22a (100 MHz, $CDCl_3$).

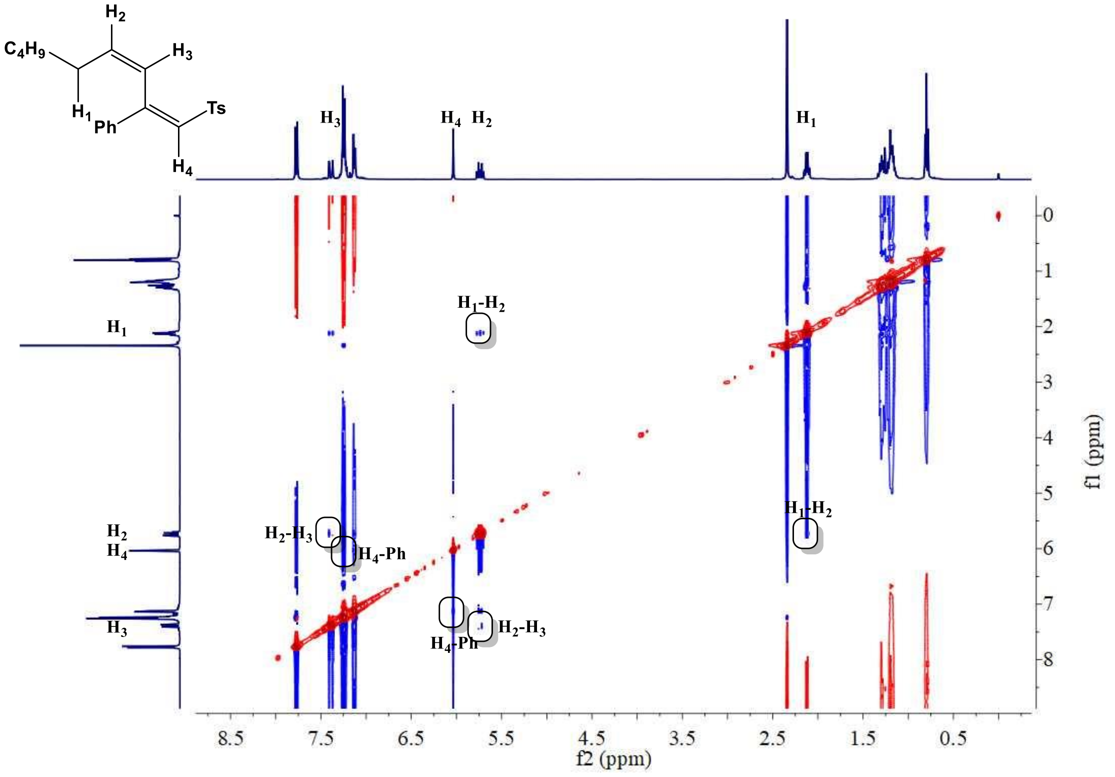

Supplementary Figure 100: NOESY of 22a.

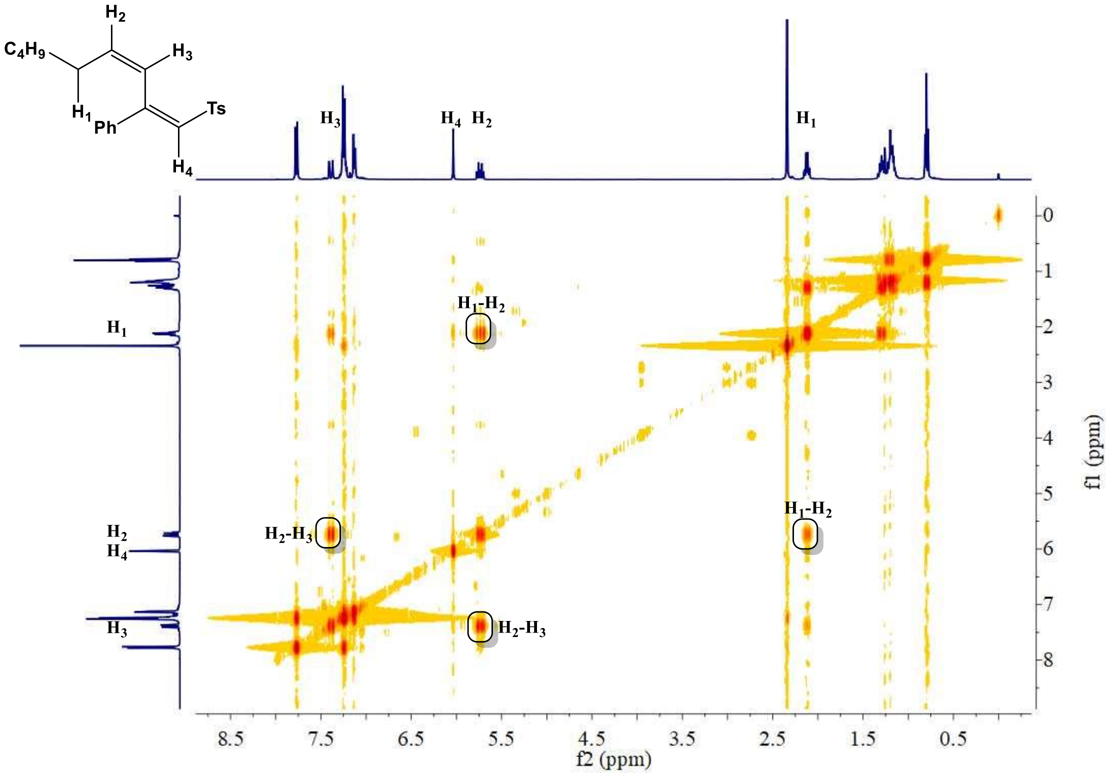

Supplementary Figure 101: COSY of 22a.

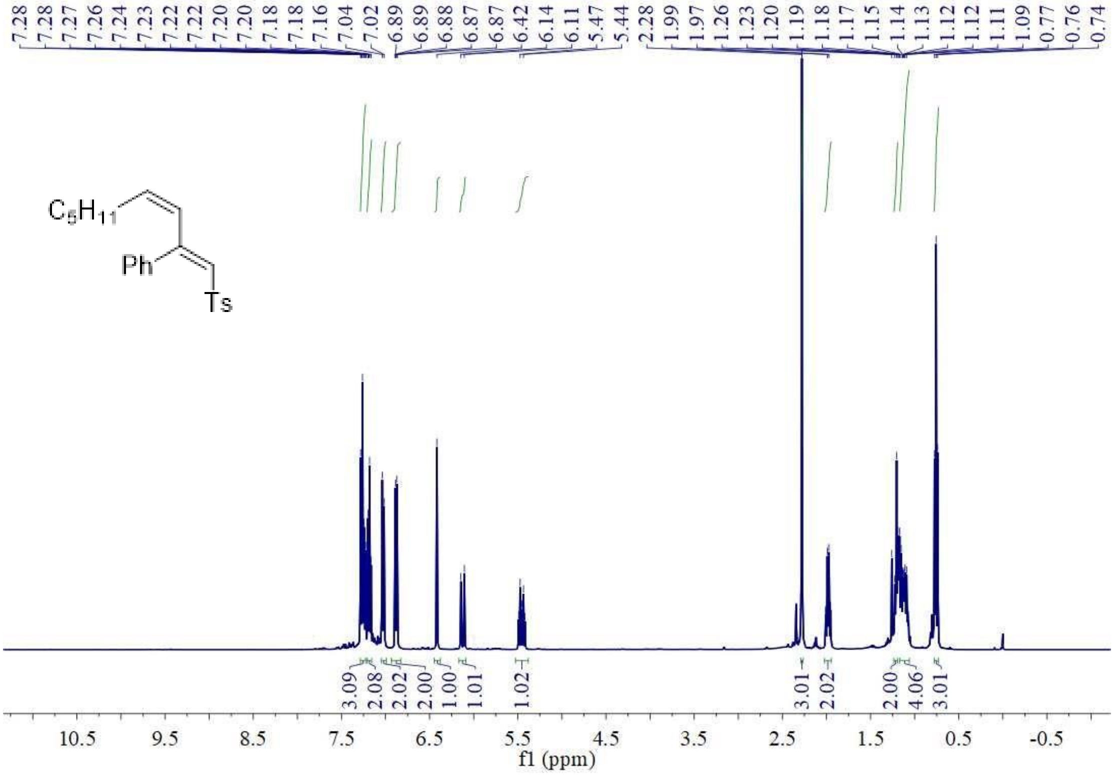

Supplementary Figure 102: ^{1}H NMR of 22b (400 MHz, $CDCl_3$).

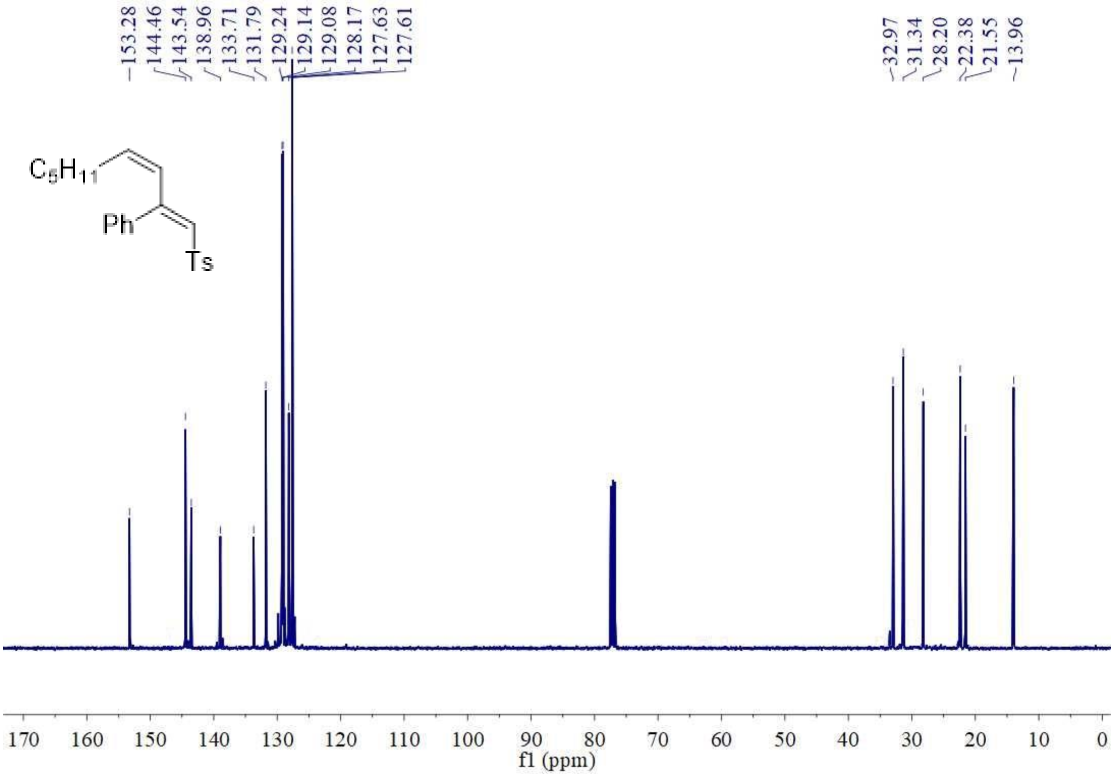

Supplementary Figure 103: ^{13}C NMR of 22b (100 MHz, $CDCl_3$).

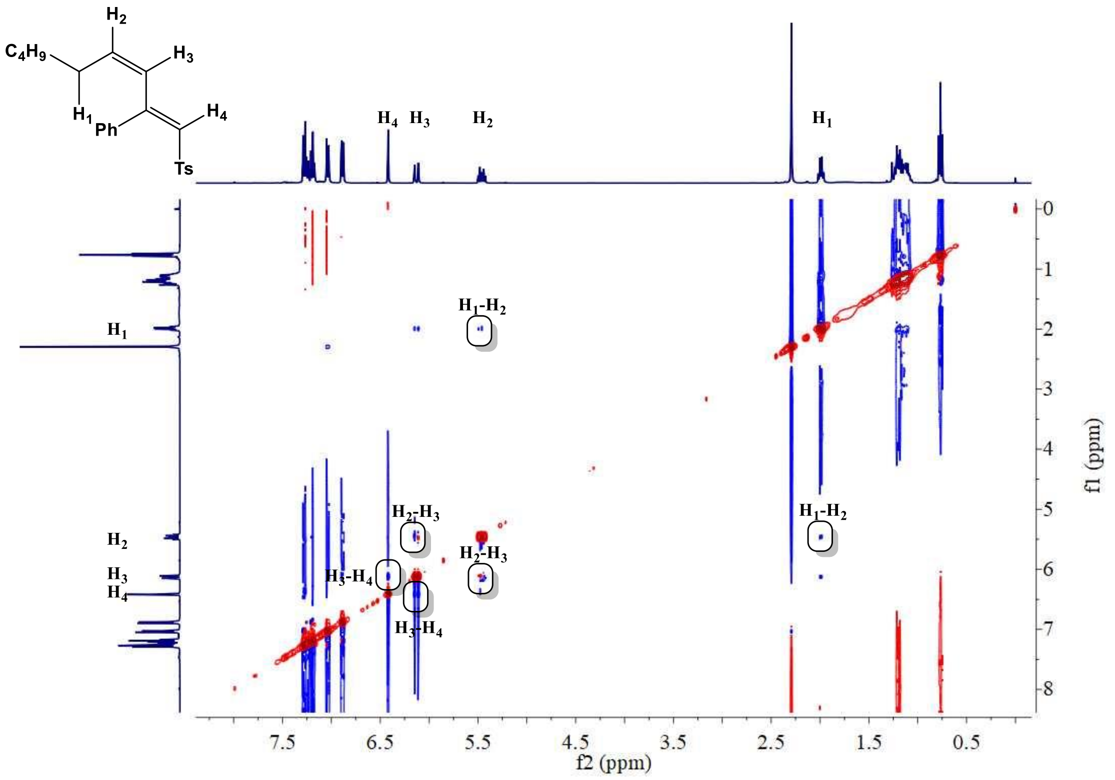

Supplementary Figure 104: NOESY of 22b.

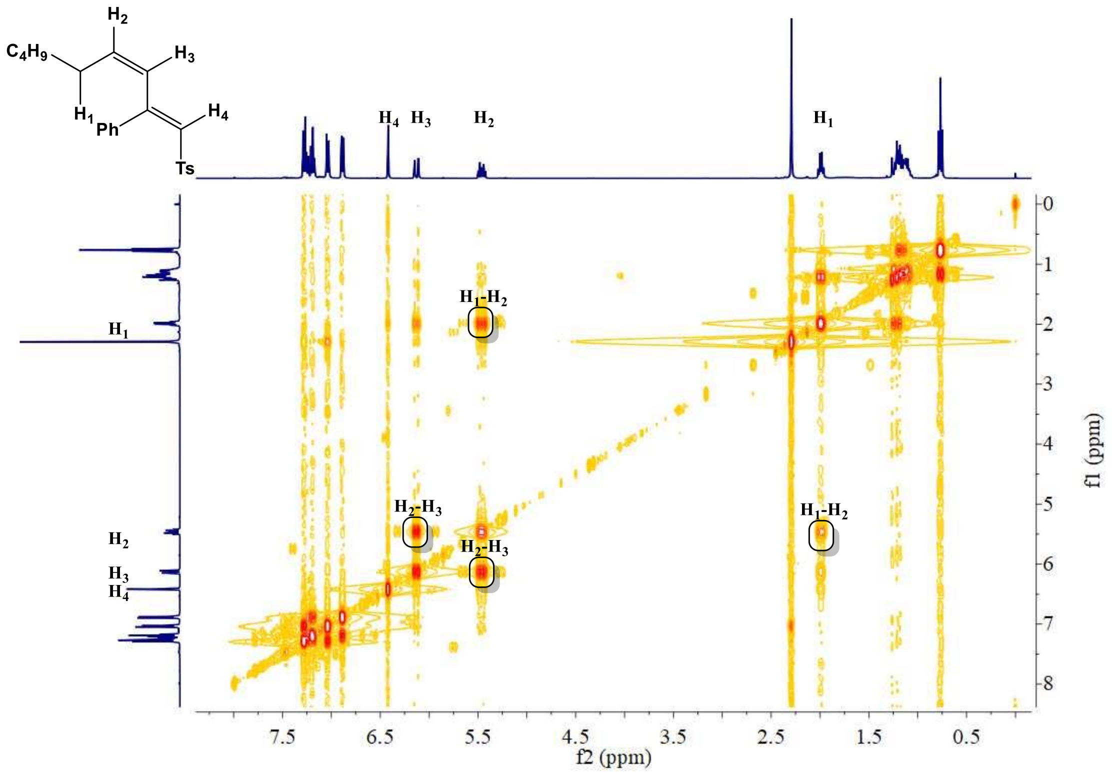

Supplementary Figure 105: COSY of 22b.

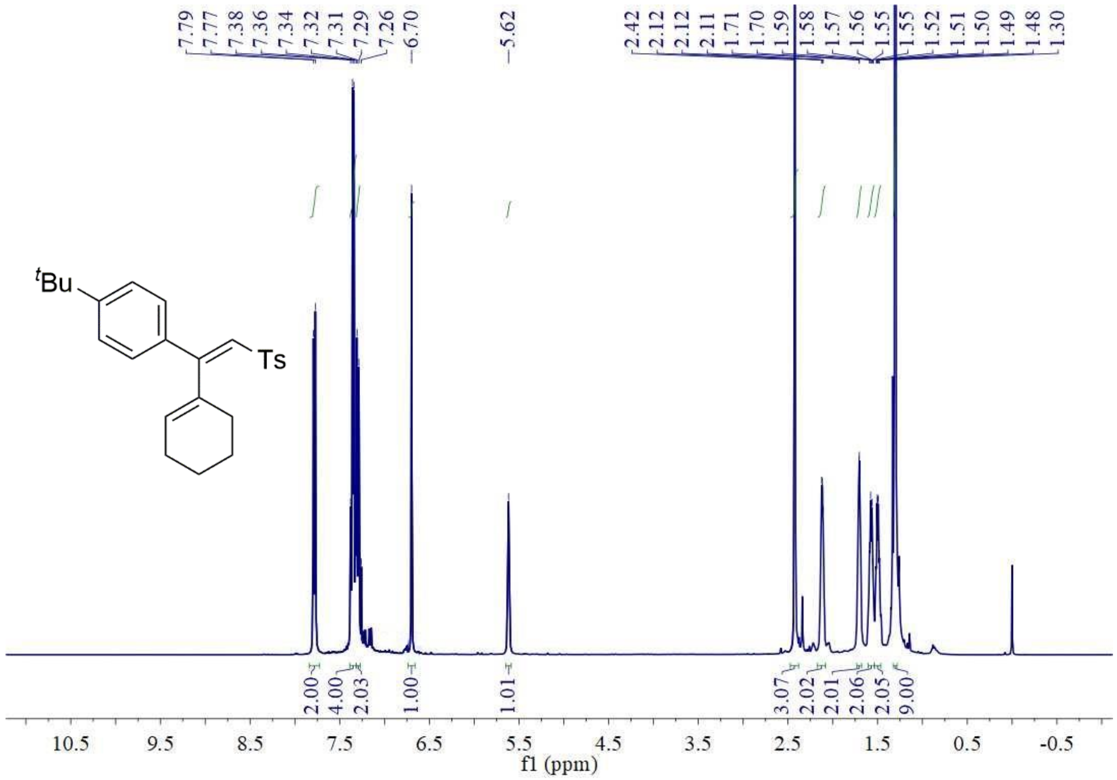

Supplementary Figure 106: ^{1}H NMR of 23a (400 MHz, $CDCl_3$).

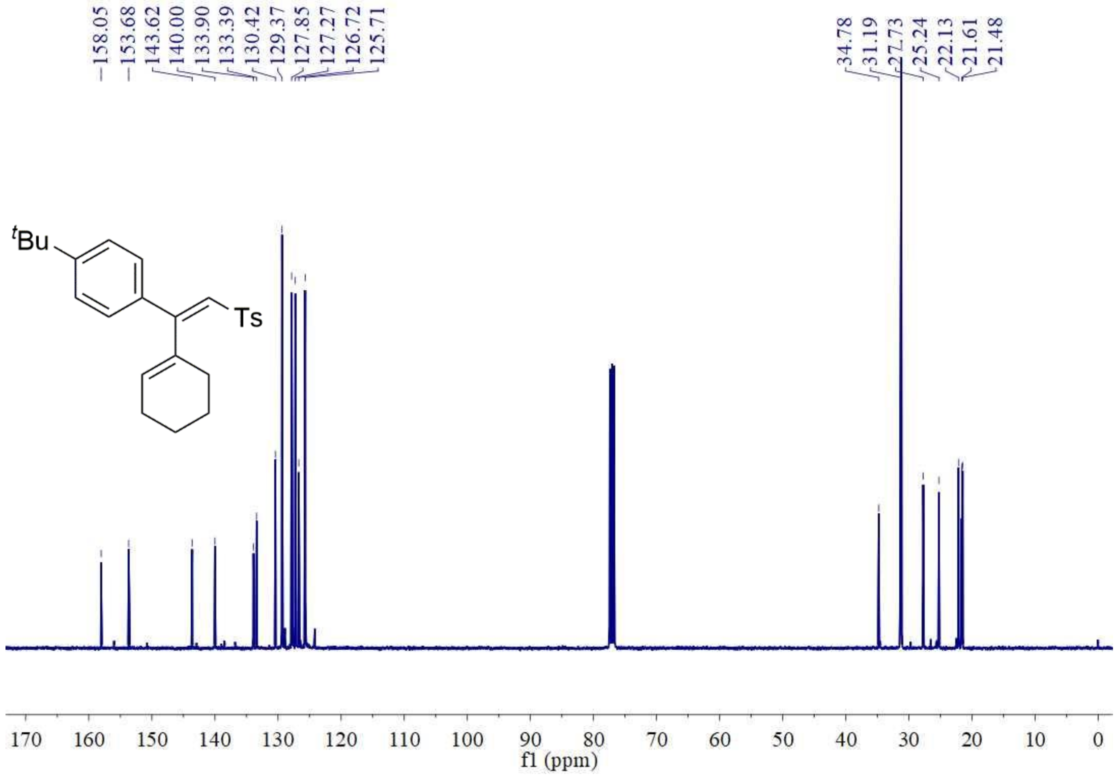

Supplementary Figure 107: ^{13}C NMR of 23a (100 MHz, $CDCl_3$).

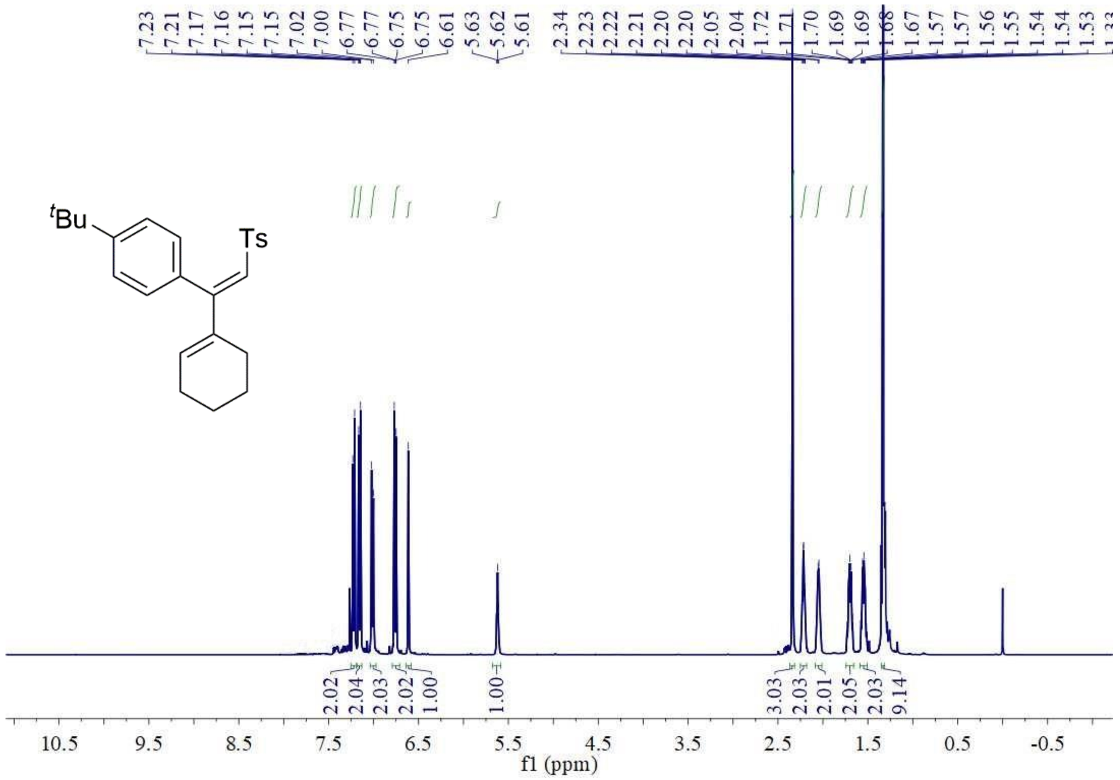

Supplementary Figure 108: ^{1}H NMR of 23b (400 MHz, $CDCl_3$).

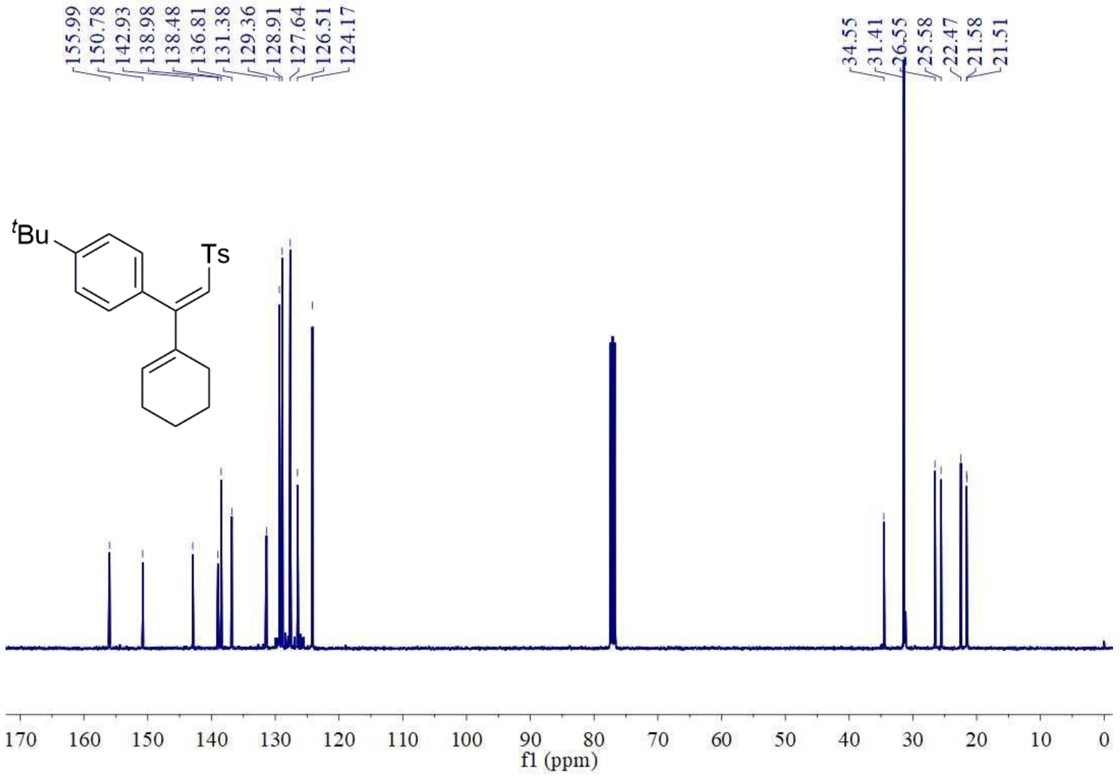

Supplementary Figure 109: ^{13}C NMR of 23b (100 MHz, $CDCl_3$).

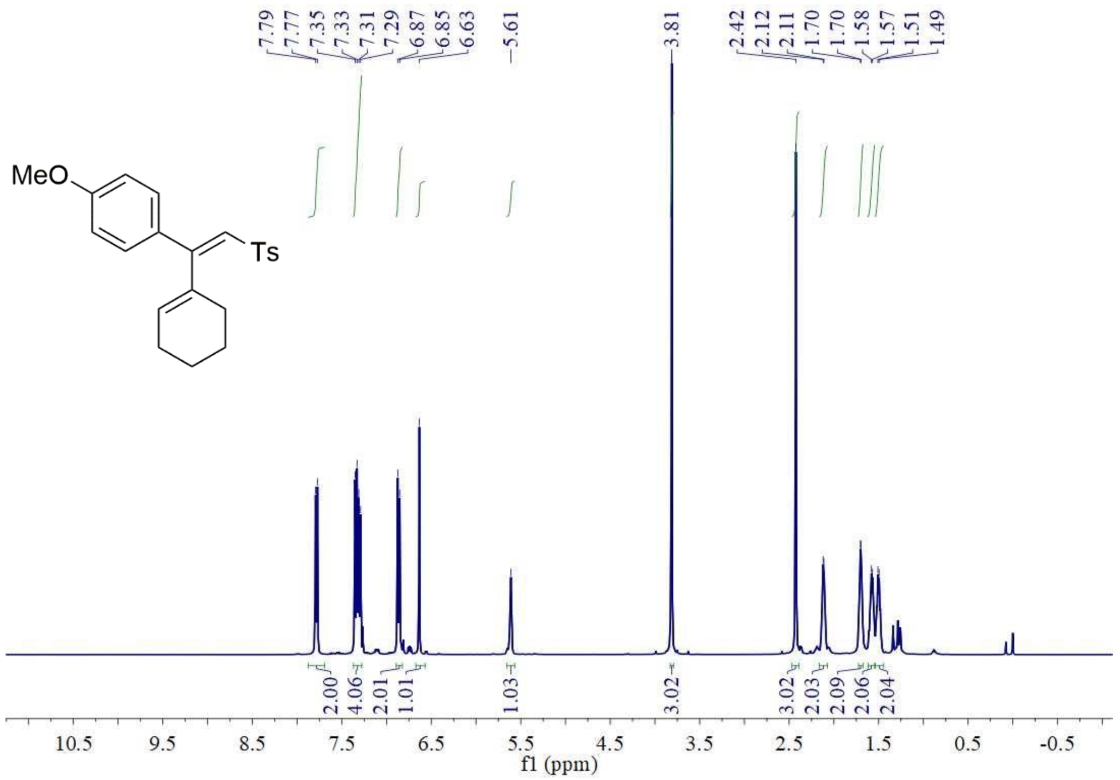

Supplementary Figure 110: ^{1}H NMR of 24a (400 MHz, $CDCl_3$).

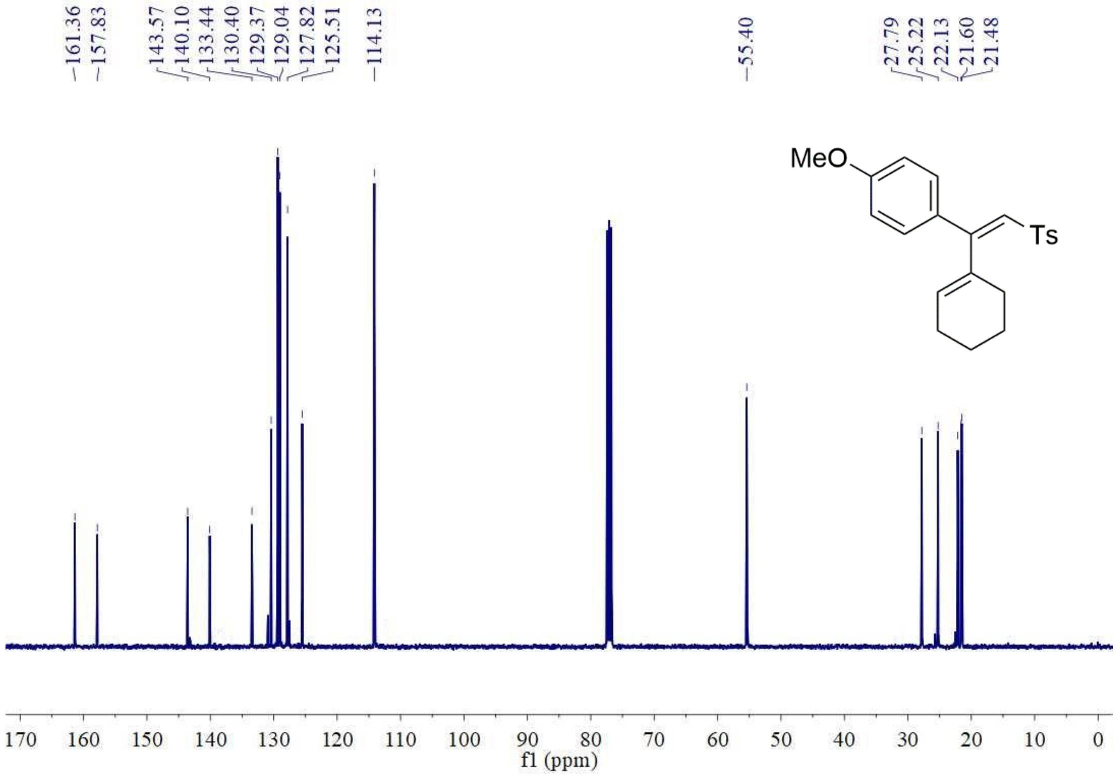

Supplementary Figure 111: ^{13}C NMR of 24a (100 MHz, $CDCl_3$).

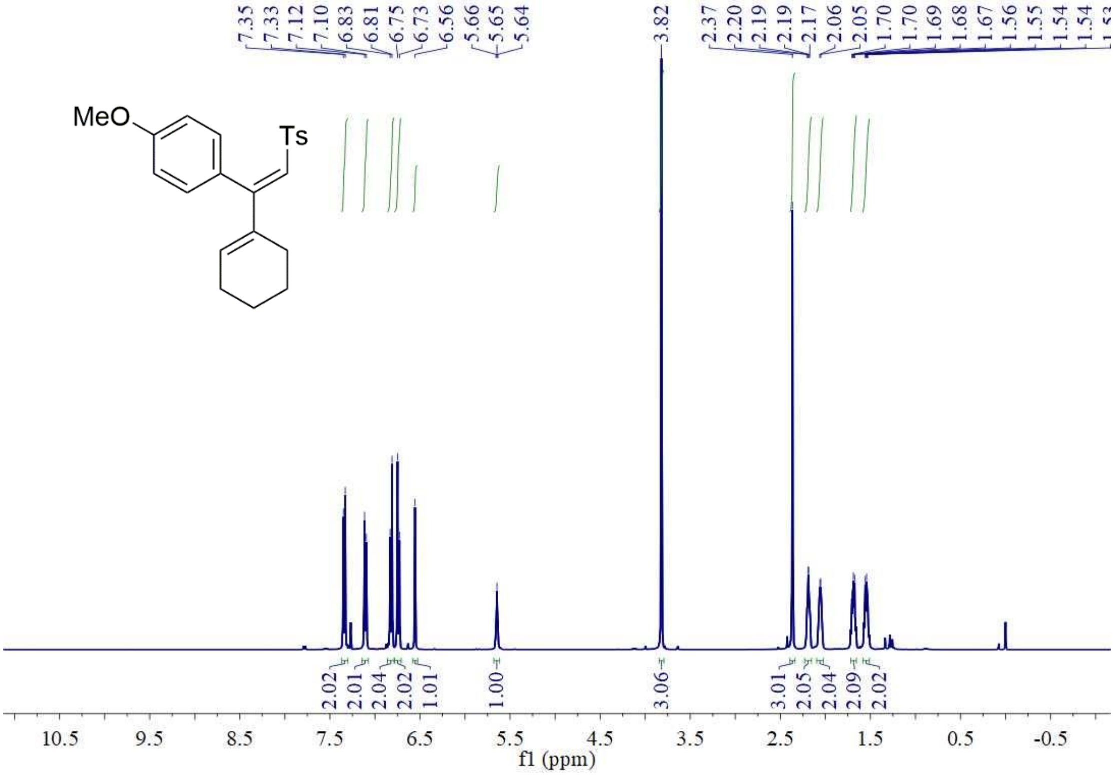

Supplementary Figure 112: ^{1}H NMR of 24b (400 MHz, $CDCl_3$).

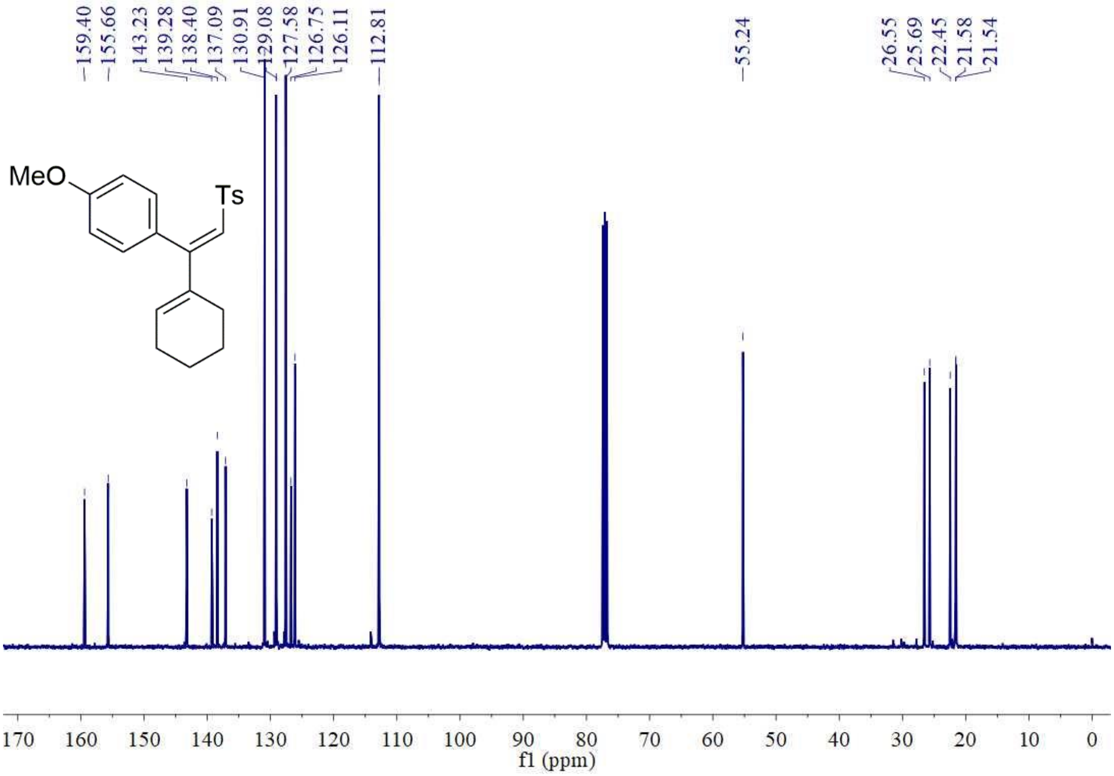

Supplementary Figure 113: ^{13}C NMR of 24b (100 MHz, $CDCl_3$).

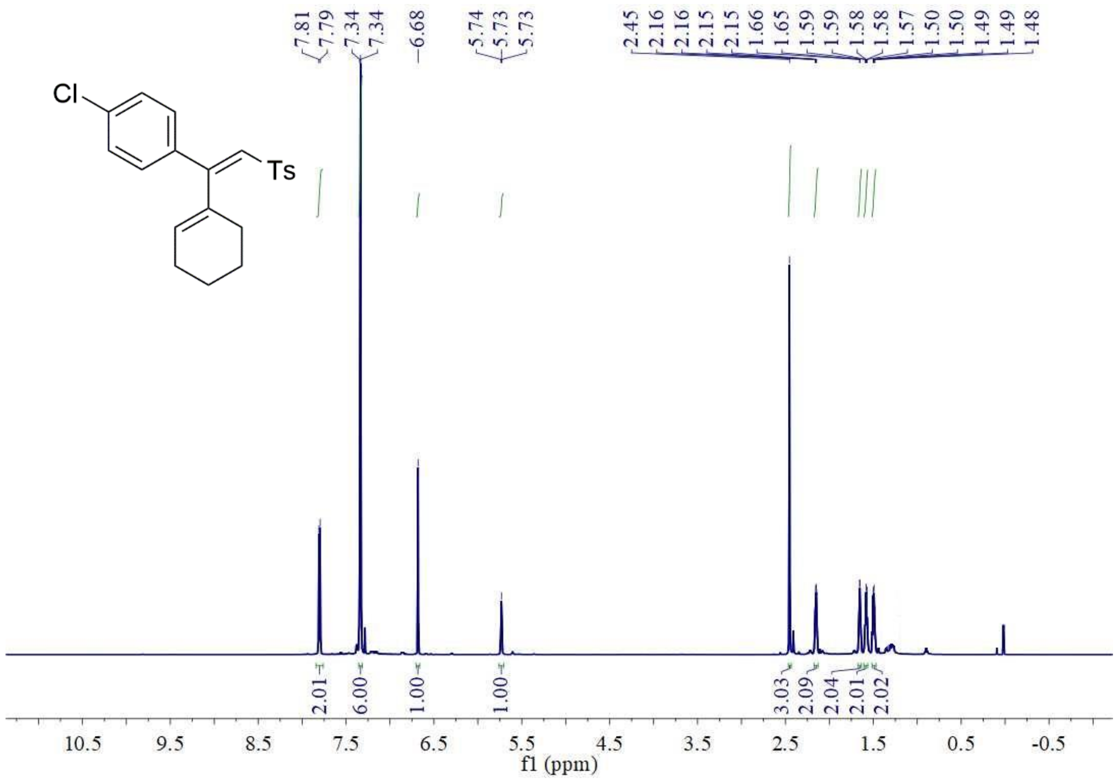

Supplementary Figure 114: ^{1}H NMR of 25a (600 MHz, $CDCl_3$).

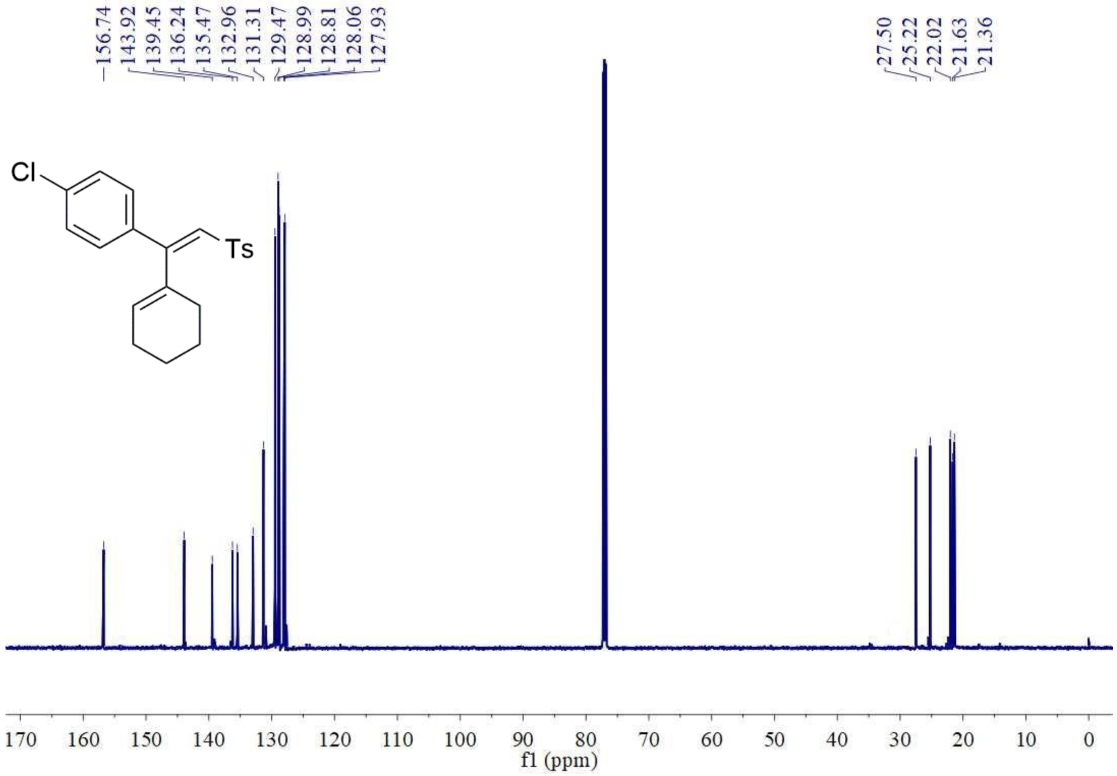

Supplementary Figure 115: ^{13}C NMR of 25a (150 MHz, $CDCl_3$).

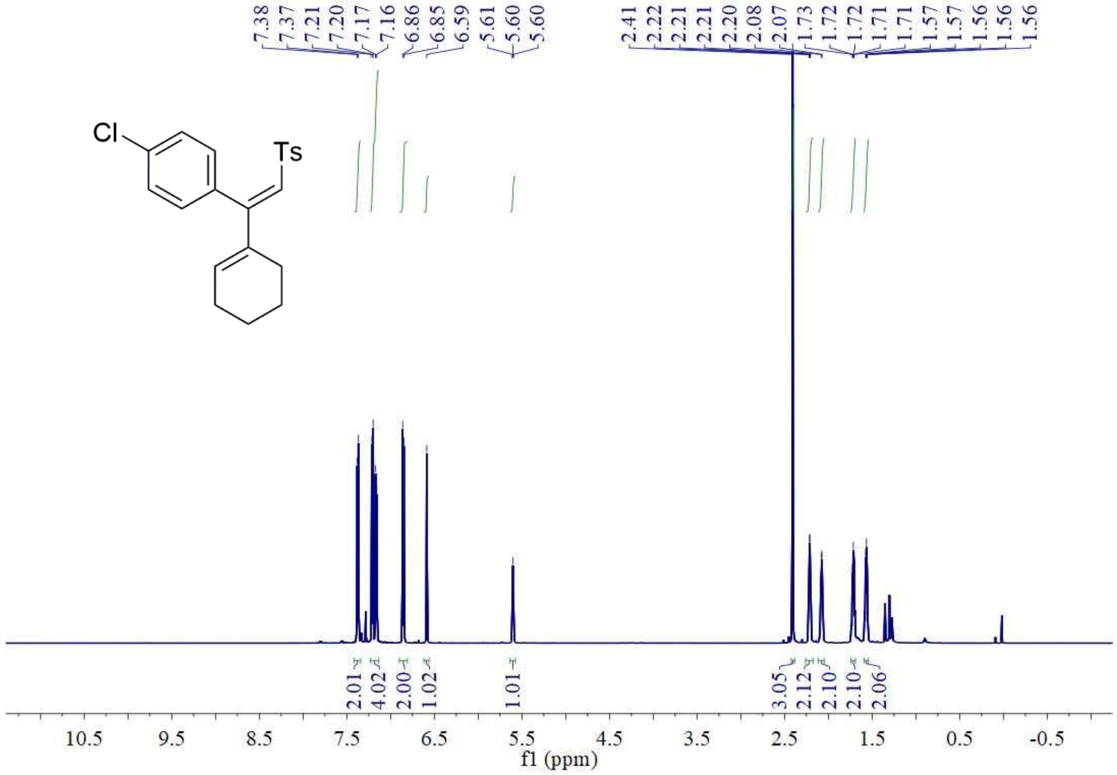

Supplementary Figure 116: ^{1}H NMR of 25b (600 MHz, $CDCl_3$).

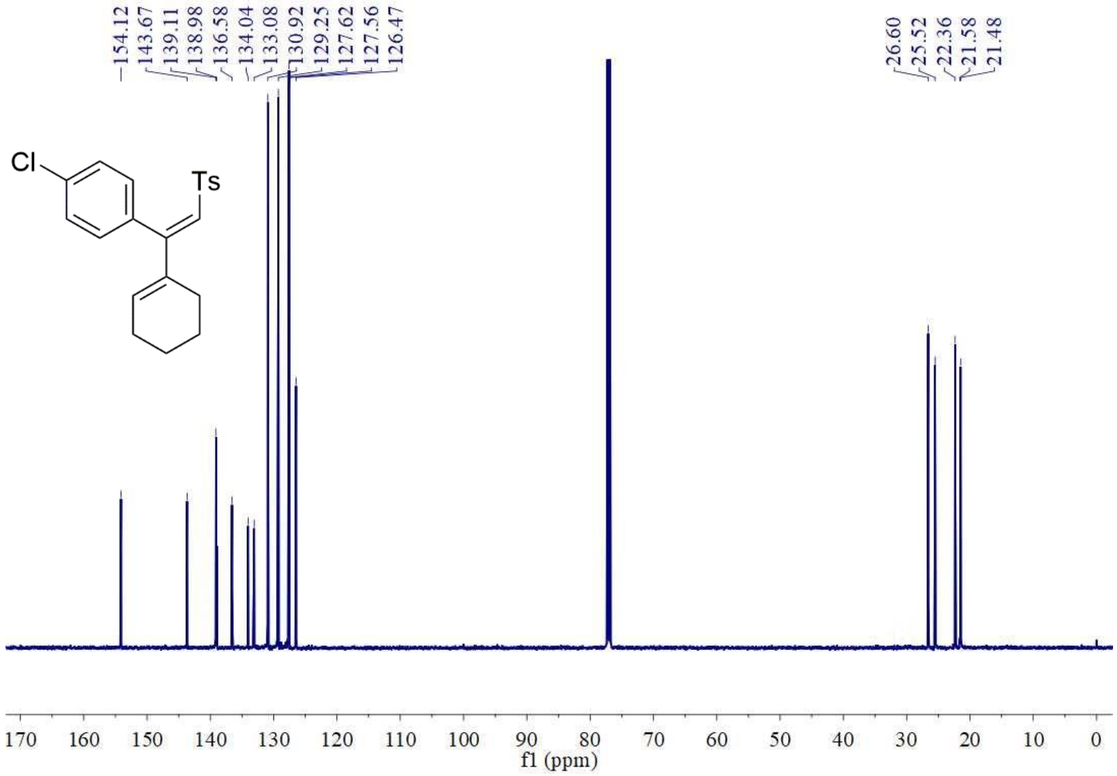

Supplementary Figure 117: ^{13}C NMR of 25b (150 MHz, $CDCl_3$).

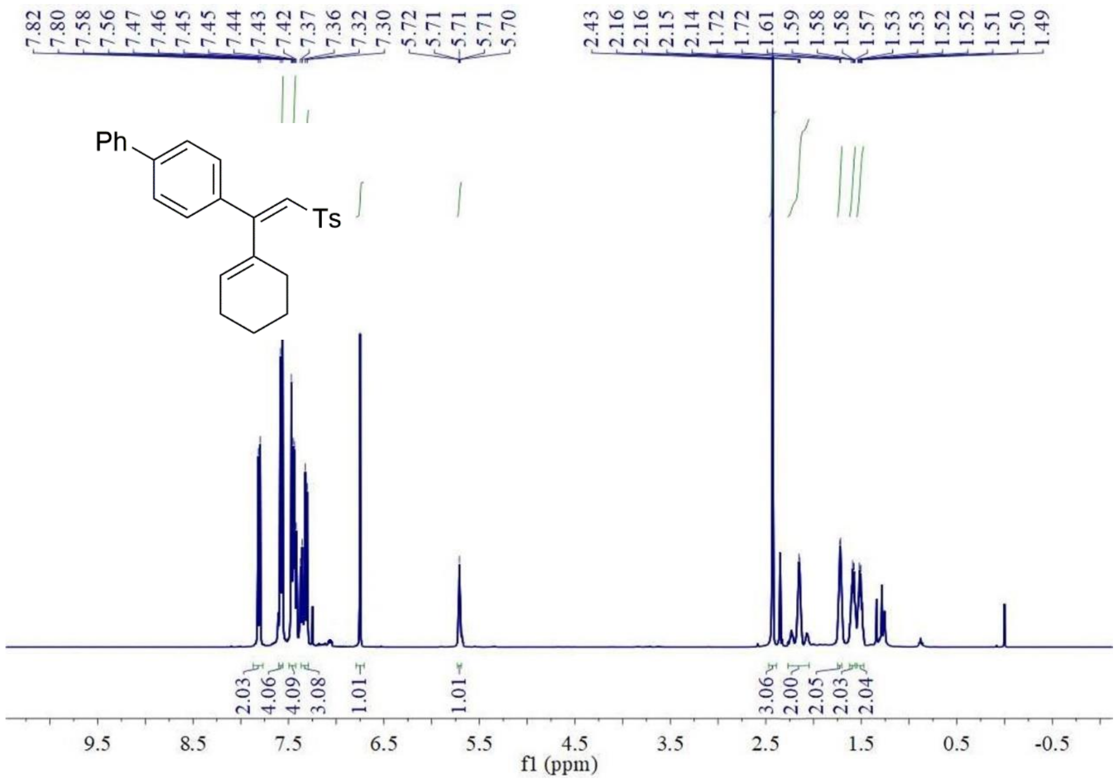

Supplementary Figure 118: ^{1}H NMR of 26a (400 MHz, $CDCl_3$).

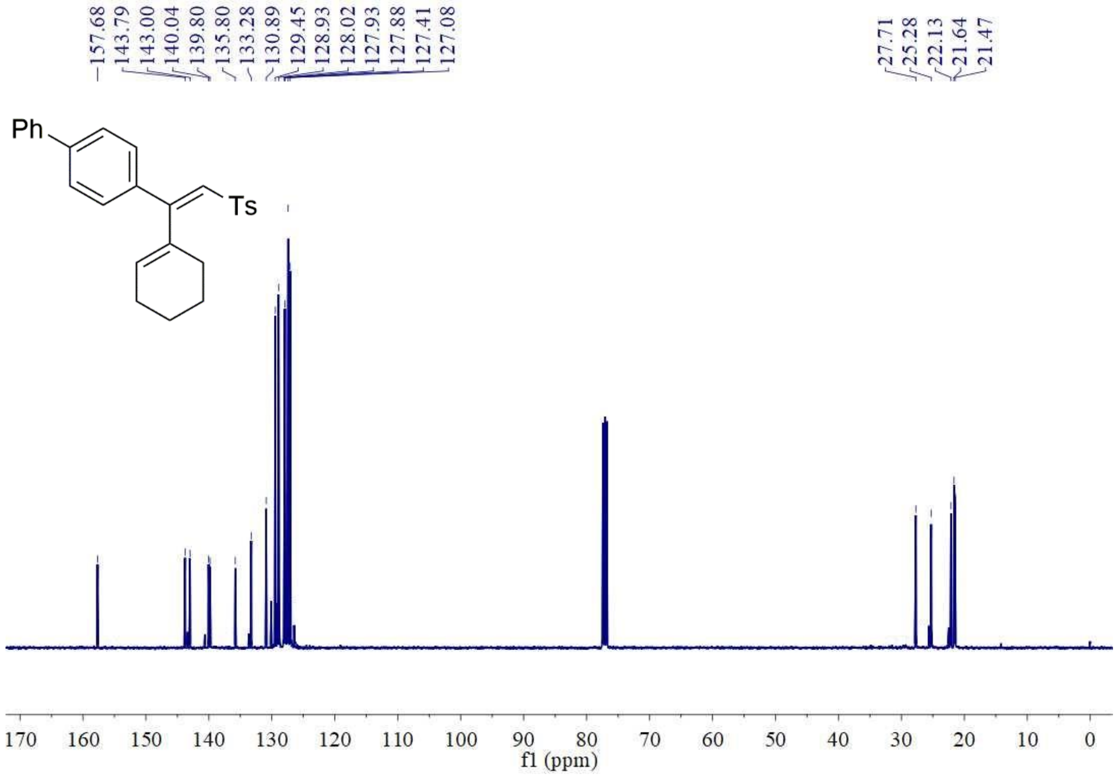

Supplementary Figure 119: ^{13}C NMR of 26a (100 MHz, $CDCl_3$).

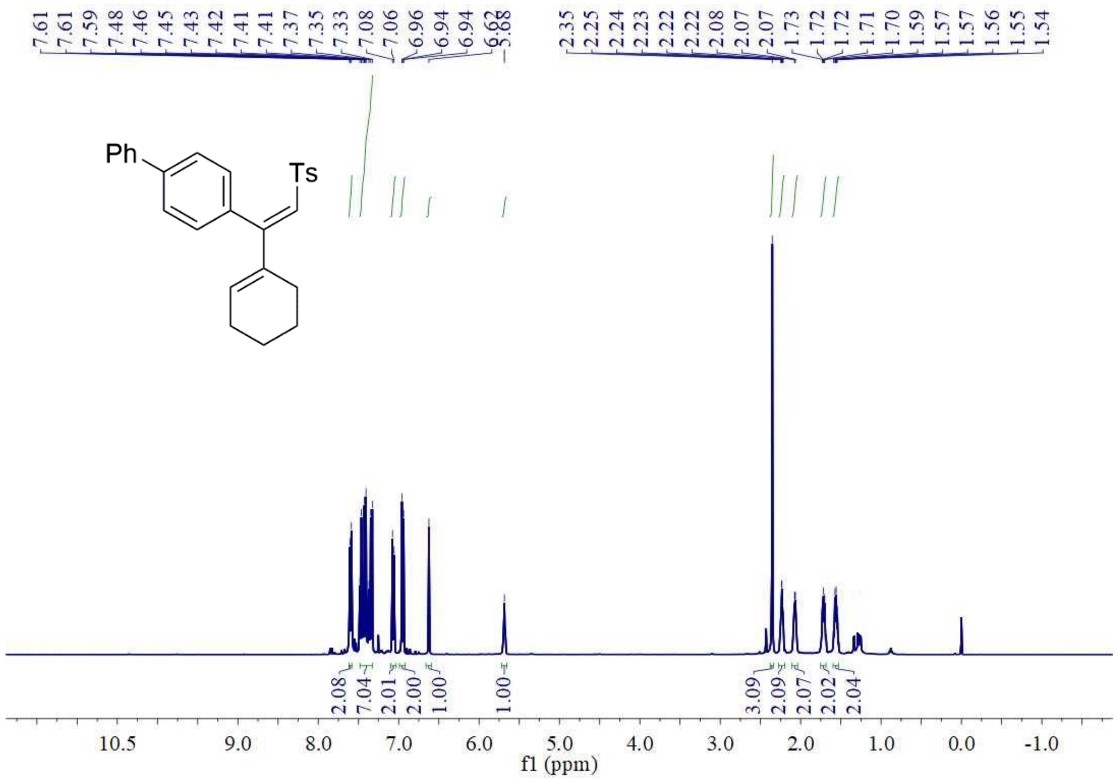

Supplementary Figure 120: ^{1}H NMR of 26b (400 MHz, $CDCl_3$).

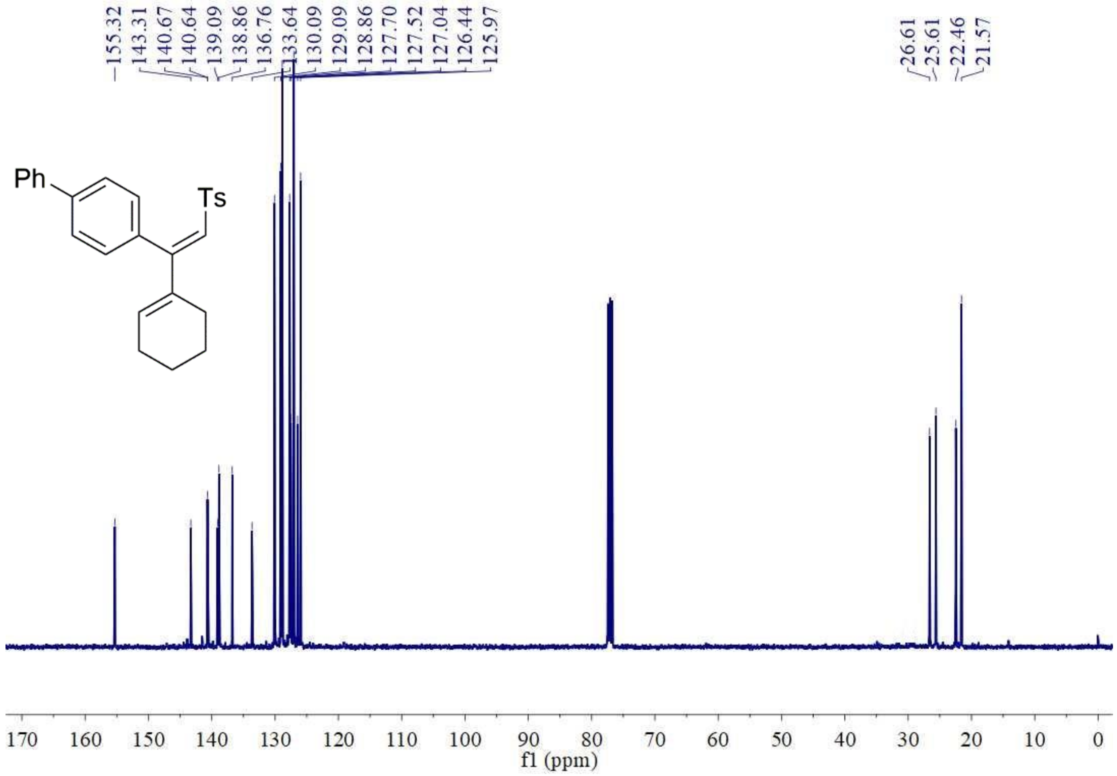

Supplementary Figure 121: ^{13}C NMR of 26b (100 MHz, $CDCl_3$).

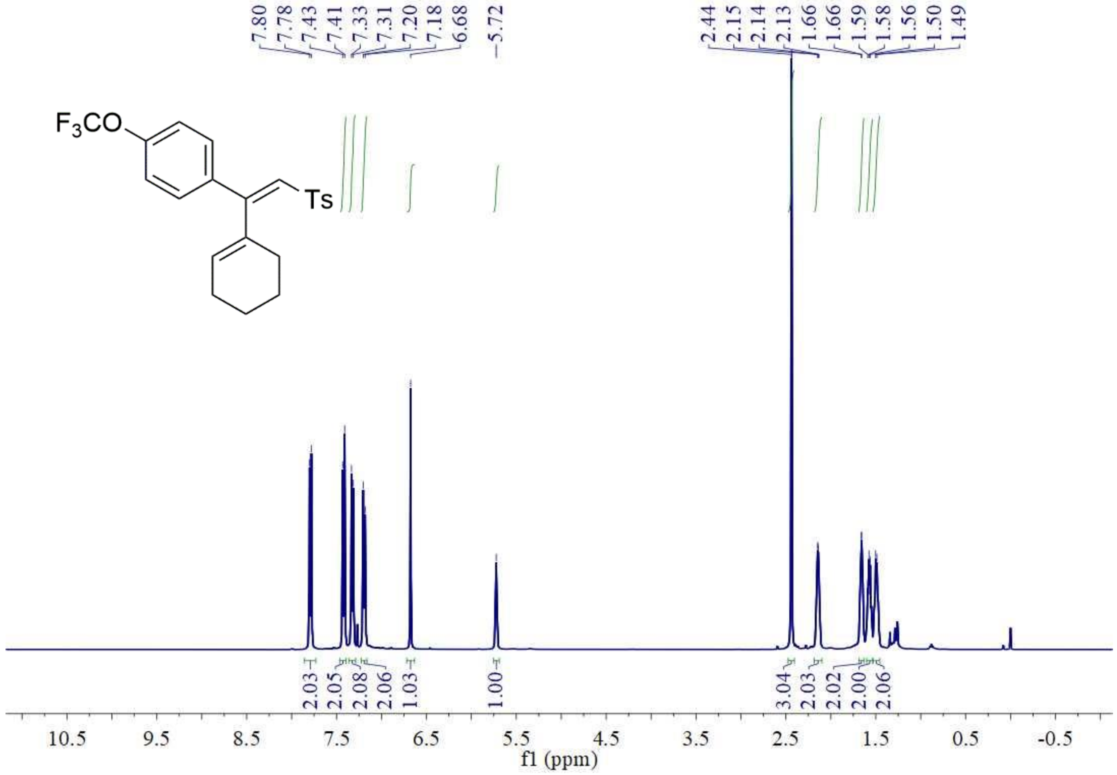

Supplementary Figure 122: ^{1}H NMR of 27a (400 MHz, $CDCl_3$).

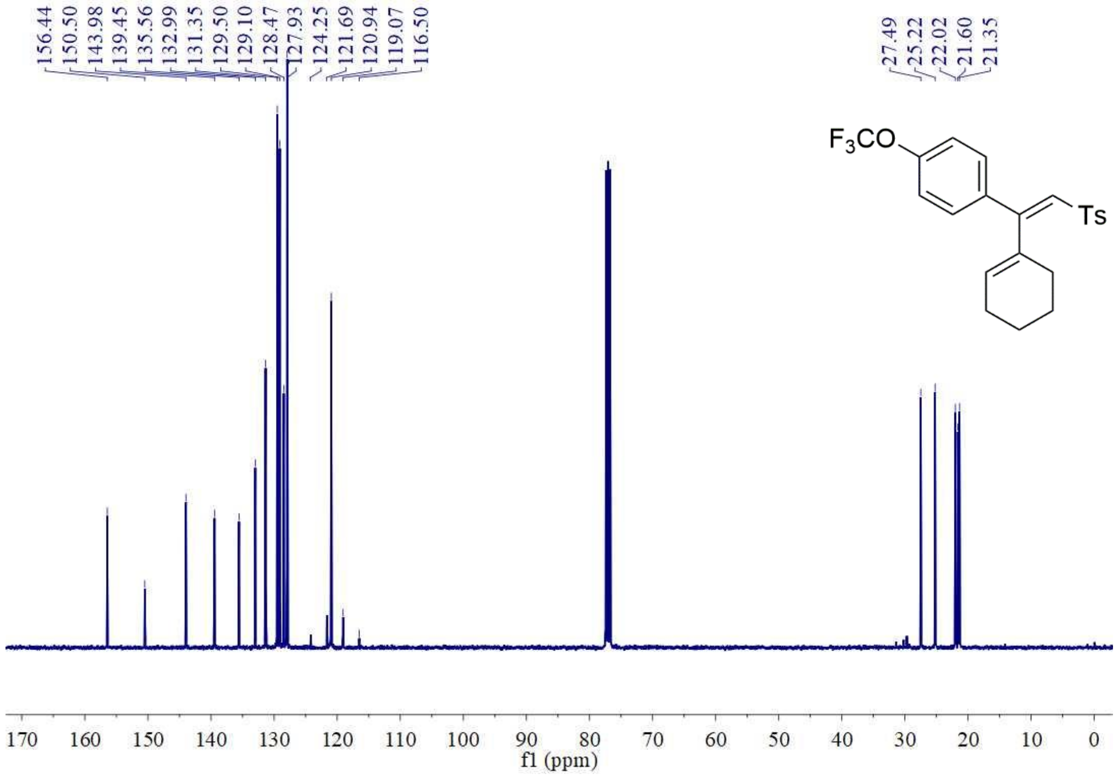

Supplementary Figure 123: ^{13}C NMR of 27a (100 MHz, $CDCl_3$).

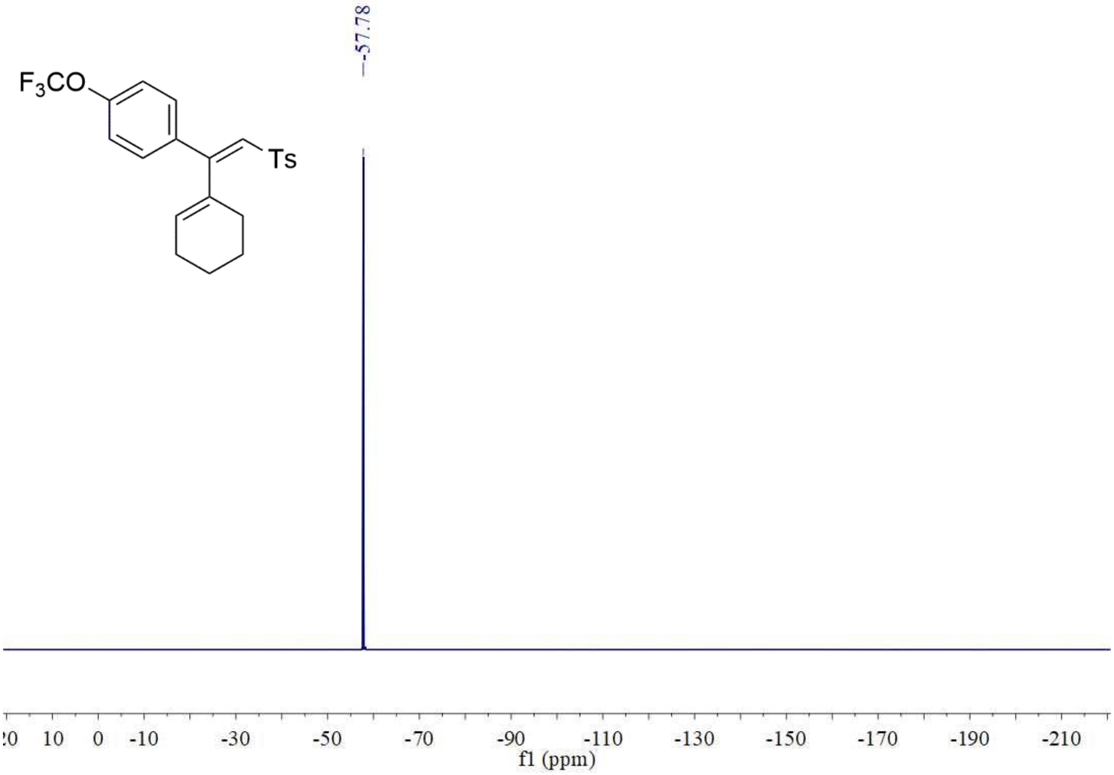

Supplementary Figure 124: ^{19}F NMR of 27a (377 MHz, $CDCl_3$).

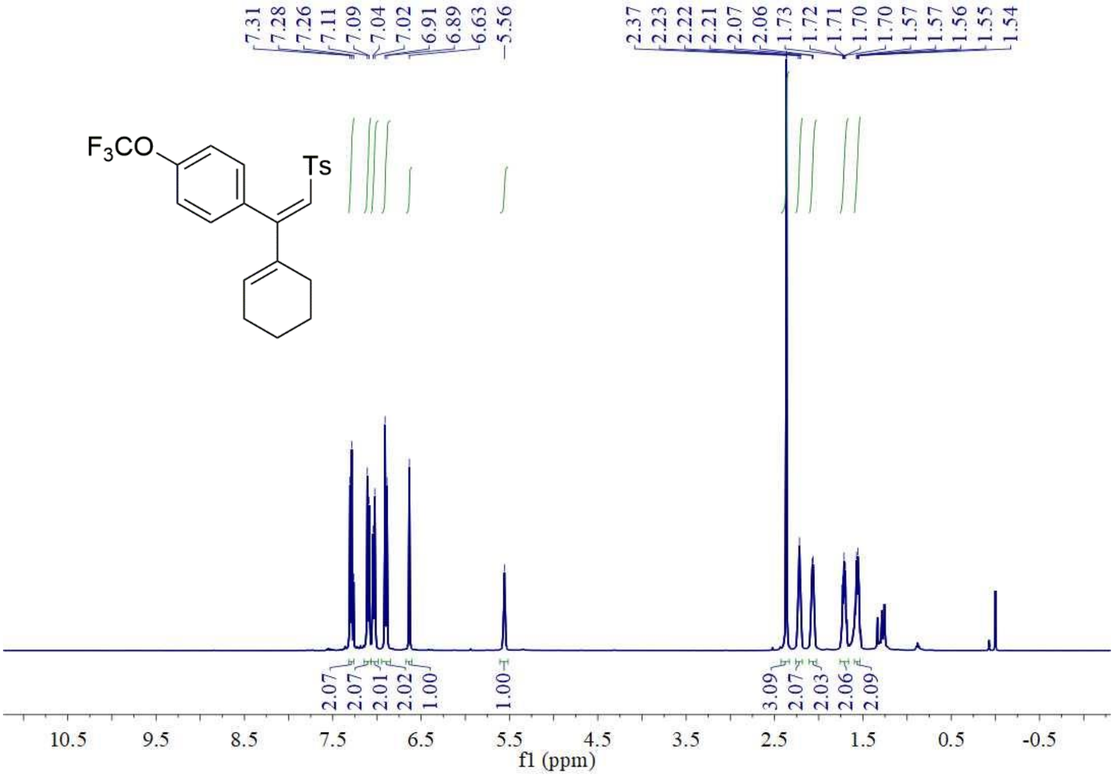

Supplementary Figure 125: ^{1}H NMR of 27b (400 MHz, $CDCl_3$).

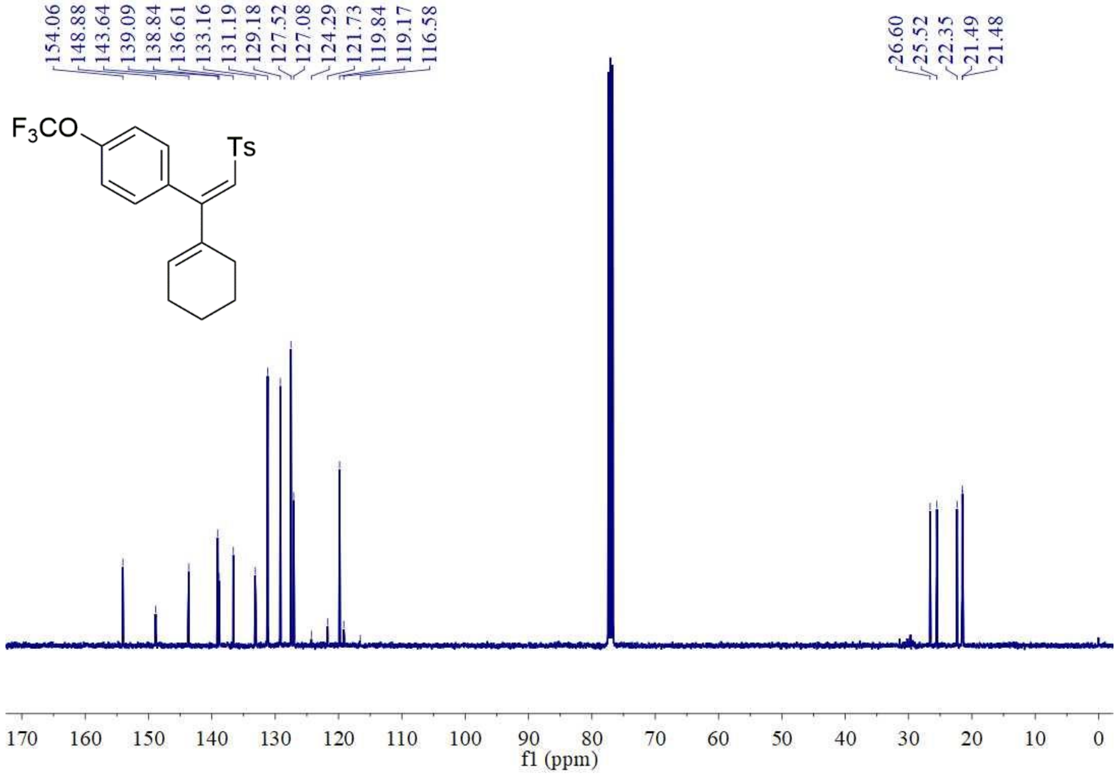

Supplementary Figure 126: ^{13}C NMR of 27b (100 MHz, $CDCl_3$).

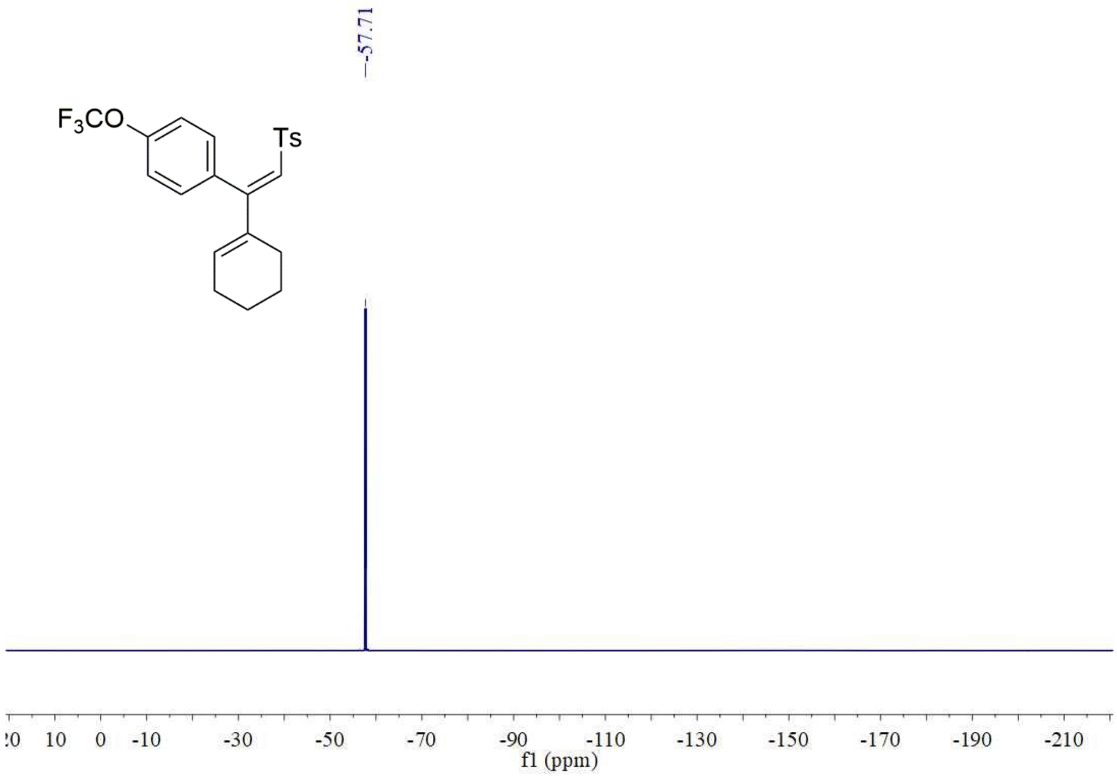

Supplementary Figure 127: ^{19}F NMR of 27b (377 MHz, $CDCl_3$).

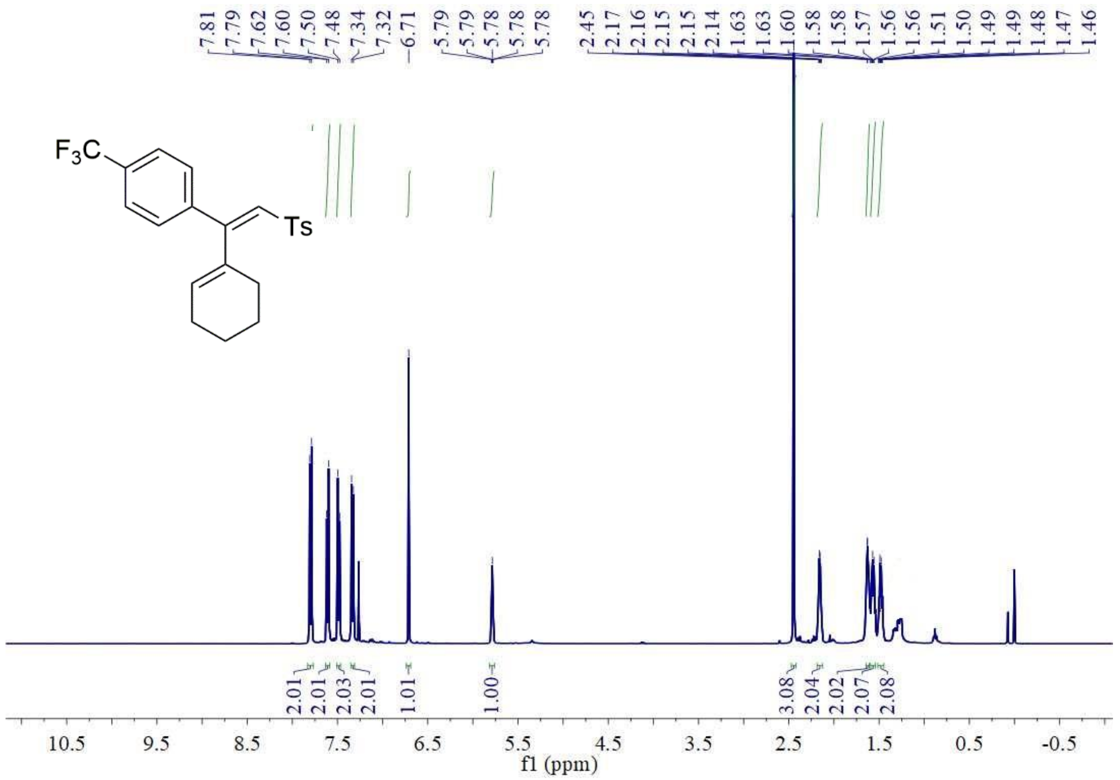

Supplementary Figure 128: ^{1}H NMR of 28a (400 MHz, $CDCl_3$).

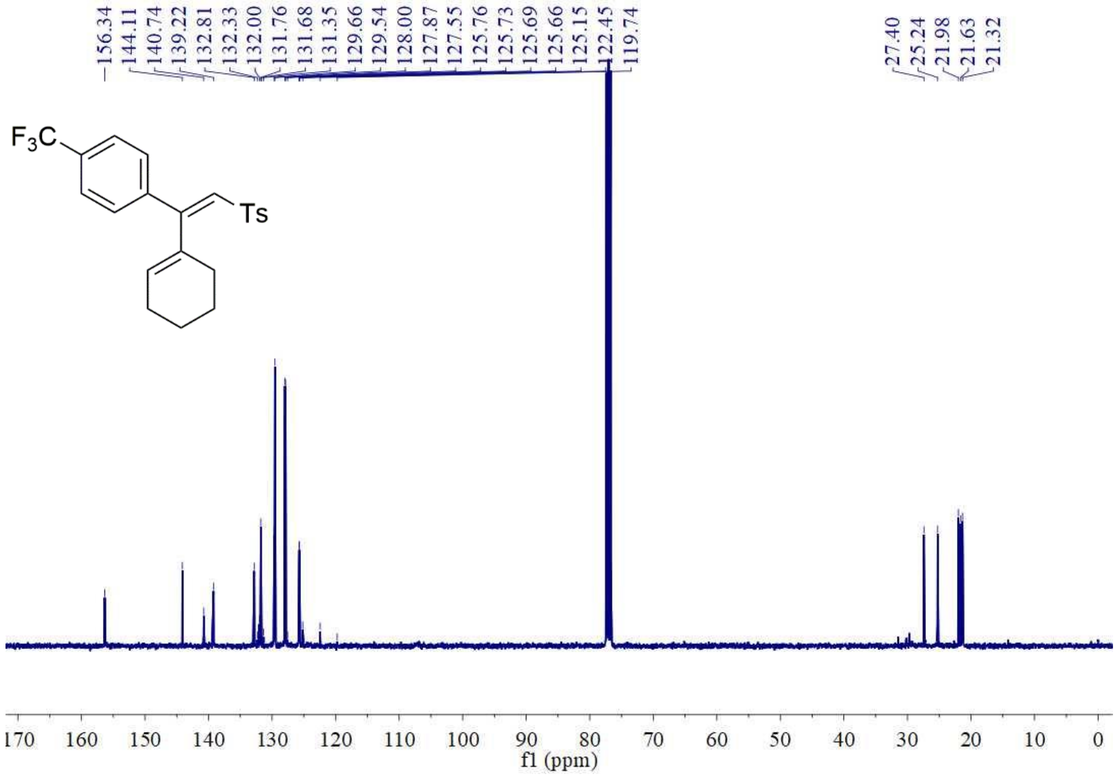

Supplementary Figure 129: ^{13}C NMR of 28a (100 MHz, $CDCl_3$).

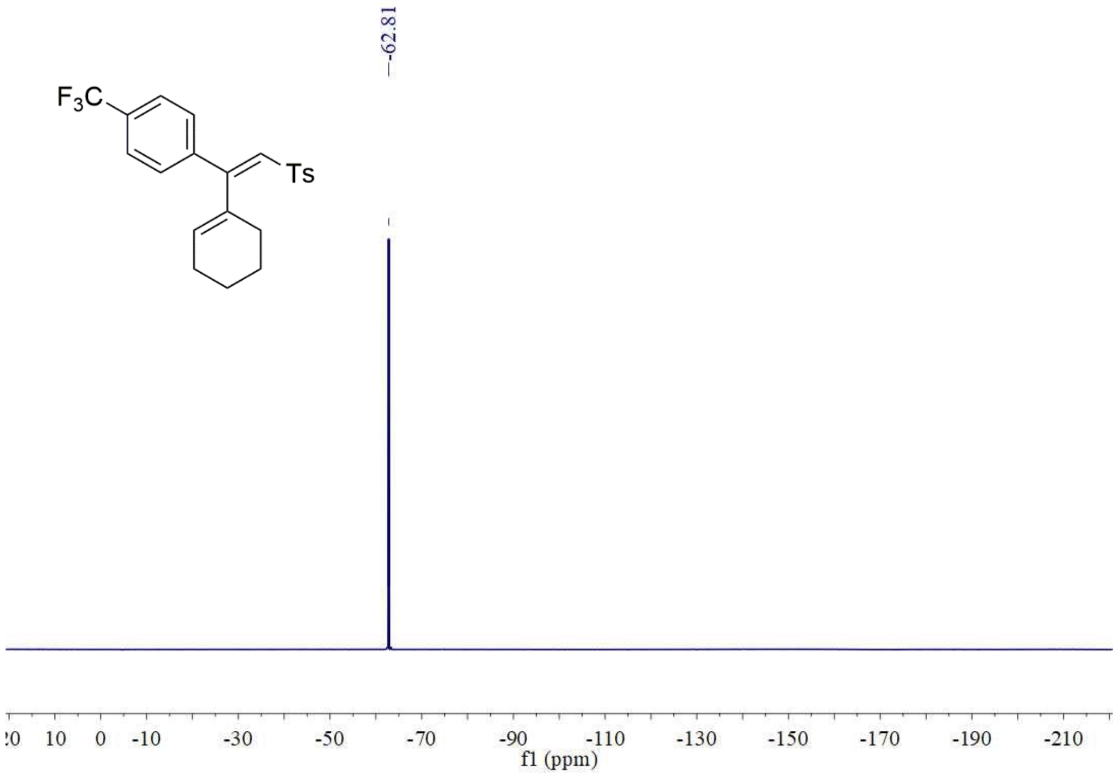

Supplementary Figure 130: ^{19}F NMR of 28a (377 MHz, $CDCl_3$).

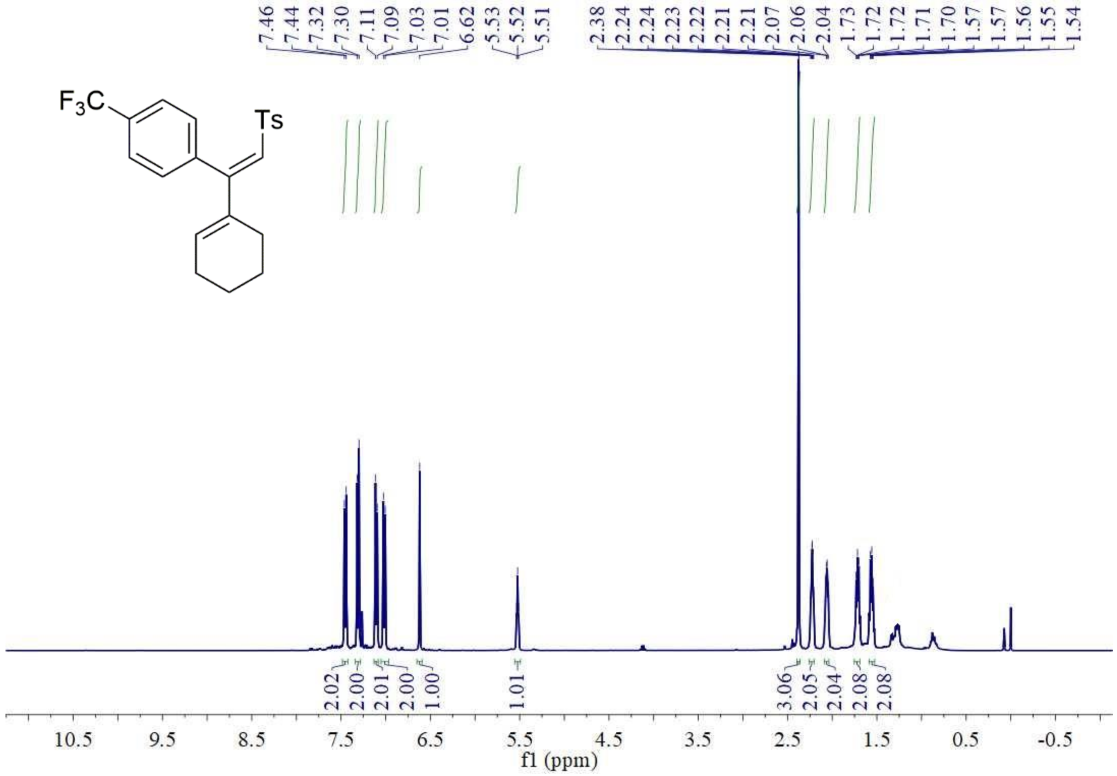

Supplementary Figure 131: ^{1}H NMR of 28b (400 MHz, $CDCl_3$).

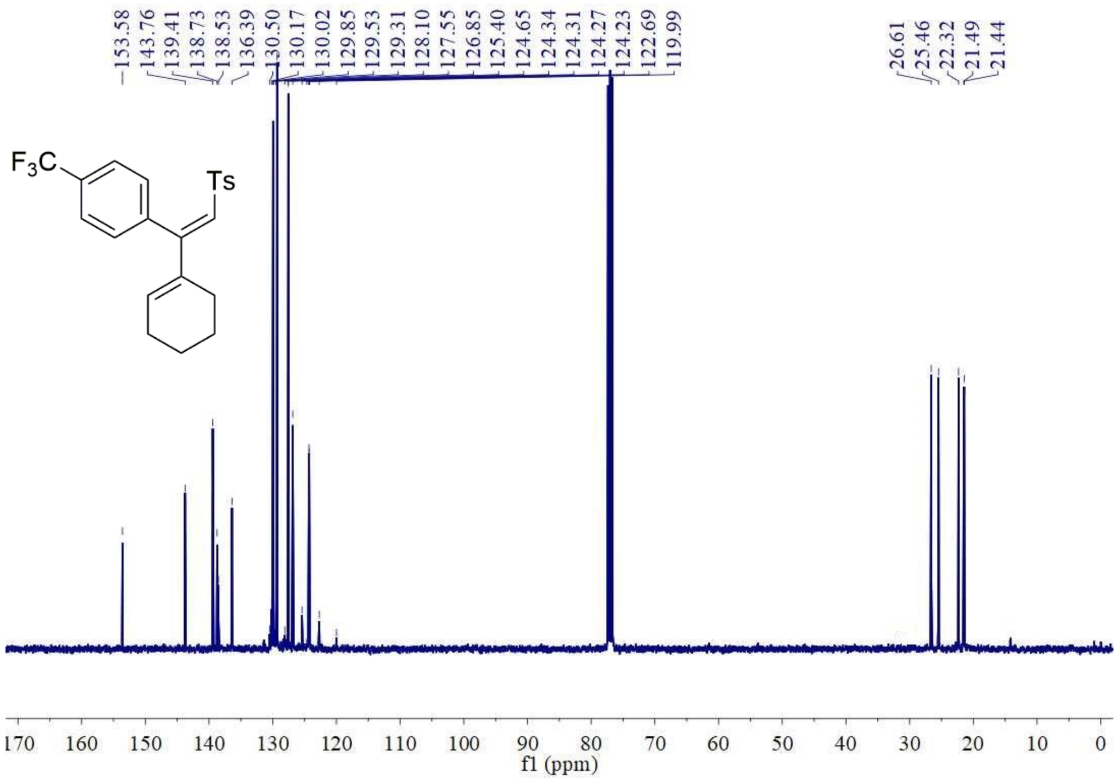

Supplementary Figure 132: ^{13}C NMR of 28b (100 MHz, $CDCl_3$).

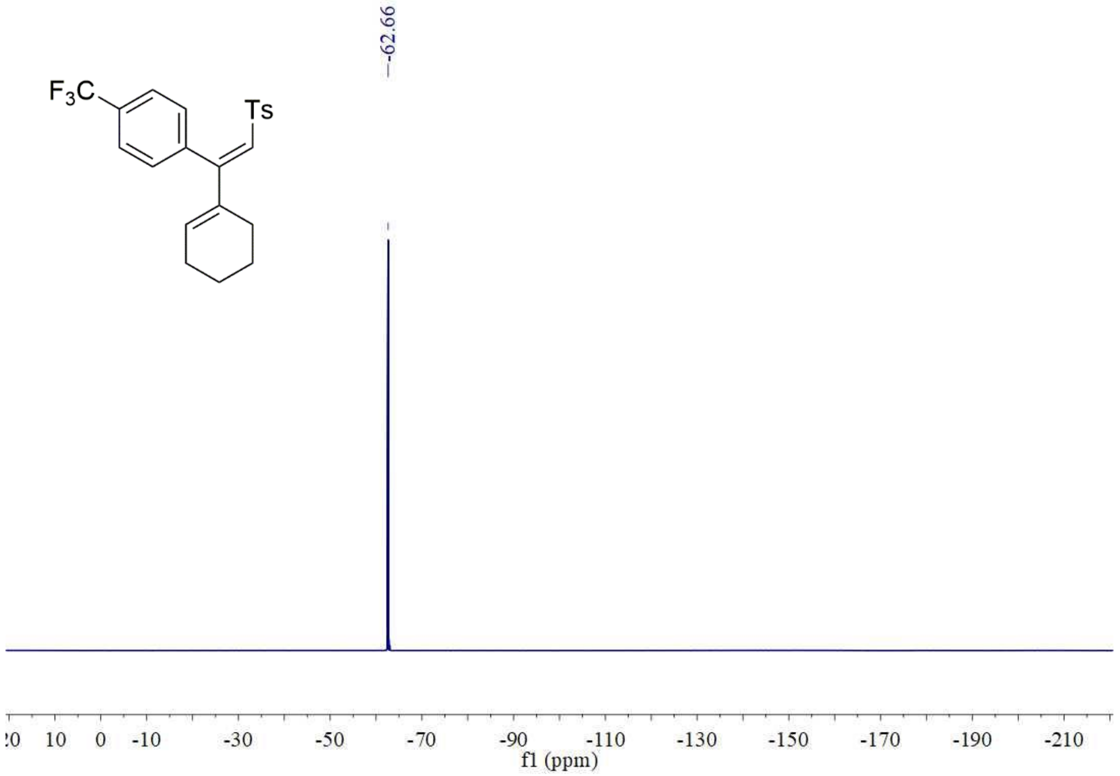

Supplementary Figure 133: ^{19}F NMR of 28b (377 MHz, $CDCl_3$).

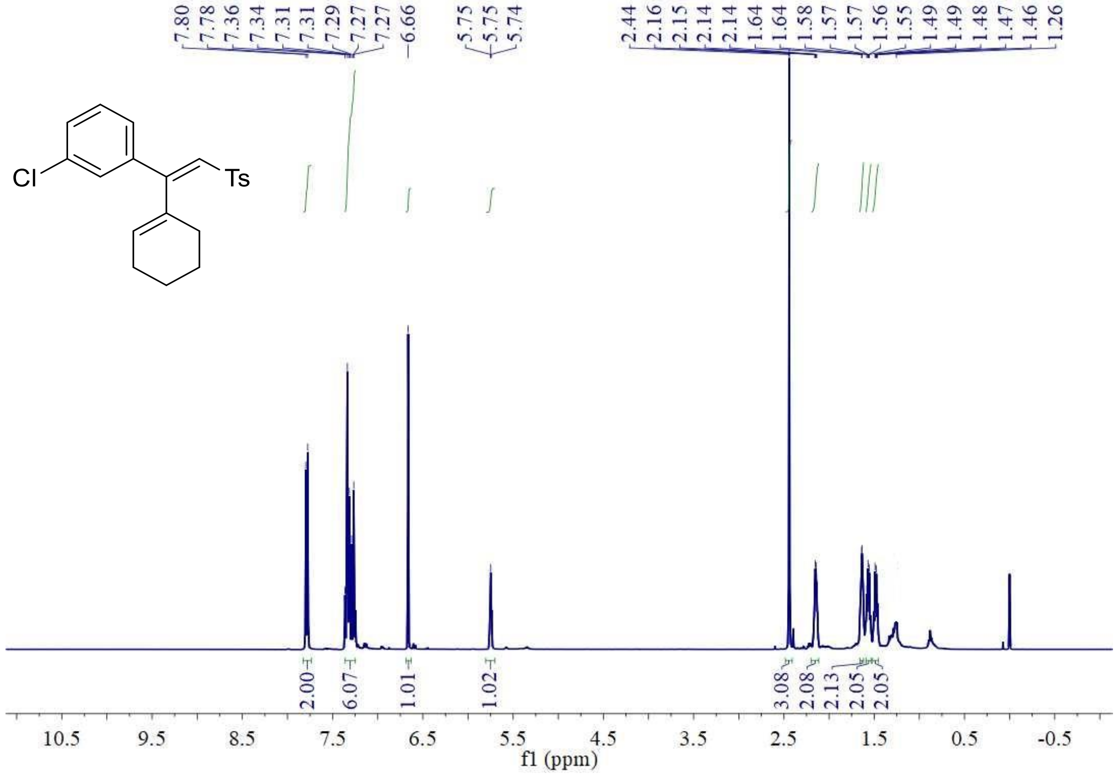

Supplementary Figure 134: ^{1}H NMR of 29a (400 MHz, $CDCl_3$).

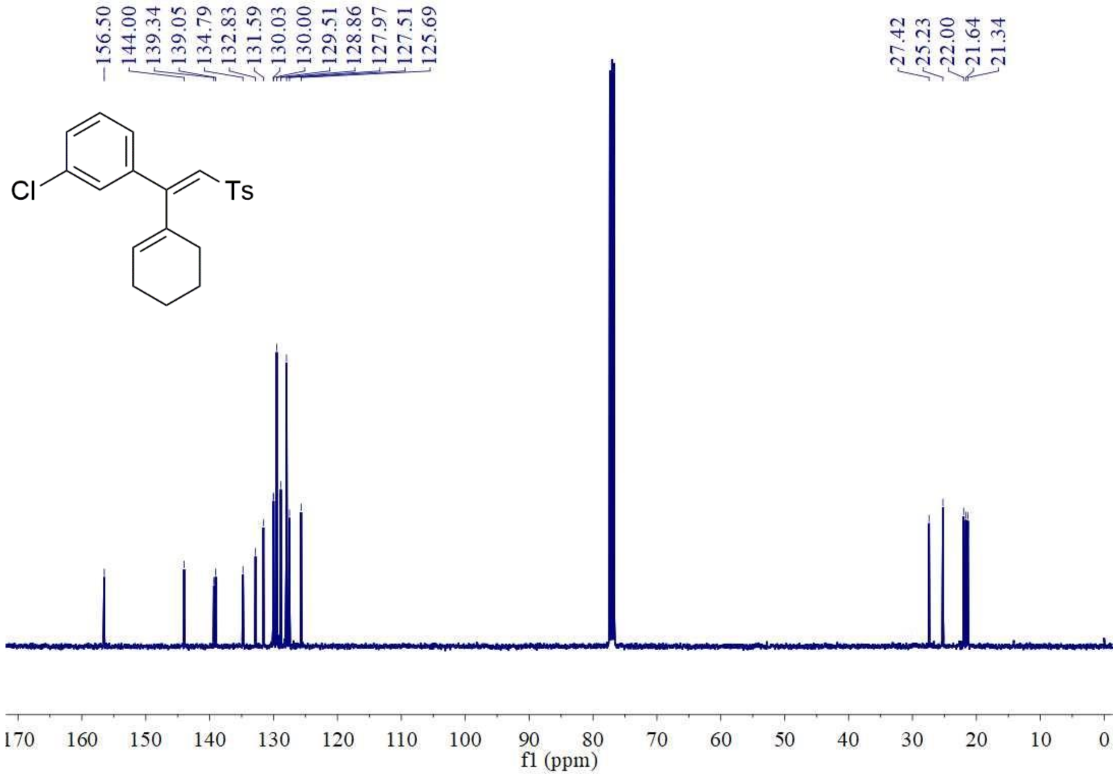

Supplementary Figure 135: ^{13}C NMR of 29a (100 MHz, $CDCl_3$).

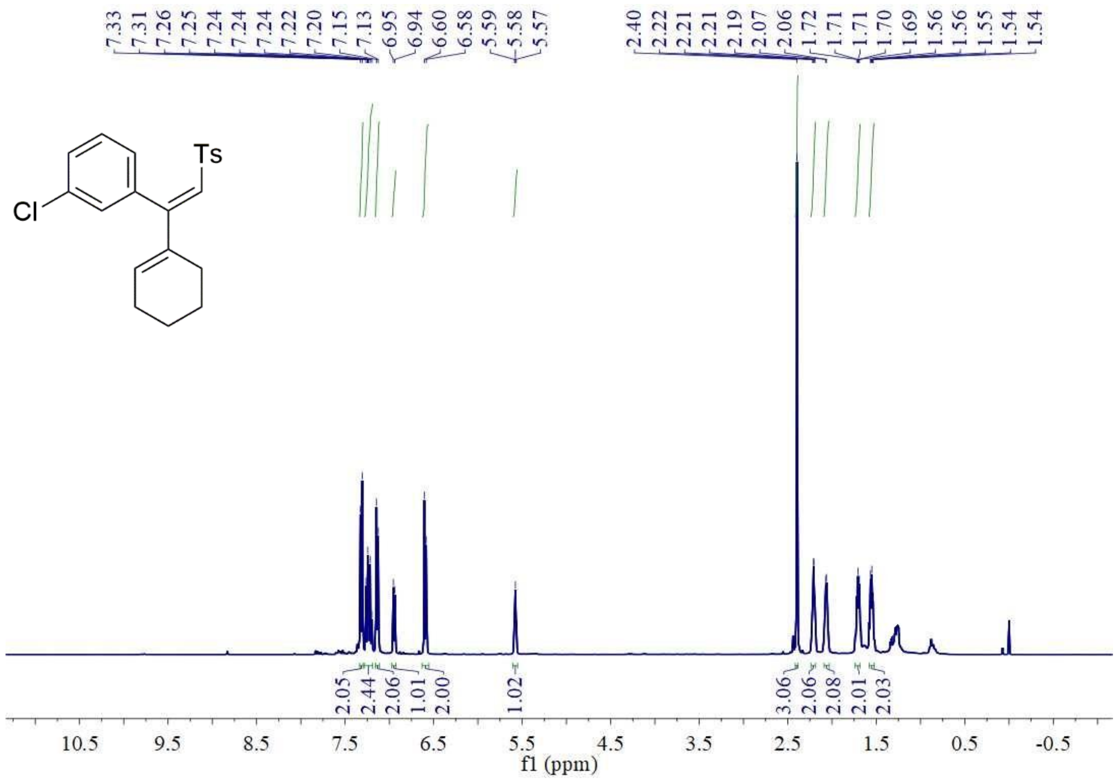

Supplementary Figure 136: ^{1}H NMR of 29b (400 MHz, $CDCl_3$).

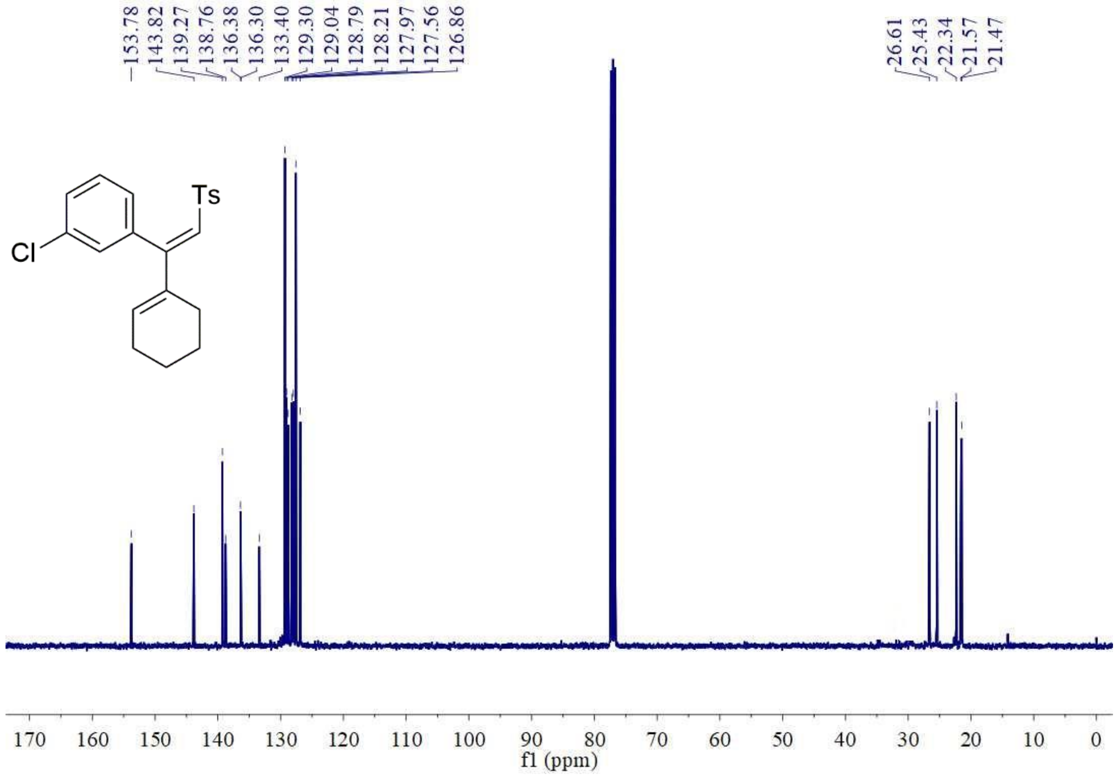

Supplementary Figure 137: ^{13}C NMR of 29b (100 MHz, $CDCl_3$).

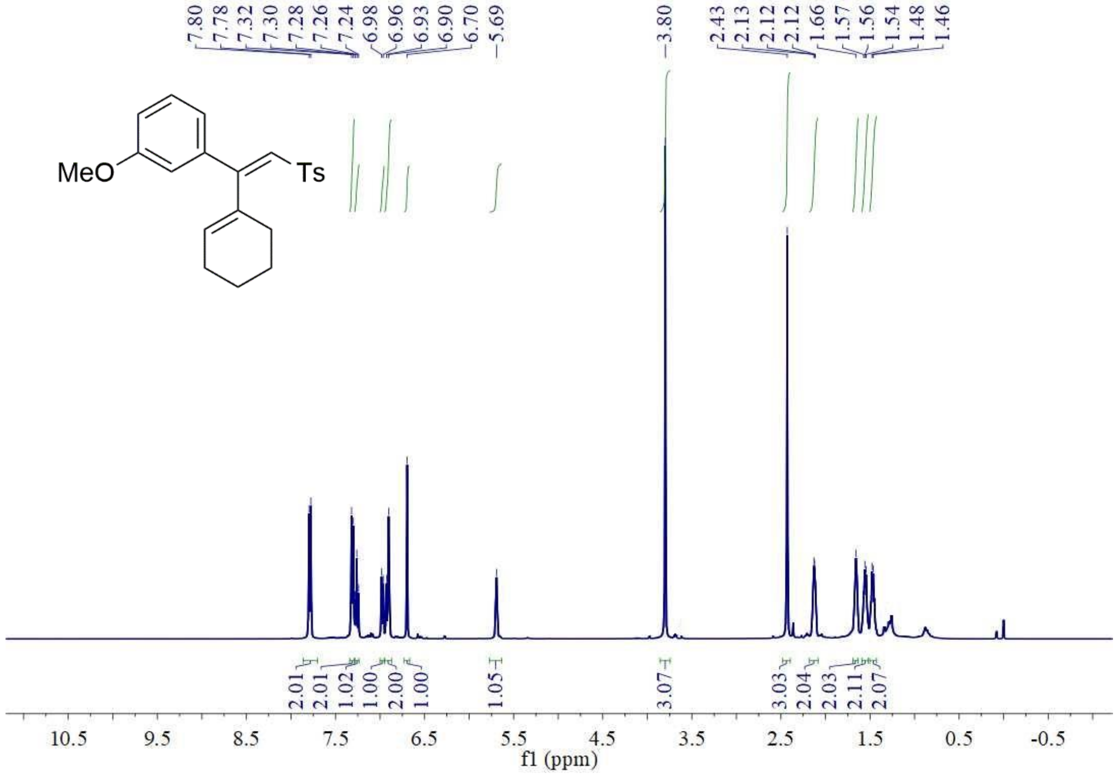

Supplementary Figure 138: ^{1}H NMR of 30a (400 MHz, $CDCl_3$).

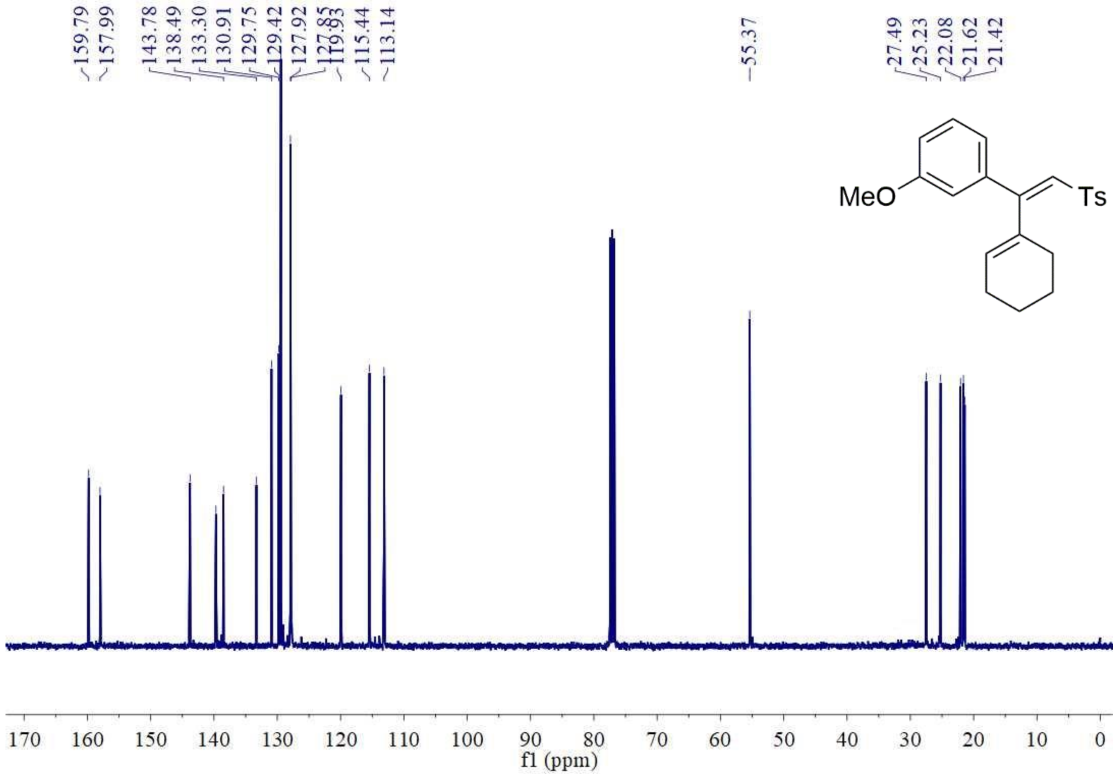

Supplementary Figure 139: ^{13}C NMR of 30a (100 MHz, $CDCl_3$).

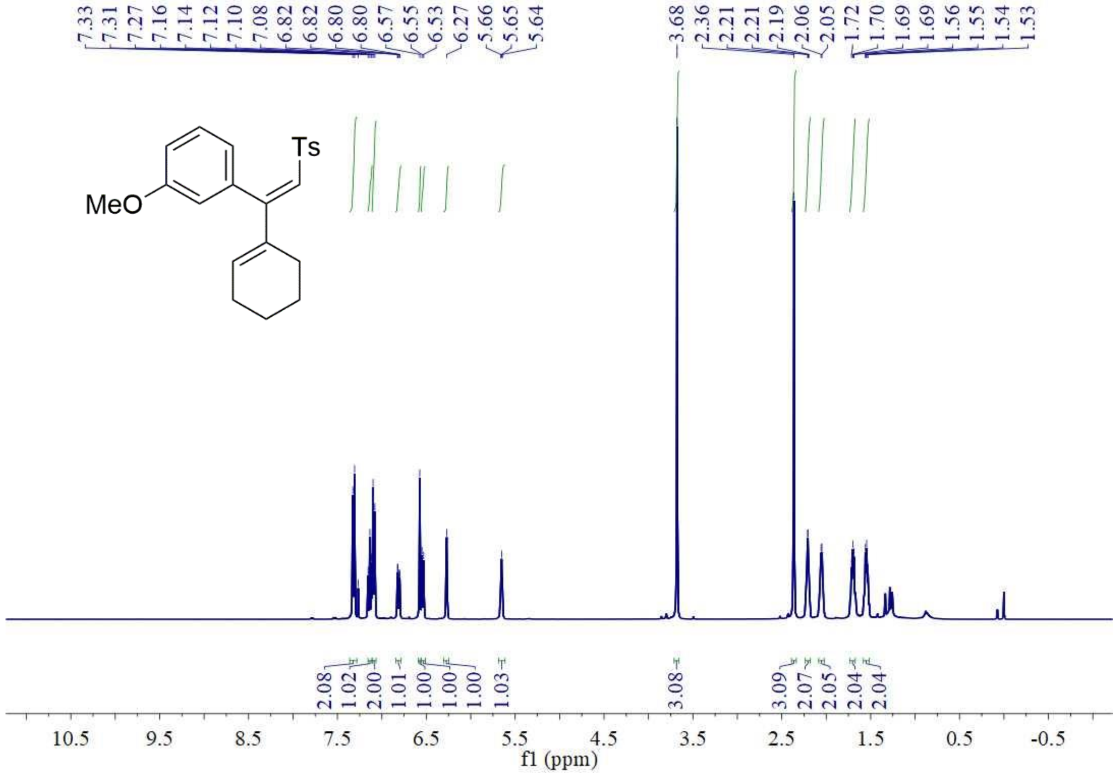

Supplementary Figure 140: ^{1}H NMR of 30b (400 MHz, $CDCl_3$).

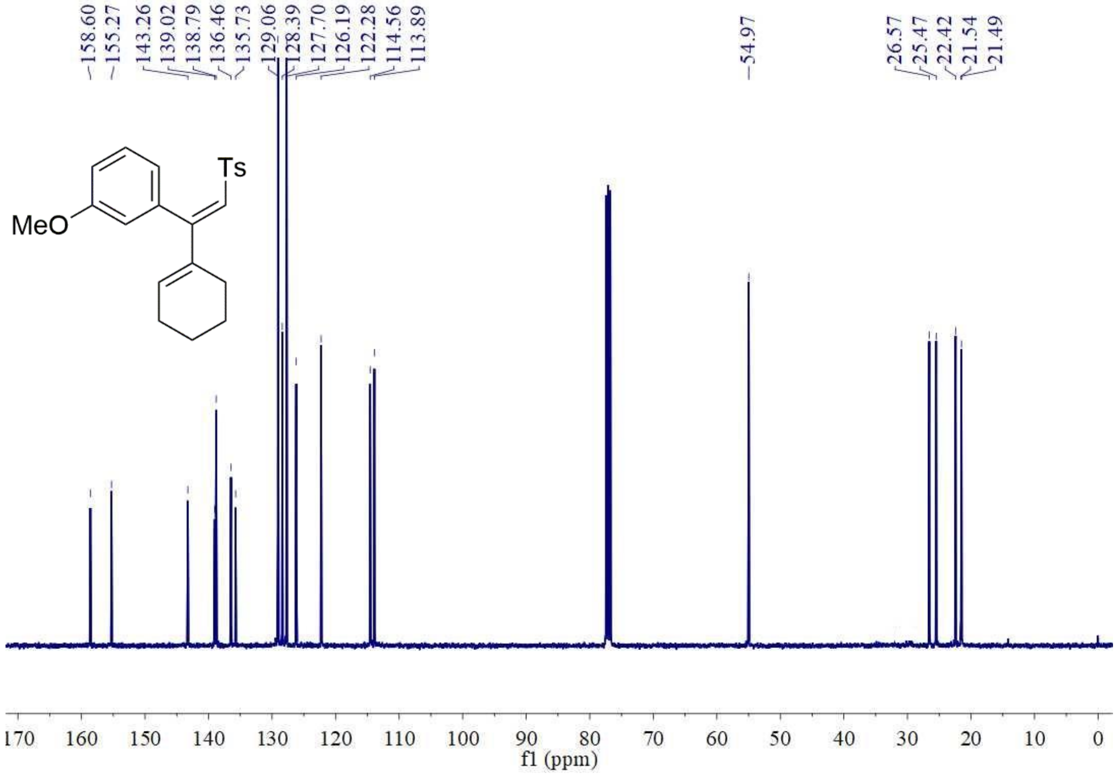

Supplementary Figure 141: ^{13}C NMR of 30b (100 MHz, $CDCl_3$).

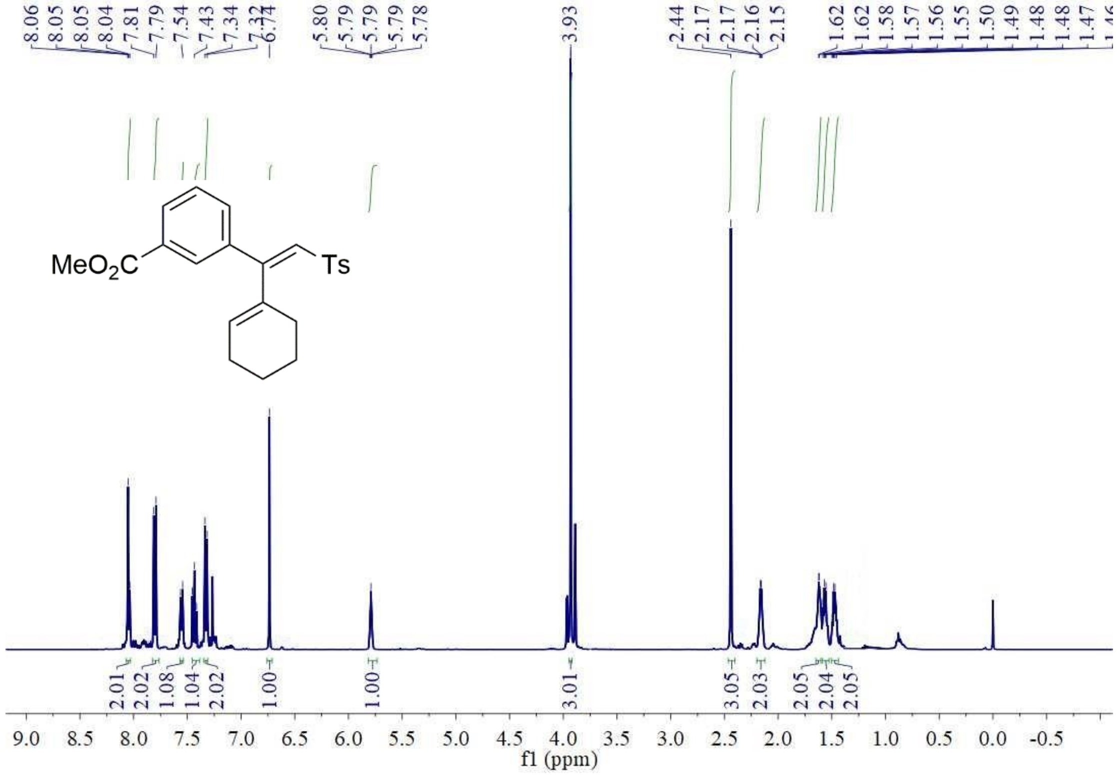

Supplementary Figure 142: ^{1}H NMR of 31a (400 MHz, $CDCl_3$).

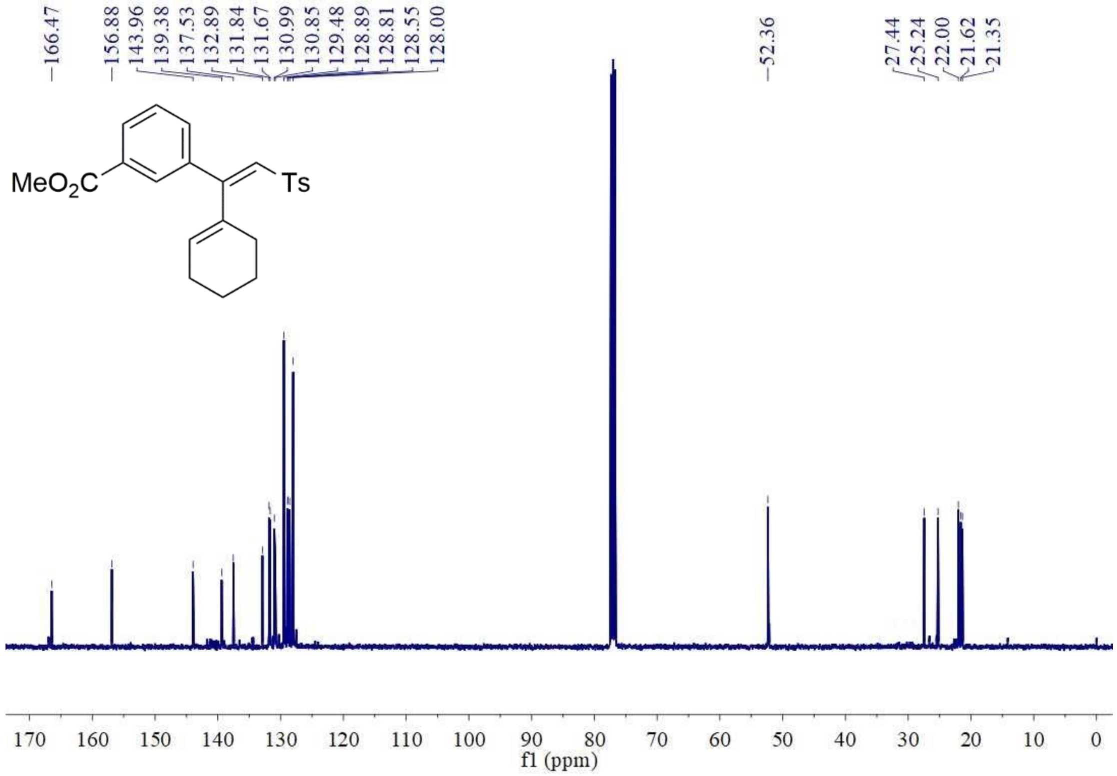

Supplementary Figure 143: ^{13}C NMR of 31a (100 MHz, $CDCl_3$).

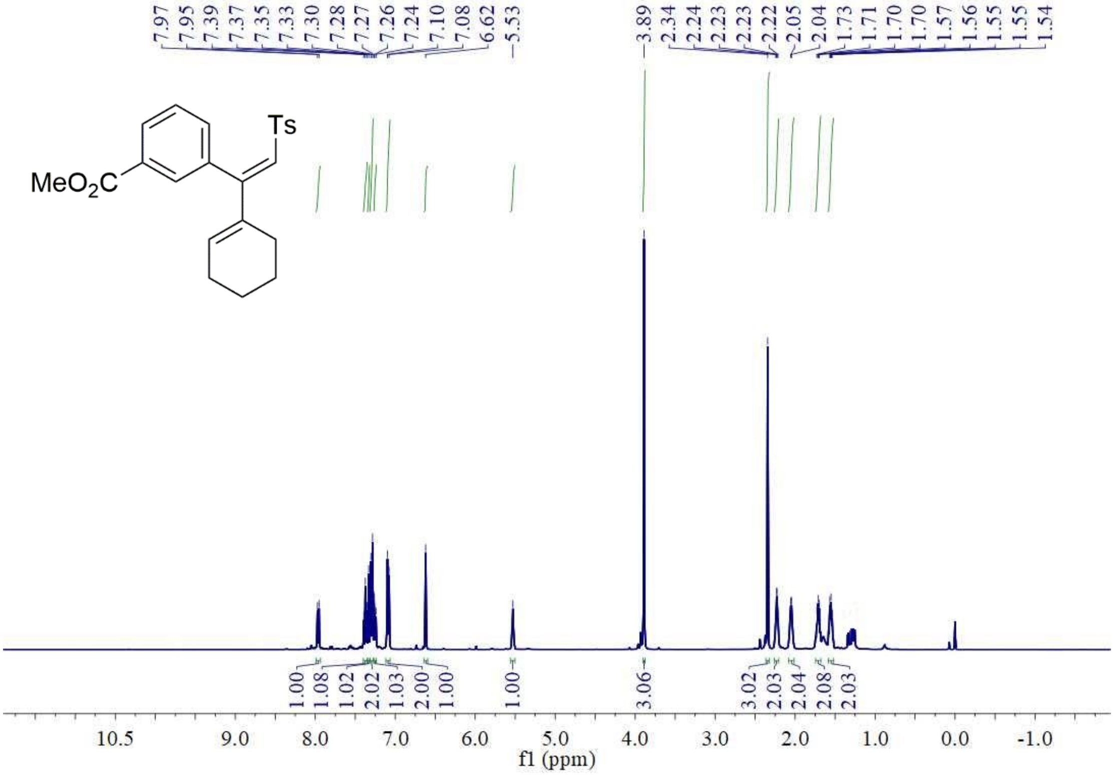

Supplementary Figure 144: ^{1}H NMR of 31b (400 MHz, CDCl3).

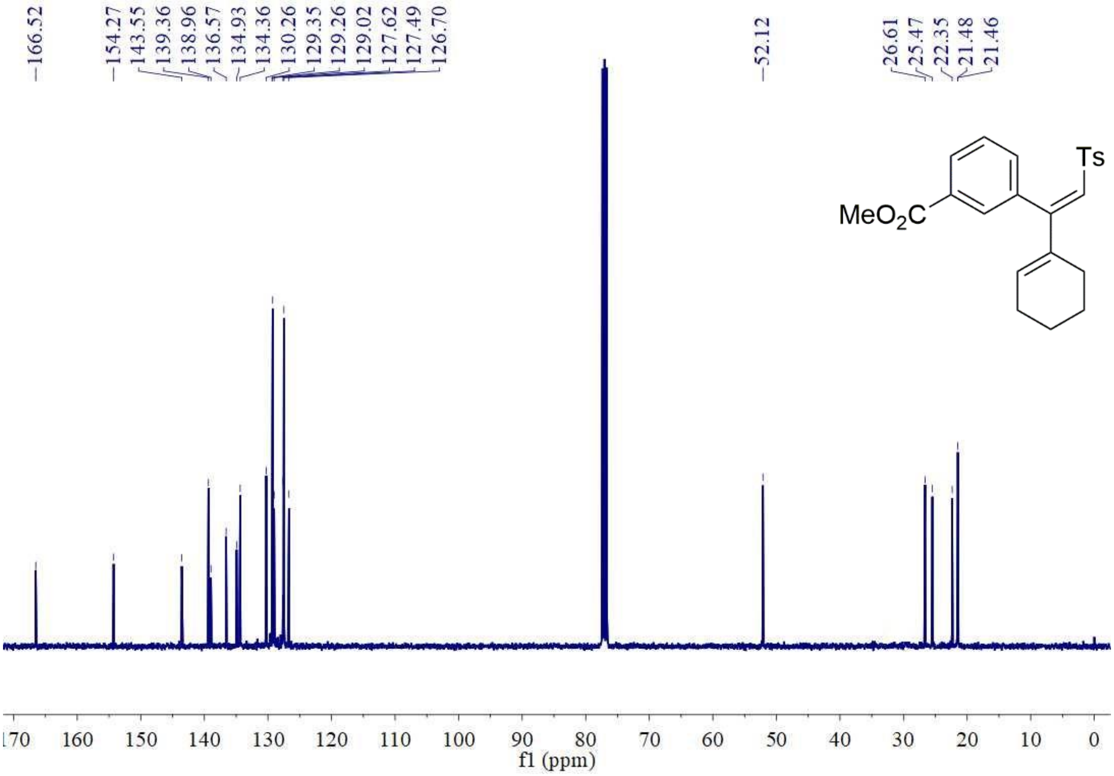

Supplementary Figure 145: ^{13}C NMR of 31b (100 MHz, CDCl3).

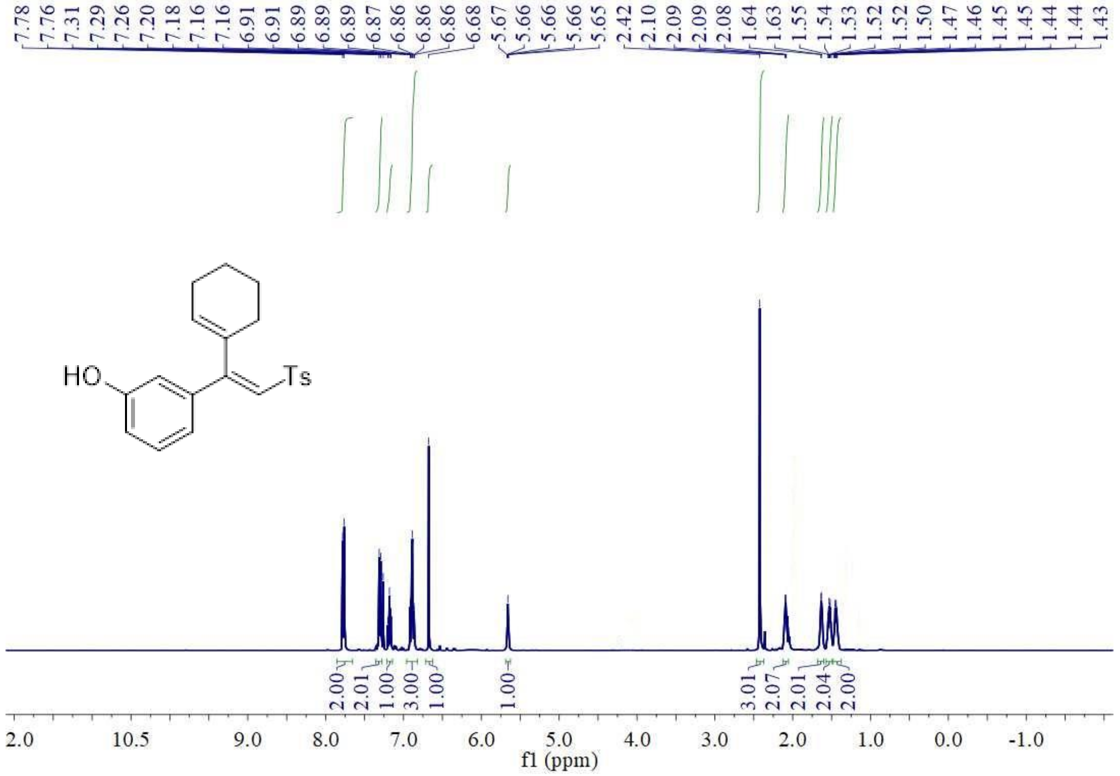

Supplementary Figure 146: ^{1}H NMR of 32a (400 MHz, $CDCl_3$).

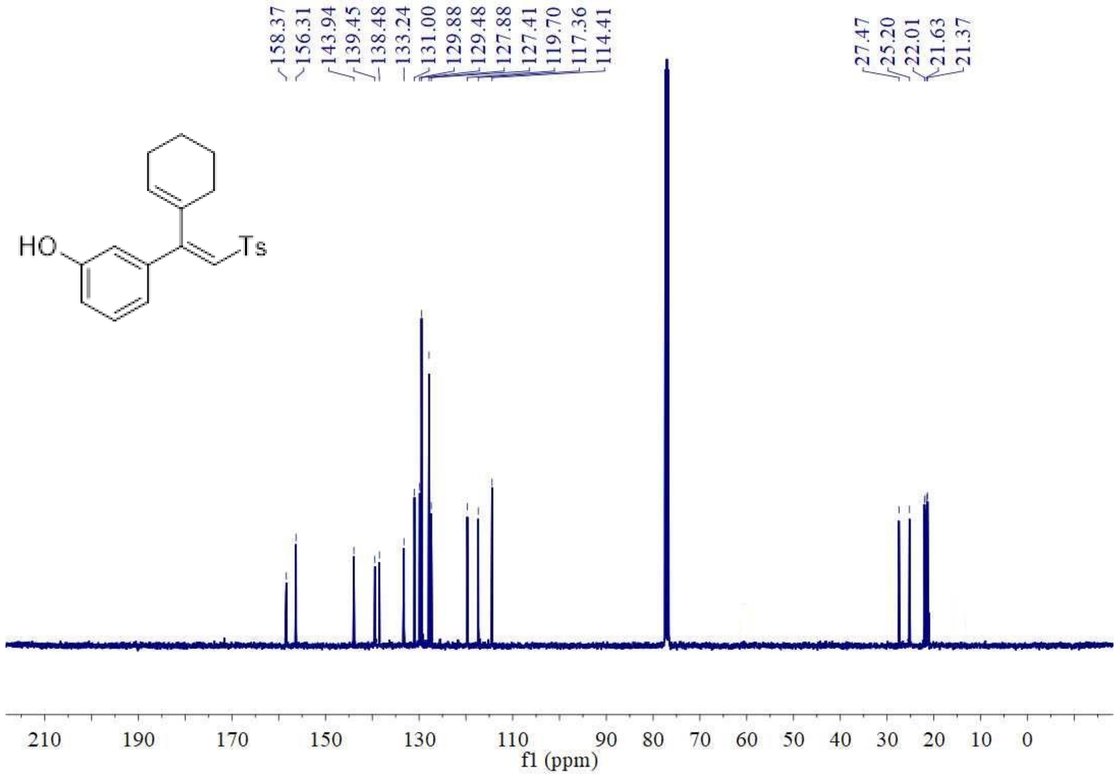

Supplementary Figure 147: ^{13}C NMR of 32a (100 MHz, $CDCl_3$).

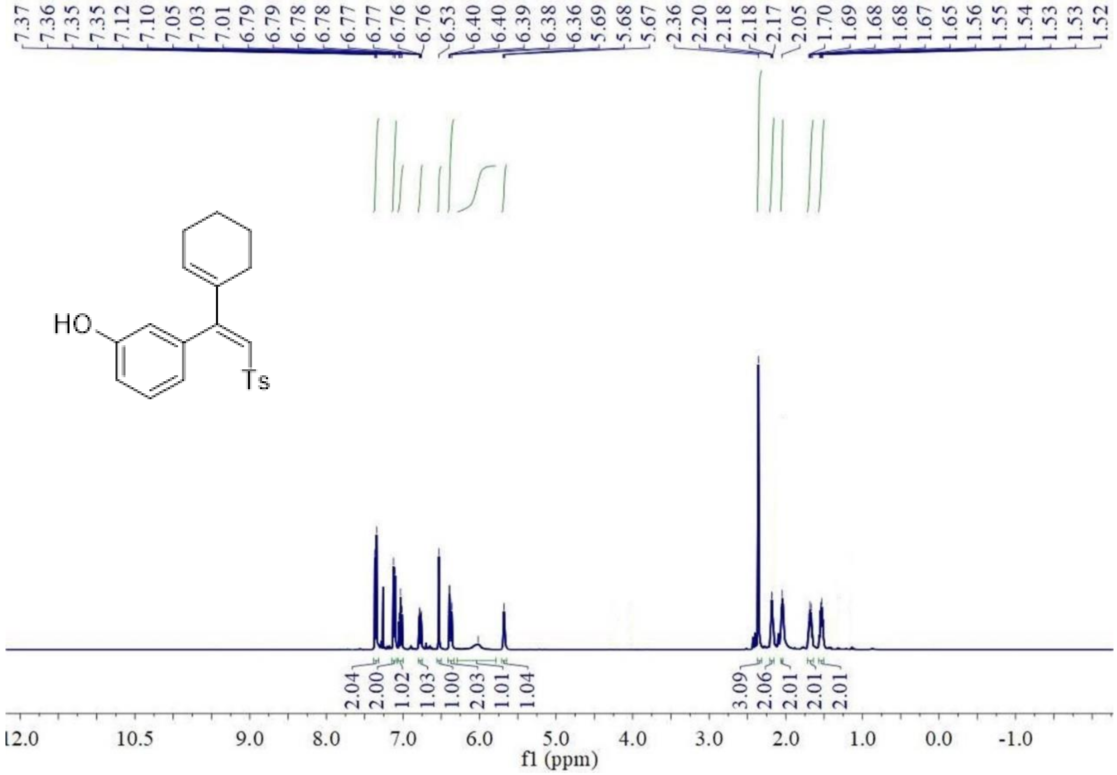

Supplementary Figure 148: ^{1}H NMR of 32b (400 MHz, $CDCl_3$).

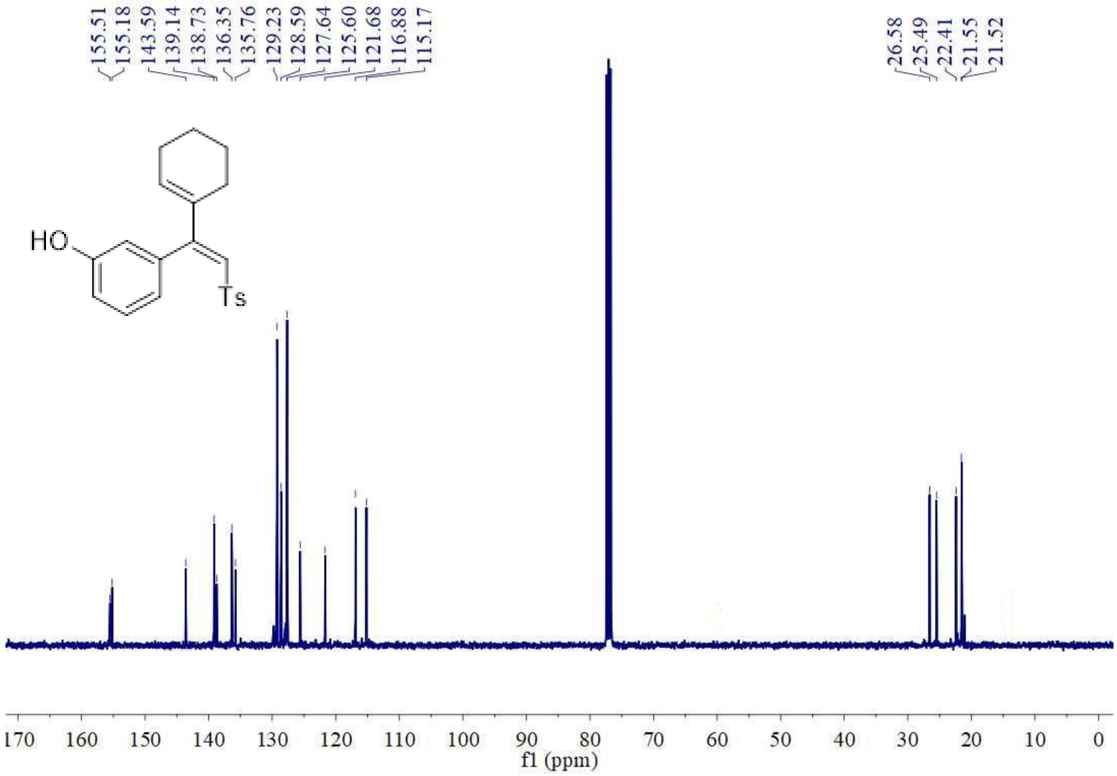

Supplementary Figure 149: ^{13}C NMR of 32b (100 MHz, $CDCl_3$).

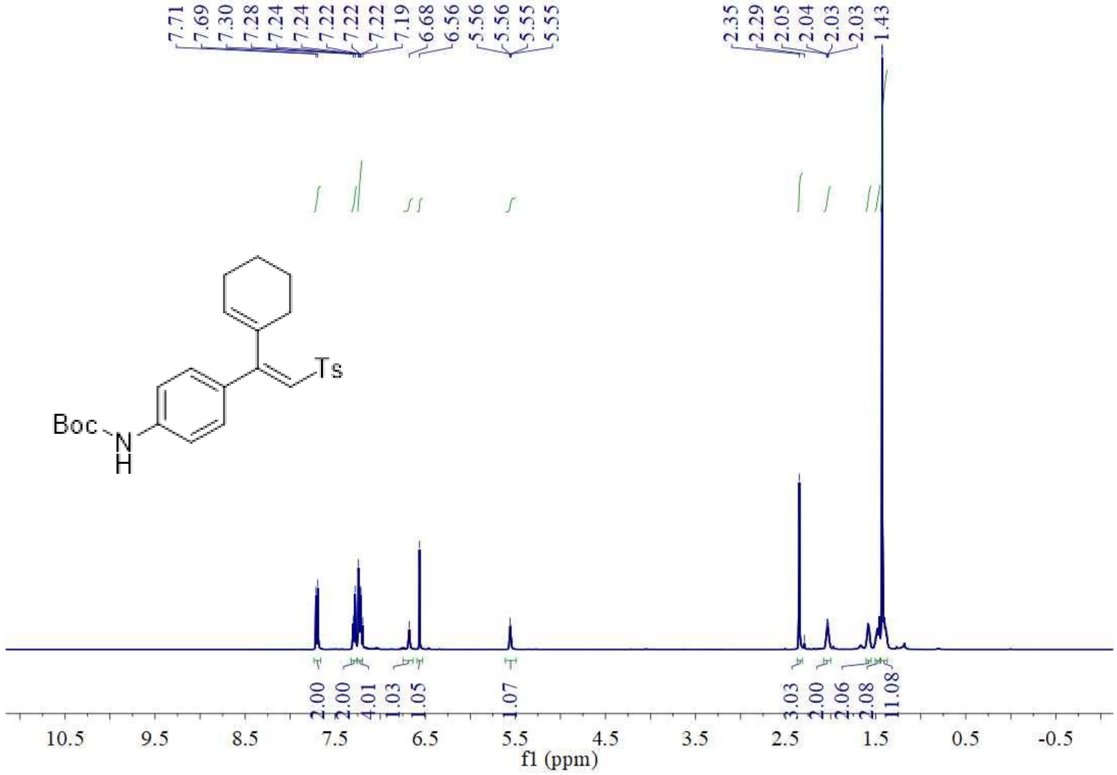

Supplementary Figure 150: ^{1}H NMR of 33a (400 MHz, $CDCl_3$).

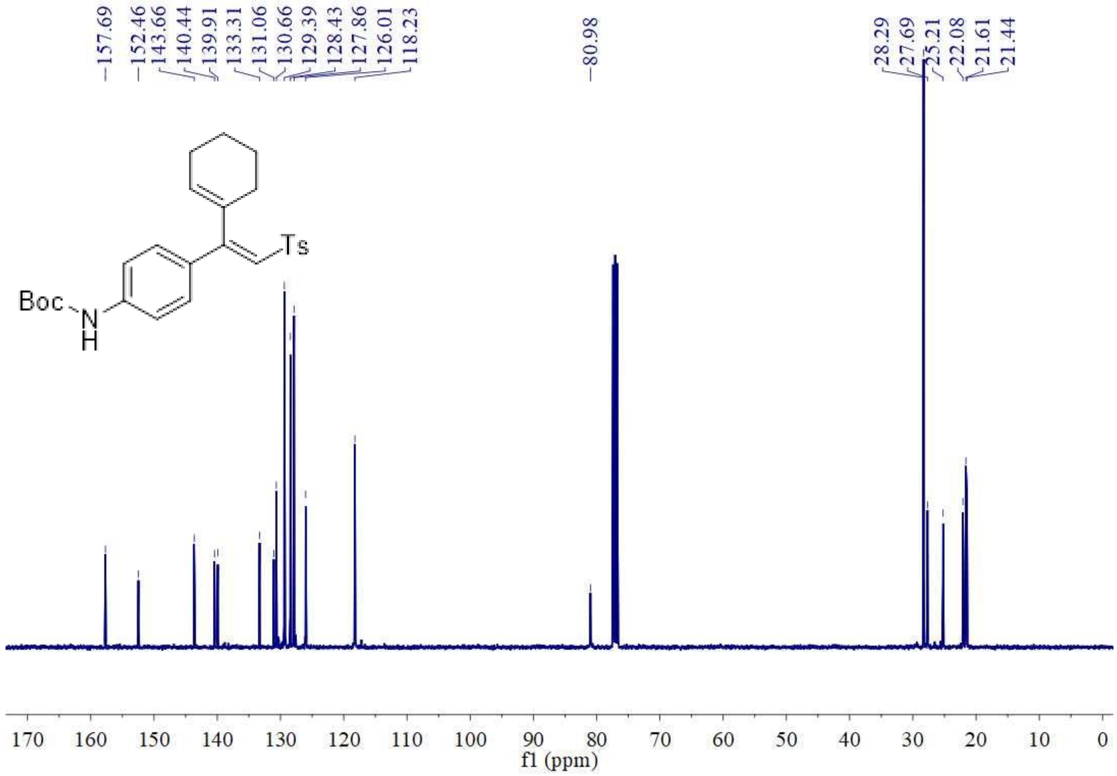

Supplementary Figure 151: ^{13}C NMR of 33a (100 MHz, $CDCl_3$).

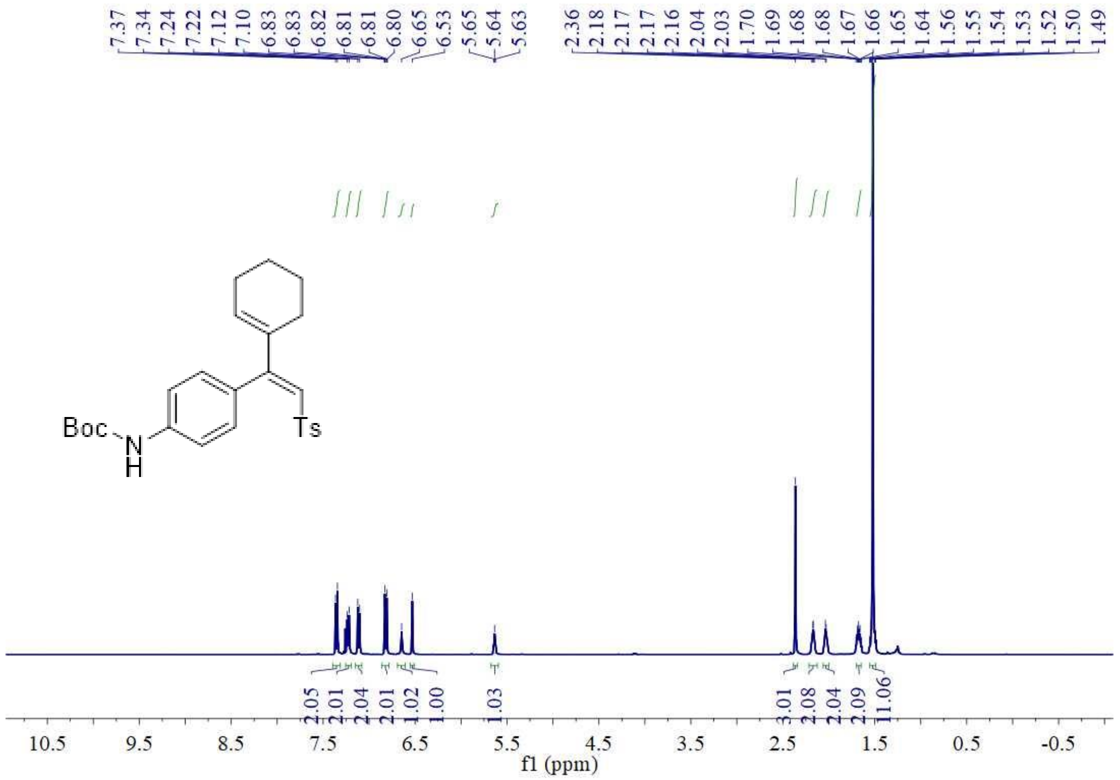

Supplementary Figure 152: ^{1}H NMR of 33b (400 MHz, $CDCl_3$).

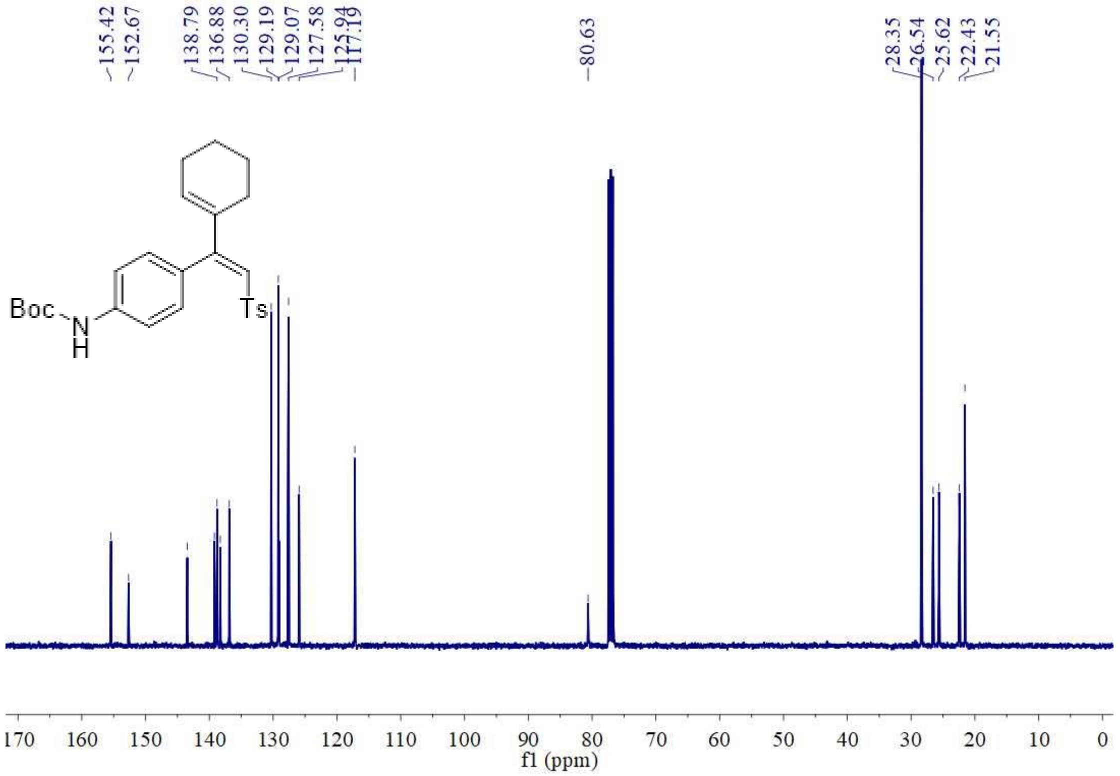

Supplementary Figure 153: ^{13}C NMR of 33b (100 MHz, $CDCl_3$).

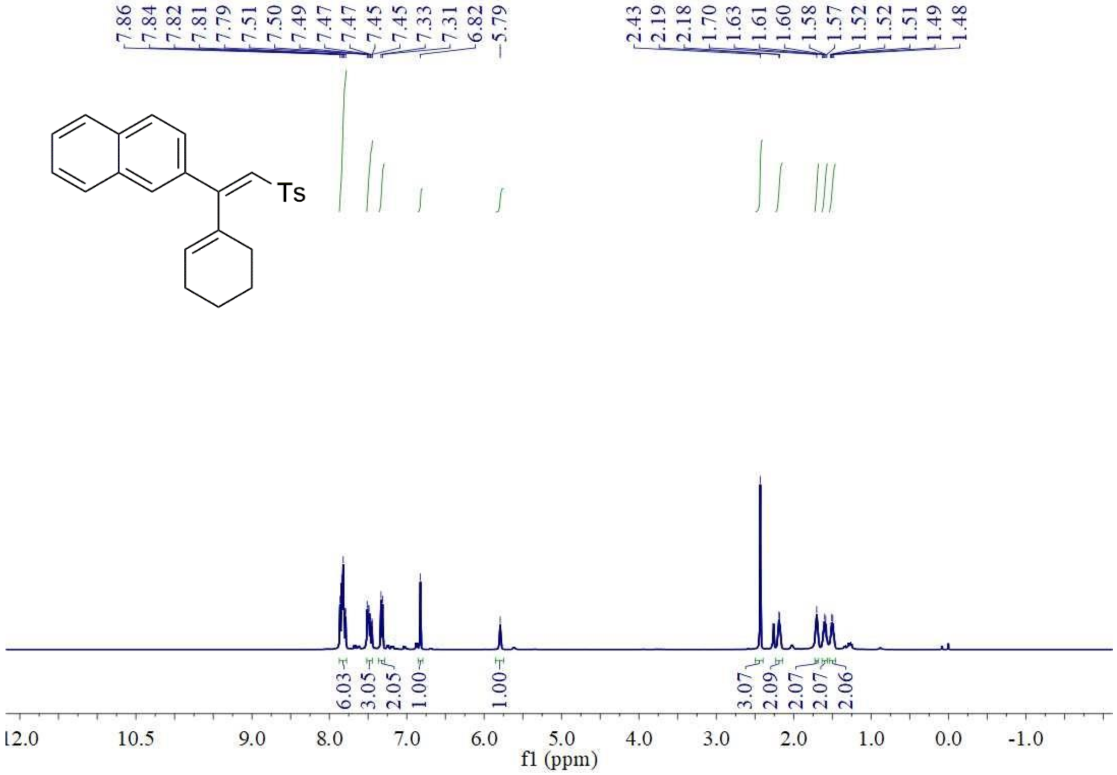

Supplementary Figure 154: ^{1}H NMR of 34a (400 MHz, $CDCl_3$).

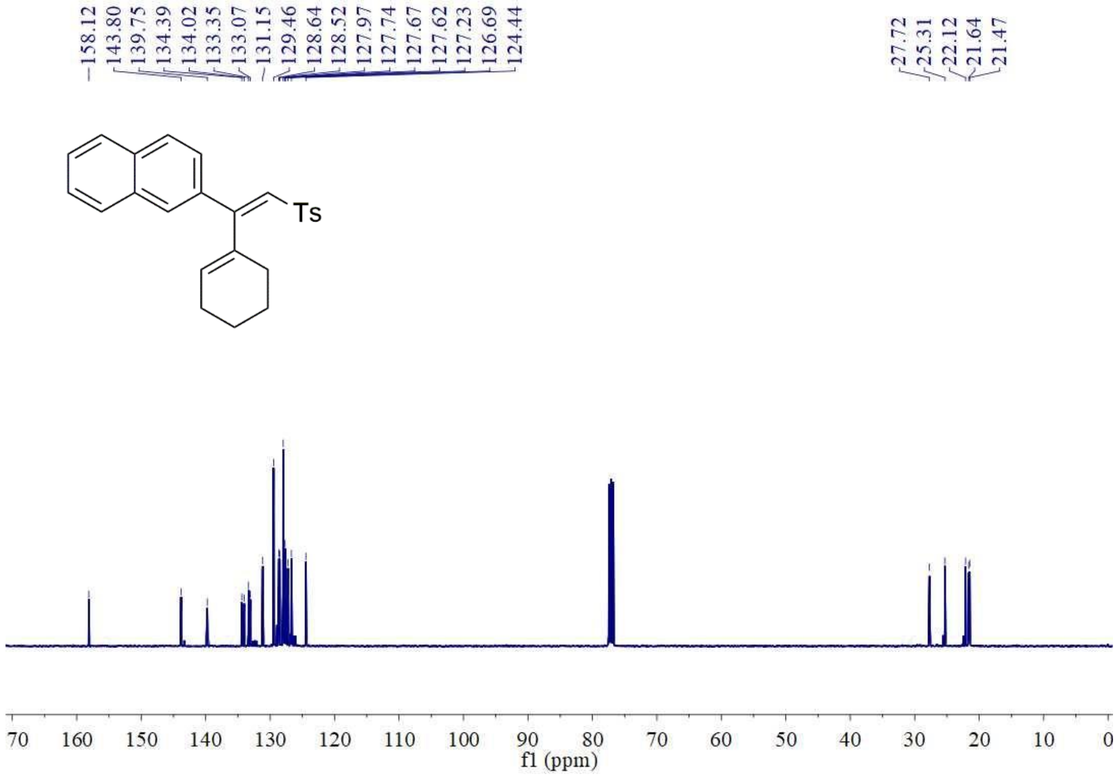

Supplementary Figure 155: ^{13}C NMR of 34a (100 MHz, $CDCl_3$).

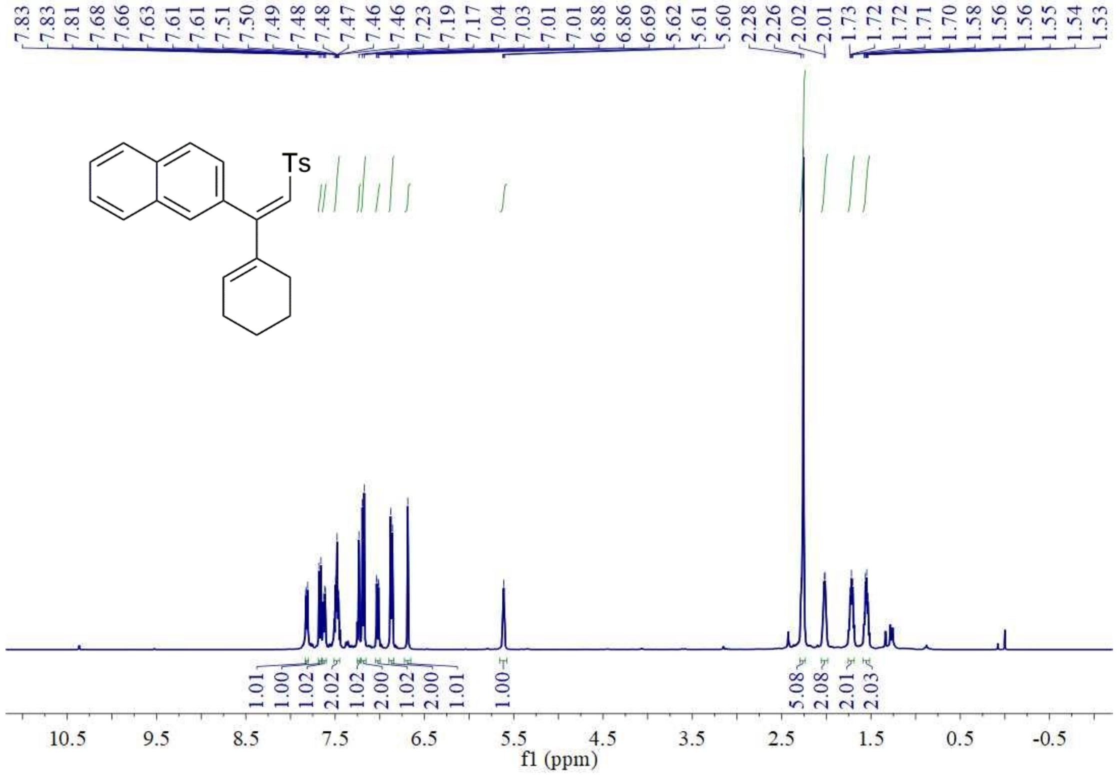

Supplementary Figure 156: ^{1}H NMR of 34b (400 MHz, $CDCl_3$).

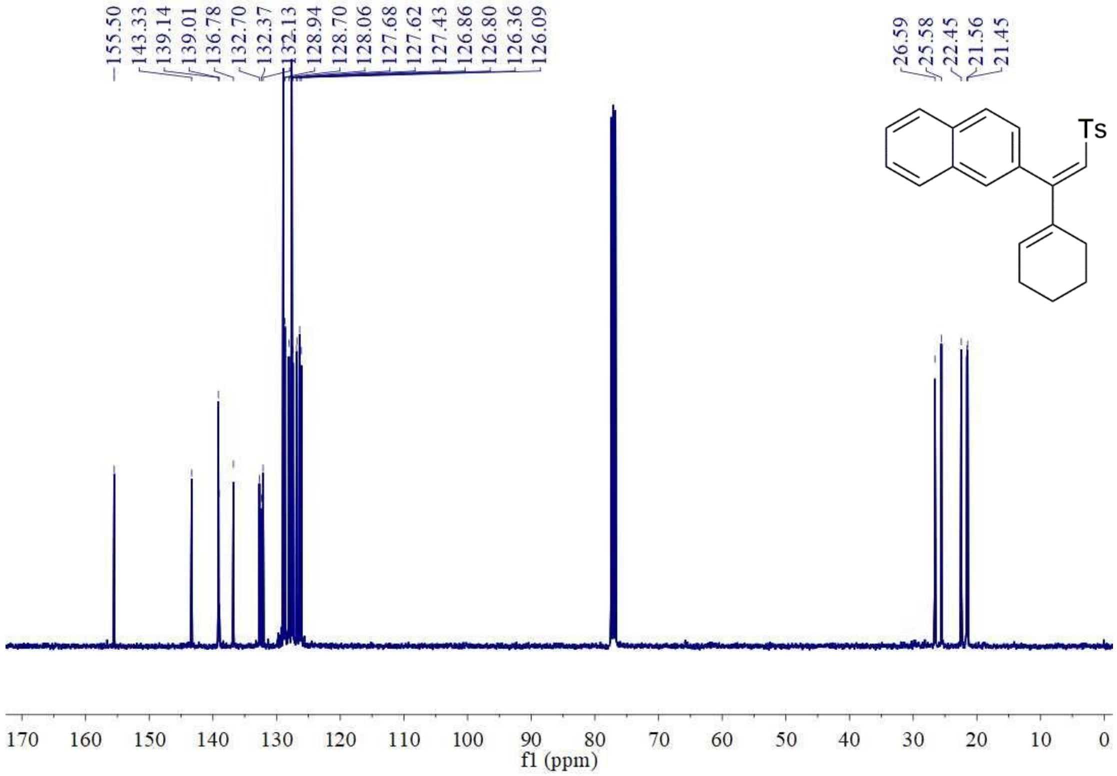

Supplementary Figure 157: ^{13}C NMR of 34b (100 MHz, $CDCl_3$).

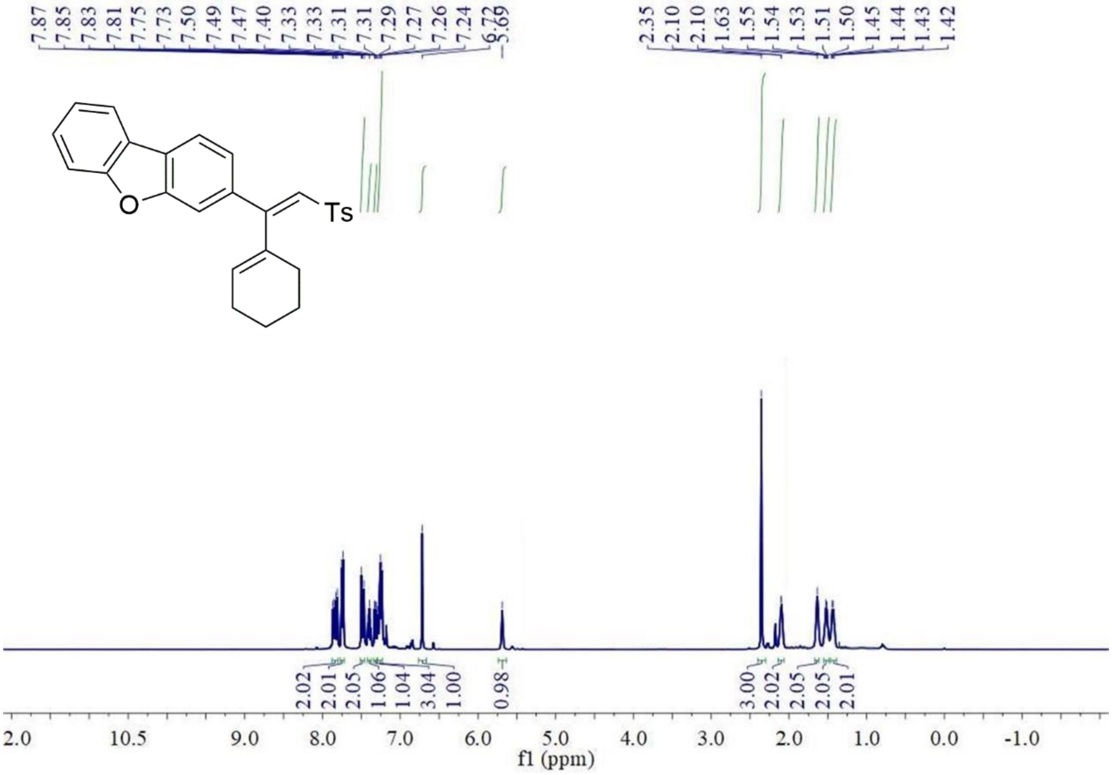

Supplementary Figure 158: ^{1}H NMR of 35a (400 MHz, $CDCl_3$).

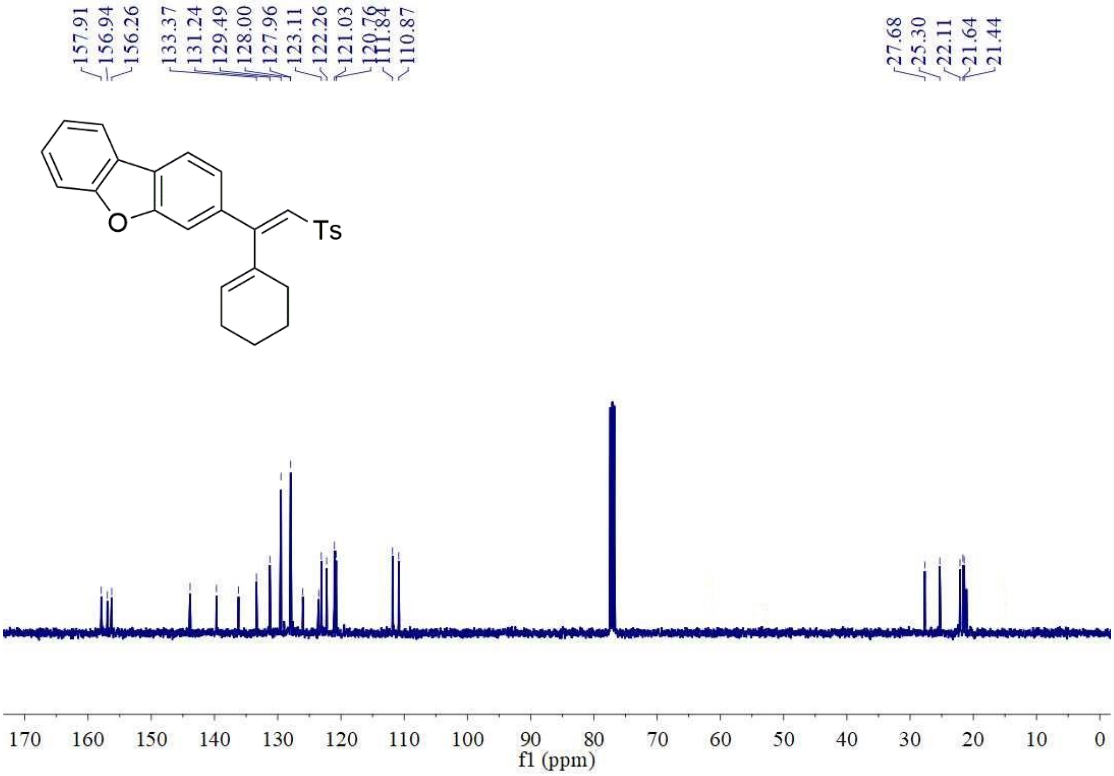

Supplementary Figure 159: ^{13}C NMR of 35a (100 MHz, $CDCl_3$).

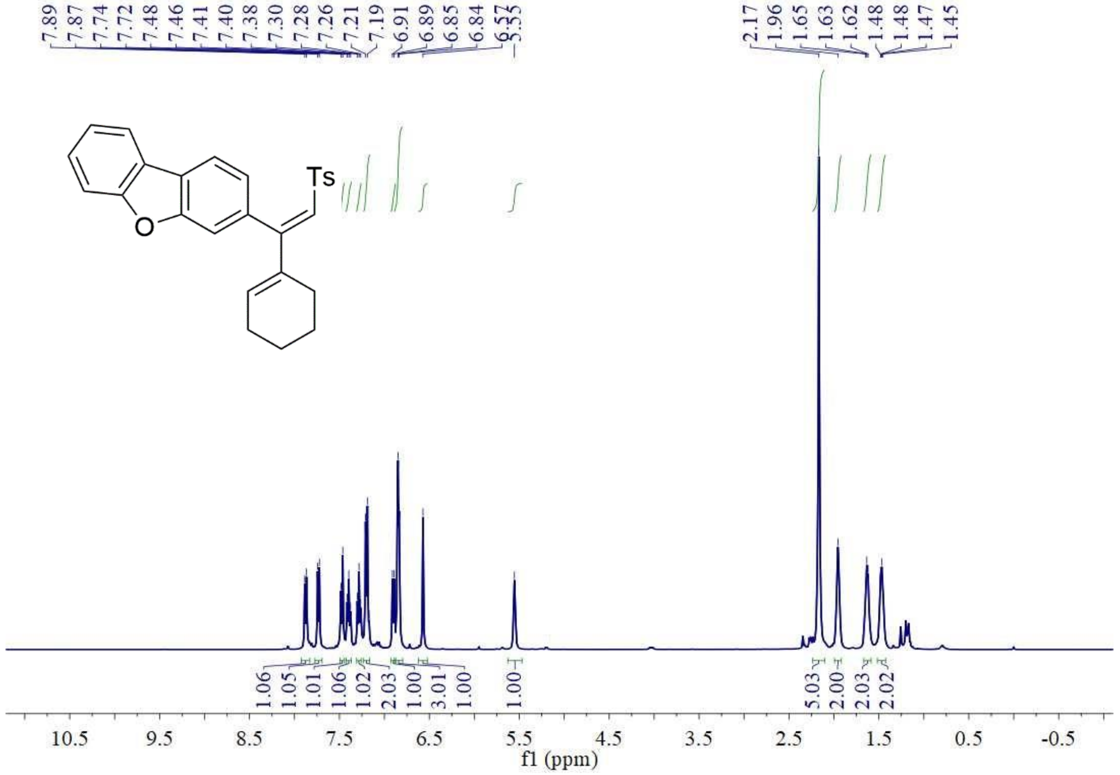

Supplementary Figure 160: ^{1}H NMR of 35b (400 MHz, $CDCl_3$).

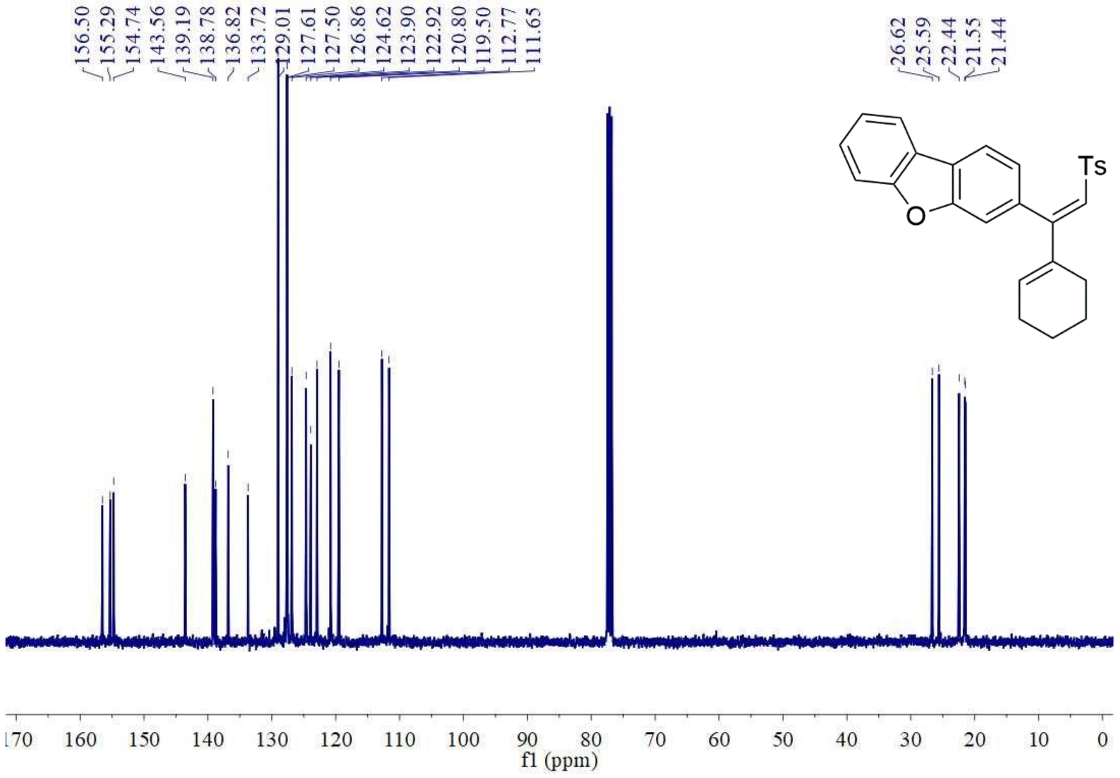

Supplementary Figure 161: ^{13}C NMR of 35b (100 MHz, $CDCl_3$).

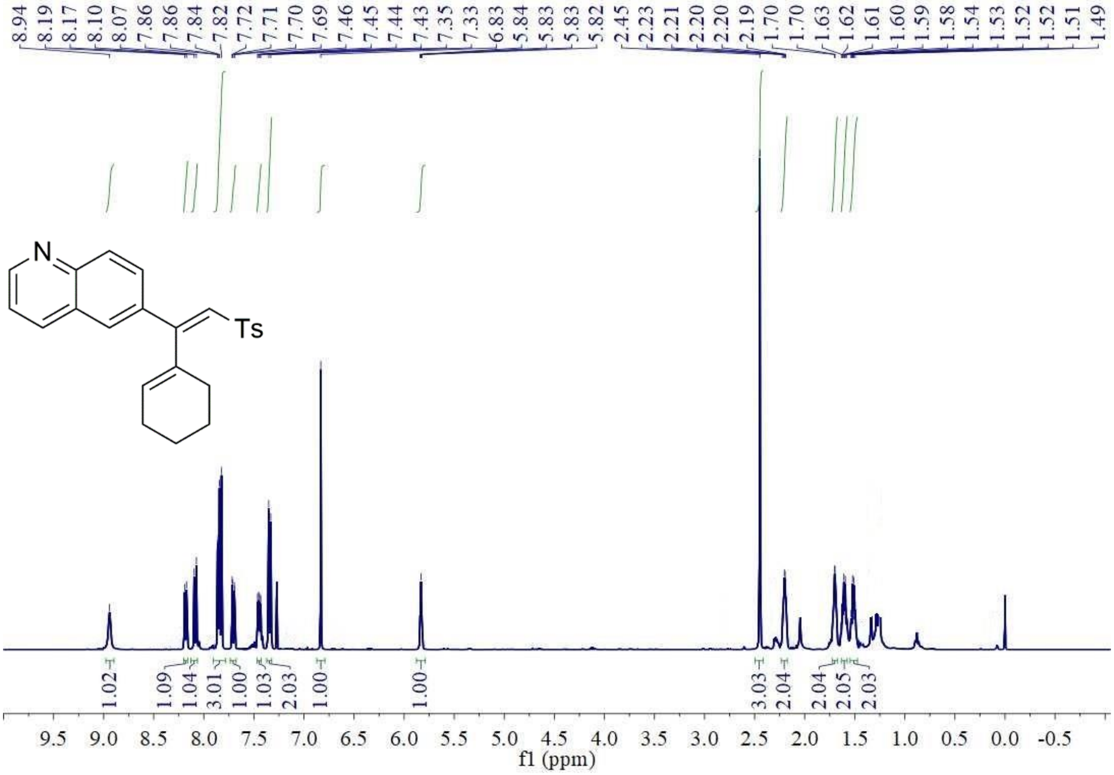

Supplementary Figure 162: ^{1}H NMR of 36a (400 MHz, $CDCl_3$).

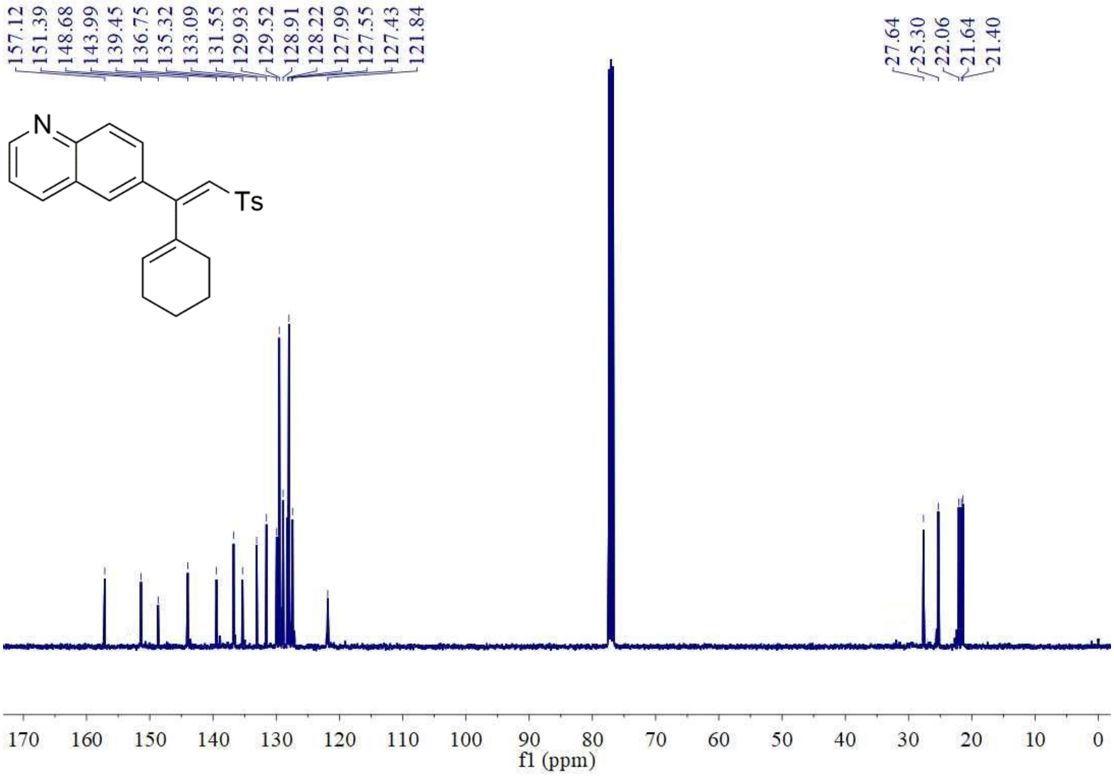

Supplementary Figure 163: ^{13}C NMR of 36a (100 MHz, $CDCl_3$).

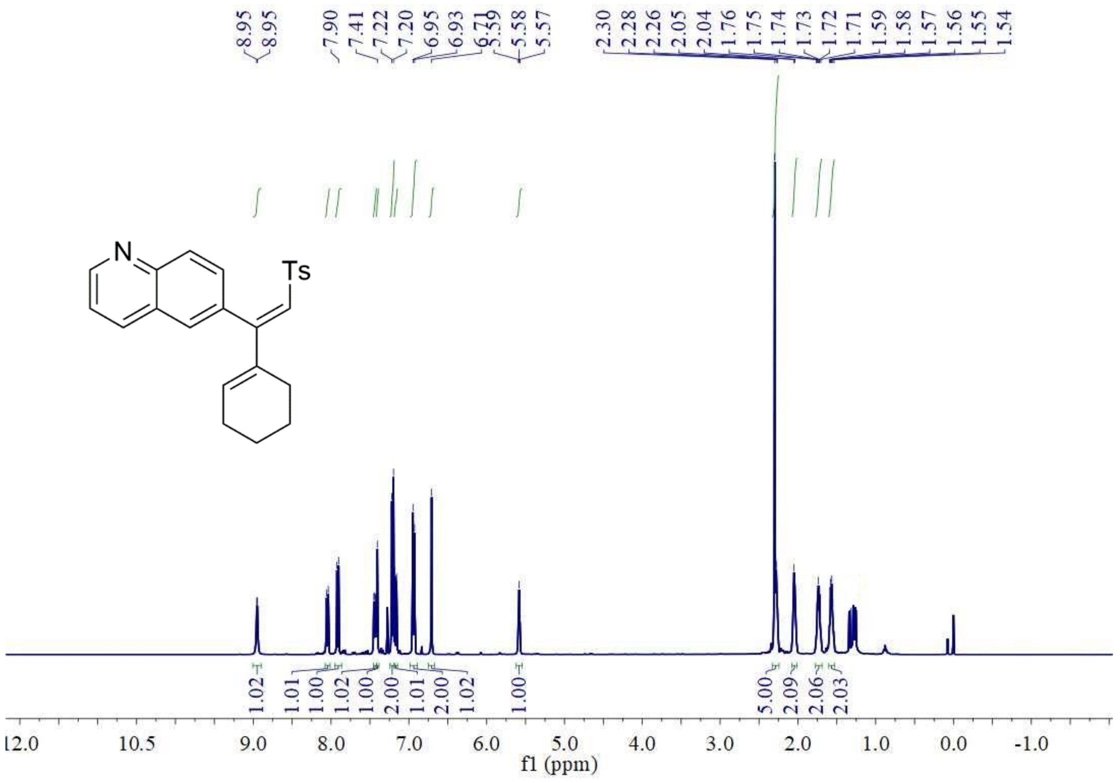

Supplementary Figure 164: ^{1}H NMR of 36b (400 MHz, $CDCl_3$).

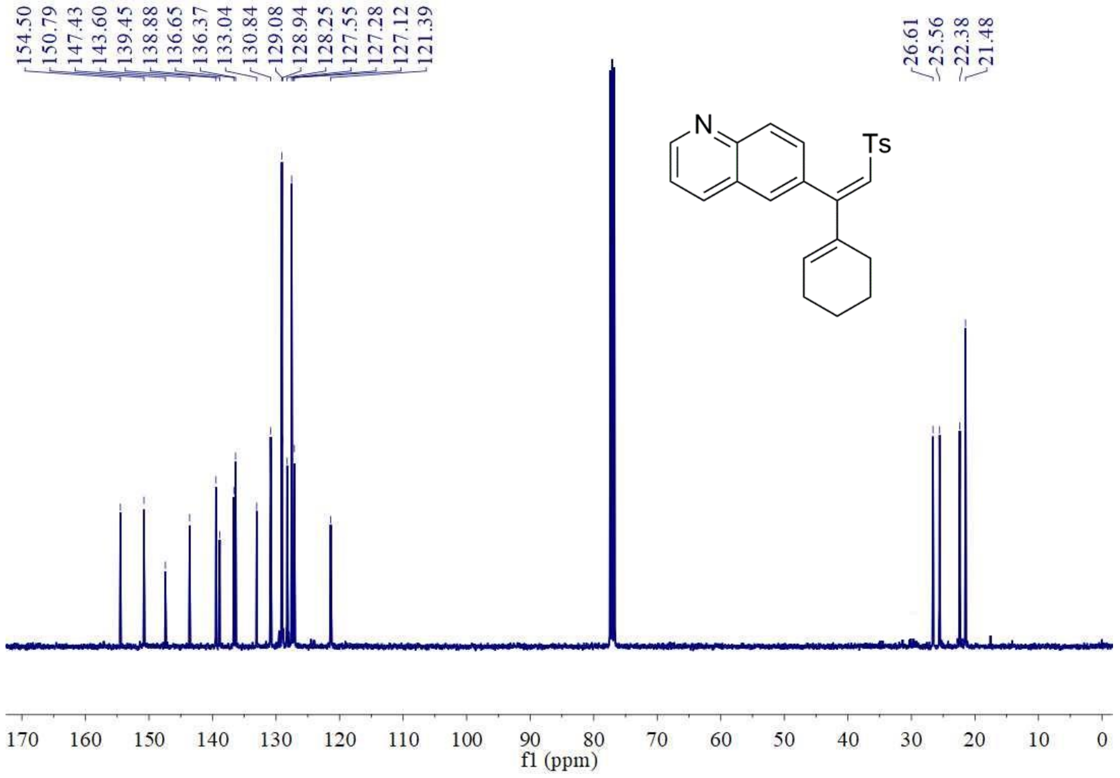

Supplementary Figure 165: ^{13}C NMR of 36b (100 MHz, $CDCl_3$).

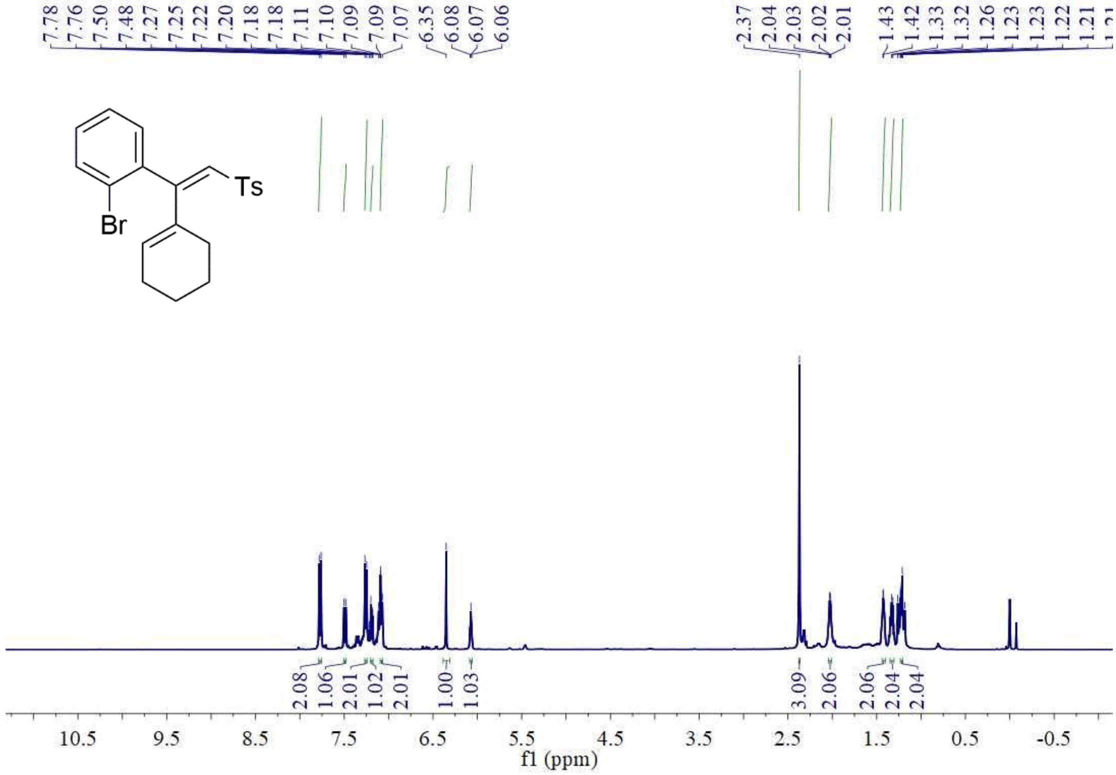

Supplementary Figure 166: ^{1}H NMR of 37a (400 MHz, $CDCl_3$).

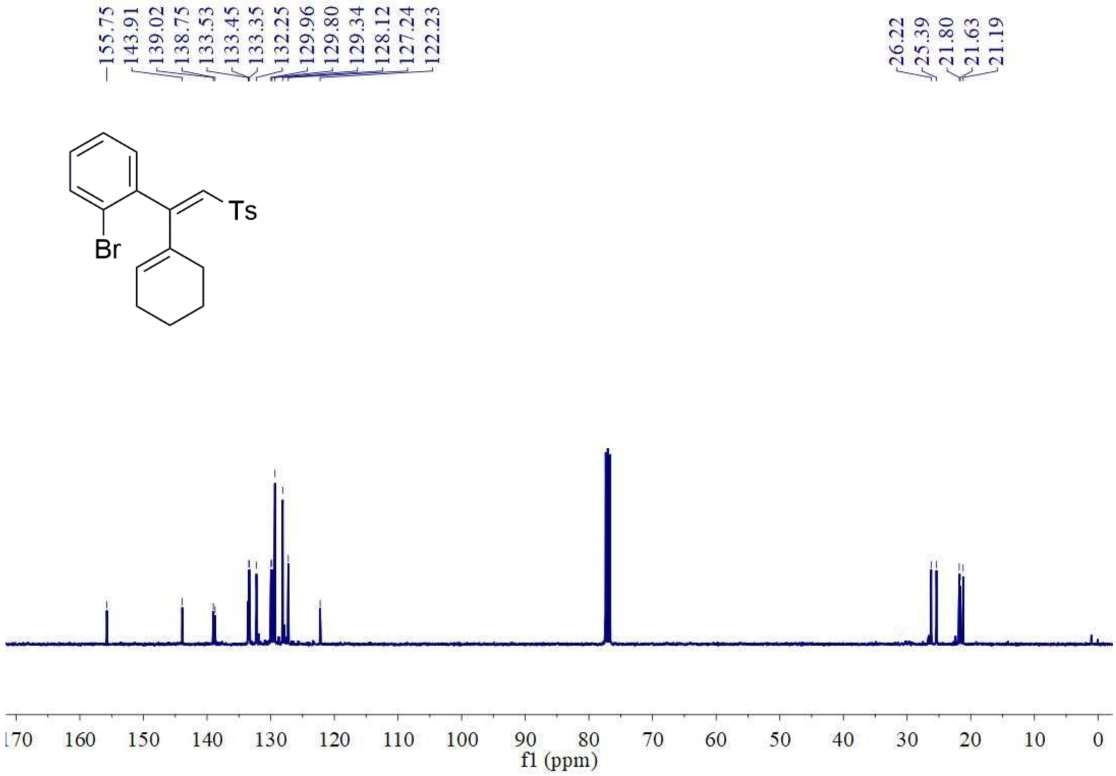

Supplementary Figure 167: ^{13}C NMR of 37a (100 MHz, $CDCl_3$).

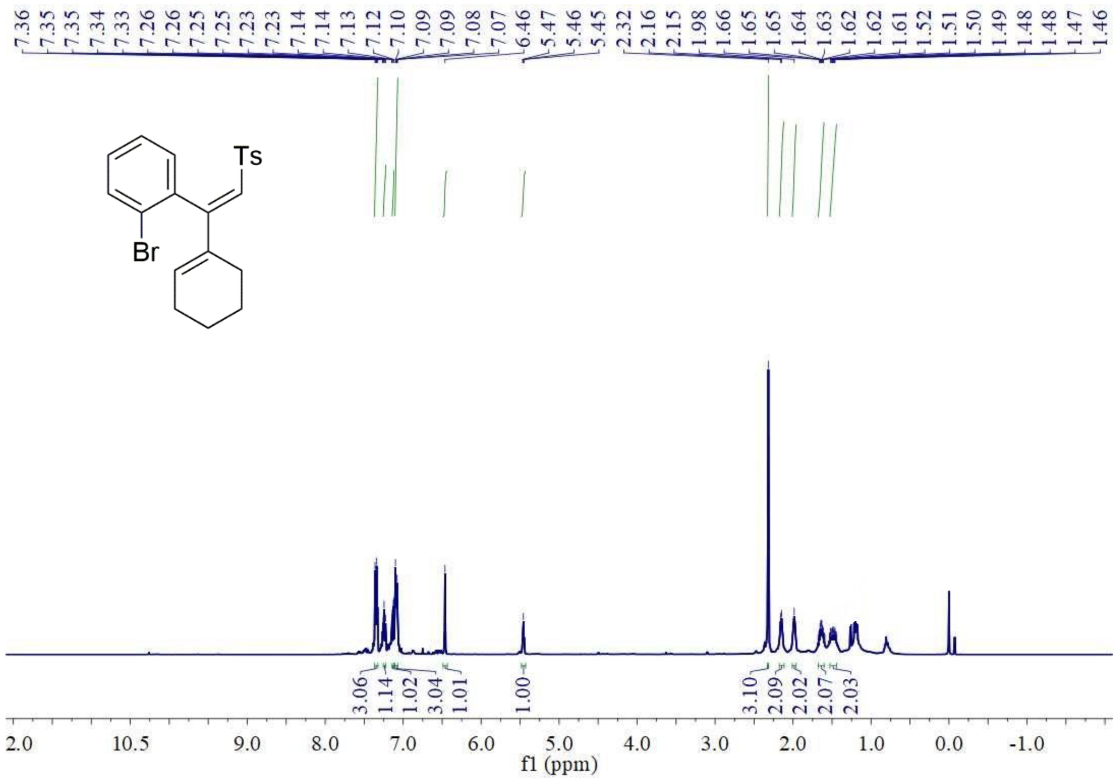

Supplementary Figure 168: ^{1}H NMR of 37b (400 MHz, $CDCl_3$).

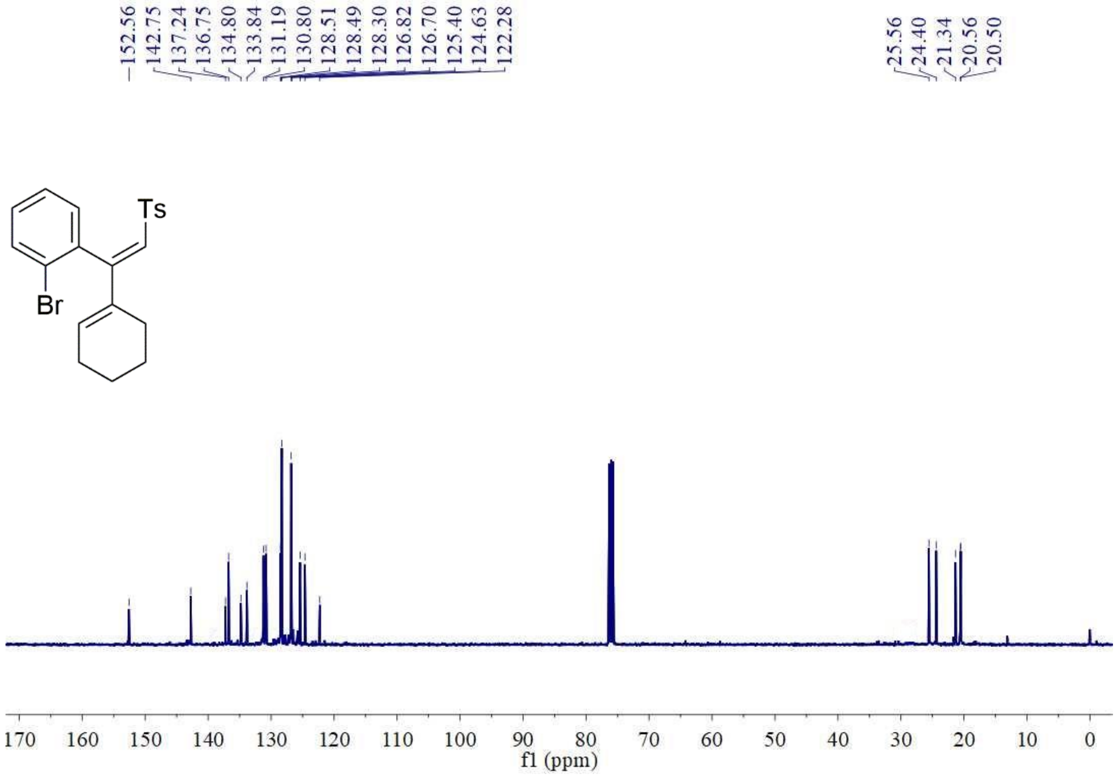

Supplementary Figure 169: ^{13}C NMR of 37b (100 MHz, $CDCl_3$).

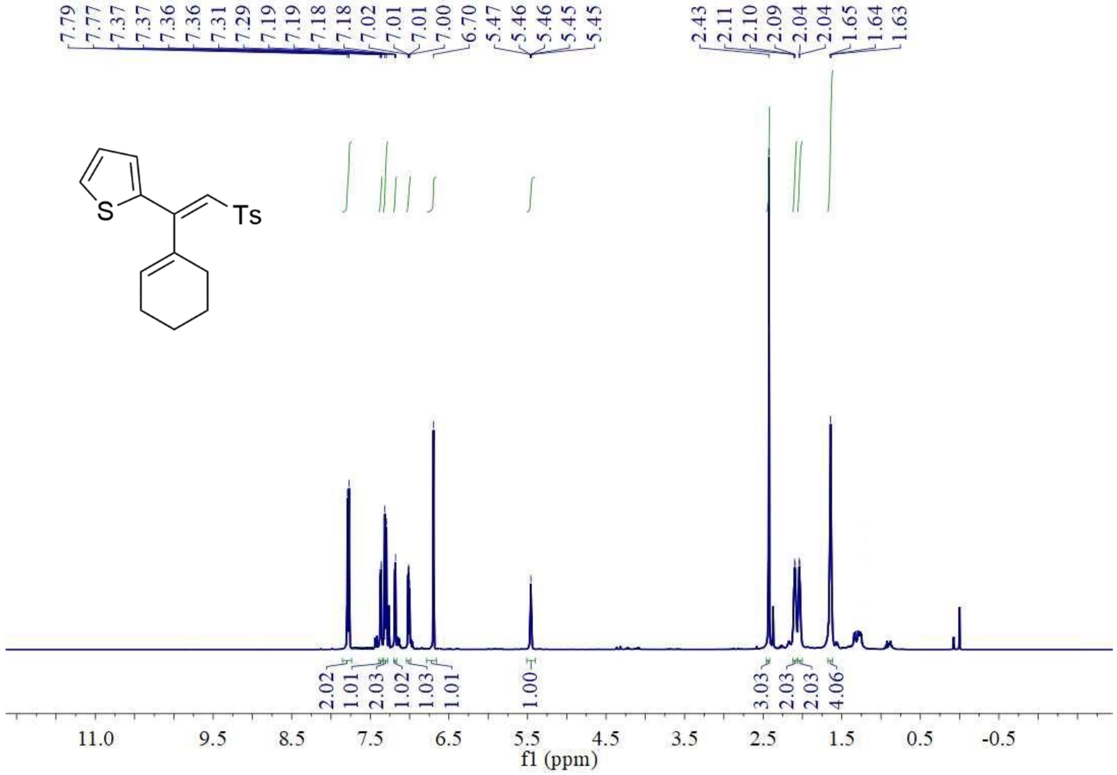

Supplementary Figure 170: ^{1}H NMR of 38a (400 MHz, $CDCl_3$).

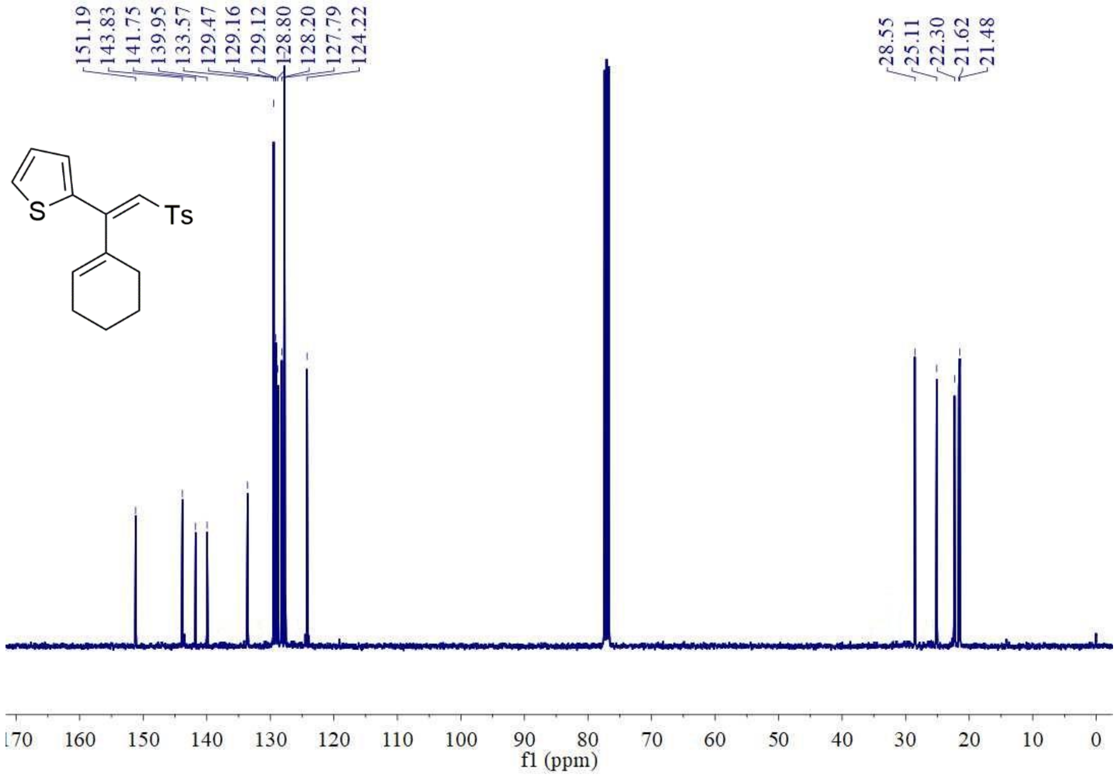

Supplementary Figure 171: ^{13}C NMR of 38a (100 MHz, $CDCl_3$).

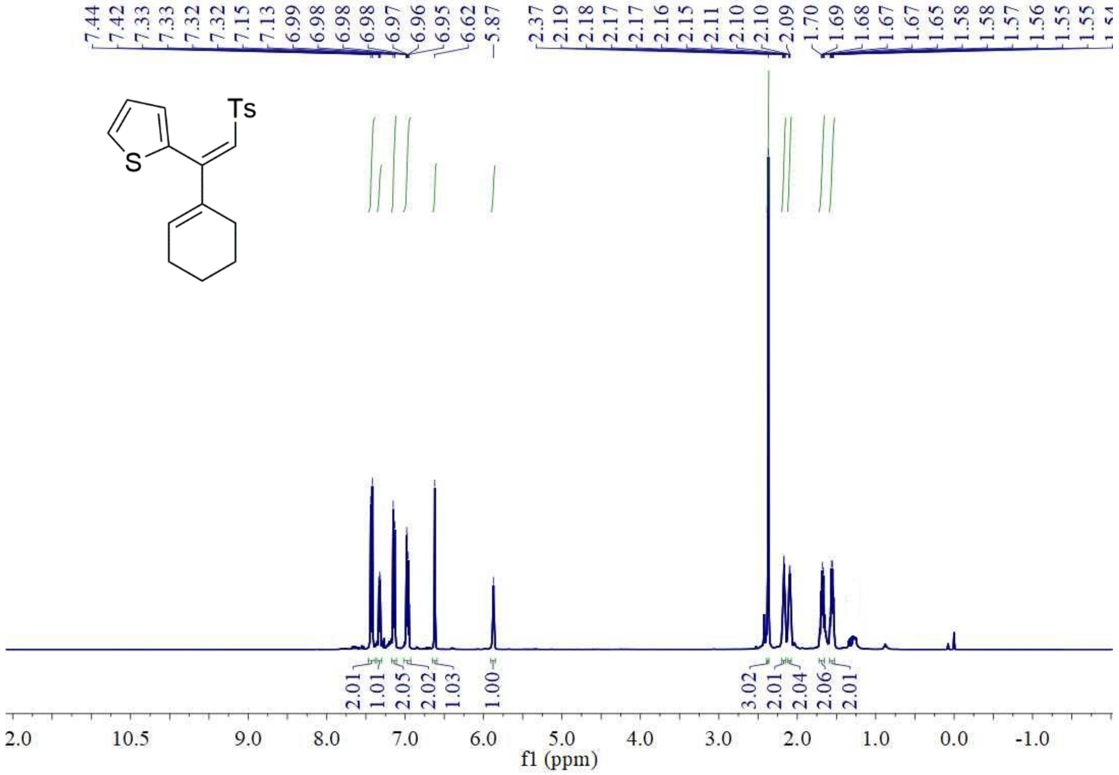

Supplementary Figure 172: ^{1}H NMR of 38b (400 MHz, $CDCl_3$).

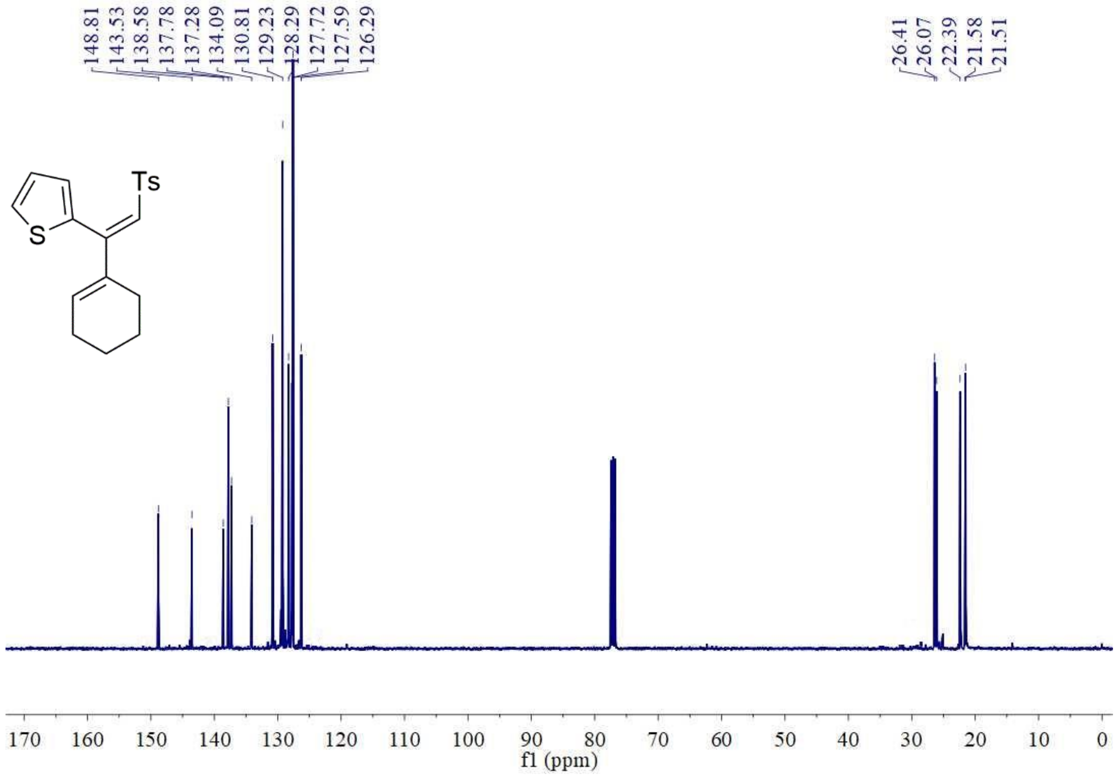

Supplementary Figure 173: ^{13}C NMR of 38b (100 MHz, $CDCl_3$).

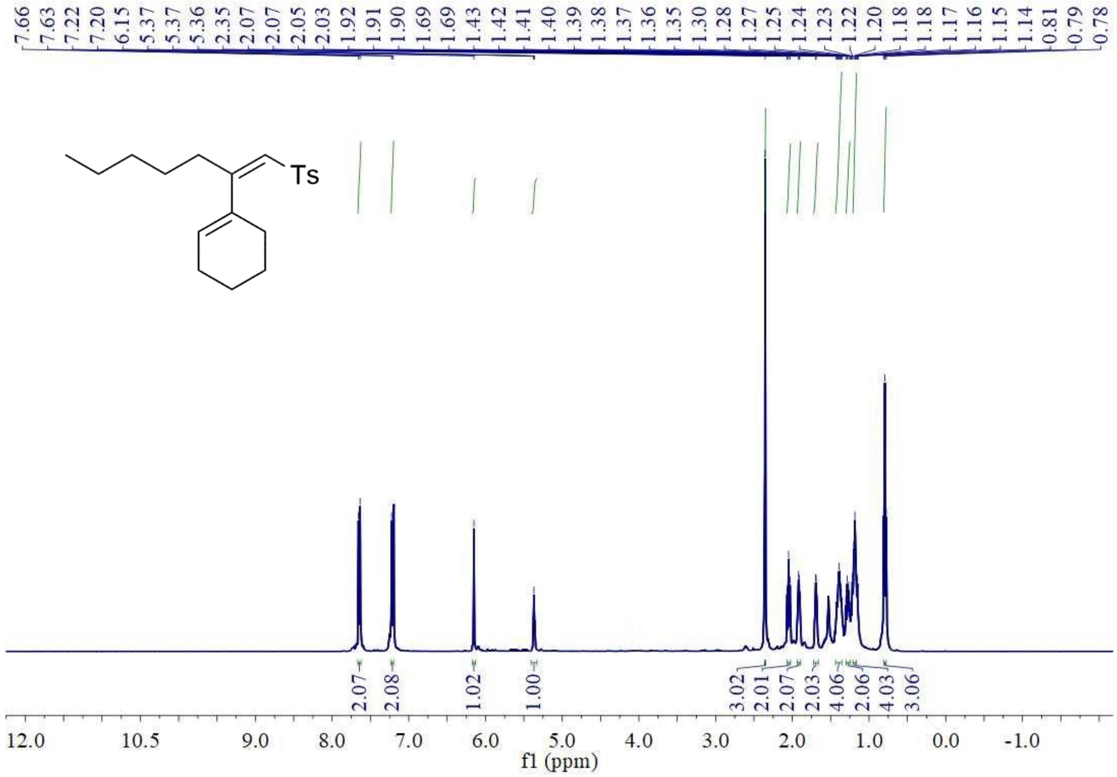

Supplementary Figure 174: ^{1}H NMR of 39a (400 MHz, $CDCl_3$).

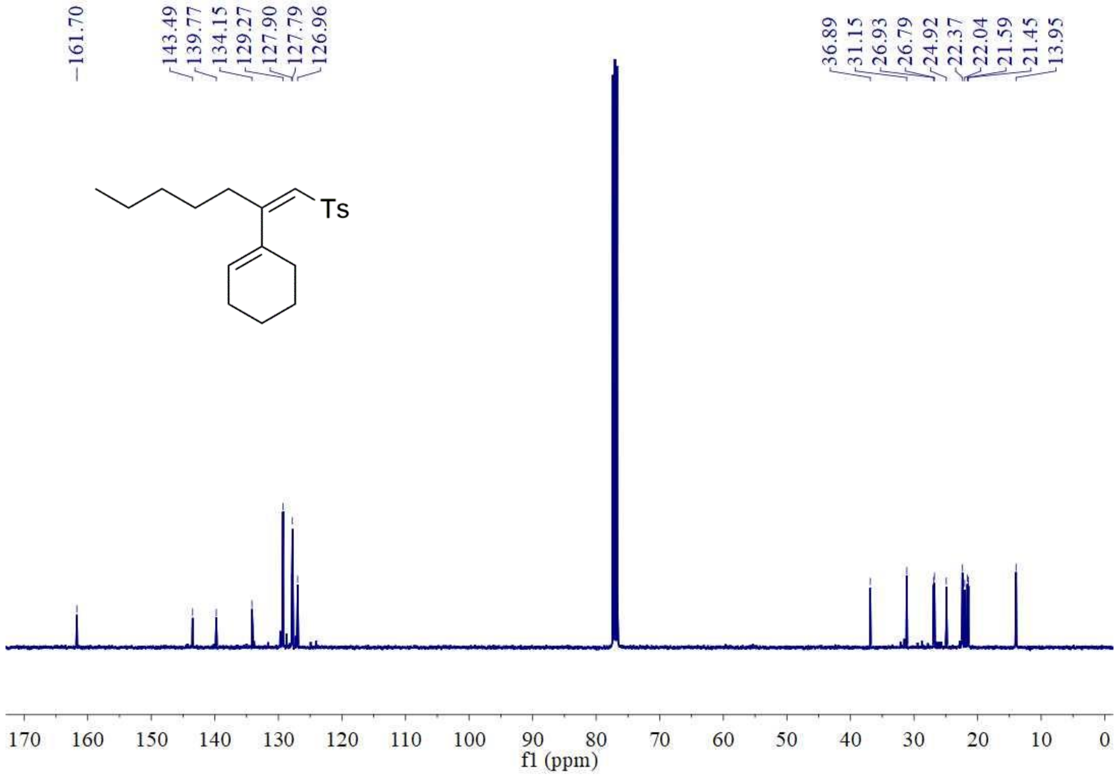

Supplementary Figure 175: ^{13}C NMR of 39a (100 MHz, $CDCl_3$).

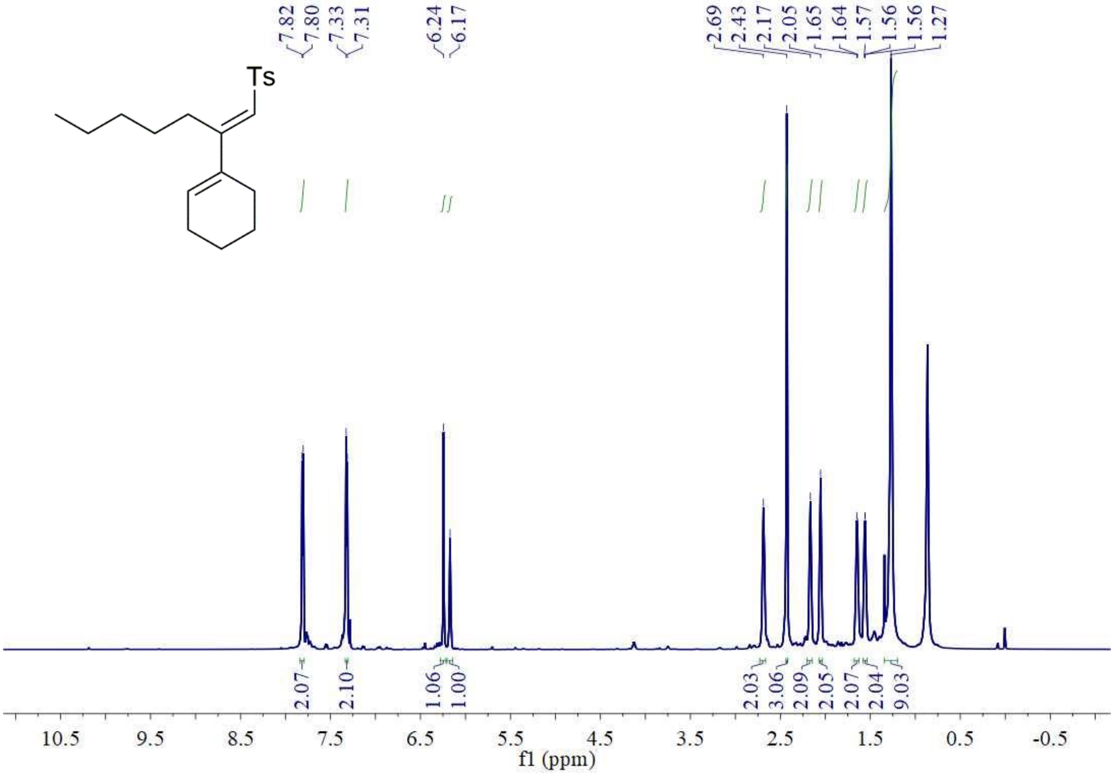

Supplementary Figure 176: ^{1}H NMR of 39b (600 MHz, $CDCl_3$).

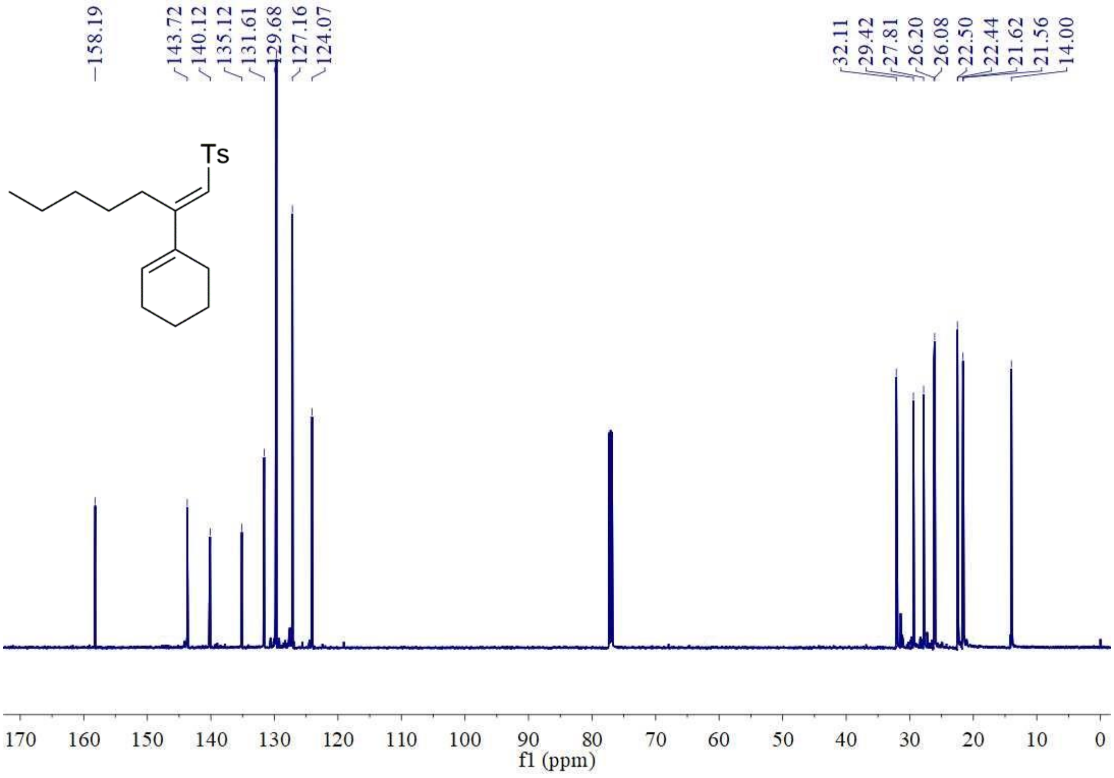

Supplementary Figure 177: ^{13}C NMR of 39b (150 MHz, $CDCl_3$).

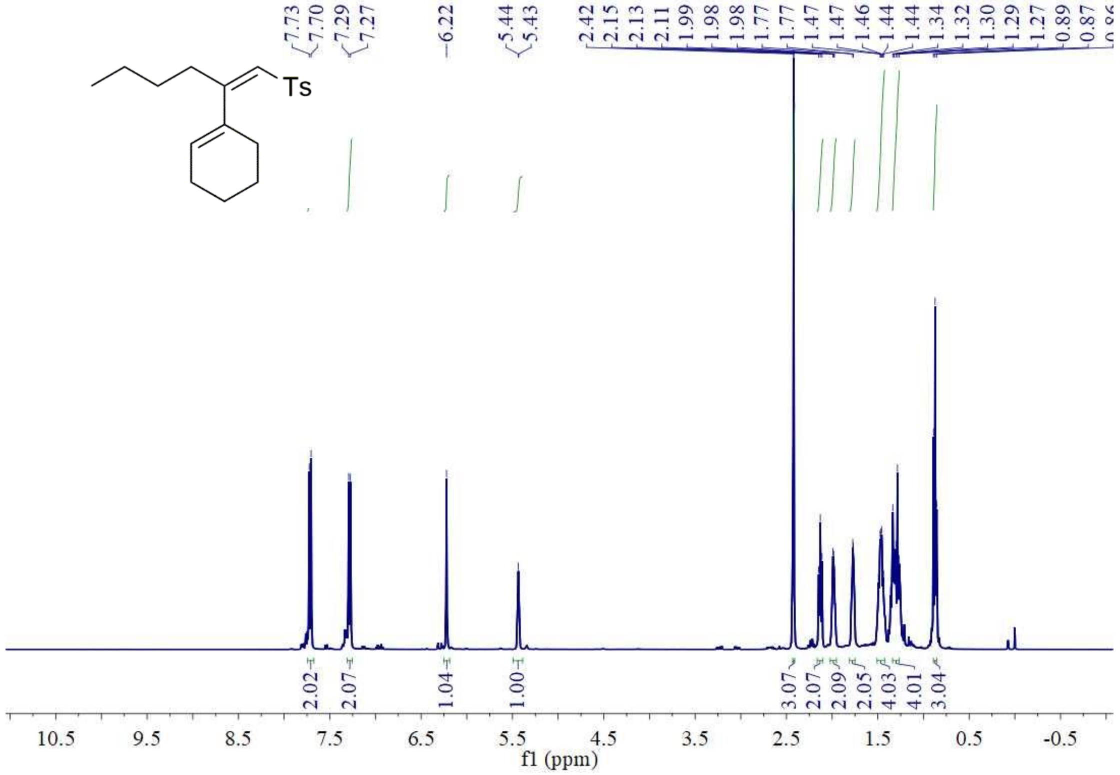

Supplementary Figure 178: ^{1}H NMR of 40a (400 MHz, $CDCl_3$).

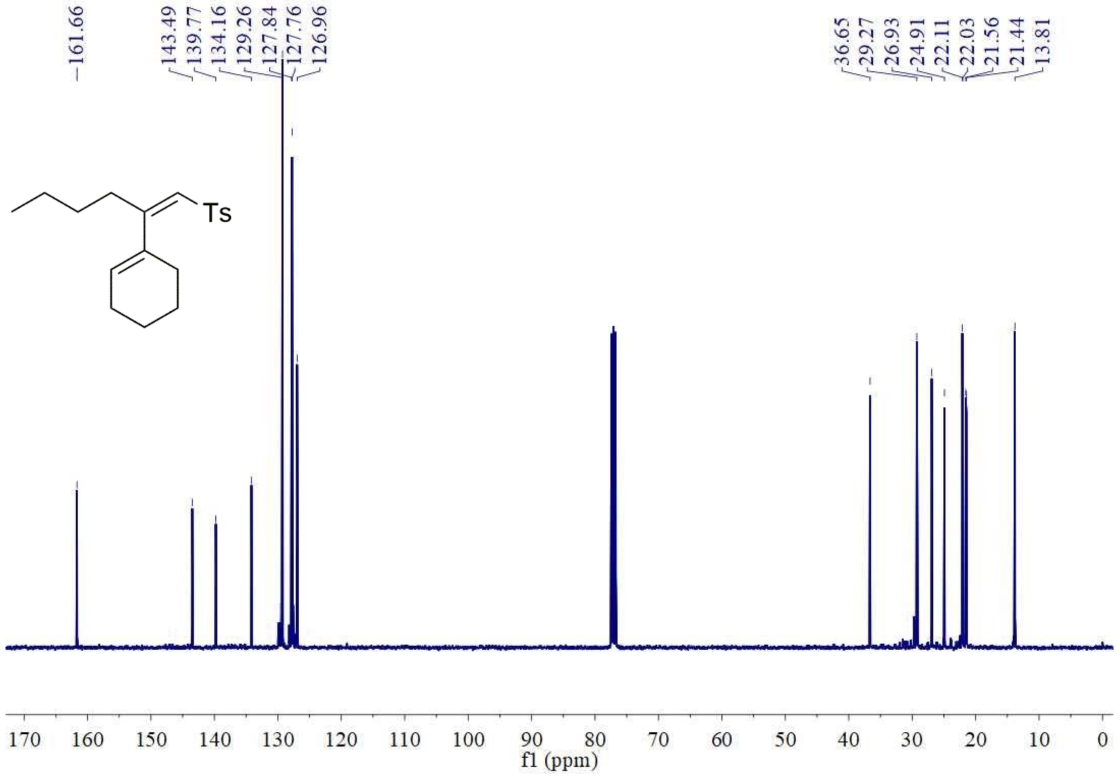

Supplementary Figure 179: ^{13}C NMR of 40a (100 MHz, $CDCl_3$).

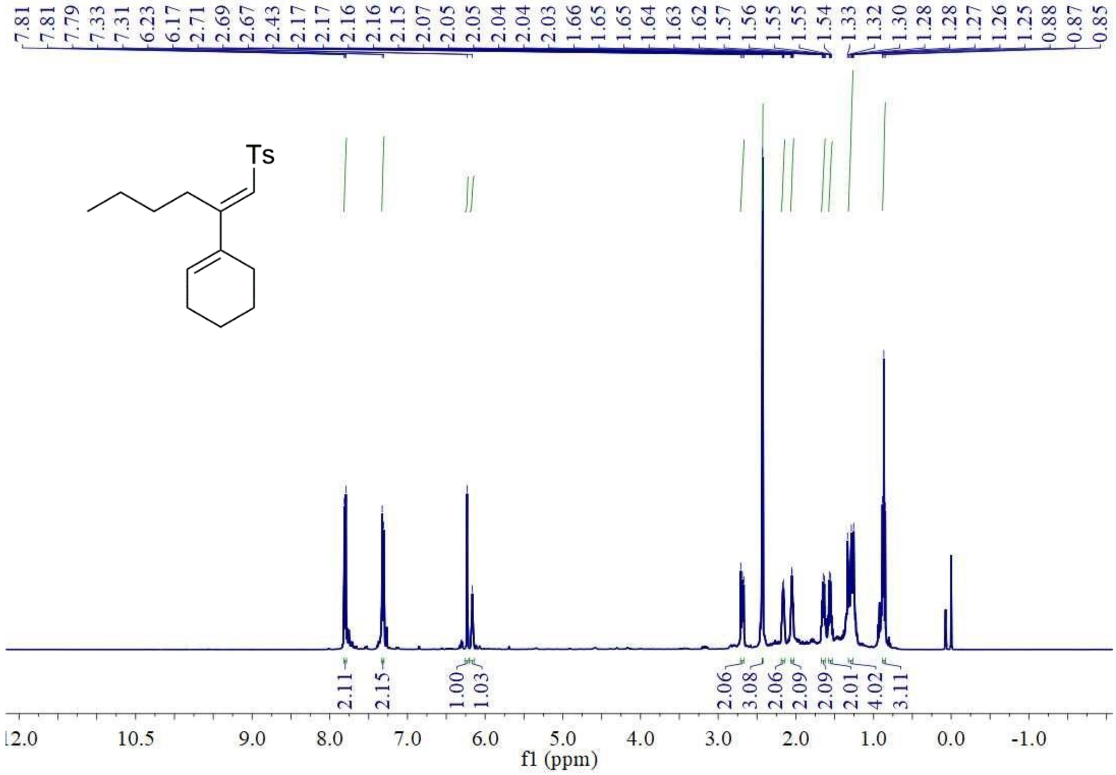

Supplementary Figure 180: ^{1}H NMR of 40b (400 MHz, $CDCl_3$).

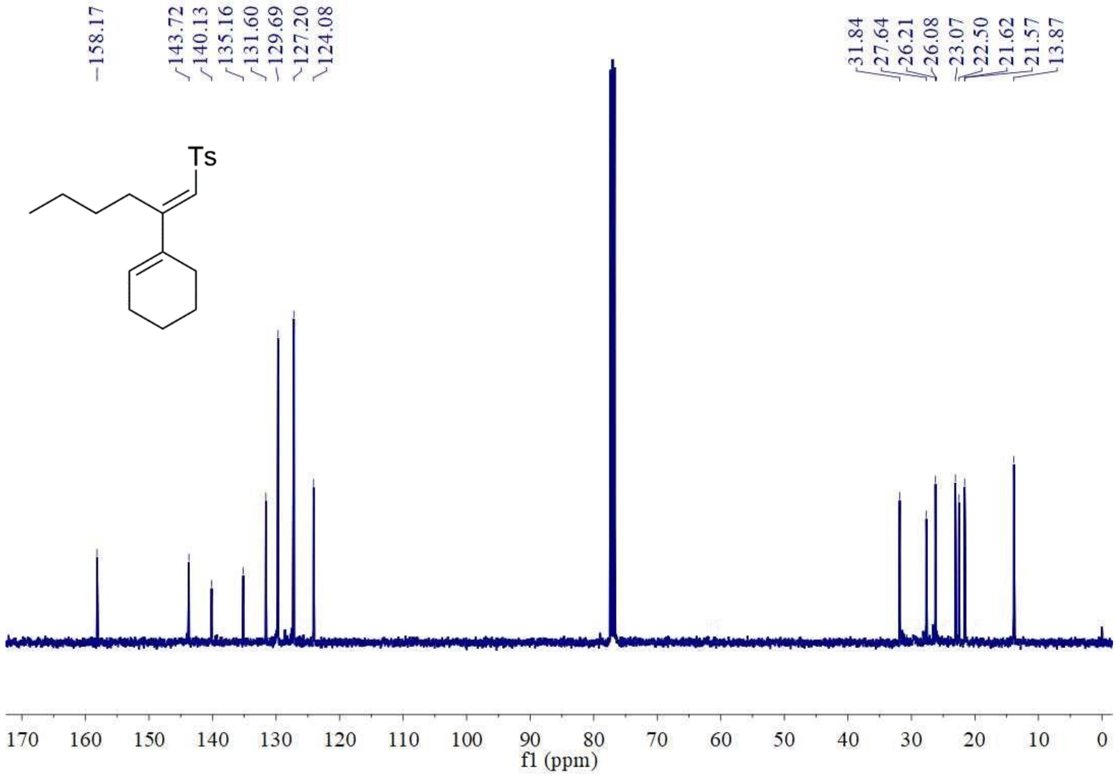

Supplementary Figure 181: ^{13}C NMR of 40b (100 MHz, $CDCl_3$).

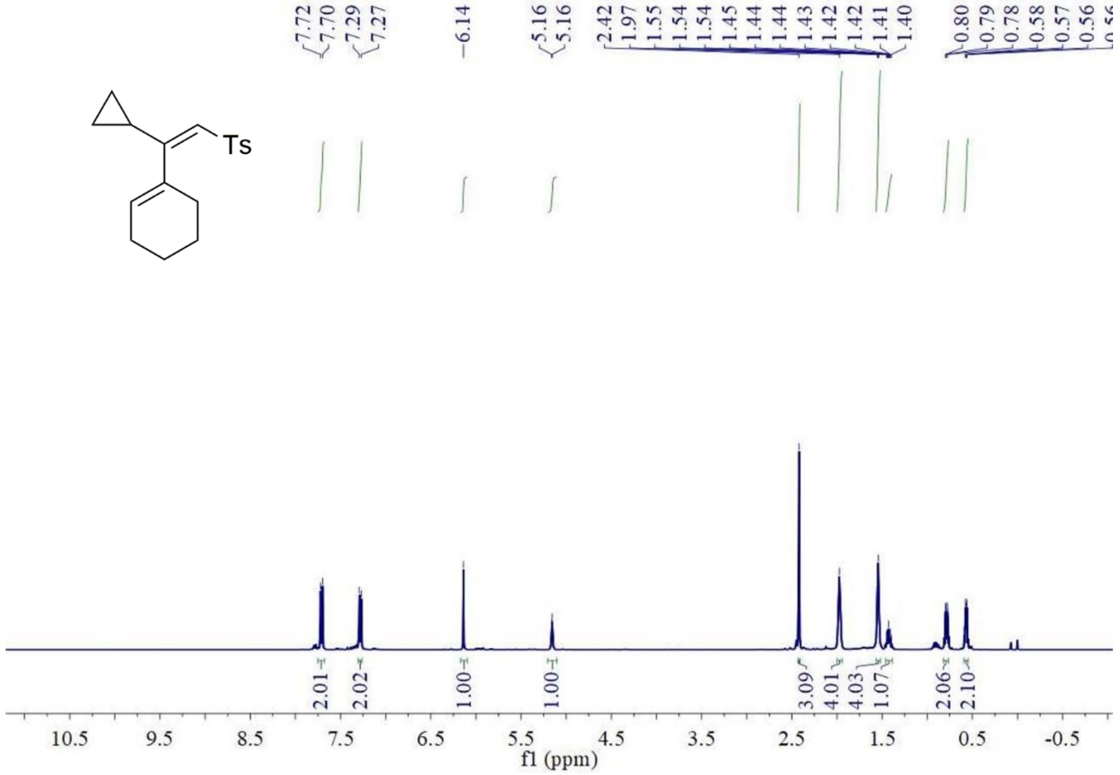

Supplementary Figure 182: ^{1}H NMR of 41a (400 MHz, $CDCl_3$).

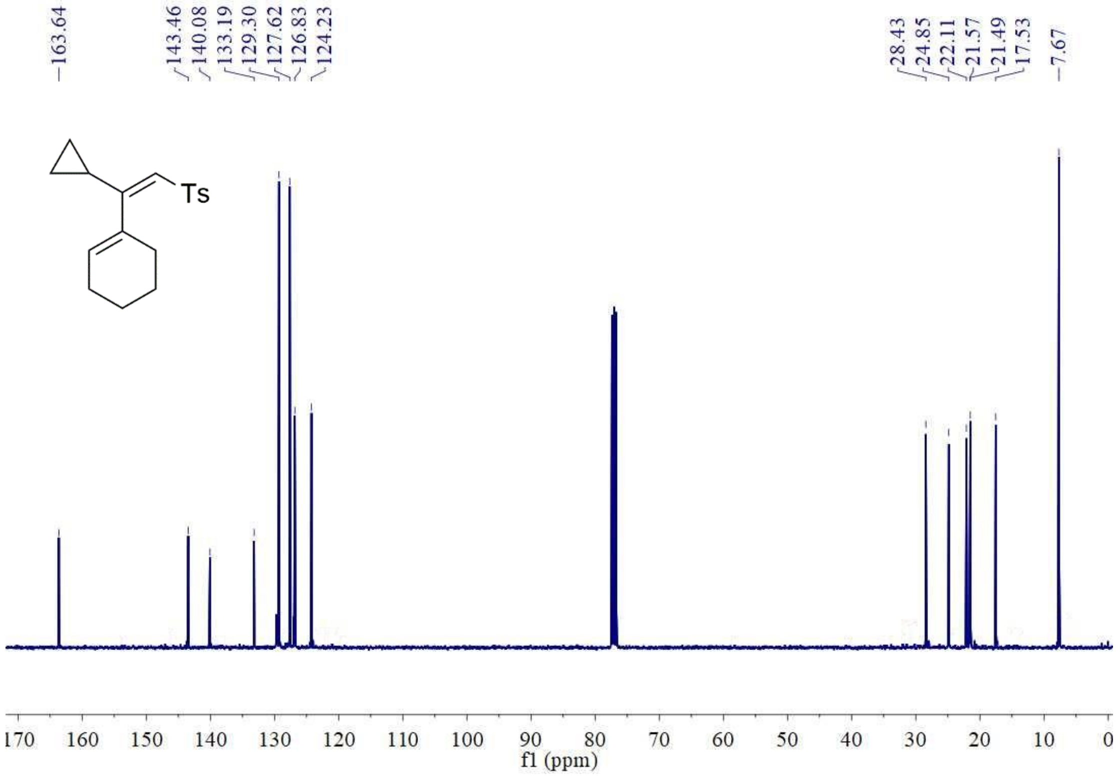

Supplementary Figure 183: ^{13}C NMR of 41a (100 MHz, $CDCl_3$).

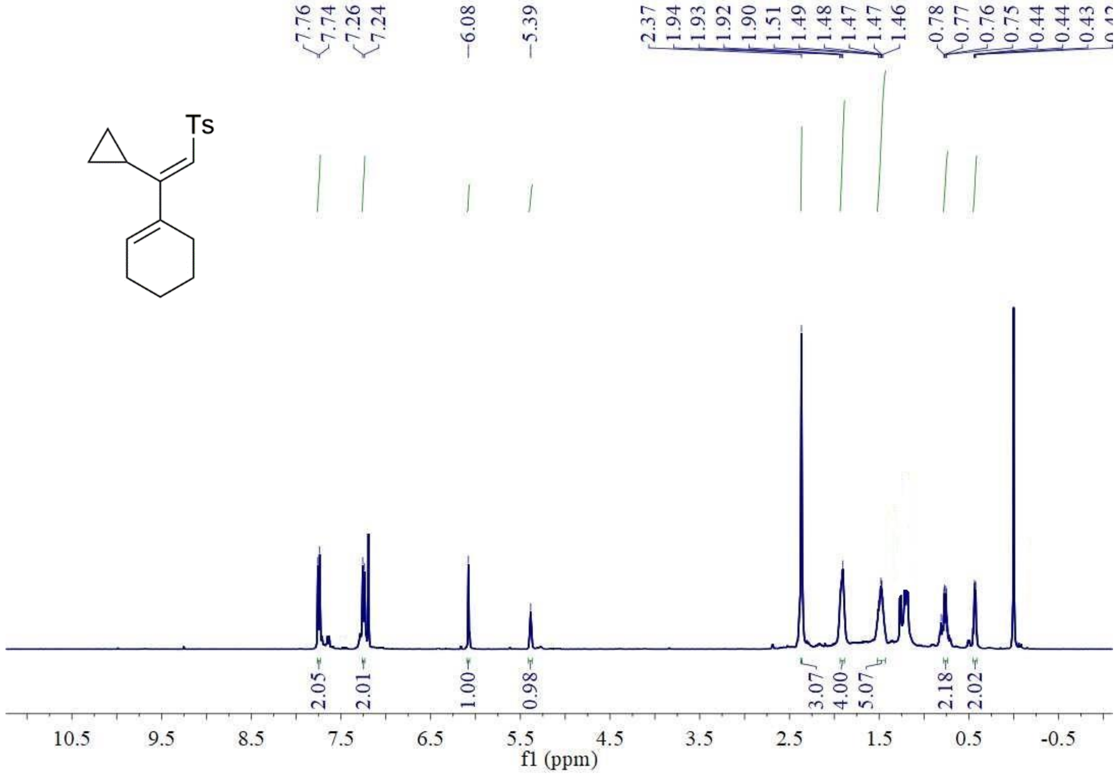

Supplementary Figure 184: ¹H NMR of 41b (400 MHz, $CDCl_3$).

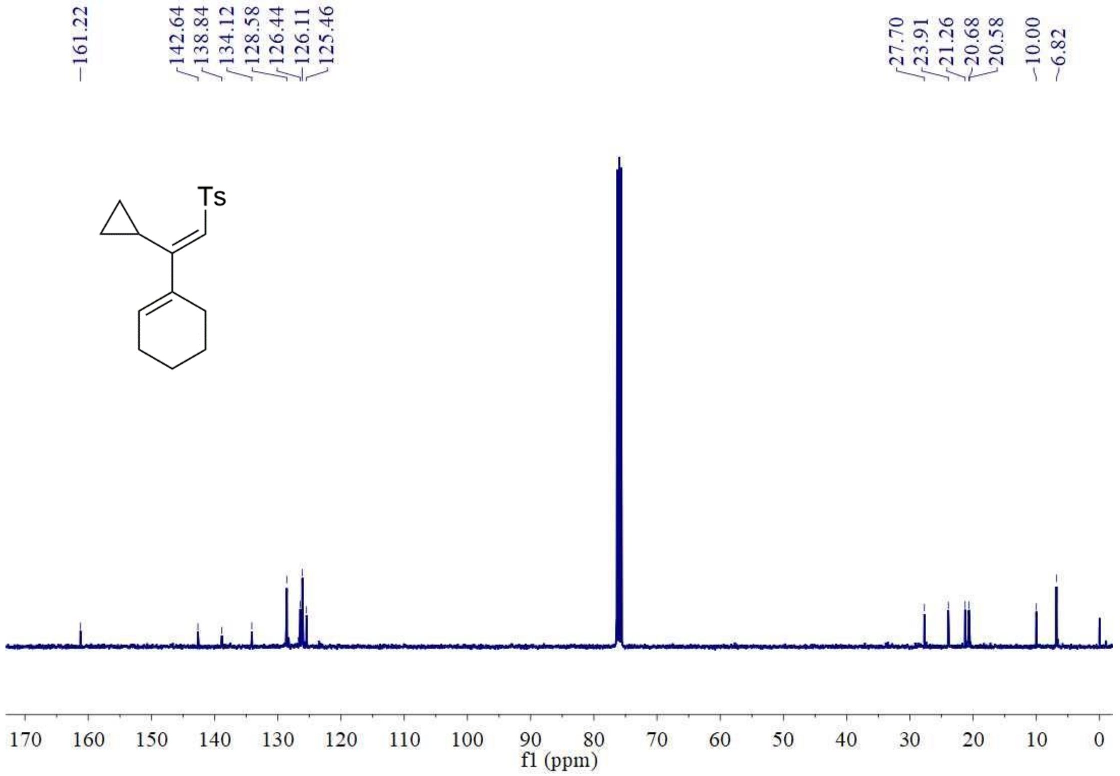

Supplementary Figure 185: ¹³C NMR of 41b (100 MHz, $CDCl_3$).

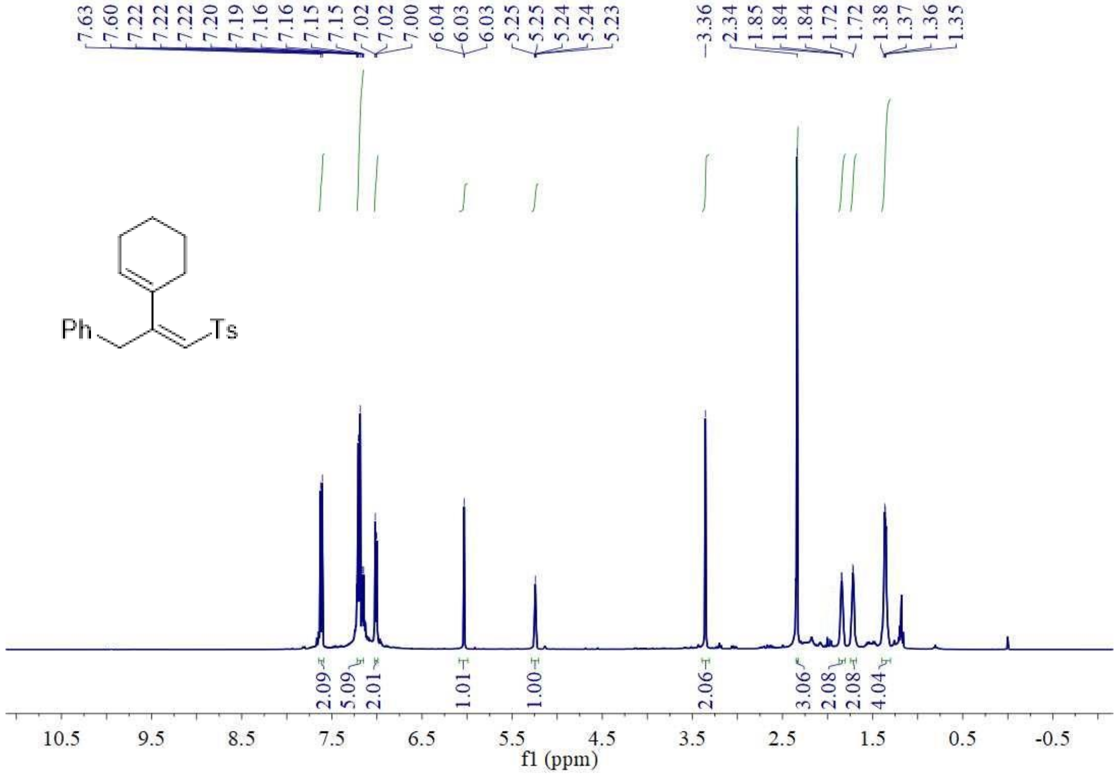

Supplementary Figure 186: ^{1}H NMR of 42a (400 MHz, $CDCl_3$).

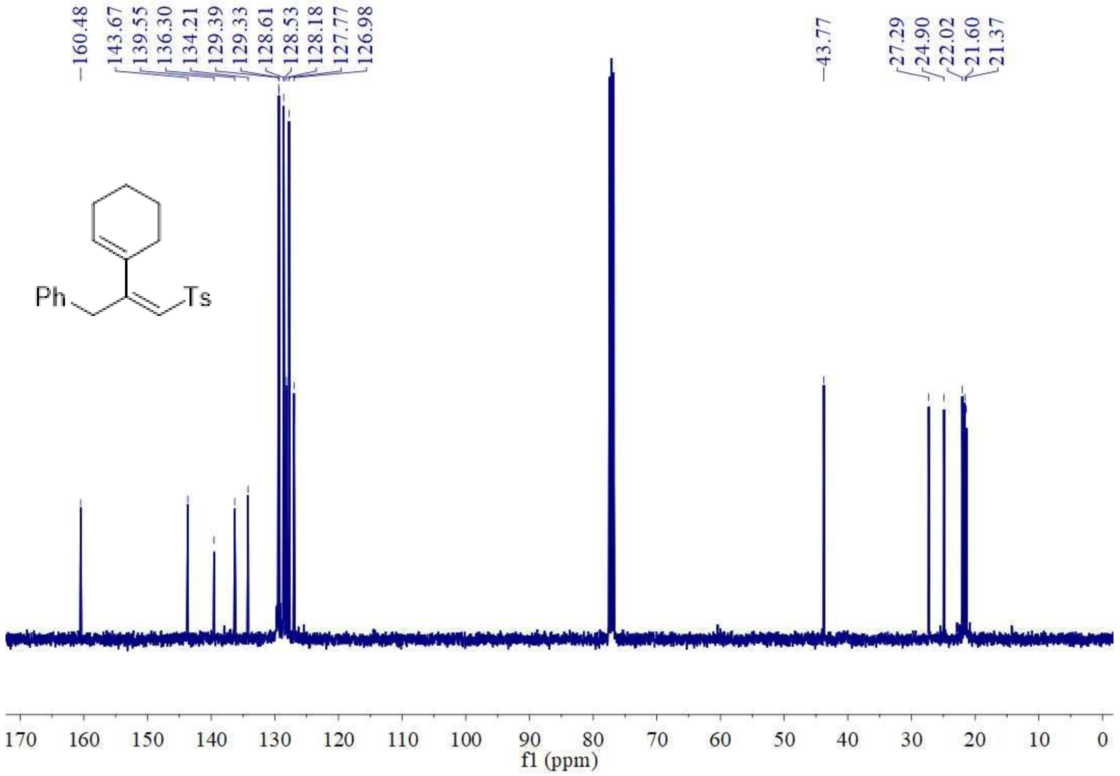

Supplementary Figure 187: ^{13}C NMR of 42a (100 MHz, $CDCl_3$).

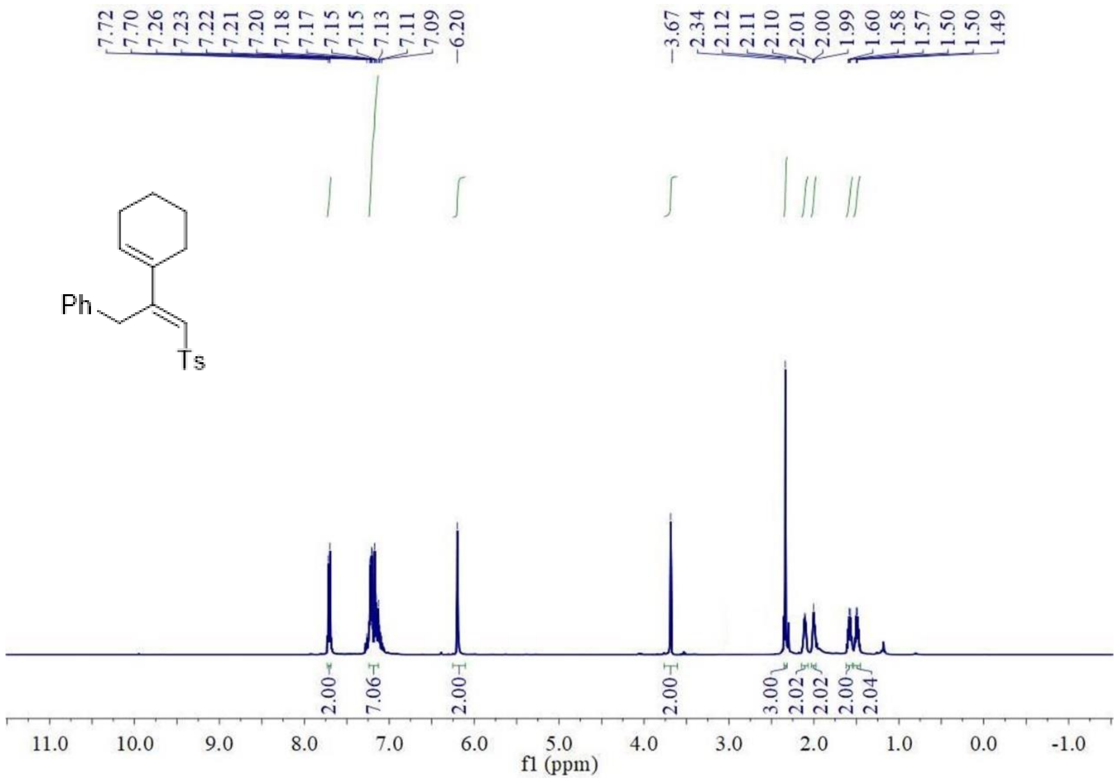

Supplementary Figure 188: ¹H NMR of 42b (400 MHz, $CDCl_3$).

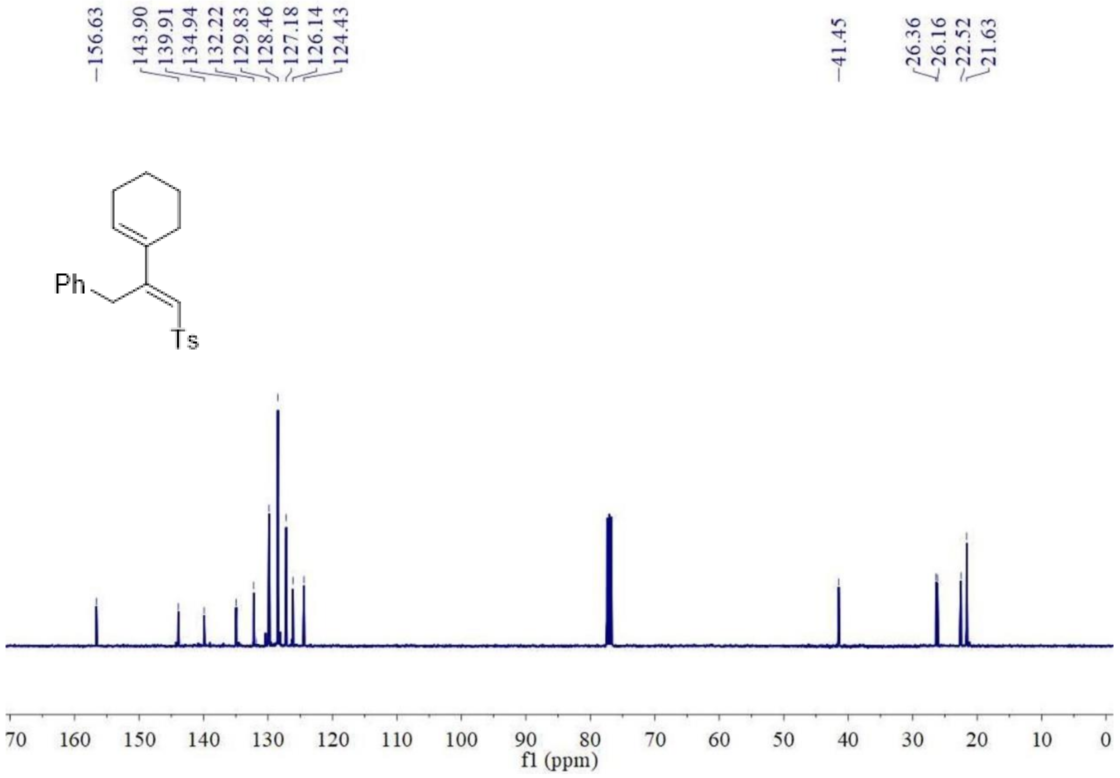

Supplementary Figure 189: ¹³C NMR of 42b (100 MHz, $CDCl_3$).

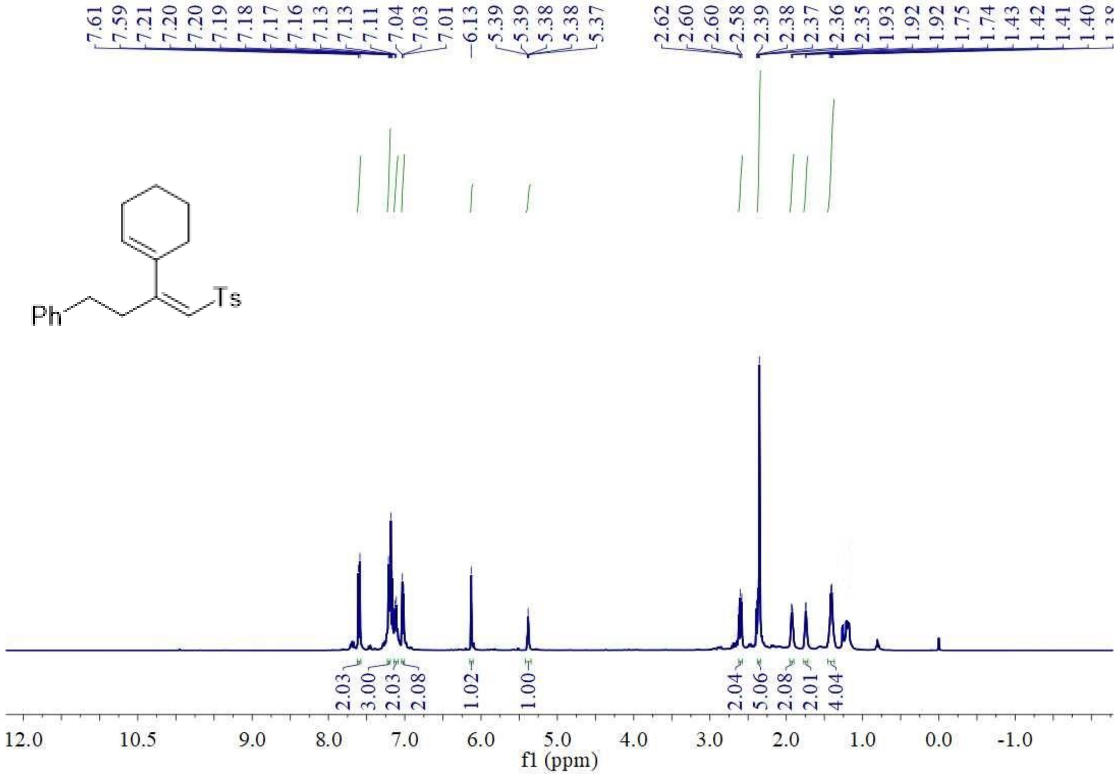

Supplementary Figure 190: ^{1}H NMR of 43a (400 MHz, $CDCl_3$).

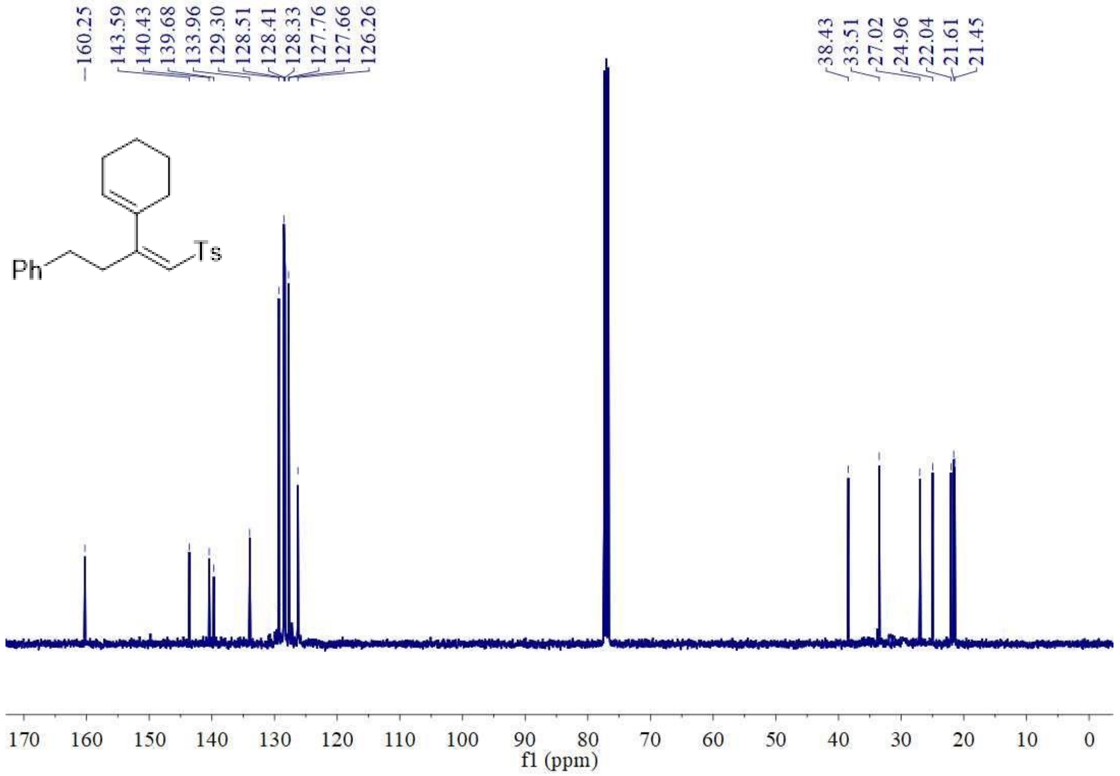

Supplementary Figure 191: ^{13}C NMR of 43a (100 MHz, $CDCl_3$).

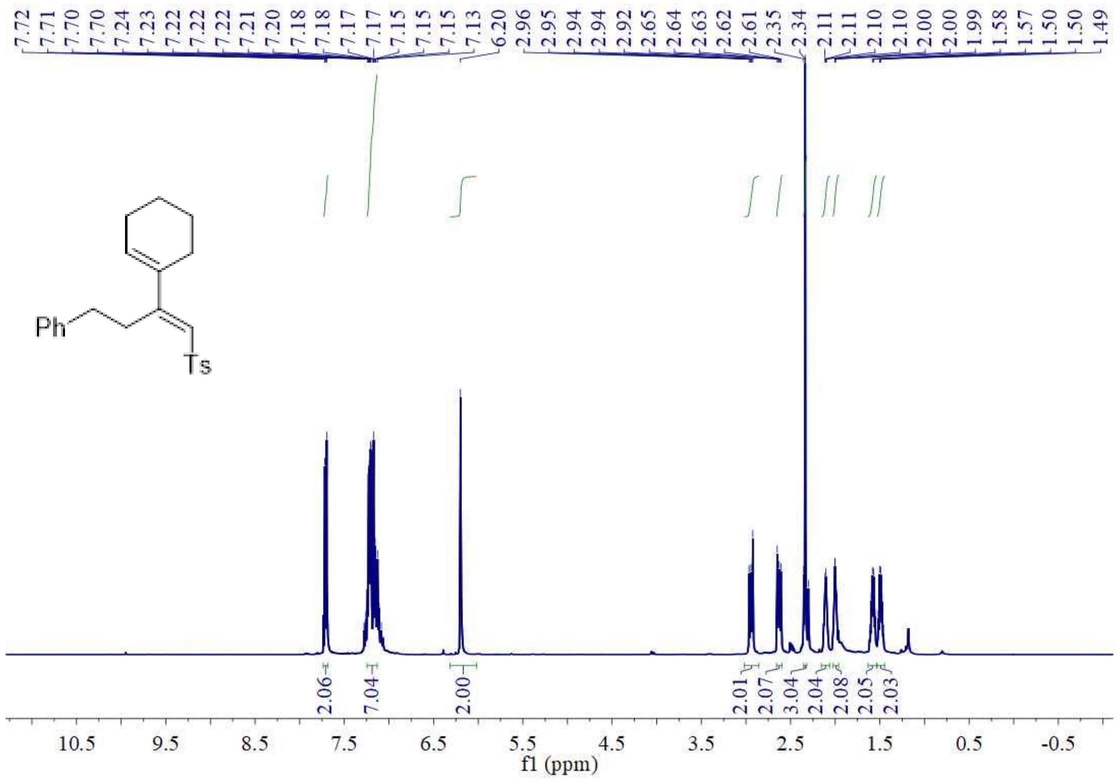

Supplementary Figure 192: ^{1}H NMR of 43b (400 MHz, $CDCl_3$).

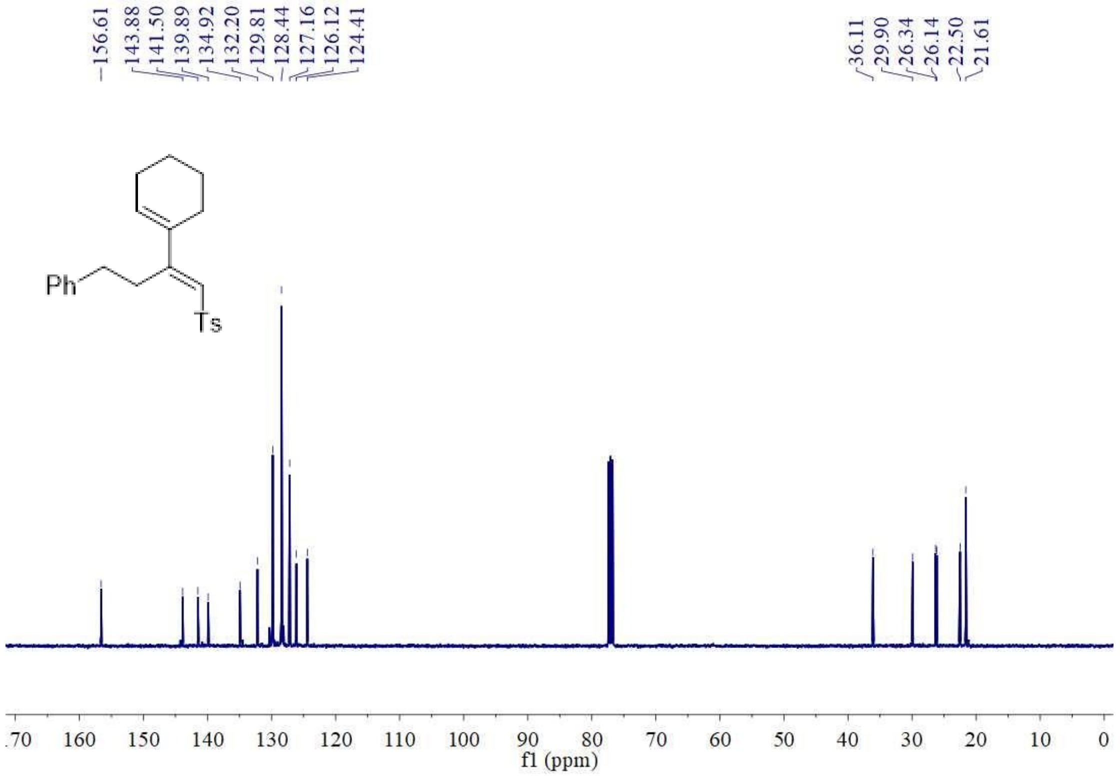

Supplementary Figure 193: ^{13}C NMR of 43b (100 MHz, $CDCl_3$).

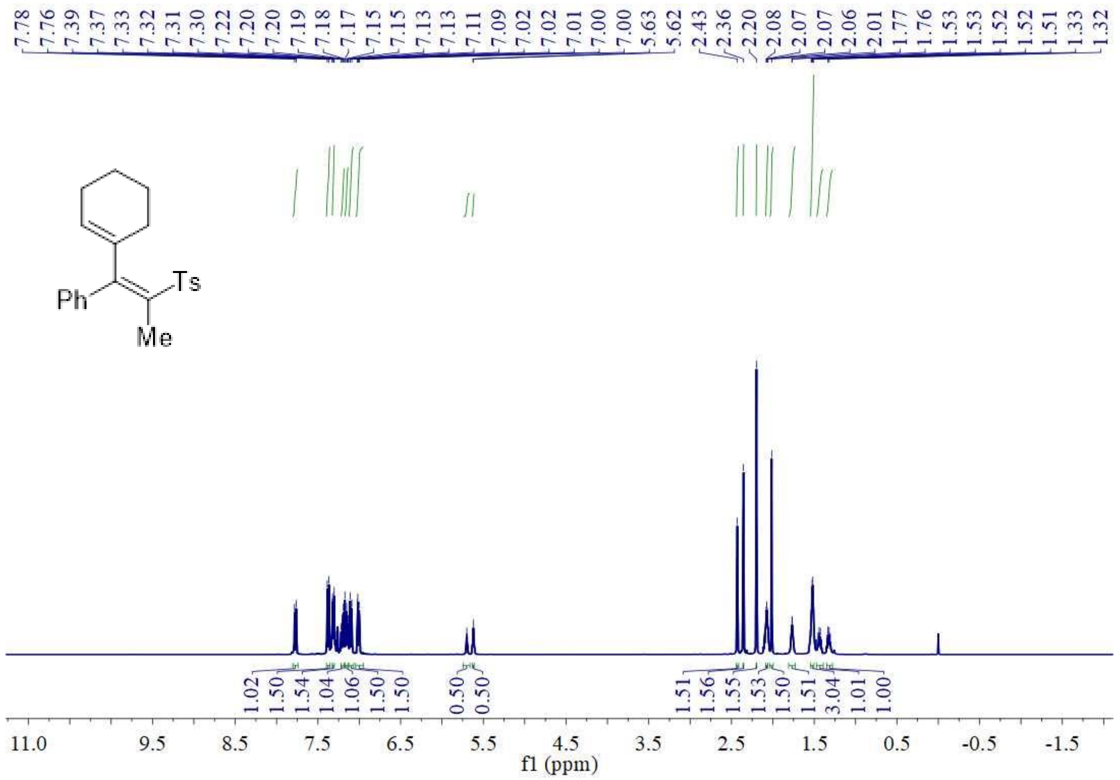

Supplementary Figure 194: ^{1}H NMR of 44a (400 MHz, $CDCl_3$).

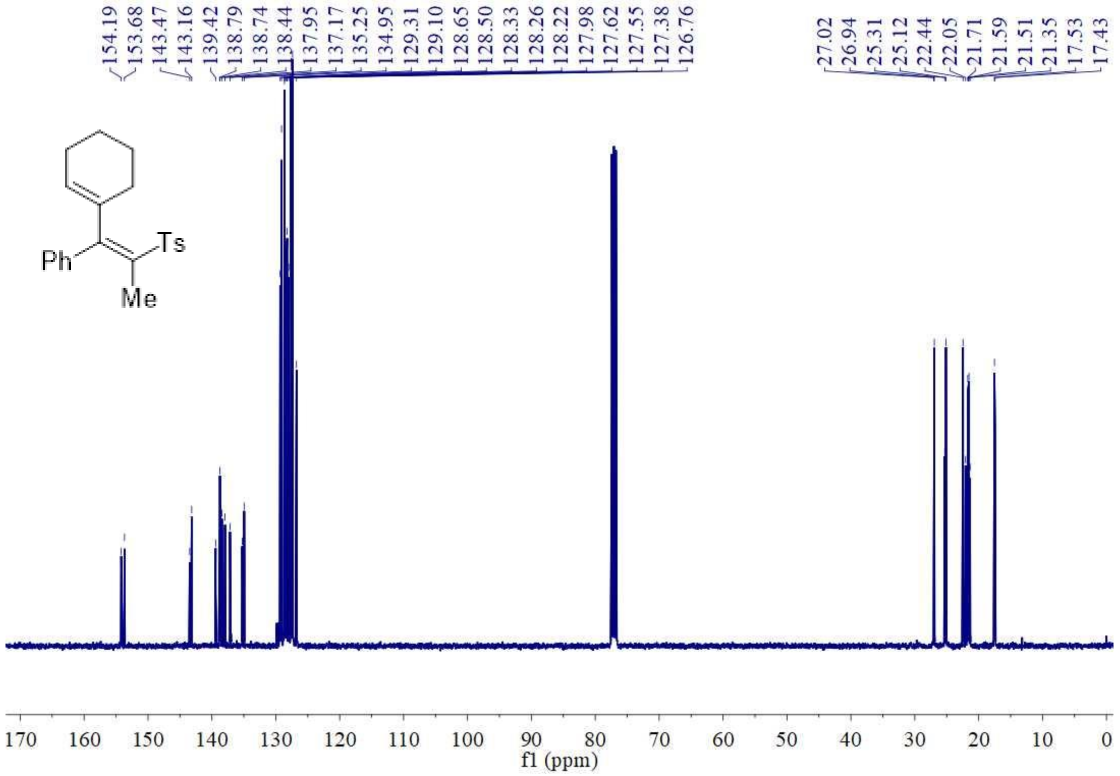

Supplementary Figure 195: ^{13}C NMR of 44a (100 MHz, $CDCl_3$).

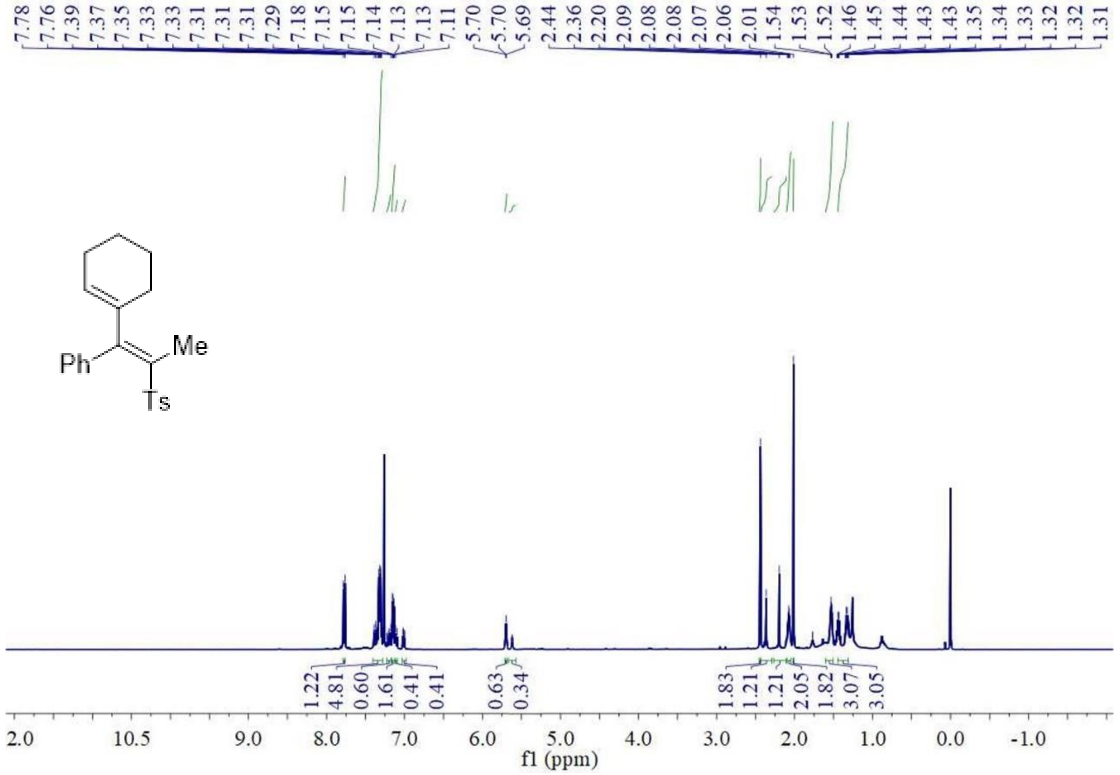

Supplementary Figure 196: ^{1}H NMR of 44b (400 MHz, $CDCl_3$).

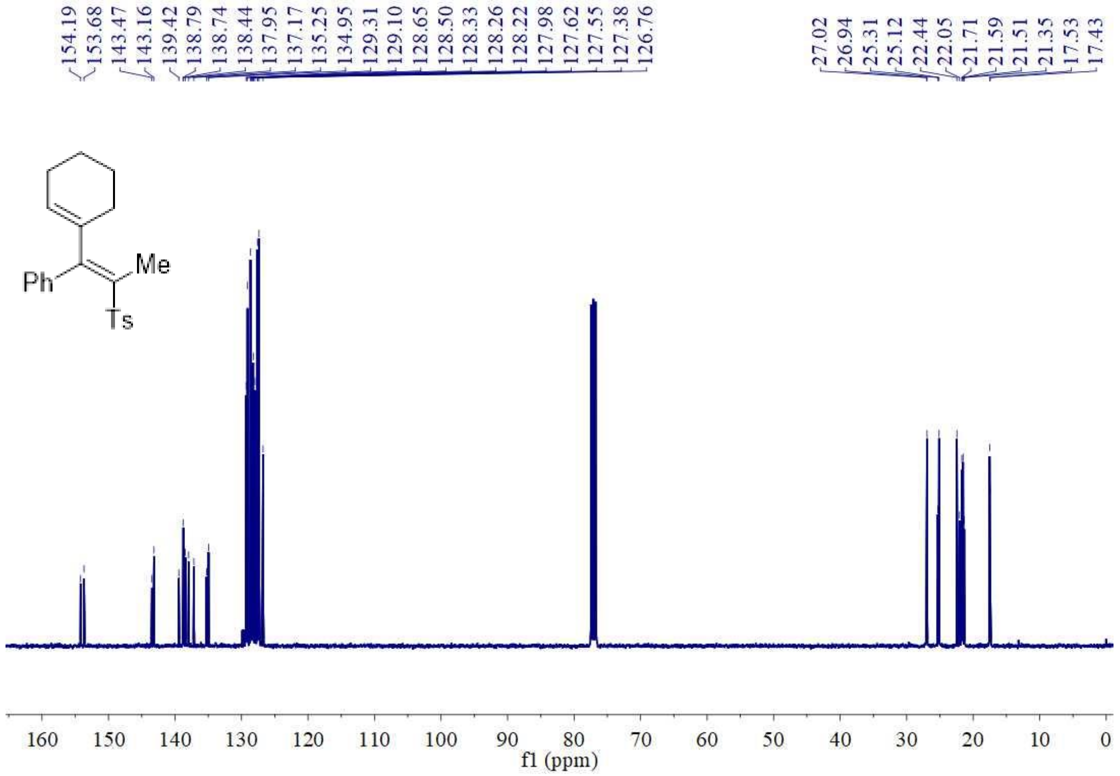

Supplementary Figure 197: ^{13}C NMR of 44b (100 MHz, $CDCl_3$).

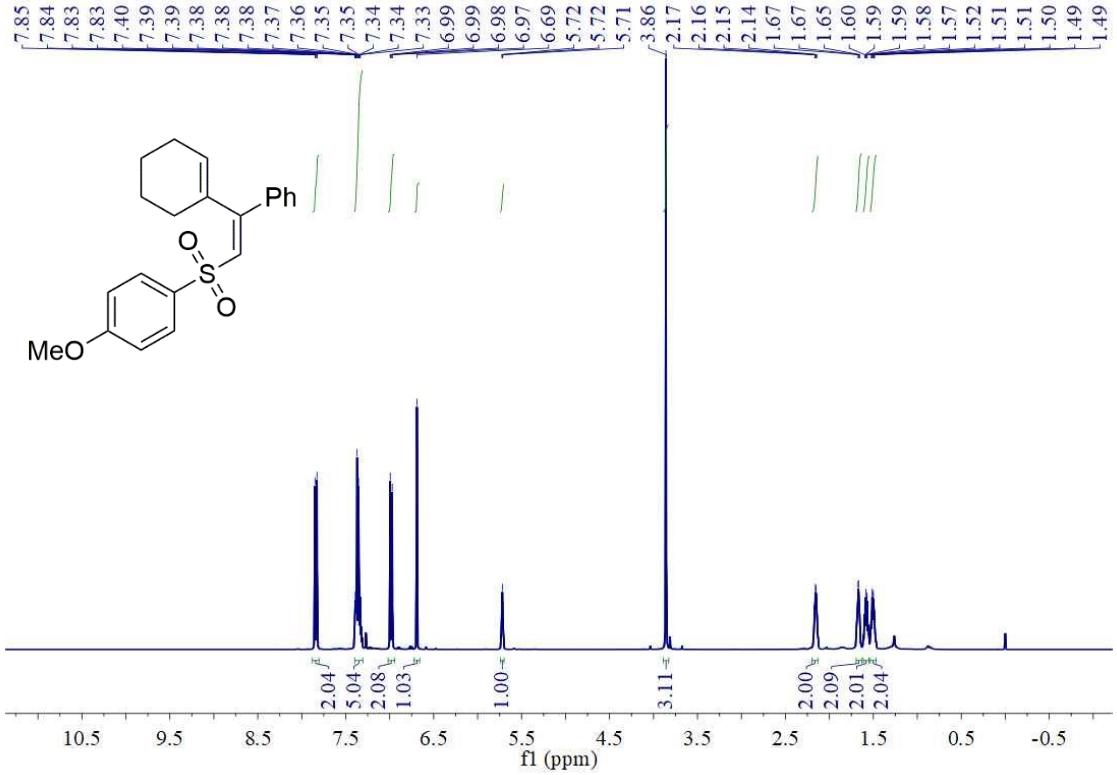

Supplementary Figure 198: ^{1}H NMR of 45a (400 MHz, $CDCl_3$).

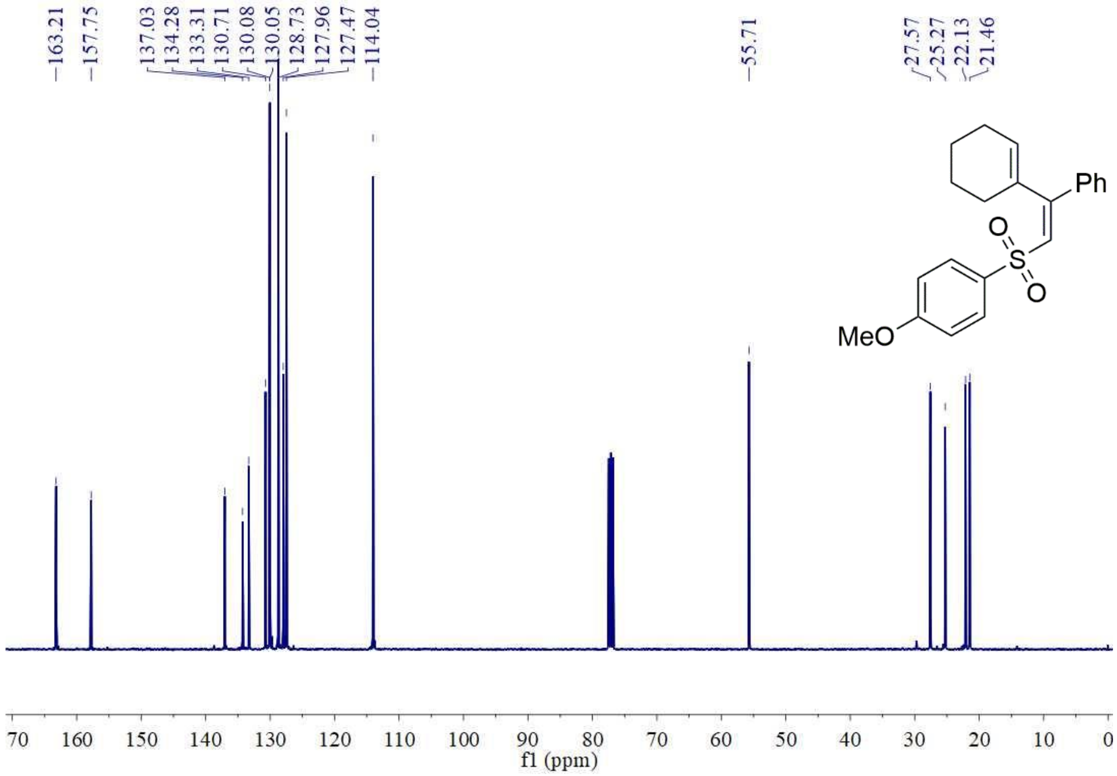

Supplementary Figure 199: ^{13}C NMR of 45a (100 MHz, $CDCl_3$).

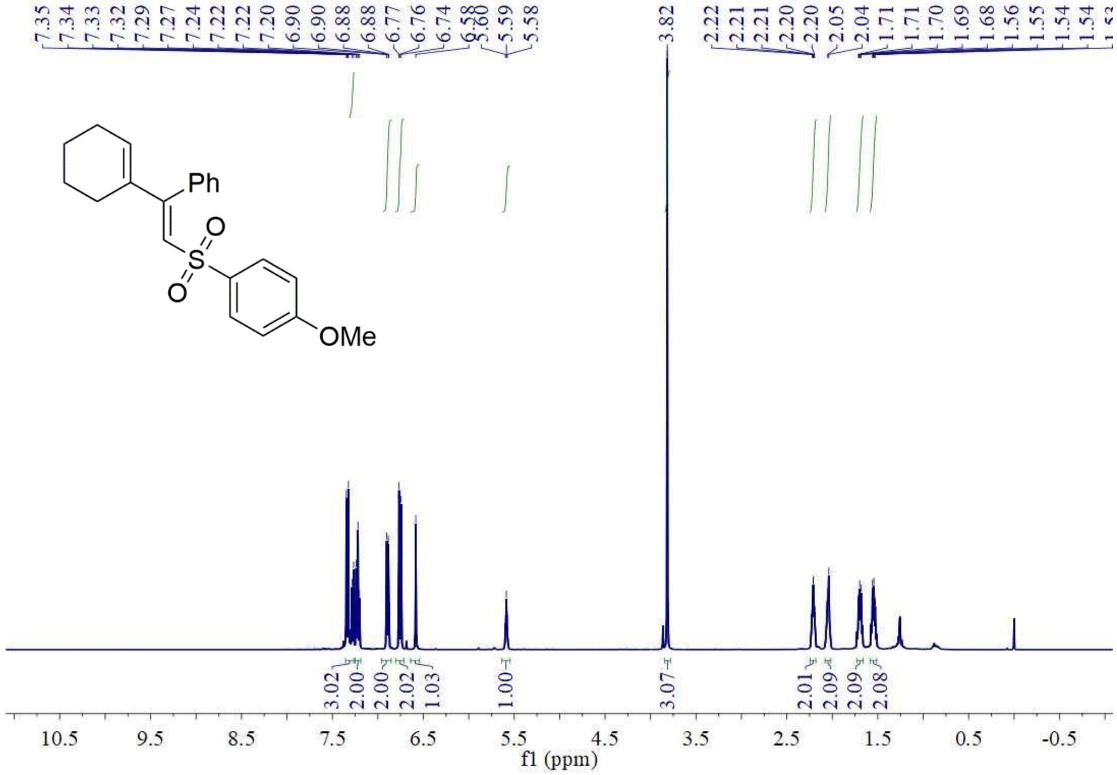

Supplementary Figure 200: ^{1}H NMR of 45b (400 MHz, $CDCl_3$).

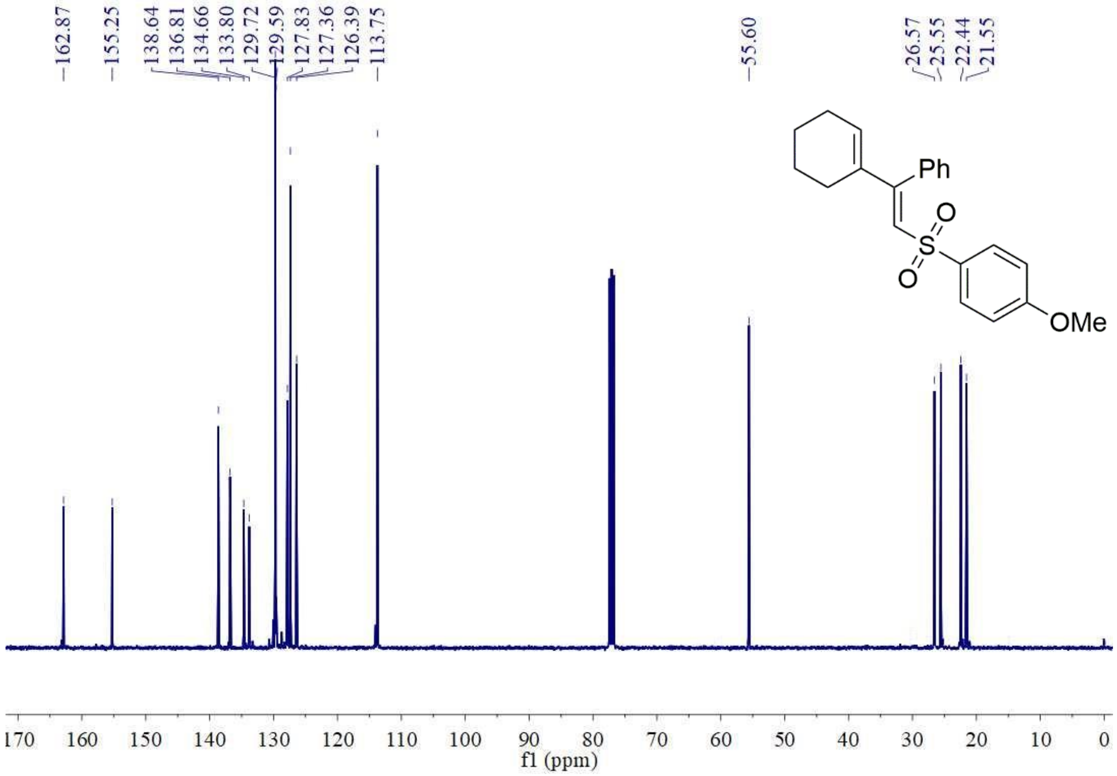

Supplementary Figure 201: ^{13}C NMR of 45b (100 MHz, $CDCl_3$).

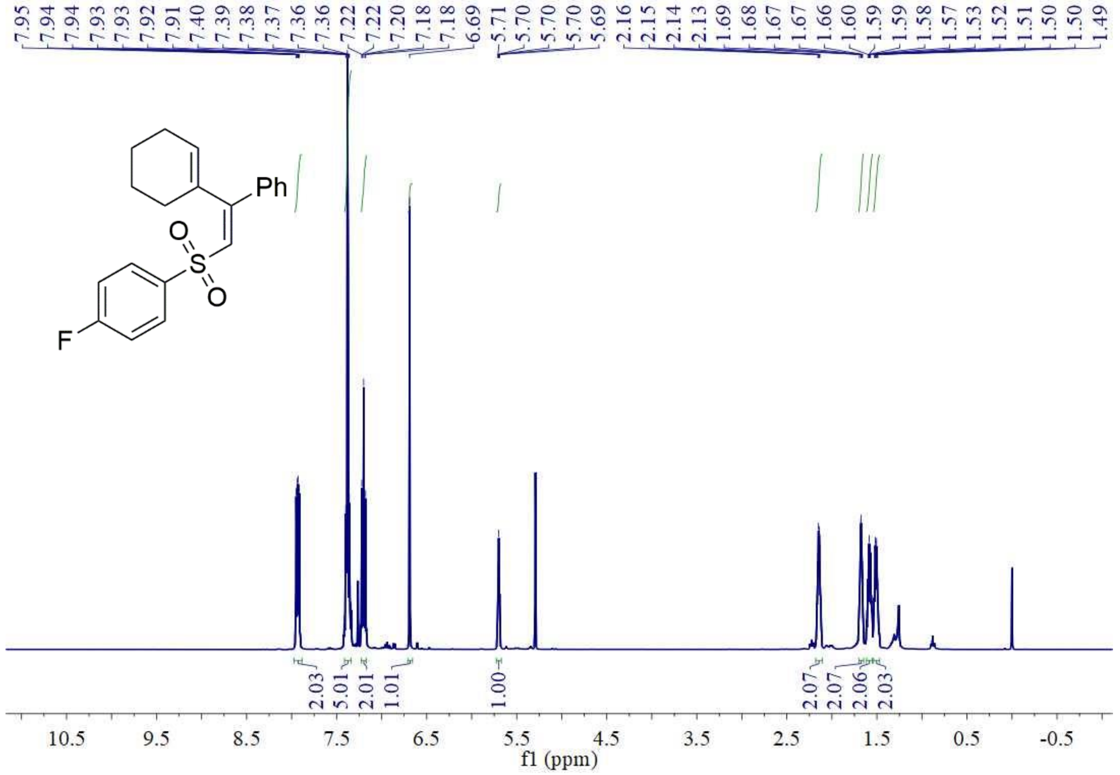

Supplementary Figure 202: ^{1}H NMR of 46a (400 MHz, $CDCl_3$).

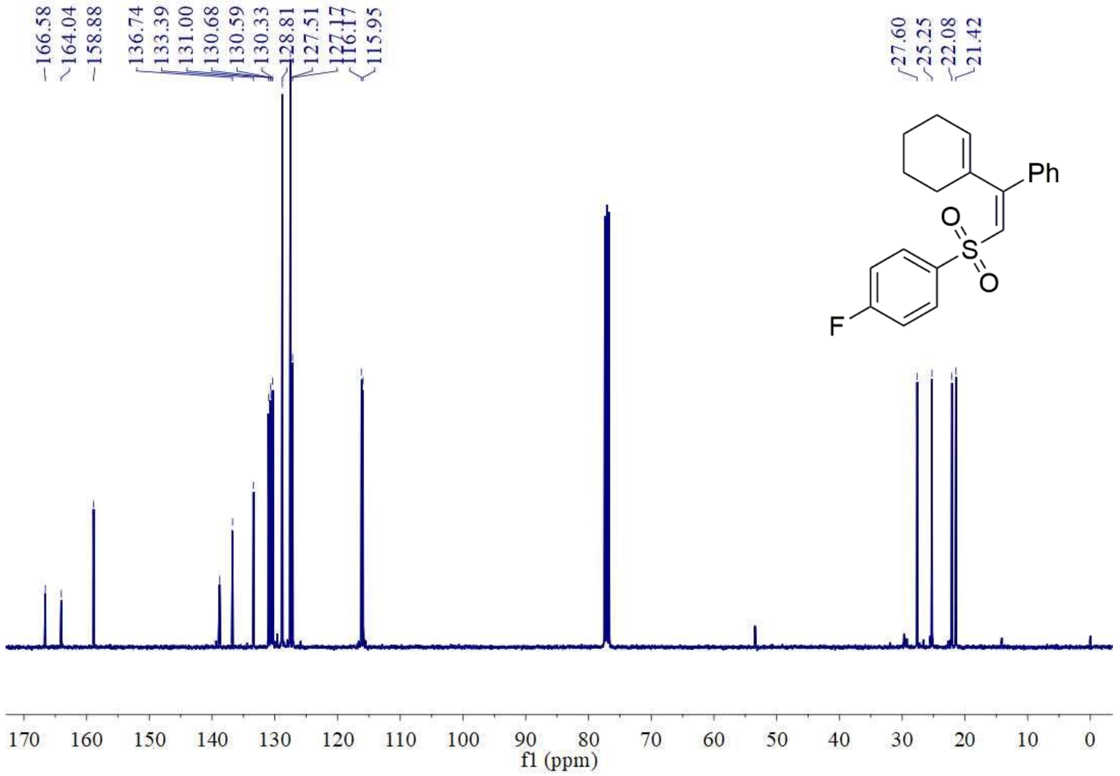

Supplementary Figure 203: ^{13}C NMR of 46a (100 MHz, $CDCl_3$).

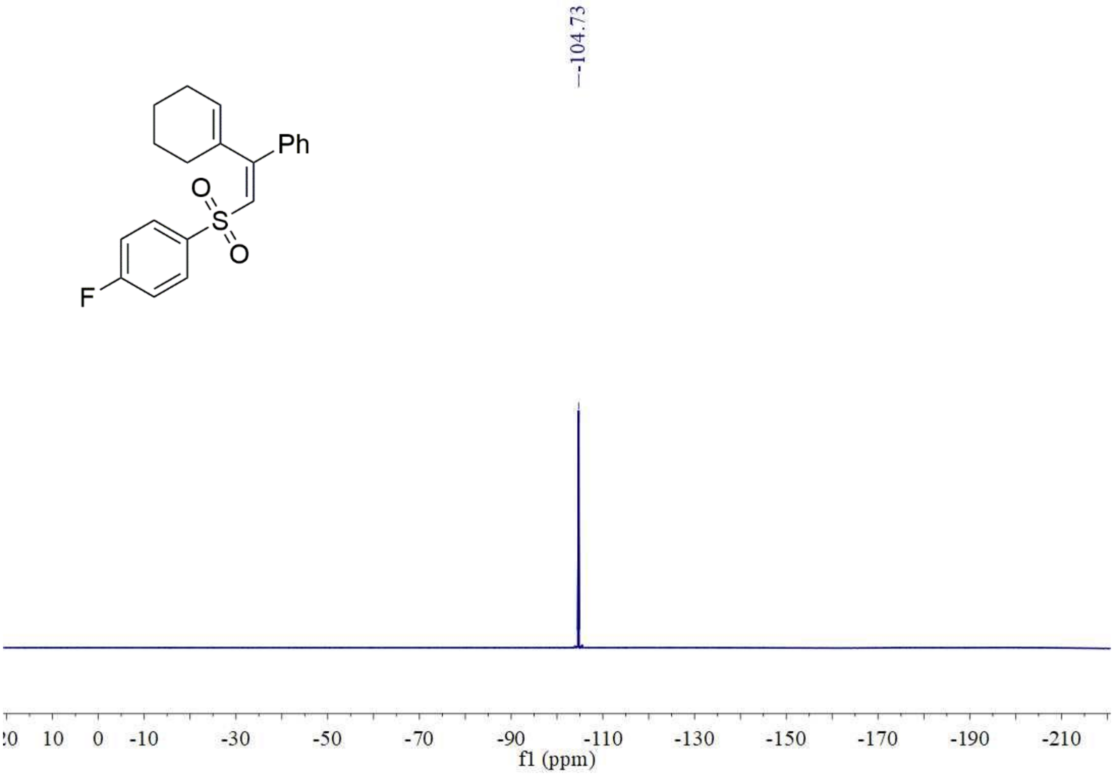

Supplementary Figure 204: ^{19}F NMR of 46a (377 MHz, $CDCl_3$).

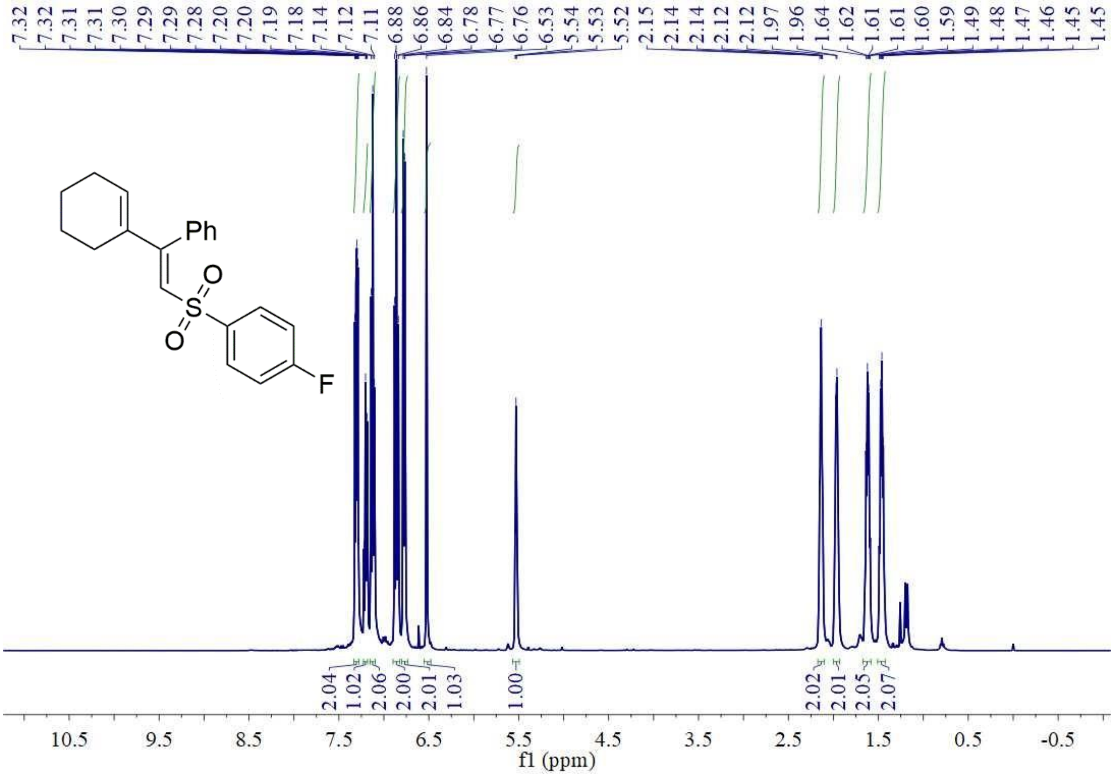

Supplementary Figure 205: ^{1}H NMR of 46b (400 MHz, $CDCl_3$).

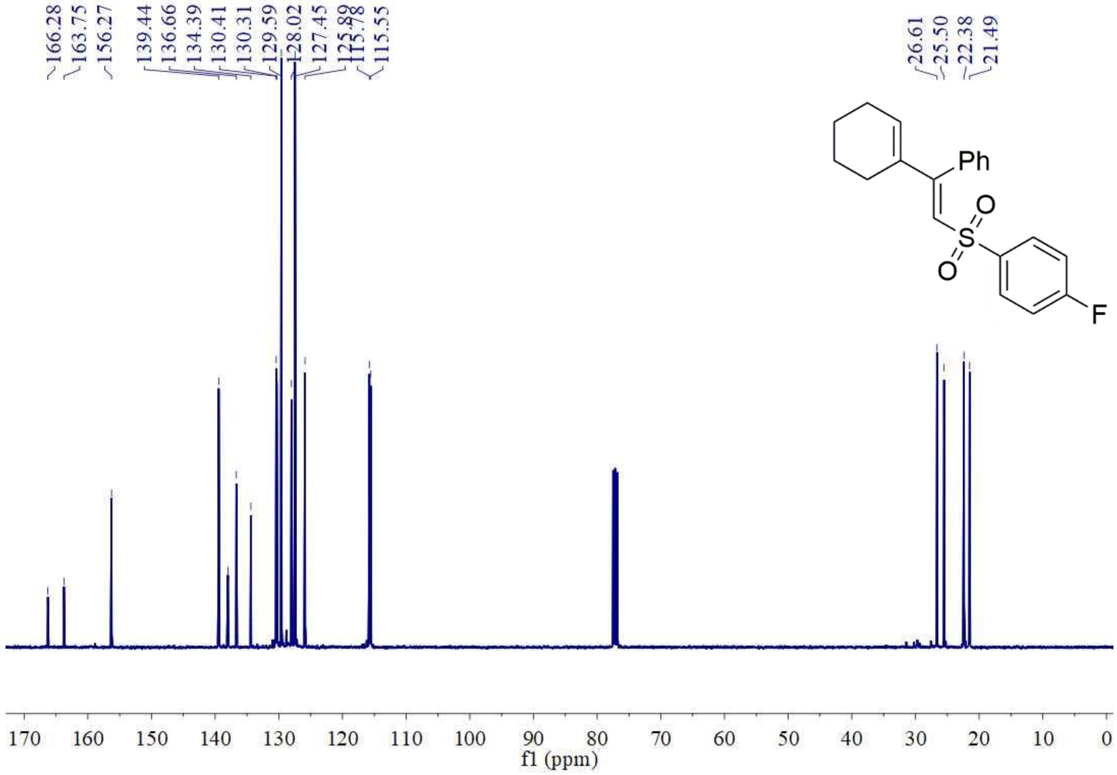

Supplementary Figure 206: ^{13}C NMR of 46b (100 MHz, $CDCl_3$).

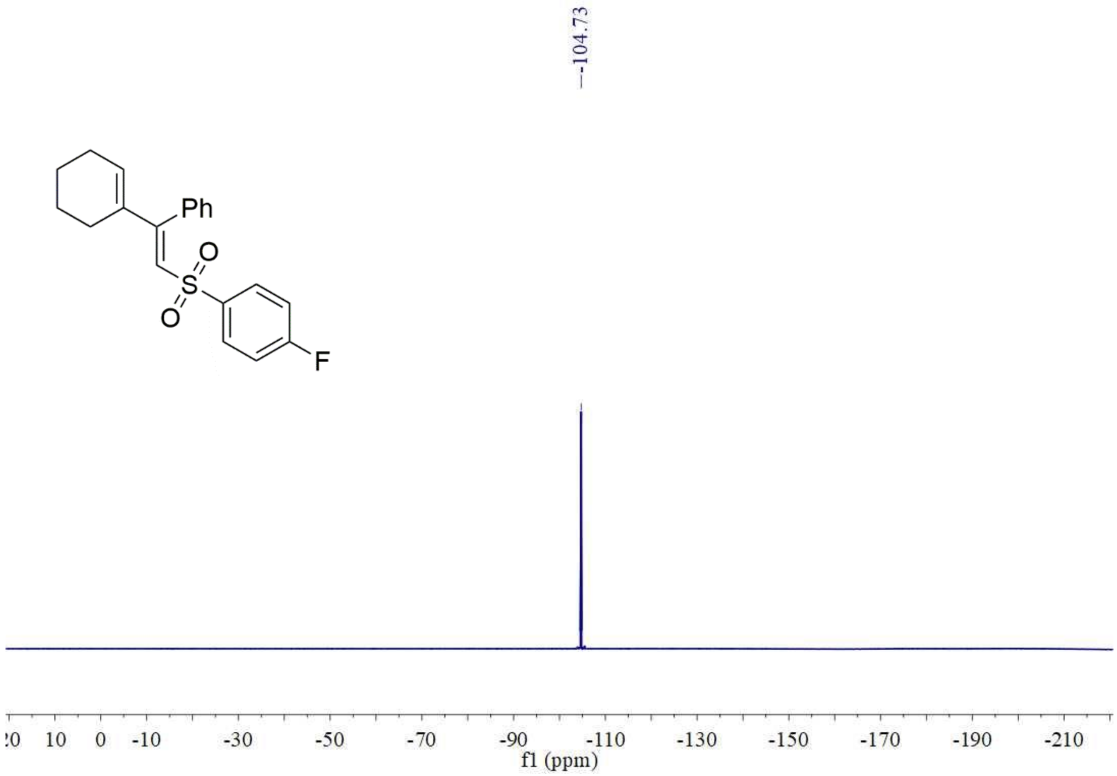

Supplementary Figure 207: ^{19}F NMR of 46b (377 MHz, $CDCl_3$).

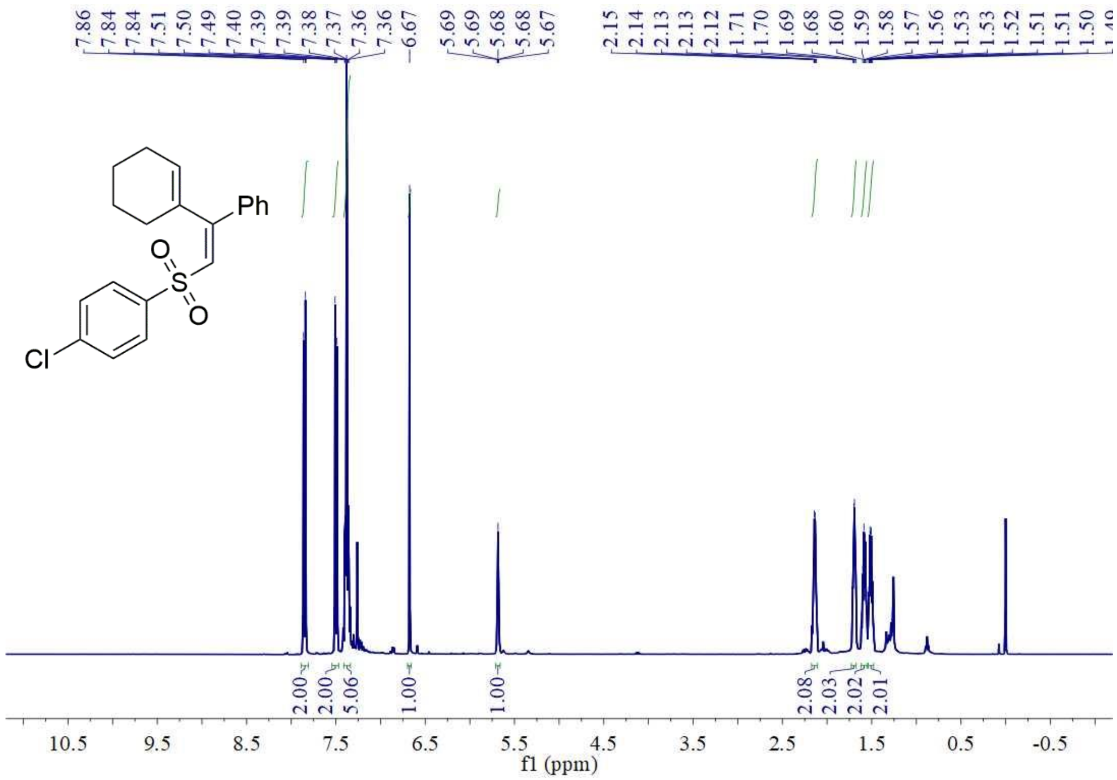

Supplementary Figure 208: ^{1}H NMR of 47a (400 MHz, $CDCl_3$).

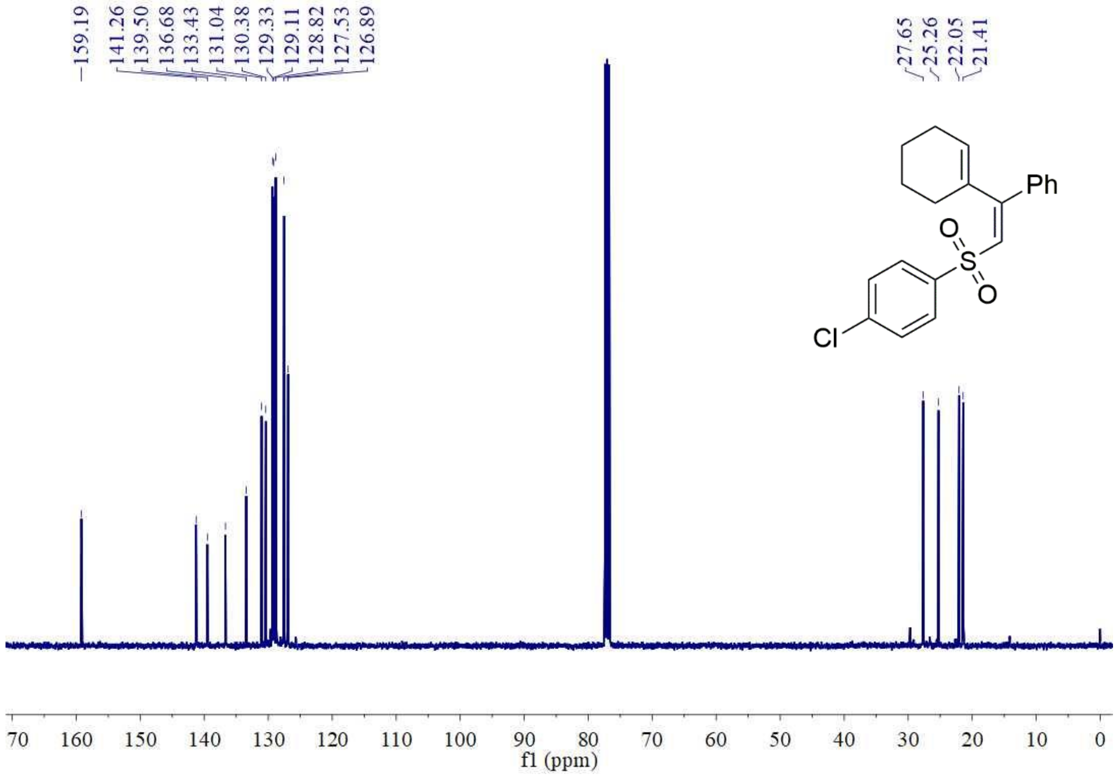

Supplementary Figure 209: ^{13}C NMR of 47a (100 MHz, $CDCl_3$).

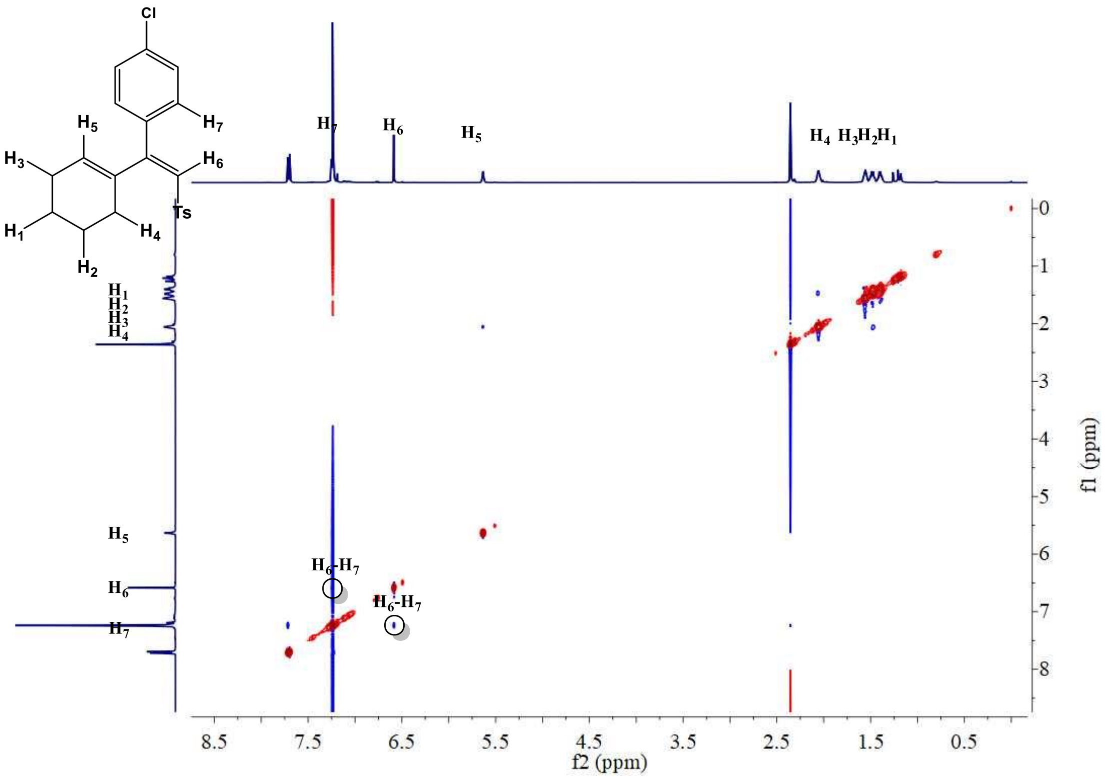

Supplementary Figure 210: NOESY of 47a.

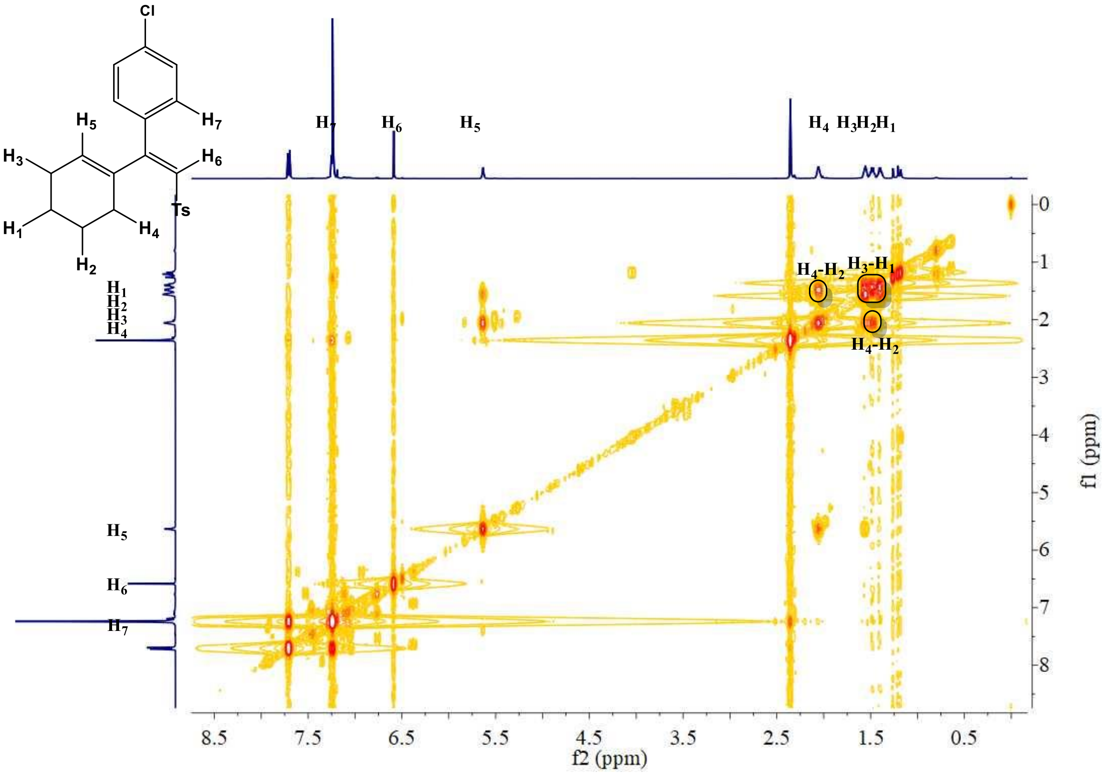

Supplementary Figure 211: COSY of 47a.

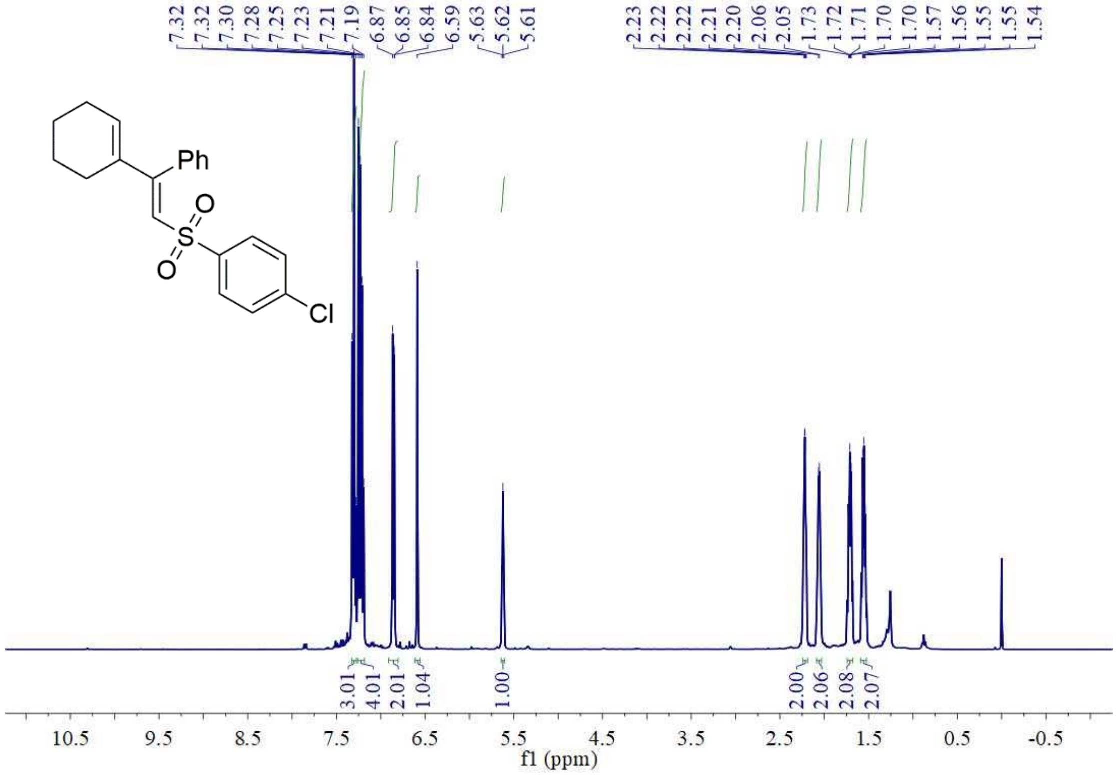

Supplementary Figure 212: ^{1}H NMR of 47b (400 MHz, $CDCl_3$).

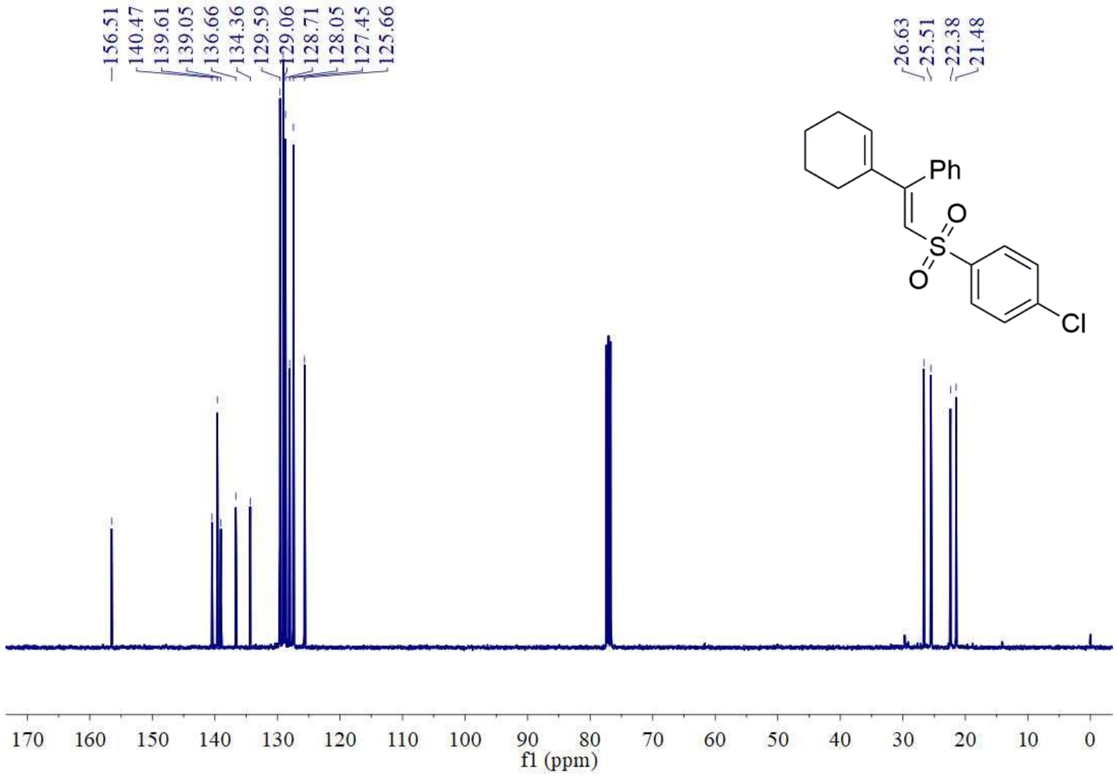

Supplementary Figure 213: ^{13}C NMR of 47b (100 MHz, $CDCl_3$).

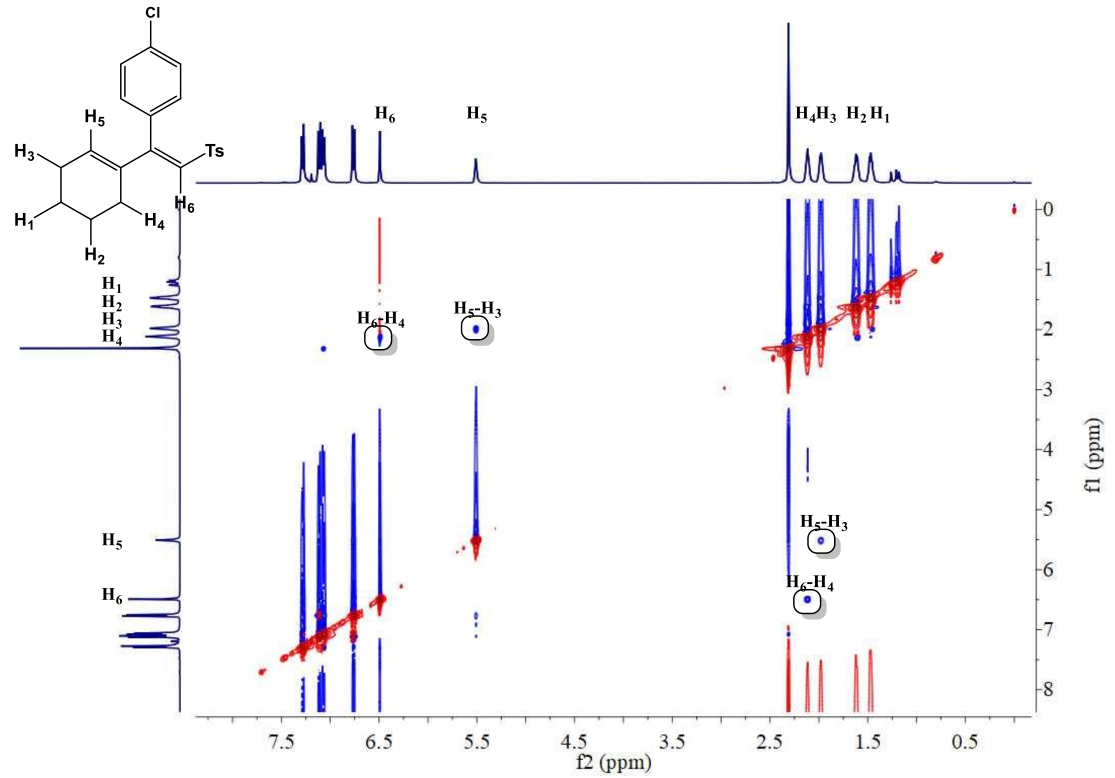

Supplementary Figure 214: NOESY of 47b.

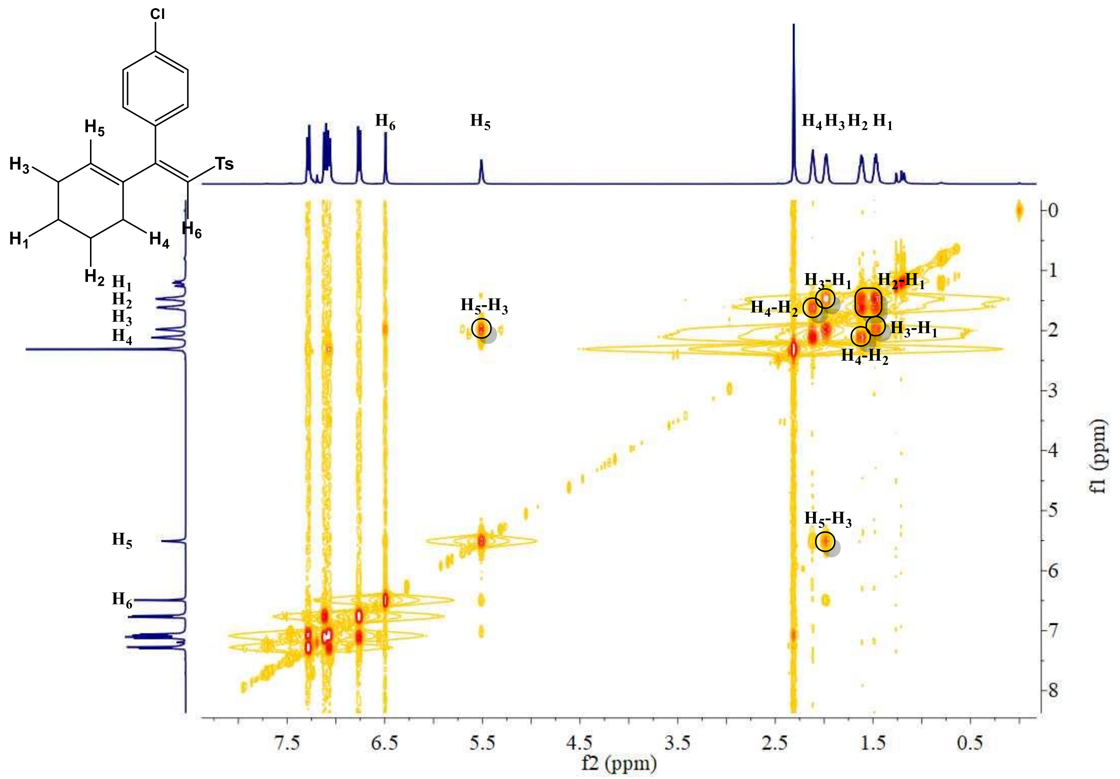

Supplementary Figure 215: COSY of 47b.

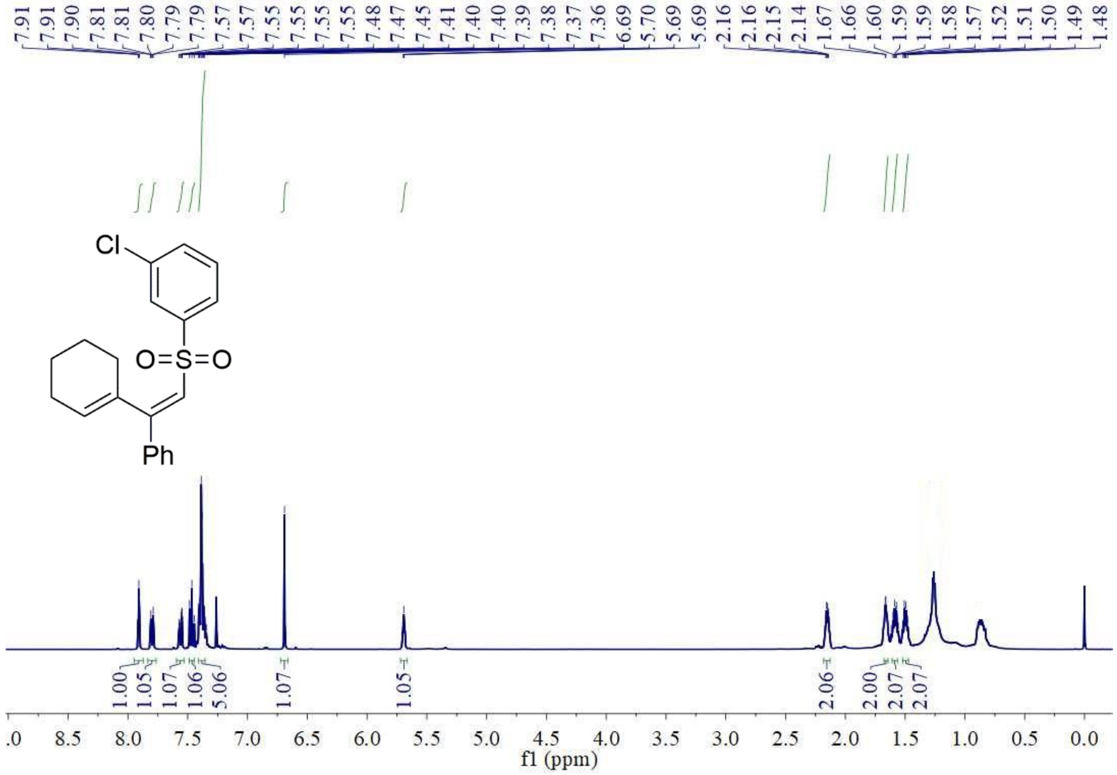

Supplementary Figure 216: ^{1}H NMR of 48a (400 MHz, $CDCl_3$).

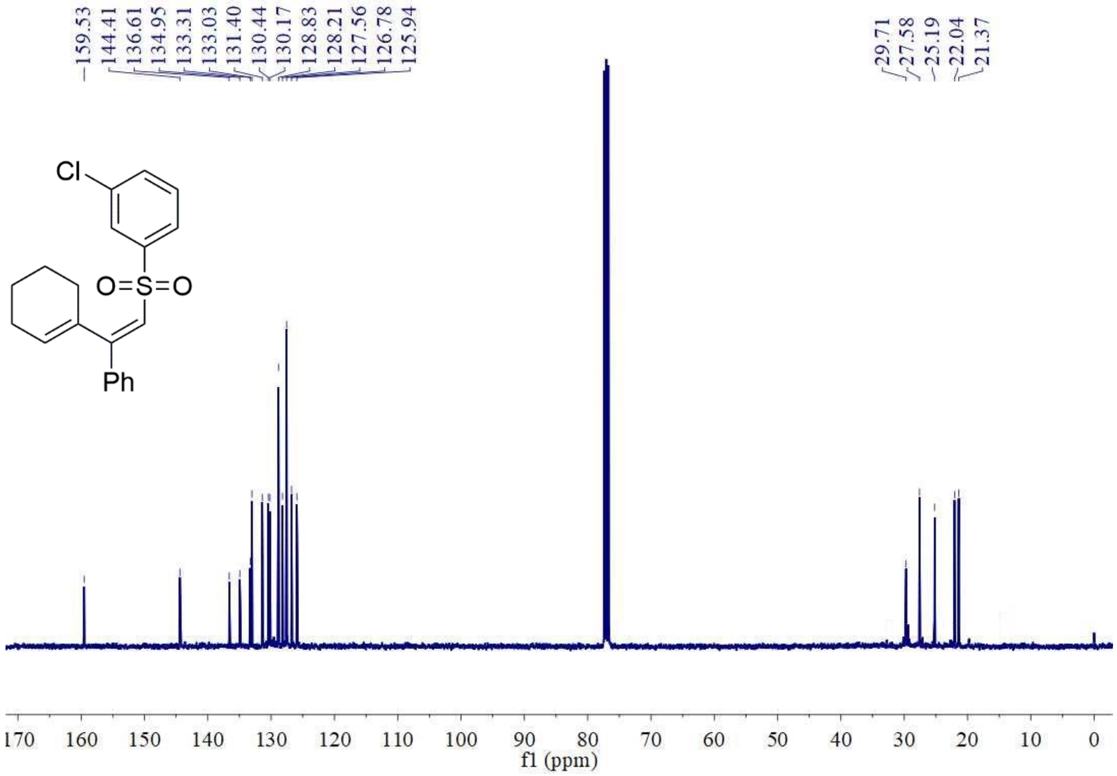

Supplementary Figure 217: ^{13}C NMR of 48a (100 MHz, $CDCl_3$).

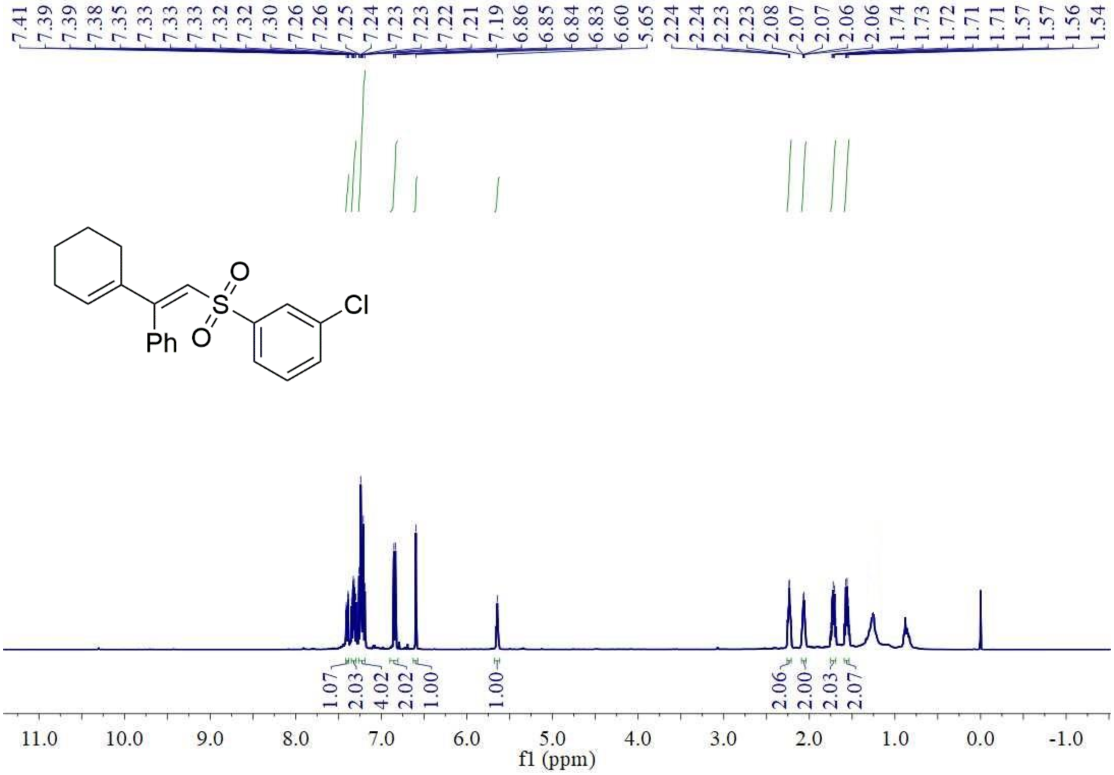

Supplementary Figure 218: ^{1}H NMR of 48b (400 MHz, $CDCl_3$).

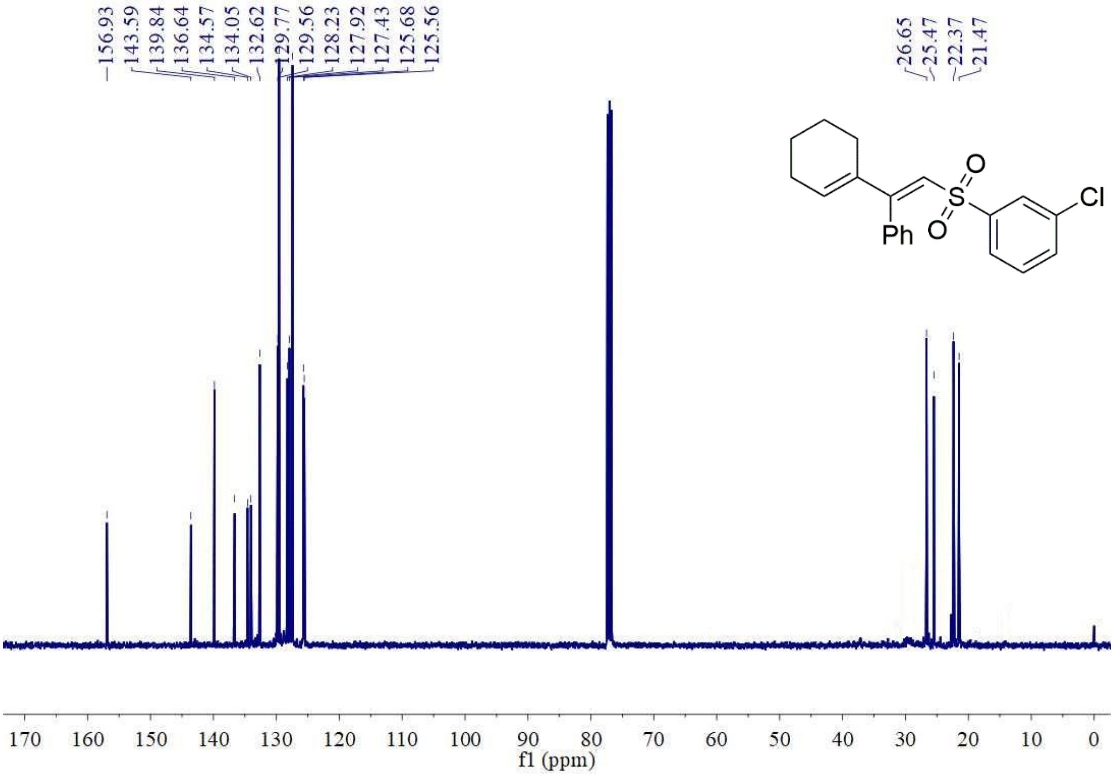

Supplementary Figure 219: ^{13}C NMR of 48b (100 MHz, $CDCl_3$).

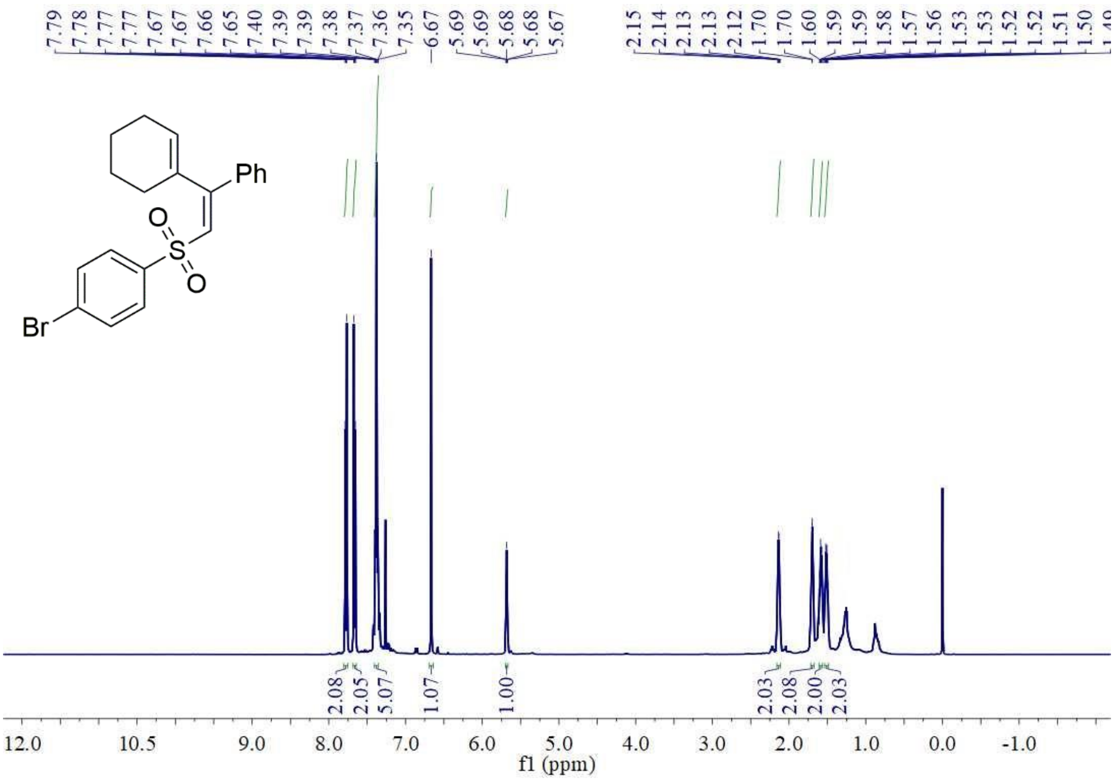

Supplementary Figure 220: ^{1}H NMR of 49a (400 MHz, $CDCl_3$)

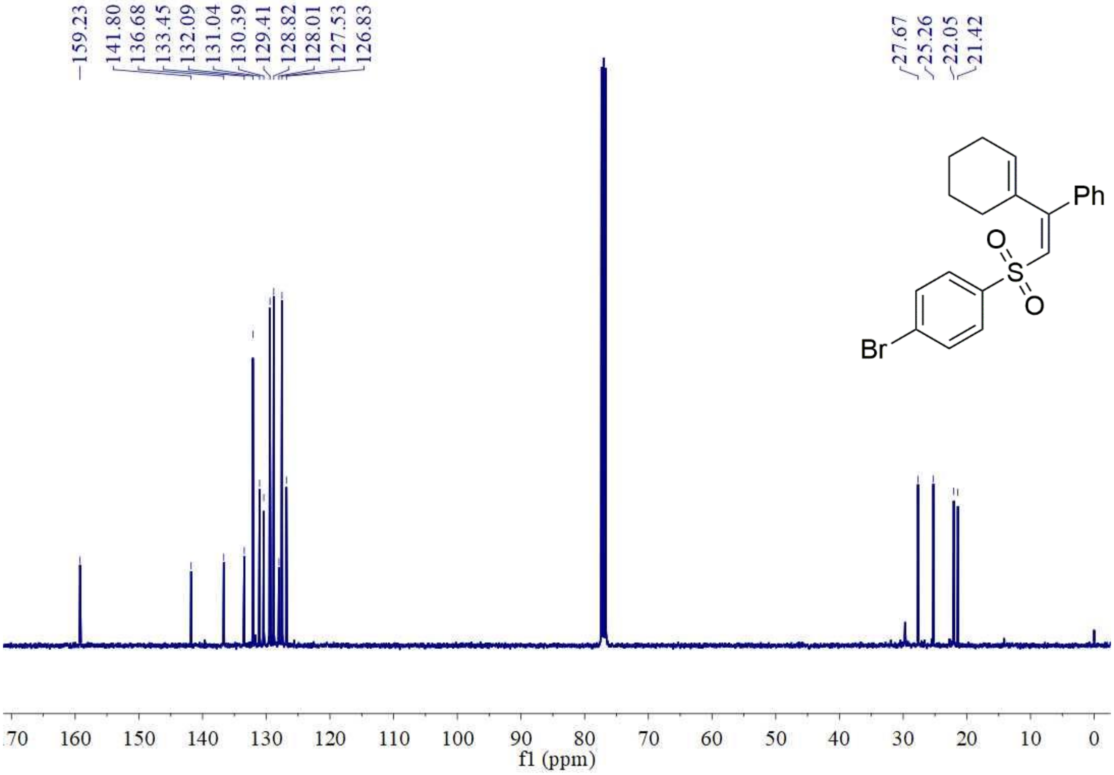

Supplementary Figure 221: ^{13}C NMR of 49a (100 MHz, $CDCl_3$)

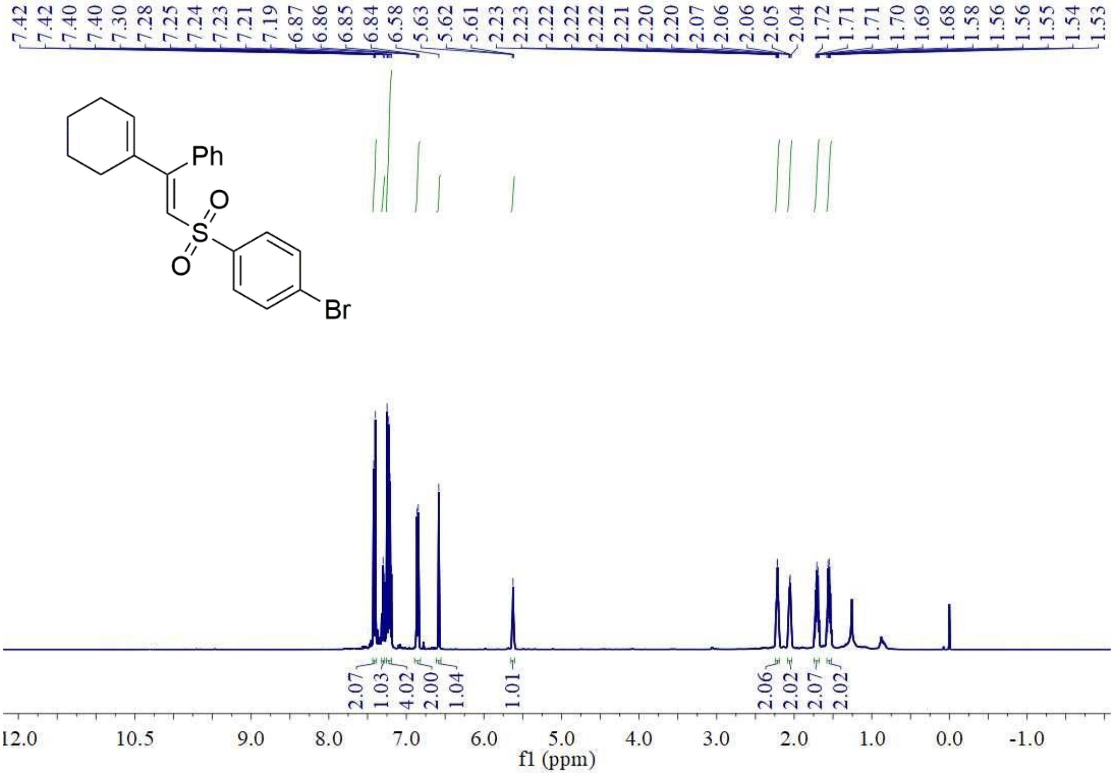

Supplementary Figure 222: ^{1}H NMR of 49b (400 MHz, $CDCl_3$)

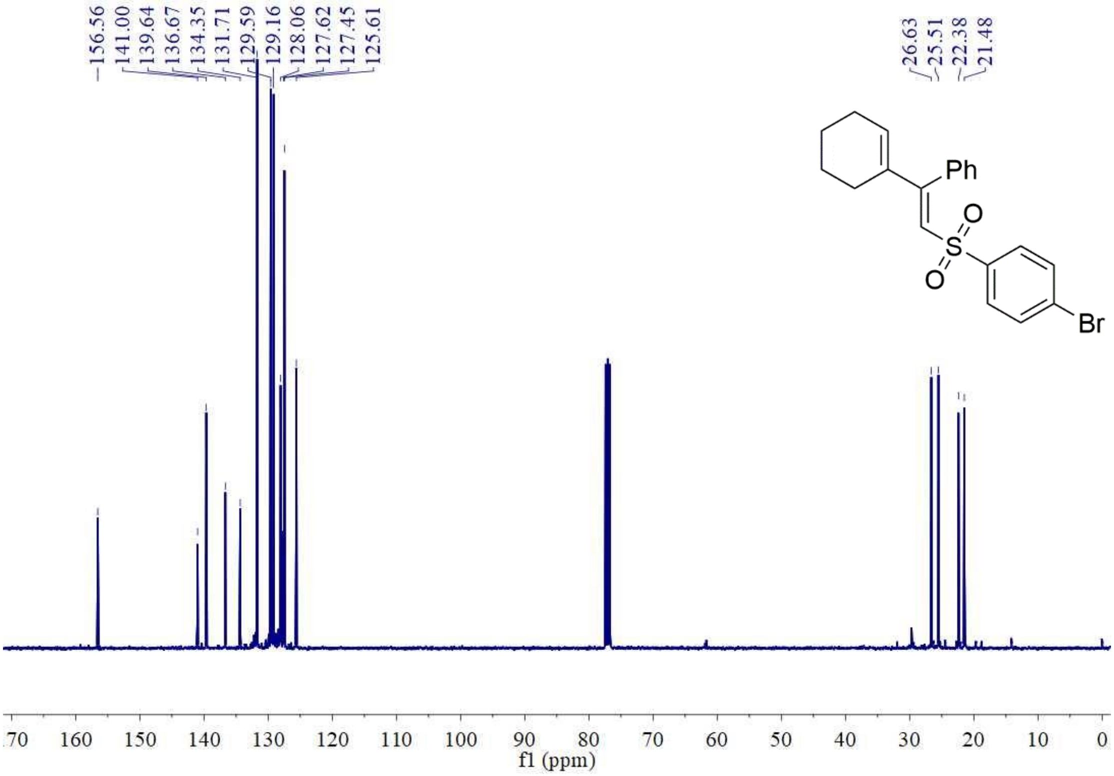

Supplementary Figure 223: ^{13}C NMR of 49b (100 MHz, $CDCl_3$)

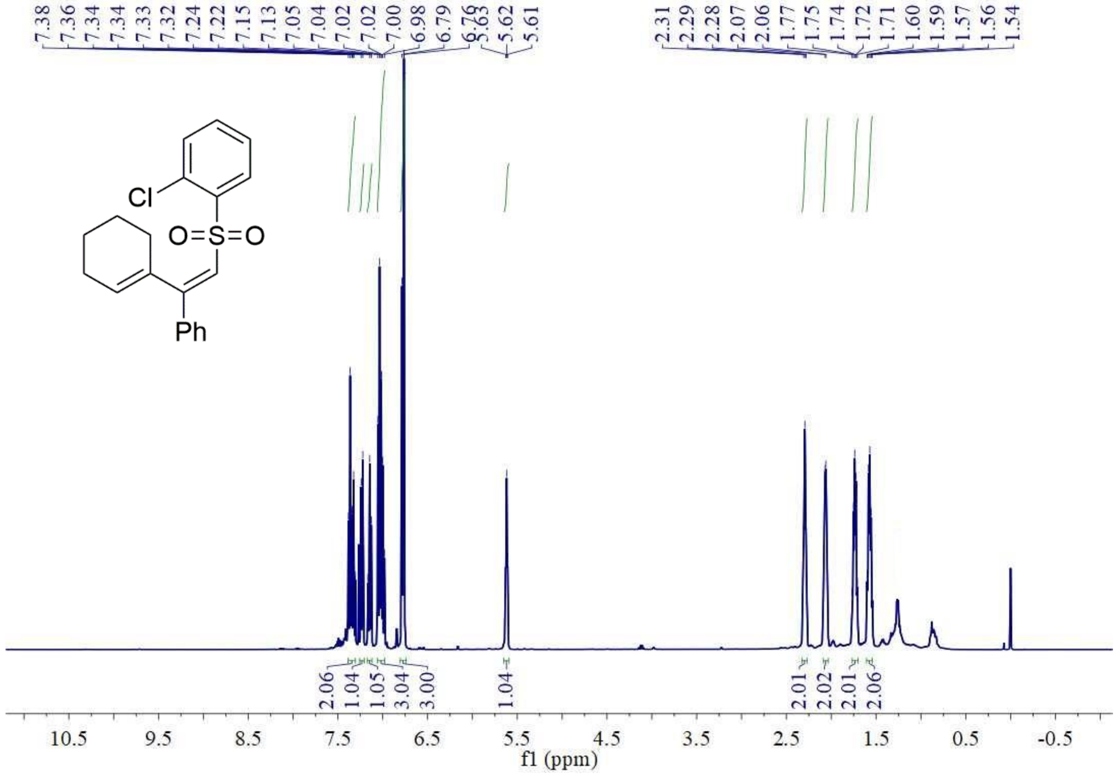

Supplementary Figure 224: ^{1}H NMR of 50a (400 MHz, $CDCl_3$)

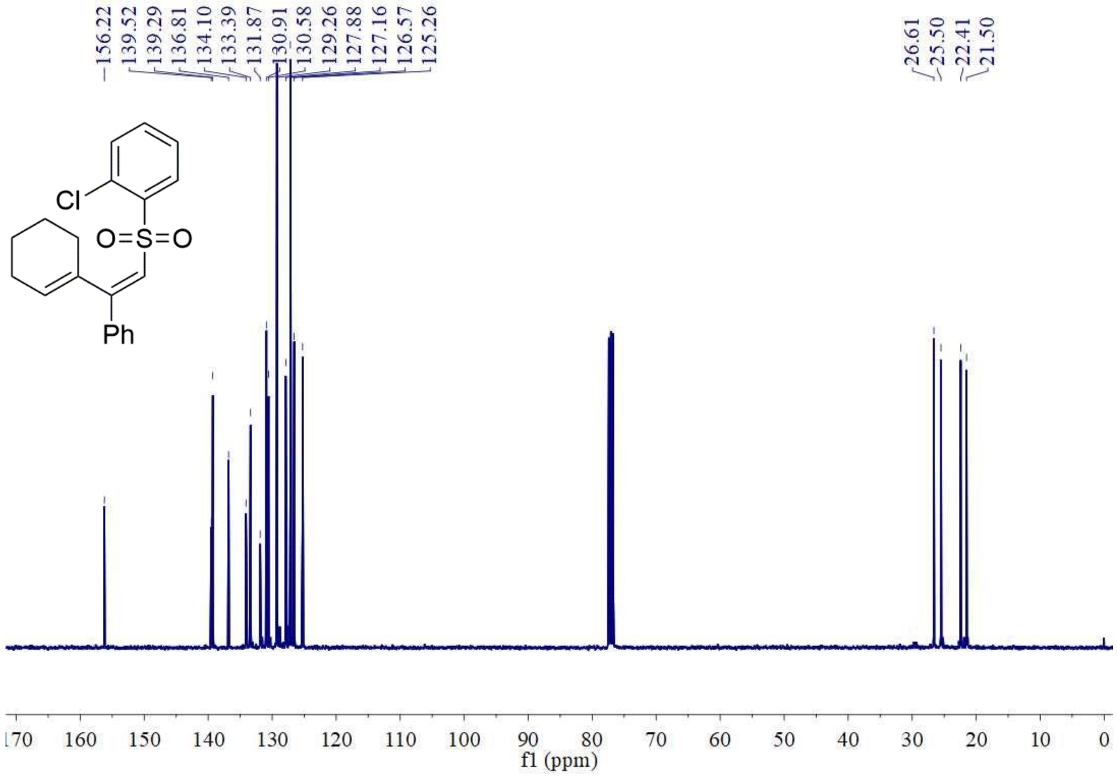

Supplementary Figure 225: ^{13}C NMR of 50a (100 MHz, $CDCl_3$)

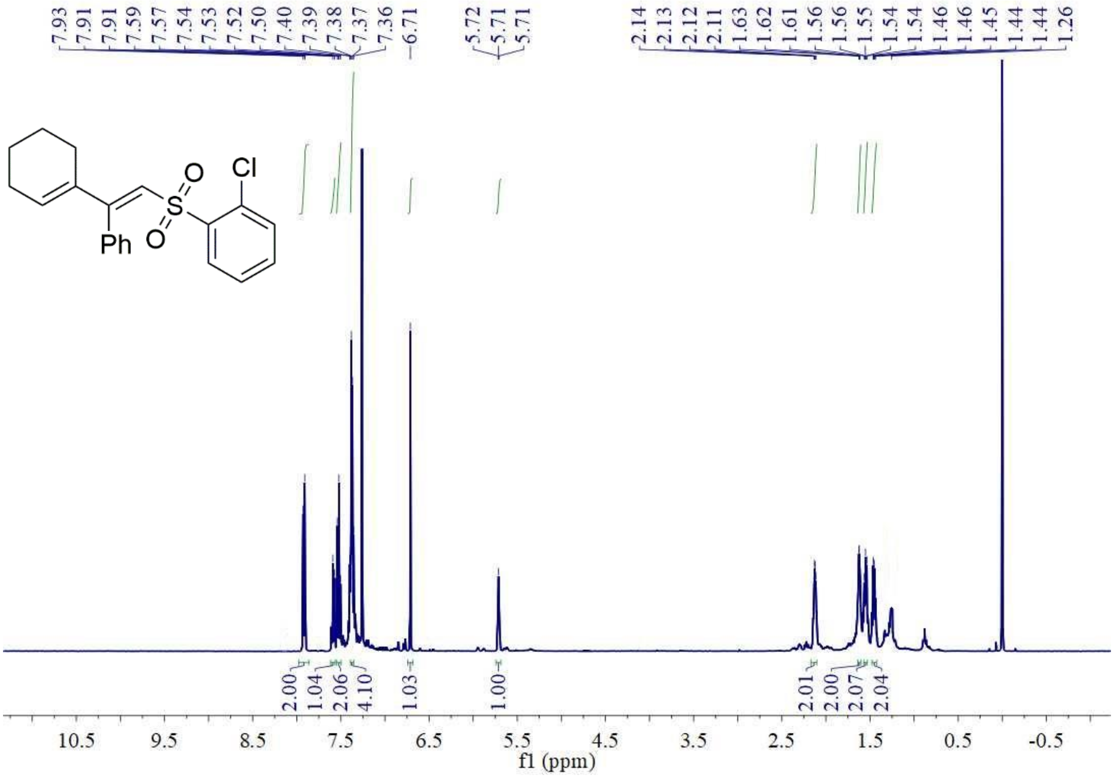

Supplementary Figure 226: ¹H NMR of 50b (400 MHz, $CDCl_3$)

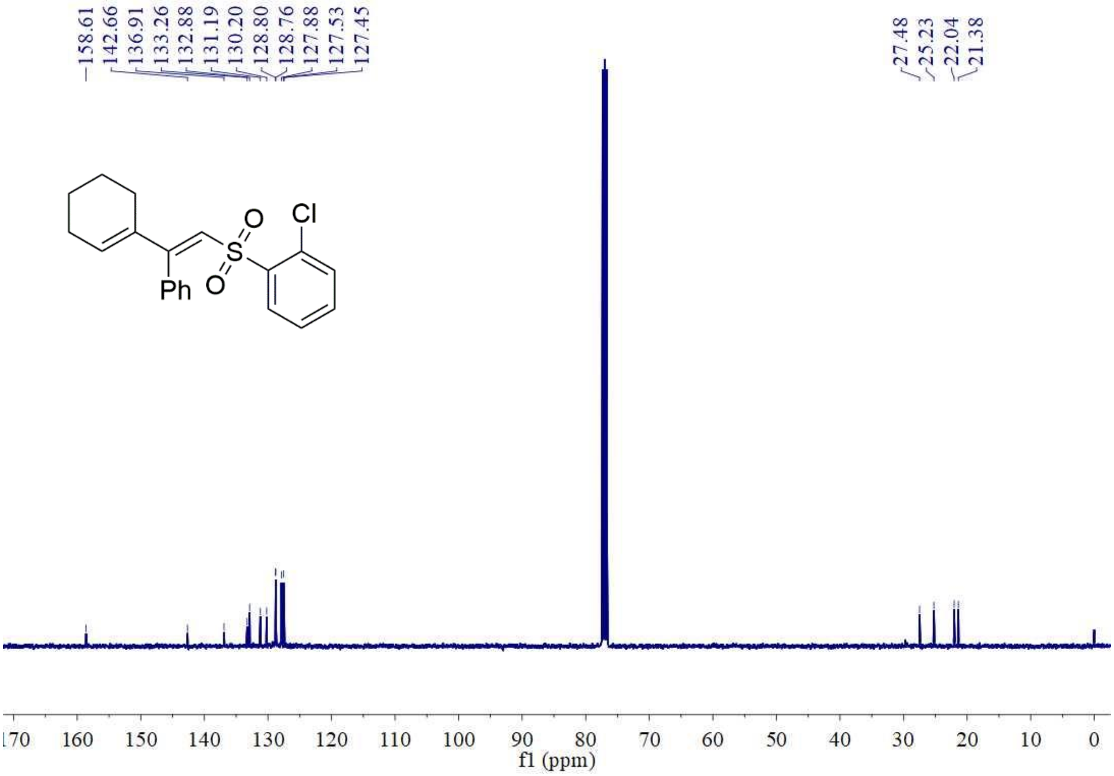

Supplementary Figure 227: ¹³C NMR of 50b (100 MHz, $CDCl_3$)

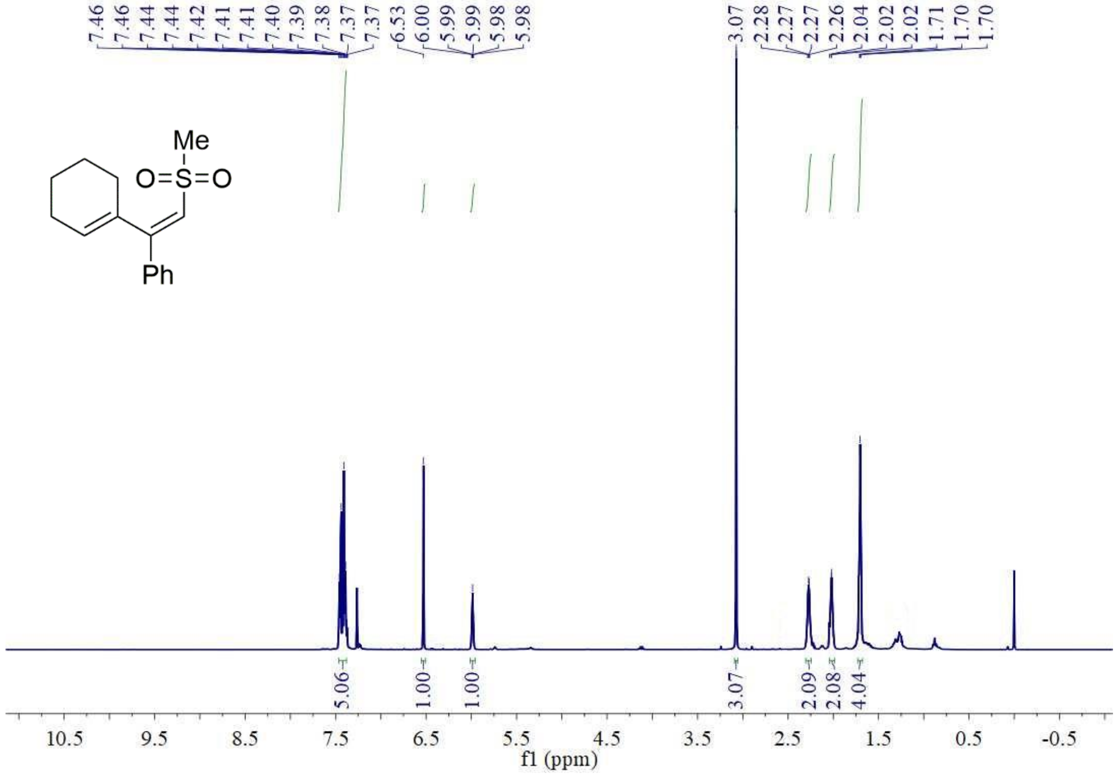

Supplementary Figure 228: ^{1}H NMR of 51a (400 MHz, $CDCl_3$)

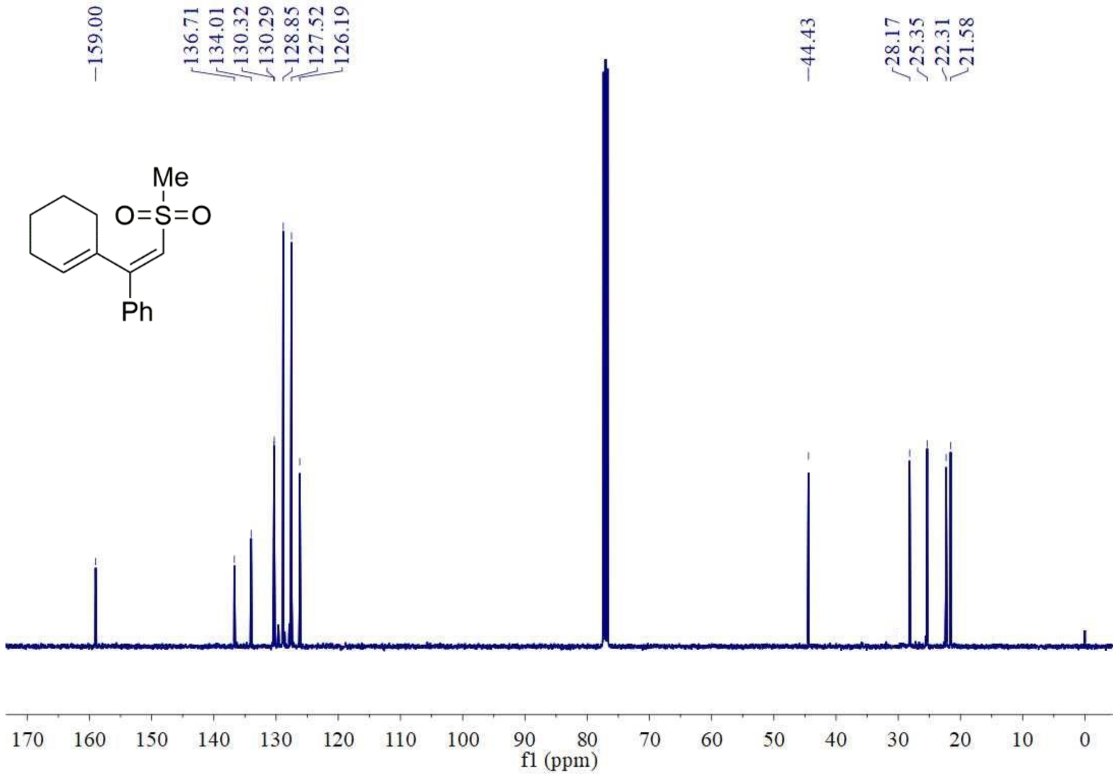

Supplementary Figure 229: ^{13}C NMR of 51a (100 MHz, $CDCl_3$)

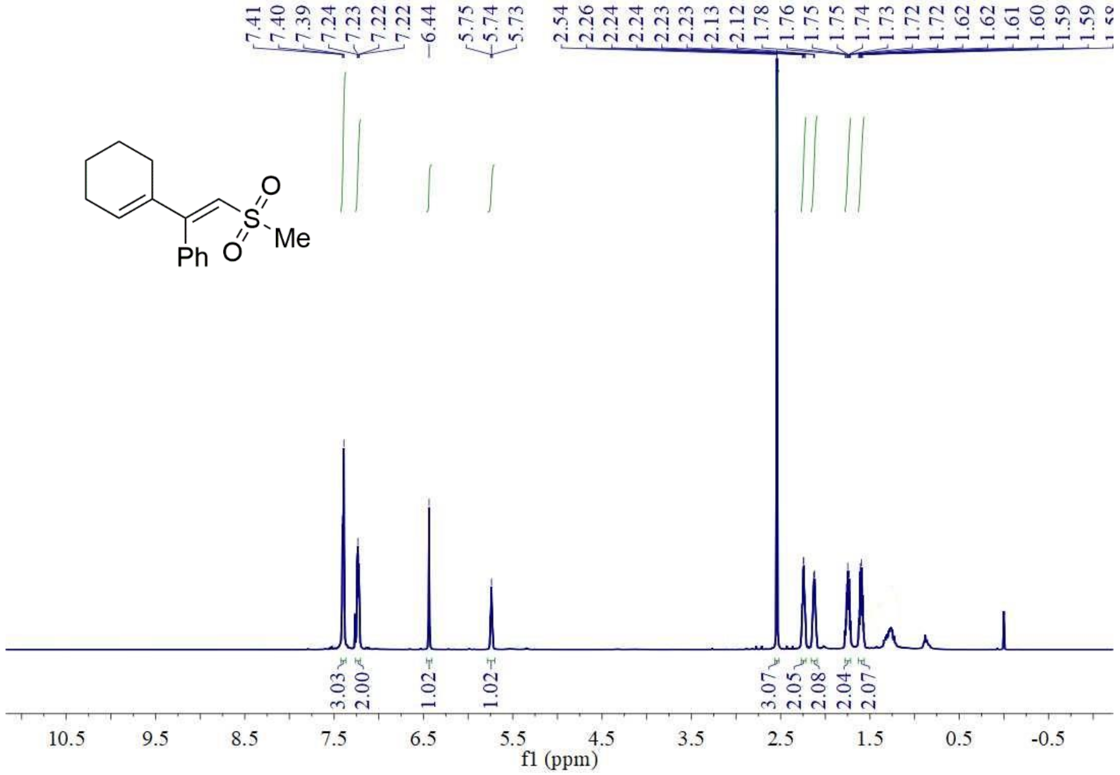

Supplementary Figure 230: ^{1}H NMR of 51b (400 MHz, $CDCl_3$)

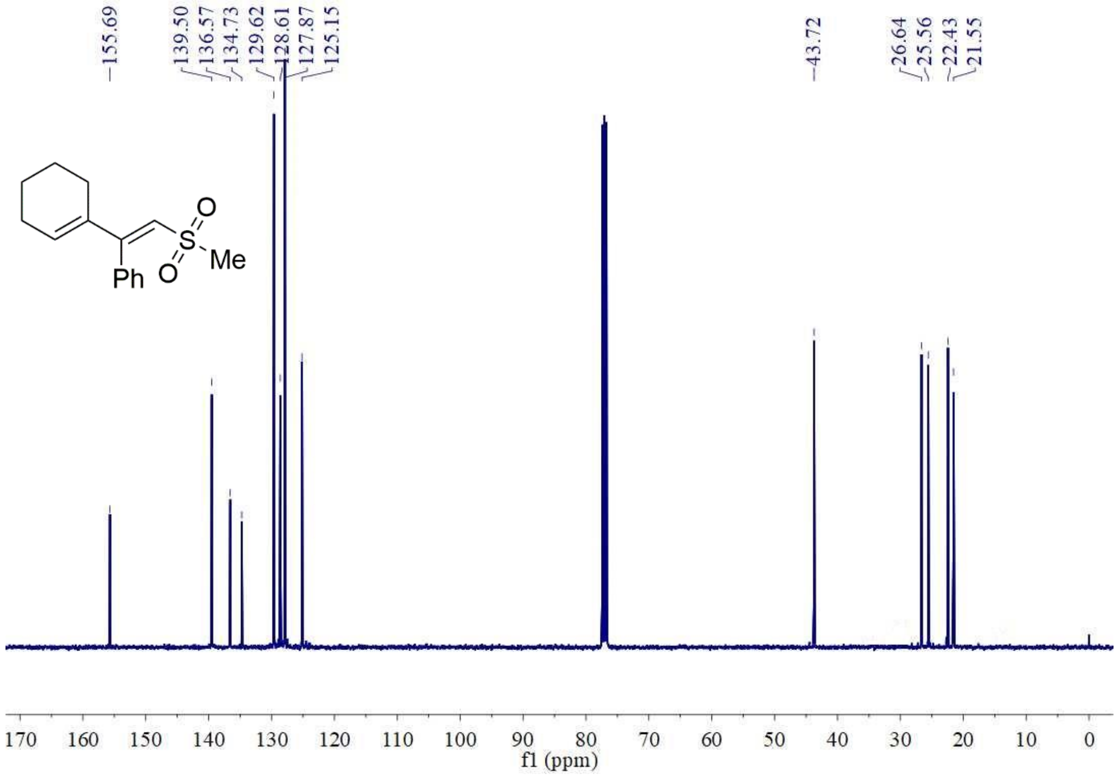

Supplementary Figure 231: ^{13}C NMR of 51b (100 MHz, $CDCl_3$)

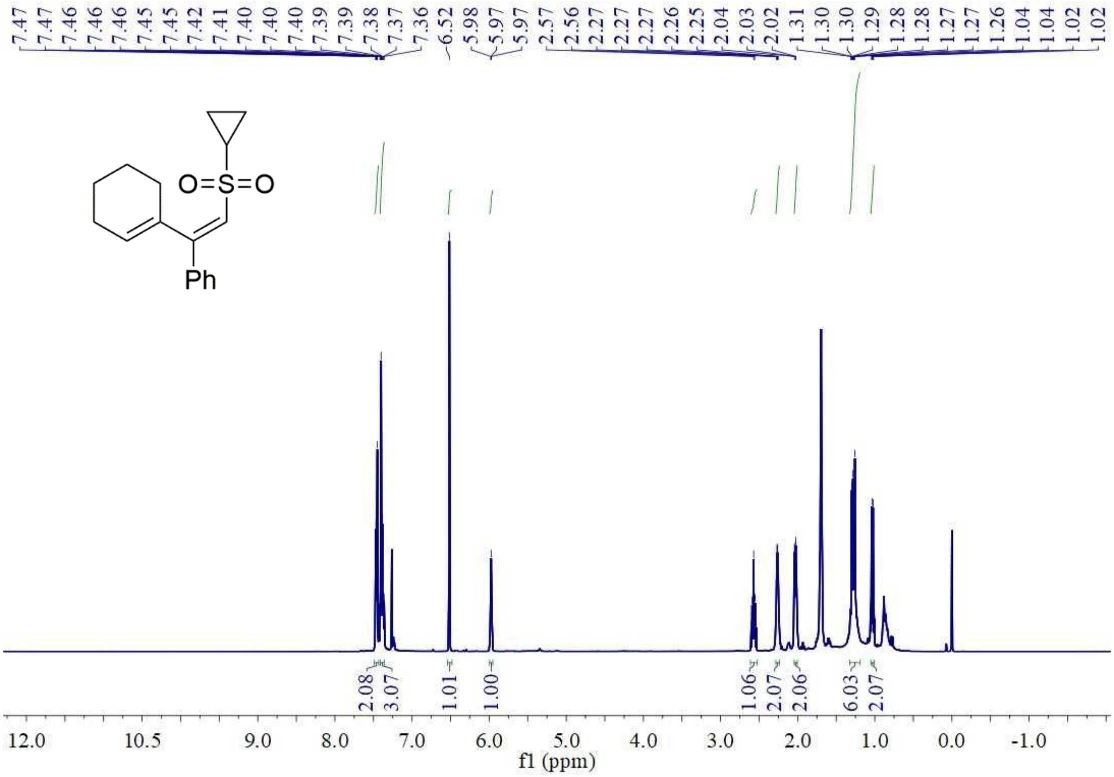

Supplementary Figure 232: ^{1}H NMR of 52a (400 MHz, $CDCl_3$)

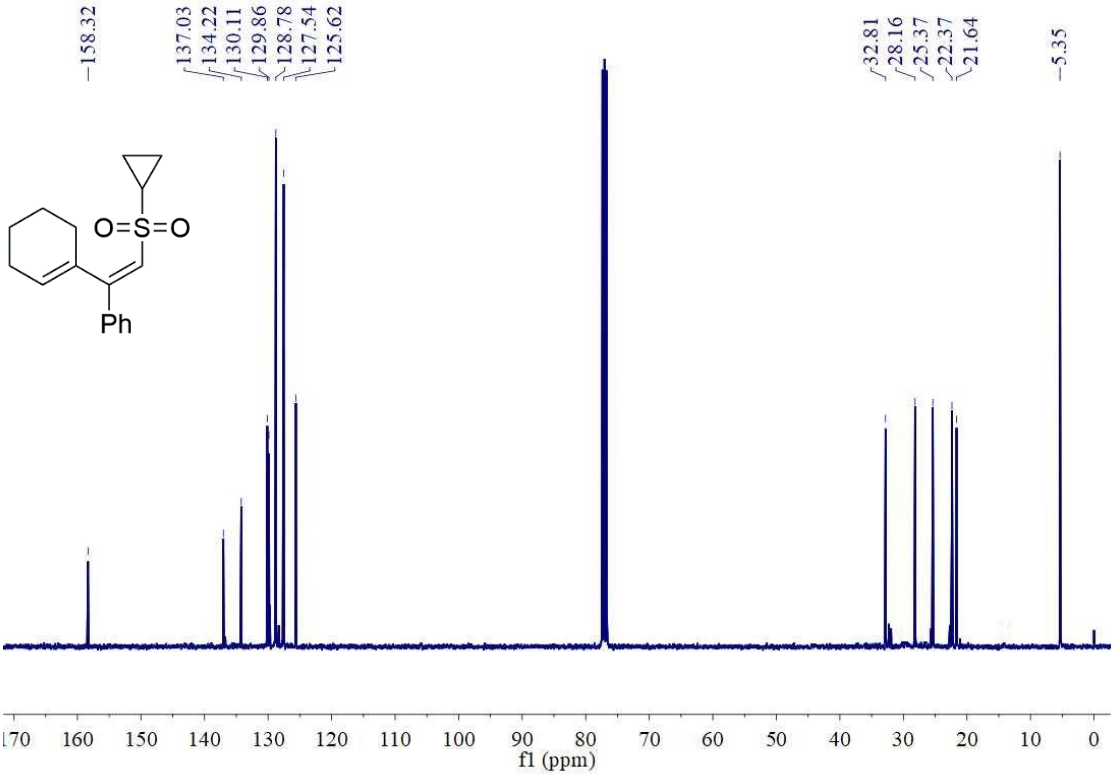

Supplementary Figure 233: ^{13}C NMR of 52a (100 MHz, $CDCl_3$)

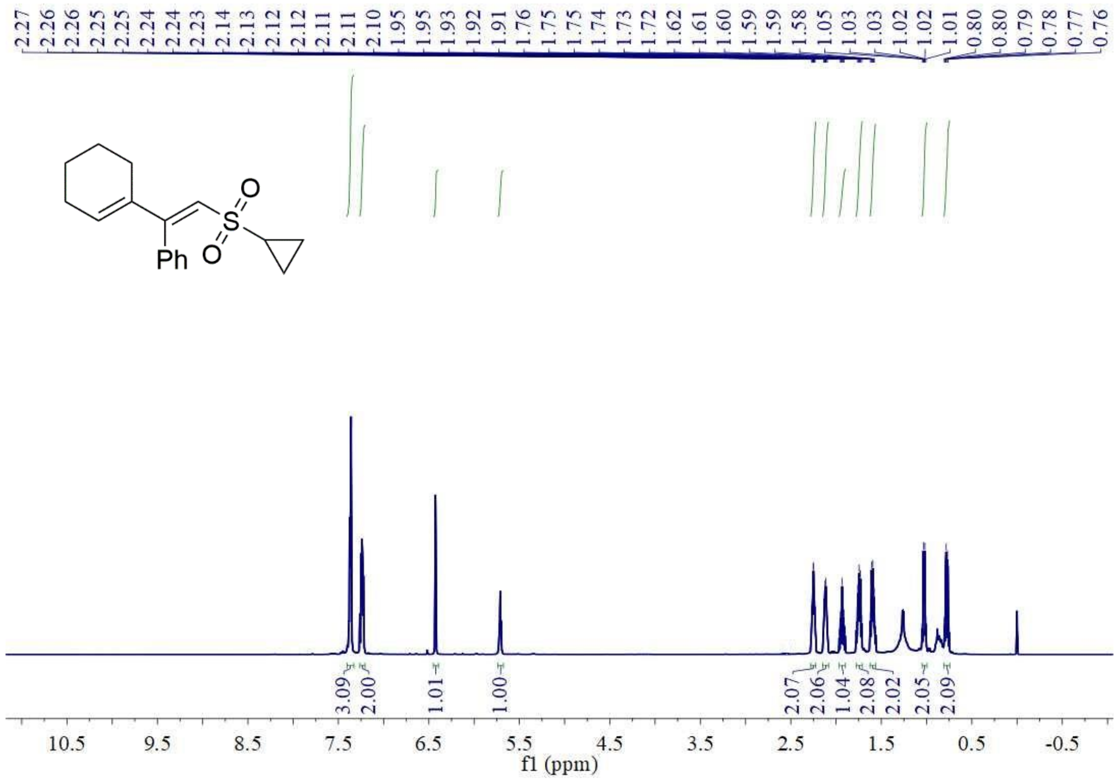

Supplementary Figure 234: ^{1}H NMR of 52b (400 MHz, $CDCl_3$)

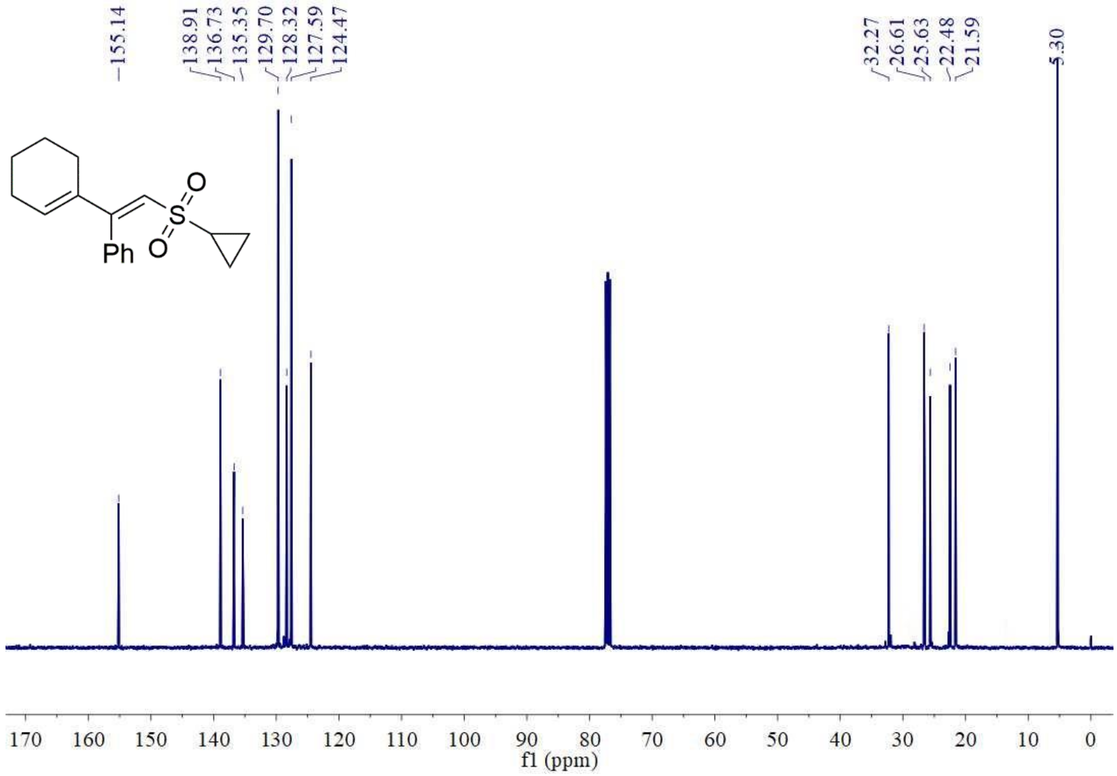

Supplementary Figure 235: ^{13}C NMR of 52b (100 MHz, $CDCl_3$)

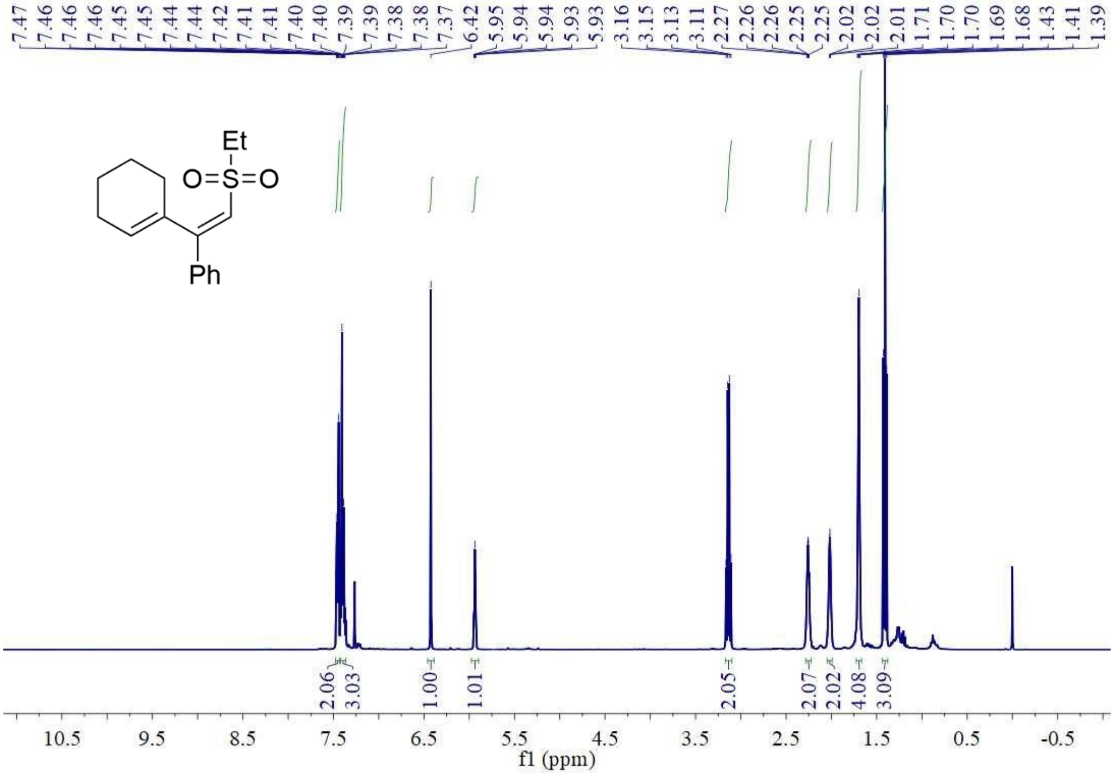

Supplementary Figure 236: ^{1}H NMR of 53a (400 MHz, $CDCl_3$)

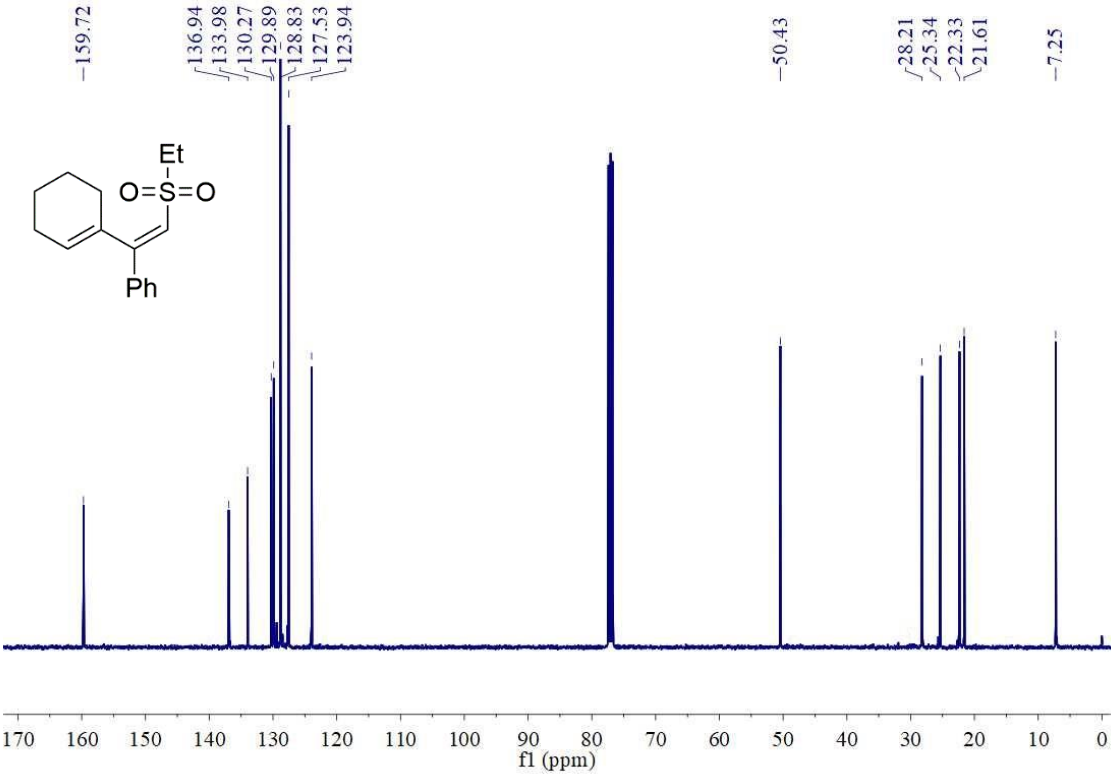

Supplementary Figure 237: ^{13}C NMR of 53a (100 MHz, $CDCl_3$)

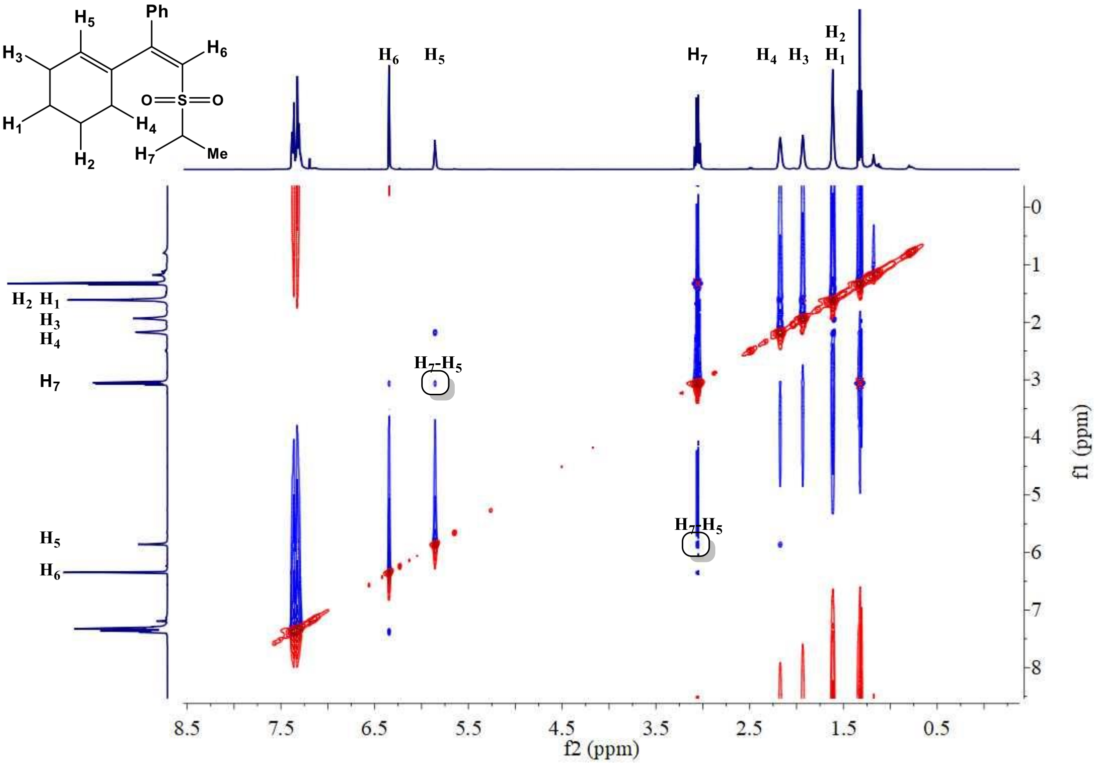

Supplementary Figure 238: NOESY of 53a

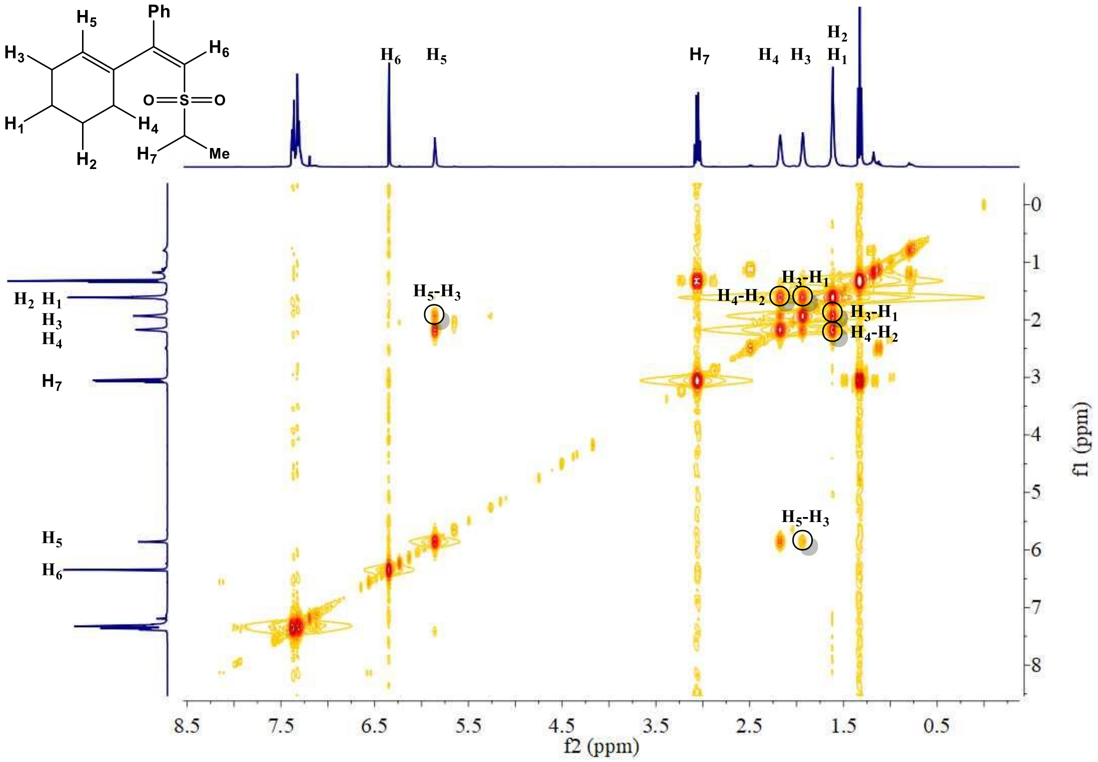

Supplementary Figure 239: COSY of 53a

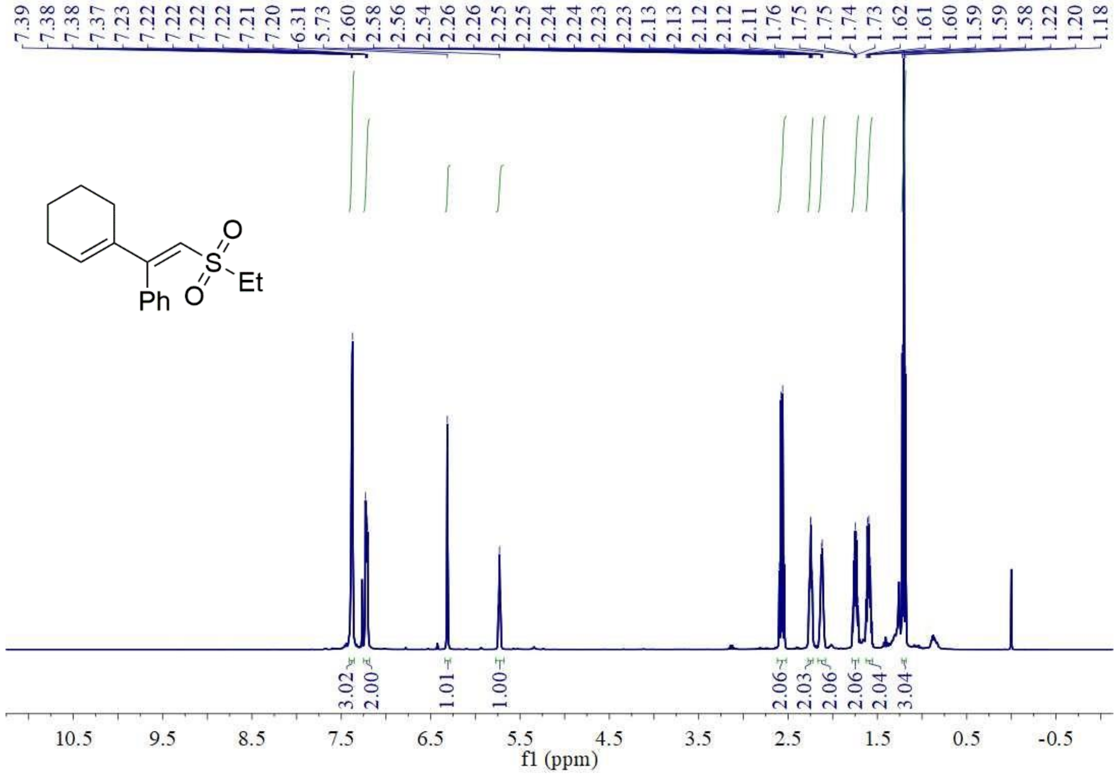

Supplementary Figure 240: ^{1}H NMR of 53b (400 MHz, $CDCl_3$)

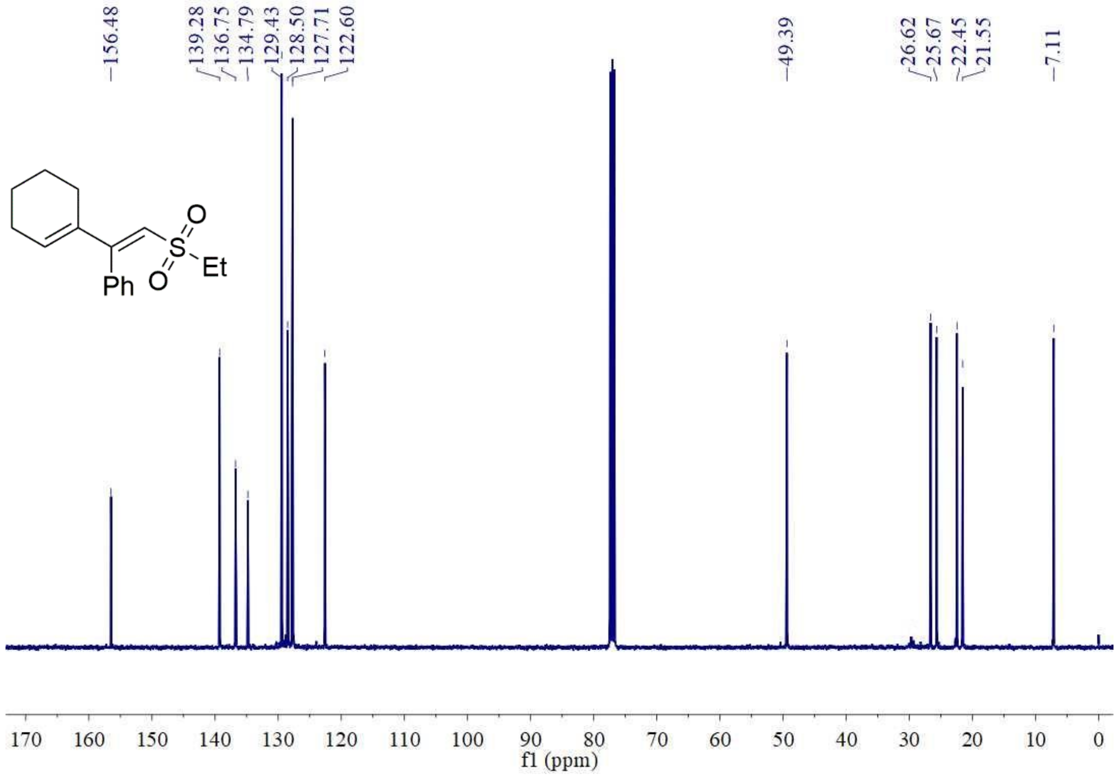

Supplementary Figure 241: ^{13}C NMR of 53b (100 MHz, $CDCl_3$)

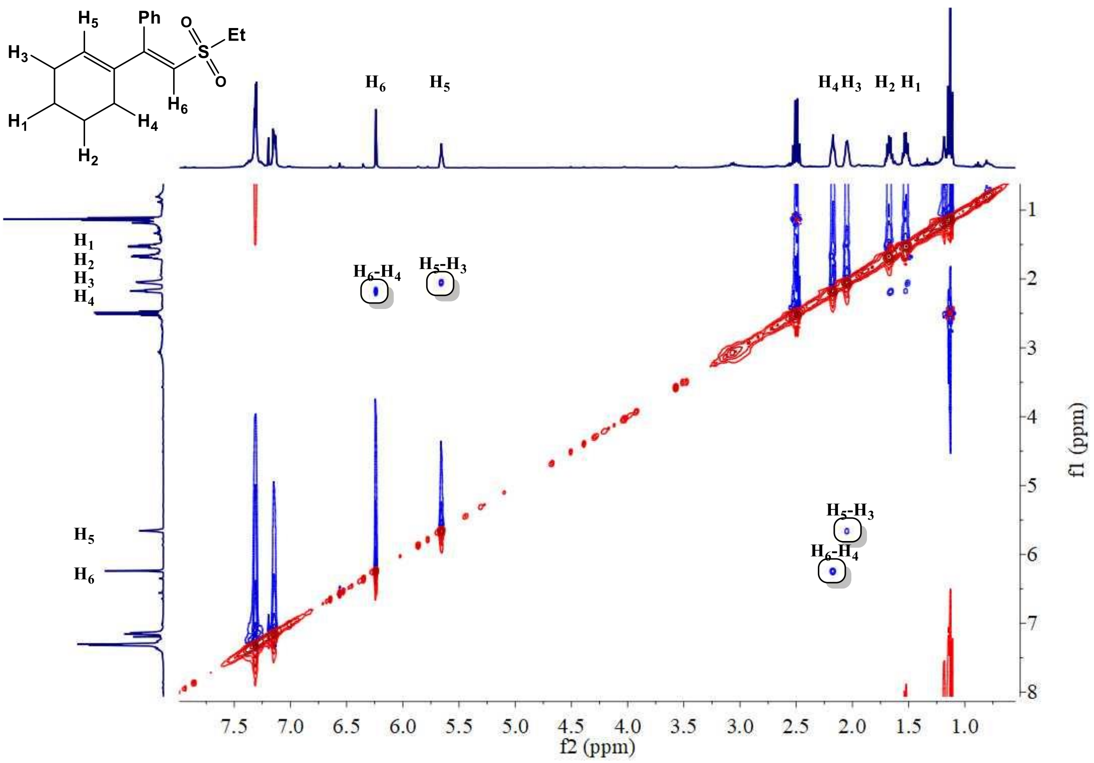

Supplementary Figure 242: NOESY of 53b

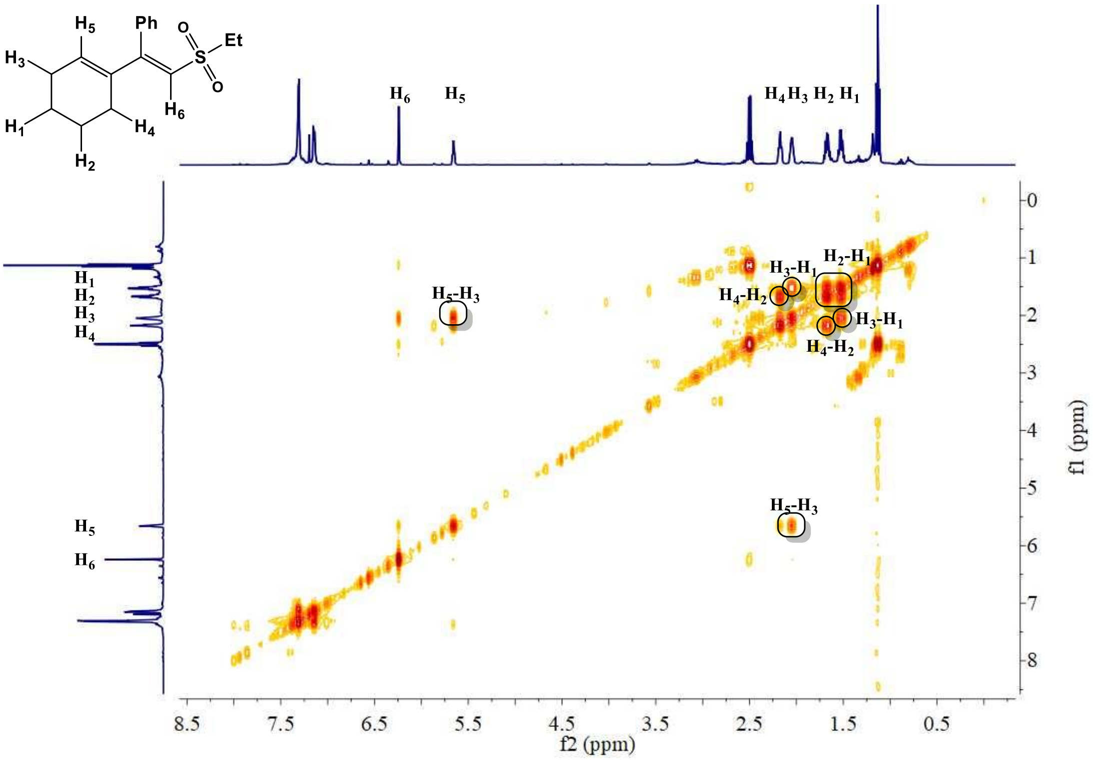

Supplementary Figure 243: COSY of 53b

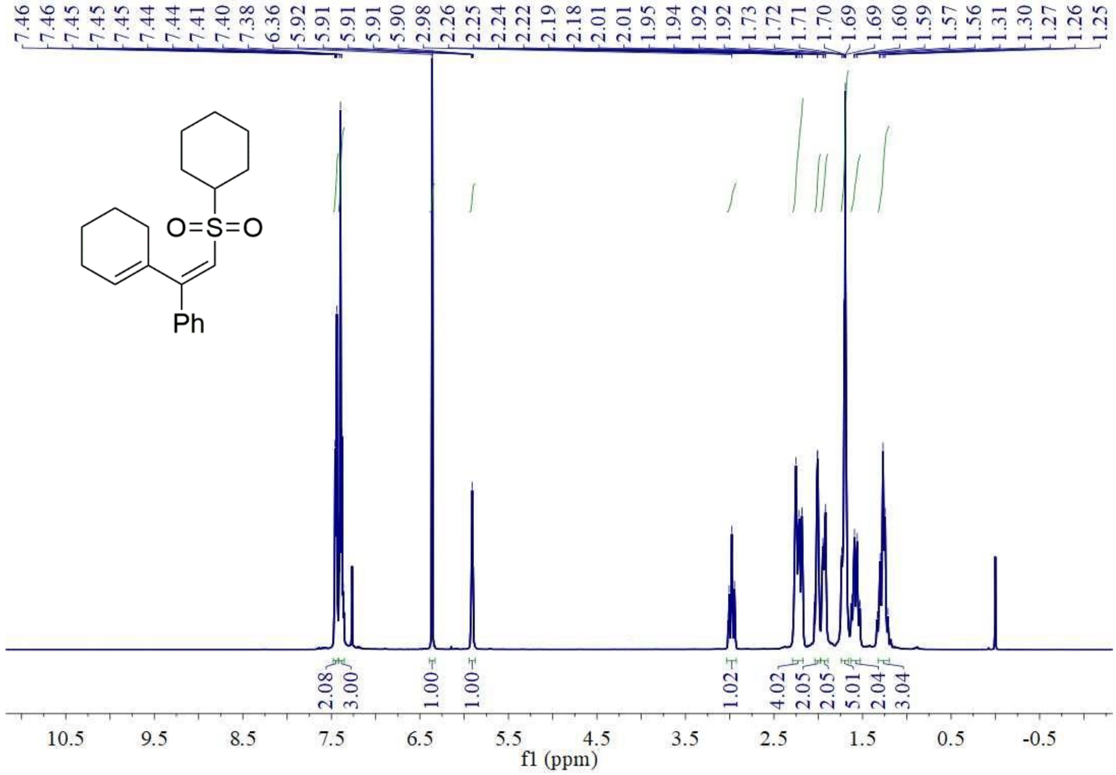

Supplementary Figure 244: ^{1}H NMR of 54a (400 MHz, $CDCl_3$)

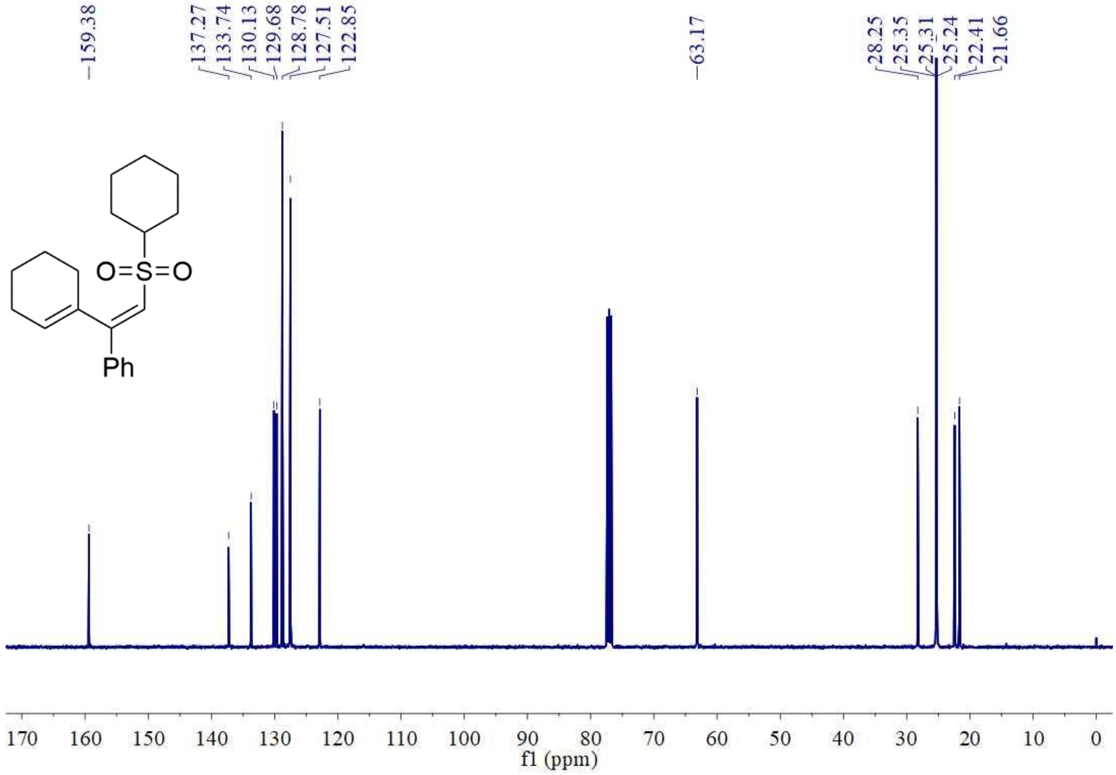

Supplementary Figure 245: ^{13}C NMR of 54a (100 MHz, $CDCl_3$)

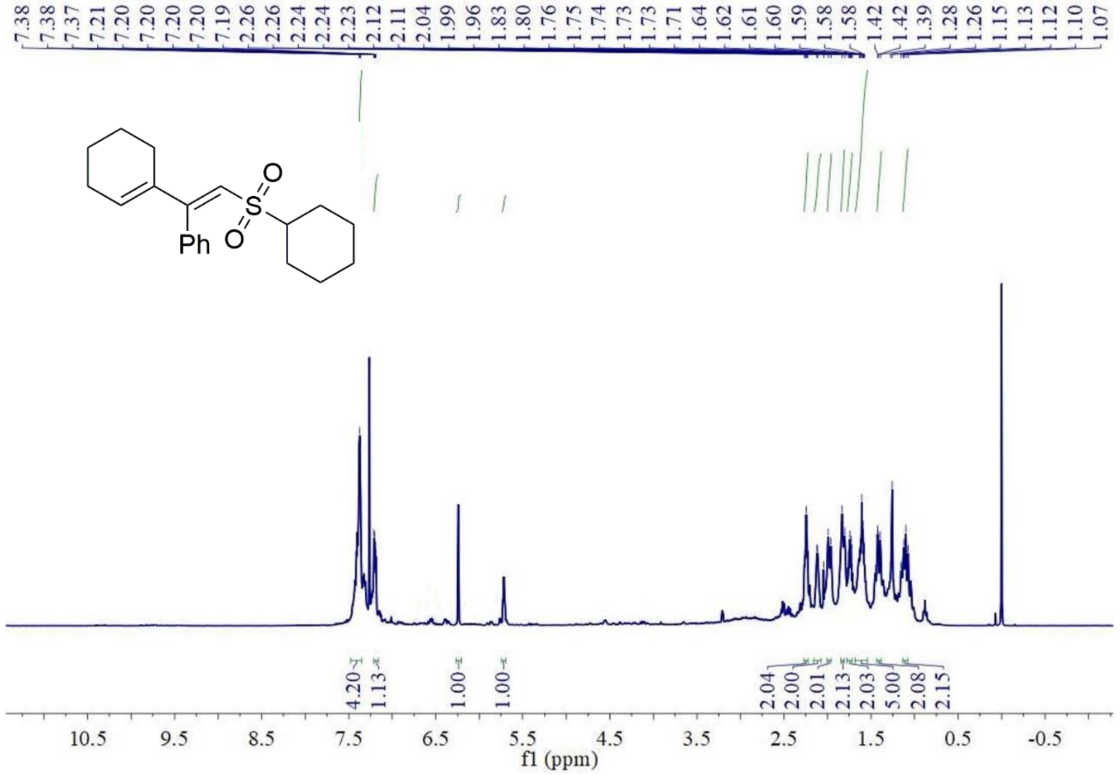

Supplementary Figure 246: ¹H NMR of 54b (400 MHz, $CDCl_3$)

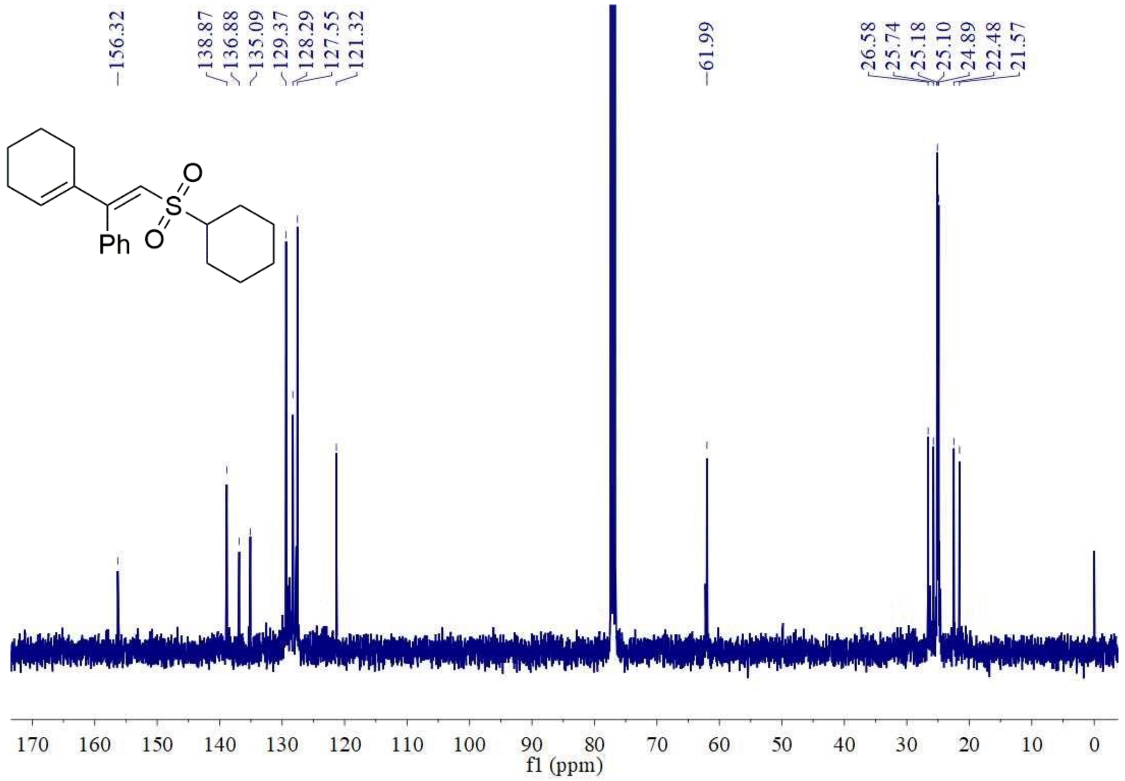

Supplementary Figure 247: ¹³C NMR of 54b (100 MHz, $CDCl_3$)

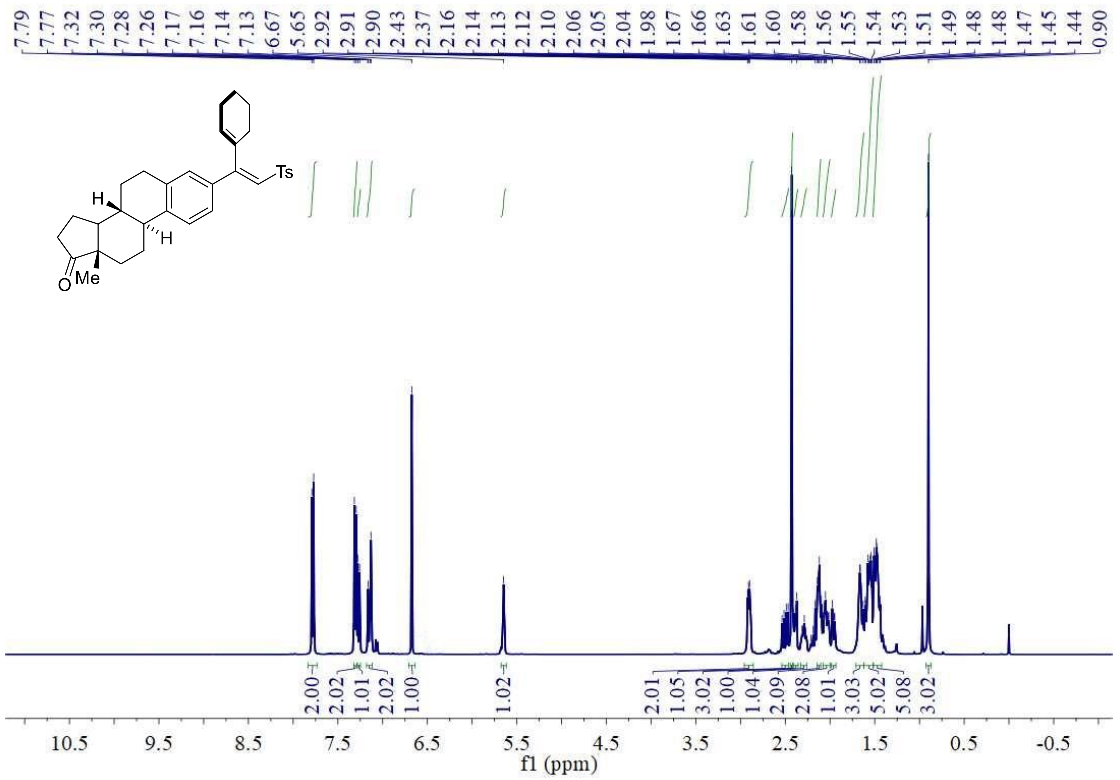

Supplementary Figure 248: ^{1}H NMR of 55a (400 MHz, $CDCl_3$)

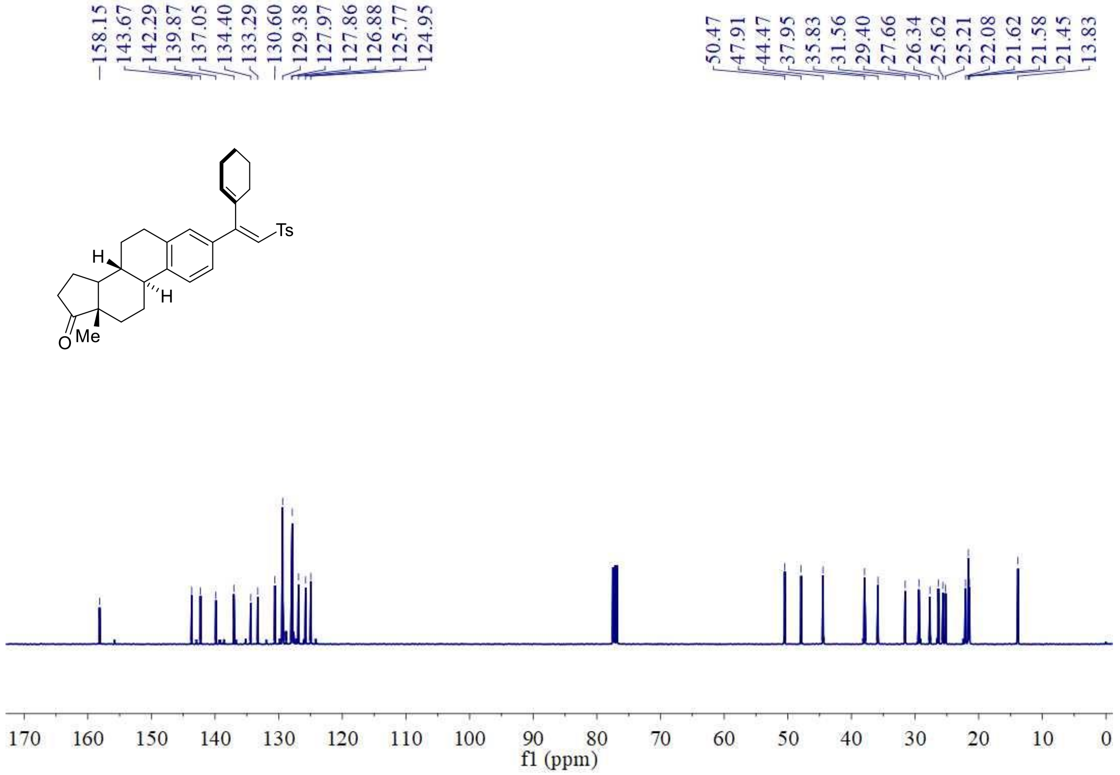

Supplementary Figure 249: ^{13}C NMR of 55a (100 MHz, $CDCl_3$)

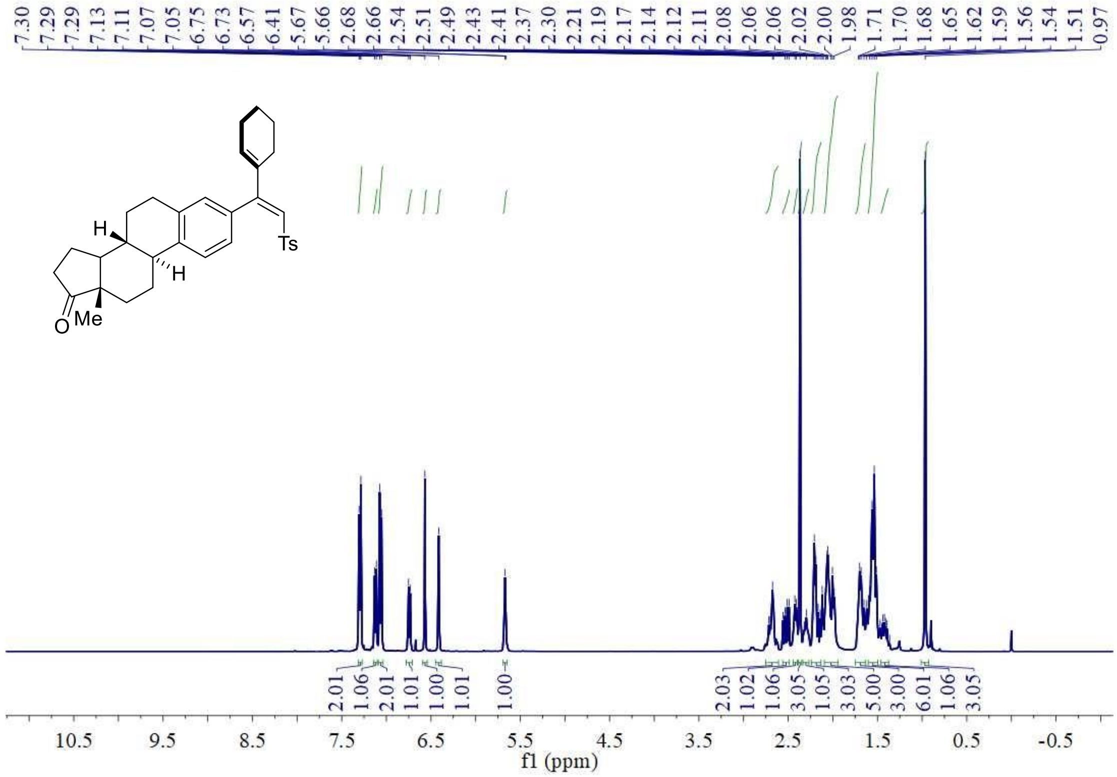

Supplementary Figure 250: ^{1}H NMR of 55b (400 MHz, $CDCl_3$)

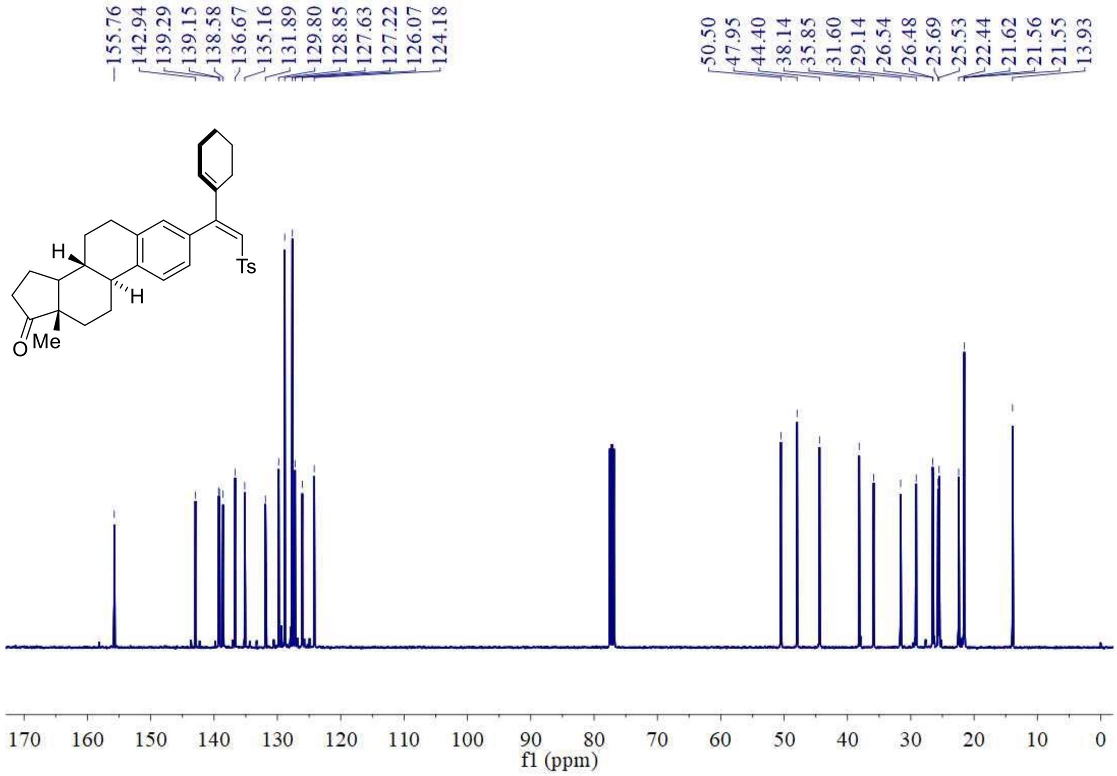

Supplementary Figure 251: ^{13}C NMR of 55b (100 MHz, $CDCl_3$)

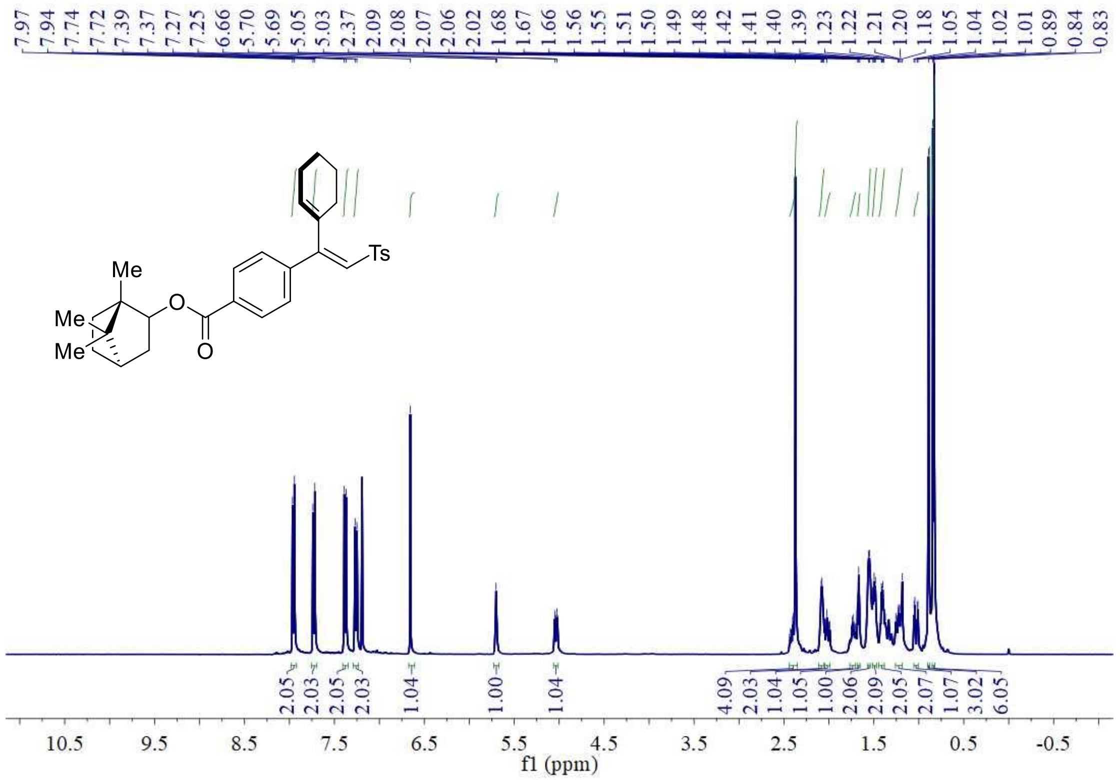

Supplementary Figure 252: ^{1}H NMR of 56a (400 MHz, $CDCl_3$)

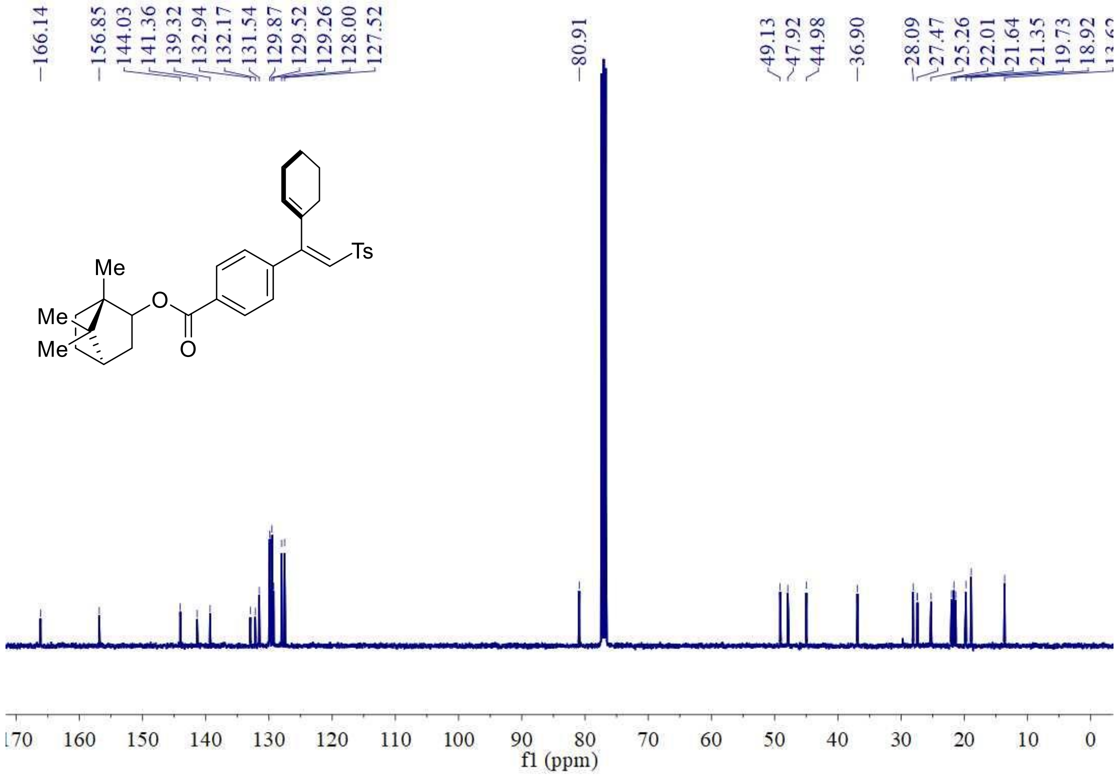

Supplementary Figure 253: ^{13}C NMR of 56a (100 MHz, $CDCl_3$)

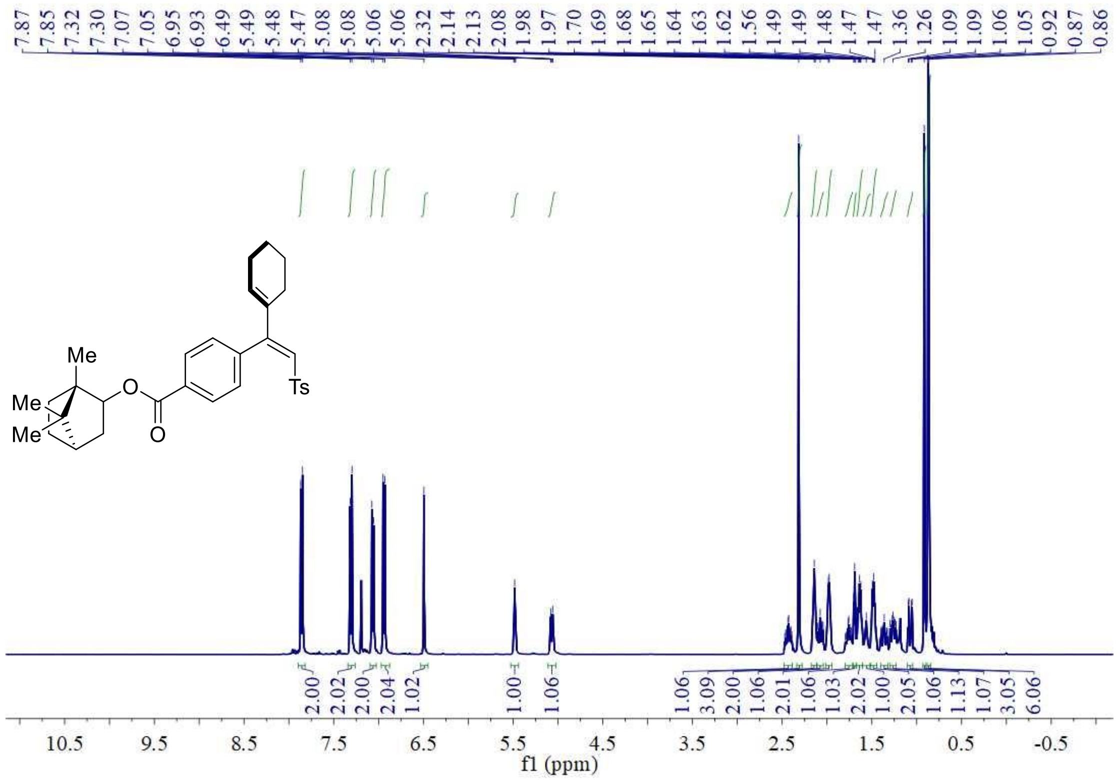

Supplementary Figure 254: ^{1}H NMR of 56b (400 MHz, $CDCl_3$)

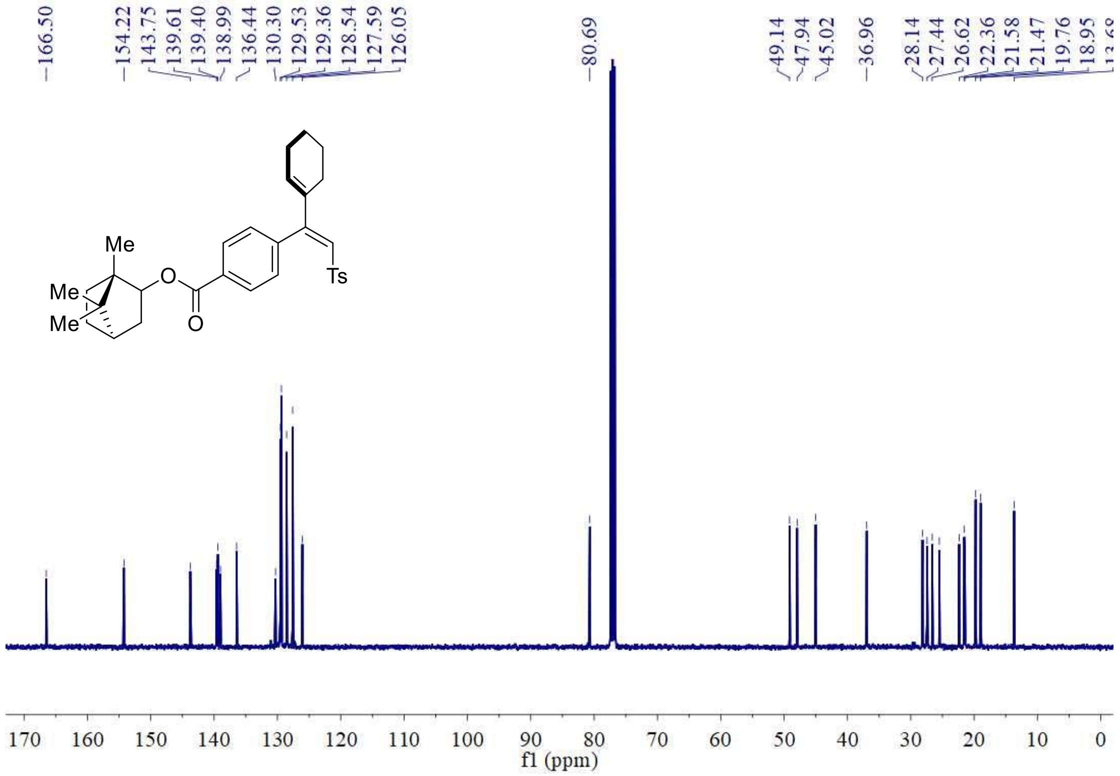

Supplementary Figure 255: ^{13}C NMR of 56b (100 MHz, $CDCl_3$)

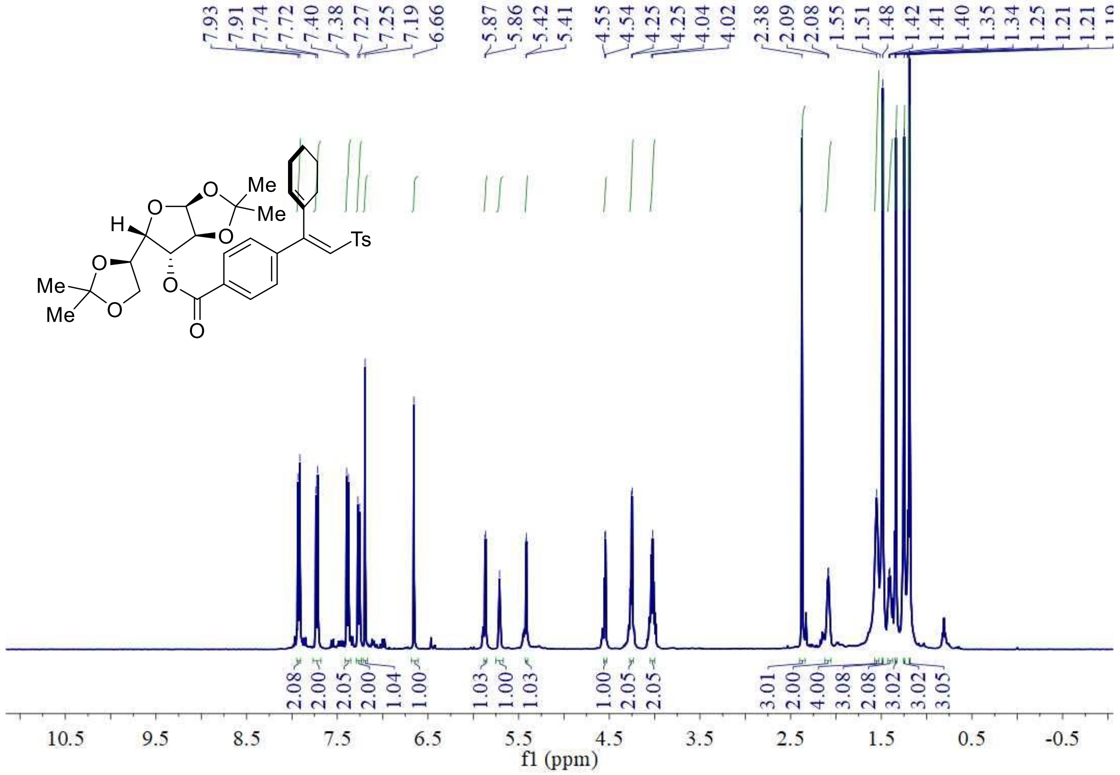

Supplementary Figure 256: ^{1}H NMR of 57a (400 MHz, $CDCl_3$)

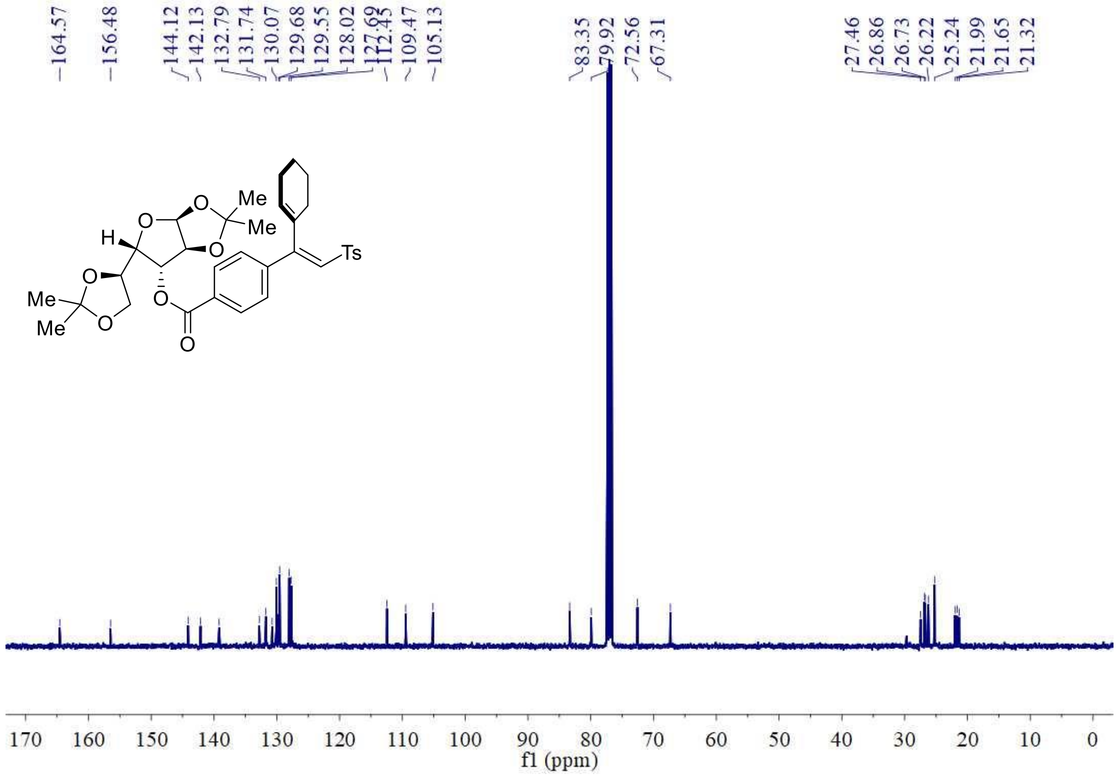

Supplementary Figure 257: ^{13}C NMR of 57a (100 MHz, $CDCl_3$)

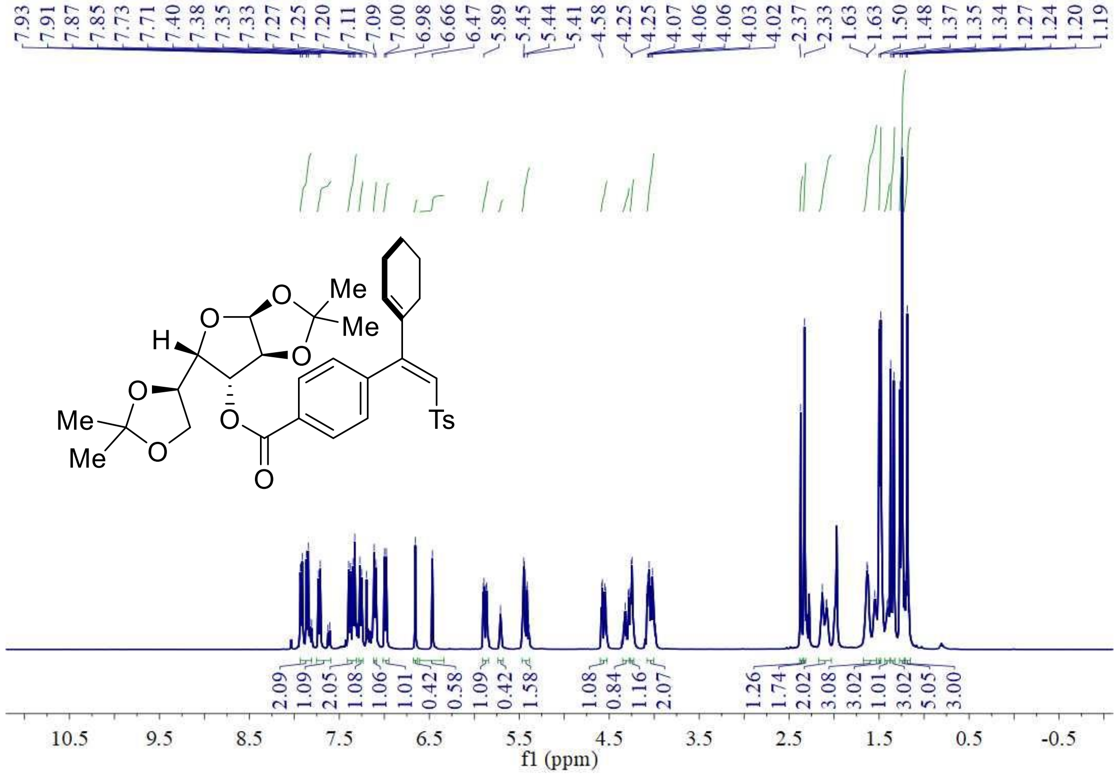

Supplementary Figure 258: ^{1}H NMR of 57b (400 MHz, $CDCl_3$)

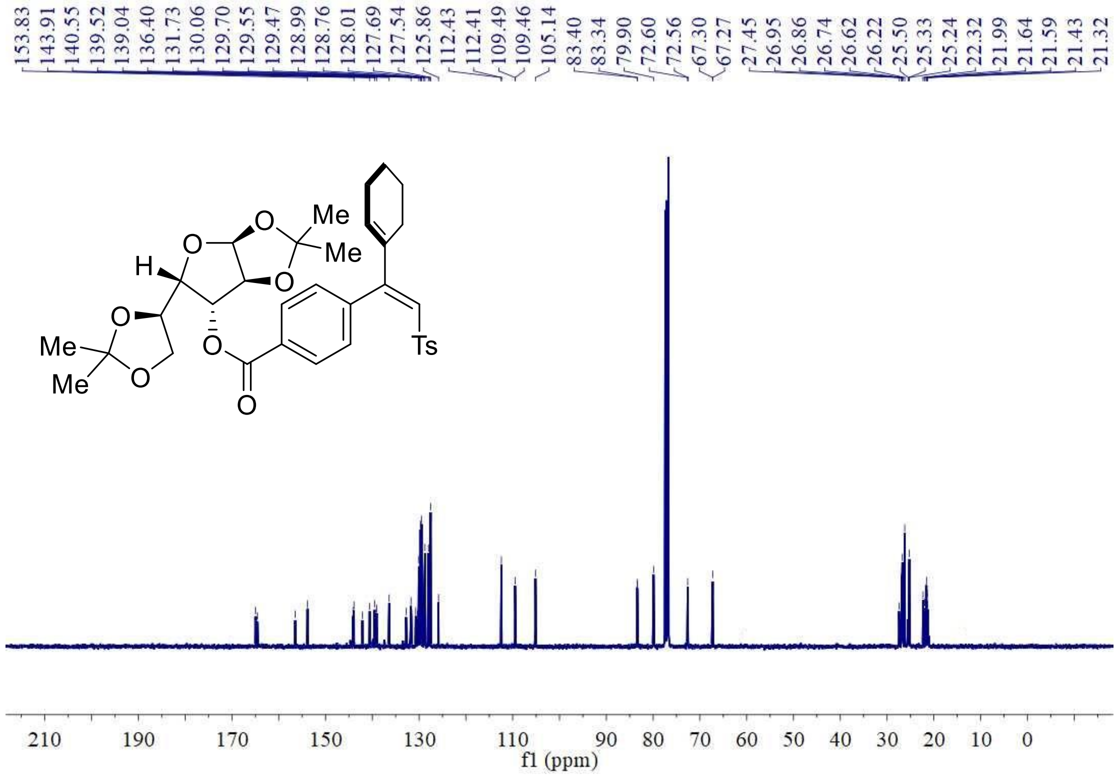

Supplementary Figure 259: ^{13}C NMR of 57b (100 MHz, $CDCl_3$)

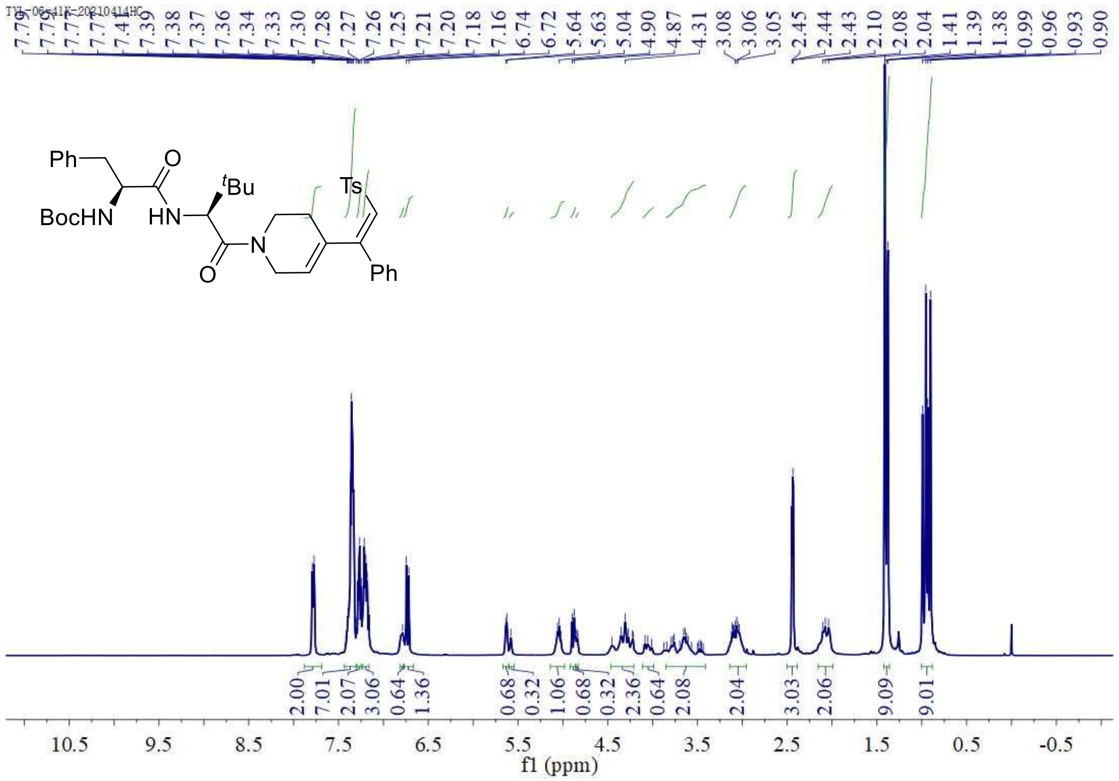

Supplementary Figure 260: ^{1}H NMR of 58a (400 MHz, $CDCl_3$)

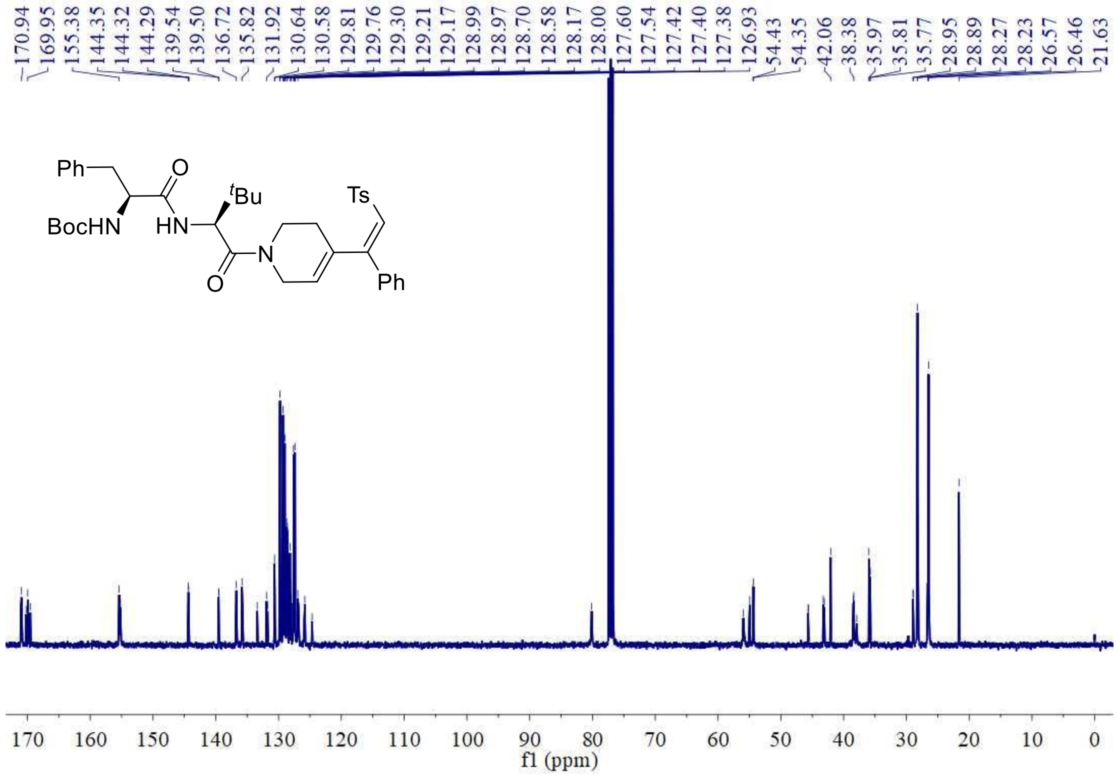

Supplementary Figure 261: ^{13}C NMR of 58a (100 MHz, $CDCl_3$)

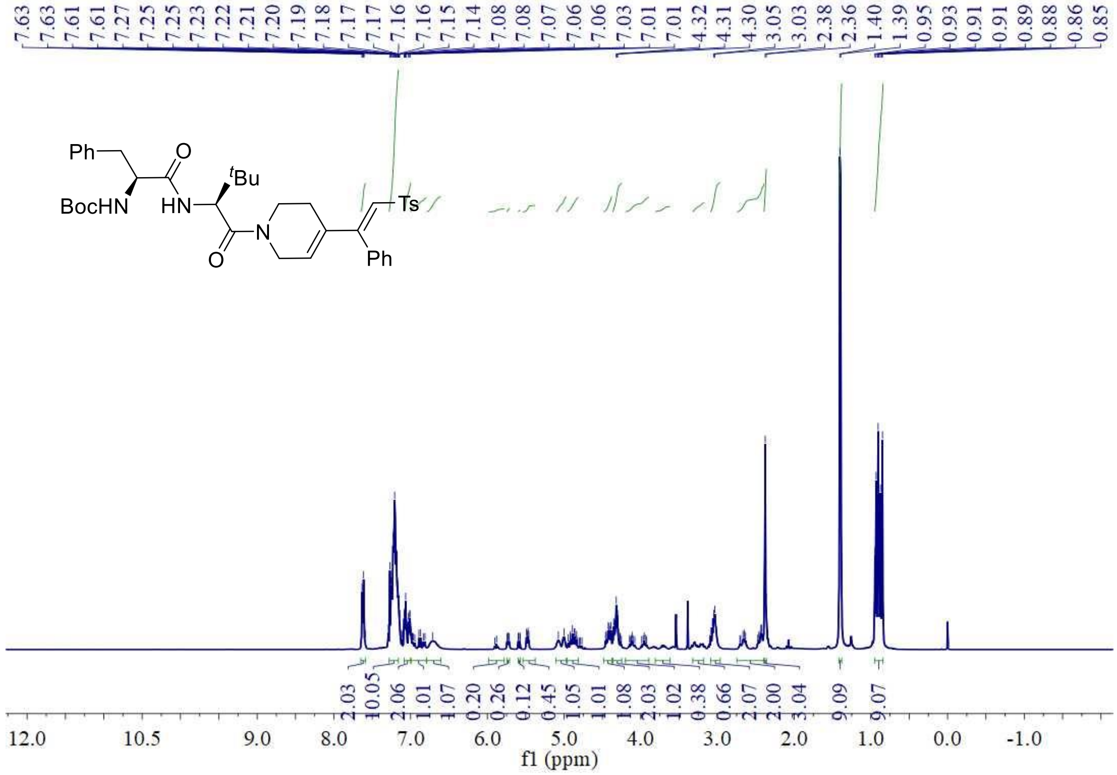

Supplementary Figure 262: ^{1}H NMR of 58b (400 MHz, $CDCl_3$)

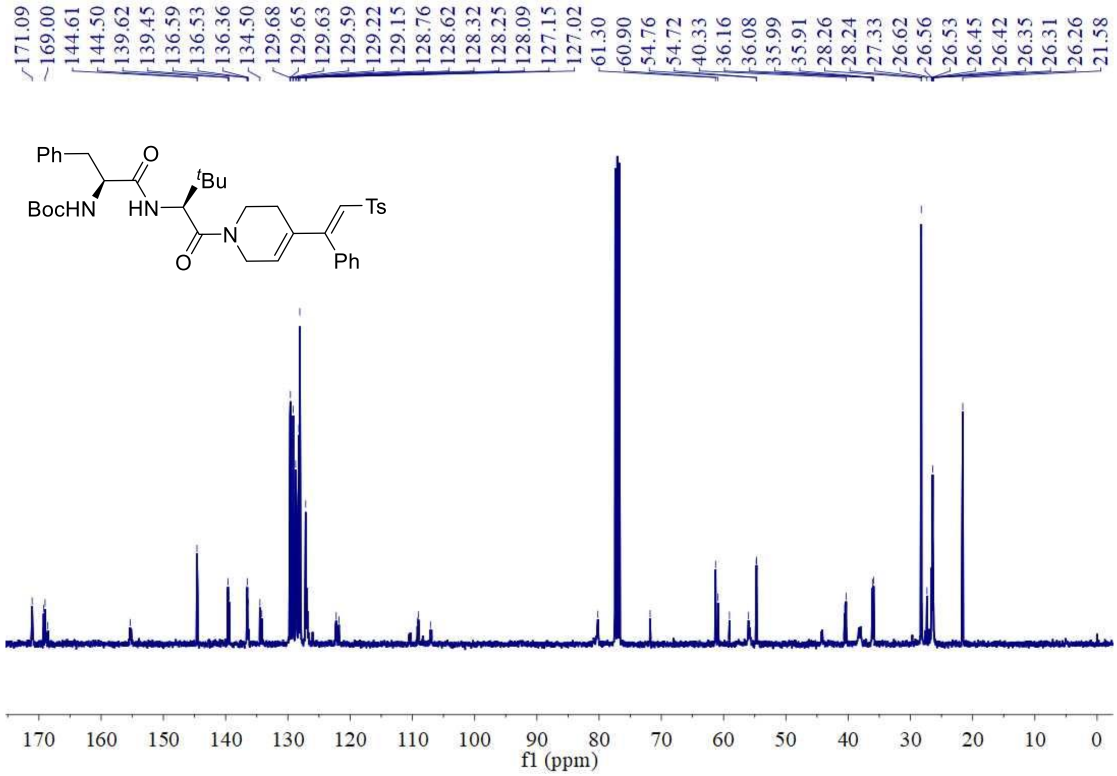

Supplementary Figure 263: ^{13}C NMR of 58b (100 MHz, $CDCl_3$)

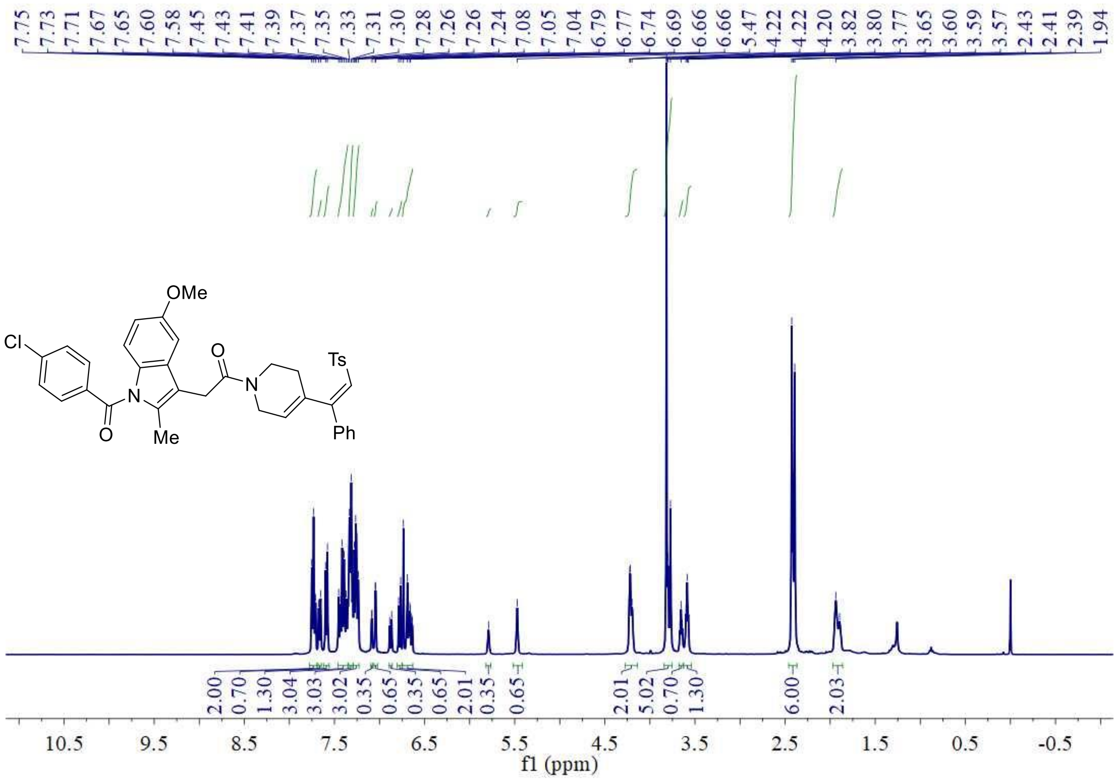

Supplementary Figure 264: ^{1}H NMR of 59a (400 MHz, $CDCl_3$)

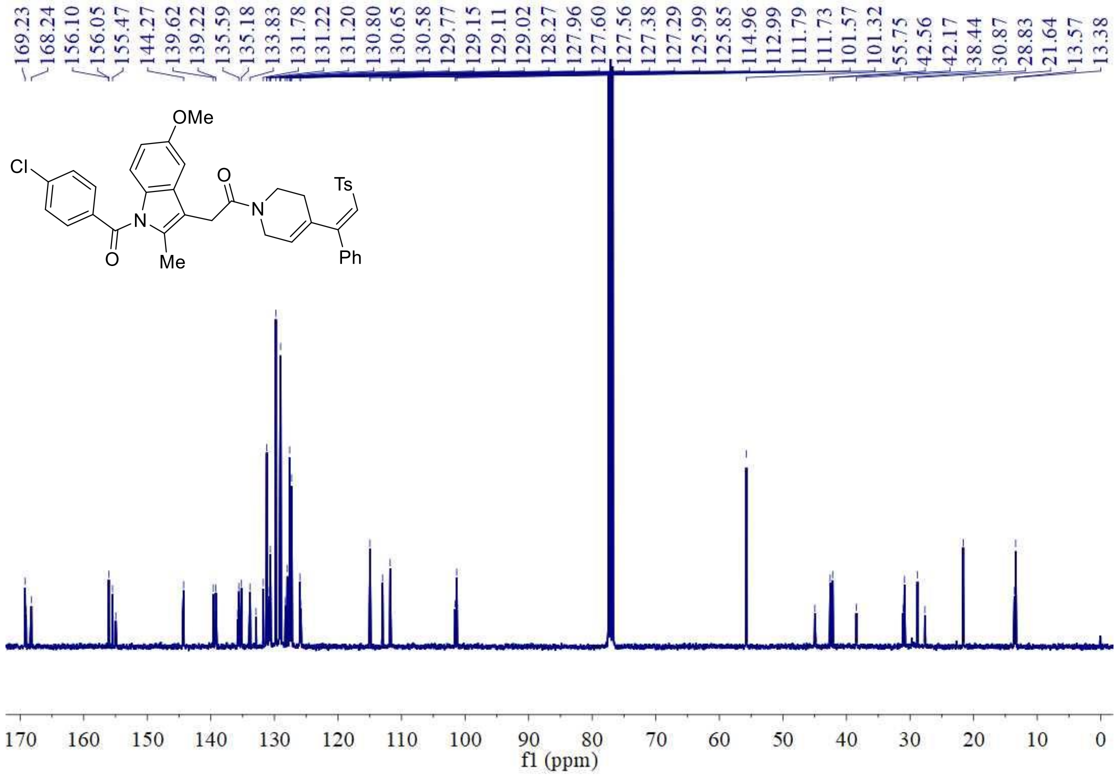

Supplementary Figure 265: ^{13}C NMR of 59a (100 MHz, $CDCl_3$)

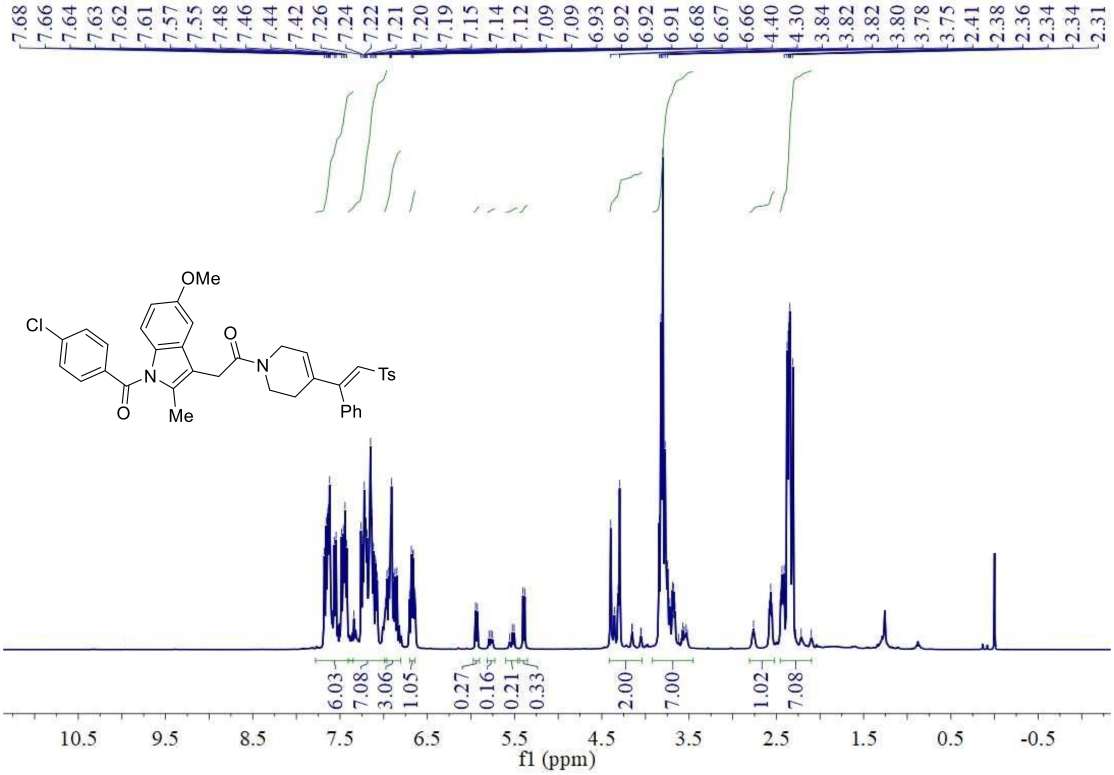

Supplementary Figure 266: ^{1}H NMR of 59b (400 MHz, $CDCl_3$)

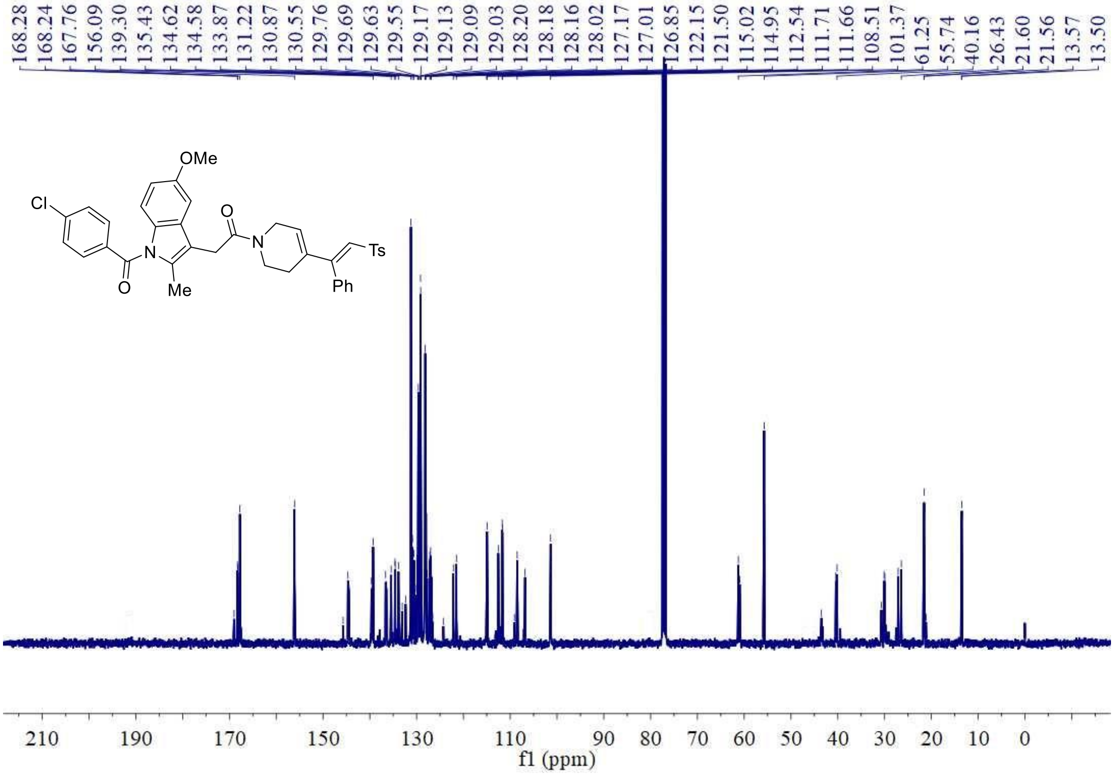

Supplementary Figure 267: ^{13}C NMR of 59b (100 MHz, $CDCl_3$)

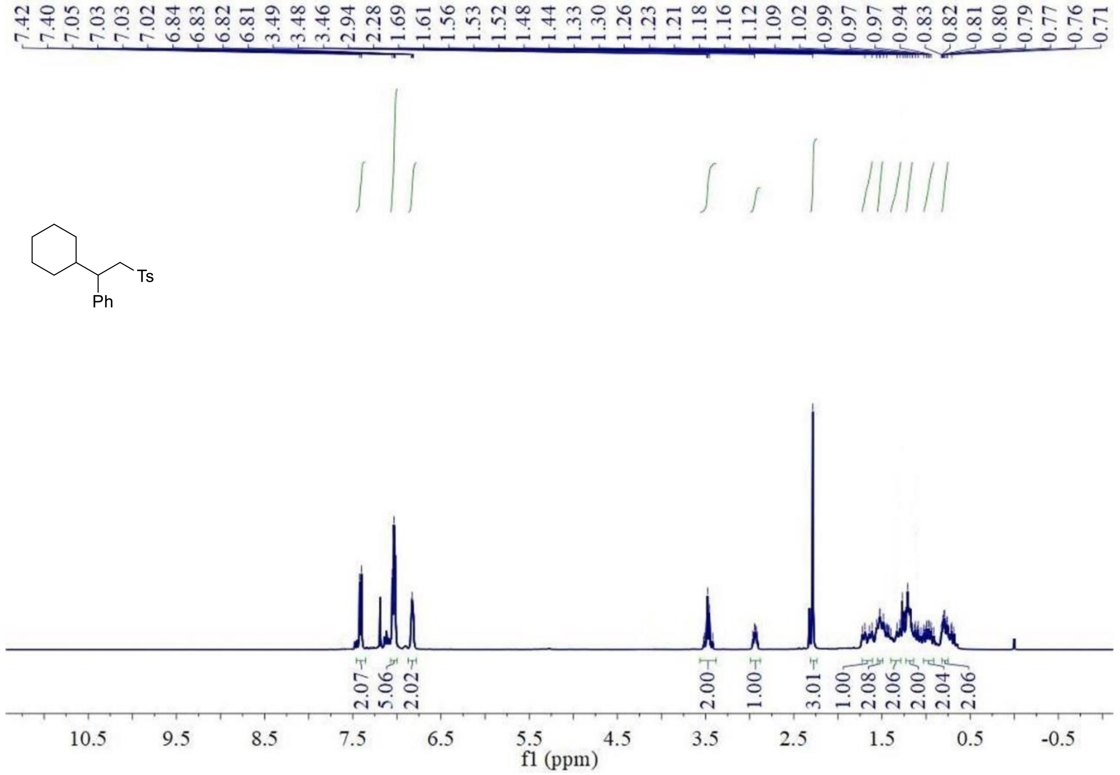

Supplementary Figure 268: ¹H NMR of 60 (400 MHz, $CDCl_3$)

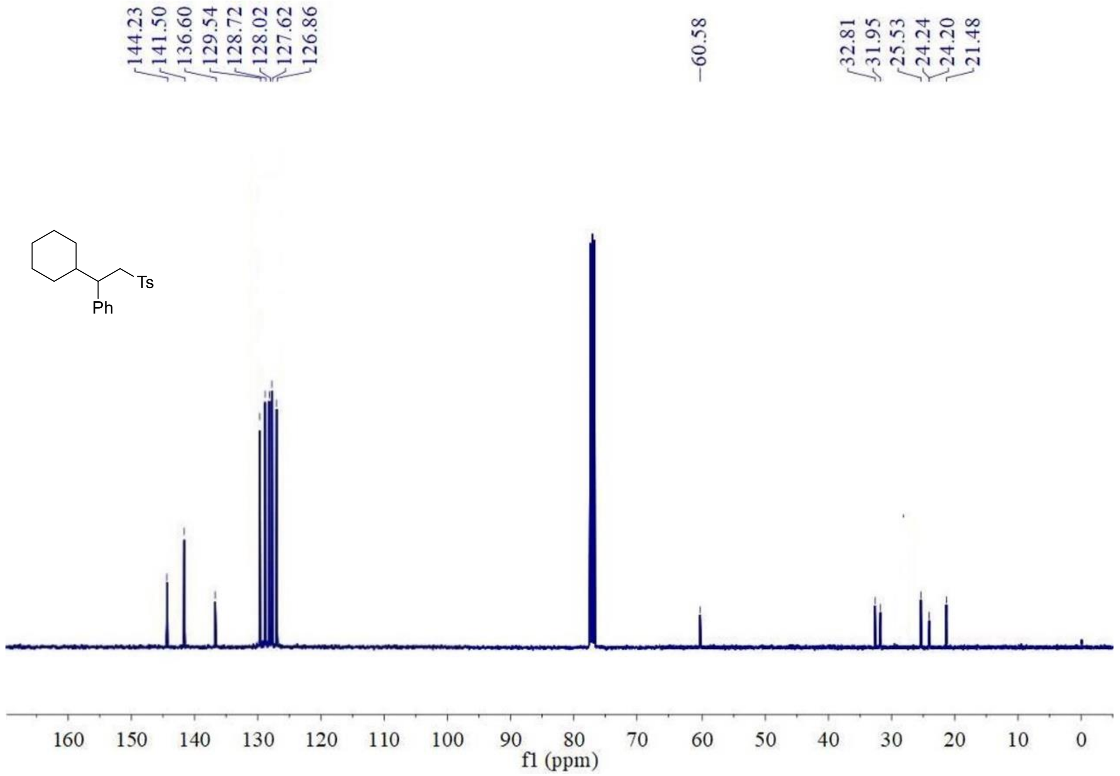

Supplementary Figure 269: ¹³C NMR of 60 (100 MHz, $CDCl_3$)

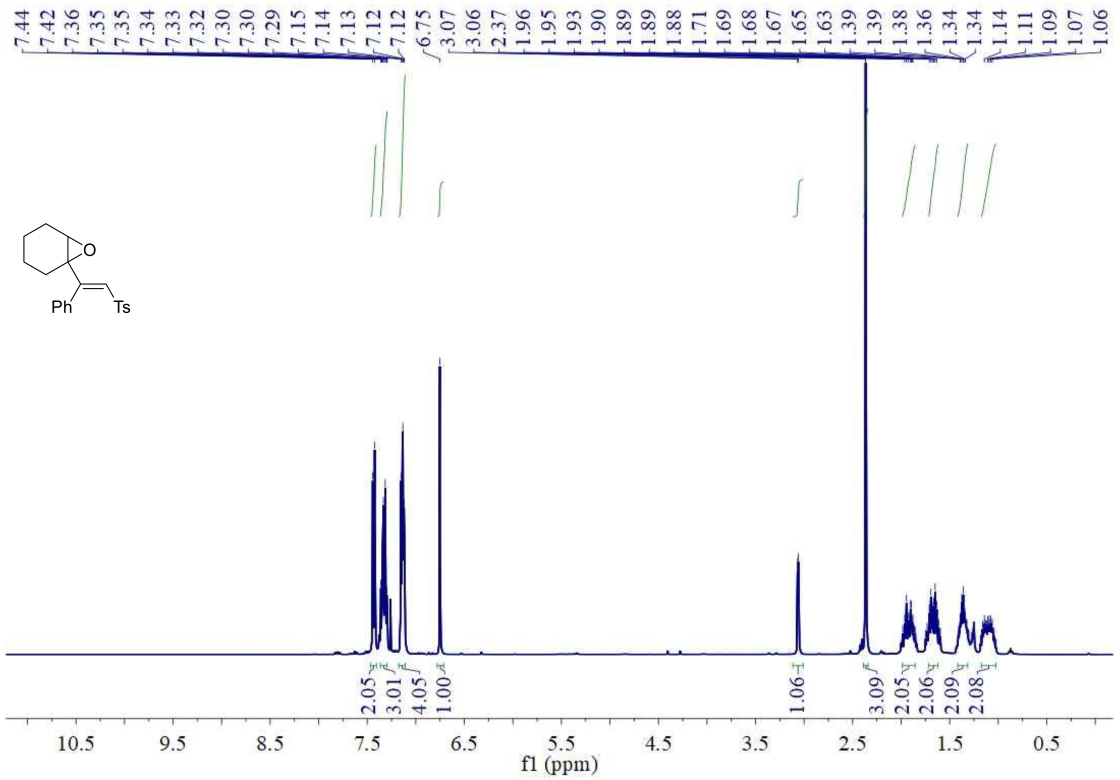

Supplementary Figure 270: ^{1}H NMR of 61 (400 MHz, $CDCl_3$)

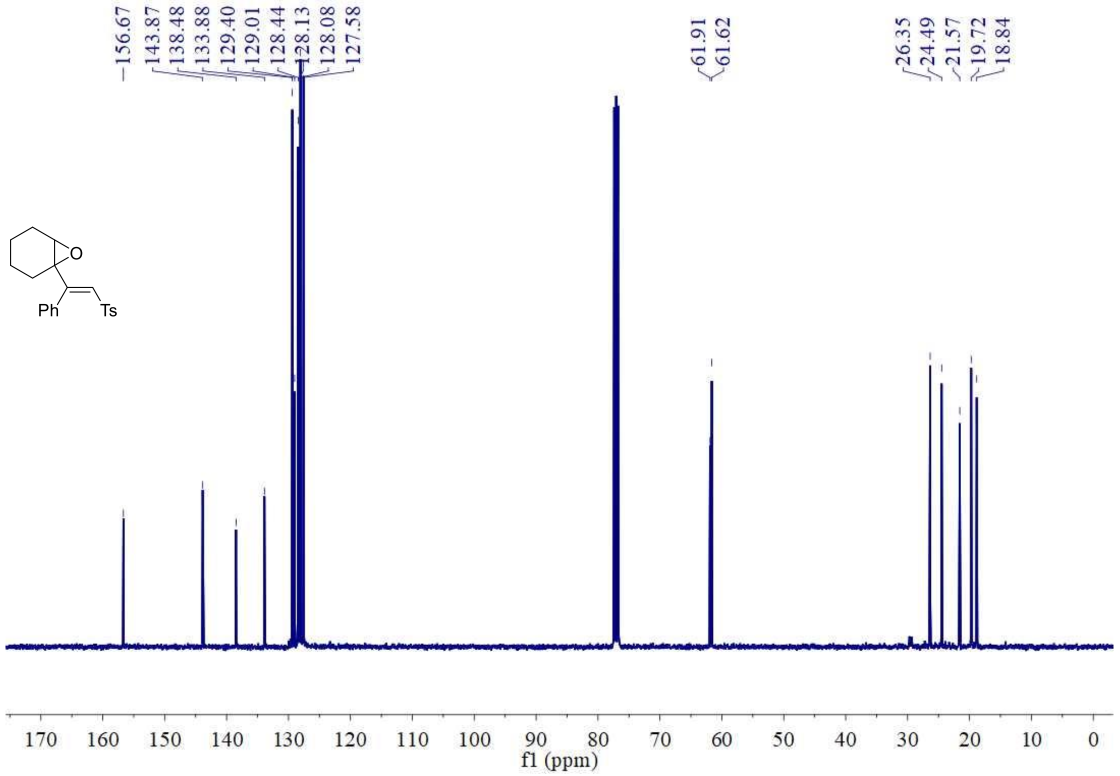

Supplementary Figure 271: ^{13}C NMR of 61 (100 MHz, $CDCl_3$)

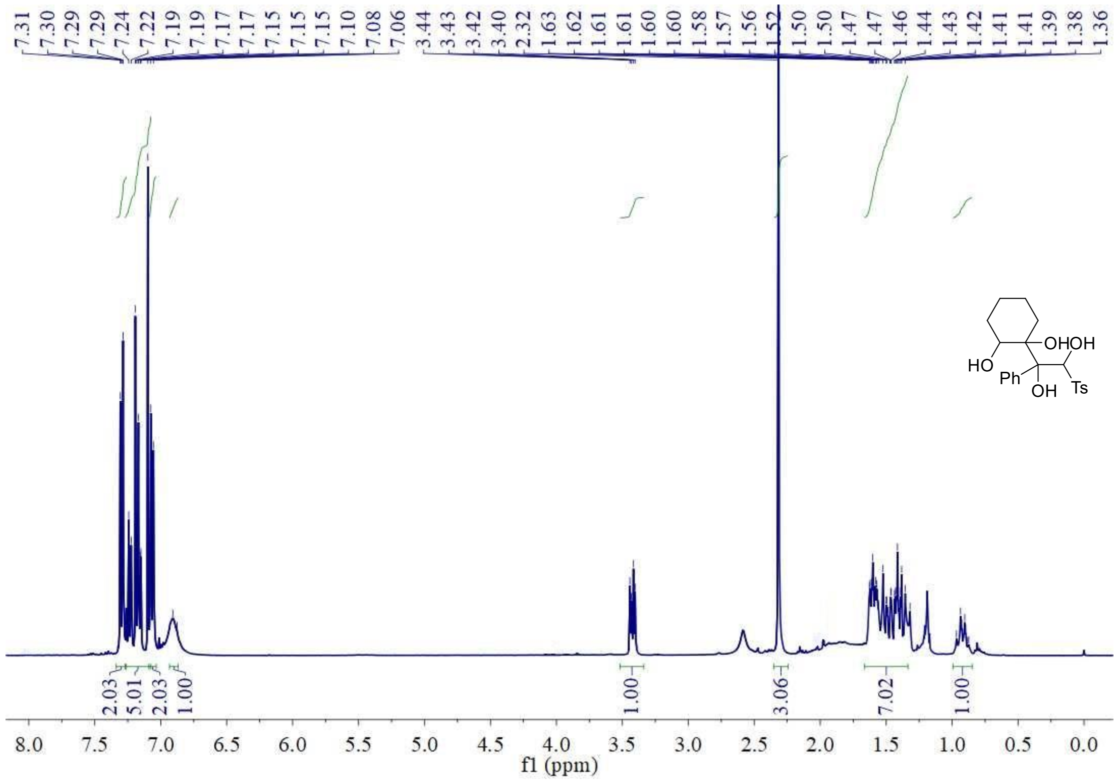

Supplementary Figure 272: ^{1}H NMR of 62 (400 MHz, $CDCl_3$)

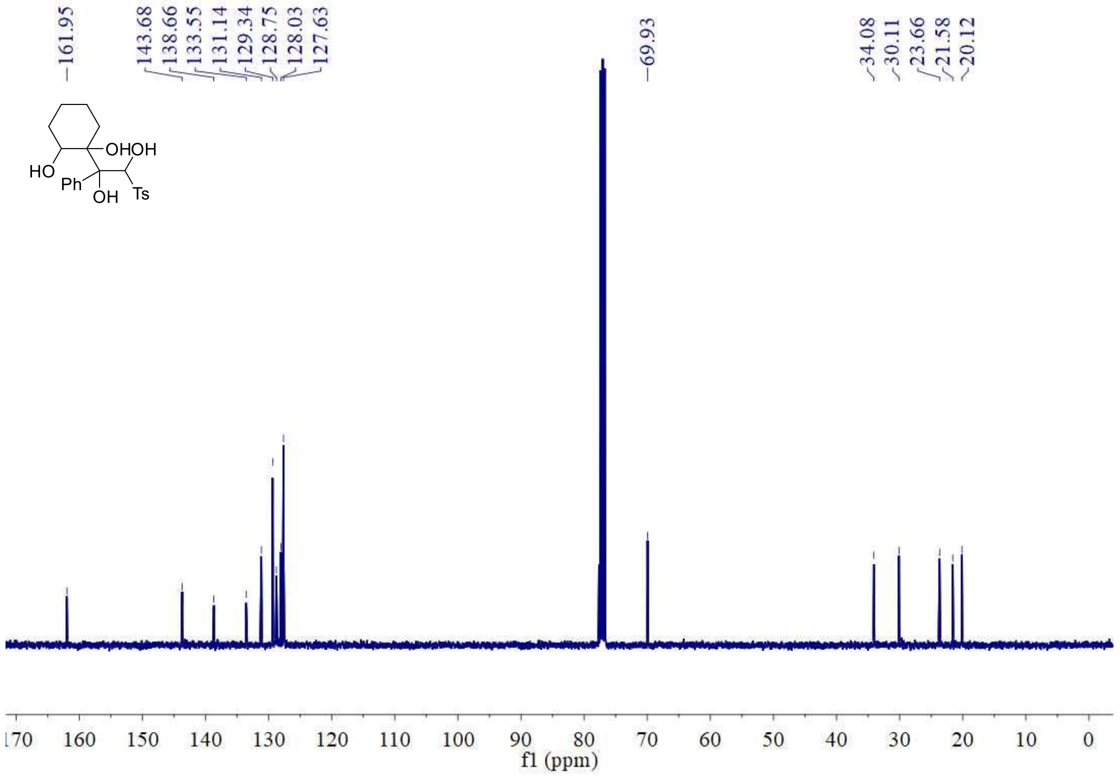

Supplementary Figure 273: ^{13}C NMR of 62 (100 MHz, $CDCl_3$)

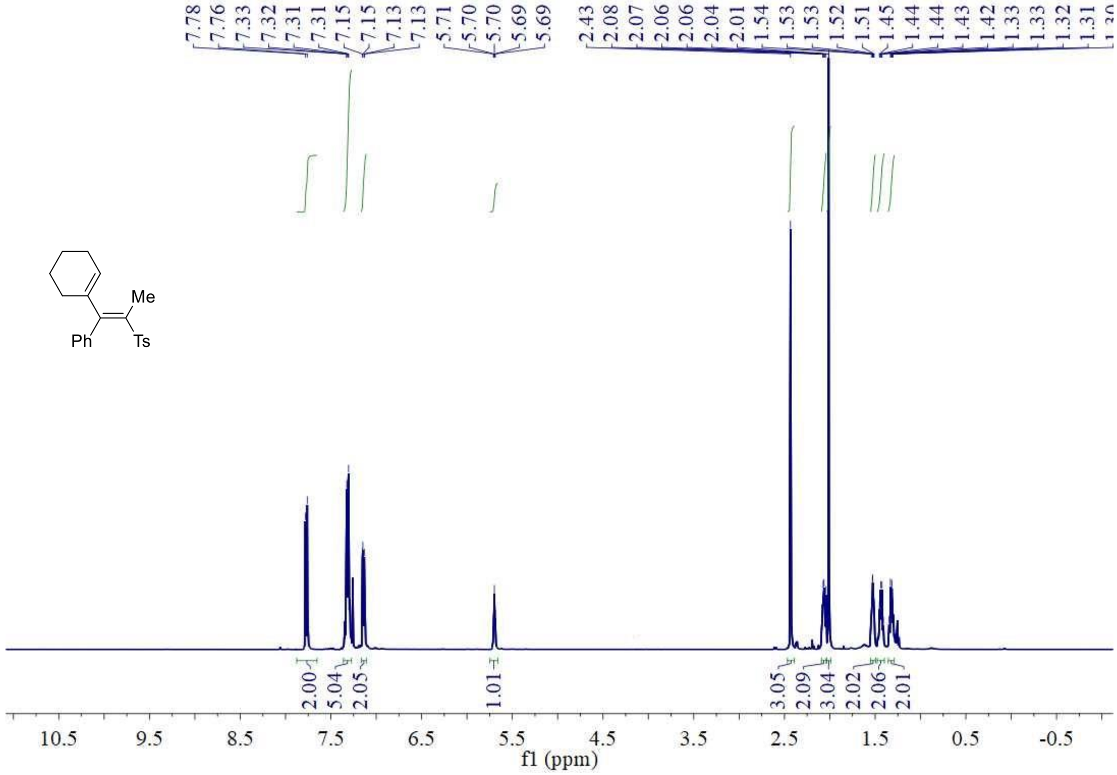

Supplementary Figure 274: ^{1}H NMR of 63 (400 MHz, $CDCl_3$)

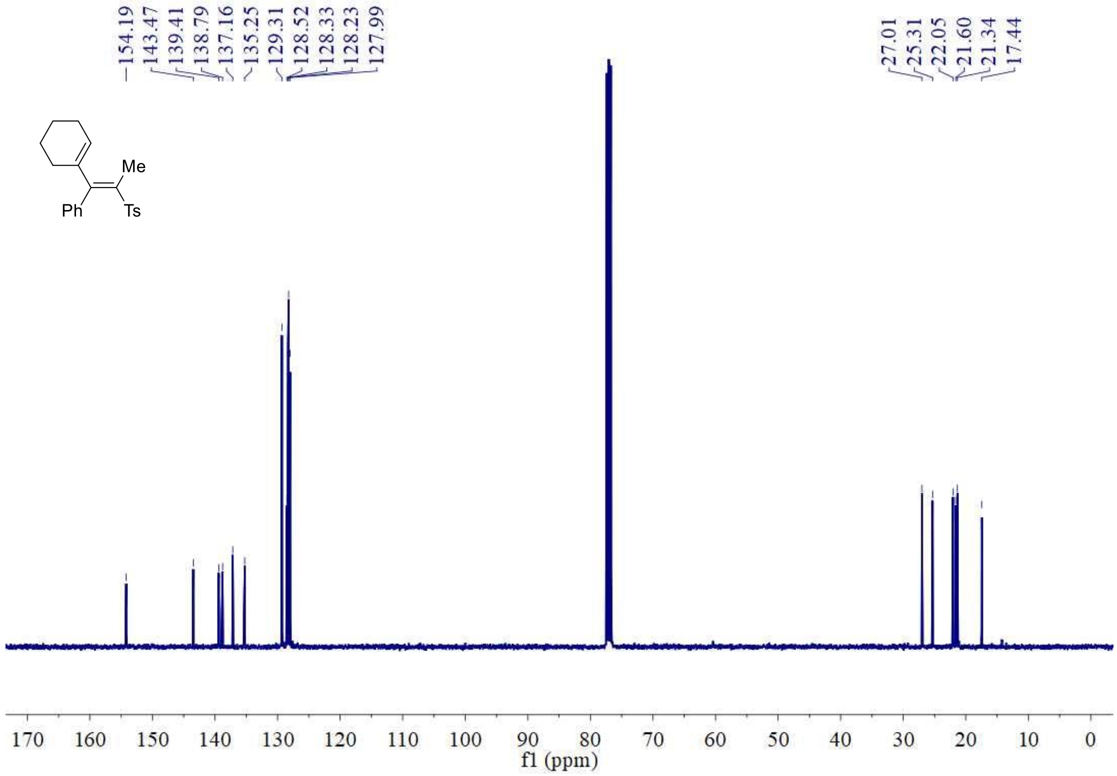

Supplementary Figure 275: ^{13}C NMR of 63 (100 MHz, $CDCl_3$)

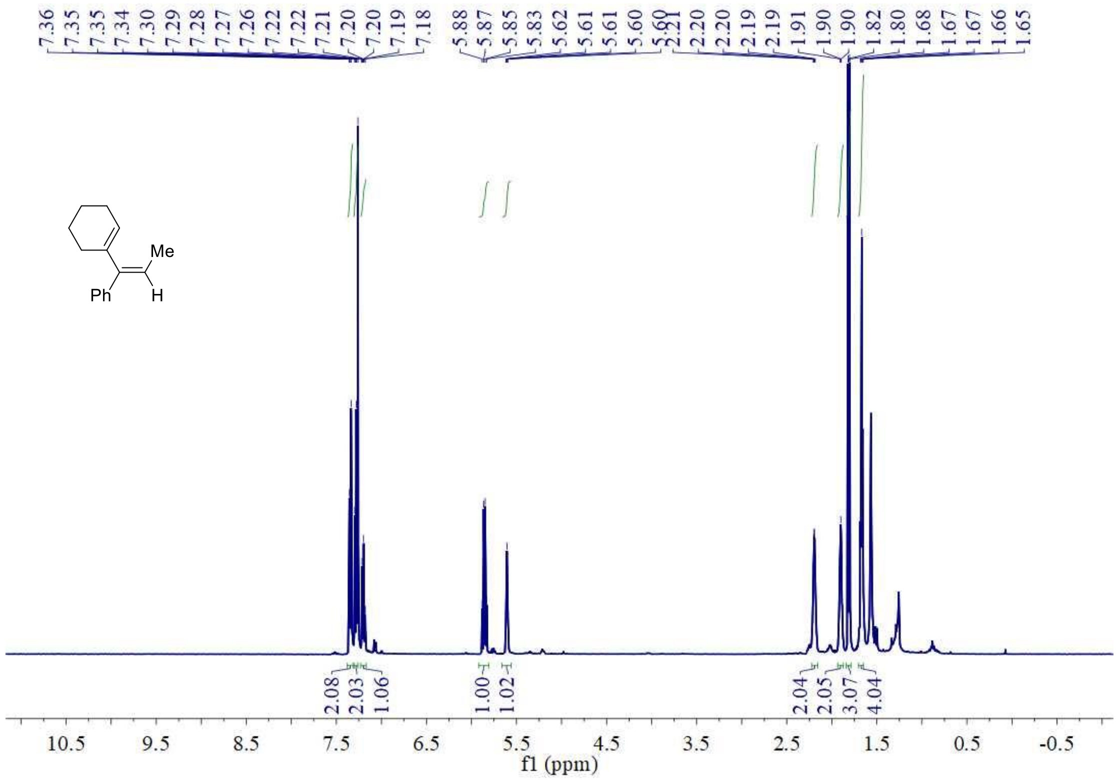

Supplementary Figure 276: ^{1}H NMR of 64 (400 MHz, $CDCl_3$)

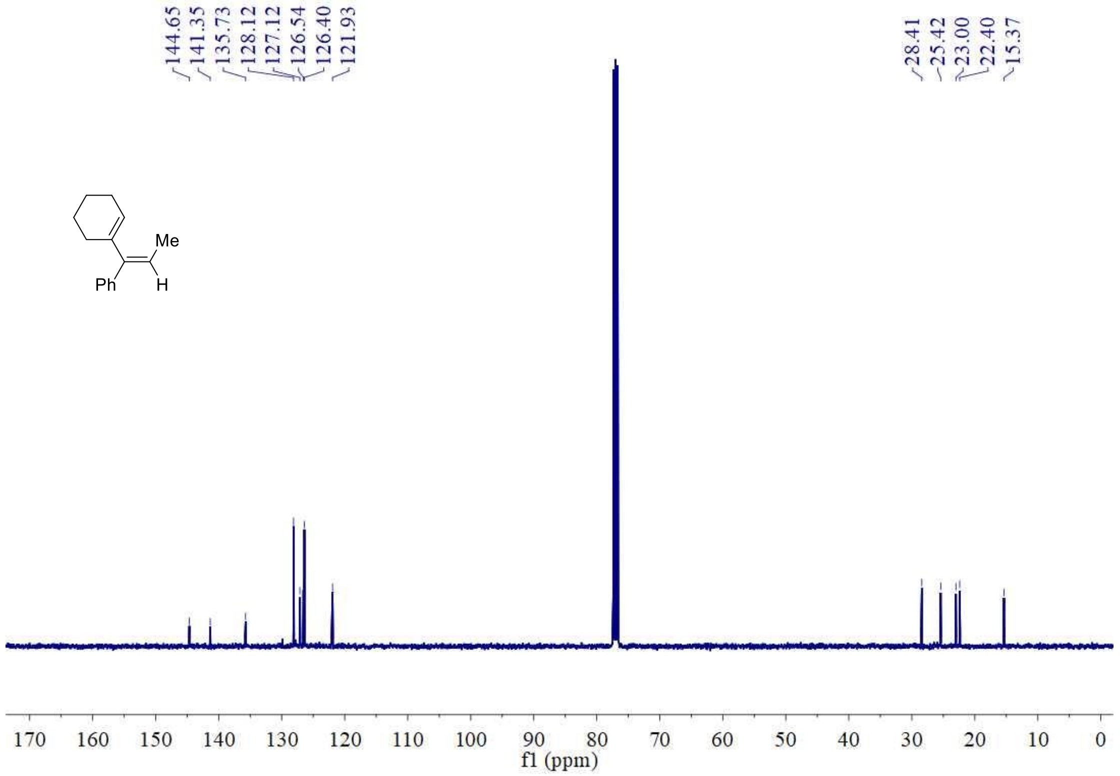

Supplementary Figure 277: ^{13}C NMR of 64 (100 MHz, $CDCl_3$)

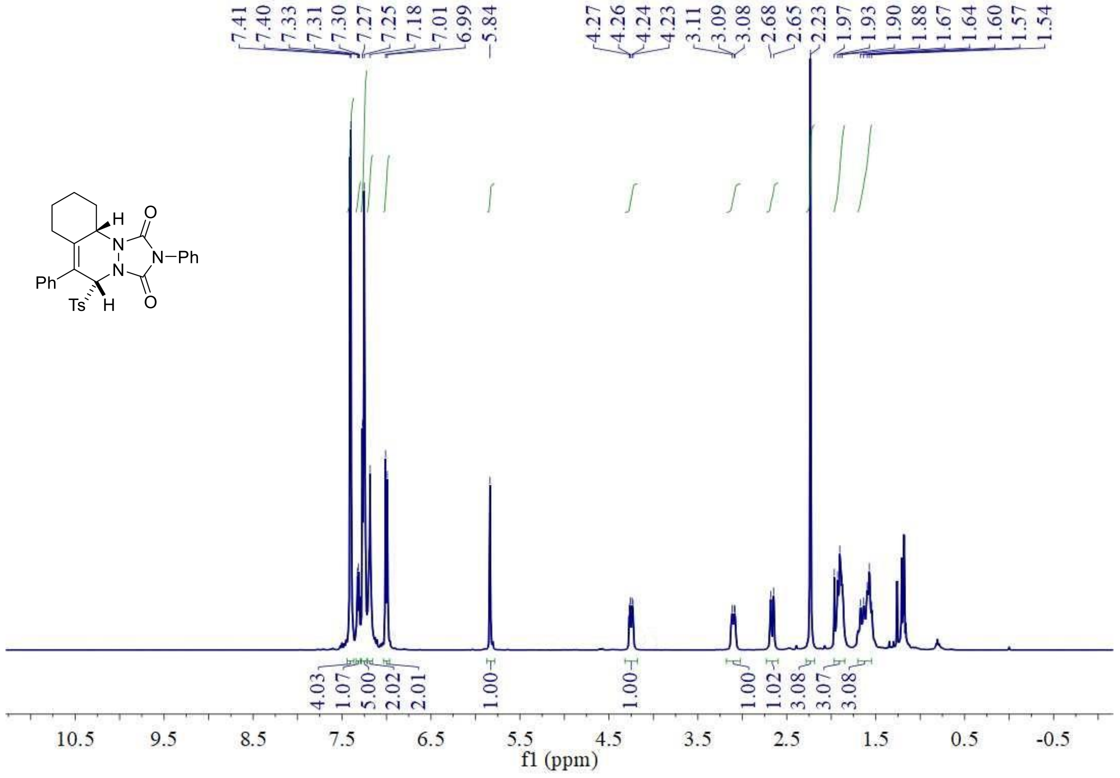

Supplementary Figure 278: ^{1}H NMR of 66 (400 MHz, $CDCl_3$)

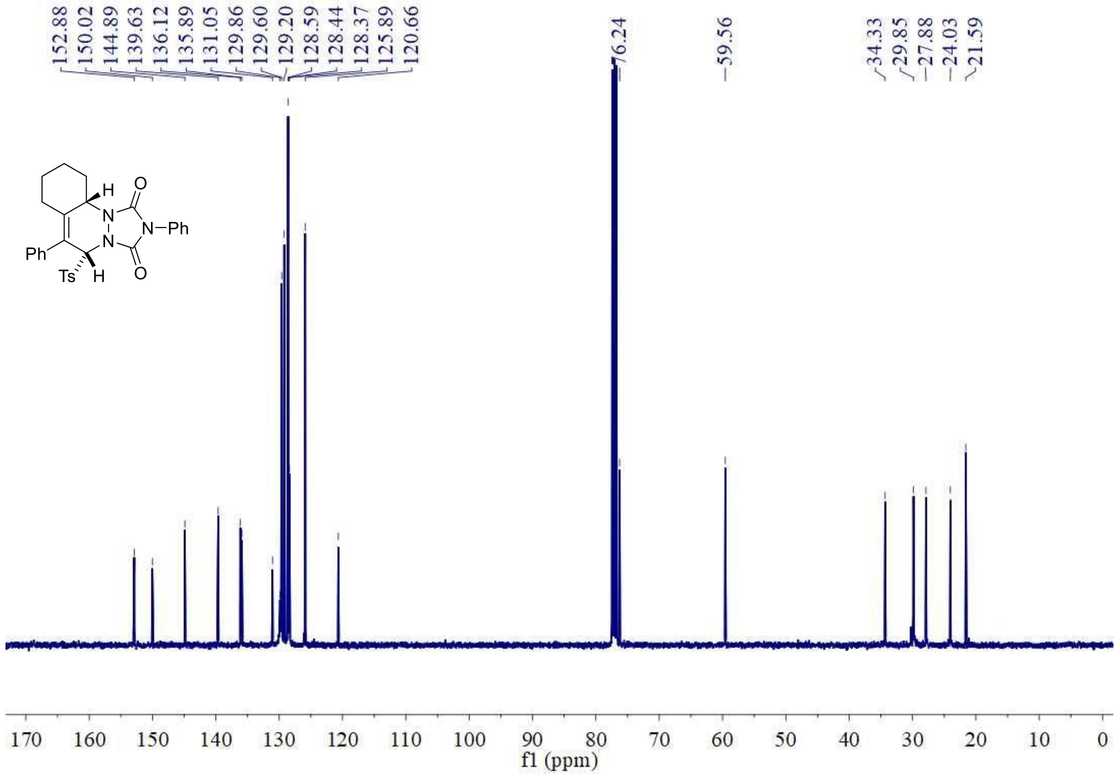

Supplementary Figure 279: ^{13}C NMR of 66 (100 MHz, $CDCl_3$)

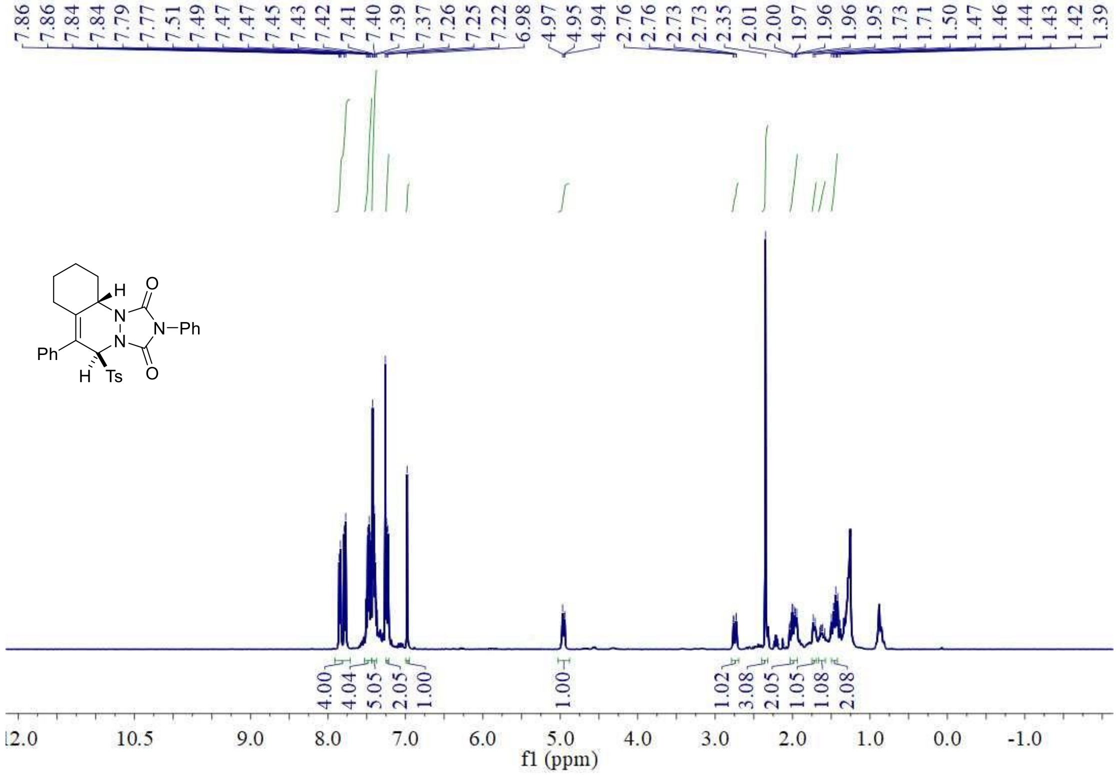

Supplementary Figure 280: ^{1}H NMR of 66’ (400 MHz, $CDCl_3$)

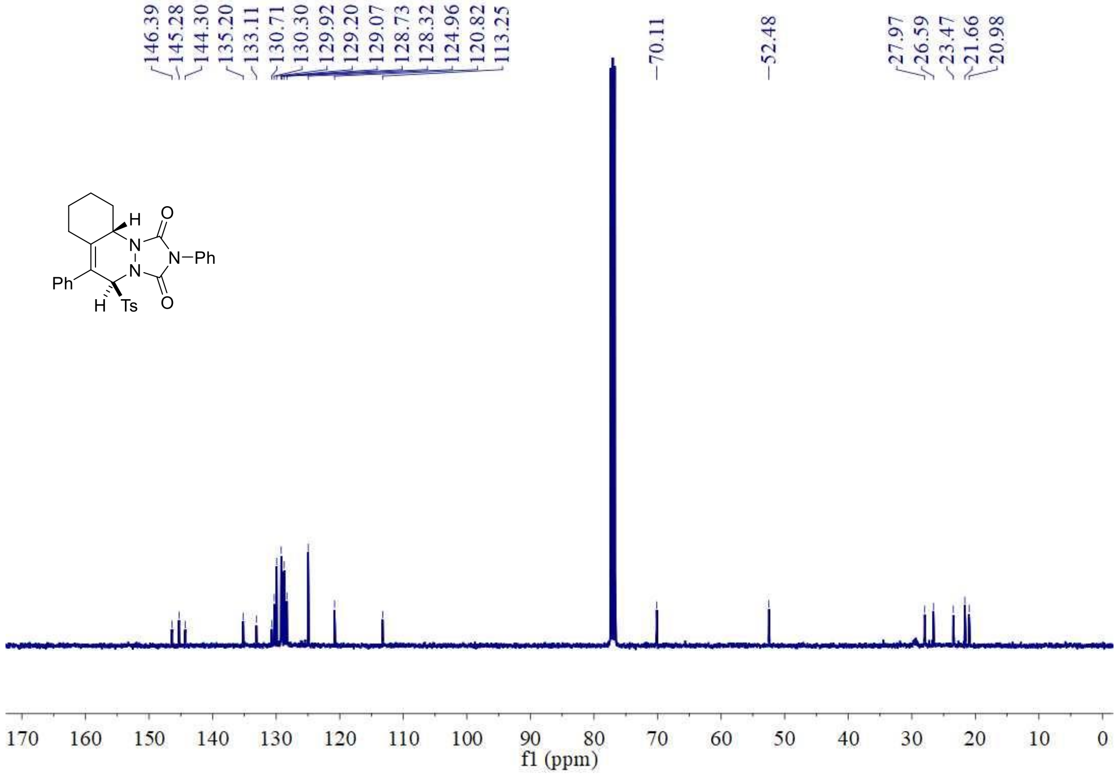

Supplementary Figure 281: ^{13}C NMR of 66’ (100 MHz, $CDCl_3$)

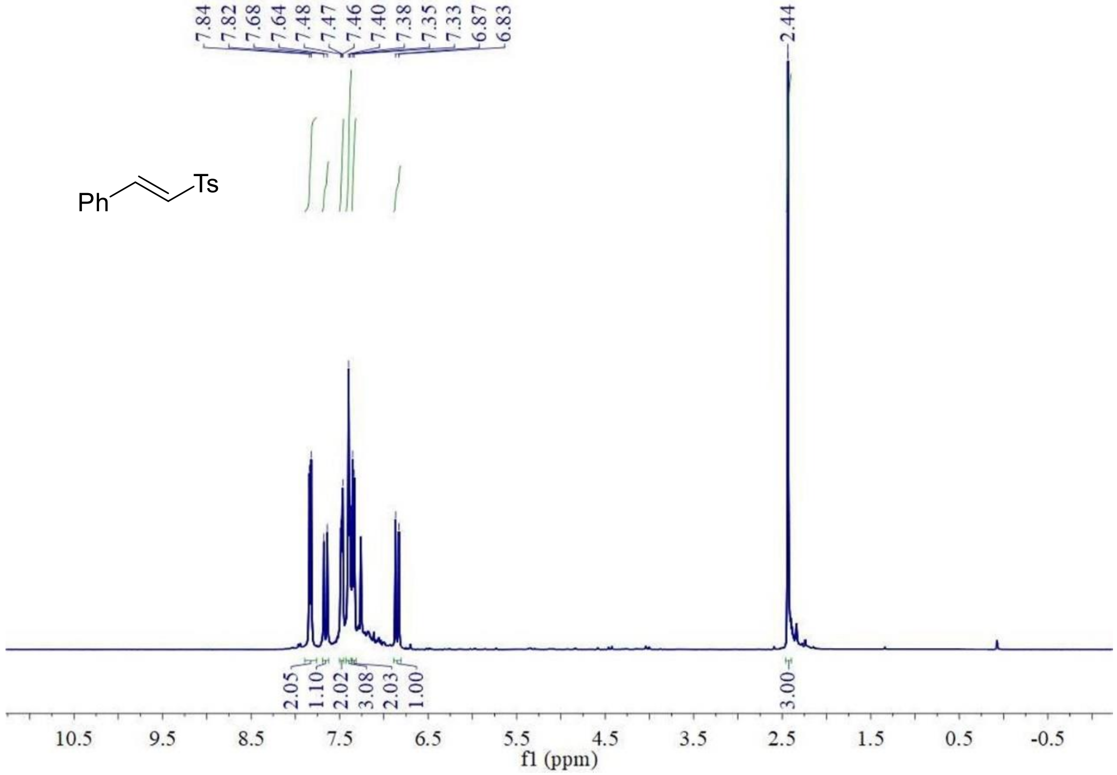

Supplementary Figure 282: ^{1}H NMR of 67 (400 MHz, $CDCl_3$)

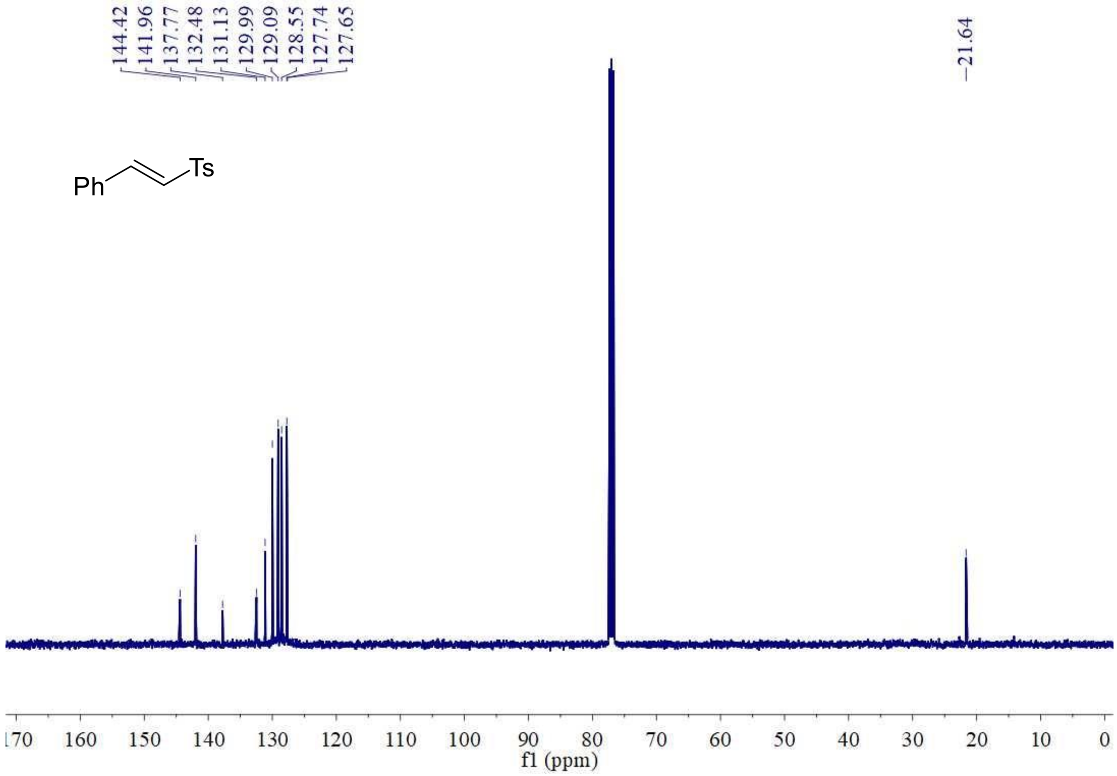

Supplementary Figure 283: ^{13}C NMR of 67 (100 MHz, $CDCl_3$)

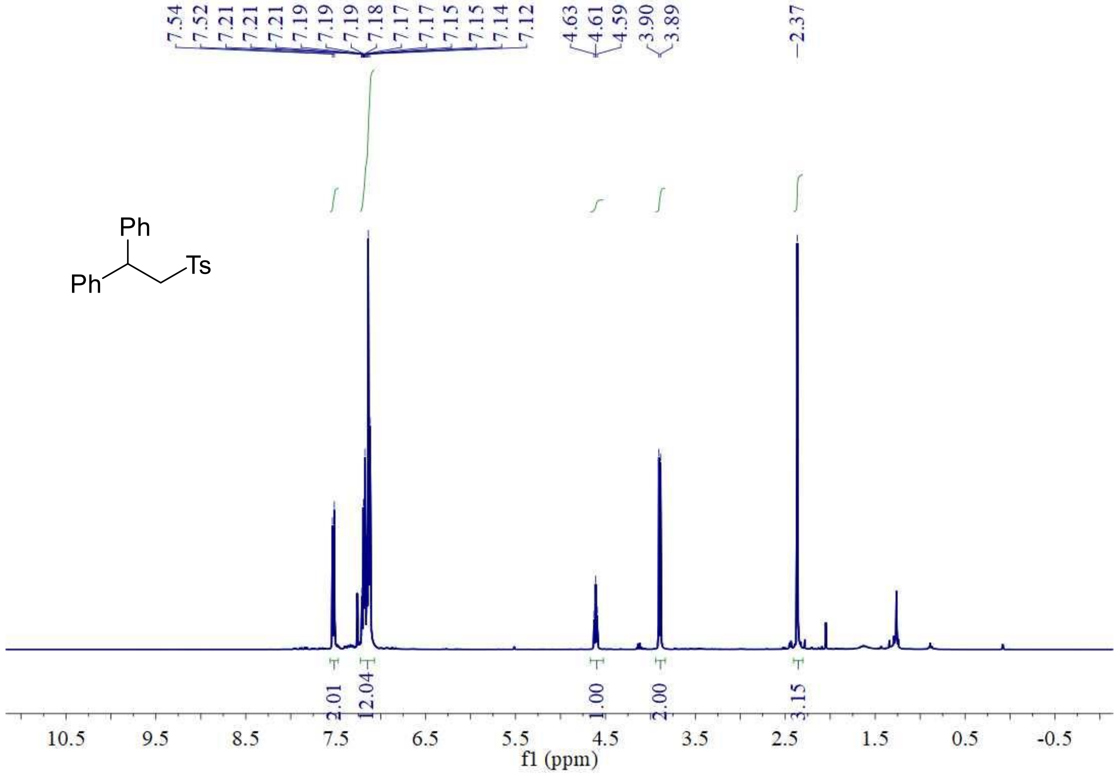

Supplementary Figure 284: ^{1}H NMR of 68 (400 MHz, $CDCl_3$)

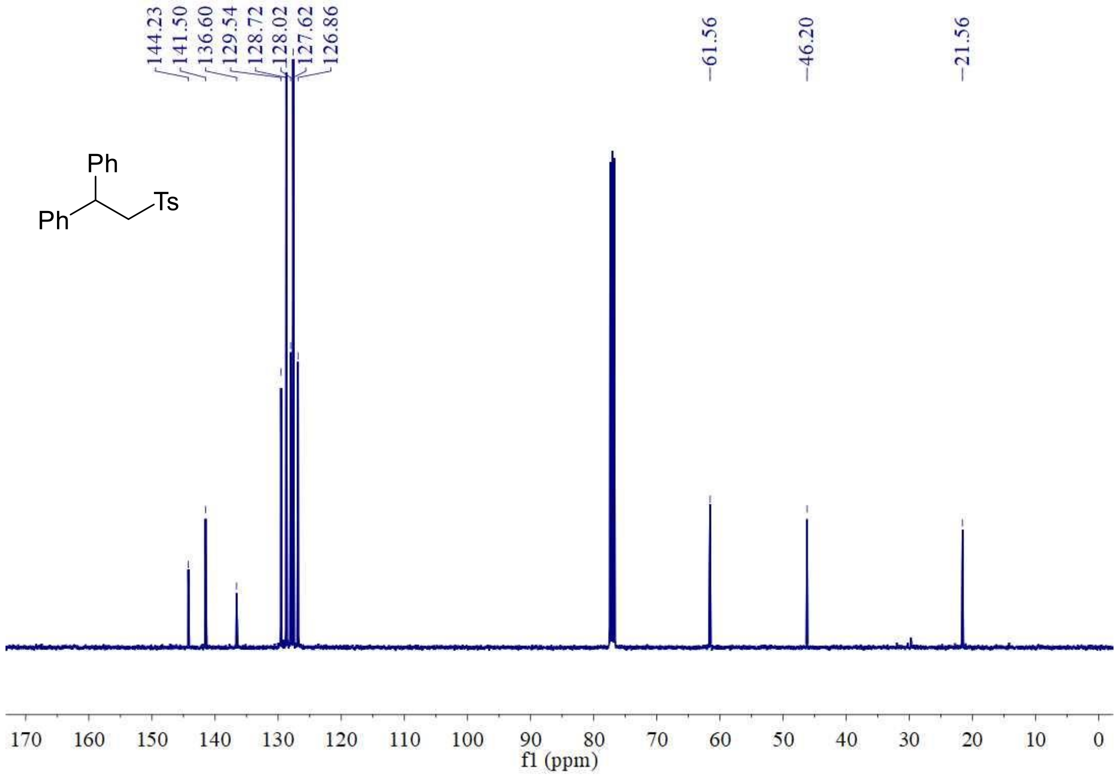

Supplementary Figure 285: ^{13}C NMR of 68 (100 MHz, $CDCl_3$)

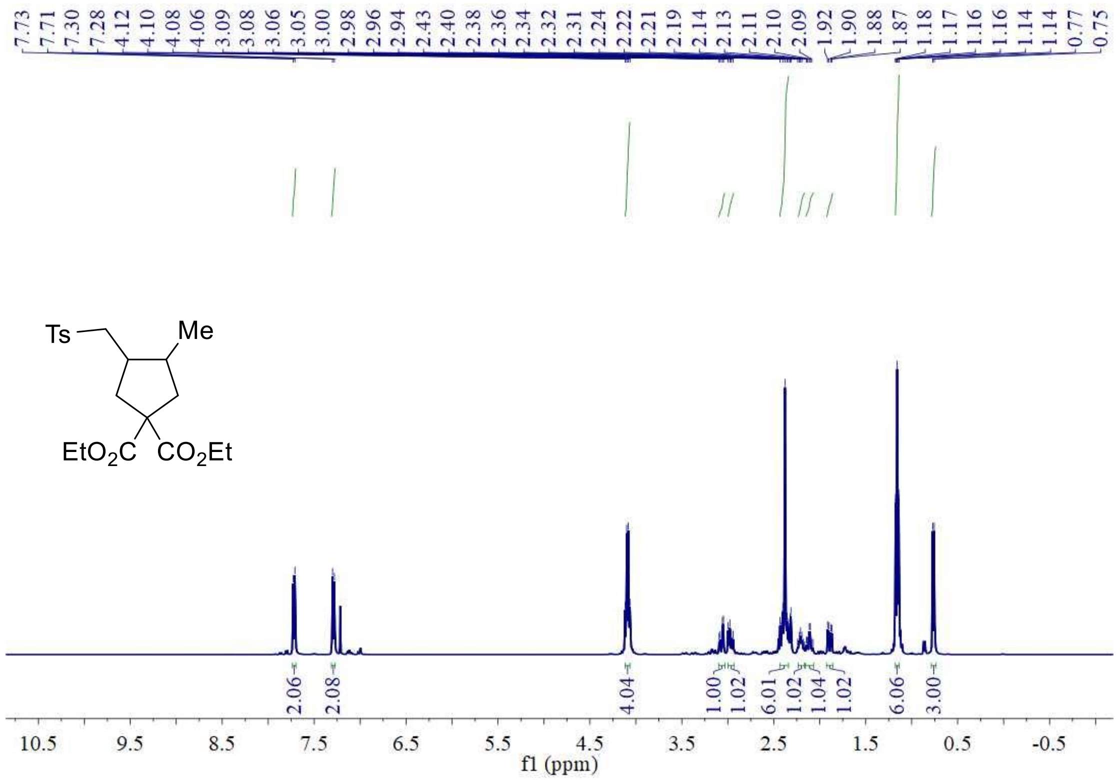

Supplementary Figure 286: ^{1}H NMR of 70 (400 MHz, $CDCl_3$)

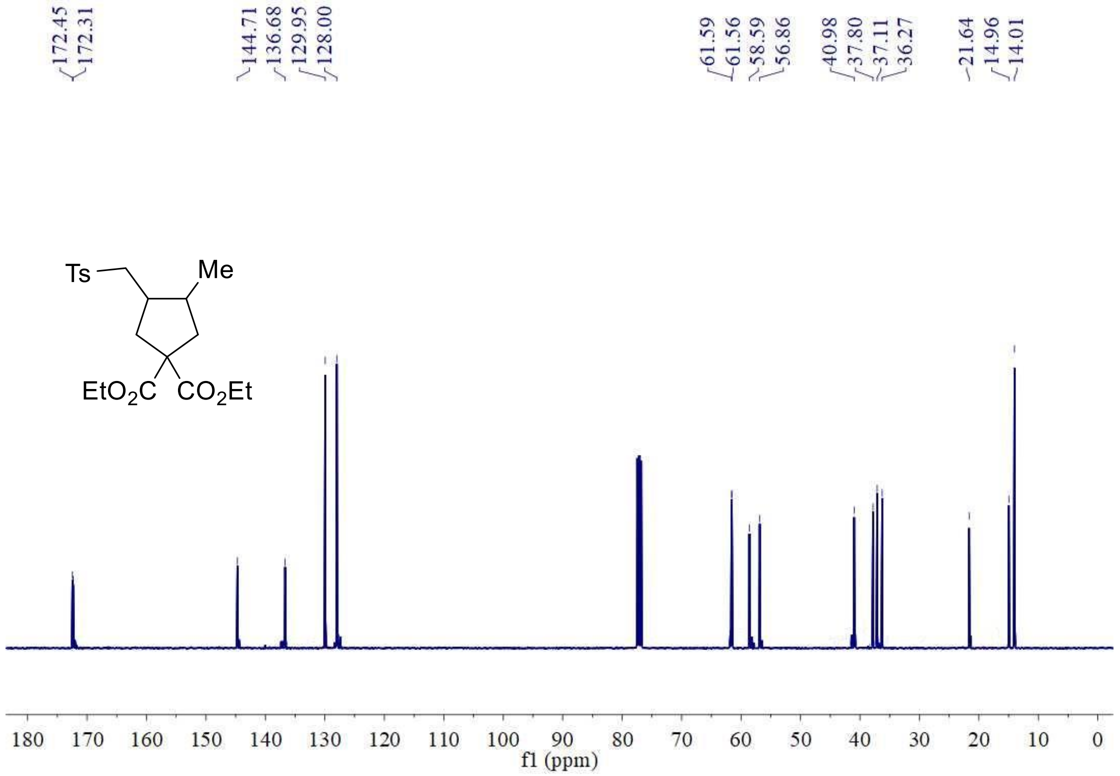

Supplementary Figure 287: ^{13}C NMR of 70 (100 MHz, $CDCl_3$)

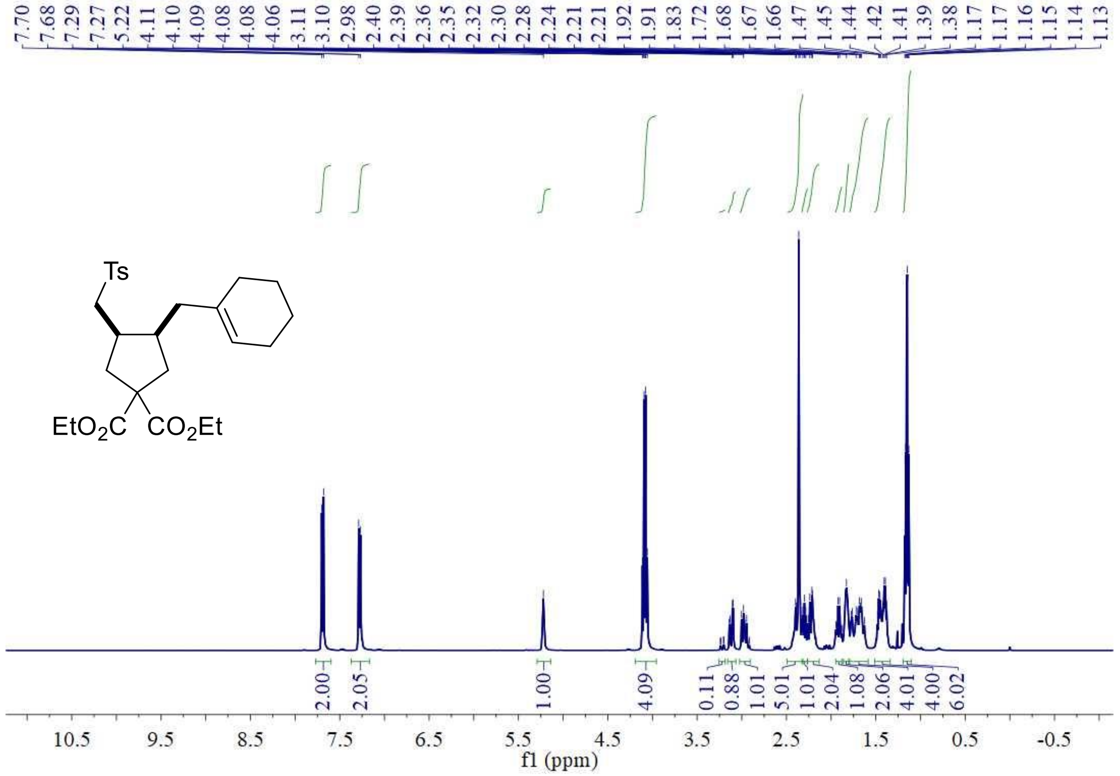

Supplementary Figure 288: ^{1}H NMR of 70' (400 MHz, $CDCl_3$)

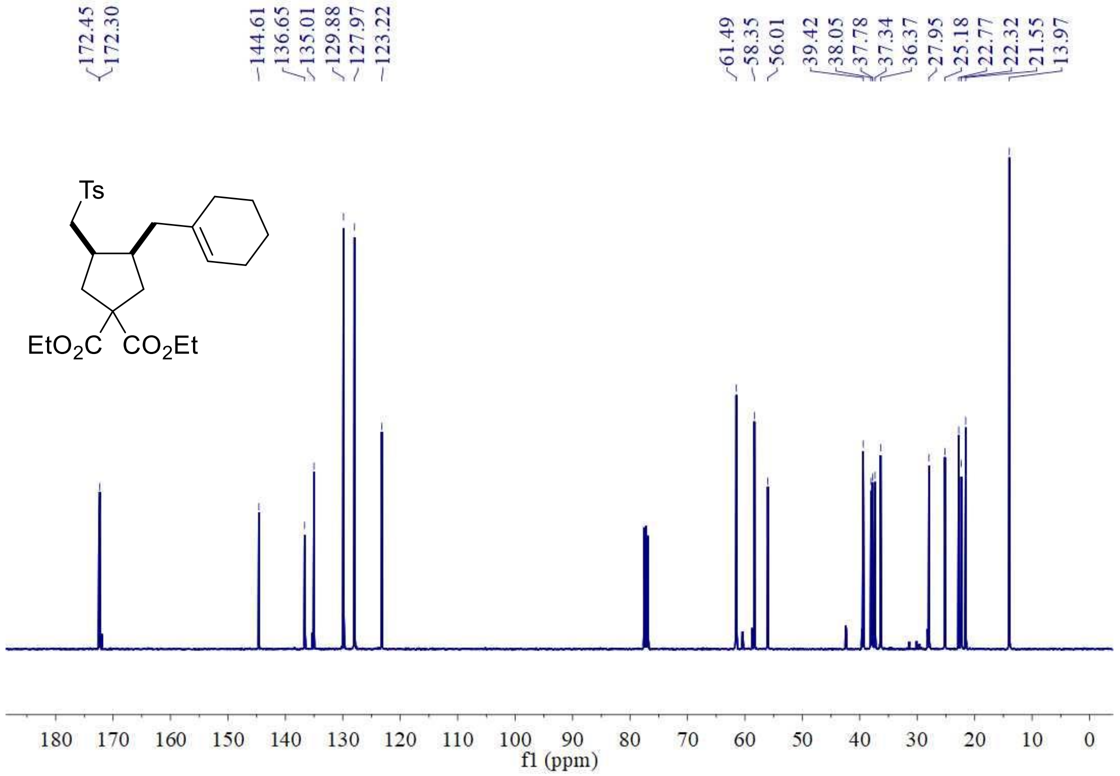

Supplementary Figure 289: ^{13}C NMR of 70' (100 MHz, $CDCl_3$)

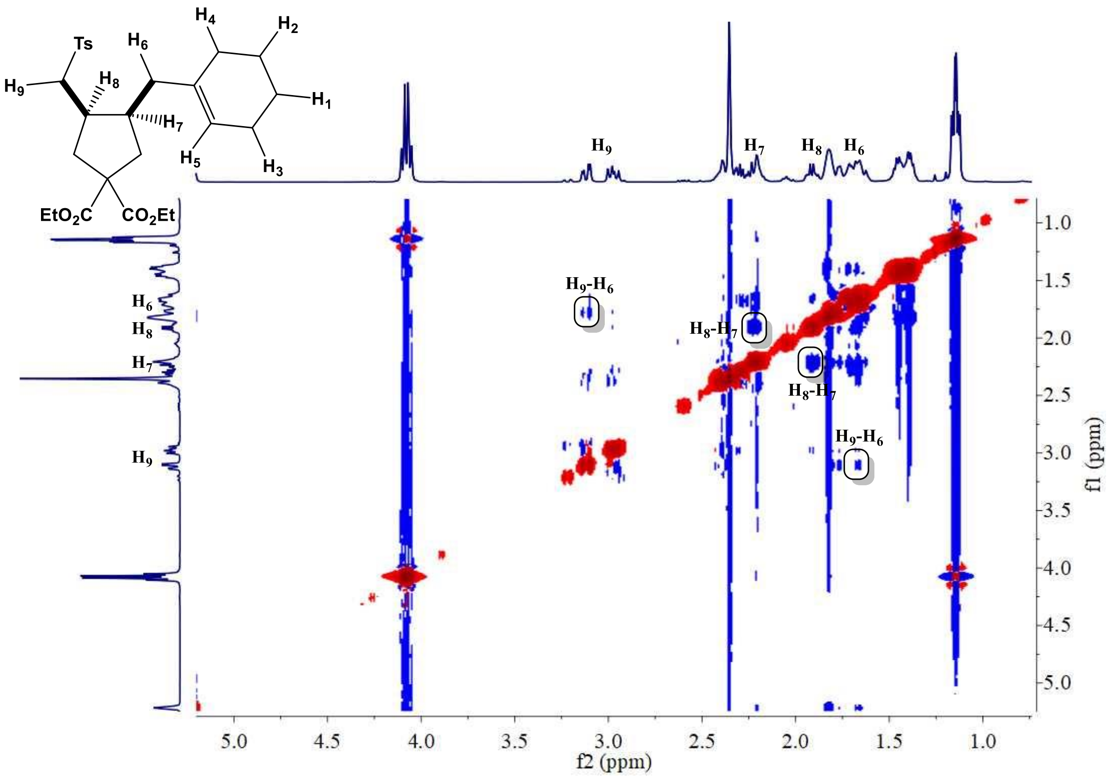

Supplementary Figure 290: NOESY of 70’

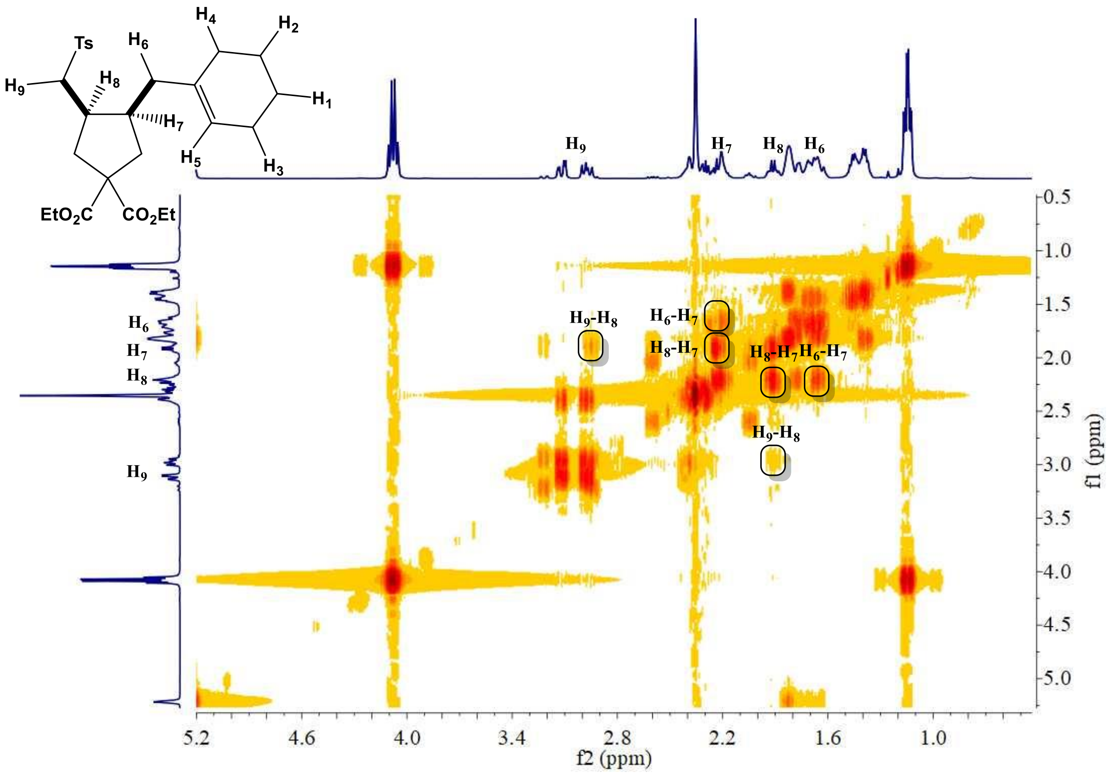

Supplementary Figure 291: COSY of 70’

Supplementary References

[1].L. Su, T. Ren, J. Dong, L. Liu, S. Xie, L. Yuan, Y. Zhou, S. F. Yin, *J. Am. Chem. Soc.* **2019**, *141*, 2535-2544.

[2].Z. X. Tian, J. B. Qiao, G. L. Xu, X. Pang, L. Qi, W. Y. Ma, Z. Z. Zhao, J. Duan, Y. F. Du, P. Su, X. Y. Liu, X. Z. Shu, *J. Am. Chem. Soc.* **2019**, *141*, 7637-7643.

[3].a) A. K. Croft, M. K. Foley, *Org. Biomol. Chem.* **2008**, *6*, 1594-600; b) D. Gartner, A. L. Stein, S. Grupe, J. Arp, A. Jacobi von Wangelin, *Angew. Chem. Int. Ed.* **2015**, *54*, 10545-10549.

[4].P. He, X. Liu, H. Zheng, et al., *Org. Lett.* **2012**, *14*, 5134-5137.

[5].K. Griesbaum, A. R. Bandyopadhyay, M. Meister, *Can. J. Chem.* **1986**, *64*, 1553-1559.

[6].Z. Luo, C. Zhao, J. Xie, H. Lu, *Synthesis* **2016**, *48*, 3696-3700.

[7].Z. Cheng, J. Xiao, C. Liu, Q. Chen, *Eur. J. Org. Chem.* **2006**, *2006*, 5581-5587.

[8].C. Cruché, W. Neiderer, S. K. Collins, *ACS Catalysis* **2021**, *11*, 8829-8836.

[9].Y. Dai, F. Wang, S. Zhu, L. Chu, *Chin. Chem. Lett.* **2022**, *33*, 4074-4078.

[10].A. Kaga, X. Wu, J. Y. J. Lim, H. Hayashi, Y. Lu, E. K. L. Yeow, S. Chiba, *Beilstein J. Org. Chem.* **2018**, *14*, 3047-3058.

[11].C. Hatchard, C. Parker, *Proc. R. Soc. London,* **1956**, *235*, 518-536.

[12].Gaussian 16, Revision B.01, M. J. Frisch, G. W. Trucks, H. B. Schlegel, G. E. Scuseria, M. A. Robb, J. R. Cheeseman, G. Scalmani, V. Barone, G. A. Petersson, H. Nakatsuji, X. Li, M. Caricato, A. V. Marenich, J. Bloino, B. G. Janesko, R. Gomperts, B. Mennucci, H. P. Hratchian, J. V. Ortiz, A. F. Izmaylov, J. L. Sonnenberg, D. Williams-Young, F. Ding, F. Lipparini, F. Egidi, J. Goings, B. Peng, A. Petrone, T. Henderson, D. Ranasinghe, V. G. Zakrzewski, J. Gao, N. Rega, G. Zheng, W. Liang, M. Hada, M. Ehara, K. Toyota, R. Fukuda, J. Hasegawa, M. Ishida, T. Nakajima, Y. Honda, O. Kitao, H. Nakai, T. Vreven, K. Throssell, J. A. Montgomery, Jr., J. E. Peralta, F. Ogliaro, M. J. Bearpark, J. J. Heyd, E. N. Brothers, K. N. Kudin, V. N. Staroverov, T. A. Keith, R. Kobayashi, J. Normand, K. Raghavachari, A. P. Rendell, J. C. Burant, S. S. Iyengar, J. Tomasi, M. Cossi, J. M. Millam, M. Klene, C. Adamo, R. Cammi, J. W.

Ochterski, R. L. Martin, K. Morokuma, O. Farkas, J. B. Foresman, and D. J. Fox, Gaussian, Inc., Wallingford CT, 2016.

[13] a) J. P. Perdew, K. Burke, M. Ernzerhof, *Phys. Rev. Lett.* **1996**, *77*, 3865-3868; b) J. P. Perdew, K. Burke, M. Ernzerhof, *Phys. Rev. Lett.* **1997**, *78*, 1396-1396.

[14] F. Weigend, R. Ahlrichs, *PCCP* **2005**, *7*, 3297-3305.

[15] Y. Zhao, D. G. Truhlar, *Theor. Chem. Acc.* **2008**, *120*, 215-241.

[16] A. V. Marenich, C. J. Cramer, D. G. Truhlar, *J. Phys. Chem. B* **2009**, *113*, 6378-6396.

[17] CYLview20; Legault, C. Y., Université de Sherbrooke, **2020** (http://www.cylview.org).
