# Supplementary material for: Ligand-controlled stereodivergent alkenylation of alkynes to access functionalized trans- and cis-1,3-dienes
Source: Nat Commun. 2023 Jan 4;14:55. doi: 10.1038/s41467-022-35688-2 (PMC9813127; doi:10.1038/s41467-022-35688-2)
Supplement: Supplementary file 2 — Description of Additional Supplementary Files [file 41467_2022_35688_MOESM2_ESM.pdf]

Description of Additional Supplementary Files

Supplementary Data 1: Cartesian Coordinates and Energies of Calculated Structures
