# Supplementary material for: Ligand-controlled stereodivergent alkenylation of alkynes to access functionalized trans- and cis-1,3-dienes
Source: Nat Commun. 2023 Jan 4;14:55. doi: 10.1038/s41467-022-35688-2 (PMC9813127; doi:10.1038/s41467-022-35688-2)
Supplement: Supplementary file 3 — Supplementary Data 1 [file 41467_2022_35688_MOESM3_ESM.pdf]

Ligand-Controlled Stereodivergent Alkenylation of Alkynes to Access Functionalized *trans*- and *cis*-1,3-Dienes

Tianyu Long,[1,†] Chen Zhu,[2,†] Ling Li,[1] Liang Shao,[1] Shengqing Zhu,[1] Magnus Rueping,[2,*] and Lingling Chu[1,*]

[1] State Key Laboratory for Modification of Chemical Fibers and Polymer Materials, Center for Advanced Low- Dimension Materials, Donghua University, College of Chemistry and Chemical Engineering, Shanghai 201620, China.
[2] King Abdullah University of Science and Technology (KAUST), KAUST Catalysis Center (KCC), Thuwal, 23955-6900 Saudi Arabia.
† These authors contributed equally.
*E-mail: lingling.chu1@dhu.edu.cn; magnus.rueping@kaust.edu.sa

Cartesian Coordinates and Energies of Calculated Structures (in Hartree)

vinyl-OTf:

E_{el} = -1195.445063
Zero-point correction = 0.160199 (Hartree/Particle)
Thermal correction to Energy = 0.174024
Thermal correction to Enthalpy = 0.174968
Thermal correction to Gibbs Free Energy = 0.117778

C	-1.29423000	-0.20082300	-0.51236300
C	-1.54421100	1.23139300	-0.14464800
C	-2.21755200	-1.18090000	-0.50671900
C	-3.04930000	1.53120200	-0.08530100
H	-1.03256500	1.88744600	-0.88030400
H	-1.05697800	1.43493300	0.83533500
C	-3.64333700	-0.92960600	-0.09883600
H	-1.91047300	-2.19303300	-0.81474800
C	-3.80872000	0.40977100	0.63350300
H	-3.44143400	1.63254400	-1.12098200
H	-3.21974200	2.50633500	0.41386600
H	-3.99626700	-1.76903300	0.53808500

H	-4.29385500	-0.96074400	-1.00431000
H	-3.41337100	0.30871000	1.66783300
H	-4.88421400	0.66324200	0.72766100
O	0.56320000	-0.71367300	1.57601700
S	1.15573600	-0.84950700	0.23921600
O	0.01576800	-0.50743900	-0.94053300
O	1.91021800	-2.02186100	-0.20368600
C	2.27494300	0.65051100	-0.05356000
F	3.30524400	0.57040800	0.79194500
F	1.59269900	1.77869500	0.18059000
F	2.71739800	0.64602900	-1.30994700

Phenylacetylene:

Eel = -308.265099
Zero-point correction = 0.106858
Thermal correction to Energy = 0.113445
Thermal correction to Enthalpy = 0.114390
Thermal correction to Gibbs Free Energy = 0.076300

C	0.12093700	1.22065300	0.00000300
C	-0.59969600	-0.00000300	0.00001100
C	0.12094700	-1.22065600	0.00000400
C	1.52082400	-1.21518000	-0.00003600
C	2.22549800	0.00000700	-0.00006000
C	1.52081600	1.21518800	-0.00003600
H	-0.43563000	2.16891900	0.00002400
H	-0.43561700	-2.16892300	0.00002600
H	2.06851200	-2.16980500	-0.00004700
H	3.32596300	0.00000800	-0.00008800
H	2.06849300	2.16981900	-0.00004800
C	-2.02967400	-0.00001400	0.00007200
C	-3.25555700	-0.00002300	0.00009600
H	-4.33629200	0.00015100	-0.00019400

Sulfonyl radical (SO_2Me radical):

Eel = -588.480369
Zero-point correction = 0.043341
Thermal correction to Energy = 0.048210
Thermal correction to Enthalpy = 0.049154
Thermal correction to Gibbs Free Energy = 0.015043

S	-0.21640300	0.00000000	-0.29068600

O	-0.73711000	-1.31449300	0.22020400
O	-0.73710800	1.31449500	0.22020000
C	1.59003800	-0.00000100	0.09840000
H	2.02800200	-0.91765700	-0.33269500
H	2.02800300	0.91765500	-0.33269700
H	1.65995700	0.00000100	1.20272500

OTf anion (OTf^-):

Eel = -961.684587
Zero-point correction = 0.026139
Thermal correction to Energy = 0.033556
Thermal correction to Enthalpy = 0.034500
Thermal correction to Gibbs Free Energy = -0.006775

O	1.25646700	-1.42626700	-0.31749000
O	-1.25459500	1.40713200	-0.39427200
S	-0.94519600	-0.00004700	0.00001300
O	-1.25511500	-1.04496100	-1.02142300
O	-1.25494300	-0.36209200	1.41571000
C	0.95324300	-0.00000200	-0.00007000
F	1.46352100	0.90038000	0.88074700
F	1.46363900	0.31257000	-1.22007300
F	1.46405000	-1.21293500	0.33933500

***Cis-selective* product (Z):**

Eel = -1130.783589
Zero-point correction = 0.289798
Thermal correction to Energy = 0.307298
Thermal correction to Enthalpy = 0.308243
Thermal correction to Gibbs Free Energy = 0.243839

C	0.41968900	-0.32353800	-0.08947800
C	-0.27260100	-1.49509300	-0.18857000
H	0.22778300	-2.45728500	-0.37782900
O	-2.58510200	-0.63035300	0.87943200
O	-2.62875900	-1.78887500	-1.41979100
S	-2.04708200	-1.64633700	-0.05893000
C	-2.21495400	-3.25964000	0.74967100
H	-3.30713400	-3.40811000	0.84121500
H	-1.77365000	-4.04343300	0.10721400
H	-1.74050000	-3.22183000	1.74705100
C	-0.24978800	1.00289200	-0.00339500

C	-1.24610800	1.39729900	-1.07887900
C	0.05307500	1.83802000	1.02258300
C	-2.20357000	2.48728600	-0.59546700
H	-1.80776300	0.51424100	-1.44031200
H	-0.66842000	1.75311500	-1.96529000
C	-0.61270800	3.16490000	1.25007000
H	0.78780400	1.50695400	1.77671300
C	-1.43123800	3.64615000	0.04439900
H	-2.89105500	2.03038700	0.14845900
H	-2.82691600	2.84963900	-1.43839400
H	0.14969300	3.92192400	1.53880000
H	-1.26962800	3.06788800	2.14669500
H	-0.74468400	4.08556600	-0.71312100
H	-2.11808700	4.46113800	0.35241800
C	1.90644300	-0.36091400	-0.08283900
C	2.61597400	-1.45091900	0.47364600
C	2.64812700	0.70373200	-0.64585200
C	4.01565300	-1.48385500	0.45160300
H	2.05806300	-2.26531900	0.95979100
C	4.04823700	0.66572400	-0.67550900
H	2.10974000	1.56281300	-1.07148900
C	4.73805400	-0.42772900	-0.12809700
H	4.54870600	-2.33426900	0.90334600
H	4.60635000	1.49854100	-1.12966400
H	5.83820300	-0.45264600	-0.14315100

Trans-selective **product (E):**

Eel = -1130.787040
Zero-point correction = 0.289797
Thermal correction to Energy = 0.307384
Thermal correction to Enthalpy = 0.308328
Thermal correction to Gibbs Free Energy = 0.243441

C	-0.27805500	-0.24760900	0.05296700
C	0.44719600	-1.40359100	0.03238300
H	-0.01573500	-2.39678700	0.12696300
O	2.77046800	-0.51375100	-1.01537800
O	2.75395100	-1.49945700	1.36404900
S	2.23257900	-1.47798700	-0.02834800
C	2.49758100	-3.13931900	-0.70715700
H	3.59751600	-3.24617200	-0.74594900
H	2.06266400	-3.89377900	-0.02623500
H	2.06488600	-3.19557300	-1.72262400

C	-1.75612100	-0.28376200	0.09603700
C	-2.46167100	-1.61745500	-0.06120600
C	-2.47228700	0.86390600	0.27337200
C	-3.95555600	-1.56519500	0.28471400
H	-1.96926100	-2.37610200	0.58369900
H	-2.31449500	-1.97974900	-1.10452300
C	-3.97026400	0.94107100	0.25046800
H	-1.92623200	1.80538500	0.44210300
C	-4.62182400	-0.32508000	-0.31403600
H	-4.07454600	-1.53975600	1.39050400
H	-4.45383400	-2.49325600	-0.06249100
H	-4.27997200	1.84158600	-0.32409300
H	-4.33439100	1.13289100	1.28825100
H	-4.50074800	-0.34025600	-1.41973100
H	-5.71281200	-0.32251600	-0.11454700
C	0.41244100	1.07575500	0.02667800
C	0.20246400	1.97544600	-1.04003200
C	1.29495400	1.43629700	1.06578400
C	0.86595000	3.20875700	-1.06848000
H	-0.47416500	1.69092500	-1.85963200
C	1.94751000	2.67660700	1.03967400
H	1.48424300	0.72674300	1.88398400
C	1.73656500	3.56476500	-0.02556300
H	0.70652700	3.89558700	-1.91367100
H	2.63450300	2.94576000	1.85613000
H	2.25593600	4.53496000	-0.04755300

Ru^+ ($Ru(dtbbpy)_3^+$):

Eel = -2523.627587
Zero-point correction = 1.120816
Thermal correction to Energy = 1.185280
Thermal correction to Enthalpy = 1.186225
Thermal correction to Gibbs Free Energy = 1.017852

C	-2.10378200	-0.48758700	-2.14659300
C	-1.79536100	-2.22380200	-0.59629000
C	-3.08011700	-1.20791400	-2.82572400
H	-1.80947700	0.52034100	-2.47263300
C	-2.77393100	-2.99588600	-1.26360500
C	-3.44092900	-2.51450500	-2.39329200
H	-3.56084100	-0.74443800	-3.69809600
H	-3.00363800	-3.99462800	-0.87376800
C	-1.07011900	-2.65429800	0.58849600

C	0.59981300	-2.08863500	2.13901900
C	-1.26954800	-3.88150300	1.25377600
C	0.44335000	-3.29587600	2.81907900
H	1.33945300	-1.34466500	2.46865000
C	-0.52369400	-4.23780700	2.38554600
H	-2.03400800	-4.56772000	0.86498700
H	1.08231500	-3.49026300	3.68899400
N	-1.45290800	-0.96803300	-1.05856400
N	-0.13382800	-1.74803500	1.05482200
C	-1.76071400	2.25067900	0.59618800
C	-2.09671700	0.51927200	2.14612600
C	-2.72677600	3.03814200	1.26374700
C	-3.06155700	1.25486300	2.82533000
H	-1.81850700	-0.49328900	2.47195000
C	-3.40135600	2.56721600	2.39332800
H	-2.94039200	4.04058700	0.87427100
H	-3.54970400	0.79889100	3.69753400
C	-1.02883600	2.66984800	-0.58857700
C	0.63245500	2.07862700	-2.13875800
C	-1.20950000	3.89985900	-1.25403700
H	1.36082800	1.32357700	-2.46824900
C	-0.45815500	4.24464100	-2.38574900
H	-1.96356500	4.59759300	-0.86545700
N	-1.43814000	0.98958400	1.05828100
C	2.83197900	-0.44348900	-0.59350300
C	1.48562300	-1.58049600	-2.14318500
C	3.98576600	-0.90131000	-1.26040200
C	2.60072100	-2.06562800	-2.82571600
H	0.46649600	-1.83146100	-2.47100300
C	3.90631000	-1.72212000	-2.39430000
H	4.96819600	-0.59934500	-0.87286100
H	2.43730800	-2.71187900	-3.69668100
C	2.83833500	0.39962900	0.59411800
C	1.50927300	1.55716300	2.14353200
C	3.99889300	0.84010900	1.26095600
H	0.49408300	1.82359400	2.47139000
C	3.93183400	1.66242000	2.39457200
H	4.97665000	0.52322500	0.87348300
N	1.57280300	-0.77831500	-1.05724100
C	-4.50585000	-3.31691100	-3.14836700
C	-4.74747200	-4.69654700	-2.51368500
H	-5.10843100	-4.61321100	-1.46859600
H	-5.52185200	-5.24226600	-3.08767100
H	-3.83108300	-5.32082200	-2.51918500

C	-5.83366800	-2.52412600	-3.13259600
H	-5.73055600	-1.53153900	-3.61400300
H	-6.61739800	-3.08291200	-3.68300700
H	-6.18984900	-2.36408200	-2.09507400
C	-4.03779000	-3.51727800	-4.60871400
H	-3.88169900	-2.55225300	-5.13033900
H	-3.08566700	-4.08396600	-4.64756000
H	-4.79966600	-4.08652900	-5.17877400
C	-0.76724600	-5.57946500	3.08996200
C	0.16423500	-5.76963100	4.29922000
H	-0.04141700	-6.74661600	4.77918800
H	1.23260100	-5.76460700	4.00328900
H	0.01097500	-4.98503200	5.06731400
C	-2.23279900	-5.62960900	3.58058200
H	-2.44147000	-4.81058400	4.29801800
H	-2.95339800	-5.54061900	2.74328100
H	-2.43161000	-6.59301600	4.09236700
C	-0.51603800	-6.72834700	2.08604100
H	0.52711000	-6.70974800	1.71142700
H	-0.68771800	-7.70791800	2.57643000
H	-1.19316500	-6.66987500	1.21066500
Ru	0.00428500	-0.00034900	0.00015200
N	1.58434000	0.75357500	1.05772200
N	-0.10649500	1.74928400	-1.05469600
C	0.49454300	3.28806000	-2.81894600
H	1.13663700	3.47259400	-3.68871000
C	2.63156700	2.02560900	2.82592300
H	2.47787500	2.67440300	3.69675700
C	-4.45312000	3.38645800	3.14877400
C	-4.67233200	4.77010800	2.51471900
H	-3.74590900	5.37938900	2.52051700
H	-5.03461000	4.69315400	1.46959800
H	-5.43773100	5.32806600	3.08898400
C	-5.79360600	2.61528000	3.13275200
H	-6.15250800	2.46171400	2.09519000
H	-5.70649500	1.62083000	3.61348600
H	-6.56809400	3.18634000	3.68365100
C	-3.98178300	3.57857400	4.60918400
H	-3.02053100	4.12962100	4.64822300
H	-4.73423700	4.15993900	5.17953300
H	-3.84143200	2.61091400	5.13039900
C	-0.68147500	5.58958000	-3.09056700
C	0.25386800	5.76602300	-4.29893100
H	0.06301600	6.74589000	-4.77913100

H	1.32174500	5.74533400	-4.00192300
H	0.08986700	4.98373400	-5.06716200
C	-2.14569800	5.66072200	-3.58260900
H	-2.36546700	4.84461300	-4.30005400
H	-2.86824600	5.58225800	-2.74593300
H	-2.33014500	6.62677800	-4.09477900
C	-0.41471500	6.73496900	-2.08668500
H	0.62769500	6.70144600	-1.71105300
H	-0.57176000	7.71677700	-2.57750100
H	-1.09347100	6.68658400	-1.21195400
C	5.18118300	-2.20242100	-3.10074400
C	6.03235400	-3.01922000	-2.10100700
H	6.96056200	-3.37538600	-2.59199600
H	6.33050900	-2.41599700	-1.22040400
H	5.47600800	-3.90503200	-1.73410200
C	5.98338500	-0.97082600	-3.58110200
H	6.91158700	-1.29580200	-4.09329400
H	5.39212400	-0.36335400	-4.29553600
H	6.27899300	-0.31392000	-2.73883600
C	4.86354100	-3.08885700	-4.31704100
H	4.30773700	-4.00365100	-4.02859200
H	4.27090800	-2.54641400	-5.08098200
H	5.80659900	-3.41326600	-4.79924600
C	5.21378400	2.12406800	3.10061100
C	5.99805700	0.88101400	3.58107100
H	6.28401800	0.21967700	2.73893900
H	6.93095000	1.19260000	4.09305800
H	5.39814300	0.28235100	4.29572700
C	6.07651300	2.92810500	2.10044100
H	5.53309500	3.82192100	1.73357700
H	7.01003100	3.27066700	2.59104000
H	6.36539700	2.32037200	1.21985600
C	4.90954200	3.01530200	4.31683600
H	4.36730000	3.93821300	4.02842000
H	4.30913200	2.48181100	5.08100700
H	5.85742100	3.32578900	4.79876400

Ru^{2+} ($Ru(dtbbpy)_3^{2+}$):

Eel = -2523.530832

Zero-point correction = 1.124083

Thermal correction to Energy = 1.188425

Thermal correction to Enthalpy = 1.189369

Thermal correction to Gibbs Free Energy = 1.021415

C	-2.15193000	-0.49353000	-2.10594300
C	-1.80196000	-2.23208500	-0.58192100
C	-3.15548100	-1.21009200	-2.75251700
H	-1.86321500	0.51226300	-2.44213700
C	-2.80648700	-2.99411400	-1.20170800
C	-3.51667300	-2.50506200	-2.31133500
H	-3.65895100	-0.74479900	-3.61142600
H	-3.03694400	-3.98894700	-0.80285700
C	-1.02280800	-2.68062900	0.58380500
C	0.64841000	-2.10173100	2.11332100
C	-1.17175600	-3.92828100	1.20310400
C	0.53642900	-3.33249400	2.76381200
H	1.36922800	-1.34478600	2.45371000
C	-0.39348800	-4.29556500	2.31948000
H	-1.91287600	-4.63236900	0.80238900
H	1.18857200	-3.52823500	3.62434800
N	-1.47154000	-0.98288100	-1.03939400
N	-0.11046900	-1.76227500	1.04605200
C	-1.80247800	2.23166100	0.58199000
C	-2.15192000	0.49302400	2.10604400
C	-2.80719300	2.99342700	1.20180000
C	-3.15562800	1.20932800	2.75265500
H	-1.86291900	-0.51268500	2.44224500
C	-3.51720400	2.50418400	2.31145600
H	-3.03794700	3.98818700	0.80293600
H	-3.65892600	0.74391500	3.61160000
C	-1.02350700	2.68037300	-0.58379000
C	0.64767400	2.10177800	-2.11348200
C	-1.17277200	3.92797800	-1.20310500
H	1.36857200	1.34493000	-2.45392100
C	-0.39469800	4.29538900	-2.31957600
H	-1.91398900	4.63193900	-0.80234300
N	-1.47171700	0.98254300	1.03946100
C	2.83289300	-0.45024000	-0.58244100
C	1.49854600	-1.61737400	-2.10703300
C	3.98872400	-0.94211200	-1.20215300
C	2.62135400	-2.13265900	-2.75818600
H	0.48298600	-1.86809000	-2.44493000
C	3.91963000	-1.80235900	-2.31668700
H	4.96880800	-0.64830500	-0.80371200
H	2.46610300	-2.79779700	-3.61700400
C	2.83277100	0.45104600	0.58239600
C	1.49809600	1.61780300	2.10698200

C	3.98846800	0.94322000	1.20212000
H	0.48246500	1.86822200	2.44486100
C	3.91913100	1.80343200	2.31666300
H	4.96863100	0.64967100	0.80368800
N	1.58242400	-0.78707300	-1.04210000
C	-4.61819400	-3.29813300	-3.01828400
C	-4.84459600	-4.67348400	-2.36889200
H	-5.16588300	-4.58668600	-1.31106000
H	-5.64657000	-5.21205600	-2.90949100
H	-3.93733600	-5.30962300	-2.41364000
C	-5.93390200	-2.48648100	-2.94752200
H	-5.84430300	-1.49983800	-3.44376700
H	-6.74420700	-3.04244400	-3.45953300
H	-6.24620100	-2.31759200	-1.89746300
C	-4.20846900	-3.50171700	-4.49664500
H	-4.07477800	-2.53981800	-5.03017100
H	-3.26332400	-4.07536000	-4.57680100
H	-4.99760800	-4.06897200	-5.02891600
C	-0.57486300	-5.66480200	2.98015800
C	0.37217800	-5.85291800	4.17704600
H	0.20928000	-6.85178900	4.62582200
H	1.43802000	-5.79887500	3.87668800
H	0.19088300	-5.10288800	4.97314800
C	-2.03683600	-5.78790700	3.47216200
H	-2.27725000	-5.00491000	4.21897400
H	-2.76685000	-5.70863200	2.64184800
H	-2.18839500	-6.77485400	3.95252300
C	-0.28267000	-6.76386700	1.93071200
H	0.75659100	-6.69232700	1.55213500
H	-0.41014600	-7.76383900	2.39071500
H	-0.97027800	-6.70879600	1.06308600
Ru	-0.00078400	0.00000100	-0.00002900
N	1.58220800	0.78752600	1.04205000
N	-0.11100700	1.76221400	-1.04611100
C	0.53536200	3.33249800	-2.76400000
H	1.18735800	3.52835500	-3.62462000
C	2.62075800	2.13338700	2.75814700
H	2.46532300	2.79850700	3.61694500
C	-4.61893500	3.29693700	3.01842800
C	-4.84580800	4.67218800	2.36898600
H	-3.93875700	5.30863200	2.41364300
H	-5.16713300	4.58522800	1.31117800
H	-5.64792900	5.21052800	2.90960000
C	-5.93439500	2.48487300	2.94773900

H	-6.24664900	2.31579600	1.89769700
H	-5.84449300	1.49829800	3.44406300
H	-6.74486700	3.04062500	3.45971300
C	-4.20921600	3.50071800	4.49676000
H	-3.26428300	4.07471900	4.57683800
H	-4.99853800	4.06770800	5.02904200
H	-4.07514100	2.53889500	5.03032500
C	-0.57641300	5.66457700	-2.98026900
C	0.37044000	5.85284300	-4.17728300
H	0.20729300	6.85167600	-4.62605300
H	1.43633200	5.79901000	-3.87706700
H	0.18918800	5.10276500	-4.97334900
C	-2.03846700	5.78739200	-3.47209900
H	-2.27882200	5.00431300	-4.21884600
H	-2.76837000	5.70802000	-2.64169700
H	-2.19027000	6.77428900	-3.95248700
C	-0.28429100	6.76371900	-1.93088300
H	0.75503000	6.69238100	-1.55243500
H	-0.41201200	7.76365900	-2.39088800
H	-0.97178200	6.70852900	-1.06317400
C	5.19745300	-2.32636300	-2.97760800
C	4.88948600	-3.23852500	-4.17674900
H	4.32936100	-2.70597500	-4.97163500
H	5.83705600	-3.59333700	-4.62610000
H	4.31209000	-4.13685300	-3.87883100
C	6.03407100	-1.11981800	-3.46624700
H	6.32847000	-0.44894000	-2.63443800
H	6.96578600	-1.47983400	-3.94589100
H	5.47649600	-0.51952500	-4.21282000
C	6.00282000	-3.13003300	-1.92864300
H	5.42192200	-3.99595800	-1.55285900
H	6.93399800	-3.51712800	-2.38785200
H	6.29615500	-2.50839100	-1.05909000
C	5.19680100	2.32775400	2.97763400
C	6.03353300	1.12140400	3.46656500
H	6.32808900	0.45043700	2.63488600
H	6.96515000	1.48162000	3.94625000
H	5.47595300	0.52116400	4.21317700
C	6.00218100	3.13137500	1.92864600
H	5.42119500	3.99713500	1.55261600
H	6.93322200	3.51871200	2.38793000
H	6.29577800	2.50964500	1.05924500
C	4.88854700	3.24007900	4.17657800
H	4.31101500	4.13824000	3.87842100

H	4.32843100	2.70757600	4.97150300
H	5.83600000	3.59514800	4.62596900

N1:

Eel = -2839.099914
Zero-point correction = 0.268857
Thermal correction to Energy = 0.288928
Thermal correction to Enthalpy = 0.289872
Thermal correction to Gibbs Free Energy = 0.220235

C	-0.91796600	3.57528400	0.24816500
C	-0.99863100	2.17961600	0.13982200
C	1.38737500	2.00504500	0.12551700
C	1.51471500	3.39584900	0.22883300
C	0.34921200	4.18485700	0.30634900
H	-1.83259900	4.18433600	0.26912300
H	2.50876000	3.86435100	0.23811800
H	0.43012600	5.27820100	0.38408200
C	-2.17865000	1.34700400	-0.01794300
C	-3.50934700	1.80261400	0.04113000
C	-2.91465300	-0.82743800	-0.55983300
C	-4.55883000	0.92129500	-0.21580500
H	-3.70465600	2.85662100	0.28491600
C	-4.24722700	-0.41777000	-0.53738600
H	-2.61581400	-1.86163800	-0.80835800
H	-5.60223500	1.26629300	-0.17556800
H	-5.03337900	-1.14726100	-0.77770000
C	2.43325300	1.00353600	-0.02151900
C	3.81482900	1.26403600	-0.00015000
C	4.72030400	0.22426600	-0.21715000
H	4.16558000	2.28943000	0.18350900
C	2.83379700	-1.28396300	-0.42218400
C	4.21342800	-1.07236100	-0.43624000
H	5.80330100	0.41569600	-0.21113000
H	2.39265100	-2.29207600	-0.51167700
H	4.88140700	-1.92709700	-0.61239300
N	0.14684600	1.43074700	0.12623000
N	1.94494800	-0.28008600	-0.22559500
N	-1.88338200	0.01512700	-0.29027900
Ni	0.00943900	-0.42033600	-0.12589000
S	-0.22607000	-2.59846200	0.16856500
O	1.06117200	-3.35401800	0.41864400
O	-1.15251900	-3.21415300	-0.85798000

C	-1.10554400	-2.76252800	1.77337500
H	-2.08060600	-2.24403400	1.71965900
H	-1.23892500	-3.85277500	1.91047700
H	-0.46528500	-2.33645600	2.56711700

N2:

Eel = -3147.381348
Zero-point correction = 0.376078
Thermal correction to Energy = 0.404759
Thermal correction to Enthalpy = 0.405704
Thermal correction to Gibbs Free Energy = 0.316782

C	1.17090800	3.11071000	1.01478600
C	0.82795800	1.74948100	0.95099000
C	-1.39452700	2.28004300	0.33091800
C	-1.10874200	3.65578200	0.36560000
C	0.19061500	4.06861200	0.70504600
H	2.18321400	3.42094800	1.30753700
H	-1.88722400	4.39763100	0.13947500
H	0.43654400	5.13979800	0.73908600
C	1.69377500	0.60457600	1.29468700
C	3.02886000	0.72166400	1.71912900
C	1.74105000	-1.71263000	1.59118100
C	3.73240000	-0.43157500	2.08064800
H	3.51053500	1.70791400	1.76085600
C	3.07104600	-1.66922500	2.02980600
H	1.14575600	-2.64302100	1.57760600
H	4.78059900	-0.36398000	2.40727700
H	3.57277400	-2.59910300	2.33415800
C	-2.70268200	1.65572700	0.07062100
C	-3.90296300	2.37411700	-0.08931500
C	-5.09541100	1.67460800	-0.29819300
H	-3.89946300	3.47206200	-0.03811500
C	-3.83031500	-0.38481000	-0.17068300
C	-5.05773200	0.27040200	-0.33418100
H	-6.04369700	2.21793000	-0.42513600
H	-3.71874300	-1.48749500	-0.21096900
H	-5.97024100	-0.32279200	-0.49005200
N	-0.42221200	1.37123600	0.59368900
N	-2.67757700	0.28868200	0.02350700
N	1.07224500	-0.60881800	1.20568600
Ni	-0.73707600	-0.51557600	0.09670800
C	0.54315200	-0.27252700	-1.91651200

C	1.90477200	0.09816600	-1.82957500
C	2.92341700	-0.88640900	-1.67101500
C	2.28418700	1.47136300	-1.83081900
C	4.25568400	-0.50566100	-1.50624200
H	2.63925900	-1.94819800	-1.66767100
C	3.62535500	1.83703800	-1.67177100
H	1.50579300	2.23670300	-1.95845300
C	4.61596800	0.85592800	-1.49962500
H	5.02775000	-1.27889400	-1.37438300
H	3.90013500	2.90319100	-1.67705700
H	5.66873000	1.14831700	-1.36933000
C	-0.67924200	-0.58033600	-1.91049900
H	-1.57609900	-0.85848500	-2.46714500
O	-0.92299100	-3.29475400	1.51359900
O	-2.57457600	-3.05478200	-0.45872100
S	-1.21385000	-2.72127500	0.13690400
C	-0.07424500	-3.67317700	-0.96215500
H	-0.23007100	-3.36945500	-2.01250700
H	0.97347500	-3.50130000	-0.65046500
H	-0.36567600	-4.72787300	-0.79913900

N2-3TS:

Eel = -3147.364130
Zero-point correction = 0.374591
Thermal correction to Energy = 0.402904
Thermal correction to Enthalpy = 0.403848
Thermal correction to Gibbs Free Energy = 0.314671

C	-2.77750500	0.53299200	-2.02674900
C	-1.67909600	-0.12589200	-1.44795900
C	-0.49968800	1.92213300	-1.23476200
C	-1.56332200	2.63099600	-1.81334400
C	-2.70852200	1.92104700	-2.21961100
H	-3.68083700	-0.02438800	-2.31052500
H	-1.51229000	3.72228700	-1.93140200
H	-3.55692000	2.45669500	-2.66902000
C	-1.57590700	-1.55477200	-1.11338600
C	-2.56508400	-2.52010000	-1.38984100
C	-0.23183100	-3.17162400	-0.07519200
C	-2.36438800	-3.84209500	-0.98615600
H	-3.48501200	-2.22824000	-1.91533600
C	-1.17715700	-4.17615600	-0.30521300
H	0.72542000	-3.36394300	0.43710700

H	-3.12512100	-4.60851100	-1.19611400
H	-0.98239500	-5.20225500	0.03752700
C	0.75379600	2.48411100	-0.69082800
C	1.14623800	3.83034200	-0.80517300
C	2.37496600	4.22606900	-0.26470100
H	0.50165600	4.55295900	-1.32523100
C	2.73411600	1.94208000	0.44859400
C	3.18390300	3.26654700	0.36649000
H	2.70450200	5.27267300	-0.34628200
H	3.33102200	1.12824200	0.90269300
H	4.16339500	3.53360500	0.78811600
N	-0.56994700	0.57507300	-1.09458100
N	1.54274000	1.57057400	-0.05501900
N	-0.41639200	-1.89815500	-0.46872300
Ni	0.71789100	-0.29177300	0.05680300
C	-0.22199800	-0.15031400	1.99368600
C	-1.58859400	0.26493000	1.94619500
C	-2.63343300	-0.69328000	1.87102200
C	-1.92259900	1.64417600	1.88211500
C	-3.96377600	-0.28055000	1.73075100
H	-2.38054200	-1.76240500	1.91602600
C	-3.25492700	2.04329600	1.73860500
H	-1.11562300	2.38931300	1.93459600
C	-4.28181300	1.08573600	1.65866200
H	-4.76197200	-1.03649400	1.67333600
H	-3.49721500	3.11571000	1.68550900
H	-5.32849100	1.40553200	1.54447400
C	0.96639400	-0.54259500	2.08577900
H	1.98638200	-0.80516500	2.40622700
O	2.99472800	-0.71945200	-1.15361900
O	3.75055600	-0.97828700	1.32189000
S	2.97917400	-1.58180200	0.12599900
C	4.09338200	-2.99250800	-0.34166300
H	4.24351300	-3.65120400	0.53441000
H	3.64780500	-3.53619600	-1.19629000
H	5.03898000	-2.49768100	-0.63812700

N3:

Eel = -3147.381226

Zero-point correction = 0.375446

Thermal correction to Energy = 0.404258

Thermal correction to Enthalpy = 0.405202

Thermal correction to Gibbs Free Energy = 0.314909

C	2.96181300	-0.34164900	-1.85682400
C	1.78483400	0.24906600	-1.36970700
C	0.72526900	-1.86518400	-1.21718200
C	1.87184500	-2.50785800	-1.70954200
C	2.99828500	-1.73358300	-2.03611600
H	3.84540200	0.27199700	-2.08022400
H	1.89584600	-3.60112800	-1.81949700
H	3.91087400	-2.21770800	-2.41200400
C	1.56952900	1.68028700	-1.08647600
C	2.49171700	2.70831600	-1.36490200
C	0.04275900	3.22382700	-0.20201900
C	2.15981700	4.02681500	-1.03909500
H	3.45731400	2.47313700	-1.83418800
C	0.90997900	4.29413800	-0.44856100
H	-0.95172800	3.36351300	0.25446900
H	2.86672200	4.84384900	-1.24668800
H	0.61012600	5.31839900	-0.18453100
C	-0.52590900	-2.51226500	-0.77063500
C	-0.82730000	-3.87972100	-0.91253700
C	-2.06250700	-4.35062300	-0.45091300
H	-0.10905000	-4.56190600	-1.38930500
C	-2.60489500	-2.09754300	0.24564500
C	-2.96778700	-3.44676400	0.13207200
H	-2.32083600	-5.41538800	-0.55274100
H	-3.26802900	-1.32267900	0.68192400
H	-3.95000700	-3.77966600	0.49747700
N	0.69670900	-0.51306000	-1.07919200
N	-1.41058000	-1.65560700	-0.18759700
N	0.36566900	1.95499600	-0.50517000
Ni	-0.73435700	0.28031800	-0.01869400
C	0.19890200	0.12992600	1.89998600
C	1.58551600	-0.21557700	1.91412200
C	1.99428500	-1.57581300	1.87944900
C	2.58349700	0.79404800	1.87823100
C	3.35089500	-1.90604300	1.80263800
H	1.22586800	-2.36196800	1.90491300
C	3.93867800	0.45025800	1.80479300
H	2.27432000	1.84876800	1.90482700
C	4.33032300	-0.89790900	1.76016700
H	3.65011800	-2.96502500	1.77268300
H	4.69847500	1.24659800	1.77882700
H	5.39630000	-1.16395800	1.69840500
C	-1.00815200	0.46294100	2.01656200

H	-1.99734500	0.64663500	2.44628900
O	-2.48333300	1.00216300	-0.67026200
O	-3.94924500	0.60827900	1.42502800
S	-3.43656300	1.66412600	0.43187100
C	-4.88836900	1.92078700	-0.67199200
H	-5.12110000	0.92013200	-1.08423000
H	-5.72733000	2.30561300	-0.06160900
H	-4.61904700	2.62875800	-1.47872600

N3':

Eel = -3147.373645
Zero-point correction = 0.375509
Thermal correction to Energy = 0.404391
Thermal correction to Enthalpy = 0.405335
Thermal correction to Gibbs Free Energy = 0.314717

C	-2.60743400	3.50306900	-0.00358300
C	-1.57855500	2.56046300	0.14875800
C	-3.08035700	0.77814600	-0.29308400
C	-4.14757300	1.67859400	-0.44775100
C	-3.90534600	3.05425900	-0.30029600
H	-2.40186400	4.57645100	0.11183800
H	-5.15777200	1.31406200	-0.68015700
H	-4.72651500	3.77571400	-0.41771900
C	-0.17014300	2.84474300	0.47668100
C	0.37772900	4.12784900	0.66451800
C	1.89529700	1.83436400	0.93841800
C	1.73412600	4.24459400	0.98990700
H	-0.25105300	5.02312700	0.55746500
C	2.50699000	3.07846400	1.13517400
H	2.42055300	0.87398500	1.05957000
H	2.18467800	5.23770500	1.13548800
H	3.57312000	3.12728700	1.39912900
C	-3.14211200	-0.68691500	-0.43755300
C	-4.30544800	-1.39607200	-0.79368800
C	-4.24039900	-2.78477300	-0.94287900
H	-5.25068500	-0.85976900	-0.95741800
C	-1.88906200	-2.67017500	-0.37448100
C	-3.00874900	-3.42942100	-0.73796600
H	-5.13873400	-3.35635500	-1.22033500
H	-0.89407800	-3.12321800	-0.16785200
H	-2.90643400	-4.51813500	-0.85328500
N	-1.83257500	1.22880200	0.00634700

N	-1.95403700	-1.32921000	-0.21754200
N	0.59710800	1.72522800	0.60447100
Ni	-0.35422800	-0.02496000	0.07056400
C	1.25615400	-0.31669900	-1.77539700
C	2.63989600	-0.44789900	-1.50423900
C	3.16669900	-1.64741400	-0.94991700
C	3.51693500	0.64672400	-1.74662900
C	4.53083200	-1.73296400	-0.65266500
H	2.48345600	-2.48161800	-0.72480300
C	4.87870300	0.54008600	-1.44642300
H	3.10610700	1.57570300	-2.16829900
C	5.39206700	-0.64860000	-0.89763900
H	4.92698600	-2.66146200	-0.21357700
H	5.54754000	1.39293100	-1.64022200
H	6.46371300	-0.72822200	-0.65916200
C	0.02768900	-0.12193900	-1.94372100
H	-0.82576700	-0.06433700	-2.61941500
O	0.96494600	-0.92912200	1.28944200
O	0.86625700	-3.41587500	0.57146000
S	1.17327500	-2.44242200	1.72277900
C	-0.28872100	-2.65432200	2.84694800
H	-0.13344300	-2.02148500	3.74189000
H	-1.18793300	-2.32100300	2.29458200
H	-0.36431400	-3.72245300	3.12691200

N3-4ZTS:

Eel = -3147.351487
Zero-point correction = 0.374564
Thermal correction to Energy = 0.402914
Thermal correction to Enthalpy = 0.403858
Thermal correction to Gibbs Free Energy = 0.314646

C	-3.40138400	-0.34724400	-1.53008400
C	-2.08815700	-0.67596700	-1.15919900
C	-1.46153400	1.60959200	-1.14883300
C	-2.75837200	1.99743400	-1.52103800
C	-3.73410200	1.00485400	-1.71238100
H	-4.15583000	-1.13286200	-1.67552500
H	-3.00921600	3.05852300	-1.65665700
H	-4.75563200	1.28617000	-2.00594300
C	-1.54509600	-2.02056600	-0.91151200
C	-2.30321000	-3.20573100	-0.86676300
C	0.42470100	-3.20725200	-0.45902500

C	-1.65921400	-4.41948200	-0.60863200
H	-3.39106200	-3.16918600	-1.01996700
C	-0.26524900	-4.42110100	-0.40152200
H	1.51394600	-3.13004900	-0.31678400
H	-2.23442700	-5.35598500	-0.56372400
H	0.28093800	-5.35454800	-0.20340100
C	-0.31390200	2.49361100	-0.88125100
C	-0.38578600	3.89859600	-0.85828700
C	0.76716900	4.63733800	-0.57561500
H	-1.34278400	4.40375500	-1.05037400
C	1.97091200	2.54815300	-0.35723800
C	1.96529000	3.94781600	-0.31972200
H	0.73150900	5.73665400	-0.54974400
H	2.88999000	1.96601300	-0.15608300
H	2.89804900	4.48420000	-0.09399900
N	-1.15753400	0.29814900	-0.98429800
N	0.85997000	1.83209000	-0.63423900
N	-0.19132900	-2.03536500	-0.70748700
Ni	0.63092000	-0.19888100	-0.44934500
C	0.68613400	-0.22545900	1.70542500
C	-0.70757700	0.07720600	1.98970800
C	-1.66542700	-0.95988400	2.12133700
C	-1.14199400	1.41990200	2.13133500
C	-3.01246700	-0.65820400	2.35798900
H	-1.33629400	-2.00522400	2.03300900
C	-2.49189700	1.70920400	2.36258300
H	-0.40346500	2.23032800	2.05071400
C	-3.43905300	0.67584500	2.46709200
H	-3.74019000	-1.47923000	2.45459600
H	-2.80919200	2.75902200	2.46293200
H	-4.49994300	0.90879400	2.64268800
C	1.83205500	-0.43576200	2.19900000
H	2.57557900	-0.52351400	2.98420700
O	2.48334800	-0.87665600	-0.79557500
O	4.36974300	0.57164200	0.23740300
S	3.54708100	-0.69754300	0.34595100
C	4.74027100	-2.01632800	-0.15294900
H	5.60562100	-1.97074000	0.53403900
H	4.24551000	-3.00507500	-0.11217300
H	5.04268800	-1.76111900	-1.18632900

N3’-4’TS:

Eel = -3147.346381

Zero-point correction = 0.374839
Thermal correction to Energy = 0.402902
Thermal correction to Enthalpy = 0.403846
Thermal correction to Gibbs Free Energy = 0.314288

C	-4.32848300	1.47831700	-1.09152000
C	-3.06017000	1.35108700	-0.50253900
C	-3.16666800	-1.01953900	-0.66900700
C	-4.43766700	-0.94783000	-1.26029200
C	-5.01816900	0.31548200	-1.47585900
H	-4.78166000	2.46927400	-1.23621500
H	-4.97736300	-1.86327700	-1.54065800
H	-6.01599400	0.39206400	-1.93109600
C	-2.19591800	2.42782400	-0.00142300
C	-2.43315300	3.80604300	-0.16821300
C	-0.22020500	2.86722800	1.18399600
C	-1.53147000	4.72700200	0.37191500
H	-3.31800400	4.14802800	-0.72398500
C	-0.40224400	4.24643800	1.06662500
H	0.63661100	2.42232500	1.71351800
H	-1.69997100	5.80728700	0.25232500
H	0.32728600	4.93588800	1.51540200
C	-2.40465400	-2.23391100	-0.33788600
C	-2.79647500	-3.54129100	-0.68320700
C	-1.99152600	-4.61897100	-0.30371400
H	-3.72401800	-3.70610400	-1.24973800
C	-0.46344300	-3.03710600	0.70772700
C	-0.80367500	-4.35956900	0.40469400
H	-2.28117700	-5.64833800	-0.56201100
H	0.47219300	-2.78700200	1.23873700
H	-0.13753800	-5.17417700	0.72304800
N	-2.50626000	0.11904000	-0.32272800
N	-1.24432400	-1.99346800	0.35338500
N	-1.08810100	1.97266100	0.66942800
Ni	-0.75058100	-0.01119800	0.46280300
C	1.88367800	0.03503400	-0.84109400
C	3.26399100	0.12141700	-1.21838000
C	4.19236000	-0.90416800	-0.88956400
C	3.72530300	1.24509500	-1.95489600
C	5.52580800	-0.80650700	-1.29944500
H	3.84530800	-1.75761000	-0.28789900
C	5.06265500	1.32905200	-2.36170200
H	3.01242700	2.04603400	-2.20079100
C	5.96892700	0.30545600	-2.03852000

H	6.23232400	-1.60963100	-1.03846100
H	5.40269700	2.20577100	-2.93455400
H	7.02004800	0.37572900	-2.35713200
C	0.60924700	0.05302100	-1.01090600
H	0.05375100	0.07312500	-1.96363100
O	0.60999100	-0.08090600	1.97373400
O	2.39131900	-1.94138200	1.77557100
S	2.06016200	-0.47718200	1.54997400
C	3.04850700	0.40245000	2.82766800
H	2.71279300	-0.00122900	3.80172900
H	4.11380300	0.16862600	2.64704300
H	2.86115200	1.49002500	2.75452000

N4Z:

Eel = -3147.393563

Zero-point correction = 0.378603

Thermal correction to Energy = 0.405998

Thermal correction to Enthalpy = 0.406943

Thermal correction to Gibbs Free Energy = 0.318165

C	4.22867900	0.06116500	-0.90698800
C	2.89990800	-0.32378200	-0.69157600
C	2.27839700	1.94908900	-0.21080800
C	3.59852800	2.37342600	-0.41604000
C	4.57665900	1.42158700	-0.76531200
H	4.98786600	-0.68487200	-1.18072000
H	3.86507300	3.43415100	-0.30620700
H	5.61591500	1.73996300	-0.92878100
C	2.29507700	-1.64108000	-0.77291600
C	2.96807900	-2.83636800	-1.08473800
C	0.26770200	-2.82288400	-0.51465500
C	2.27221200	-4.04421600	-1.11285900
H	4.04614900	-2.80065200	-1.29725100
C	0.89050100	-4.03099200	-0.81521800
H	-0.79866300	-2.78304500	-0.25847400
H	2.78993600	-4.98409600	-1.35235500
H	0.29655500	-4.95520300	-0.80528300
C	1.10344600	2.71822800	0.14330700
C	1.06214100	4.10603100	0.37798600
C	-0.13881200	4.72497800	0.71818500
H	1.99119300	4.68756500	0.29060800
C	-1.21378600	2.55792400	0.57798400
C	-1.30361500	3.92518200	0.81953200

H	-0.17900500	5.80788900	0.90352700
H	-2.09745500	1.90952400	0.64753700
H	-2.27734500	4.36154900	1.08222500
N	1.97112100	0.62089400	-0.35140500
N	-0.05322700	1.93855300	0.24401700
N	0.92713300	-1.63645900	-0.49709100
Ni	0.22535800	0.08633400	-0.06391000
C	-1.59202400	-0.43226900	0.09830500
C	-2.46504200	-0.22888700	-1.07780400
C	-3.85155800	0.03588900	-0.94954700
C	-1.91473200	-0.26688400	-2.38237700
C	-4.65471100	0.24004700	-2.07888400
H	-4.29561700	0.10096800	0.05592000
C	-2.72123900	-0.08028400	-3.51206400
H	-0.83308400	-0.44642700	-2.48445500
C	-4.09447900	0.17675400	-3.36573400
H	-5.72716300	0.45629900	-1.95498400
H	-2.27245500	-0.12417600	-4.51626500
H	-4.72585100	0.33901600	-4.25261100
C	-2.07665600	-0.97579600	1.24436700
H	-3.08339900	-1.41108700	1.38725500
O	-0.90772800	-2.79296700	2.78153500
O	0.26797800	-0.51968000	2.46961100
S	-0.98343300	-1.31825000	2.60838700
C	-1.85774600	-0.65778400	4.05201700
H	-2.83621000	-1.16346700	4.14948600
H	-1.21220700	-0.89917600	4.91733000
H	-1.96809900	0.43574300	3.93600900

N4’:

Eel = -3147.387067
Zero-point correction = 0.378889
Thermal correction to Energy = 0.406060
Thermal correction to Enthalpy = 0.407004
Thermal correction to Gibbs Free Energy = 0.319371

C	-4.69739200	1.20013200	0.12054800
C	-3.30611900	1.21051700	-0.05569800
C	-3.27642800	-1.18954700	-0.20130000
C	-4.66438900	-1.24113000	-0.02958000
C	-5.37704900	-0.03279100	0.13218700
H	-5.24805600	2.14278800	0.24913700
H	-5.18959600	-2.20643300	-0.01876900

H	-6.46715300	-0.05536500	0.27101500
C	-2.38743700	2.32764000	-0.10064700
C	-2.72669000	3.68810200	0.03627600
C	-0.10400100	2.90278500	-0.31089400
C	-1.73589000	4.66637100	0.00044600
H	-3.78263800	3.96048100	0.17718800
C	-0.38977400	4.25704400	-0.17265900
H	0.92747700	2.54557800	-0.44136500
H	-1.99342100	5.72969500	0.10840200
H	0.43199900	4.98617700	-0.19928100
C	-2.32652900	-2.27525200	-0.37404000
C	-2.63972400	-3.64621300	-0.39608200
C	-1.62458900	-4.59276900	-0.53229300
H	-3.69008300	-3.95488900	-0.29556500
C	-0.03182000	-2.76991900	-0.61973300
C	-0.29089900	-4.13795400	-0.63747400
H	-1.85830800	-5.66700900	-0.54686500
H	0.99420400	-2.38480100	-0.68057300
H	0.55001100	-4.83973500	-0.72427800
N	-2.64113800	0.02138100	-0.21386100
N	-1.00991900	-1.83642400	-0.50535800
N	-1.05705900	1.93642400	-0.28814300
Ni	-0.80521400	0.05968400	-0.43659500
C	2.08958000	-0.00119800	-0.05698900
C	3.52145300	0.09771900	-0.42909200
C	4.50956600	-0.76593500	0.10448200
C	3.92517500	1.08204500	-1.36314000
C	5.84918000	-0.64453900	-0.29124800
H	4.20604400	-1.54098500	0.82348500
C	5.26357900	1.19273100	-1.76100600
H	3.17124100	1.77291400	-1.77087200
C	6.23410200	0.33058300	-1.22501200
H	6.60091400	-1.32925700	0.13096500
H	5.55328800	1.96691600	-2.48813900
H	7.28701900	0.42108100	-1.53221700
C	1.01502000	0.13213600	-0.87450600
H	1.24909700	0.29630300	-1.94897400
O	0.27535600	-0.11635500	1.94787300
O	2.20781000	-1.80671900	1.94218500
S	1.70395300	-0.42958000	1.66850100
C	2.68306800	0.72502100	2.66323300
H	2.48026000	0.43910200	3.71213300
H	3.75522700	0.61095100	2.42202700
H	2.32474200	1.75006300	2.45757500

N4Z-ETS:

Eel = -3147.353850
Zero-point correction = 0.377382
Thermal correction to Energy = 0.404410
Thermal correction to Enthalpy = 0.405355
Thermal correction to Gibbs Free Energy = 0.318309

C	4.15274300	0.56392500	-0.89162800
C	2.86828500	0.02426300	-0.72531500
C	1.97210200	2.17972800	-0.26224200
C	3.23472000	2.77131100	-0.42204700
C	4.33607600	1.94687800	-0.71480200
H	4.99469600	-0.08026200	-1.18131200
H	3.35599200	3.86068300	-0.34318200
H	5.33361800	2.38976200	-0.84740800
C	2.41732800	-1.34117800	-0.98808800
C	3.25211200	-2.44731800	-1.22171200
C	0.50130800	-2.66652700	-1.30571100
C	2.68463600	-3.68681000	-1.52296100
H	4.34269000	-2.32670900	-1.15420000
C	1.28027200	-3.78942100	-1.57704800
H	-0.59675700	-2.70617700	-1.30044300
H	3.32170500	-4.56412800	-1.70618800
H	0.78702000	-4.74129700	-1.81977300
C	0.68425600	2.84552000	-0.05336600
C	0.52785900	4.19589500	0.31029500
C	-0.75596100	4.72774100	0.45013700
H	1.41720400	4.81542900	0.49372400
C	-1.63840400	2.54534300	-0.10408200
C	-1.86128900	3.88151800	0.23184100
H	-0.89793700	5.78128100	0.73174100
H	-2.47628200	1.85238900	-0.27332300
H	-2.89231000	4.25389000	0.31534400
N	1.83489700	0.83004800	-0.34933800
N	-0.40112800	2.01853400	-0.25417600
N	1.04055900	-1.45664600	-1.02031900
Ni	0.11313800	0.04409700	-0.27777900
C	-1.53341400	-0.70166100	0.20396300
C	-2.81485800	-0.63610100	-0.43006000
C	-4.02203000	-0.88163700	0.29322800
C	-2.92425100	-0.34337600	-1.82383100
C	-5.26422900	-0.81655800	-0.34260100

H	-3.95287900	-1.12971000	1.36365600
C	-4.17174100	-0.27010800	-2.44701900
H	-1.99447400	-0.16595200	-2.38725700
C	-5.35137600	-0.50719200	-1.71411600
H	-6.18237500	-1.00894800	0.23460100
H	-4.23179100	-0.03365100	-3.52088700
H	-6.33221100	-0.46077100	-2.21074500
C	-1.14111300	-0.42162800	1.50146900
H	-1.67755200	0.27092200	2.18554300
O	0.79006600	-2.35069200	1.66750700
O	0.97097500	-0.23022100	3.12672600
S	0.14367400	-1.25695000	2.43985300
C	-0.81224700	-2.08637200	3.75017700
H	-0.05851800	-2.57918200	4.39293600
H	-1.37756500	-1.33230500	4.32790100
H	-1.48222900	-2.83242300	3.28553800

N4E:

Eel = -3147.387810
Zero-point correction = 0.379086
Thermal correction to Energy = 0.406398
Thermal correction to Enthalpy = 0.407342
Thermal correction to Gibbs Free Energy = 0.319216

C	4.38269900	1.70086600	0.15860400
C	2.99961600	1.49373700	0.08703500
C	3.33059700	-0.86792300	-0.23118600
C	4.72105900	-0.70160900	-0.16660100
C	5.24565400	0.59139300	0.02929700
H	4.78953800	2.71006300	0.31296300
H	5.39131400	-1.56705900	-0.26641600
H	6.33378900	0.73708600	0.08250400
C	1.91304000	2.45422500	0.18971100
C	2.06249300	3.83839700	0.39082800
C	-0.43817800	2.69124000	0.14647200
C	0.93804000	4.65922800	0.47326600
H	3.07494500	4.25746400	0.48014300
C	-0.33807800	4.06538100	0.34750700
H	-1.42043100	2.21146100	0.03301400
H	1.04485900	5.74211600	0.63047200
H	-1.25916900	4.66198000	0.40031300
C	2.54792500	-2.07396300	-0.42074500
C	3.06035400	-3.37426200	-0.58969700

C	2.19447300	-4.45238300	-0.76319200
H	4.14983200	-3.52203700	-0.58133300
C	0.33973900	-2.90301900	-0.59430300
C	0.80020100	-4.20482400	-0.76349100
H	2.58700000	-5.47073400	-0.89531400
H	-0.73420700	-2.67460900	-0.58677800
H	0.07373600	-5.01906400	-0.89246600
N	2.51853200	0.22846100	-0.10511300
N	1.16847900	-1.84059200	-0.42528300
N	0.64798600	1.87613700	0.06679800
Ni	0.68464800	-0.02388800	-0.18618100
C	-1.19046200	-0.22561000	-0.22401700
C	-1.80836800	-0.62464300	1.05121000
C	-2.75537200	-1.67648800	1.09711200
C	-1.40817700	-0.03745100	2.27641800
C	-3.29826800	-2.10946800	2.31326500
H	-3.05261900	-2.16650100	0.15749200
C	-1.96939700	-0.45429100	3.48867600
H	-0.65216500	0.76096300	2.26322600
C	-2.91513900	-1.49343700	3.51471800
H	-4.02935600	-2.93251300	2.32306600
H	-1.65803300	0.03016000	4.42672700
H	-3.34496400	-1.82812000	4.47092700
C	-1.83301800	0.07527800	-1.37923000
H	-1.31496900	0.31000900	-2.32346500
O	-4.01661100	1.04433200	-0.13160100
O	-3.54538700	1.62849400	-2.59932100
S	-3.54068000	0.64719400	-1.48303300
C	-4.57817700	-0.73924800	-2.02963700
H	-5.57798100	-0.29593200	-2.19883600
H	-4.16842900	-1.13924300	-2.97508200
H	-4.62047800	-1.50913300	-1.23913100

N4-5ETS:

Eel = -4342.826084

Zero-point correction = 0.539460

Thermal correction to Energy = 0.582218

Thermal correction to Enthalpy = 0.583162

Thermal correction to Gibbs Free Energy = 0.459867

C	-4.76672200	-1.52171700	0.90650200
C	-3.49428600	-0.92622600	0.95297400
C	-2.65472200	-2.47231900	-0.61378900

C	-3.90540800	-3.10683500	-0.70604300
C	-4.96764700	-2.62989500	0.07325000
H	-5.59089900	-1.12510900	1.51389500
H	-4.04747900	-3.96372300	-1.37785600
H	-5.95246000	-3.11635800	0.02555600
C	-3.15800400	0.28724300	1.72115200
C	-4.12620000	1.11004100	2.32800600
C	-1.46200100	1.77199700	2.29495700
C	-3.72359400	2.29894000	2.94283500
H	-5.18921900	0.83717100	2.28745900
C	-2.36491900	2.64566100	2.91304000
H	-0.39827100	2.03089500	2.21081600
H	-4.46489500	2.96121000	3.41358100
H	-1.99936700	3.58644800	3.34769100
C	-1.46203000	-2.82902200	-1.40384000
C	-1.46259600	-3.77248500	-2.44746600
C	-0.27443000	-4.01509500	-3.14689700
H	-2.38555500	-4.30533100	-2.71444100
C	0.80679000	-2.36665400	-1.74687100
C	0.87863800	-3.30174200	-2.78871900
H	-0.25262300	-4.75097700	-3.96434800
H	1.68034700	-1.77809700	-1.41422000
H	1.84010900	-3.45905500	-3.29772400
N	-2.46618100	-1.42095100	0.22195400
N	-0.33484700	-2.14260900	-1.06765300
N	-1.83677100	0.60672900	1.74120700
Ni	-0.63958500	-0.67210700	0.43375200
C	-0.50389600	0.90347700	-0.76016300
C	0.65377700	1.57897600	-0.84486800
H	1.60789300	1.21536500	-0.43293700
O	-0.02967700	3.47392700	-2.69831900
O	0.61576100	4.15367400	-0.29940300
S	0.78856900	3.25390000	-1.48114800
C	2.53539700	3.34040400	-1.95661400
H	2.71837200	2.62380300	-2.77773600
H	2.68912800	4.38160500	-2.29511200
H	3.16686100	3.10970200	-1.08002900
C	0.98731100	-0.60172200	1.39389800
C	1.62120900	0.45713700	2.22676500
C	0.75394200	-1.88775900	1.80414000
C	1.33147600	0.18386000	3.72153900
H	1.33159200	1.47923900	1.91216300
H	2.71590800	0.37769800	2.07407200
C	0.81198200	-2.25812000	3.28037100

H	0.61946600	-2.70523400	1.08004400
C	1.67772500	-1.26746700	4.06895400
H	0.26268300	0.38615900	3.95422000
H	1.93256200	0.88887100	4.33233900
H	1.21223600	-3.28903400	3.37816400
H	-0.22239900	-2.29093000	3.69387100
H	2.74232400	-1.44691000	3.81041800
H	1.56229900	-1.44598000	5.15830200
O	4.21071700	-1.26259900	1.57269900
S	3.98213400	-1.08887400	0.11409100
O	2.58254400	-0.57246000	-0.23958800
O	4.44447200	-2.15269100	-0.80492300
C	4.99930700	0.43927500	-0.33253100
F	6.29582100	0.25957900	-0.05120600
F	4.55364200	1.51089100	0.37339300
F	4.87489100	0.73055900	-1.64084300
C	-1.81288100	1.33445000	-1.27738100
C	-2.52395800	0.53947400	-2.20758300
C	-2.42427400	2.52232300	-0.80513400
C	-3.80671500	0.90402200	-2.63293100
H	-2.04731300	-0.36821500	-2.60532000
C	-3.71450000	2.87507700	-1.22103100
H	-1.85834100	3.17885000	-0.12849200
C	-4.41635200	2.06608900	-2.12907900
H	-4.33685600	0.27693500	-3.36657100
H	-4.17011200	3.80300900	-0.84195800
H	-5.42852700	2.34799700	-2.45704100

N4-5ZTS:

Eel = -4342.813475
Zero-point correction = 0.538576
Thermal correction to Energy = 0.581106
Thermal correction to Enthalpy = 0.582050
Thermal correction to Gibbs Free Energy = 0.461001

C	-3.16640600	2.19936200	-2.48277400
C	-2.60051300	1.13051100	-1.76649500
C	-0.99722900	2.56148600	-0.77412600
C	-1.52448400	3.67092100	-1.45905100
C	-2.61604000	3.47945900	-2.31844100
H	-4.01927600	2.04021700	-3.15631300
H	-1.08916700	4.66996900	-1.32322500
H	-3.04026900	4.33384900	-2.86549800

C	-3.05409600	-0.27967300	-1.79251500
C	-4.19275700	-0.73716500	-2.48221400
C	-2.63350500	-2.42956200	-0.95017600
C	-4.53857600	-2.09055600	-2.39721400
H	-4.80666300	-0.03840300	-3.06712600
C	-3.75208600	-2.95175800	-1.61441100
H	-2.00270900	-3.03924900	-0.27763000
H	-5.42452900	-2.46895200	-2.92854900
H	-4.00116300	-4.01710700	-1.50834800
C	0.14113300	2.57413300	0.17063700
C	0.86357700	3.71847800	0.54454600
C	1.91133900	3.57987800	1.46393200
H	0.61566700	4.70143600	0.12081500
C	1.43197400	1.21641500	1.58515700
C	2.19644900	2.31744100	1.99912600
H	2.50582700	4.45760400	1.75742700
H	1.61225600	0.19429000	1.94920100
H	3.02203500	2.16783500	2.70752600
N	-1.54883800	1.33878800	-0.94754400
N	0.44988500	1.35613100	0.68434300
N	-2.29223600	-1.13402500	-1.05888900
Ni	-0.64367500	-0.21823700	-0.12158300
C	-1.58365200	-0.41458500	1.64497500
C	-1.48150600	-1.34163600	2.61287300
H	-2.09691000	-1.36177000	3.53325200
O	-0.92494700	-3.72043300	1.50179100
O	1.04236400	-2.29979900	2.35243700
S	-0.37167700	-2.73374300	2.48632500
C	-0.53547300	-3.51582500	4.11147500
H	-1.58476900	-3.82537300	4.26890300
H	0.12864100	-4.39817100	4.06415900
H	-0.19060800	-2.81075800	4.88964600
C	0.73496900	-1.39923000	-0.65877700
C	1.11856000	-2.81131400	-0.77701300
C	0.76138400	-0.44559500	-1.65382700
C	0.93686800	-3.28393900	-2.25126600
H	0.60088500	-3.47141000	-0.05943600
H	2.20064200	-2.79977900	-0.51285700
C	0.58109500	-0.92377400	-3.09514000
H	1.27871100	0.52222600	-1.50932300
C	1.36877500	-2.23181200	-3.27331400
H	-0.13349000	-3.54585000	-2.40671500
H	1.51357800	-4.22232200	-2.38214300
H	0.95107400	-0.13812900	-3.78263900

H	-0.49649600	-1.07986800	-3.32647600
H	2.44627100	-1.99856900	-3.13855000
H	1.23059000	-2.62184600	-4.30426900
O	4.09055800	-0.67316200	-1.97530500
S	3.70301500	0.09785700	-0.76401000
O	3.03110800	-0.71855700	0.31909200
O	3.06348500	1.43553000	-1.01049300
C	5.34020800	0.56122100	0.04799600
F	6.07470600	1.33213300	-0.77204500
F	6.05026800	-0.53602200	0.35602200
F	5.12047900	1.25450700	1.19014300
C	-2.58344800	0.66214500	1.78298200
C	-2.23931200	1.92427900	2.32681200
C	-3.90744400	0.47900600	1.31155200
C	-3.17353200	2.96836100	2.36429300
H	-1.22611300	2.07648800	2.72534700
C	-4.83654200	1.52789500	1.34888500
H	-4.20143200	-0.50461000	0.91629200
C	-4.47288700	2.78311300	1.86347900
H	-2.88210600	3.93951700	2.79402700
H	-5.85938900	1.36003600	0.97656100
H	-5.20193300	3.60697200	1.88882600

N5E:

Eel = -3381.164492
Zero-point correction = 0.513261
Thermal correction to Energy = 0.547473
Thermal correction to Enthalpy = 0.548417
Thermal correction to Gibbs Free Energy = 0.445641

C	3.71339200	-1.96929300	-1.68193800
C	2.40758600	-1.57749500	-1.33820500
C	3.19609200	0.48473800	-0.48162800
C	4.52273800	0.14741700	-0.80001200
C	4.77469500	-1.09701700	-1.39725800
H	3.90170700	-2.93347800	-2.17251600
H	5.34569600	0.84660800	-0.60083200
H	5.80399300	-1.38210200	-1.65834800
C	1.16622400	-2.33771700	-1.58541100
C	1.13098400	-3.62790000	-2.13862000
C	-1.17049800	-2.28668700	-1.42393700
C	-0.10664000	-4.25223400	-2.34194100
H	2.06477900	-4.14203900	-2.40412400

C	-1.27555700	-3.56874500	-1.98036400
H	-2.07838900	-1.75108400	-1.10259200
H	-0.15417500	-5.26241600	-2.77423900
H	-2.27295300	-4.01040800	-2.11472600
C	2.72407500	1.76117500	0.08929700
C	3.57391500	2.78880400	0.53083300
C	3.01434900	3.97455300	1.02200200
H	4.66427900	2.66034000	0.49385800
C	0.83265600	3.03060800	0.60420100
C	1.61796200	4.10045100	1.05262200
H	3.66167600	4.79062600	1.37466300
H	-0.26596400	3.08893500	0.60148700
H	1.13242700	5.01641900	1.41747200
N	2.19464000	-0.38676100	-0.73373300
N	1.36539300	1.88876700	0.13705200
N	0.01899000	-1.68196900	-1.23358700
Ni	0.39001600	0.07329900	-0.17005300
C	-1.44714100	0.42697800	0.17234200
C	-2.29306500	-0.21994000	0.99433800
H	-1.96461900	-0.92379000	1.77493000
O	-4.58524400	0.82122700	-0.03460600
O	-4.12579800	-1.71316800	-0.11084300
S	-4.04217100	-0.40152600	0.59633700
C	-4.84149100	-0.61457500	2.20090400
H	-4.73527300	0.31897500	2.78274200
H	-5.90475700	-0.80982700	1.96569500
H	-4.39637800	-1.48275800	2.72022000
C	0.60702400	-0.60370100	1.65652200
C	0.57631200	-2.10398600	1.76717500
C	0.83111700	0.21791200	2.69838600
C	0.52240400	-2.56704800	3.23782700
H	1.47025900	-2.53682600	1.26151200
H	-0.29624700	-2.51977000	1.22002900
C	1.16705100	-0.28961400	4.08772800
H	0.77546000	1.31216100	2.58620000
C	1.51154100	-1.78124100	4.09907500
H	0.71752600	-3.65779200	3.28918700
H	-0.50794200	-2.41064700	3.62728200
H	2.00247800	0.31213700	4.50635400
H	0.29880300	-0.08622400	4.75752400
H	1.52176600	-2.16639100	5.13883900
H	2.53914400	-1.92706900	3.69887400
C	-1.71630300	1.44038500	-0.85160400
C	-2.35177700	2.66273500	-0.50163600

C	-1.20733000	1.29568300	-2.17148600
C	-2.46630300	3.69551600	-1.43527200
H	-2.77628100	2.76826400	0.50642600
C	-1.31624700	2.34399900	-3.09783800
H	-0.76701500	0.33851200	-2.48722900
C	-1.93916000	3.54555700	-2.73220300
H	-2.98156400	4.62665900	-1.15567500
H	-0.92780800	2.21133200	-4.11847700
H	-2.03281400	4.36305700	-3.46237100

N5Z:

Eel = -3381.161950
Zero-point correction = 0.513672
Thermal correction to Energy = 0.547565
Thermal correction to Enthalpy = 0.548509
Thermal correction to Gibbs Free Energy = 0.448361

C	-1.83003200	-3.99246700	-0.10815800
C	-1.66949500	-2.59878100	-0.19789800
C	0.53826900	-2.84929600	-1.00507900
C	0.44124900	-4.24995400	-0.93577900
C	-0.75255700	-4.81677500	-0.46579100
H	-2.78044800	-4.43126000	0.22303300
H	1.27555100	-4.88936700	-1.25336000
H	-0.85212100	-5.90976300	-0.39930600
C	-2.70137900	-1.57528600	0.06199500
C	-3.98611700	-1.87059600	0.54706600
C	-3.21949600	0.69196100	-0.09462200
C	-4.91162000	-0.83176200	0.70702700
H	-4.26005500	-2.90442500	0.79747500
C	-4.52393200	0.47141100	0.36941600
H	-2.87651400	1.70344200	-0.35161400
H	-5.92214500	-1.04025400	1.08761400
H	-5.21575000	1.31995800	0.46452800
C	1.67092000	-2.06824300	-1.53736800
C	2.86681600	-2.62918900	-2.01056000
C	3.83287500	-1.79278700	-2.58428000
H	3.03731000	-3.71191800	-1.93886200
C	2.38469700	0.07873300	-2.12492300
C	3.57825100	-0.41791100	-2.66164900
H	4.77337300	-2.21389900	-2.96828000
H	2.16041100	1.15509200	-2.12252000
H	4.30079400	0.27394700	-3.11627000

N	-0.49408400	-2.07650100	-0.60189400
N	1.44984200	-0.71998200	-1.58233700
N	-2.32398900	-0.29919800	-0.24000400
Ni	-0.17859400	-0.15713100	-0.46156300
C	0.31152400	1.68292000	-0.32687400
C	1.38750600	2.31945100	0.19131800
H	1.51289600	3.41058700	0.06192400
O	2.45916200	1.73623300	2.59562900
O	3.20156700	0.33720500	0.56274100
S	2.74911400	1.63252400	1.14305800
C	4.05555300	2.83410400	0.79489100
H	3.75480300	3.82683300	1.17665500
H	4.93127700	2.45801000	1.35586200
H	4.26751100	2.85453600	-0.28976200
C	-0.04004400	-0.16791300	1.50096600
C	-0.99492800	0.62407700	2.34694500
C	0.91186300	-0.98579200	1.97235100
C	-0.45213200	0.74918100	3.78688400
H	-1.99208900	0.12981000	2.36104300
H	-1.16482100	1.63371600	1.91873000
C	1.15147500	-1.16912200	3.45776800
H	1.63089200	-1.48221200	1.30176700
C	0.00968000	-0.61172900	4.31209100
H	-1.24187300	1.18266000	4.43460400
H	0.40639100	1.45049500	3.77789100
H	1.33389900	-2.24272300	3.67780700
H	2.10260500	-0.63565100	3.68366900
H	0.33668000	-0.52848000	5.36880400
H	-0.84608500	-1.32324300	4.30335800
C	-0.70174900	2.47080100	-1.05788700
C	-1.31290400	3.60610800	-0.46966600
C	-1.10787600	2.07927800	-2.35900100
C	-2.30694500	4.31695400	-1.15793800
H	-1.00953900	3.91171300	0.54331200
C	-2.10004600	2.79368000	-3.04268500
H	-0.62828400	1.21070200	-2.83853000
C	-2.70706300	3.91032500	-2.44210800
H	-2.77437700	5.19500400	-0.68724800
H	-2.39990300	2.48135800	-4.05415200
H	-3.48798300	4.46862200	-2.97937900

N5-6ETS:

Eel = -3381.165112

Zero-point correction = 0.512980
Thermal correction to Energy = 0.546481
Thermal correction to Enthalpy = 0.547425
Thermal correction to Gibbs Free Energy = 0.446949

C	-3.86777600	2.53284000	-0.54136900
C	-2.55740100	2.03830400	-0.66143000
C	-3.31700800	-0.19148300	-0.45648600
C	-4.64892700	0.24069100	-0.32618800
C	-4.91550100	1.61772700	-0.35826800
H	-4.07212200	3.61050000	-0.59432500
H	-5.46868500	-0.48118700	-0.21402000
H	-5.94883200	1.97944900	-0.25685000
C	-1.33528200	2.82780700	-0.91454500
C	-1.32438900	4.21604200	-1.12019800
C	0.96893800	2.70009600	-1.30217200
C	-0.11623200	4.85315000	-1.43574900
H	-2.25561400	4.79380400	-1.04399500
C	1.04655700	4.07940100	-1.54125700
H	1.88208100	2.08777000	-1.34490800
H	-0.08917500	5.93981600	-1.60353800
H	2.02059100	4.52053300	-1.79541500
C	-2.84800100	-1.59048200	-0.52553700
C	-3.70348200	-2.69937300	-0.42132800
C	-3.17618300	-3.98760400	-0.57670800
H	-4.77519300	-2.55676400	-0.22727200
C	-1.01369700	-2.97071300	-0.92251700
C	-1.80890300	-4.12366100	-0.84660700
H	-3.82835000	-4.86967400	-0.49815500
H	0.06113000	-3.04483200	-1.12884500
H	-1.34674100	-5.10910300	-0.99864200
N	-2.32085700	0.70933300	-0.58789900
N	-1.50875700	-1.73263700	-0.74885300
N	-0.18716100	2.08608100	-0.98308400
Ni	-0.46541800	0.12221600	-0.39393300
C	1.37477600	-0.19725400	0.14440100
C	2.20927400	0.72505200	0.67138800
H	1.86994300	1.63982700	1.18345300
O	4.48852800	-0.46073900	-0.21327600
O	4.05502000	2.05793200	-0.57350300
S	3.96001000	0.83139300	0.27048400
C	4.75584100	1.20704300	1.84750300
H	4.64437700	0.33663100	2.51926000
H	5.82081800	1.37439400	1.59758900

H	4.31303100	2.12461400	2.27612500
C	-0.36797200	-0.14325200	1.54508700
C	-0.56680900	1.15173400	2.28779500
C	-0.43909900	-1.35217800	2.13977200
C	-0.41825300	0.95600800	3.80738300
H	-1.57927800	1.55341800	2.05349300
H	0.13610400	1.93276100	1.92904000
C	-0.78865900	-1.53405900	3.59905500
H	-0.20835300	-2.26859200	1.57231000
C	-1.25614600	-0.23818200	4.27177900
H	-0.71625200	1.88381600	4.33688300
H	0.65291200	0.77720600	4.04636300
H	-1.55570700	-2.33213200	3.70308800
H	0.11338300	-1.93705300	4.11738600
H	-1.21458100	-0.34444800	5.37450800
H	-2.32225600	-0.05104600	4.01567000
C	1.81713800	-1.43101400	-0.52853400
C	1.84385800	-1.48942600	-1.94295600
C	2.20807900	-2.56800000	0.21633800
C	2.24750500	-2.66158300	-2.59502200
H	1.56828700	-0.59804800	-2.52756600
C	2.60491400	-3.73891000	-0.44292200
H	2.22056700	-2.51170600	1.31390200
C	2.62208200	-3.79127700	-1.84725100
H	2.28020200	-2.69154000	-3.69417900
H	2.91956000	-4.61480000	0.14407900
H	2.94330400	-4.70961500	-2.36073200

N5-6ZTS:

Eel = -3381.159116
Zero-point correction = 0.513163
Thermal correction to Energy = 0.546433
Thermal correction to Enthalpy = 0.547378
Thermal correction to Gibbs Free Energy = 0.448714

C	2.59775500	3.61043000	0.45279800
C	2.19961300	2.30678000	0.10815800
C	0.07354400	3.07659200	-0.58029800
C	0.41083500	4.40239800	-0.25718400
C	1.68279600	4.65969300	0.27660700
H	3.60436500	3.81043200	0.84350000
H	-0.30076900	5.22256300	-0.42133700
H	1.96870300	5.68755100	0.54231400

C	3.02957000	1.08564000	0.15748900
C	4.36554600	1.06935900	0.59212400
C	3.11114000	-1.18852400	-0.35154200
C	5.08800300	-0.12883400	0.54030600
H	4.83844900	1.98931600	0.96176300
C	4.45126000	-1.27626100	0.04912600
H	2.57824200	-2.07308000	-0.72486000
H	6.13513700	-0.16266600	0.87471500
H	4.97659100	-2.23877100	-0.02370400
C	-1.18316100	2.60680300	-1.19580300
C	-2.23737200	3.44997900	-1.57841100
C	-3.34579900	2.90344000	-2.23776100
H	-2.18614400	4.52786900	-1.37333900
C	-2.30232900	0.74003300	-2.04127900
C	-3.36971000	1.52665600	-2.49058000
H	-4.17832600	3.54917100	-2.55269100
H	-2.29983700	-0.35113500	-2.17928300
H	-4.21218600	1.05477900	-3.01495000
N	0.95631000	2.07773200	-0.36192700
N	-1.23405600	1.25758200	-1.41211100
N	2.40758300	-0.04326700	-0.29384200
Ni	0.30459300	0.23536600	-0.49403300
C	-0.68578600	-1.44899800	-0.32963300
C	-1.94536700	-1.89037300	-0.06966700
H	-2.28884800	-2.84683000	-0.50342600
O	-2.97643300	-1.50619800	2.39335600
O	-3.43454000	0.26746900	0.58092900
S	-3.21341800	-1.15456700	0.97002500
C	-4.67806900	-2.07237700	0.44380600
H	-4.54620600	-3.14370300	0.68120600
H	-5.50018000	-1.64396300	1.04673000
H	-4.85787000	-1.90376100	-0.63367000
C	0.02827400	-0.44964400	1.35105900
C	0.90549600	-1.43318800	2.08000700
C	-0.74361200	0.46031900	1.98778200
C	0.37697800	-1.64609600	3.50983000
H	1.95007300	-1.05246200	2.12215200
H	0.96760000	-2.39830400	1.53932300
C	-0.79213000	0.59014600	3.48962000
H	-1.45282600	1.08832700	1.42716500
C	0.22590700	-0.29591900	4.21647600
H	1.06713300	-2.31296000	4.06566200
H	-0.61124600	-2.14973500	3.45667200
H	-0.68524400	1.65689100	3.78262000

H	-1.83094000	0.29994100	3.77019200
H	-0.08475500	-0.43717200	5.27136900
H	1.21600500	0.21079400	4.24730100
C	0.15893900	-2.34029500	-1.17398000
C	0.58716400	-1.90833000	-2.45575400
C	0.54194700	-3.62952900	-0.73205900
C	1.40321600	-2.72587300	-3.24946900
H	0.26479200	-0.92511700	-2.83424500
C	1.36245800	-4.44272400	-1.52904500
H	0.18657100	-3.98944400	0.24526100
C	1.80361400	-3.99000900	-2.78343300
H	1.72345400	-2.37698000	-4.24249300
H	1.65304700	-5.44134600	-1.16956400
H	2.44590000	-4.62964100	-3.40672500

N6:

Eel = -2250.426321
Zero-point correction = 0.224738
Thermal correction to Energy = 0.239149
Thermal correction to Enthalpy = 0.240093
Thermal correction to Gibbs Free Energy = 0.182486

C	1.22475200	2.74263400	-0.00005200
C	1.19144100	1.33731700	-0.00004600
C	-1.19144100	1.33731700	-0.00004600
C	-1.22475200	2.74263400	-0.00005300
C	0.00000000	3.43527700	-0.00006100
H	2.17550800	3.29313200	-0.00005100
H	-2.17550800	3.29313200	-0.00005200
H	0.00000000	4.53479200	-0.00007000
C	2.31504200	0.37013000	-0.00001600
C	3.66808400	0.73232800	0.00004200
C	2.88296700	-1.90869100	0.00002200
C	4.65184600	-0.26709500	0.00010300
H	3.95090700	1.79411300	0.00004400
C	4.25146400	-1.61007300	0.00009500
H	2.53129200	-2.95132900	-0.00000500
H	5.71791800	0.00233200	0.00015600
H	4.98565700	-2.42781200	0.00014100
C	-2.31504200	0.37013000	-0.00001600
C	-3.66808400	0.73232800	0.00004300
C	-4.65184600	-0.26709500	0.00010400
H	-3.95090700	1.79411300	0.00004400

C	-2.88296700	-1.90869100	0.00002200
C	-4.25146400	-1.61007300	0.00009500
H	-5.71791800	0.00233200	0.00015600
H	-2.53129200	-2.95132900	-0.00000600
H	-4.98565700	-2.42781200	0.00014100
N	0.00000000	0.70970000	-0.00005300
N	-1.92738100	-0.95390900	-0.00003000
N	1.92738100	-0.95390900	-0.00003000
Ni	0.00000000	-1.17639600	-0.00004000

N7:

Eel = -2838.977612
Zero-point correction = 0.271636
Thermal correction to Energy = 0.291543
Thermal correction to Enthalpy = 0.292487
Thermal correction to Gibbs Free Energy = 0.223635

C	-0.97098100	3.54445900	0.36226900
C	-1.02206700	2.16154200	0.12099800
C	1.34629700	2.02147600	0.10449900
C	1.46188100	3.40155900	0.34056700
C	0.28695600	4.15916100	0.46956500
H	-1.89175800	4.13358900	0.46812600
H	2.44746800	3.87772600	0.43027000
H	0.35211200	5.24014500	0.65987500
C	-2.21249000	1.31158800	-0.05193100
C	-3.52542800	1.79576500	-0.10484300
C	-2.94618700	-0.87424000	-0.48817400
C	-4.57925800	0.90569400	-0.35526800
H	-3.71878000	2.86858100	0.03137800
C	-4.27905900	-0.44484000	-0.56471700
H	-2.65251700	-1.92397600	-0.66540100
H	-5.61603800	1.26953200	-0.40187200
H	-5.06399600	-1.18047600	-0.78982000
C	2.42325200	1.03137700	-0.05902300
C	3.78371700	1.34893800	-0.13964500
C	4.71867100	0.32499200	-0.35219900
H	4.10762600	2.39501400	-0.05163000
C	2.88093800	-1.24895900	-0.36925500
C	4.25596000	-0.98874200	-0.47701600
H	5.79127000	0.55720600	-0.42383300
H	2.46895400	-2.27443600	-0.40340600
H	4.94444000	-1.82771400	-0.65005900

N	0.12386300	1.45458900	-0.00203700
N	1.98054300	-0.26942200	-0.15941300
N	-1.93113000	-0.02666700	-0.21451900
Ni	0.01234600	-0.44439300	-0.10938600
S	-0.18900700	-2.62830400	0.19222200
O	1.12560000	-3.33968500	0.35584700
O	-1.15843100	-3.19355000	-0.80943700
C	-0.97312200	-2.78133800	1.83653700
H	-1.95002300	-2.26496000	1.83458000
H	-1.09887900	-3.87131500	1.98430600
H	-0.28621300	-2.35725600	2.59107100

N4-5ZTS2:

Eel = -4342.777741
Zero-point correction = 0.538083
Thermal correction to Energy = 0.580822
Thermal correction to Enthalpy = 0.581766
Thermal correction to Gibbs Free Energy = 0.460927

C	3.14343400	-1.24622300	-2.86850300
C	1.99692500	-1.22061800	-2.05993400
C	2.04783600	1.14275000	-1.96800300
C	3.18884900	1.18572600	-2.79039200
C	3.75796100	-0.02681800	-3.20533000
H	3.53136700	-2.19728100	-3.25643500
H	3.60942600	2.14691500	-3.11597700
H	4.65667100	-0.02304500	-3.83926000
C	1.08234600	-2.34272700	-1.77717000
C	1.36118300	-3.68365300	-2.09085300
C	-1.08107100	-2.87202600	-1.11122000
C	0.35790400	-4.63991300	-1.91034300
H	2.35820800	-3.96823300	-2.45255300
C	-0.89523800	-4.22190900	-1.43843600
H	-2.05758800	-2.50891000	-0.75138400
H	0.54912800	-5.69842700	-2.14133900
H	-1.72951100	-4.92746300	-1.31690700
C	1.20549600	2.27234200	-1.54761500
C	1.51808600	3.62598200	-1.76065900
C	0.57196500	4.60343400	-1.44003800
H	2.49886000	3.90355900	-2.17012900
C	-0.90831600	2.83210300	-0.72356700
C	-0.67692000	4.19432600	-0.94084900
H	0.79723200	5.66894100	-1.59282500

H	-1.87813700	2.46319000	-0.36291100
H	-1.47360700	4.92066200	-0.72552800
N	1.55001200	-0.04751000	-1.56253500
N	0.01835500	1.88569300	-0.97599200
N	-0.10924600	-1.95418500	-1.24650900
Ni	0.16640400	0.00452800	-0.14786300
C	1.71074700	0.19880900	1.12019500
C	2.43525500	-0.78963700	1.69730900
H	3.34707300	-0.56963100	2.28792200
O	1.02655300	-3.12774600	1.28155000
O	3.35095000	-2.80442100	0.25149300
S	2.39305400	-2.54507800	1.36626100
C	3.15310200	-3.23354000	2.86237500
H	3.18102700	-4.32415000	2.68329700
H	4.17694500	-2.83292900	2.97400100
H	2.52299500	-2.99625700	3.73923600
C	-1.22999900	0.06674700	1.16992200
C	-1.65904500	1.27477800	1.95737000
C	-1.12237100	-1.18436700	1.73885200
C	-1.87085300	0.94217900	3.44942500
H	-2.62209600	1.62803100	1.54143100
H	-0.93736600	2.10236100	1.82382600
C	-0.90787800	-1.35676500	3.21568900
H	-1.10425600	-2.09530600	1.13013100
C	-0.80399000	-0.02693600	3.96523000
H	-2.87246500	0.47583600	3.55668000
H	-1.88003100	1.88464100	4.03494200
H	-0.00928700	-1.99424900	3.36130600
H	-1.76417000	-1.95754000	3.60716300
H	0.20845200	0.40605400	3.80824400
H	-0.91618500	-0.19309400	5.05634100
O	-2.47481500	-0.01645900	-0.34075300
S	-3.90790300	-0.51176000	0.03935200
O	-4.27404000	-0.23934700	1.44801000
O	-4.20939900	-1.84278900	-0.53404800
C	-4.91510000	0.73076300	-0.96522200
F	-4.67041000	0.59975200	-2.27465400
F	-4.58532200	1.98630300	-0.58716800
F	-6.22256300	0.54789000	-0.74123300
C	2.30039900	1.55083900	1.22477800
C	3.55889800	1.77678800	0.60651000
C	1.68022000	2.64941700	1.86715100
C	4.14486700	3.05039200	0.59344400
H	4.06409600	0.92787800	0.12216800

C	2.27123100	3.91788900	1.86204100
H	0.73086200	2.49734900	2.39513700
C	3.49876700	4.13239100	1.21022300
H	5.11770600	3.19570400	0.09796000
H	1.76721400	4.75136300	2.37502100
H	3.95440900	5.13410100	1.19924900

N4-5ETS2:

Eel = -4342.798823
Zero-point correction = 0.538302
Thermal correction to Energy = 0.580987
Thermal correction to Enthalpy = 0.581932
Thermal correction to Gibbs Free Energy = 0.460461

C	-1.98029400	0.49536800	3.50704700
C	-1.31835300	-0.17221500	2.46377500
C	-0.08089300	1.75683500	1.95372800
C	-0.69187600	2.46469700	3.00822200
C	-1.65157900	1.82934700	3.79352800
H	-2.72620700	-0.04713500	4.10335100
H	-0.42774000	3.51363600	3.19268200
H	-2.14475000	2.36378100	4.61825500
C	-1.64901500	-1.60238400	2.19442200
C	-2.99937800	-2.01062500	2.12002700
C	-0.89344100	-3.75528400	1.88309300
C	-3.27169200	-3.36976900	1.91641600
H	-3.80694800	-1.26514900	2.13853800
C	-2.19965800	-4.26811300	1.80555400
H	-0.02224400	-4.42801000	1.78611500
H	-4.31251500	-3.72003500	1.83748800
H	-2.36663000	-5.34497700	1.65416100
C	0.85540800	2.38537900	1.00757100
C	1.20544200	3.74941800	1.03536600
C	2.07790400	4.25600200	0.06985400
H	0.78650400	4.41337900	1.80229400
C	2.17952000	2.04902500	-0.89504200
C	2.58147200	3.38644200	-0.91210400
H	2.35800400	5.31936300	0.07914900
H	2.55376900	1.31828500	-1.62562500
H	3.28146100	3.73526100	-1.68422400
N	-0.37730600	0.44652300	1.70764000
N	1.33962800	1.55989500	0.03671300
N	-0.61823600	-2.45659200	2.06638900

Ni	0.41851700	-0.19321100	-0.04908100
C	-1.32525300	0.42831700	-0.82333000
C	-2.28591900	-0.51325200	-0.84776900
H	-2.11765800	-1.56526100	-0.57104000
O	-4.64962300	0.01341100	0.28217600
O	-4.26081000	0.86990200	-2.10967700
S	-4.03568900	-0.16178700	-1.07126800
C	-4.66859000	-1.74094500	-1.70875200
H	-5.75993700	-1.59429100	-1.80860500
H	-4.20643500	-1.94479000	-2.69209100
H	-4.44738900	-2.54191700	-0.97887400
C	1.02474000	-1.14829300	-1.54425400
C	0.86538900	-0.87742300	-3.00658200
C	0.53991400	-2.26520400	-0.88852400
C	0.45870500	-2.16365300	-3.74992800
H	1.81168500	-0.46581800	-3.40912400
H	0.08932900	-0.09445300	-3.14061000
C	-0.33851500	-3.26775900	-1.61281600
H	0.94162000	-2.57016600	0.08823100
C	-0.72115600	-2.83360600	-3.03549400
H	1.32605900	-2.85829400	-3.77934100
H	0.19848900	-1.92163000	-4.80075400
H	-1.24262900	-3.50539900	-1.00942700
H	0.23573900	-4.22312400	-1.65608500
H	-1.56499300	-2.11131800	-2.99227600
H	-1.08021200	-3.71258600	-3.60945300
O	2.34694100	-0.93168000	1.09210600
S	3.34837700	-1.42038200	0.08536500
O	2.96430200	-0.94316000	-1.34356300
O	3.77507700	-2.82828000	0.16177300
C	4.90274700	-0.39366000	0.39771500
F	5.42151100	-0.74825300	1.57875100
F	4.60976600	0.91713200	0.42681200
F	5.80344600	-0.61704200	-0.56710500
C	-1.52936100	1.86893900	-0.98903600
C	-2.34800900	2.57177300	-0.06872500
C	-0.84484200	2.61289700	-1.98082300
C	-2.46106300	3.96635800	-0.13276200
H	-2.90999700	2.00247500	0.68637300
C	-0.97444500	4.00332300	-2.05074300
H	-0.20163300	2.08342600	-2.69883200
C	-1.77260300	4.69049500	-1.11850300
H	-3.10374100	4.49096100	0.59117300
H	-0.43869000	4.56083100	-2.83431500

H	-1.86381600	5.78613700	-1.16900800

N5Z':

Eel = -4342.859110
Zero-point correction = 0.541546
Thermal correction to Energy = 0.584232
Thermal correction to Enthalpy = 0.585176
Thermal correction to Gibbs Free Energy = 0.465320

C	1.02438500	2.28258200	-3.17968700
C	0.93649200	1.60960300	-1.94687100
C	-0.82319000	0.26612900	-2.74341200
C	-0.77966100	0.89548900	-4.00013500
C	0.14354400	1.92989400	-4.20540100
H	1.76848200	3.07529300	-3.32626100
H	-1.44663100	0.57692700	-4.81149900
H	0.18652400	2.44737900	-5.17485500
C	1.86230600	1.80643500	-0.81431200
C	3.01995800	2.59583600	-0.89072300
C	2.37933400	1.14317000	1.35649800
C	3.88375200	2.63695200	0.20887400
H	3.25063700	3.15877800	-1.80440400
C	3.56412300	1.89084100	1.34778700
H	2.08124600	0.53576600	2.21913600
H	4.80205000	3.24152900	0.17016300
H	4.21924300	1.87700800	2.22968200
C	-1.69389300	-0.88768900	-2.41606300
C	-2.61402400	-1.43748100	-3.32950800
C	-3.37808600	-2.54339500	-2.94298800
H	-2.73513200	-1.00379600	-4.33113300
C	-2.25406900	-2.47258400	-0.81410400
C	-3.19230300	-3.07816000	-1.66184200
H	-4.10873700	-2.98383800	-3.63748100
H	-2.07893900	-2.85507300	0.20019300
H	-3.76286600	-3.95015600	-1.31315300
N	-0.00632500	0.66588600	-1.74581400
N	-1.53467100	-1.40356400	-1.17523200
N	1.55043400	1.11190000	0.30554800
Ni	-0.17353200	-0.09647500	0.09758800
C	-1.70291200	0.99316700	0.62039800
C	-1.78719200	2.32631600	0.85319700
H	-2.77089200	2.79841900	1.03298100
O	0.49366500	3.29288100	1.98141900

O	-0.01876000	3.88102200	-0.46501100
S	-0.50501100	3.56468400	0.91049300
C	-1.44846800	5.01540800	1.45338700
H	-0.69526600	5.82298400	1.50218100
H	-2.22724800	5.25454000	0.70660000
H	-1.87806700	4.82319400	2.45335300
C	-0.31891300	-0.70894200	1.93165900
C	-0.48717200	-2.20467900	2.07712100
C	-0.14859100	0.10372800	2.99742500
C	0.14471200	-2.70652000	3.38827300
H	-0.04877600	-2.75372500	1.22221500
H	-1.57567300	-2.44776800	2.08013100
C	0.03129200	-0.40452700	4.41270400
H	-0.06948400	1.19446500	2.85991400
C	-0.34906700	-1.88034700	4.57686800
H	1.24696800	-2.62329700	3.28589500
H	-0.09142600	-3.78287000	3.52231700
H	-0.54992800	0.23455900	5.11362300
H	1.09801500	-0.25204600	4.70281100
H	-1.45701600	-1.96995000	4.64734900
H	0.05527800	-2.27739300	5.53151200
O	1.17289100	-1.54887400	-0.52608400
S	2.32086400	-2.28168500	0.18453100
O	2.16717800	-3.75222700	0.13118400
O	2.75217500	-1.67490600	1.47247700
C	3.73555200	-1.88951700	-0.99698900
F	3.93308900	-0.55341900	-1.06749700
F	3.46608600	-2.33866900	-2.23385200
F	4.87147100	-2.46011600	-0.56782600
C	-3.03233100	0.31833100	0.58677900
C	-3.88555300	0.51600000	-0.52398000
C	-3.49189400	-0.49519400	1.64625600
C	-5.13219700	-0.12103700	-0.59800400
H	-3.54676000	1.16824000	-1.34310100
C	-4.74648200	-1.11769400	1.57765100
H	-2.85154100	-0.61689500	2.53029100
C	-5.56655100	-0.94840000	0.45004900
H	-5.77175400	0.03488300	-1.48063100
H	-5.08808500	-1.74075600	2.41876000
H	-6.54545700	-1.44828300	0.39430000

N5E':

Eel = -4342.870287

Zero-point correction = 0.541079
Thermal correction to Energy = 0.584201
Thermal correction to Enthalpy = 0.585145
Thermal correction to Gibbs Free Energy = 0.461666

C	-1.03485400	-0.05977400	-4.01318600
C	-0.90667400	-0.31653400	-2.63559600
C	0.11100500	1.77690100	-2.28310200
C	0.00425400	2.09500700	-3.65017800
C	-0.56523300	1.15738300	-4.52029600
H	-1.50897600	-0.79397700	-4.67721900
H	0.35140900	3.06476400	-4.02927700
H	-0.65788100	1.38270000	-5.59261600
C	-1.43600000	-1.51654600	-1.95229500
C	-2.12612400	-2.54752000	-2.61203500
C	-1.80603000	-2.51012800	0.12474000
C	-2.67202900	-3.59007800	-1.85270800
H	-2.24734100	-2.53274800	-3.70342000
C	-2.52218400	-3.56509800	-0.46132300
H	-1.66768300	-2.42376400	1.21038200
H	-3.21894000	-4.40706900	-2.34602700
H	-2.95585900	-4.34782000	0.17661800
C	0.68009500	2.67827000	-1.25478600
C	1.25479700	3.92539100	-1.56358700
C	1.79770000	4.69862900	-0.53206300
H	1.28917500	4.28425700	-2.60090800
C	1.14447200	2.95945500	1.00227000
C	1.74362800	4.20695700	0.77790300
H	2.26048300	5.67211500	-0.75123300
H	1.08197700	2.53697100	2.01337800
H	2.15835100	4.77336200	1.62324700
N	-0.31799400	0.58471300	-1.81931800
N	0.62993100	2.21391200	0.01646500
N	-1.26325300	-1.52867400	-0.60742100
Ni	-0.12333800	0.12146700	0.11586400
C	1.65419600	-0.63146100	-0.07247000
C	1.78313700	-1.84880100	-0.63736000
H	0.95370400	-2.49629100	-0.95531700
O	4.08190800	-2.59396500	0.56513500
O	2.90395300	-4.16813000	-1.10889300
S	3.31237600	-2.80318200	-0.68694300
C	4.31151800	-2.11145100	-2.03469600
H	5.20328400	-2.76446700	-2.08932800
H	3.73122000	-2.16298600	-2.97383400

H	4.59577000	-1.07534400	-1.77674900
C	0.11324700	-0.46979500	1.95973600
C	-0.19417300	0.59748600	2.98832100
C	0.37913600	-1.74152000	2.32997000
C	-0.78723800	-0.01716300	4.26845300
H	-0.88051100	1.36870400	2.58980100
H	0.75681700	1.11930400	3.25090200
C	0.25694800	-2.25526600	3.74890000
H	0.68481300	-2.48920500	1.58055900
C	0.11400900	-1.13763000	4.78859900
H	-1.79710700	-0.40743900	4.02250600
H	-0.92036300	0.77693100	5.03260400
H	1.12865600	-2.90490000	3.98277900
H	-0.62885400	-2.93256900	3.79500800
H	1.11934900	-0.71721300	5.01664400
H	-0.27384600	-1.54986100	5.74345700
O	-1.91833300	1.10089600	0.41388700
S	-3.15078100	0.72961800	1.25454400
O	-3.58717300	1.83831300	2.12847700
O	-3.12668200	-0.64348100	1.82671800
C	-4.45116500	0.62685700	-0.10685000
F	-4.10933200	-0.32161600	-1.01317400
F	-4.56260700	1.79804600	-0.75394300
F	-5.64494700	0.30083100	0.40868500
C	2.79427900	0.22993000	0.30254800
C	3.41257400	1.03194000	-0.68532100
C	3.26293400	0.31806800	1.63245600
C	4.44995500	1.91453500	-0.35070400
H	3.05494700	0.96779400	-1.72447400
C	4.30734900	1.19124400	1.96030800
H	2.80151400	-0.32370700	2.39454900
C	4.89982000	2.00099800	0.97608100
H	4.91036100	2.53732300	-1.13330000
H	4.66939600	1.23514100	2.99895700
H	5.71580300	2.69018600	1.24134200

N8:

Eel = -3445.991293

Zero-point correction = 0.384431

Thermal correction to Energy = 0.413753

Thermal correction to Enthalpy = 0.414697

Thermal correction to Gibbs Free Energy = 0.323300

C	-4.33722100	1.89992500	-0.39169000
C	-2.96805200	1.64833500	-0.21564900
C	-3.30811300	-0.68583800	-0.45868200
C	-4.68817800	-0.49485800	-0.64165900
C	-5.20768500	0.80871400	-0.57474700
H	-4.71760500	2.93072200	-0.42296100
H	-5.34377100	-1.34668200	-0.87181700
H	-6.28336300	0.98122200	-0.72393100
C	-1.88041000	2.63187800	-0.15822400
C	-2.06200500	4.00197300	0.10564000
C	0.42390100	2.93028000	-0.40064600
C	-0.95428800	4.85495300	0.10498600
H	-3.06899600	4.38696900	0.32208700
C	0.31279400	4.30287100	-0.16053600
H	1.39493700	2.46860900	-0.62666900
H	-1.07294200	5.92902300	0.30984300
H	1.21381800	4.93229200	-0.19648200
C	-2.53975500	-1.91054900	-0.66915700
C	-3.09391600	-3.19415600	-0.81962000
C	-2.26714100	-4.27512100	-1.13598700
H	-4.17676800	-3.33358100	-0.68765900
C	-0.39057100	-2.74007900	-1.13083000
C	-0.89007800	-4.02837300	-1.31738900
H	-2.68346700	-5.28561000	-1.25620700
H	0.67312200	-2.50996000	-1.29004000
H	-0.20154500	-4.83156400	-1.61751700
N	-2.50307800	0.37259800	-0.15823800
N	-1.17260000	-1.68415900	-0.77177400
N	-0.63767500	2.08942300	-0.39786600
Ni	-0.61982100	0.01707900	0.01820900
C	0.97844500	-0.42283100	1.08900400
C	1.27842700	-1.84955200	1.54065200
C	0.08949800	0.42651900	1.81645400
C	0.58815200	-2.21444200	2.86714400
H	2.37853600	-1.94334800	1.64541100
H	0.99459800	-2.58256900	0.76046300
C	-0.75742600	-0.10761500	2.95453000
H	0.35472500	1.49555900	1.87624700
C	-0.82738100	-1.63724200	2.96228800
H	1.18528400	-1.81778800	3.71832300
H	0.57464200	-3.31804600	2.98077100
H	-1.77320700	0.34004800	2.91086100
H	-0.31462900	0.23822800	3.91984500
H	-1.43886000	-1.99223000	2.10384500

H	-1.33251200	-1.99085000	3.88480200
O	2.61961400	-1.53189000	-1.15216400
S	2.78832300	-0.10338900	-0.83311300
O	2.19874200	0.28665700	0.64398200
O	2.48716800	0.93808700	-1.82808100
C	4.59452400	0.13261100	-0.33302700
F	5.35277200	-0.09502800	-1.41036800
F	4.91881200	-0.73391200	0.63161400
F	4.79152800	1.38152700	0.09751700

P1:

Eel = -5962.161875
Zero-point correction = 0.665847
Thermal correction to Energy = 0.714803
Thermal correction to Enthalpy = 0.715747
Thermal correction to Gibbs Free Energy = 0.578636

Fe	3.19225900	1.97918200	-0.21670500
P	-0.17503700	1.65331300	-0.20333400
P	1.84628700	-1.12722000	0.18476900
C	-0.36664500	2.18757400	1.55639000
C	-1.56241600	2.58683500	-0.98848600
C	-0.33735700	1.20686800	2.56607400
H	-0.20898500	0.15134400	2.28463500
C	3.18359600	0.08741700	0.55377500
C	3.04985400	-1.91579000	-2.21725900
H	2.84413500	-0.87865600	-2.52422600
C	3.85349300	-4.14713100	-2.75469700
H	4.29402900	-4.85502300	-3.47295200
C	1.32074900	2.53607400	-0.78395500
C	-2.52542300	3.85997300	-2.83091700
H	-2.39405000	4.41467500	-3.77281700
C	-0.57554900	3.53685700	1.91033500
H	-0.65262300	4.30333400	1.12371200
C	1.70530600	-2.00466600	1.80732200
C	-3.80520300	3.71832400	-2.27181700
H	-4.67991600	4.15634700	-2.77607200
C	-0.65378100	2.91773000	4.26333800
H	-0.76384700	3.20526800	5.32017400
C	2.71953100	-2.33366100	-0.90790300
C	2.94660800	-3.67489200	-0.54267600
H	2.67948700	-4.02014400	0.46698500
C	3.08692800	1.07917800	1.60327800

H	2.25726200	1.18114600	2.31254000
C	3.62516700	-2.81227100	-3.12865000
H	3.88336900	-2.47021000	-4.14262200
C	-2.85198300	2.44968700	-0.42849300
H	-2.98553100	1.87532000	0.50051800
C	-3.96463000	3.01295700	-1.06637300
H	-4.96474600	2.88398400	-0.62512300
C	-0.70925500	3.89947200	3.25871900
H	-0.86737300	4.95513300	3.52817000
C	4.41455500	0.33450900	-0.15983000
H	4.77873500	-0.23690300	-1.02109400
C	3.22649900	3.86587400	-1.01401600
H	3.99813000	4.62262800	-0.82437900
C	-1.40652000	3.29770500	-2.19397100
H	-0.40539000	3.41038900	-2.63777600
C	5.06379100	1.46528000	0.44298300
H	6.00598400	1.91721700	0.10862800
C	3.50732900	-4.57457200	-1.46379900
H	3.67599700	-5.62098000	-1.16621200
C	1.39768700	-3.29045500	4.29762000
H	1.28035700	-3.79045500	5.27129400
C	2.67688500	-2.91769600	3.84916600
H	3.56104300	-3.12550800	4.47135800
C	2.83189200	-2.27491300	2.61195300
H	3.83347700	-1.97419600	2.26734400
C	-0.47618600	1.56985500	3.91494700
H	-0.45155600	0.79099700	4.69229600
C	0.27119800	-3.01852100	3.50543700
H	-0.73803200	-3.29661300	3.84538100
C	2.08236800	2.05856000	-1.91926300
H	1.80107600	1.19721100	-2.53940100
C	0.42455100	-2.37798900	2.26601500
H	-0.46326200	-2.14697500	1.65691900
C	2.04261500	3.65838900	-0.22654900
H	1.75901900	4.21944900	0.67107400
C	4.24828100	1.92008900	1.53398000
H	4.45434000	2.78213000	2.18041600
C	3.25188400	2.87958000	-2.05851700
H	4.04136900	2.75552000	-2.80990800
Ni	-0.07769500	-0.47878800	-0.54332400
C	-1.87693300	-1.10064300	-1.09864600
C	-2.58988100	-0.54140400	-2.30433200
C	-0.96515700	-2.19480500	-1.16627200
C	-1.76189400	-0.79784700	-3.56772500

H	-2.80008100	0.53757100	-2.17252200
H	-3.57469700	-1.05322900	-2.39710300
C	-0.50795800	-2.74282200	-2.51775600
H	-0.95814200	-2.90633400	-0.32395300
C	-1.40322200	-2.28379000	-3.67921400
H	-0.83141000	-0.18536600	-3.52448300
H	-2.32559200	-0.45740200	-4.46040900
H	-0.48682200	-3.85160300	-2.46935000
H	0.54275400	-2.44481600	-2.72268900
H	-2.34735000	-2.87173000	-3.67212200
H	-0.90220000	-2.49713800	-4.64655900
O	-4.06590000	-2.97196200	-0.52333000
S	-3.77966300	-2.02542000	0.56574400
O	-2.59453600	-0.93241000	0.15221900
O	-3.53493700	-2.45223200	1.95097000
C	-5.21486400	-0.78695600	0.63769900
F	-6.31908100	-1.42276600	1.04191200
F	-5.42307700	-0.25974300	-0.57530000
F	-4.92437900	0.19487600	1.50093100

P1-2TS:

Eel = -5962.147793
Zero-point correction = 0.663872
Thermal correction to Energy = 0.712871
Thermal correction to Enthalpy = 0.713815
Thermal correction to Gibbs Free Energy = 0.577119

Fe	-3.07112400	2.01545400	-0.40539400
P	0.27295100	1.65980800	0.16397800
P	-1.78812700	-1.12600900	-0.11553300
C	0.75965100	1.81793400	-1.60611500
C	1.50968400	2.75773000	0.98295900
C	0.89576400	0.65033400	-2.37941100
H	0.73774800	-0.32998400	-1.91095300
C	-3.00786200	0.03131200	-0.84726300
C	-2.86362700	-1.76835000	2.37000000
H	-2.19691100	-0.95739800	2.70767800
C	-4.48825000	-3.51305400	2.84066800
H	-5.12006400	-4.06037400	3.55665900
C	-1.27895900	2.61214000	0.32444000
C	2.09131100	4.56347800	2.51415600
H	1.77759100	5.36878800	3.19610300
C	1.04171200	3.07289400	-2.18537300

H	0.99761700	3.98480900	-1.57017000
C	-1.39824100	-2.21552400	-1.55129600
C	3.45513600	4.31549500	2.29605200
H	4.21571700	4.92326200	2.80958100
C	1.51867300	1.98730500	-4.31075800
H	1.81743800	2.05533600	-5.36800400
C	-2.86586700	-2.12317100	1.00525900
C	-3.67659100	-3.18797700	0.56745900
H	-3.66980800	-3.48560300	-0.49163100
C	-2.78162100	0.83347900	-2.03065900
H	-1.87768800	0.81571000	-2.65187800
C	-3.67888700	-2.45406200	3.28289500
H	-3.67226600	-2.16909300	4.34607100
C	2.88308200	2.50588600	0.76852600
H	3.19240300	1.67071500	0.12244500
C	3.84597000	3.28716700	1.42131900
H	4.91389600	3.08160000	1.25136100
C	1.40882100	3.15543800	-3.53630900
H	1.62569400	4.13705500	-3.98486000
C	-4.30332800	0.38587500	-0.31765800
H	-4.75470900	-0.03568600	0.58831600
C	-3.11664800	4.01908100	0.02394800
H	-3.80926800	4.74970100	-0.41239900
C	1.11899100	3.79009000	1.85864900
H	0.05095100	3.99426700	2.02716100
C	-4.86981900	1.39455900	-1.16848100
H	-5.83621700	1.89180400	-1.01897700
C	-4.48075900	-3.88123800	1.48518300
H	-5.10491600	-4.71902700	1.13853900
C	-0.67670100	-3.80471300	-3.76531900
H	-0.39615800	-4.42027600	-4.63379400
C	-1.97471400	-3.27330200	-3.67183200
H	-2.71095200	-3.47207700	-4.46578600
C	-2.33494000	-2.47580800	-2.57514600
H	-3.34278200	-2.03591200	-2.52474400
C	1.27275000	0.73475000	-3.72818200
H	1.38993400	-0.18848100	-4.31510400
C	0.26171000	-3.53769600	-2.75666300
H	1.29047400	-3.92383300	-2.81349300
C	-2.21914600	2.37630900	1.40100800
H	-2.08531600	1.63376700	2.19798600
C	-0.09707400	-2.74875700	-1.65349100
H	0.67887200	-2.53854300	-0.90109700
C	-1.84735100	3.63398400	-0.52683500

H	-1.40574900	4.00997200	-1.45644000
C	-3.93574300	1.66543000	-2.22615500
H	-4.06266700	2.40663000	-3.02488700
C	-3.34660300	3.24482500	1.21212000
H	-4.24220400	3.28412100	1.84424000
Ni	0.01547600	-0.36830700	0.90617200
C	1.43136600	-0.59887800	2.03250800
C	2.26854500	0.05619700	3.06100600
C	0.89730900	-1.87086100	2.07012600
C	1.92497900	-0.56918300	4.43954400
H	2.15517800	1.15687100	3.07474900
H	3.32397300	-0.17464000	2.79877300
C	0.73385300	-2.56886300	3.41773700
H	0.89006100	-2.50588000	1.16690500
C	1.84188500	-2.09826100	4.37279200
H	0.95440800	-0.15671800	4.79377600
H	2.69302300	-0.24721600	5.17229800
H	0.77733500	-3.66681700	3.27400500
H	-0.26983800	-2.35248700	3.84715500
H	2.81451300	-2.49578000	4.00978800
H	1.67606100	-2.51777600	5.38718800
O	4.48959500	-1.83482200	1.16199000
S	3.48750500	-1.77951900	0.07223200
O	2.56799200	-0.54732900	0.14366000
O	2.76373900	-3.03789200	-0.26984900
C	4.44297200	-1.36276900	-1.50386800
F	5.42068000	-2.25717500	-1.70796900
F	4.98956300	-0.13639900	-1.41711300
F	3.61074100	-1.38528300	-2.56526900

P2:

Eel = -5000.483029
Zero-point correction = 0.637318
Thermal correction to Energy = 0.677739
Thermal correction to Enthalpy = 0.678684
Thermal correction to Gibbs Free Energy = 0.562614

Fe	-0.62325000	-2.29505300	-1.70851500
P	1.61158200	-0.45452700	0.04782600
P	-1.72484500	0.30456500	0.13007900
C	1.51794300	-1.53888800	1.52597300
C	3.41306900	-0.16191500	-0.14615200
C	0.58750300	-1.25536700	2.54424400

H	-0.05816500	-0.36898700	2.46084400
C	-1.98378900	-1.37174400	-0.54038700
C	-2.20815600	2.00705000	-2.04399800
H	-1.11646600	1.95422400	-2.18835000
C	-4.38761800	2.83431900	-2.72974200
H	-5.00912300	3.42532900	-3.41897000
C	1.18104900	-1.47646700	-1.39321400
C	5.36619400	0.15586700	-1.56501000
H	5.82187900	0.16895300	-2.56638100
C	2.37566200	-2.65300200	1.65358500
H	3.14417400	-2.85133700	0.89122500
C	-2.59130300	0.27090300	1.75160500
C	6.14937900	0.42250600	-0.43084500
H	7.22052700	0.64779500	-0.54191300
C	1.31295900	-3.22558100	3.76799500
H	1.23176700	-3.88921800	4.64186300
C	-2.80313300	1.32630000	-0.96122300
C	-4.19592800	1.41067800	-0.76367500
H	-4.66594500	0.89235100	0.08550500
C	-1.55432500	-2.61876300	0.06192600
H	-1.06146900	-2.72916900	1.03476200
C	-3.00002300	2.75510700	-2.92835300
H	-2.53095900	3.28225200	-3.77263600
C	4.20153300	0.10615300	0.99200300
H	3.74812100	0.08605100	1.99506300
C	5.56597700	0.39336800	0.84700900
H	6.17907800	0.59425300	1.73836700
C	2.26375900	-3.49586500	2.76843100
H	2.93053100	-4.36605500	2.86263100
C	-2.56852500	-1.68156200	-1.82695600
H	-2.97254600	-0.94933000	-2.53557200
C	0.79610900	-3.25016100	-2.85452100
H	0.71758400	-4.26507300	-3.26327600
C	4.00014000	-0.13736200	-1.42688700
H	3.39245000	-0.35431300	-2.31813900
C	-2.49536100	-3.10093100	-2.01258400
H	-2.82713000	-3.64745000	-2.90374600
C	-4.98210600	2.16523600	-1.64682900
H	-6.06849700	2.23320100	-1.48629000
C	-3.87539800	0.29461600	4.25710100
H	-4.37613400	0.30171600	5.23677100
C	-4.19775200	-0.69091100	3.31049100
H	-4.95259700	-1.45609000	3.54597000
C	-3.55907200	-0.70700600	2.06075400

H	-3.81015000	-1.48598100	1.32553300
C	0.48195100	-2.09970500	3.66015800
H	-0.25104700	-1.86988500	4.44788600
C	-2.90996300	1.26917500	3.95458300
H	-2.65207400	2.04053400	4.69553000
C	0.59241300	-0.94753400	-2.60874300
H	0.35496300	0.10731000	-2.79388100
C	-2.26741300	1.25607300	2.70860800
H	-1.49799500	2.01184500	2.48155300
C	1.30429600	-2.91098100	-1.55675000
H	1.67725400	-3.61723700	-0.80696000
C	-1.87845300	-3.67696600	-0.85189700
H	-1.65740800	-4.74067600	-0.70109100
C	0.35699500	-2.04657100	-3.50043300
H	-0.11001600	-1.98051000	-4.49051500
Ni	0.30398600	1.23601200	0.19173700
C	1.58901300	2.56977700	0.18424800
C	2.97469900	3.08637700	0.06447900
C	0.48114200	3.30913500	0.30154300
C	2.91378900	4.59291900	-0.29472100
H	3.55823800	2.52015500	-0.69002000
H	3.50894000	2.92818900	1.02666400
C	0.44218400	4.82081700	0.32382300
H	-0.55495200	2.79841100	0.35712200
C	1.87195900	5.34997400	0.54036700
H	2.66705500	4.69411700	-1.37396800
H	3.91843200	5.04103100	-0.15727100
H	-0.24666000	5.18836000	1.11265900
H	0.01876300	5.18137500	-0.64063200
H	2.13164600	5.25988800	1.61750600
H	1.90139000	6.43204100	0.30029100

R1-2TS:

Eel = -896.741211
Zero-point correction = 0.150872
Thermal correction to Energy = 0.163350
Thermal correction to Enthalpy = 0.164295
Thermal correction to Gibbs Free Energy = 0.109123

O	1.81063000	-1.49803100	0.44102000
O	3.86123500	0.07551400	0.06757500
S	2.38941500	-0.19237600	-0.01165600
C	1.02075300	1.54807600	0.99080900

H	1.82038100	2.12547100	1.45008100
C	-0.17127100	1.23868600	0.77893800
C	-1.37963600	0.59369900	0.40983000
C	-1.55244300	-0.78508000	0.71948000
C	-2.41122400	1.28559700	-0.28016000
C	-2.73116200	-1.43940000	0.34978300
H	-0.73125300	-1.32192300	1.21582100
C	-3.58424700	0.61558600	-0.64107200
H	-2.27601600	2.35036300	-0.51930600
C	-3.75023400	-0.74465800	-0.32580300
H	-2.85512600	-2.50706700	0.58592500
H	-4.38031300	1.15878300	-1.17230700
H	-4.67637100	-1.26602200	-0.61119600
C	1.90021700	-0.00353000	-1.76605500
H	2.26820500	0.97630300	-2.11866700
H	2.38185500	-0.83762700	-2.31079100
H	0.79857000	-0.07397400	-1.81633000

P2-5TS:

Eel = -5588.959118
Zero-point correction = 0.681922
Thermal correction to Energy = 0.728119
Thermal correction to Enthalpy = 0.729063
Thermal correction to Gibbs Free Energy = 0.599197

Fe	1.82518600	-1.30946100	-2.24815200
P	-0.59873100	-1.54949200	0.18072800
P	1.65120900	1.12339300	0.00764500
C	-2.20062700	-2.09079300	-0.53420900
C	-0.38357700	-2.51362300	1.73138600
C	-2.66241100	-1.45852600	-1.70836400
H	-2.08877500	-0.63126800	-2.15102600
C	2.05142000	0.61549300	-1.69205200
C	2.76643400	-0.27893100	2.14225300
H	1.72761900	-0.58829300	2.33896100
C	5.12899300	-0.34294200	2.70759700
H	5.94609600	-0.70335600	3.35033500
C	0.62706700	-2.29432200	-0.94657400
C	0.23135500	-4.57307900	2.87826700
H	0.58665200	-5.61366000	2.83852500
C	-2.96755100	-3.11017100	0.06328800
H	-2.61609300	-3.60103000	0.98256700
C	1.66147900	2.96406100	0.03327800

C	-0.05920000	-3.97424500	4.11520300
H	0.07174900	-4.54496400	5.04677200
C	-4.63794200	-2.88383100	-1.69367800
H	-5.58927600	-3.19690100	-2.14915400
C	3.04439800	0.57578000	1.05536500
C	4.37367200	0.98570700	0.81264000
H	4.59808200	1.67771500	-0.01317700
C	1.04888200	0.50944000	-2.73327900
H	-0.00242700	0.80764400	-2.63556700
C	3.80702800	-0.73971700	2.96301800
H	3.58090600	-1.40976100	3.80594600
C	-0.67956100	-1.91792200	2.97528700
H	-1.04305600	-0.87913600	3.00268500
C	-0.51648300	-2.64745300	4.16207500
H	-0.74664700	-2.17594800	5.12920000
C	-4.18640200	-3.49917000	-0.51649000
H	-4.78372200	-4.29317400	-0.04389600
C	3.29352100	0.09530400	-2.22241800
H	4.23691800	-0.01148100	-1.67592100
C	1.64882800	-3.29035000	-2.78698600
H	1.81588700	-3.70421500	-3.78888800
C	0.07403300	-3.84836700	1.68777200
H	0.30923000	-4.32094500	0.72274600
C	3.05184900	-0.32288500	-3.57329100
H	3.78756700	-0.79407100	-4.23656400
C	5.40989400	0.52278800	1.63665600
H	6.44476600	0.84330600	1.44421500
C	1.37871500	5.76076800	0.16157600
H	1.26932800	6.85437000	0.21306100
C	0.58167900	5.01411300	-0.72183900
H	-0.16584300	5.51450600	-1.35512300
C	0.71753800	3.62091200	-0.78838000
H	0.06092700	3.04439300	-1.45804400
C	-3.87217400	-1.86659900	-2.28848000
H	-4.22173200	-1.38762600	-3.21643500
C	2.30750900	5.10836800	0.98730900
H	2.92925900	5.68900200	1.68533000
C	2.02647700	-2.50757600	-0.62828200
H	2.52087700	-2.24410000	0.31425900
C	2.44758700	3.71236000	0.93269300
H	3.16698700	3.21032200	1.59579500
C	0.40415100	-2.77802000	-2.29313000
H	-0.54932100	-2.75371700	-2.83256000
C	1.67561500	-0.06667900	-3.88630300

H	1.17722200	-0.30348500	-4.83407300
C	2.64286600	-3.12911700	-1.76519200
H	3.70289900	-3.39806100	-1.84910900
Ni	-0.39769900	0.52325500	0.65961300
C	-2.10039300	0.76667800	1.28313700
C	-1.53721100	2.07941500	1.75467400
C	-3.34859800	0.32451800	1.54234600
C	-2.23440700	2.47875200	3.07528100
H	-1.68120900	2.88580800	1.00064900
H	-0.40537000	2.02405000	1.91387800
C	-4.30277600	1.13621500	2.38816800
H	-3.67388500	-0.67554200	1.20842700
C	-3.74608500	2.50863800	2.80069400
H	-1.87191300	3.47118700	3.40877700
H	-1.98836800	1.74551800	3.87263800
H	-5.27620700	1.25085000	1.86252600
H	-4.54092600	0.51774900	3.28541200
H	-4.28807100	2.88068800	3.69332900
H	-3.93383500	3.23907900	1.98595600
S	-3.07368500	2.13986100	-1.23236000
O	-3.05196300	3.53634500	-0.65003400
O	-1.82412900	1.66634300	-1.96126200
C	-4.38789100	2.17718000	-2.51910200
H	-5.31436400	2.53740100	-2.03726000
H	-4.03538900	2.87953000	-3.29797400
H	-4.50151100	1.15164400	-2.91337200

R2:

Eel = -896.767453
Zero-point correction = 0.152987
Thermal correction to Energy = 0.165097
Thermal correction to Enthalpy = 0.166041
Thermal correction to Gibbs Free Energy = 0.111556

O	1.40818900	-0.80592300	-1.21544000
O	3.53797500	-0.80114300	0.26184300
S	2.26912700	-0.18074400	-0.18675500
C	1.24475100	0.09449300	1.34048800
H	1.86241900	0.14317100	2.25405700
C	-0.05474500	0.13210300	1.22380900
C	-1.31016800	0.07170100	0.62710900
C	-1.89340800	-1.19603700	0.29538000
C	-2.05672800	1.26291400	0.34560700

C	-3.15565400	-1.25365300	-0.29071100
H	-1.31648700	-2.11055000	0.49054200
C	-3.31634600	1.17873000	-0.24445300
H	-1.61973100	2.23905900	0.60121200
C	-3.87683400	-0.07400100	-0.56388600
H	-3.58650100	-2.23250000	-0.55033000
H	-3.87618400	2.10138600	-0.46035400
H	-4.87282400	-0.13071400	-1.02739500
C	2.64547800	1.50350400	-0.74831800
H	3.19543100	2.03458800	0.04968500
H	3.28272500	1.37598700	-1.64414600
H	1.69774000	2.00948100	-1.00656400

P5:

Eel = -5588.981779
Zero-point correction = 0.683928
Thermal correction to Energy = 0.730187
Thermal correction to Enthalpy = 0.731131
Thermal correction to Gibbs Free Energy= 0.603621

Fe	1.12960900	-2.87150400	-0.70872100
P	-1.48871600	-0.75119000	0.01633100
P	1.91383200	0.26212700	0.20282100
C	-2.50253600	-0.87589400	-1.50531900
C	-2.64898700	-0.76676100	1.44874700
C	-1.97225500	-0.37198200	-2.71048500
H	-0.99724200	0.13849600	-2.71447500
C	2.30269700	-1.23829000	-0.74563200
C	1.09129500	-0.14455500	2.86239000
H	0.09096200	0.13826600	2.49711900
C	2.54222600	-0.83296900	4.68361400
H	2.68663000	-1.09304800	5.74286600
C	-0.69493900	-2.39738300	0.04965900
C	-3.12570700	-1.30949200	3.78217600
H	-2.82206700	-1.79403300	4.72215800
C	-3.73617300	-1.55956700	-1.51333200
H	-4.14267200	-1.98406800	-0.58376100
C	3.29255400	1.41416500	-0.15302300
C	-4.35678400	-0.64274500	3.69694700
H	-5.02190400	-0.59393100	4.57183200
C	-3.92129900	-1.19097200	-3.91348100
H	-4.48026000	-1.31024800	-4.85360900
C	2.18029900	-0.16928600	1.96931400

C	3.46432900	-0.49759700	2.45437800
H	4.33169200	-0.46778200	1.77753300
C	1.78975000	-1.50879300	-2.07488900
H	1.19177300	-0.80793300	-2.66804400
C	1.26796100	-0.47846700	4.21408400
H	0.40836200	-0.45236600	4.90077400
C	-3.89311500	-0.10274000	1.36897400
H	-4.20732700	0.36608800	0.42850800
C	-4.74109700	-0.04937100	2.48345600
H	-5.71312700	0.45961400	2.39995000
C	-4.44327500	-1.70756300	-2.71594800
H	-5.40803200	-2.23650300	-2.71717500
C	3.06862200	-2.39258500	-0.32760600
H	3.55615100	-2.52678300	0.64457500
C	-0.07580600	-4.54052100	-0.64205200
H	0.03983700	-5.44637900	-1.24955400
C	-2.27732400	-1.37584700	2.66655900
H	-1.32949800	-1.92054800	2.75315200
C	3.01626600	-3.35992100	-1.38454400
H	3.46626600	-4.35992700	-1.35782600
C	3.63871100	-0.83639100	3.80395700
H	4.64145200	-1.09528300	4.17564300
C	5.29674400	3.29269300	-0.72443700
H	6.08335100	4.02855200	-0.94876200
C	5.01273000	2.26794500	-1.64019600
H	5.57564600	2.19768500	-2.58283900
C	4.00825100	1.32951400	-1.36184200
H	3.77865500	0.53827400	-2.08930500
C	-2.68228500	-0.53050500	-3.91048200
H	-2.26096800	-0.13707600	-4.84785500
C	4.57522500	3.38128300	0.47757900
H	4.79338400	4.18596100	1.19539700
C	0.16729600	-2.97704400	1.06288800
H	0.52289000	-2.48885600	1.97681700
C	3.57214500	2.44751100	0.76456000
H	3.00353200	2.52519500	1.70396800
C	-0.82027900	-3.37486200	-1.01709900
H	-1.38861000	-3.23667200	-1.94311700
C	2.23466500	-2.81514800	-2.45660100
H	1.98753700	-3.32614400	-3.39512100
C	0.52855700	-4.29870900	0.63437500
H	1.19141500	-4.98441600	1.17596500
Ni	-0.20076300	1.07969900	-0.09567000
C	-1.74065300	2.13229000	0.16550200

C	-1.30265200	2.84028300	1.42959500
C	-2.91450900	2.35635900	-0.45790500
C	-2.52988500	3.41896800	2.17017900
H	-0.58630100	3.65031900	1.16653800
H	-0.74620400	2.15674300	2.11469300
C	-3.93976600	3.33651800	0.06547400
H	-3.17644800	1.79157500	-1.36867500
C	-3.39989500	4.21952500	1.19743000
H	-2.18419000	4.05237600	3.01184200
H	-3.11624900	2.58233400	2.60640800
H	-4.32233000	3.95809100	-0.77310200
H	-4.82742800	2.75674200	0.41432800
H	-4.24071300	4.70230400	1.73546900
H	-2.79244800	5.04452000	0.76562900
S	0.35176200	2.34390200	-1.80190600
O	1.11903800	3.43270500	-1.09028100
O	1.05104600	1.46416000	-2.81241500
C	-0.89623900	3.22603200	-2.79202200
H	-1.41503000	3.94589800	-2.13797300
H	-0.29969100	3.73251200	-3.57433500
H	-1.59403400	2.49380300	-3.23499500

P2-3ZTS:

Eel = -5897.260455
Zero-point correction = 0.793905
Thermal correction to Energy = 0.846244
Thermal correction to Enthalpy = 0.847188
Thermal correction to Gibbs Free Energy = 0.707871

Fe	-1.49968000	-3.27394600	0.48413700
P	-2.01836700	0.11575100	0.35398900
P	1.07801800	-1.34105600	-0.40646300
C	-3.19454500	0.03104000	-1.05892400
C	-2.79738700	1.21820700	1.60785500
C	-2.68074600	-0.09948500	-2.36555500
H	-1.58879100	-0.07131300	-2.52314900
C	0.16247700	-2.90858500	-0.57812700
C	1.60248700	-0.67954000	2.26367300
H	0.86074600	0.11722500	2.09068900
C	3.15457300	-1.84214300	3.72668100
H	3.62002800	-1.96801700	4.71573600
C	-2.27141700	-1.53233400	1.12661400
C	-3.06750600	1.86092700	3.94620500

H	-2.84465200	1.68830600	5.00973900
C	-4.59033600	0.03422800	-0.86037900
H	-5.00479600	0.13585900	0.15356200
C	2.40564800	-1.52720400	-1.67857300
C	-3.90809400	2.91782500	3.56391000
H	-4.34658700	3.57837900	4.32668200
C	-4.93832800	-0.23304100	-3.25563400
H	-5.62081700	-0.33329700	-4.11276500
C	1.95623800	-1.52962100	1.19676400
C	2.94061500	-2.52033800	1.39924100
H	3.24527700	-3.17270300	0.56699900
C	-0.97382600	-3.09427400	-1.46089600
H	-1.38577300	-2.34783300	-2.14939400
C	2.18950400	-0.84320300	3.52662900
H	1.90379000	-0.17599200	4.35262200
C	-3.63035800	2.29071200	1.22754700
H	-3.83494900	2.47616600	0.16415500
C	-4.19083800	3.12643000	2.20474300
H	-4.85032000	3.95229600	1.89825100
C	-5.45591900	-0.09496400	-1.95772000
H	-6.54419000	-0.08833300	-1.79542800
C	0.31959400	-4.12880500	0.18620200
H	1.05990200	-4.30350900	0.97448000
C	-3.17766800	-3.62738000	1.62490100
H	-3.80502600	-4.52629200	1.58705300
C	-2.51830100	1.01051500	2.97509600
H	-1.87951500	0.17331600	3.29079500
C	-0.70908500	-5.04126200	-0.22019200
H	-0.88132700	-6.03862100	0.20254500
C	3.53641100	-2.67272000	2.66009800
H	4.30167800	-3.44882200	2.81162400
C	4.38403000	-1.74127700	-3.68288400
H	5.15686800	-1.83640900	-4.46018900
C	3.06315700	-2.14108100	-3.94731700
H	2.79811800	-2.55002800	-4.93393100
C	2.07901400	-2.03401200	-2.95347200
H	1.05166500	-2.36187300	-3.17052200
C	-3.54893300	-0.23524800	-3.45861900
H	-3.13842000	-0.33268500	-4.47478800
C	4.71327800	-1.22616200	-2.41832700
H	5.74688800	-0.91663000	-2.20019600
C	-1.46836600	-2.14828500	2.16510200
H	-0.56190600	-1.73376800	2.61884100
C	3.72843400	-1.11583300	-1.42345800

H	3.99371500	-0.72894100	-0.43069900
C	-3.33395700	-2.46642000	0.79929700
H	-4.09708700	-2.32472000	0.02698300
C	-1.50275300	-4.40777900	-1.23328000
H	-2.38544500	-4.83571900	-1.72368900
C	-2.03192700	-3.43335700	2.46381500
H	-1.62744600	-4.15569700	3.18320700
Ni	-0.09449800	0.61097600	-0.54936400
C	2.71202500	1.96207200	0.01739000
C	2.83625800	2.59299400	-1.13706800
H	3.51502400	3.44573700	-1.33746500
S	1.77474800	2.25556800	-2.54766900
O	1.13839800	3.51405200	-2.98735600
O	0.93379500	1.02764200	-2.21988000
C	2.87301700	1.68175800	-3.84475300
H	2.21583800	1.47027200	-4.70924700
H	3.57163100	2.50416300	-4.08497200
H	3.39448700	0.76875900	-3.50444100
C	-0.87321600	2.38199900	-0.49701700
C	-0.39167900	3.21052900	0.67083100
C	-1.57079800	2.90985500	-1.52462000
C	-1.01952200	4.61811200	0.70032200
H	0.71139000	3.31113800	0.59021300
H	-0.58231000	2.68478500	1.63181400
C	-1.85568500	4.38745300	-1.66373400
H	-1.93215500	2.26945100	-2.34476800
C	-1.02409000	5.23662000	-0.69881900
H	-0.46156900	5.25449700	1.41844600
H	-2.05900300	4.54463700	1.08270700
H	-1.65854200	4.68659900	-2.71507100
H	-2.94665900	4.56261600	-1.50514100
H	-1.40697000	6.27708900	-0.66950600
H	0.01746100	5.28653400	-1.08361900
C	3.29427500	1.88913200	1.30527200
C	2.70140600	2.52035900	2.44170500
C	4.51608000	1.17027300	1.49204100
C	3.32006500	2.45138300	3.68997800
H	1.76033500	3.07104300	2.31927000
C	5.11155100	1.09775500	2.75105000
H	4.99748800	0.69764000	0.62504600
C	4.52117000	1.73686400	3.85680300
H	2.86057000	2.96263800	4.54916800
H	6.05563500	0.54625500	2.87223400
H	4.99891500	1.68190200	4.84606500

P2-3ETS:

Eel = -5897.227780
Zero-point correction = 0.793562
Thermal correction to Energy = 0.846417
Thermal correction to Enthalpy = 0.847361
Thermal correction to Gibbs Free Energy = 0.705716

Fe	-0.73586400	-0.28806900	2.72160500
P	1.91825600	-0.72365200	0.56883200
P	-1.17294200	1.74419300	-0.13517200
C	3.16075500	0.45658300	1.22861600
C	2.88534300	-2.13964500	-0.04271300
C	2.72939400	1.51162700	2.06084600
H	1.66936600	1.56932600	2.34462200
C	-1.56260000	1.29718200	1.59696100
C	-2.88562100	1.46545900	-2.32501200
H	-1.98346600	1.10871100	-2.84511300
C	-5.25531800	2.00834600	-2.33986400
H	-6.21394200	2.07600900	-2.87561400
C	0.98800000	-1.26596100	2.02925300
C	3.72178900	-4.41001300	0.15986600
H	3.77670300	-5.35778900	0.71569200
C	4.52135200	0.37246200	0.87542300
H	4.87756300	-0.44979600	0.23913400
C	-0.62377100	3.49712100	-0.14849400
C	4.40780600	-4.26535000	-1.05636600
H	5.00116700	-5.10184500	-1.45489400
C	4.99445300	2.38556300	2.16526700
H	5.71128900	3.13721100	2.52787600
C	-2.80789100	1.82870000	-0.96457100
C	-3.95990000	2.29227200	-0.29754100
H	-3.90323700	2.58802700	0.76070100
C	-0.94332800	1.76591700	2.81623000
H	-0.17222400	2.53875500	2.89717000
C	-4.10550800	1.56152000	-3.01207200
H	-4.16050600	1.27982900	-4.07407400
C	3.56129700	-1.99937800	-1.27296900
H	3.47518100	-1.06311000	-1.84477500
C	4.32885000	-3.05952700	-1.77086600
H	4.85185400	-2.94975300	-2.73218900
C	5.42931200	1.33663000	1.34070300
H	6.48994600	1.26107900	1.05808900

C	-2.55280300	0.31176800	1.96998000
H	-3.19131900	-0.24842200	1.28158400
C	0.08131700	-1.49309900	4.15566400
H	-0.08358200	-1.38489900	5.23459500
C	2.96323700	-3.34922800	0.67370100
H	2.42530400	-3.46570300	1.62539700
C	-2.58684600	0.22970500	3.39938200
H	-3.23929500	-0.42838900	3.98578900
C	-5.18097900	2.37282000	-0.98441200
H	-6.07998700	2.72879300	-0.45918200
C	0.34196100	6.14669000	-0.30888800
H	0.71607700	7.17942400	-0.37080500
C	0.97558500	5.21506400	0.52858100
H	1.85368100	5.50930500	1.12316700
C	0.50216500	3.89824100	0.60060700
H	1.03142600	3.17541400	1.23269300
C	3.64112900	2.46611600	2.53227400
H	3.29250200	3.27891800	3.18775400
C	-0.76892000	5.75200900	-1.06969900
H	-1.27101700	6.47489500	-1.72993800
C	-0.17824000	-2.11875600	1.93655900
H	-0.50941200	-2.61660700	1.02160800
C	-1.25200200	4.43668900	-0.99308900
H	-2.12493200	4.14581300	-1.59432800
C	1.12730000	-0.86109700	3.40698700
H	1.91057500	-0.21430100	3.81510300
C	-1.59234500	1.12300200	3.92197100
H	-1.35373800	1.28077200	4.98081100
C	-0.72013700	-2.26947400	3.25065500
H	-1.60941000	-2.85616500	3.51087000
Ni	0.22558100	0.07122100	-0.55402200
C	-1.93009600	-1.36690000	-1.12242600
C	-0.75447300	-1.57386300	-1.72947900
H	-0.30760800	-0.86201100	-2.45200200
S	0.00841800	-3.23233700	-2.00742400
O	0.10295000	-3.94573100	-0.70211200
O	1.21365100	-3.00086500	-2.84123500
C	-1.20202800	-4.11922000	-3.01500500
H	-0.77235000	-5.12786700	-3.16505000
H	-2.15876400	-4.17740600	-2.46354100
H	-1.31055400	-3.58971400	-3.97841300
C	1.45710200	1.03549100	-1.69265600
C	1.36704200	0.53271800	-3.12004000
C	2.25102200	2.06240300	-1.33611800

C	2.48075000	1.13197500	-4.00540100
H	0.37048400	0.79510300	-3.54899200
H	1.44616800	-0.57531800	-3.16014300
C	3.02966300	2.90708200	-2.32072600
H	2.36194900	2.35063600	-0.28293100
C	2.62673400	2.63470500	-3.77117700
H	2.25939900	0.90247600	-5.06781200
H	3.44149400	0.62503400	-3.76563100
H	2.88485600	3.97864500	-2.06190400
H	4.11783100	2.71300400	-2.17163400
H	3.36891300	3.07273800	-4.46915200
H	1.65622000	3.13406400	-3.98544700
C	-3.05096300	-1.99666800	-0.48819700
C	-2.94599500	-3.25461500	0.17480300
C	-4.32324500	-1.36090400	-0.54173100
C	-4.06903600	-3.82409300	0.78484600
H	-1.98166300	-3.78224200	0.18740200
C	-5.43878900	-1.94978200	0.06220900
H	-4.41903500	-0.40248800	-1.06784400
C	-5.31513400	-3.17523800	0.73840200
H	-3.97180200	-4.79464500	1.29467300
H	-6.41461200	-1.44476700	0.00640600
H	-6.19283900	-3.63138400	1.22019200

P3Z:

Eel = -5897.296752
Zero-point correction = 0.795807
Thermal correction to Energy = 0.848437
Thermal correction to Enthalpy = 0.849382
Thermal correction to Gibbs Free Energy = 0.709260

Fe	-1.03578600	-3.44319700	0.21121800
P	-1.96314300	-0.15045900	0.14758900
P	1.49613100	-1.16561700	0.20845700
C	-3.10641100	-0.10101800	-1.29982000
C	-2.90828900	0.79399900	1.42836300
C	-2.64267000	0.10524200	-2.61197400
H	-1.57321100	0.25186600	-2.79823900
C	0.78036500	-2.76404000	-0.27481900
C	1.01516700	-0.53705300	2.89104500
H	0.23176700	0.12320000	2.48144500
C	2.23156400	-1.42682200	4.79484000
H	2.39651600	-1.47925300	5.88139500

C	-2.20407500	-1.88333500	0.71751600
C	-3.46502400	1.22926400	3.76582600
H	-3.34903900	0.97756400	4.83067000
C	-4.48756700	-0.31366000	-1.07785500
H	-4.86527700	-0.48211300	-0.05858000
C	3.17636300	-1.29131200	-0.54686900
C	-4.32169700	2.27073500	3.37636800
H	-4.87887000	2.84133700	4.13411700
C	-4.91641800	-0.09485500	-3.46209500
H	-5.62316100	-0.08710200	-4.30546600
C	1.81642500	-1.29701600	2.01270600
C	2.84559600	-2.10478000	2.53741100
H	3.49944800	-2.67190600	1.85730400
C	-0.00883600	-2.98113000	-1.47313800
H	-0.28013700	-2.20442000	-2.19724300
C	1.21797300	-0.60371200	4.27729400
H	0.58926300	-0.00415500	4.95273300
C	-3.76079500	1.85003200	1.04432400
H	-3.87342900	2.10594300	-0.01840700
C	-4.46946300	2.57434600	2.01462700
H	-5.14178200	3.38678500	1.70006400
C	-5.38383300	-0.31080100	-2.15438300
H	-6.45575300	-0.47649600	-1.96923500
C	0.89399700	-4.03388700	0.41340800
H	1.40374900	-4.20295100	1.36911300
C	-2.90145500	-4.11277700	0.77733900
H	-3.35959200	-5.06829900	0.49434100
C	-2.76539300	0.49189500	2.79940400
H	-2.12092700	-0.33478300	3.12541400
C	0.17241700	-5.01187600	-0.34932900
H	0.03435100	-6.06412600	-0.07193900
C	3.04548400	-2.17161200	3.92477700
H	3.84855900	-2.80518900	4.33016100
C	5.73934200	-1.45210300	-1.72258100
H	6.73839600	-1.51483000	-2.17928200
C	4.77003400	-2.42520000	-2.01076700
H	5.00643000	-3.25747800	-2.69073700
C	3.49498000	-2.34891300	-1.42616000
H	2.74602300	-3.12035400	-1.65450400
C	-3.54797500	0.10982200	-3.68611100
H	-3.17205800	0.28030400	-4.70607900
C	5.43161300	-0.40437800	-0.83914200
H	6.18835300	0.35743900	-0.59891800
C	-1.63924200	-2.51477800	1.89639700

H	-0.95410600	-2.05983400	2.61963300
C	4.15954700	-0.32149300	-0.25625100
H	3.94055600	0.50197300	0.43677100
C	-2.98464100	-2.89359300	0.02912900
H	-3.51677000	-2.75272200	-0.91749400
C	-0.37969100	-4.36625500	-1.50685100
H	-1.01166800	-4.84081600	-2.26745300
C	-2.07863100	-3.88028500	1.92702700
H	-1.79465500	-4.62487100	2.68063200
Ni	0.20423900	0.72119000	-0.26560400
C	1.74418000	1.85812200	-0.66439100
C	2.21232000	1.84882200	-1.94269800
H	3.05683400	2.43760800	-2.33644000
S	1.29101600	0.95508600	-3.13119100
O	0.60468800	1.81092000	-4.12549100
O	0.40609500	0.02955300	-2.27779000
C	2.42009200	-0.11932100	-4.01898400
H	1.78717900	-0.71682400	-4.70193200
H	3.10283300	0.52685200	-4.60118100
H	2.96496600	-0.75817800	-3.30068900
C	-0.67503100	2.47326700	-0.33482700
C	-0.69302300	3.15601100	1.01172900
C	-1.14241100	3.03328700	-1.46815000
C	-1.51893600	4.46313000	0.99544600
H	0.34594600	3.38718800	1.31997000
H	-1.08779500	2.48093900	1.80129700
C	-1.70808400	4.43664200	-1.51220900
H	-1.08102300	2.50260300	-2.43043500
C	-1.28778100	5.25712000	-0.28982400
H	-1.25306000	5.06075500	1.89155700
H	-2.59630800	4.21948400	1.08621900
H	-1.37895800	4.92509300	-2.45403900
H	-2.82018100	4.38508600	-1.58551000
H	-1.84271000	6.21722300	-0.25845500
H	-0.20943800	5.51580800	-0.37616500
C	2.37769800	2.75353500	0.31820600
C	2.65860000	2.33336600	1.64070000
C	2.69033300	4.08783800	-0.04478000
C	3.25906200	3.20464400	2.55808700
H	2.39733000	1.31397600	1.95399900
C	3.27579100	4.96106300	0.88023300
H	2.43506800	4.44135800	-1.05466500
C	3.56745400	4.52243900	2.18298200
H	3.47747100	2.85312700	3.57761200

H	3.50233300	5.99626300	0.58375000
H	4.02679500	5.21100500	2.90768300

P3E:

Eel = -5897.274485
Zero-point correction = 0.794893
Thermal correction to Energy = 0.848242
Thermal correction to Enthalpy = 0.849186
Thermal correction to Gibbs Free Energy = 0.704586

Fe	-1.90096400	-2.34509500	-1.74450400
P	0.69696400	-1.50410700	0.49528000
P	-1.99531700	0.81005600	-0.30146100
C	0.26767000	-2.18197300	2.15740700
C	2.44892000	-2.03413800	0.28467700
C	-0.57074700	-1.48948400	3.04999200
H	-0.94712600	-0.49447200	2.78715900
C	-2.63248300	-0.47976100	-1.42491700
C	-1.89267500	3.51400400	-1.20694400
H	-0.90035100	3.56599200	-0.73912300
C	-3.68454000	4.60069400	-2.43907000
H	-4.09149100	5.48819800	-2.94620800
C	-0.20973500	-2.62629200	-0.62005500
C	4.18261200	-3.44801000	-0.68457000
H	4.46556500	-4.30686300	-1.31165200
C	0.78264700	-3.44562000	2.52376200
H	1.45158700	-3.98943300	1.84026000
C	-3.06880200	0.68552400	1.18790300
C	5.17364400	-2.66923500	-0.06632100
H	6.23714700	-2.90323800	-0.22298900
C	-0.39703900	-3.31503300	4.64848400
H	-0.65231000	-3.75649500	5.62352600
C	-2.64168800	2.32340400	-1.14460200
C	-3.92273600	2.28263200	-1.73757100
H	-4.51820100	1.35818500	-1.69863900
C	-3.68027000	-1.45536000	-1.22774700
H	-4.26571800	-1.60203600	-0.31446900
C	-2.41184500	4.64836600	-1.85167800
H	-1.81657200	5.57269300	-1.89389800
C	3.45058800	-1.28585000	0.93974200
H	3.17890300	-0.43382400	1.57514100
C	4.80548800	-1.59120900	0.75514700
H	5.56758000	-0.95300900	1.22372200

C	0.44702200	-4.00720600	3.76312900
H	0.85349700	-4.99095300	4.04156900
C	-2.14334600	-0.62504100	-2.77873500
H	-1.33390000	-0.04036400	-3.23028100
C	-1.58104900	-4.33693600	-1.41617500
H	-2.33444000	-5.13259900	-1.46485300
C	2.82527000	-3.13371400	-0.51705600
H	2.06300700	-3.74743000	-1.01540200
C	-2.88591500	-1.68200700	-3.40364600
H	-2.73594600	-2.05451100	-4.42425400
C	-4.43872100	3.41645300	-2.37904100
H	-5.43799400	3.37465400	-2.83766800
C	-4.62002700	0.50992800	3.53779400
H	-5.22814300	0.44134200	4.45203800
C	-4.04463200	-0.64528600	2.98550200
H	-4.19462200	-1.62397800	3.46555700
C	-3.26513500	-0.55906200	1.82264800
H	-2.80406300	-1.46770800	1.41094300
C	-0.90450500	-2.05752700	4.28953400
H	-1.56316500	-1.50538500	4.97691400
C	-4.42360300	1.75207300	2.91386300
H	-4.87961200	2.66043600	3.33511800
C	0.04482200	-2.78754300	-2.03686400
H	0.75132100	-2.19367300	-2.62868700
C	-3.65884800	1.84175400	1.74087800
H	-3.53847800	2.81517100	1.24274700
C	-1.24047200	-3.57353900	-0.24851900
H	-1.64831900	-3.70507100	0.76053300
C	-3.82386400	-2.19853200	-2.44606900
H	-4.52001100	-3.03035600	-2.60898300
C	-0.78400400	-3.86106000	-2.51298300
H	-0.82981900	-4.22394800	-3.54708700
Ni	0.31501400	0.77009600	0.06557200
C	2.08209600	1.37778200	-0.30293100
C	3.10120100	1.93113200	0.37014700
H	3.08467100	2.11390800	1.45506400
S	4.73859200	2.20434300	-0.33703800
O	4.74828300	1.85950200	-1.77766700
O	5.66639900	1.54767600	0.62092800
C	4.99425100	3.98776200	-0.20051100
H	6.03053600	4.15777600	-0.55024700
H	4.26967900	4.50359700	-0.85631500
H	4.89044400	4.29139800	0.85718100
C	0.48241300	1.67538100	1.75551700

C	-0.11404700	3.05941500	1.76895000
C	1.05851700	1.11143400	2.83516700
C	0.17914300	3.79832100	3.09399500
H	-1.21131600	3.00747700	1.60065000
H	0.30025700	3.65292100	0.92587700
C	0.96982800	1.70412400	4.22460300
H	1.58333600	0.14664300	2.76034800
C	-0.01910000	2.87023400	4.29237600
H	-0.46993500	4.69523800	3.16052700
H	1.22951700	4.16285000	3.08131400
H	0.69051400	0.90042300	4.94052200
H	1.98749900	2.03420500	4.53980800
H	0.10115400	3.42429600	5.24526400
H	-1.05972200	2.47622800	4.28126200
C	1.90853500	0.99514900	-1.70597900
C	2.64106300	-0.06721900	-2.30985700
C	0.92087600	1.66929600	-2.48083500
C	2.39478100	-0.41955200	-3.63757400
H	3.43077800	-0.56687500	-1.73705600
C	0.68211400	1.30183700	-3.81573900
H	0.39907900	2.53697500	-2.05397600
C	1.41421000	0.25596400	-4.39331600
H	2.99264000	-1.21780000	-4.10243800
H	-0.06120500	1.85778700	-4.40646500
H	1.24148500	-0.02353200	-5.44348100

P3-4ZTS:

Eel = -5897.294314
Zero-point correction = 0.795148
Thermal correction to Energy = 0.847088
Thermal correction to Enthalpy = 0.848032
Thermal correction to Gibbs Free Energy = 0.709998

Fe	1.57541200	-2.99430300	-0.99334000
P	2.07308500	0.19693900	-0.11504900
P	-1.29073000	-1.44056100	-0.14123900
C	3.24231300	-0.11246900	1.28645000
C	2.95118700	1.60937500	-0.92106100
C	2.81043400	-0.32863200	2.60799900
H	1.74020800	-0.28242400	2.84060400
C	-0.19700300	-2.88901000	-0.05525300
C	-1.83729100	-0.78457200	-2.82915500
H	-1.11993900	0.03804600	-2.68143500

C	-3.41655100	-1.96084700	-4.25148100
H	-3.91448300	-2.08453700	-5.22479900
C	2.42861800	-1.19302100	-1.24380000
C	3.95042000	2.69753900	-2.85961900
H	4.18721500	2.69213800	-3.93411600
C	4.62865100	-0.11263200	1.00271300
H	4.98384700	0.09421200	-0.01779100
C	-2.60060200	-1.85502700	1.08874300
C	4.36299800	3.76949600	-2.05297300
H	4.92290100	4.60781000	-2.49346800
C	5.12046500	-0.59135200	3.33611800
H	5.85280400	-0.77749400	4.13599200
C	-2.14596300	-1.64596100	-1.75679000
C	-3.11140400	-2.65836600	-1.93910200
H	-3.37689800	-3.32054300	-1.10101300
C	0.87612400	-3.03183800	0.90860300
H	1.12561100	-2.31375500	1.69810500
C	-2.46665300	-0.94263200	-4.07293600
H	-2.22105600	-0.26204900	-4.90176800
C	3.35680400	2.69469000	-0.11531100
H	3.14122600	2.68682000	0.96376300
C	4.06548700	3.76487800	-0.67942800
H	4.39588400	4.59775700	-0.04035900
C	5.55825700	-0.35934800	2.02090700
H	6.63353900	-0.36021500	1.78707400
C	-0.16202000	-4.04544900	-0.92777700
H	-0.82732500	-4.22594600	-1.77977700
C	3.24035000	-3.16732500	-2.18792600
H	3.82648600	-4.08000900	-2.35090400
C	3.24866100	1.61779800	-2.29769300
H	2.95443400	0.76912900	-2.93282600
C	0.92449700	-4.88141000	-0.50245000
H	1.23639900	-5.81755000	-0.98145200
C	-3.74006300	-2.81418500	-3.18337600
H	-4.49255200	-3.60549600	-3.31854500
C	-4.61012800	-2.43891900	2.98297400
H	-5.39578700	-2.67028600	3.71773400
C	-3.44411800	-3.22013400	2.92770800
H	-3.31384300	-4.06785100	3.61711000
C	-2.44277300	-2.93173500	1.98642400
H	-1.53770700	-3.55427400	1.94566600
C	3.74955900	-0.56723000	3.62579600
H	3.40050200	-0.72537800	4.65733300
C	-4.77305000	-1.36724200	2.08950900

H	-5.68709800	-0.75529300	2.11985600
C	1.60816500	-1.52813800	-2.38993800
H	0.73484000	-0.97005000	-2.74243400
C	-3.77390400	-1.07194300	1.15010700
H	-3.92136300	-0.23659800	0.45226300
C	3.44507500	-2.22079300	-1.12902900
H	4.20471800	-2.28577200	-0.34269500
C	1.56015100	-4.26025000	0.62526400
H	2.44198300	-4.63728000	1.15736800
C	2.11132300	-2.74194300	-2.96414800
H	1.68266300	-3.26762900	-3.82590500
Ni	-0.26333200	0.59812300	0.25000000
C	-1.83975700	1.73454700	0.73279100
C	-2.15512900	1.62466300	2.06941500
H	-3.06482200	2.03097300	2.53765400
S	-0.98234000	0.93265100	3.14819100
O	-0.18474400	1.91572300	3.92027100
O	-0.18841900	-0.02390000	2.23485200
C	-1.85647600	-0.11013200	4.31466600
H	-1.07208200	-0.58470800	4.93364300
H	-2.48500800	0.54929500	4.94135300
H	-2.45510500	-0.86081800	3.76920900
C	-0.14587300	2.54937400	0.06789100
C	-0.16645900	2.69378000	-1.43987800
C	0.21729300	3.54492400	0.91359000
C	0.52126100	3.99120800	-1.91854100
H	-1.21749500	2.67074600	-1.79437500
H	0.33530200	1.82262800	-1.92931300
C	0.61500700	4.91407200	0.42899400
H	0.17456700	3.37265300	2.00249400
C	0.16077300	5.16987700	-1.01293600
H	0.21713300	4.18644700	-2.96690300
H	1.61879600	3.85201400	-1.92618200
H	0.19944900	5.67705400	1.12174400
H	1.72175200	5.02116100	0.51069400
H	0.61973200	6.10308700	-1.39746800
H	-0.93993900	5.32640300	-1.02689200
C	-2.88251900	2.39126700	-0.10378200
C	-3.50762900	1.73753800	-1.18686300
C	-3.27429300	3.71469900	0.20474200
C	-4.50706700	2.38226100	-1.93106900
H	-3.20784100	0.71705900	-1.45527600
C	-4.26640900	4.35989300	-0.54514800
H	-2.77228200	4.23866200	1.03257800

C	-4.88795800	3.69530800	-1.61578900
H	-4.98506000	1.85088100	-2.76777900
H	-4.55535600	5.39142400	-0.29344400
H	-5.66562400	4.20340500	-2.20511700

P3-4ETS:

Eel = -5897.271689
Zero-point correction = 0.794743
Thermal correction to Energy = 0.847284
Thermal correction to Enthalpy = 0.848228
Thermal correction to Gibbs Free Energy = 0.706681

Fe	-2.44766400	-1.36435200	-2.21718100
P	0.40262400	-1.77072100	-0.18615500
P	-1.76522900	1.10562600	0.09790600
C	0.05577500	-2.81958400	1.29481600
C	1.91205000	-2.52203900	-0.94789100
C	-0.64972700	-2.26091200	2.37817100
H	-0.92688900	-1.20025700	2.35751700
C	-2.65877000	0.45068900	-1.34506000
C	-1.35965700	3.81925600	0.78062200
H	-0.70098200	3.42925000	1.56720200
C	-2.34918600	5.71843000	-0.36964100
H	-2.47668300	6.80553700	-0.48111400
C	-0.86808800	-2.34731800	-1.37169100
C	2.98565000	-4.29332800	-2.24114700
H	2.90677400	-5.22803100	-2.81667300
C	0.45882700	-4.16953900	1.35557600
H	1.03320900	-4.61652100	0.53195400
C	-2.84560700	0.66102800	1.52763200
C	4.23551700	-3.67503300	-2.06736900
H	5.13734300	-4.12211100	-2.51185000
C	-0.58359900	-4.39167500	3.54605500
H	-0.83133900	-5.00696700	4.42399900
C	-2.02133800	2.92765600	-0.08987800
C	-2.85305900	3.44965600	-1.10087700
H	-3.38258900	2.77356200	-1.78615800
C	-3.94620900	-0.20286800	-1.40941400
H	-4.58992500	-0.44277000	-0.55660200
C	-1.52921800	5.20449600	0.64786600
H	-1.01233500	5.88652300	1.33975200
C	3.17172000	-1.91582200	-0.77576400
H	3.26525500	-0.97431800	-0.22478100

C	4.32656600	-2.48133200	-1.33721700
H	5.28367500	-1.95554300	-1.20451900
C	0.14012500	-4.94805000	2.47861400
H	0.46316200	-5.99906800	2.51869400
C	-2.15821000	0.57196500	-2.69858500
H	-1.20229100	1.02632600	-2.98206400
C	-2.71936000	-3.39144200	-2.33223200
H	-3.67643200	-3.91379000	-2.45119500
C	1.82763900	-3.72125500	-1.69535900
H	0.85602900	-4.20875200	-1.85952300
C	-3.13303500	-0.00663200	-3.57961700
H	-3.03997600	-0.09678900	-4.66876500
C	-3.00866800	4.83783200	-1.24055800
H	-3.65805300	5.23213100	-2.03654600
C	-4.40278300	-0.10153500	3.75748300
H	-5.01120100	-0.39683900	4.62522400
C	-4.05232800	-1.05091900	2.78451500
H	-4.38153300	-2.09610800	2.88503700
C	-3.27239400	-0.67573900	1.68151700
H	-2.99637000	-1.42733600	0.92987600
C	-0.97915400	-3.04586700	3.49270100
H	-1.54097600	-2.59544700	4.32509800
C	-3.98032800	1.22881500	3.61186600
H	-4.26140300	1.98303800	4.36203300
C	-0.74123100	-2.26408700	-2.81222200
H	0.08791700	-1.79447600	-3.35477000
C	-3.21141200	1.61291500	2.50229400
H	-2.91610400	2.66547700	2.38913000
C	-2.10480100	-3.03811800	-1.08312400
H	-2.47534300	-3.28855400	-0.08187500
C	-4.22592600	-0.49253700	-2.78396800
H	-5.11360900	-1.01632700	-3.15920400
C	-1.87355200	-2.92689000	-3.39597900
H	-2.07636400	-3.02301900	-4.46971700
Ni	0.46146500	0.48390400	0.26897800
C	2.28327300	1.17054500	0.33096500
C	3.55434400	1.19039200	0.77274700
H	3.89746000	0.69884400	1.69517000
S	4.92923400	1.67010500	-0.29913500
O	4.44540200	2.41943300	-1.47956400
O	5.71018000	0.41307600	-0.45135200
C	5.91268700	2.79570200	0.71254800
H	6.81117800	3.01618900	0.10507900
H	5.32911000	3.71605400	0.89597100

H	6.19929400	2.28783600	1.65152300
C	1.24746700	0.53968500	2.06546500
C	0.69934000	1.67710600	2.89572000
C	1.87697500	-0.52713400	2.61339200
C	1.20181900	1.60938800	4.35230500
H	-0.41354300	1.64989500	2.88717400
H	0.99229000	2.64788900	2.44380300
C	1.94273500	-0.78192000	4.09691000
H	2.36293400	-1.27707200	1.96799200
C	1.07165700	0.18753200	4.90160400
H	0.63412700	2.33465000	4.96992200
H	2.26780200	1.92387800	4.38471000
H	1.65224600	-1.83657000	4.29533600
H	3.00890100	-0.71025200	4.41875200
H	1.34558600	0.15122500	5.97507800
H	0.00750300	-0.13085500	4.83645400
C	1.74102400	1.82488800	-0.87827100
C	1.63988500	1.10751500	-2.10689400
C	1.32920200	3.18594400	-0.84207200
C	1.14846100	1.74832200	-3.25193900
H	2.02909200	0.08155600	-2.16570700
C	0.82434800	3.80460800	-1.98792300
H	1.43086000	3.74845400	0.09593300
C	0.73436200	3.09008600	-3.19498200
H	1.11041500	1.19900000	-4.20468900
H	0.50220500	4.85513500	-1.94136600
H	0.35162000	3.58656800	-4.09897700

P4:

Eel = -4766.539124

Zero-point correction = 0.505229

Thermal correction to Energy = 0.539465

Thermal correction to Enthalpy = 0.540409

Thermal correction to Gibbs Free Energy = 0.434596

Fe	0.00008300	0.00019200	2.33501900
P	-1.84978000	0.05065600	-0.48383300
P	1.84973500	-0.05070900	-0.48396000
C	-2.67439000	-1.58040700	-0.34571800
C	-3.13998300	1.16193400	-1.16677300
C	-1.98454500	-2.74322500	-0.74429600
H	-0.96591100	-2.65534600	-1.15853400
C	1.57201700	-0.60510200	1.21880200

C	1.98456800	2.74313300	-0.74483500
H	0.96589300	2.65523300	-1.15896600
C	3.90327300	4.09637900	-0.12049700
H	4.38676100	5.08078900	-0.03254200
C	-1.57192800	0.60531500	1.21882000
C	-4.55763800	3.13593900	-1.06219000
H	-4.91228800	4.02452000	-0.51881300
C	-3.99236700	-1.67872500	0.14810000
H	-4.54518600	-0.76961600	0.43119700
C	3.13984500	-1.16212900	-1.16684800
C	-5.07813500	2.83207100	-2.32979900
H	-5.83798100	3.48563000	-2.78349100
C	-3.90314600	-4.09644900	-0.11959000
H	-4.38659100	-5.08086200	-0.03145100
C	2.67441000	1.58034800	-0.34615600
C	3.99243500	1.67869100	0.14752900
H	4.54524700	0.76960300	0.43070600
C	0.74418600	-1.73604100	1.58951500
H	0.22066000	-2.40690000	0.89858000
C	2.59867100	3.99962700	-0.62970900
H	2.05970800	4.90497700	-0.94631600
C	-3.66272500	0.85934600	-2.44394700
H	-3.32269000	-0.04023500	-2.98274700
C	-4.63110500	1.69164700	-3.01942700
H	-5.04145000	1.44914100	-4.01099500
C	-4.59920800	-2.93729500	0.26466500
H	-5.62658600	-3.01404300	0.65098600
C	2.03311700	0.02148600	2.44046800
H	2.65607200	0.92128300	2.50197500
C	-1.49988000	0.72643900	3.54261500
H	-1.64168100	0.48837500	4.60393800
C	-3.58789800	2.30633600	-0.47731800
H	-3.18866300	2.54403700	0.51979000
C	1.50013900	-0.72585100	3.54262200
H	1.64201600	-0.48761500	4.60389600
C	4.59933100	2.93725600	0.26386000
H	5.62674600	3.01402400	0.65007600
C	5.07784900	-2.83249000	-2.32979700
H	5.83763600	-3.48613700	-2.78346000
C	4.55749500	-3.13612700	-1.06207400
H	4.91219900	-4.02461400	-0.51858000
C	3.58782900	-2.30641100	-0.47723900
H	3.18870600	-2.54393100	0.51995700
C	-2.59859300	-3.99972400	-0.62893600

H	-2.05962800	-4.90510000	-0.94546300
C	4.63074900	-1.69218600	-3.01957800
H	5.04098200	-1.44986100	-4.01123600
C	-0.74406100	1.73630800	1.58929000
H	-0.22057900	2.40704900	0.89820900
C	3.66244200	-0.85977400	-2.44413500
H	3.32235300	0.03971400	-2.98305700
C	-2.03294400	-0.02107100	2.44062100
H	-2.65590200	-0.92085300	2.50231800
C	0.71127400	-1.80522300	3.02103700
H	0.14678600	-2.53578600	3.61312400
C	-0.71104500	1.80572000	3.02079900
H	-0.14650600	2.53637300	3.61272700
Ni	-0.00006500	-0.00008900	-1.65659700

P2-6ZTS:

Eel = -5308.752798
Zero-point correction = 0.747087
Thermal correction to Energy = 0.794030
Thermal correction to Enthalpy = 0.794974
Thermal correction to Gibbs Free Energy = 0.666135

Fe	2.29285000	-1.37024300	-2.06217200
P	-0.58672400	-1.37143700	-0.28009100
P	1.89207600	0.98826800	0.28534900
C	-1.81087300	-1.35070000	-1.65297300
C	-1.22313700	-2.62515300	0.92240100
C	-2.15671400	-0.11703000	-2.23397000
H	-1.72890800	0.80686700	-1.81610700
C	2.50817900	0.47829900	-1.34472600
C	2.04605100	-0.68371600	2.52371100
H	0.94709400	-0.66996700	2.46795700
C	4.09833400	-1.42091700	3.59410400
H	4.61198300	-1.99698700	4.37821300
C	0.80168900	-2.28123000	-1.07049300
C	-0.79639600	-4.14106200	2.79032900
H	-0.08477600	-4.60725900	3.48829100
C	-2.36641400	-2.54048100	-2.17062500
H	-2.09631600	-3.51385500	-1.73462400
C	2.61115900	2.67410000	0.46881100
C	-2.16055000	-4.46433700	2.85238300
H	-2.52607300	-5.18213100	3.60166900
C	-3.61045200	-1.24613500	-3.81690600

H	-4.31885900	-1.20769500	-4.65792300
C	2.79176300	0.04528800	1.57756300
C	4.19946100	0.07222000	1.67300000
H	4.79012000	0.69136800	0.98176800
C	1.67281100	0.49455200	-2.53249300
H	0.62650300	0.82150800	-2.57135500
C	2.69656400	-1.41796800	3.52765400
H	2.10380800	-1.98413600	4.26193000
C	-2.59122200	-2.95745000	0.98910300
H	-3.30165400	-2.50365600	0.28595500
C	-3.05397800	-3.87436000	1.94477900
H	-4.12408800	-4.12899300	1.97759500
C	-3.26287200	-2.48350100	-3.24800000
H	-3.69493500	-3.41393600	-3.64590600
C	3.79999100	-0.05479300	-1.72885600
H	4.65383900	-0.23045900	-1.06599700
C	2.12250000	-3.32647200	-2.68993300
H	2.46822700	-3.73335400	-3.64796000
C	-0.32888000	-3.23066300	1.83074700
H	0.74473800	-3.00573100	1.79079600
C	3.74671200	-0.36982300	-3.12743600
H	4.55666600	-0.82673700	-3.70907400
C	4.84683800	-0.66917600	2.67140600
H	5.94498200	-0.65174300	2.73775500
C	3.58670600	5.29813500	0.76569300
H	3.97183600	6.32214700	0.88158400
C	3.34607900	4.77803100	-0.51530900
H	3.54373800	5.39200700	-1.40666800
C	2.85754500	3.47072000	-0.66718600
H	2.67787000	3.06604200	-1.67442700
C	-3.05532600	-0.06142000	-3.30930200
H	-3.32939200	0.91330200	-3.73990000
C	3.33755800	4.50809400	1.90052600
H	3.52823700	4.91077500	2.90658800
C	2.08498000	-2.61045300	-0.47900700
H	2.41360600	-2.37548300	0.53869500
C	2.84907400	3.20154000	1.75615800
H	2.65729000	2.58681900	2.64927700
C	0.83987500	-2.73413800	-2.44925300
H	0.03973100	-2.61036000	-3.18641300
C	2.44244300	-0.03312800	-3.62129800
H	2.08211900	-0.18506800	-4.64584400
C	2.88668200	-3.25257200	-1.48004500
H	3.92104700	-3.59193400	-1.34678700

Ni	-0.34289500	0.74469200	0.47168700
C	-0.39829200	2.64975600	0.78821400
H	0.33638400	3.43100300	0.99591400
C	-1.60055400	2.37998500	0.41897200
C	-2.82004600	2.88101100	-0.19770300
C	-2.77259800	3.27400900	-1.55653000
C	-4.02093600	3.05436600	0.52606500
C	-3.91000800	3.80441200	-2.18023200
H	-1.82942100	3.16957800	-2.11337800
C	-5.15076900	3.59390100	-0.10057500
H	-4.05290100	2.77326200	1.58738600
C	-5.10277800	3.95993300	-1.45638800
H	-3.86136800	4.10655600	-3.23726000
H	-6.07767400	3.73439500	0.47522000
H	-5.99496100	4.37716100	-1.94651800
C	-2.06148600	0.43488000	1.38234800
C	-3.31734900	0.13615800	0.98351700
C	-1.63350600	0.36634100	2.82996300
C	-4.41529800	-0.27252700	1.93335100
H	-3.60633700	0.25949400	-0.07254600
C	-2.84854600	0.48583300	3.77186400
H	-1.12556600	-0.60841700	3.01127200
H	-0.88453600	1.15436400	3.05720900
C	-3.93273700	-0.51529800	3.36890800
H	-4.93800000	-1.17000200	1.53602600
H	-5.18887100	0.53134600	1.90547800
H	-2.52393800	0.31865100	4.81896100
H	-3.24499500	1.52367400	3.72326800
H	-4.79083600	-0.46983300	4.06974500
H	-3.51640400	-1.54245900	3.44652500

P2-8TS:

Eel = -5308.743543
Zero-point correction = 0.746695
Thermal correction to Energy = 0.793854
Thermal correction to Enthalpy = 0.794798
Thermal correction to Gibbs Free Energy = 0.665923

Fe	-1.58114700	-2.85909800	0.20805800
P	-2.09649700	0.39166400	-0.39561700
P	1.21960900	-0.91300100	0.43254500
C	-2.74320700	0.24317100	-2.11864200
C	-3.19786100	1.67573700	0.33146900

C	-1.86289500	0.10525100	-3.20970100
H	-0.77526600	0.14290000	-3.04793300
C	0.37282300	-2.45371400	-0.05198100
C	0.47131000	-0.22598300	3.06905600
H	-0.18220400	0.51795500	2.58870100
C	1.36890500	-1.26206300	5.07818600
H	1.39597000	-1.35014100	6.17459000
C	-2.67471900	-1.17103700	0.35457600
C	-4.32440400	2.64938700	2.26225300
H	-4.65418500	2.56905300	3.30903700
C	-4.13621200	0.23533100	-2.35256700
H	-4.83456900	0.35602000	-1.51060500
C	2.96181300	-1.27115500	-0.08019400
C	-4.61891000	3.80301400	1.51846600
H	-5.17441600	4.63198500	1.98158800
C	-3.74747000	-0.06699700	-4.73580500
H	-4.14014100	-0.18616900	-5.75667900
C	1.30466300	-1.03576300	2.27056500
C	2.18395600	-1.94898700	2.88961900
H	2.85754100	-2.56614500	2.27606600
C	-0.27846500	-2.65972600	-1.33079800
H	-0.35477200	-1.92625100	-2.14212000
C	0.50390000	-0.34016600	4.46712300
H	-0.14561900	0.30047600	5.08285100
C	-3.50639700	2.83375100	-0.41661900
H	-3.20127200	2.90521900	-1.47180000
C	-4.21368600	3.88971200	0.17612300
H	-4.45546900	4.78456000	-0.41685800
C	-4.63270400	0.08089900	-3.65443800
H	-5.71952900	0.07886200	-3.82649200
C	0.21306400	-3.67037100	0.71716000
H	0.56516000	-3.82980200	1.74273000
C	-3.54505400	-3.31974300	0.61938900
H	-4.03908300	-4.27662700	0.41098600
C	-3.61671300	1.58923300	1.67458500
H	-3.39728300	0.68782000	2.26510000
C	-0.52926300	-4.60321600	-0.08092700
H	-0.84615000	-5.60476200	0.23484700
C	2.20943000	-2.06297400	4.28789000
H	2.89608200	-2.77923500	4.76351200
C	5.61867300	-1.78688200	-0.88360100
H	6.65468100	-1.99033100	-1.19324300
C	4.56417200	-2.55328100	-1.40177700
H	4.76922500	-3.36523000	-2.11577800

C	3.24190600	-2.30000800	-1.00330300
H	2.42627400	-2.91855000	-1.40484100
C	-2.36275000	-0.05296700	-4.51228400
H	-1.66471900	-0.15799900	-5.35624900
C	5.34632000	-0.76396500	0.03899400
H	6.16745600	-0.16139700	0.45496000
C	-2.30584700	-1.65822000	1.66926200
H	-1.68800200	-1.13545700	2.40660800
C	4.02832400	-0.50549600	0.43747700
H	3.83547400	0.28720600	1.17234500
C	-3.44611900	-2.21564600	-0.28988300
H	-3.84770900	-2.17962100	-1.30835300
C	-0.83139200	-3.98359800	-1.33986600
H	-1.41515100	-4.42815400	-2.15501700
C	-2.84746100	-2.97681000	1.82437500
H	-2.71473100	-3.62238500	2.70098200
Ni	0.19040400	0.92029800	-0.47447300
C	1.32158500	1.85971400	-1.75905100
C	0.11925000	2.33913300	-1.70755700
C	0.61975900	2.24909200	0.89072000
C	-0.36607800	3.05897700	1.31191900
C	2.03479100	2.30778300	1.36956800
C	-0.20019900	3.94308700	2.53344300
H	-1.34247300	3.10241200	0.81125800
C	2.27058300	3.57690100	2.21530800
H	2.25935700	1.40246800	1.97332800
H	2.73981700	2.28310300	0.51296900
C	1.14606000	3.75176900	3.23788600
H	-1.04993200	3.76445000	3.22785700
H	-0.32236900	4.99717600	2.19168300
H	3.25983900	3.50294600	2.71114900
H	2.30948500	4.46198200	1.54382800
H	1.34970600	4.61191900	3.90736100
H	1.10597900	2.84988500	3.88722900
C	2.70700400	1.84965900	-2.13680400
C	3.51042600	2.99906200	-1.91303600
C	3.29050900	0.70112500	-2.72745200
C	4.86564500	2.98554400	-2.26187000
H	3.05374400	3.89546400	-1.46807800
C	4.63853600	0.70823400	-3.09535200
H	2.66998600	-0.18894700	-2.89946900
C	5.43107700	1.84412600	-2.85530500
H	5.48384100	3.87782100	-2.08260200
H	5.07890300	-0.18389400	-3.56312100

H	6.49454900	1.84198500	-3.13731600
H	-0.61626800	3.04013900	-2.11279700

P6Z:

Eel = -5308.811475
Zero-point correction = 0.750343
Thermal correction to Energy = 0.797152
Thermal correction to Enthalpy = 0.798097
Thermal correction to Gibbs Free Energy = 0.667833

Fe	-3.06247700	-0.36323100	-1.57393000
P	-0.24461700	-1.72659500	-0.37211500
P	-1.09151900	1.57916400	0.36142500
C	-1.05399600	-2.94901300	0.72877400
C	1.15793500	-2.58769200	-1.21060600
C	-1.42187900	-2.53298600	2.02452700
H	-1.18719400	-1.50568300	2.34422900
C	-2.74637200	0.99358600	-0.12802100
C	-0.28683700	3.04699500	-1.91375900
H	0.22943100	2.12441100	-2.21972700
C	-0.88293900	5.36533600	-2.33448700
H	-0.85565500	6.25354800	-2.98316500
C	-1.44581900	-1.53616500	-1.73788800
C	2.94521700	-2.39264600	-2.86526900
H	3.45105300	-1.81397000	-3.65234000
C	-1.36362400	-4.26165400	0.30972900
H	-1.09385600	-4.59875200	-0.70216200
C	-1.33563600	2.20610200	2.07921400
C	3.43523900	-3.65455100	-2.49005400
H	4.32483200	-4.07083700	-2.98550400
C	-2.38001300	-4.71964800	2.47421700
H	-2.89447600	-5.41355200	3.15562900
C	-0.94850700	3.09717300	-0.67059300
C	-1.56993100	4.29315800	-0.25956900
H	-2.08223500	4.34257000	0.71295300
C	-3.45111200	-0.15999200	0.39740700
H	-3.09816400	-0.83149800	1.18739100
C	-0.25743300	4.17659400	-2.74429300
H	0.26314000	4.13097400	-3.71255200
C	1.65553000	-3.85204400	-0.83793100
H	1.17210700	-4.42817500	-0.03697200
C	2.78807400	-4.38046300	-1.47835500
H	3.16756400	-5.36899800	-1.17919700

C	-2.02077800	-5.14114200	1.18308900
H	-2.25636500	-6.16360100	0.85172400
C	-3.57044200	1.56613900	-1.17692200
H	-3.31267300	2.44031700	-1.78491700
C	-3.32274200	-1.70803700	-3.11312700
H	-4.28293200	-2.03800500	-3.52810500
C	1.81518300	-1.86147000	-2.22949300
H	1.45367400	-0.86190100	-2.51488100
C	-4.75939500	0.77308000	-1.28517300
H	-5.56828000	0.93135200	-2.00876500
C	-1.53417600	5.42241700	-1.09194800
H	-2.01775900	6.35482000	-0.76443800
C	-1.66875300	3.23506500	4.68202600
H	-1.79811900	3.63390800	5.69923200
C	-2.75691800	2.66292400	4.00685100
H	-3.74460500	2.61655600	4.48958900
C	-2.59714900	2.15278000	2.70826100
H	-3.46160400	1.72469000	2.18174900
C	-2.08384600	-3.41326800	2.89350400
H	-2.36666500	-3.07830900	3.90270200
C	-0.41298000	3.30029700	4.05432800
H	0.44274700	3.75511400	4.57539600
C	-1.39854900	-0.47073500	-2.71842200
H	-0.63178400	0.30868100	-2.78249000
C	-0.24369400	2.78305800	2.76367300
H	0.74272700	2.83117300	2.27593400
C	-2.64751100	-2.30366700	-1.99611100
H	-3.00659300	-3.15548400	-1.40845200
C	-4.68855100	-0.28423000	-0.31863700
H	-5.43301300	-1.07627700	-0.17241600
C	-2.55511100	-0.58185100	-3.55861100
H	-2.82161200	0.09933400	-4.37565300
Ni	0.58166800	0.20447600	0.43417200
C	1.67256700	1.70827800	0.30582000
H	1.55273600	2.72292600	-0.09984200
C	2.85634800	1.03838400	0.46155500
C	4.14651700	1.49105800	-0.11799500
C	4.19542800	2.02997400	-1.42222900
C	5.33689300	1.43887600	0.64303400
C	5.40317600	2.50074900	-1.95507300
H	3.27234500	2.06193700	-2.02135600
C	6.54276100	1.91270900	0.10951700
H	5.30779600	1.04802900	1.67209500
C	6.57992000	2.44152200	-1.19144900

H	5.42864300	2.91286600	-2.97508200
H	7.46046000	1.87497500	0.71547600
H	7.52874100	2.80904500	-1.60994800
C	2.72606200	-0.23524600	1.22267300
C	1.79161100	-0.29981500	2.26126400
C	3.61596800	-1.41412900	0.89342900
C	1.66571500	-1.50075700	3.15943400
H	1.38729800	0.64618700	2.65775200
C	3.65431000	-2.49115100	1.98594700
H	3.28299300	-1.84866100	-0.07032100
H	4.63529800	-1.03396100	0.68241700
C	2.26288700	-2.77260300	2.55973000
H	0.61150600	-1.65426500	3.46414500
H	2.19565700	-1.24501500	4.10735100
H	4.10415900	-3.41586700	1.57198300
H	4.32294500	-2.16019400	2.81075900
H	2.30549100	-3.57187100	3.32669200
H	1.59404000	-3.14616600	1.75686700

P8:

Eel = -5308.808362
Zero-point correction = 0.750202
Thermal correction to Energy = 0.796770
Thermal correction to Enthalpy = 0.797714
Thermal correction to Gibbs Free Energy = 0.671439

Fe	-1.55700900	-1.72250300	2.30688700
P	-1.83393500	-0.30766900	-0.67642900
P	1.25303700	-0.35477700	0.81317900
C	-1.64334000	-1.89606100	-1.59074800
C	-3.18140000	0.49021200	-1.64459900
C	-0.35529800	-2.38047500	-1.89157700
H	0.52791100	-1.81122500	-1.56490300
C	0.40660400	-1.62744100	1.83713400
C	0.93480900	1.97889500	2.32741200
H	0.09887600	2.17295900	1.63973500
C	2.27945200	2.60488800	4.25237000
H	2.49401500	3.29392100	5.08286100
C	-2.59917000	-0.72588200	0.91356800
C	-5.42945900	1.39882300	-1.85951300
H	-6.42043600	1.59730300	-1.42438500
C	-2.77320800	-2.61405900	-2.03630100
H	-3.78457300	-2.22094200	-1.85132700

C	2.80921500	-1.19360400	0.31736800
C	-5.14664400	1.79499400	-3.17599900
H	-5.91502400	2.30732900	-3.77395200
C	-1.31962600	-4.30594500	-3.01485400
H	-1.19651600	-5.24739500	-3.57076300
C	1.73915900	0.84178400	2.12482200
C	2.81222800	0.58269200	3.00313000
H	3.43287200	-0.31539900	2.87014400
C	-0.16886400	-2.89652000	1.42877400
H	-0.24514000	-3.28088900	0.40637100
C	1.20090800	2.85588900	3.38974900
H	0.56226500	3.73895300	3.54472800
C	-2.90345100	0.87947000	-2.97231300
H	-1.91803700	0.66287300	-3.41675200
C	-3.88362100	1.53027200	-3.73360400
H	-3.66296100	1.83030100	-4.76892300
C	-2.60805000	-3.81925900	-2.73451300
H	-3.49325600	-4.37816100	-3.07326100
C	0.22359600	-1.52116500	3.27331900
H	0.52272900	-0.66997700	3.89382500
C	-3.57523600	-1.86680300	2.69511600
H	-4.06413900	-2.64878500	3.28897100
C	-4.45074400	0.74727600	-1.09200900
H	-4.67292300	0.44230700	-0.05814800
C	-0.43803600	-2.70631500	3.73036400
H	-0.74217700	-2.90907900	4.76446900
C	3.08246800	1.46873000	4.05650400
H	3.92482300	1.26537800	4.73452300
C	5.18038800	-2.42409500	-0.58743000
H	6.10613200	-2.90343800	-0.93883300
C	3.99394000	-3.16712000	-0.49263000
H	3.98573600	-4.23405900	-0.76128800
C	2.81414400	-2.55858900	-0.04181200
H	1.90202700	-3.16223500	0.04525700
C	-0.19185900	-3.58106600	-2.59920300
H	0.82114500	-3.94529400	-2.82842700
C	5.18343000	-1.06827000	-0.22556100
H	6.10869400	-0.47835400	-0.29674600
C	-2.44931900	0.08175800	2.11171700
H	-1.93500600	1.04747600	2.17608600
C	4.00589300	-0.45128400	0.21600500
H	4.02056400	0.61229500	0.48970300
C	-3.30292700	-1.93436700	1.28862300
H	-3.53966700	-2.77264700	0.62347500

C	-0.67666600	-3.55315400	2.59944100
H	-1.19565300	-4.51929600	2.61304700
C	-3.05412100	-0.62883700	3.20067800
H	-3.07996300	-0.29823600	4.24598900
Ni	0.11050500	0.82232800	-0.69456400
C	1.65791900	1.73013600	-1.39620300
C	1.55592600	3.09733600	-1.23356600
C	0.34401000	3.59487200	-0.64744100
C	-0.81599200	2.82761200	-0.82443800
C	0.33181000	4.88202400	0.13518600
C	-2.12329900	3.18018900	-0.15578300
H	-0.87489600	2.29187400	-1.80164900
C	-1.07636900	5.40237100	0.43756300
H	0.89706100	4.71314200	1.08057600
H	0.93915500	5.62907000	-0.42183000
C	-1.97126100	4.25958700	0.92528100
H	-2.59696600	2.27741400	0.28142400
H	-2.84496700	3.51437500	-0.93441100
H	-1.02767700	6.21771900	1.18657500
H	-1.51730200	5.84442500	-0.48200800
H	-2.97308200	4.63430600	1.21571700
H	-1.53048300	3.81642500	1.84690800
C	2.78574600	1.10729900	-2.09446600
C	4.06543700	1.72073000	-2.14445900
C	2.61096100	-0.11564500	-2.78766700
C	5.11484900	1.13798800	-2.86230500
H	4.23681700	2.65943500	-1.59692800
C	3.66176500	-0.70324300	-3.49892400
H	1.61835000	-0.58956700	-2.77915600
C	4.92035300	-0.07948800	-3.53699100
H	6.09832300	1.63122700	-2.88956700
H	3.49996900	-1.65196600	-4.03207900
H	5.74902800	-0.54064500	-4.09482000
H	2.32362200	3.81080600	-1.58806100

P6Z-ETS:

Eel = -5308.774774
Zero-point correction = 0.747389
Thermal correction to Energy = 0.794402
Thermal correction to Enthalpy = 0.795346
Thermal correction to Gibbs Free Energy = 0.663916

Fe	-2.10675900	-2.46042500	-0.84327100

P	-1.83013500	0.88732100	-0.20884100
P	0.73526900	-1.38327800	0.59492500
C	-2.24835800	1.81478500	-1.74533000
C	-2.66119200	1.81267200	1.14545500
C	-1.78629600	1.31043400	-2.98112100
H	-1.22461800	0.36472500	-3.01095400
C	-0.12592500	-2.57819600	-0.46811800
C	-0.51034900	-0.81392900	3.04819200
H	-0.77333600	0.14690100	2.57716000
C	-0.57985800	-2.31275800	4.95990400
H	-0.89684800	-2.52905600	5.99115600
C	-2.84148100	-0.63028800	-0.35379000
C	-4.63828400	2.36115600	2.45778100
H	-5.70932300	2.22139100	2.66779800
C	-2.96007600	3.02917000	-1.71490400
H	-3.32436700	3.43122000	-0.75805700
C	2.49405800	-1.92451900	0.56514100
C	-3.88149400	3.25955000	3.22832900
H	-4.35951800	3.82230000	4.04412000
C	-2.75464400	3.21961000	-4.13507900
H	-2.95340000	3.76891900	-5.06733600
C	0.22785200	-1.76865200	2.31927800
C	0.58481400	-2.98829000	2.93147300
H	1.19792000	-3.72041600	2.38353900
C	-0.35857500	-2.33198700	-1.87762600
H	0.02050000	-1.46762800	-2.43865100
C	-0.91593900	-1.08710100	4.36378200
H	-1.49427400	-0.33659300	4.92346000
C	-1.90493800	2.71532300	1.92169300
H	-0.83349700	2.84346100	1.70324300
C	-2.51501100	3.43745200	2.95819400
H	-1.92062400	4.14075600	3.56091300
C	-3.20744600	3.72761700	-2.90835100
H	-3.76392700	4.67640400	-2.87630000
C	-0.77809200	-3.82344900	-0.12760600
H	-0.82456300	-4.27200100	0.87150900
C	-4.13646200	-2.38434600	-1.17536100
H	-4.70095600	-3.03218200	-1.85710000
C	-4.03372000	1.63762000	1.41924800
H	-4.62873200	0.93211500	0.82010000
C	-1.40231500	-4.33174500	-1.31605700
H	-2.00801000	-5.24415300	-1.37973200
C	0.17385400	-3.25937900	4.24468500
H	0.45005500	-4.21400100	4.71722800

C	5.22357700	-2.61143300	0.52066800
H	6.28961600	-2.88351900	0.50613700
C	4.33231800	-3.20955200	-0.38215500
H	4.69729700	-3.95000500	-1.10972400
C	2.96926800	-2.86902600	-0.36265600
H	2.27452200	-3.33854200	-1.07476900
C	-2.04637400	2.00636300	-4.16985700
H	-1.69071400	1.60144600	-5.12951300
C	4.75059300	-1.66778000	1.45076100
H	5.44531800	-1.20362300	2.16720800
C	-3.05505500	-1.58011100	0.71980400
H	-2.66525600	-1.49809500	1.74097400
C	3.39304000	-1.32218600	1.47116000
H	3.02542800	-0.58308600	2.20069400
C	-3.51715800	-1.13966400	-1.52678200
H	-3.54287100	-0.65901100	-2.51097900
C	-1.14455700	-3.41626500	-2.39161000
H	-1.51107400	-3.51361900	-3.42071700
C	-3.85798900	-2.65246800	0.20626500
H	-4.17112500	-3.54160800	0.76699600
Ni	0.33544000	0.72140600	0.02421300
C	1.00888600	2.38076800	-0.30373600
H	0.84712100	3.43795500	-0.57292800
C	2.14254600	1.51842800	-0.55555800
C	2.62668000	0.86483900	-1.77441100
C	1.85896400	0.91523400	-2.96445200
C	3.83640500	0.13401900	-1.76921000
C	2.28764500	0.23942100	-4.11280100
H	0.92980500	1.50490800	-2.98045100
C	4.26377600	-0.53162700	-2.92532000
H	4.42752900	0.06654700	-0.84412800
C	3.49087500	-0.48828800	-4.09716800
H	1.68690500	0.29167200	-5.03366200
H	5.20497000	-1.10085100	-2.90557500
H	3.82855900	-1.01618500	-5.00138200
C	2.87584900	2.29360000	0.43517200
C	2.15818900	2.54027300	1.62039800
C	4.07238400	3.12945900	0.05459700
C	2.43525700	3.71560200	2.51357000
H	1.47585200	1.75613500	1.98576900
C	4.06088400	4.50752900	0.73153900
H	4.97395100	2.55723700	0.37297400
H	4.14259200	3.20533200	-1.04914300
C	3.79234400	4.38251000	2.23449700

H	2.35378300	3.39956800	3.57462600
H	1.61056300	4.45357000	2.37399600
H	5.02480900	5.02236000	0.54816500
H	3.27552600	5.13917100	0.26061200
H	3.83164700	5.37523700	2.72509100
H	4.59837000	3.77350000	2.69827500

P6E:

Eel = -5308.797892
Zero-point correction = 0.749841
Thermal correction to Energy = 0.796712
Thermal correction to Enthalpy = 0.797657
Thermal correction to Gibbs Free Energy = 0.667018

Fe	2.80084600	-1.97936500	-0.93910100
P	-0.48499900	-1.41981300	-0.11932000
P	2.02540300	0.95222000	0.41476400
C	-1.17176400	-1.44584400	-1.82418200
C	-1.65602500	-2.43744100	0.87162100
C	-1.02000700	-0.32147500	-2.65821300
H	-0.54080700	0.58890500	-2.26780500
C	3.06411400	-0.00206400	-0.72344400
C	1.64435000	-0.08814000	2.99700200
H	0.61795000	-0.29390500	2.64554700
C	3.33065600	-0.14129000	4.74536900
H	3.63214000	-0.39900200	5.77168700
C	0.98070500	-2.50145100	-0.25544200
C	-2.09308900	-4.00812200	2.68366800
H	-1.71516700	-4.67684500	3.47131400
C	-1.80440700	-2.60570600	-2.32212800
H	-1.93062200	-3.48757100	-1.67663300
C	2.63845500	2.67909700	0.25001400
C	-3.47383200	-3.87989400	2.47061100
H	-4.18290900	-4.44430000	3.09436600
C	-2.11885300	-1.50951500	-4.47284000
H	-2.49234000	-1.53498200	-5.50752500
C	2.56766600	0.51560700	2.11791000
C	3.86709200	0.82007400	2.57411200
H	4.57653700	1.33870100	1.91110600
C	2.67023200	-0.31300200	-2.08500700
H	1.76252100	0.04115200	-2.58757600
C	2.02600000	-0.41730200	4.30687100
H	1.29937700	-0.88433600	4.98844300

C	-3.04796600	-2.30945700	0.66251800
H	-3.42755500	-1.64066900	-0.12325900
C	-3.94842500	-3.03196600	1.45592000
H	-5.02984400	-2.92803000	1.28049100
C	-2.27544500	-2.63249400	-3.64256700
H	-2.76980900	-3.53791400	-4.02532200
C	4.30443700	-0.69912600	-0.45651400
H	4.84603400	-0.70643800	0.49612700
C	2.53562800	-4.01595000	-1.10937700
H	3.07354100	-4.68175000	-1.79528700
C	-1.18451600	-3.29136900	1.88882900
H	-0.10539800	-3.41365900	2.05733600
C	4.65964100	-1.43041700	-1.63808100
H	5.52911000	-2.09087700	-1.74296000
C	4.24748500	0.48096200	3.88054200
H	5.26424300	0.71362900	4.23099200
C	3.38784400	5.37656900	-0.03188100
H	3.67956700	6.43143900	-0.14345900
C	3.84051700	4.42074600	-0.95479700
H	4.49217400	4.72383200	-1.78804700
C	3.46841700	3.07333200	-0.81805900
H	3.82340100	2.32685200	-1.54444500
C	-1.49125000	-0.35576300	-3.97954800
H	-1.37224200	0.53070200	-4.61987700
C	2.56759400	4.98393300	1.03990400
H	2.21936700	5.72924100	1.77051400
C	1.99484200	-2.73456000	0.75542200
H	2.05117600	-2.25785600	1.74074200
C	2.19348600	3.64100100	1.18270300
H	1.55851100	3.33648200	2.03055300
C	1.33126100	-3.30158300	-1.41409200
H	0.78251000	-3.32858900	-2.36183100
C	3.65862500	-1.19195900	-2.63843700
H	3.63236000	-1.63443500	-3.64159000
C	2.94317700	-3.66957700	0.22086700
H	3.84687500	-4.02366100	0.73163500
Ni	-0.20771200	0.68733900	0.39694000
C	-2.00329200	0.59303500	0.96210700
H	-2.32637300	-0.09925900	1.75873700
C	-2.90214100	1.41406000	0.33500200
C	-2.29200200	2.22107400	-0.73877200
C	-2.87831600	2.55206400	-1.97882900
C	-0.92308800	2.59128500	-0.52696700
C	-2.12572300	3.18781800	-2.97650300

H	-3.91748000	2.26860200	-2.18593300
C	-0.17104100	3.22624100	-1.53645500
H	-0.55029300	2.64524500	0.53160500
C	-0.77142300	3.51211600	-2.76887900
H	-2.60181100	3.42635700	-3.93982100
H	0.86687700	3.52675200	-1.33900900
H	-0.19662200	4.02079600	-3.55675100
C	-4.33015200	1.46708400	0.74207800
C	-4.65147800	1.65464100	2.04835300
C	-5.40526000	1.20377500	-0.30175500
C	-6.05252300	1.63678100	2.59117200
H	-3.83390100	1.82789500	2.76887300
C	-6.71371800	0.71974900	0.33565400
H	-5.60199200	2.13480800	-0.88094600
H	-5.03426800	0.46115400	-1.04124300
C	-7.12232800	1.63680900	1.49165100
H	-6.19749100	2.49612300	3.28121300
H	-6.16398800	0.73465000	3.23853000
H	-7.51231300	0.66922000	-0.43211400
H	-6.57242100	-0.31640700	0.71710400
H	-8.10369900	1.33509500	1.90977200
H	-7.25199700	2.67040900	1.10260400

P6-7ZTS:

Eel = -5897.298775
Zero-point correction = 0.793705
Thermal correction to Energy = 0.846785
Thermal correction to Enthalpy = 0.847729
Thermal correction to Gibbs Free Energy = 0.702722

Fe	-3.11852300	-1.38072300	-1.55761300
P	-0.44628100	-1.74947100	0.46712400
P	-1.62456500	1.37252600	-0.37616400
C	-0.45207400	-2.06228100	2.27899300
C	0.59020600	-3.06352700	-0.29483400
C	0.49690400	-1.41015500	3.09519100
H	1.23146400	-0.73519300	2.63218700
C	-2.51454900	0.51745300	-1.71346600
C	-1.70329500	4.23749600	-0.69074500
H	-2.39729800	4.28704500	0.16074500
C	-0.37026200	5.38180600	-2.37758400
H	-0.03411300	6.31612400	-2.85156200
C	-2.10314700	-2.27706900	-0.07168700

C	1.75398600	-3.91491600	-2.25703200
H	2.06761900	-3.78901300	-3.30457100
C	-1.39638800	-2.93086800	2.86684900
H	-2.14048900	-3.44346400	2.24006000
C	-2.83097000	1.68880700	0.96350300
C	2.11377300	-5.07040200	-1.54280400
H	2.70920600	-5.85698400	-2.03006000
C	-0.44284300	-2.48568300	5.06294200
H	-0.44281200	-2.64929300	6.15100400
C	-1.22338600	2.99606500	-1.15245300
C	-0.31247400	2.95619900	-2.23086400
H	0.08964300	1.99833500	-2.59554500
C	-3.93493800	0.42339900	-1.98017200
H	-4.73833600	0.88557800	-1.39680600
C	-1.28062600	5.42437100	-1.30955600
H	-1.66231100	6.39082100	-0.94733400
C	0.95642100	-4.21746800	0.42303300
H	0.64892500	-4.33733900	1.47254400
C	1.71802700	-5.21705600	-0.20411800
H	2.00209500	-6.11789800	0.36023900
C	-1.38604500	-3.14031000	4.25425600
H	-2.12284900	-3.82102600	4.70640600
C	-1.82826300	-0.29675200	-2.69743200
H	-0.74000100	-0.42212500	-2.77844900
C	-4.41421200	-2.40705800	-0.33775400
H	-5.48836600	-2.21424100	-0.22814900
C	0.99865700	-2.90865500	-1.63755200
H	0.76349500	-1.98782300	-2.19329800
C	-2.82032600	-0.89217400	-3.54280800
H	-2.62313900	-1.59049200	-4.36502900
C	0.11534300	4.14607800	-2.83437000
H	0.84561900	4.09934000	-3.65526100
C	-4.61189400	2.05781700	3.11453100
H	-5.31091400	2.20080800	3.95212900
C	-3.39621000	1.38630400	3.32021600
H	-3.13606700	0.99994000	4.31703300
C	-2.50767600	1.20538500	2.25017100
H	-1.55384800	0.67516500	2.40475700
C	0.50061700	-1.62342900	4.48078100
H	1.24449400	-1.11180400	5.11020200
C	-4.93424400	2.55424100	1.84011800
H	-5.88307500	3.08824500	1.68106600
C	-2.38762400	-3.23245800	-1.12664300
H	-1.64071900	-3.78937600	-1.70283500

C	-4.04882500	2.37522200	0.76670400
H	-4.30369700	2.78067700	-0.22356000
C	-3.37153700	-1.76480300	0.41064800
H	-3.50710900	-1.01701600	1.20025200
C	-4.11243100	-0.45124600	-3.10374000
H	-5.07678800	-0.75804900	-3.52690800
C	-3.81046300	-3.30410600	-1.28042700
H	-4.34226800	-3.91868900	-2.01684900
Ni	0.26353500	0.30109200	0.23430400
S	2.73526000	0.53864500	-2.43582900
O	1.59118200	0.05168300	-3.30387600
O	2.96618300	2.01949000	-2.28460600
C	4.27166600	-0.15735200	-3.15972600
H	5.10206900	0.10844100	-2.47992700
H	4.13678700	-1.25118800	-3.23512800
H	4.38879800	0.31022900	-4.15598600
C	2.02413500	-0.39163300	0.17482400
H	2.32629400	-1.34203000	-0.29852000
C	2.97438600	0.39264000	0.79054100
C	4.42600700	0.17659500	0.64809200
C	4.97253200	-1.12722100	0.59719600
C	5.27788200	1.28482200	0.42476600
C	6.33456400	-1.31687500	0.33094500
H	4.31885200	-1.99313300	0.78405700
C	6.63862100	1.09091200	0.15341800
H	4.85108700	2.29954900	0.41530600
C	7.17067100	-0.20943000	0.10629100
H	6.75102400	-2.33507700	0.30494200
H	7.28880100	1.95929900	-0.03080200
H	8.24032000	-0.35965100	-0.10328700
C	2.38787300	1.52461000	1.51518800
C	1.29417000	2.16695000	0.94529600
C	2.89413500	1.86899400	2.89339200
C	0.54459100	3.25721800	1.66857100
H	1.24993500	2.14500900	-0.17038300
C	2.32517700	3.17699400	3.44915700
H	2.63537900	1.01722900	3.56594900
H	4.00439000	1.87137700	2.87168700
C	0.82148700	3.26302600	3.17526600
H	-0.54389000	3.16678700	1.48629400
H	0.82045300	4.23609300	1.21513900
H	2.54109600	3.25135800	4.53355300
H	2.83564100	4.03830700	2.96621300
H	0.38476800	4.17234300	3.63412400

H	0.30933900	2.39462600	3.64781600

P6-7ETS:

Eel = -5897.291030
Zero-point correction = 0.793821
Thermal correction to Energy = 0.846905
Thermal correction to Enthalpy = 0.847849
Thermal correction to Gibbs Free Energy = 0.703812

Fe	2.89397900	-2.23890300	-0.44522400
P	2.01311700	0.91993000	0.13927300
P	-0.41282500	-1.64058600	0.11569000
C	2.82895500	1.12440400	1.77219500
C	2.34805600	2.52477100	-0.71457800
C	2.19488400	0.62386500	2.92683800
H	1.19906100	0.16226100	2.84312800
C	1.08981700	-2.65034600	0.35690900
C	-1.00372700	-1.91366000	-2.60959900
H	-0.47711900	-0.95907700	-2.73768100
C	-2.19494900	-3.78845500	-3.59260600
H	-2.60331900	-4.30104500	-4.47645800
C	3.06893200	-0.26315700	-0.74757000
C	3.25557300	3.80435900	-2.57913500
H	3.78888000	3.82931500	-3.54119400
C	4.07732400	1.77111500	1.88640500
H	4.56538600	2.18688800	0.99141500
C	-1.48724500	-2.01533500	1.56293400
C	2.81538900	4.99822900	-1.98554800
H	3.00488600	5.96318100	-2.47908700
C	4.06230800	1.37645800	4.28825400
H	4.54594200	1.47611300	5.27153100
C	-1.17540200	-2.47029800	-1.32809700
C	-1.84584200	-3.70414100	-1.18377800
H	-1.96750600	-4.15758200	-0.18890600
C	2.10477500	-2.52872500	1.38622300
H	2.13615600	-1.78769400	2.19152400
C	-1.51654900	-2.56861400	-3.73812000
H	-1.39121700	-2.10790800	-4.72885500
C	1.90220900	3.72813600	-0.12428200
H	1.39217000	3.71129400	0.85006200
C	2.13571700	4.95749400	-0.75571200
H	1.79338100	5.88914700	-0.28096900
C	4.69089900	1.89219600	3.14195400

H	5.66494300	2.39679500	3.22741500
C	1.50391600	-3.72183400	-0.53218100
H	0.96642200	-4.04958500	-1.42846500
C	4.72357000	-1.80938600	-1.28585200
H	5.62016700	-2.43875700	-1.22930000
C	3.02059000	2.56989600	-1.95247800
H	3.36300000	1.64221900	-2.43037900
C	2.73895200	-4.25425900	-0.03826900
H	3.31958500	-5.05777800	-0.50750900
C	-2.35474200	-4.35571000	-2.31656700
H	-2.88097700	-5.31505300	-2.20067500
C	-3.16196800	-2.45058700	3.79020300
H	-3.81532700	-2.62751100	4.65761100
C	-1.76994700	-2.40053800	3.95521000
H	-1.32537300	-2.53902800	4.95198600
C	-0.93416300	-2.18796900	2.84817500
H	0.15409500	-2.17591500	2.99240600
C	2.81254900	0.74580900	4.18084500
H	2.30995000	0.35590600	5.07889200
C	-3.71795600	-2.27947800	2.51120700
H	-4.80851500	-2.32045900	2.37030800
C	2.68656300	-0.92388400	-1.98074600
H	1.79020300	-0.70375000	-2.57306100
C	-2.88953100	-2.05275100	1.40443800
H	-3.33885300	-1.90702500	0.41311700
C	4.33853800	-0.81640900	-0.32464000
H	4.87976700	-0.55377900	0.59164700
C	3.10806400	-3.52500500	1.13917200
H	4.02012200	-3.67095800	1.73055300
C	3.71275500	-1.87123400	-2.30249400
H	3.70536100	-2.55106100	-3.16304000
Ni	-0.18960900	0.51313200	0.03911100
C	-1.97528100	0.49614400	-0.55300000
H	-2.27719200	0.12881700	-1.54970400
C	-2.89304000	1.00221700	0.33418200
C	-2.29516700	1.46204000	1.60745500
C	-1.04136200	2.14116300	1.54757700
C	-2.83811400	1.15834700	2.87439100
C	-0.36361100	2.49996900	2.73038200
H	-0.72448600	2.57276400	0.57258900
C	-2.14876800	1.50289600	4.04323500
H	-3.78488800	0.60353400	2.93429800
C	-0.91255800	2.17425700	3.97613500
H	0.58819600	3.04797500	2.68160900

H	-2.57650700	1.24188600	5.02294200
H	-0.38566400	2.45751800	4.89936500
C	-4.33465100	1.12801700	0.06135700
C	-4.96207100	0.24962000	-0.77619300
C	-5.09452300	2.28204200	0.69333900
C	-6.40933000	0.32912100	-1.15465000
H	-4.37107800	-0.56789900	-1.22420900
C	-6.40110400	2.59364300	-0.04556900
H	-5.31292200	2.04586000	1.75891800
H	-4.43775800	3.17715800	0.72872100
C	-7.20618600	1.31417600	-0.28983000
H	-6.86053500	-0.68678600	-1.12601000
H	-6.46452700	0.62494800	-2.23003400
H	-6.99540900	3.33061400	0.53130800
H	-6.16576100	3.07179600	-1.02186300
H	-8.17985200	1.54182700	-0.76798400
H	-7.44081900	0.83906900	0.68812800
S	-0.44951600	1.68574600	-3.21753000
O	0.79855400	1.03401900	-3.77128700
O	-1.79595000	1.24948200	-3.73266200
C	-0.31187500	3.47369500	-3.59350500
H	-1.20430200	3.96542500	-3.16745700
H	0.62724900	3.84447100	-3.14505000
H	-0.29642500	3.55171600	-4.69734700

P7Z:

Eel = -5897.315042
Zero-point correction = 0.795957
Thermal correction to Energy = 0.848901
Thermal correction to Enthalpy = 0.849845
Thermal correction to Gibbs Free Energy = 0.707369

Fe	-3.09598400	-2.19337600	0.14082700
P	0.33640100	-1.67690600	0.26991100
P	-1.93035000	0.98957700	-0.15590100
C	1.28911900	-1.96065200	1.81548100
C	1.10785900	-2.71888200	-1.03163700
C	2.04338600	-0.93168700	2.40906200
H	2.08066000	0.05566800	1.93317400
C	-3.19864400	-0.28009500	-0.46192000
C	-2.26695500	3.75289400	-0.64483900
H	-1.69318800	3.91517100	0.27689900
C	-3.42917100	4.66894400	-2.57240700

H	-3.76001900	5.53748400	-3.16118100
C	-1.19235600	-2.59096900	0.65631600
C	1.12961600	-3.42632800	-3.36214200
H	0.69654800	-3.38169200	-4.37287800
C	1.22411400	-3.22773100	2.43883700
H	0.61792900	-4.03209500	1.99600800
C	-2.14542400	1.41619500	1.61870700
C	2.25067000	-4.23419600	-3.10781000
H	2.69823200	-4.82754900	-3.91895100
C	2.68897900	-2.43085700	4.21156700
H	3.23670500	-2.61575300	5.14768700
C	-2.57954700	2.44573900	-1.07026300
C	-3.31950500	2.25885400	-2.25479000
H	-3.55765500	1.24712500	-2.60798600
C	-4.32529100	-0.59058200	0.39120100
H	-4.54949400	-0.12069600	1.35554900
C	-2.69309100	4.85802500	-1.39227800
H	-2.44665600	5.87419900	-1.04996200
C	2.23581600	-3.52318500	-0.77818100
H	2.67597600	-3.56100400	0.22891300
C	2.80293500	-4.27701100	-1.81843900
H	3.68498000	-4.90284400	-1.61607800
C	1.93135000	-3.45979500	3.62617200
H	1.88534700	-4.45088900	4.10167700
C	-3.25097800	-1.17160100	-1.60400800
H	-2.53580800	-1.16238700	-2.43433700
C	-3.05485800	-3.37927500	1.81257600
H	-3.85767700	-3.47822500	2.55329700
C	0.56058800	-2.66257300	-2.33204900
H	-0.29048600	-1.99942300	-2.55101100
C	-4.39715900	-2.01850100	-1.44213900
H	-4.69820000	-2.82405100	-2.12295700
C	-3.74316100	3.36934700	-2.99761500
H	-4.32217200	3.21452300	-3.92014800
C	-2.44033100	1.99603300	4.35934900
H	-2.55771800	2.22479300	5.42914900
C	-1.32325600	1.27198000	3.91325700
H	-0.55861000	0.93036400	4.62717000
C	-1.17558300	0.98925300	2.54744900
H	-0.28724700	0.44266300	2.19591300
C	2.73910800	-1.16692900	3.60499300
H	3.32489700	-0.35601600	4.06379300
C	-3.40460400	2.43739100	3.43716200
H	-4.27633200	3.01189900	3.78458300

C	-1.75132500	-3.70162000	-0.08925800
H	-1.36315500	-4.09741800	-1.03422300
C	-3.26117600	2.15230800	2.07097800
H	-4.01179700	2.51097900	1.35071800
C	-2.01289500	-2.39258000	1.83605700
H	-1.86131900	-1.62642900	2.60546200
C	-5.05423000	-1.66434500	-0.21678600
H	-5.93971700	-2.15485400	0.20567900
C	-2.89452500	-4.17993800	0.63218900
H	-3.55305600	-4.99731700	0.31375900
Ni	0.27188100	0.43181800	-0.51555100
S	0.10574000	1.01327600	-2.67891500
O	-1.02761300	0.19788100	-3.26163000
O	0.02527100	2.51476000	-2.71792900
C	1.53586800	0.59802900	-3.72810800
H	2.41243600	1.15590400	-3.35561400
H	1.69602900	-0.49460600	-3.70540500
H	1.23521800	0.93982200	-4.73648700
C	2.12384800	0.20086700	-0.77624100
H	2.50372300	-0.60742100	-1.42446900
C	3.03450700	1.06827900	-0.21869900
C	4.47913600	0.89313200	-0.50917700
C	5.06992300	-0.39205700	-0.50742900
C	5.28278300	2.01082100	-0.84077700
C	6.42509900	-0.55437700	-0.82218200
H	4.45679300	-1.26394500	-0.23175500
C	6.63530400	1.84432100	-1.16285300
H	4.82839900	3.01360600	-0.86915400
C	7.21119200	0.56214900	-1.15162400
H	6.87476000	-1.55851000	-0.80459400
H	7.24550500	2.72011500	-1.42994200
H	8.27544900	0.43419600	-1.39952600
C	2.56906800	2.14543300	0.67803600
C	1.34522600	2.73728900	0.48224700
C	3.43691000	2.52387800	1.86373300
C	0.73527600	3.71443100	1.44589200
H	0.85028000	2.62608300	-0.50738800
C	2.98216200	3.80130100	2.57270300
H	3.44308200	1.66902000	2.57993400
H	4.49103800	2.60584400	1.53122400
C	1.46820700	3.78366400	2.78899300
H	-0.32889500	3.44755700	1.61467000
H	0.70658200	4.71641800	0.95806600
H	3.52284200	3.91221300	3.53386700

H	3.25529900	4.68373500	1.95372400
H	1.13172100	4.67238900	3.35925300
H	1.19392300	2.89586900	3.40095400

P7E:

Eel = -5897.312972
Zero-point correction = 0.795384
Thermal correction to Energy = 0.848495
Thermal correction to Enthalpy = 0.849439
Thermal correction to Gibbs Free Energy = 0.707811

Fe	-2.95625600	2.25732000	0.40969900
P	-2.02843900	-0.91188500	-0.17471000
P	0.42209500	1.60214800	0.26978600
C	-2.15339000	-1.35835000	1.60490000
C	-2.78651200	-2.36942900	-1.00128800
C	-1.03843200	-1.17264400	2.44374900
H	-0.08420500	-0.82084900	2.02095700
C	-1.01842200	2.48231000	0.97572400
C	0.95304100	2.66190800	-2.30184000
H	0.55466000	1.72687100	-2.71783900
C	1.92979700	4.85796400	-2.68406900
H	2.27155500	5.64154600	-3.37690600
C	-3.22437400	0.43932000	-0.41513300
C	-4.14161400	-3.35263700	-2.76497300
H	-4.81773700	-3.22913900	-3.62410000
C	-3.34685300	-1.88507700	2.14517700
H	-4.20575300	-2.08886800	1.48786300
C	1.67545800	1.58757800	1.62429700
C	-3.77354400	-4.63914500	-2.33859300
H	-4.16019900	-5.52530300	-2.86373500
C	-2.32847700	-1.93952900	4.35544300
H	-2.40128500	-2.16056500	5.43089000
C	1.06568900	2.85226800	-0.91248800
C	1.61042400	4.05573000	-0.40932200
H	1.69557900	4.21422700	0.67623500
C	-1.85758600	2.11382700	2.10111000
H	-1.77813400	1.20324000	2.70427200
C	1.38656800	3.66558600	-3.18346500
H	1.30125500	3.50075300	-4.26768600
C	-2.42524900	-3.66295700	-0.56693800
H	-1.76463500	-3.78227100	0.30437800
C	-2.91752300	-4.79337000	-1.23523100

H	-2.63612000	-5.79954300	-0.88965600
C	-3.43309300	-2.16698100	3.51573500
H	-4.36775900	-2.57176700	3.93201000
C	-1.54614200	3.72278200	0.43572500
H	-1.15354800	4.25419900	-0.43768300
C	-4.93512100	1.98873800	-0.10058100
H	-5.78679200	2.52693800	0.33326100
C	-3.64962100	-2.21722700	-2.10389800
H	-3.92156400	-1.21293200	-2.45576900
C	-2.66533100	4.11761700	1.23926700
H	-3.29030900	5.00318200	1.07122200
C	2.04165000	5.05100700	-1.29566700
H	2.46824200	5.98465300	-0.89939800
C	3.66303500	1.49465200	3.62606700
H	4.43717000	1.46521200	4.40728200
C	2.30657200	1.40265600	3.97308700
H	2.01084800	1.30542900	5.02845400
C	1.31558900	1.44981600	2.98006800
H	0.26063300	1.39589100	3.27598300
C	-1.12839900	-1.45252100	3.81624700
H	-0.24814200	-1.30174900	4.45855700
C	4.02679300	1.63652000	2.27625000
H	5.08670300	1.71835200	1.99234500
C	-3.11985200	1.44887600	-1.45153400
H	-2.37036200	1.44922600	-2.25072000
C	3.04176800	1.67754100	1.28030000
H	3.33902800	1.79702100	0.23012200
C	-4.35693600	0.78435900	0.41935500
H	-4.68375000	0.25301100	1.31983700
C	-2.85634800	3.13211100	2.26205000
H	-3.65321500	3.12875500	3.01563000
C	-4.17732900	2.39294000	-1.24928600
H	-4.35106300	3.29472900	-1.84898800
Ni	0.13451600	-0.41629200	-0.75098500
C	1.97056700	-0.37905500	-1.13182400
H	2.33186900	0.19248600	-2.00440000
C	2.84666200	-1.10489700	-0.35194300
C	2.28336600	-1.96699300	0.72828600
C	1.32747800	-2.96449900	0.41465600
C	2.70147000	-1.83047000	2.07215400
C	0.83100000	-3.81927300	1.41270200
H	1.03451000	-3.11268800	-0.63573400
C	2.18754400	-2.67199800	3.06633800
H	3.42406000	-1.04437400	2.33421200

C	1.26005500	-3.67711600	2.73883000
H	0.12166100	-4.61722200	1.14756700
H	2.51881800	-2.54655400	4.10840600
H	0.87298800	-4.34855800	3.51933100
C	4.30326300	-1.11190000	-0.57234000
C	4.92625200	-0.09174500	-1.24716100
C	5.10947200	-2.28762600	-0.04995500
C	6.39247600	-0.03718300	-1.53553400
H	4.31663700	0.74701900	-1.62389500
C	6.47121900	-2.42376700	-0.74026400
H	5.25432400	-2.17275600	1.04756400
H	4.51368000	-3.21807100	-0.15925500
C	7.20610000	-1.08140700	-0.76211000
H	6.76963400	0.99264700	-1.34936200
H	6.52580200	-0.17796800	-2.63610400
H	7.07606900	-3.19795300	-0.22701600
H	6.32219800	-2.77990300	-1.78332000
H	8.21591400	-1.18514000	-1.20654300
H	7.35741300	-0.73044600	0.28243600
S	-0.16488000	-0.83371900	-2.97847000
O	-1.57206300	-0.37482300	-3.26924000
O	0.91543900	-0.24973300	-3.84253100
C	-0.11938500	-2.62493000	-3.30551500
H	0.91088300	-2.96932600	-3.10419100
H	-0.86727200	-3.12983700	-2.66830800
H	-0.37051800	-2.72399300	-4.37866500

P7-4ZTS:

Eel = -5897.307801
Zero-point correction = 0.796500
Thermal correction to Energy = 0.848421
Thermal correction to Enthalpy = 0.849365
Thermal correction to Gibbs Free Energy = 0.709860

Fe	3.46505900	-1.56417100	-0.58234200
P	1.68612800	1.25838300	-0.18917700
P	0.18895800	-1.82905500	0.37600800
C	2.39787200	1.68077000	1.45155200
C	1.59323400	2.86762900	-1.08097600
C	1.90696500	1.02784600	2.60098700
H	1.08556500	0.29996300	2.50629300
C	1.91688500	-2.40555200	0.42274300
C	-0.55187400	-2.79642600	-2.14717600

H	-0.08290500	-1.88173800	-2.53889600
C	-1.76237800	-4.86748200	-2.54508600
H	-2.22535000	-5.58077600	-3.24327200
C	3.02059200	0.34836900	-1.02873800
C	1.65961500	4.13466400	-3.16019700
H	1.87624600	4.17844500	-4.23811900
C	3.42911300	2.63460300	1.58343700
H	3.80369100	3.16575300	0.69506100
C	-0.47757300	-2.22011800	2.04832200
C	1.18609800	5.27455900	-2.49034900
H	1.02752900	6.21325400	-3.04192000
C	3.48478100	2.25016200	3.98687200
H	3.90986100	2.47480700	4.97653600
C	-0.59978100	-3.03822700	-0.75804400
C	-1.23084400	-4.19790400	-0.26522800
H	-1.27393300	-4.38815700	0.81725700
C	2.97086000	-1.98868800	1.32996300
H	2.88066500	-1.25674700	2.13979700
C	-1.13440100	-3.71130300	-3.03564000
H	-1.10983900	-3.50627600	-4.11599200
C	1.13095400	4.01924300	-0.40793400
H	0.94232000	3.98522800	0.67414700
C	0.92782300	5.21585000	-1.11071600
H	0.57360500	6.10935000	-0.57460500
C	3.96915900	2.91478600	2.84762800
H	4.77301400	3.65988000	2.94446900
C	2.49484300	-3.35899700	-0.50384400
H	1.96094400	-3.84912600	-1.32573400
C	5.06608700	-0.65565700	-1.50959900
H	6.11334700	-0.97383300	-1.43725100
C	1.86034400	2.93339000	-2.46406800
H	2.21207200	2.04276500	-3.00175100
C	3.87309200	-3.53695700	-0.15428200
H	4.58782000	-4.17856700	-0.68390700
C	-1.80866100	-5.10879400	-1.16245900
H	-2.30253200	-6.01278700	-0.77572000
C	-1.58227400	-2.80289100	4.57802000
H	-2.01110300	-3.02864500	5.56578900
C	-0.26043600	-3.17096900	4.28380300
H	0.34789500	-3.69430800	5.03669600
C	0.29262000	-2.88699400	3.02515900
H	1.32089100	-3.20178600	2.80045600
C	2.45149400	1.30754100	3.86331300
H	2.06095200	0.79201300	4.75354300

C	-2.35829200	-2.15351700	3.60363600
H	-3.40023900	-1.87312000	3.81928900
C	2.84889300	-0.49511100	-2.19663300
H	1.90870100	-0.62249700	-2.74420600
C	-1.81092000	-1.85836500	2.34831500
H	-2.42795300	-1.35331800	1.59144500
C	4.40253300	0.24186400	-0.61006800
H	4.84736500	0.72427000	0.26777200
C	4.16569400	-2.70017500	0.97292600
H	5.14300400	-2.58897400	1.45821500
C	4.11379900	-1.10413900	-2.48416200
H	4.30858800	-1.82177900	-3.29042500
Ni	-0.32483700	0.26108400	-0.13812600
C	-2.13162300	-0.19042000	-0.55417800
H	-2.44286700	-1.13701100	-1.02798500
C	-3.11813400	0.62428200	0.01403200
C	-4.53935600	0.24552800	-0.05880000
C	-5.40391600	0.46863000	1.04442900
C	-5.05660100	-0.39157900	-1.21560400
C	-6.74288000	0.06807400	0.99046400
H	-5.00437600	0.93411100	1.95922000
C	-6.40011500	-0.78108800	-1.26553100
H	-4.39744300	-0.53809900	-2.08521500
C	-7.24567100	-0.55590200	-0.16576800
H	-7.40102900	0.23577600	1.85618700
H	-6.79465500	-1.26004800	-2.17412700
H	-8.30023200	-0.86709600	-0.20777000
C	-2.67400400	1.81121600	0.75169200
C	-1.53800700	1.73622100	1.53670600
C	-3.45239300	3.10878600	0.65174100
C	-1.01736700	2.88297400	2.35768300
H	-1.16902900	0.72411600	1.80575000
C	-3.17937000	4.06438100	1.81603600
H	-3.17117400	3.60544600	-0.30561000
H	-4.53262400	2.88379900	0.55011300
C	-1.67134000	4.22864800	2.02172000
H	0.08852500	2.93812400	2.27811700
H	-1.19191900	2.62906900	3.42932700
H	-3.66068200	5.04371100	1.62247600
H	-3.64235000	3.66153000	2.74373700
H	-1.45292100	4.96312000	2.82264200
H	-1.22790200	4.63787700	1.08792800
S	-1.34690400	0.60107500	-2.51430200
O	0.05909900	0.46098600	-3.04123100

O	-2.40572100	-0.08480000	-3.31142300
C	-1.73726900	2.37475900	-2.51141000
H	-2.79982700	2.48343500	-2.23470300
H	-1.05924700	2.89847500	-1.81294100
H	-1.55663600	2.69676100	-3.55422700

P7-4ETS:

Eel = -5897.305632

Zero-point correction = 0.795611

Thermal correction to Energy = 0.847833

Thermal correction to Enthalpy = 0.848778

Thermal correction to Gibbs Free Energy = 0.707771

Fe	3.43787700	-1.64633000	-0.64814200
P	1.77545700	1.22236000	-0.12200700
P	0.15183800	-1.82583000	0.30549300
C	2.48538800	1.55077800	1.54086800
C	1.74998300	2.87454400	-0.93584400
C	1.95989600	0.87385900	2.66053400
H	1.12299600	0.16951800	2.52972900
C	1.85630800	-2.47043800	0.31358300
C	-0.66194300	-2.61436000	-2.25811800
H	-0.19267400	-1.67879300	-2.59814900
C	-1.90121300	-4.64494300	-2.76426100
H	-2.38432500	-5.30783000	-3.49766700
C	3.07348000	0.30254100	-1.00179800
C	1.92794600	4.24480100	-2.94114000
H	2.18838800	4.33948400	-4.00600500
C	3.54339400	2.46685400	1.71892300
H	3.94502300	3.01685100	0.85414900
C	-0.51313300	-2.29199100	1.95971200
C	1.45262100	5.35924500	-2.23068500
H	1.33652100	6.32857600	-2.73818800
C	3.55269200	2.00171600	4.10876000
H	3.96981300	2.18023100	5.11113500
C	-0.68285300	-2.94657200	-0.88711700
C	-1.31641500	-4.12955300	-0.45754500
H	-1.33868800	-4.38917700	0.61124800
C	2.92260400	-2.14127200	1.24189300
H	2.85734600	-1.44956000	2.08882100
C	-1.27144500	-3.46478300	-3.19179700
H	-1.26501500	-3.19154300	-4.25731300
C	1.28114200	3.99886900	-0.22206600

H	1.02595200	3.90783600	0.84244800
C	1.13353400	5.23435300	-0.86829700
H	0.76785900	6.10442600	-0.30243600
C	4.07427800	2.68727300	2.99872100
H	4.89963900	3.40284300	3.13111900
C	2.39685300	-3.40186600	-0.65649100
H	1.84575500	-3.82871300	-1.50207200
C	5.07136500	-0.76396100	-1.54129300
H	6.10451200	-1.12910600	-1.49110700
C	2.07379700	3.00467300	-2.30214100
H	2.42909700	2.13497800	-2.87121900
C	3.76605100	-3.65167400	-0.31480200
H	4.45629900	-4.29505000	-0.87396700
C	-1.92275600	-4.97475300	-1.39936800
H	-2.41837200	-5.89740200	-1.06188500
C	-1.61008900	-2.99251100	4.46258500
H	-2.03519300	-3.26326600	5.44058100
C	-0.33871500	-3.45900300	4.09422300
H	0.23262700	-4.10381100	4.77877500
C	0.21062300	-3.11540100	2.84903600
H	1.19993200	-3.50076600	2.56584000
C	2.49336900	1.09554600	3.93944400
H	2.07534400	0.56202300	4.80628500
C	-2.33927400	-2.18323900	3.57592600
H	-3.34005300	-1.81898000	3.85352300
C	2.85961800	-0.47763300	-2.20584900
H	1.91163600	-0.53930900	-2.75195300
C	-1.79530400	-1.83104800	2.33376700
H	-2.37267900	-1.19981400	1.64353300
C	4.45076800	0.11825900	-0.59660800
H	4.91890700	0.54203900	0.29935100
C	4.08881300	-2.88238800	0.85193200
H	5.06847900	-2.83482500	1.34288900
C	4.09624800	-1.12611100	-2.52957000
H	4.25699900	-1.81295900	-3.36944400
Ni	-0.26322300	0.29071100	-0.12186700
C	-2.09687600	-0.02381700	-0.54151900
H	-2.44800200	-0.92783000	-1.06825600
C	-3.06726000	0.76407300	0.10075100
C	-2.59337300	1.92446600	0.87277700
C	-3.26090900	3.17407200	0.83354700
C	-1.39099500	1.82771600	1.62441200
C	-2.73480100	4.28572100	1.49936200
H	-4.18440800	3.26995800	0.24148400

C	-0.87246100	2.94509700	2.30412400
H	-0.96072200	0.82341900	1.81618900
C	-1.53985600	4.17500900	2.23826400
H	-3.25885900	5.25179700	1.44640800
H	0.04020100	2.83666900	2.90896600
H	-1.14312100	5.04833900	2.77722100
C	-4.48809500	0.41088100	0.03589700
C	-4.99865900	-0.14459800	-1.11201800
C	-5.36867900	0.62709100	1.25460500
C	-6.42144800	-0.57551000	-1.26979600
H	-4.33818100	-0.22710600	-1.99254500
C	-6.86363100	0.59477400	0.91949600
H	-5.12935000	-0.17421700	1.99298300
H	-5.09762000	1.57293600	1.76516200
C	-7.20044800	-0.62046000	0.05007400
H	-6.44463000	-1.55990100	-1.78865900
H	-6.91275700	0.11795800	-1.99425800
H	-7.46041600	0.58974400	1.85364500
H	-7.13804600	1.52556900	0.37516300
H	-8.28912500	-0.67730000	-0.14932700
H	-6.93408600	-1.54764500	0.60332600
S	-1.31427800	0.89354100	-2.41499600
O	0.07246100	0.67474000	-2.96459000
O	-2.43087700	0.35497500	-3.24604800
C	-1.56492300	2.68348100	-2.27440000
H	-2.60031500	2.85787900	-1.93636100
H	-0.82176300	3.10652900	-1.57462700
H	-1.40441000	3.06082700	-3.30197300

P9Z:

Eel = -5663.524745
Zero-point correction = 0.660415
Thermal correction to Energy = 0.707247
Thermal correction to Enthalpy = 0.708191
Thermal correction to Gibbs Free Energy = 0.576847

Fe	1.45221200	-2.88123100	-0.68084600
P	1.94344800	0.37936400	-0.23635900
P	-1.21973200	-1.07881300	0.28048700
C	3.40285700	0.40194200	0.89574800
C	2.30075000	1.79410900	-1.36678400
C	3.19135600	0.33037800	2.28643400
H	2.16190600	0.32345000	2.67638300

C	-0.22573400	-2.61015600	0.42763300
C	-2.15064800	-0.64482400	-2.33925200
H	-1.42871600	0.18508800	-2.34224700
C	-3.90572800	-1.91495100	-3.44090100
H	-4.54101700	-2.10104600	-4.32047700
C	2.26141600	-1.10913000	-1.24823800
C	2.62110500	2.79248400	-3.56713800
H	2.76540700	2.67379100	-4.65219200
C	4.71758500	0.50045900	0.39317300
H	4.88216400	0.58611400	-0.69245000
C	-2.43679900	-1.23581200	1.66054200
C	2.56998500	4.07532600	-3.00086600
H	2.66727700	4.96572000	-3.64035600
C	5.59617500	0.40494800	2.65885800
H	6.45438100	0.40643900	3.34831400
C	-2.29174500	-1.43328000	-1.18245200
C	-3.26036100	-2.45935300	-1.15815900
H	-3.39576500	-3.06195300	-0.24665100
C	0.94696400	-2.69357900	1.27385000
H	1.30855200	-1.89436600	1.93309800
C	-2.95436800	-0.88418100	-3.46495800
H	-2.84424300	-0.24512400	-4.35378400
C	2.24957300	3.08873000	-0.80005600
H	2.07674300	3.20241000	0.28232100
C	2.39148200	4.21996400	-1.61303000
H	2.34745500	5.22314500	-1.16263600
C	5.80954600	0.49740500	1.27222900
H	6.83353300	0.57403600	0.87517500
C	-0.30399700	-3.84607600	-0.32158500
H	-1.05739900	-4.08215400	-1.08183000
C	3.00272200	-3.19740800	-1.98466600
H	3.58413700	-4.12298200	-2.07933900
C	2.49224700	1.65271900	-2.75502600
H	2.53526400	0.64913800	-3.20388100
C	0.80274500	-4.67574500	0.06763800
H	1.03863200	-5.66517800	-0.34390700
C	-4.05864700	-2.70135700	-2.28551700
H	-4.81159700	-3.50421900	-2.26042700
C	-4.26783800	-1.24622200	3.81303200
H	-4.98130900	-1.25221300	4.65088100
C	-3.07568500	-1.98398200	3.89476800
H	-2.85322300	-2.57388900	4.79728600
C	-2.16474400	-1.98231500	2.82582000
H	-1.23783000	-2.57086700	2.89486800

C	4.28917200	0.32848200	3.16311500
H	4.11887000	0.27928100	4.24950600
C	-4.54462900	-0.50080800	2.65319400
H	-5.47731800	0.07948600	2.57938600
C	1.33540800	-1.59906700	-2.24774600
H	0.41913800	-1.09241800	-2.57038400
C	-3.63510100	-0.48942300	1.58734600
H	-3.85308000	0.09792900	0.68261700
C	3.29349700	-2.11136100	-1.09015200
H	4.12676500	-2.05842900	-0.38026200
C	1.57227300	-3.96689400	1.05251800
H	2.49750600	-4.31781300	1.52584300
C	1.79874400	-2.87983300	-2.70088500
H	1.29676800	-3.51709100	-3.43917300
Ni	-0.10205200	0.78561100	0.47616300
C	-1.47045100	2.15315600	0.33363700
C	-1.75524400	2.89687200	1.43645400
H	-2.39835800	3.78992000	1.53295100
S	-0.78183700	2.56327200	2.87556000
O	-0.32869300	3.82090100	3.51442100
O	0.27108800	1.54607800	2.45167500
C	-1.81497400	1.68826800	4.07079600
H	-1.16036000	1.49075800	4.94035100
H	-2.65065500	2.35250500	4.35770600
H	-2.17897300	0.74928200	3.61812500
C	-2.12429000	2.44476000	-0.95259500
C	-3.53138500	2.41161900	-1.10454600
C	-1.33491000	2.72002600	-2.09847200
C	-4.12548500	2.61459800	-2.35889200
H	-4.15793900	2.21032400	-0.22185000
C	-1.93335200	2.95270400	-3.34259900
H	-0.24027300	2.74431400	-1.99363300
C	-3.33168100	2.88724400	-3.48426400
H	-5.22108900	2.56406500	-2.45698800
H	-1.29811000	3.17745400	-4.21364200
H	-3.79963300	3.05093500	-4.46697900

P9-3ZTSB:

Eel = -6858.918266
Zero-point correction = 0.819692
Thermal correction to Energy = 0.881924
Thermal correction to Enthalpy = 0.882868
Thermal correction to Gibbs Free Energy = 0.716097

Fe	-1.81176600	-1.68180100	1.91035200
P	1.45558400	-1.52468300	0.60607700
P	-3.39131700	0.30326700	-0.35843700
C	2.24522100	-1.35797900	2.26676900
C	2.43008900	-2.88714700	-0.15842600
C	1.83673600	-0.28095800	3.07622800
H	1.11041200	0.44833000	2.69995300
C	-2.94666900	-0.07305500	1.37772700
C	-4.87634200	-1.95072300	-1.10015500
H	-3.90757900	-2.31002600	-1.48056000
C	-7.25886700	-2.29018100	-0.74981200
H	-8.15696600	-2.91972100	-0.84302500
C	-0.10076600	-2.37000200	1.04901500
C	2.68588600	-5.17555500	-0.95618200
H	2.26205300	-6.17912400	-1.11398600
C	3.20144100	-2.26824600	2.76112400
H	3.52812400	-3.11646800	2.14243800
C	-4.02216000	2.03737300	-0.23871100
C	4.00629700	-4.89986700	-1.34306700
H	4.62354400	-5.68640400	-1.80324700
C	3.32616900	-1.01661400	4.84881700
H	3.75519300	-0.88150000	5.85343900
C	-4.95851500	-0.67126900	-0.51062000
C	-6.20918900	-0.20541800	-0.05503700
H	-6.28389500	0.79476800	0.39869600
C	-1.72078700	0.34861000	2.02122000
H	-0.94826600	0.98712900	1.57820200
C	-6.01853800	-2.75900100	-1.21164700
H	-5.94066400	-3.75636800	-1.67147600
C	3.75423700	-2.60704900	-0.55858900
H	4.15515500	-1.59083900	-0.41478700
C	4.53934700	-3.61579300	-1.13555500
H	5.58052100	-3.40258600	-1.42534300
C	3.73644200	-2.09454100	4.04748200
H	4.48297200	-2.81005100	4.42521400
C	-3.65747900	-0.91617900	2.31200100
H	-4.61047200	-1.41922600	2.11064800
C	-1.64052200	-3.69706700	2.19329400
H	-2.13680200	-4.27379500	2.98376700
C	1.90023900	-4.17641800	-0.35927700
H	0.86787000	-4.39793100	-0.05302000
C	-2.87986100	-1.00606100	3.51591100
H	-3.13678300	-1.59609000	4.40462200

C	-7.35188500	-1.01091100	-0.17659400
H	-8.32352100	-0.63754000	0.18222100
C	-4.79710600	4.75490600	-0.28216300
H	-5.09308700	5.81474400	-0.29932000
C	-4.31605100	4.17586600	0.90180200
H	-4.23560300	4.78024900	1.81834300
C	-3.93440000	2.82409700	0.92735600
H	-3.55930700	2.37820600	1.86058600
C	2.37499800	-0.10782500	4.35833200
H	2.05519900	0.75623100	4.96039100
C	-4.89394500	3.97594200	-1.44832300
H	-5.26929300	4.42299900	-2.38179600
C	-1.23865200	-2.58856400	0.18272100
H	-1.35559800	-2.17779600	-0.82703000
C	-4.49928400	2.63149100	-1.42911600
H	-4.55865200	2.02984300	-2.35121500
C	-0.36033500	-3.05769100	2.29709300
H	0.30779500	-3.06463800	3.16601200
C	-1.69046000	-0.21982800	3.34083000
H	-0.88314200	-0.09525900	4.07171300
C	-2.18059300	-3.41106500	0.89129200
H	-3.16240400	-3.72713900	0.51851700
Ni	1.33407800	0.38016500	-0.62583000
C	1.43496900	-0.43699100	-2.32519500
C	2.55801300	-0.34167800	-3.08588800
H	2.66953900	-0.75028900	-4.10667700
S	3.95396300	0.60861200	-2.55326900
O	3.95090700	0.68347900	-1.05248900
O	4.07653600	1.87387400	-3.32883600
C	5.37736400	-0.39710000	-3.04110500
H	6.26061100	0.15788000	-2.67393500
H	5.30832600	-1.39775500	-2.58015500
H	5.39332500	-0.45872300	-4.14463500
C	0.79687600	2.22157500	-1.09402300
C	-0.65560400	2.48499700	-1.35284600
C	1.80460100	3.01276000	-1.59852900
C	-0.81960000	3.03436200	-2.78219500
H	-1.01556400	3.24027600	-0.62157800
H	-1.27113900	1.57571300	-1.19904600
C	1.57406500	3.86252500	-2.81652300
H	2.83037000	2.92197600	-1.21889300
C	0.09880700	4.24010900	-3.00426900
H	-1.88474200	3.30462300	-2.93585700
H	-0.57589600	2.23858300	-3.52001200

H	2.21617000	4.76705700	-2.74613200
H	1.96708200	3.30480800	-3.69833800
H	-0.05654100	4.66623700	-4.01690300
H	-0.17146400	5.03520500	-2.27633500
O	0.93569200	1.92692500	0.64963300
S	1.29452000	3.12513600	1.66494600
O	0.97521700	4.42984700	1.07000700
O	0.77782300	2.74571300	2.99332700
C	3.19682000	3.04431500	1.81354100
F	3.76279200	3.72763900	0.81429900
F	3.52776000	3.61124700	2.97957000
F	3.62143800	1.78170500	1.79368200
C	0.33363500	-1.28994400	-2.82250800
C	0.58067100	-2.65289800	-3.12612900
C	-0.98035800	-0.79699400	-2.99291400
C	-0.45333400	-3.48930200	-3.56938100
H	1.59505300	-3.05322100	-2.98622600
C	-2.00444500	-1.62721400	-3.46739600
H	-1.20054000	0.24706900	-2.74285200
C	-1.75050800	-2.98061400	-3.74813000
H	-0.24090300	-4.54892700	-3.78006300
H	-3.01507600	-1.21261700	-3.60087600
H	-2.56074400	-3.63555900	-4.10333200

P9-3ZTS:

Eel = -6858.924027
Zero-point correction = 0.821261
Thermal correction to Energy = 0.882426
Thermal correction to Enthalpy = 0.883370
Thermal correction to Gibbs Free Energy = 0.725261

Fe	3.50416800	-0.81204000	-2.03658700
P	1.91701000	1.44688200	-0.05250800
P	0.78641800	-1.81178900	-0.12557400
C	3.16123500	1.18085900	1.29792100
C	1.92713300	3.29299800	-0.10888300
C	2.99315300	0.12235800	2.20854200
H	2.10629600	-0.51685300	2.13123700
C	2.41385800	-2.11809200	-0.95488600
C	-0.58374600	-2.25153200	-2.52377500
H	-0.23321900	-1.23856200	-2.76768900
C	-1.79264100	-4.26382300	-3.14743200
H	-2.39330900	-4.82872700	-3.87607800

C	2.87661900	1.03934000	-1.55553100
C	2.29194900	5.40738900	-1.26927800
H	2.58320800	5.95229900	-2.18009400
C	4.27580000	2.03361300	1.43984800
H	4.39635200	2.89553200	0.76634600
C	1.06213500	-2.92028000	1.33680800
C	1.91343100	6.11148600	-0.11639500
H	1.90241500	7.21187800	-0.11989300
C	5.06009100	0.72427600	3.33719600
H	5.80237200	0.54609600	4.13017100
C	-0.27378200	-2.81242500	-1.26619800
C	-0.73856600	-4.10288100	-0.95672400
H	-0.53642900	-4.54294600	0.02894300
C	3.71883700	-1.92297400	-0.35489000
H	3.91285700	-1.53480900	0.65064300
C	-1.33305900	-2.97465800	-3.46108100
H	-1.57061500	-2.52171400	-4.43538100
C	1.55600900	4.00538500	1.05322800
H	1.26627300	3.44893900	1.95747700
C	1.54803400	5.40640900	1.04366800
H	1.24834900	5.95328200	1.95049000
C	5.22249500	1.80055000	2.44946800
H	6.09003000	2.47101000	2.54738000
C	2.63146300	-2.63499100	-2.28985900
H	1.84664600	-2.88450100	-3.01181300
C	4.60835500	0.58709500	-3.05845900
H	5.61081700	0.43795600	-3.47847200
C	2.29792100	4.00291700	-1.26865600
H	2.60111900	3.45674200	-2.17397900
C	4.04539500	-2.73611000	-2.50782300
H	4.52714100	-3.06250500	-3.43781300
C	-1.49767500	-4.81966100	-1.89414000
H	-1.87435100	-5.81936900	-1.63075200
C	1.59156400	-4.53324200	3.59302100
H	1.79823300	-5.16274600	4.47185900
C	1.99511200	-4.95375400	2.31447200
H	2.52031800	-5.91286100	2.18931800
C	1.73985300	-4.15088500	1.19375500
H	2.07727400	-4.47760700	0.19882400
C	3.93856800	-0.11156900	3.21775700
H	3.78914100	-0.95020300	3.91541400
C	0.93011700	-3.30518500	3.74385600
H	0.61206600	-2.96735300	4.74264200
C	2.29701300	0.60572100	-2.81247300

H	1.22733100	0.47833500	-3.00558600
C	0.66649900	-2.50465300	2.62136200
H	0.14410900	-1.54494200	2.72932000
C	4.31585300	1.02219000	-1.72316600
H	5.05136600	1.25421500	-0.94513600
C	4.71503100	-2.30192700	-1.31502400
H	5.80032100	-2.23540500	-1.16956300
C	3.36570700	0.33119200	-3.73101900
H	3.25050900	-0.04509000	-4.75485700
Ni	-0.18107200	0.27571400	0.25388100
C	-1.66124600	-0.57035500	1.27162000
C	-1.90924300	-0.09886700	2.52553000
H	-2.77165200	-0.38658900	3.15132800
S	-0.96228800	1.22865800	3.17997300
O	-1.73973100	2.47494600	3.37610400
O	0.30505600	1.28953100	2.35235200
C	-0.44050400	0.68297500	4.82496300
H	0.16440100	1.51543700	5.23066000
H	-1.34196900	0.52526400	5.44552600
H	0.16481200	-0.23761300	4.73806600
O	-4.58402600	3.01203400	-0.98444000
S	-4.39622600	1.54239900	-0.89865300
O	-3.40460300	1.09909300	0.20081400
O	-4.23779700	0.79491700	-2.17802000
C	-6.00437200	0.89613600	-0.14294100
F	-7.02730300	1.22184600	-0.95017700
F	-6.21160500	1.44447100	1.06316200
F	-5.97111100	-0.44041900	-0.01008100
C	-1.43919400	1.61918700	-0.31281700
C	-1.52070700	3.04494500	0.11315800
C	-1.21885300	1.14227900	-1.58935900
C	-1.37536800	4.04076200	-1.07863500
H	-0.77117500	3.25761500	0.89606700
H	-2.49884100	3.17447600	0.60969600
C	-0.82976000	2.14494000	-2.66517900
H	-1.63810500	0.17480100	-1.89334400
C	-1.64622000	3.43241400	-2.45677400
H	-0.34808700	4.45482700	-1.06303600
H	-2.06943400	4.88611600	-0.90374100
H	-1.01417900	1.70404000	-3.66585000
H	0.25229300	2.39367200	-2.61706600
H	-2.72373500	3.19804500	-2.56355400
H	-1.38770700	4.17082400	-3.24519300
C	-2.47032800	-1.72854500	0.83443900

C	-2.59934300	-2.83891900	1.70853700
C	-3.14591100	-1.78232800	-0.40724200
C	-3.38685100	-3.94615500	1.36423100
H	-2.06397600	-2.82993400	2.66866100
C	-3.95025800	-2.87810000	-0.73553900
H	-3.11221800	-0.93024300	-1.09350200
C	-4.07570300	-3.96676900	0.14239400
H	-3.46765000	-4.79344300	2.06274300
H	-4.49265900	-2.86929400	-1.69247400
H	-4.70675900	-4.82804400	-0.12542700

P9E:

Eel = -5663.509296
Zero-point correction = 0.660278
Thermal correction to Energy = 0.707235
Thermal correction to Enthalpy = 0.708179
Thermal correction to Gibbs Free Energy = 0.575598

Fe	2.55286500	-2.34897700	-0.37406800
P	1.93987900	0.89613200	0.32660400
P	-0.71071300	-1.43076400	-0.10976000
C	2.57095500	0.88148500	2.05973100
C	2.62632000	2.48011600	-0.33209100
C	1.66841100	0.54044800	3.08714400
H	0.62132600	0.31395900	2.81594200
C	0.61860700	-2.66148100	0.15434700
C	-0.87333500	-0.62464300	-2.79528100
H	-0.43704300	0.31565400	-2.41960000
C	-1.77897100	-1.95421500	-4.61756700
H	-2.02961300	-2.06697200	-5.68348700
C	2.95238600	-0.36994800	-0.51828800
C	3.56209000	3.80910900	-2.15090400
H	4.00225500	3.86125800	-3.15854800
C	3.89831600	1.22720000	2.38616200
H	4.59592800	1.53210200	1.59084600
C	-2.14372000	-2.19608500	0.75858900
C	3.37863300	4.98165800	-1.40180600
H	3.66972000	5.95709700	-1.81989100
C	3.42381500	0.83868300	4.74185300
H	3.75934500	0.82220000	5.79007000
C	-1.14877300	-1.66652300	-1.88818500
C	-1.76498600	-2.84706800	-2.35319100
H	-2.01571600	-3.64883200	-1.64155000

C	1.48082400	-2.66864600	1.31834200
H	1.42116200	-1.96989400	2.16118000
C	-1.18425400	-0.76990600	-4.15627700
H	-0.97647700	0.05763500	-4.85116700
C	2.44839800	3.66245600	0.41912000
H	2.01118600	3.60811200	1.42887500
C	2.82393100	4.90368800	-0.11266400
H	2.68048900	5.81789500	0.48327100
C	4.32205400	1.20092800	3.72340500
H	5.35924300	1.47138700	3.97422600
C	1.07908700	-3.71108100	-0.72776300
H	0.65958300	-3.94158500	-1.71383000
C	4.47365900	-2.06241900	-1.03434200
H	5.29302400	-2.78752400	-0.95305200
C	3.18812500	2.56357800	-1.62146900
H	3.33770400	1.64972900	-2.21534500
C	2.20571700	-4.35287400	-0.11046100
H	2.79855800	-5.16674100	-0.54636700
C	-2.07336500	-2.99014000	-3.71392700
H	-2.55559500	-3.91300000	-4.07116700
C	-4.38545900	-3.11293400	2.20513400
H	-5.25845600	-3.46961300	2.77322600
C	-3.10133700	-3.59189500	2.51458700
H	-2.96622800	-4.33049500	3.31988100
C	-1.98333700	-3.13472900	1.79922300
H	-0.98156700	-3.51574100	2.04780400
C	2.09523500	0.51399100	4.42405500
H	1.38440600	0.24820400	5.22118000
C	-4.55111300	-2.17810600	1.17010900
H	-5.53972900	-1.76182900	0.92784900
C	2.60366000	-0.92087300	-1.81229000
H	1.74263800	-0.62383300	-2.42239200
C	-3.43780800	-1.72352900	0.44973400
H	-3.58510800	-0.98032100	-0.34593800
C	4.11555800	-1.08690500	-0.04303100
H	4.60511600	-0.93938600	0.92625300
C	2.45170100	-3.71292500	1.15160800
H	3.26301000	-3.95172400	1.85022000
C	3.54530900	-1.95780600	-2.12508800
H	3.53008800	-2.58637200	-3.02388400
Ni	-0.25677600	0.66932500	0.30620800
C	-1.98660600	1.36851900	0.66606200
C	-3.01860600	1.14334000	1.50510700
H	-3.00688200	0.38280100	2.30258400

S	-4.64583700	1.84192300	1.22607800
O	-5.45562700	0.86955000	0.42552400
O	-4.54699600	3.24574700	0.75833500
C	-5.38308800	1.85612400	2.88510600
H	-6.41233700	2.23242900	2.73779100
H	-5.40002800	0.82468100	3.28317200
H	-4.80210600	2.53796600	3.53235200
C	-1.92539700	2.13501500	-0.58272900
C	-0.80518400	2.98877100	-0.80820000
C	-2.81132900	1.89052500	-1.67116600
C	-0.54756800	3.52435500	-2.08161900
H	-0.16900600	3.26806100	0.04451000
C	-2.55372100	2.44041600	-2.92909400
H	-3.70287900	1.26858300	-1.50208500
C	-1.41319200	3.24012200	-3.14855600
H	0.33182600	4.17070600	-2.22620700
H	-3.25017100	2.24043900	-3.75809300
H	-1.21540500	3.65682100	-4.14806700

P9-3ETS:

Eel = -6858.933448
Zero-point correction = 0.821465
Thermal correction to Energy = 0.882944
Thermal correction to Enthalpy = 0.883888
Thermal correction to Gibbs Free Energy = 0.722627

Fe	3.84723800	-0.30357300	-1.46891400
P	1.85227700	1.02380100	1.11249300
P	0.67531400	-1.64071700	-0.95643400
C	2.42023900	0.41469900	2.77084300
C	1.75304500	2.85178000	1.34912000
C	2.03387400	-0.87350400	3.18272200
H	1.34484800	-1.45408700	2.55273400
C	2.21342100	-1.42358700	-1.92709200
C	-1.91096700	-2.01446200	-1.98299600
H	-2.26837800	-1.53166000	-1.06254700
C	-2.43116800	-3.00572500	-4.14352600
H	-3.17087200	-3.32941100	-4.89174900
C	3.42626600	0.83703900	0.17551200
C	2.75834600	5.07294000	1.44904100
H	3.65219900	5.71350200	1.39870600
C	3.26276600	1.17685900	3.60556500
H	3.55685100	2.19471400	3.31100400

C	1.07433900	-3.22607800	-0.08664300
C	1.49099800	5.64380400	1.65381000
H	1.38789400	6.73528900	1.75055000
C	3.35021500	-0.65441500	5.21121600
H	3.71418900	-1.06774200	6.16404000
C	-0.53428300	-2.17058200	-2.23864200
C	-0.10900400	-2.74275300	-3.45620500
H	0.96455500	-2.86115500	-3.66532600
C	3.40012000	-2.25430700	-1.89285800
H	3.55450900	-3.12591800	-1.24868100
C	-2.85466300	-2.43922700	-2.93109600
H	-3.92474500	-2.30845100	-2.70816700
C	0.49040300	3.43323600	1.58153000
H	-0.41069600	2.80906300	1.60275500
C	0.35588500	4.82248900	1.71807700
H	-0.65344200	5.24457500	1.82497400
C	3.72093100	0.64351500	4.81895800
H	4.37371200	1.24894300	5.46601800
C	2.43214900	-0.39371700	-2.91794000
H	1.73158300	0.40192100	-3.18362300
C	5.56309300	0.12546400	-0.44674200
H	6.50187800	-0.44017000	-0.49073000
C	2.89254500	3.68603700	1.28807700
H	3.88625700	3.25479300	1.10000900
C	3.73488900	-0.59467400	-3.48475400
H	4.20276000	0.04314600	-4.24468400
C	-1.05767400	-3.15342700	-4.40438100
H	-0.72065900	-3.59229300	-5.35599100
C	1.67760900	-5.56926000	1.37243000
H	1.90537700	-6.48062700	1.94550600
C	2.42408600	-4.39797300	1.58128800
H	3.24484400	-4.38403400	2.31481200
C	2.12185500	-3.23487300	0.85925900
H	2.71753800	-2.32596100	1.01808400
C	2.50280600	-1.41341300	4.39055600
H	2.18810300	-2.42437000	4.69225200
C	0.64375200	-5.57281200	0.42292300
H	0.06119700	-6.48939400	0.24478800
C	3.87057300	1.64689900	-0.93747800
H	3.29095600	2.43717200	-1.42474400
C	0.34495100	-4.41199800	-0.30775500
H	-0.46184300	-4.42687400	-1.05516300
C	4.48078800	-0.11102600	0.46702100
H	4.46388400	-0.85499000	1.27237200

C	4.33385700	-1.73556700	-2.84917100
H	5.33915100	-2.13032800	-3.04177300
C	5.19016100	1.21527400	-1.30629900
H	5.78899400	1.62256600	-2.13029500
Ni	-0.07542400	-0.08388100	0.52572100
C	-0.93095200	1.29349100	-0.66693300
C	-1.98668400	1.93587200	-0.15140300
H	-2.55294200	1.67361400	0.75488900
S	-2.66990600	3.49268900	-0.77186700
O	-2.12940100	3.85347000	-2.10635900
O	-2.55029400	4.45126400	0.36427200
C	-4.42114900	3.09437900	-0.95467100
H	-4.92007300	4.05914600	-1.16394000
H	-4.52908900	2.39124000	-1.79972500
H	-4.77256900	2.63945400	-0.00876500
O	-4.63705600	-1.29131600	2.34193800
S	-4.53380800	-0.27871900	1.25890800
O	-3.43006900	-0.54653100	0.23615900
O	-4.62426700	1.15148200	1.67021400
C	-6.04861600	-0.59198900	0.17243500
F	-7.16398800	-0.58884700	0.91371300
F	-5.93740300	-1.78733800	-0.43917600
F	-6.15845900	0.35842800	-0.77690600
C	-1.51998700	-0.99133800	1.34536900
C	-1.81678200	-2.43460600	1.55766800
C	-1.36512200	-0.05461400	2.33816800
C	-1.24077800	-2.86912300	2.92740600
H	-1.42908300	-3.07531800	0.74733900
H	-2.91886600	-2.54431900	1.58619600
C	-1.21341700	-0.46582300	3.79677200
H	-1.57205700	1.01112700	2.15774600
C	-1.68887700	-1.90779800	4.03329400
H	-0.13239800	-2.93101100	2.86900400
H	-1.59699500	-3.89865500	3.13750500
H	-1.81311800	0.23963700	4.40856800
H	-0.16010800	-0.32627600	4.12088800
H	-2.79815400	-1.90804700	4.05900600
H	-1.33175200	-2.26123700	5.02321900
C	-0.31446100	1.63874000	-1.95129100
C	-0.79656300	1.05745100	-3.15070500
C	0.68904000	2.63426500	-2.03684700
C	-0.28491600	1.45743700	-4.39068800
H	-1.59682900	0.30802200	-3.09724800
C	1.19171100	3.03378300	-3.28029100

H	1.02656700	3.12990000	-1.11656500
C	0.71370300	2.44408700	-4.46316700
H	-0.67840600	1.00016400	-5.31113900
H	1.94970300	3.83092800	-3.32964200
H	1.10799400	2.76280300	-5.43984400

P10:

Eel = -5355.084556
Zero-point correction = 0.551731
Thermal correction to Energy = 0.591440
Thermal correction to Enthalpy = 0.592384
Thermal correction to Gibbs Free Energy = 0.476498

Fe	0.21841800	-2.44224900	-1.49659300
P	-1.59430100	-0.09900400	0.12870600
P	1.79690700	0.10948800	-0.00333200
C	-2.93699700	0.45542300	-0.99136000
C	-2.33940600	-0.21583200	1.80297500
C	-2.60383700	1.19524000	-2.14371100
H	-1.56079300	1.49070700	-2.33393700
C	1.71346300	-1.11840000	-1.32922000
C	1.22059700	-0.80139500	2.59322700
H	0.26122900	-0.27582500	2.47423000
C	2.72793200	-2.15211100	3.93890700
H	2.94728200	-2.69319200	4.87148100
C	-1.33622600	-1.82846800	-0.37082900
C	-3.48736000	-1.45456800	3.55759800
H	-3.93965600	-2.38954600	3.92065100
C	-4.28112500	0.11476200	-0.73374900
H	-4.54703400	-0.45276700	0.17066900
C	3.28683700	1.10738300	-0.34537800
C	-3.48160500	-0.31220900	4.37355400
H	-3.92643700	-0.35172700	5.37902000
C	-4.94733200	1.24363100	-2.78479700
H	-5.73715200	1.55675900	-3.48387800
C	2.17120700	-0.77544000	1.55338500
C	3.41849200	-1.41345900	1.72457700
H	4.18316300	-1.35397700	0.93487400
C	0.99110300	-0.90792200	-2.56968800
H	0.46129700	0.01024800	-2.85325200
C	1.49852200	-1.49300500	3.78214500
H	0.75196600	-1.50882400	4.59022000
C	-2.33587000	0.92909400	2.62771900

H	-1.87837600	1.86080400	2.26099800
C	-2.90624400	0.88145000	3.90660600
H	-2.89968400	1.77785000	4.54446800
C	-5.28107300	0.51056900	-1.63308000
H	-6.33020700	0.24847900	-1.43019600
C	2.24393900	-2.46596100	-1.35154500
H	2.80934700	-2.94813900	-0.54582700
C	-1.35134700	-3.77057300	-1.65487900
H	-1.52419800	-4.47505300	-2.47752700
C	-2.91698600	-1.41252100	2.27581300
H	-2.92043800	-2.31252800	1.64367400
C	1.84899000	-3.06927000	-2.59132400
H	2.06394700	-4.10104900	-2.89543800
C	3.68787600	-2.10666400	2.91310700
H	4.65884200	-2.60729100	3.04416200
C	5.53798100	2.71137900	-0.82650300
H	6.42204700	3.33868200	-1.01466800
C	5.08699100	1.82209800	-1.81444100
H	5.61735100	1.74861700	-2.77549900
C	3.95963600	1.02048700	-1.58059600
H	3.60978900	0.32212000	-2.35523500
C	-3.61047600	1.58717700	-3.03849500
H	-3.34672200	2.17589200	-3.92928100
C	4.86137400	2.80353700	0.40184400
H	5.21345700	3.50255300	1.17493400
C	-0.56348800	-2.83795100	0.32806300
H	-0.04935300	-2.70939800	1.28761300
C	3.73431500	2.00915600	0.64419200
H	3.20201900	2.08520800	1.60449800
C	-1.81880700	-2.41706500	-1.60365500
H	-2.42183700	-1.90296600	-2.36108400
C	1.08059300	-2.11526500	-3.33863800
H	0.61203300	-2.28935500	-4.31479100
C	-0.58486500	-4.03041500	-0.47019400
H	-0.07022300	-4.96856800	-0.22926100
Ni	0.00572700	1.36686000	0.09742200
S	-0.70287500	3.37324700	-0.15727300
O	0.85486900	3.12673600	0.05692500
O	-1.17708200	3.98381400	-1.42921700
C	-1.13419100	4.51342200	1.18756700
H	-0.74652100	4.10516100	2.13769200
H	-0.65072000	5.47561600	0.93122200
H	-2.23470100	4.61641000	1.19196600

P3Z':

Eel = -6858.956944
Zero-point correction = 0.821083
Thermal correction to Energy = 0.883814
Thermal correction to Enthalpy = 0.884758
Thermal correction to Gibbs Free Energy = 0.717614

Fe	1.59006000	2.83858000	-0.16111600
P	3.25452200	-0.07310600	0.28785600
P	-1.63283900	1.39945600	-0.51633400
C	4.37840900	0.26569500	-1.14671500
C	4.48754300	-0.62359000	1.55375100
C	3.91805200	-0.04924000	-2.44193900
H	2.93761300	-0.52778700	-2.58802300
C	-0.14729500	2.30970600	-1.08821100
C	-1.88776200	2.68451100	1.92744700
H	-1.21075700	1.89291900	2.27520900
C	-3.17339700	4.70213300	2.37188500
H	-3.49729300	5.49432200	3.06379300
C	2.86321500	1.62500300	0.86065100
C	5.66312300	-0.43107900	3.68469100
H	5.89528400	0.14906900	4.59133100
C	5.66826500	0.81718400	-0.98771900
H	6.05011700	1.02687400	0.02375100
C	-2.60696200	1.27064800	-2.07884500
C	6.23558700	-1.69814700	3.49636500
H	6.91561400	-2.11645800	4.25398100
C	5.99048800	0.79977900	-3.39922900
H	6.61908500	1.01073400	-4.27811300
C	-2.34791400	2.67243100	0.59530600
C	-3.21726300	3.69028000	0.15573000
H	-3.56223400	3.70688300	-0.88753400
C	0.99600800	1.78405800	-1.79980400
H	1.18399800	0.72374800	-1.98893100
C	-2.29570200	3.69695600	2.80988400
H	-1.92708900	3.69603300	3.84719000
C	5.06041800	-1.90412900	1.37644900
H	4.80702900	-2.49043600	0.47778000
C	5.93398200	-2.43269400	2.33578600
H	6.37763600	-3.42846300	2.18166900
C	6.46703500	1.08738900	-2.10840100
H	7.47068800	1.51995300	-1.97405800
C	0.00168900	3.75273000	-1.03467600

H	-0.68923700	4.44941200	-0.54742200
C	2.73469200	3.94603600	1.11441700
H	2.94424200	5.01839700	1.01339600
C	4.79580000	0.10648400	2.71890600
H	4.35141100	1.10193000	2.87093900
C	1.21312700	4.09883100	-1.72141400
H	1.61820000	5.11245100	-1.83210100
C	-3.63220700	4.69423400	1.04397900
H	-4.31307000	5.48370300	0.69104400
C	-3.98336600	0.79851900	-4.49915200
H	-4.52223900	0.60758700	-5.43961600
C	-2.59362800	0.61647700	-4.43177400
H	-2.03048800	0.27146400	-5.31153800
C	-1.90670000	0.85696700	-3.23506900
H	-0.82225800	0.69136400	-3.20637700
C	4.72053500	0.22530800	-3.56189400
H	4.34677900	-0.02508200	-4.56652700
C	-4.68276500	1.21329200	-3.35566800
H	-5.77451800	1.34839300	-3.39397200
C	1.69829200	1.94065600	1.66177600
H	0.99449700	1.20431800	2.06664400
C	-4.00719700	1.43691400	-2.14671500
H	-4.57644000	1.69613000	-1.24701200
C	3.49981800	2.88071400	0.52778900
H	4.38556100	2.99336000	-0.10786400
C	1.82557000	2.88676800	-2.19041000
H	2.78439400	2.79878900	-2.71600900
C	1.62131300	3.36692800	1.81567700
H	0.83359400	3.91620500	2.34547400
Ni	-1.37979200	-0.80240900	0.17744300
C	-1.91075800	-0.80611800	1.97759600
C	-2.99960200	-0.36427000	2.65866700
H	-3.06153500	-0.39486300	3.76083700
S	-4.56442900	0.02817600	1.93563100
O	-5.46110200	-1.14520400	2.08593000
O	-4.38474400	0.62350300	0.57888200
C	-5.21115100	1.34319800	2.99243400
H	-6.21018000	1.57130900	2.57678100
H	-5.30449600	0.96302600	4.02625900
H	-4.54330800	2.22193400	2.92986900
C	-2.61550300	-2.23553300	0.03635000
C	-3.66071400	-2.01276100	-1.01063000
C	-2.29091000	-3.42542400	0.58414600
C	-4.51455900	-3.28723700	-1.17255400

H	-3.16911100	-1.74350200	-1.96935300
H	-4.29659200	-1.15208700	-0.72462100
C	-2.80313900	-4.72352200	0.00028000
H	-1.60316400	-3.50309700	1.44084000
C	-3.61622000	-4.52288400	-1.28365000
H	-5.15940700	-3.17933100	-2.06882300
H	-5.18585300	-3.37639400	-0.29059900
H	-1.94005100	-5.40181100	-0.17854500
H	-3.41623700	-5.22320000	0.78789700
H	-4.21452400	-5.43268700	-1.49820200
H	-2.91674200	-4.37920800	-2.13373400
O	-0.09768100	-1.01432600	-1.26674400
S	0.11154300	-2.00435400	-2.43859800
O	-1.11601600	-2.69808000	-2.87669600
O	0.98860600	-1.38943900	-3.46537500
C	1.17223300	-3.34991200	-1.62196500
F	0.65948700	-3.69973900	-0.42887200
F	1.19629200	-4.42836300	-2.41535500
F	2.43125100	-2.91856500	-1.43624800
C	-0.81931700	-1.48727300	2.72319800
C	0.51580200	-1.47460900	2.24723500
C	-1.10065100	-2.25373000	3.88351900
C	1.53110000	-2.16922600	2.91737000
H	0.77785200	-0.93138500	1.32269400
C	-0.08339900	-2.94065500	4.55777500
H	-2.14027700	-2.33590900	4.23335400
C	1.23714500	-2.89754500	4.08044200
H	2.55641500	-2.13914100	2.52255500
H	-0.32798600	-3.53082300	5.45431100
H	2.03587900	-3.44379900	4.60449100

Phen1:

Eel = -2313.531355

Zero-point correction = 0.301211

Thermal correction to Energy = 0.318581

Thermal correction to Enthalpy = 0.319525

Thermal correction to Gibbs Free Energy = 0.255604

C	-2.36990900	3.31132200	0.02704200
C	-3.48264600	2.47301600	0.01979900
C	-3.28867500	1.06663300	0.00775000
C	-1.94467100	0.60489200	0.00446300
C	-1.07079100	2.75866000	0.02212400

C	-4.34525800	0.08813500	-0.00180500
C	-1.65865700	-0.79392700	-0.00756500
C	-2.71230700	-1.74722600	-0.01874100
C	-4.06824800	-1.26079100	-0.01483400
C	-2.34298800	-3.11756200	-0.03359800
H	-3.11953400	-3.89683900	-0.04266000
C	-0.99136900	-3.45396900	-0.03733500
C	-0.00750300	-2.44324300	-0.02502900
H	-5.38747100	0.44021800	0.00089200
H	-2.48444400	4.40427900	0.03620900
H	-4.50180800	2.88768600	0.02308100
H	-0.18335500	3.40949400	0.02700000
H	-4.88721600	-1.99521300	-0.02276900
H	-0.66906500	-4.50451500	-0.04987600
H	1.06166900	-2.69163900	-0.02757900
N	-0.85410000	1.43362500	0.01104500
N	-0.32822500	-1.13750900	-0.00894000
Ni	0.78573700	0.36840700	0.00733800
C	2.49042100	-0.29937400	0.01494900
C	3.47013100	-1.41032600	0.02053700
C	2.75466000	1.00655700	0.00634200
C	4.86623800	-0.81936600	0.36657600
H	3.47960600	-1.90099800	-0.97855400
H	3.19524600	-2.20067700	0.75205600
C	4.14005500	1.61245300	-0.01309900
H	1.87498500	1.76129000	0.02499700
C	5.14415700	0.49852800	-0.37028100
H	5.64512500	-1.56915300	0.12248700
H	4.91782300	-0.65059900	1.46338300
H	4.19931400	2.45320400	-0.73467700
H	4.35665400	2.04757500	0.98810800
H	6.17003600	0.84966800	-0.13911000
H	5.11224100	0.32151100	-1.46696900

Phen1-2ZTS:

Eel = -3210.316553
Zero-point correction = 0.456708 (Hartree/Particle)
Thermal correction to Energy = 0.486629
Thermal correction to Enthalpy = 0.487573
Thermal correction to Gibbs Free Energy = 0.394482

C	-1.53861100	-4.41951200	0.65939000
C	-0.27003400	-4.98290100	0.56086900

C	0.81642600	-4.15659700	0.17235000
C	0.53571200	-2.78571600	-0.08077300
C	-1.73235300	-3.04943200	0.38830500
C	2.17082300	-4.61929200	0.00839900
C	1.58203500	-1.90595000	-0.50206300
C	2.90874600	-2.38642200	-0.68124600
C	3.17580500	-3.77254900	-0.40248300
C	3.87010300	-1.44824400	-1.14267600
H	4.91129500	-1.76637000	-1.30344500
C	3.47521200	-0.13724500	-1.39547000
C	2.13292000	0.24643500	-1.17569900
H	2.38673200	-5.67879000	0.21154300
H	-2.40862100	-5.02698200	0.94571000
H	-0.10337100	-6.05002400	0.77021600
H	-2.72847000	-2.58930900	0.43881600
H	4.20135100	-4.14927100	-0.53209000
H	4.19180200	0.61138000	-1.75900000
H	1.78800700	1.27493200	-1.35410000
N	-0.72146300	-2.23281700	0.03341100
N	1.20981800	-0.61443100	-0.73484500
Ni	-0.75660100	-0.37137200	-0.32593200
C	-2.60582700	-0.25482100	0.03552800
C	-3.01476100	-0.11214000	1.47968500
C	-3.46986300	-0.26628800	-1.00084200
C	-4.53281500	-0.32469200	1.65550000
H	-2.72858600	0.90270500	1.83184300
H	-2.44932900	-0.82275800	2.12291500
C	-4.96499800	-0.08148600	-0.82172000
H	-3.10494900	-0.41087700	-2.03444700
C	-5.31778300	0.43098000	0.57935700
H	-4.83784000	-0.00353300	2.67245800
H	-4.76334300	-1.41193900	1.58589200
H	-5.34702700	0.61450500	-1.59986000
H	-5.47725100	-1.05134100	-1.02551600
H	-6.40913600	0.34309100	0.75595100
H	-5.06528700	1.51083000	0.64388000
C	0.98384200	3.57496000	0.71357900
C	2.22240700	2.98111100	0.87750700
C	3.35376500	3.39313100	0.09329900
C	2.40272400	1.94474300	1.85751700
C	4.59461900	2.79252400	0.29193700
H	3.22899900	4.19503800	-0.64822300
C	3.65460700	1.36106000	2.03195900
H	1.54027100	1.62363900	2.45839500

C	4.75652100	1.77704700	1.25719000
H	5.45743200	3.12493100	-0.30481900
H	3.78128600	0.56958200	2.78560900
H	5.74329200	1.31651600	1.41135200
C	-0.24501900	3.93364300	0.46957600
H	-0.79682100	4.81269100	0.85131900
S	-1.38231900	2.81787900	-0.45282600
O	-2.66805100	2.81004600	0.26963100
O	-0.57737500	1.54342800	-0.70936500
C	-1.62728000	3.52449700	-2.08778500
H	-0.65626100	3.56680400	-2.61277400
H	-2.33382900	2.84111600	-2.59763600
H	-2.07504300	4.52766000	-1.96191700

Phen1-2ETS:

Eel = -3210.302122
Zero-point correction = 0.455645
Thermal correction to Energy = 0.486060
Thermal correction to Enthalpy = 0.487005
Thermal correction to Gibbs Free Energy = 0.391592

C	-0.44037700	3.86179800	-0.97792900
C	-1.81326100	3.72677300	-0.79018200
C	-2.39845600	2.43564800	-0.86589600
C	-1.52826800	1.34644600	-1.14336200
C	0.35363900	2.72564000	-1.23920900
C	-3.79652000	2.15396600	-0.66811300
C	-2.03633500	0.01269400	-1.22248500
C	-3.41692600	-0.24601000	-1.01998100
C	-4.28419900	0.86751800	-0.73974100
C	-3.83307700	-1.60139200	-1.10021700
H	-4.89069700	-1.86008100	-0.94223300
C	-2.88959200	-2.58683600	-1.36487500
C	-1.53415500	-2.23155500	-1.54648900
H	-4.47417400	2.99339600	-0.45277900
H	0.04594100	4.84560400	-0.92326500
H	-2.44413500	4.60381300	-0.58166200
H	1.43941500	2.81010700	-1.37777300
H	-5.35484100	0.67168300	-0.57966600
H	-3.16898200	-3.64785700	-1.40966000
H	-0.77341100	-3.00423600	-1.72867300
N	-0.17392400	1.49314600	-1.32508000
N	-1.11311700	-0.96230200	-1.48219700

Ni	0.69967400	-0.15960300	-1.53811200
C	2.49118900	0.14941800	-1.52750200
C	3.57000800	0.97744400	-0.94225400
C	2.60046800	-1.10648800	-2.01360900
C	4.91201300	0.20126500	-1.00602200
H	3.29960900	1.26088600	0.09836900
H	3.66116000	1.93543800	-1.50254000
C	3.80977600	-1.97812300	-1.72792400
H	1.76088600	-1.55547800	-2.61976000
C	4.71774400	-1.28972500	-0.69636100
H	5.63226000	0.65990900	-0.29926900
H	5.34774600	0.31183100	-2.02186200
H	3.48661500	-2.98077600	-1.37529600
H	4.35365000	-2.15355700	-2.68316700
H	5.69903100	-1.80446800	-0.66477900
H	4.26660300	-1.39877000	0.31476100
C	1.12379300	-0.60863900	1.34863700
C	0.96558600	0.64112200	1.99328800
C	2.07054000	1.25147900	2.65805200
C	-0.28493700	1.33010400	1.96329500
C	1.92874700	2.50662200	3.25520900
H	3.03294700	0.71982000	2.69500200
C	-0.40403400	2.58831200	2.55707300
H	-1.15102900	0.82720700	1.51382700
C	0.69739200	3.18517500	3.19828200
H	2.78623400	2.96491400	3.77004500
H	-1.37578900	3.10470900	2.53843200
H	0.59345100	4.17511100	3.66683500
C	0.73914900	-1.83873200	1.08742100
H	1.19293000	-2.54344100	0.36906900
S	-0.84243200	-2.53339200	1.75115900
O	-1.06441100	-3.80332100	1.01158100
O	-1.86415200	-1.45169900	1.70969900
C	-0.52103000	-2.91440300	3.48270500
H	-0.24065800	-1.97744100	3.99800000
H	-1.47249100	-3.31477100	3.88194400
H	0.28029900	-3.67326100	3.53453700

Phen2Z:

Eel = -3210.369901

Zero-point correction = 0.458746

Thermal correction to Energy = 0.488793

Thermal correction to Enthalpy = 0.489738

Thermal correction to Gibbs Free Energy = 0.396849

C	2.69393600	3.46058400	-0.43161000
C	3.88991900	2.79636100	-0.68671200
C	3.90600100	1.37642500	-0.71952000
C	2.66681200	0.71394700	-0.49326800
C	1.51183800	2.71546400	-0.22002400
C	5.08170800	0.57992700	-0.95680500
C	2.60667000	-0.72189900	-0.48306900
C	3.78552700	-1.48789600	-0.71147300
C	5.02393200	-0.79536300	-0.95475600
C	3.64955300	-2.90099700	-0.67323400
H	4.52936900	-3.53909500	-0.84687900
C	2.40225900	-3.46178000	-0.41274200
C	1.29042200	-2.61738800	-0.19322700
H	6.03679100	1.09507600	-1.13802800
H	2.65185300	4.55797700	-0.39046000
H	4.82170700	3.35703500	-0.85622000
H	0.54448300	3.19696400	-0.00409600
H	5.93186100	-1.38995600	-1.13485700
H	2.26652500	-4.55185500	-0.37632400
H	0.29024500	-3.02746100	0.01865300
N	1.49991900	1.38027200	-0.26208000
N	1.39013800	-1.28432100	-0.23095900
Ni	-0.04048700	0.13706000	0.09267500
C	-1.38748500	1.47770000	-0.04577300
C	-1.64901600	1.75391000	-1.50572000
C	-1.93811500	2.16240700	0.97330700
C	-2.24645600	3.16590200	-1.69135800
H	-2.34581800	0.98696200	-1.90871500
H	-0.72123300	1.65249300	-2.11520900
C	-2.89601000	3.31400100	0.74887000
H	-1.71019300	1.90192800	2.01931900
C	-3.38906800	3.39747100	-0.69988500
H	-2.59224100	3.28157300	-2.73885000
H	-1.45210800	3.92857200	-1.53255100
H	-3.75060100	3.21515300	1.45202500
H	-2.38936100	4.26157300	1.04760800
H	-3.87231000	4.37836600	-0.88473000
H	-4.17130300	2.62529800	-0.86756700
C	-1.52781700	-1.01274900	0.38098700
C	-2.35429400	-1.46531600	-0.74420700
C	-1.77124500	-1.68523700	-2.01690700
C	-3.74985300	-1.65589000	-0.59504300

C	-2.55255300	-2.11185000	-3.09771800
H	-0.69028100	-1.52169200	-2.15056600
C	-4.52996600	-2.06847000	-1.68190100
H	-4.22162100	-1.44700300	0.37659100
C	-3.93437500	-2.30334700	-2.93313800
H	-2.08385700	-2.28867300	-4.07720400
H	-5.61452100	-2.20320200	-1.55463400
H	-4.55103700	-2.62753700	-3.78458100
C	-1.72367300	-1.41987500	1.66307500
H	-2.49944900	-2.11067400	2.03443500
S	-0.75893000	-0.62076800	2.90638800
O	-1.58464000	0.13949600	3.86607200
O	0.32434000	0.14083400	2.13165600
C	0.11875500	-1.90104900	3.81366100
H	0.73088800	-2.49321900	3.10989300
H	0.75329600	-1.36346800	4.54379000
H	-0.62698900	-2.52477700	4.34066600

Phen2E:

Eel = -3210.340496
Zero-point correction = 0.457393
Thermal correction to Energy = 0.488076
Thermal correction to Enthalpy = 0.489020
Thermal correction to Gibbs Free Energy = 0.392810

C	-4.11279000	2.56865100	-0.05082900
C	-5.01002700	1.51892500	0.11867400
C	-4.52832100	0.18308100	0.12430600
C	-3.12457500	-0.00065500	-0.03562700
C	-2.73769700	2.29090600	-0.21122600
C	-5.36727600	-0.97675900	0.27187200
C	-2.56869400	-1.32688600	-0.05272400
C	-3.42368400	-2.45866900	0.07210700
C	-4.83668600	-2.24600800	0.24350600
C	-2.81106000	-3.73915100	0.00831600
H	-3.42997100	-4.64468100	0.10034200
C	-1.43654200	-3.82851000	-0.18101100
C	-0.66339800	-2.64905200	-0.28684700
H	-6.44961500	-0.82837800	0.40125400
H	-4.45546800	3.61268500	-0.06697100
H	-6.08667400	1.71081300	0.24235100
H	-2.00093400	3.09375300	-0.36819000
H	-5.49059100	-3.12457500	0.34801700

H	-0.92881600	-4.80070800	-0.25045800
H	0.42540000	-2.71289900	-0.45435900
N	-2.25471500	1.04216000	-0.19909600
N	-1.21561700	-1.43090900	-0.21219600
Ni	-0.31613000	0.44574600	-0.30955500
C	0.40990200	2.16516700	-0.55623300
C	0.77656100	2.97210500	0.65711300
C	0.51036700	2.63731100	-1.82525700
C	0.97142400	4.46085400	0.31717700
H	1.70141400	2.54306700	1.10707400
H	0.00788500	2.84636500	1.45378700
C	1.14133100	3.97058100	-2.15680800
H	0.12753700	2.04429200	-2.67741600
C	1.82473600	4.61458100	-0.94541100
H	1.43374600	4.98319000	1.17905800
H	-0.02040600	4.93863200	0.15428800
H	1.85744900	3.84473500	-2.99795300
H	0.34439900	4.63895300	-2.56259500
H	2.03518900	5.68347300	-1.15121200
H	2.81048400	4.12809200	-0.77684900
C	1.45587000	-0.20142700	-0.16165800
C	1.91702600	-0.15275200	1.22029000
C	3.17130900	0.44775600	1.52662900
C	1.08938300	-0.59277200	2.29067200
C	3.58272500	0.58378900	2.85283900
H	3.81163900	0.78827200	0.70155100
C	1.51275900	-0.45505900	3.61594800
H	0.12396400	-1.06926800	2.06724300
C	2.75687600	0.13453200	3.90097900
H	4.55933200	1.03718100	3.07787900
H	0.87328100	-0.81483000	4.43538400
H	3.08790000	0.24126600	4.94475000
C	2.03160600	-0.72172700	-1.26348400
H	1.63265900	-0.60195600	-2.28518200
S	3.20737200	-2.09089700	-1.13331000
O	2.39877700	-3.31530400	-1.38990800
O	4.00252800	-1.96491400	0.10938200
C	4.31227500	-1.87264100	-2.54288300
H	4.88012900	-0.93376500	-2.41199900
H	4.98820900	-2.74822900	-2.51445600
H	3.71703000	-1.87607800	-3.47415300

Phen2-3ZTS:

Eel = -3210.364216
Zero-point correction = 0.457790
Thermal correction to Energy = 0.487312
Thermal correction to Enthalpy = 0.488256
Thermal correction to Gibbs Free Energy = 0.396782

C	3.04095800	3.25727300	-0.58522100
C	4.17925100	2.48258900	-0.78885200
C	4.07925000	1.06626700	-0.73996400
C	2.78729700	0.52240700	-0.49195800
C	1.80081600	2.62190800	-0.34522500
C	5.18843800	0.16477600	-0.91311600
C	2.61063800	-0.90396900	-0.39549900
C	3.72710200	-1.77333500	-0.55590600
C	5.01928300	-1.19853000	-0.82585200
C	3.47989400	-3.16590500	-0.42464800
H	4.30843700	-3.88063800	-0.54329500
C	2.19113100	-3.60963000	-0.14034500
C	1.14737100	-2.66803400	0.00601500
H	6.18387400	0.58877200	-1.11270300
H	3.09073100	4.35497500	-0.60562800
H	5.15531400	2.95442200	-0.97870100
H	0.87986800	3.19999000	-0.16896700
H	5.87796000	-1.87404900	-0.95533200
H	1.97160900	-4.68077500	-0.02852500
H	0.11575000	-2.98188600	0.23493100
N	1.67875900	1.29294000	-0.31347500
N	1.35204000	-1.35332300	-0.12609100
Ni	0.03428500	0.16691700	0.08732200
C	-1.52691900	1.25605200	-0.20643700
C	-1.51773000	1.41197400	-1.71808500
C	-2.11092200	2.16753300	0.61510200
C	-1.72487700	2.88055600	-2.12249600
H	-2.31389600	0.77772300	-2.16217300
H	-0.56808300	1.02374900	-2.16139300
C	-2.77739900	3.41901800	0.11480900
H	-2.11164900	2.00521900	1.70584700
C	-2.95309500	3.45234000	-1.40809100
H	-1.83745300	2.94868500	-3.22325800
H	-0.82363100	3.47731600	-1.85990600
H	-3.75225000	3.54699200	0.63384200
H	-2.16749900	4.28691100	0.46243400
H	-3.15904300	4.48721600	-1.74712600
H	-3.84281400	2.84853500	-1.68909700

C	-1.72532500	-0.55467300	0.40379000
C	-2.56641700	-1.25943200	-0.59567300
C	-1.98172400	-1.84017100	-1.74391800
C	-3.96390200	-1.36481300	-0.41585100
C	-2.77029300	-2.53160900	-2.67390600
H	-0.89616600	-1.74824600	-1.90342200
C	-4.75134400	-2.04701200	-1.35272600
H	-4.43223000	-0.88459400	0.45678800
C	-4.15714500	-2.63627600	-2.48110100
H	-2.30014000	-2.98676600	-3.55844400
H	-5.83942700	-2.11511800	-1.20413700
H	-4.77749600	-3.17087400	-3.21564700
C	-1.88961800	-0.88040500	1.73720600
H	-2.67782800	-1.53492700	2.14179800
S	-0.86328600	-0.10025000	2.91875900
O	-1.55427400	0.90260800	3.76059400
O	0.35124100	0.38084300	2.10987800
C	-0.24342100	-1.38818300	4.00928300
H	0.29185400	-2.14789700	3.41239600
H	0.43879600	-0.87339100	4.71224900
H	-1.10111600	-1.82025800	4.55752500

Phen2-3ETS:

Eel = -3210.337570
Zero-point correction = 0.457711
Thermal correction to Energy = 0.487570
Thermal correction to Enthalpy = 0.488514
Thermal correction to Gibbs Free Energy = 0.395213

C	-4.25318700	2.10565400	0.73592400
C	-5.02611300	0.99389000	0.41040900
C	-4.39239400	-0.17749900	-0.08247400
C	-2.97502300	-0.13798800	-0.20672100
C	-2.85134100	2.05235700	0.57329700
C	-5.08272900	-1.38210900	-0.46353000
C	-2.25838200	-1.27944100	-0.70319200
C	-2.96309000	-2.45411400	-1.08586000
C	-4.39627900	-2.47352100	-0.94660200
C	-2.18526200	-3.53189900	-1.58783200
H	-2.67948900	-4.46452400	-1.89962600
C	-0.80418300	-3.38919200	-1.68089700
C	-0.19045100	-2.18416700	-1.26736300
H	-6.17795900	-1.41346800	-0.36510400

H	-4.71622100	3.02722300	1.11542700
H	-6.12022200	1.01535000	0.52739600
H	-2.21469500	2.91808600	0.81293000
H	-4.93928400	-3.38475700	-1.23870800
H	-0.17177100	-4.20000700	-2.06887400
H	0.90478400	-2.06298300	-1.32649000
N	-2.22787600	0.95862500	0.11771400
N	-0.90223400	-1.15711900	-0.78731800
Ni	-0.28016700	0.64709700	-0.11189300
C	0.70346700	2.20832500	-0.02378700
C	1.03884500	3.15461600	1.08697700
C	0.62710600	2.57162300	-1.34282000
C	0.68922900	4.60729800	0.68419800
H	2.12845400	3.06805700	1.29835600
H	0.52467400	2.88843100	2.03210600
C	0.54369500	4.01688300	-1.77555100
H	0.61274100	1.79966100	-2.13577000
C	1.17606200	4.94305900	-0.72652000
H	1.12527900	5.30103400	1.43102500
H	-0.41382600	4.74343300	0.73783100
H	1.03287800	4.13056100	-2.76543800
H	-0.52402200	4.28933800	-1.94447700
H	0.95030000	6.00020600	-0.97235100
H	2.28162200	4.83961700	-0.76900200
C	1.54126400	0.10532700	0.15959400
C	1.39328500	-0.65508500	1.41549800
C	1.78370200	-2.01818100	1.52421700
C	0.71182700	-0.06663800	2.51576200
C	1.50559200	-2.74385400	2.68874700
H	2.29304000	-2.50809000	0.68148800
C	0.44203000	-0.80036400	3.67842000
H	0.41245500	0.98848500	2.46357900
C	0.83767200	-2.14308100	3.76991600
H	1.81428700	-3.79834800	2.75089700
H	-0.07600400	-0.31683200	4.51987300
H	0.62773500	-2.72163900	4.68179200
C	2.48568200	0.08939900	-0.80807700
H	2.40455300	0.69774900	-1.72412600
S	3.91622400	-0.99026100	-1.02115100
O	4.70130200	-0.39975200	-2.12406100
O	3.39077700	-2.38494900	-1.14373500
C	4.89674300	-0.88215700	0.48924700
H	4.28485100	-1.17075800	1.36329600
H	5.72698200	-1.59831200	0.33787300

H	5.28149300	0.15013800	0.57122700

Phen3:

Eel = -2079.590356
Zero-point correction = 0.169603
Thermal correction to Energy = 0.180376
Thermal correction to Enthalpy = 0.181320
Thermal correction to Gibbs Free Energy = 0.132122

C	-0.25197300	0.00046100	-3.47486000
C	-1.49628300	0.00004000	-2.85254600
C	-1.56851200	-0.00011400	-1.43284100
C	-0.33770300	0.00007000	-0.71974800
C	0.92238600	0.00063400	-2.69090600
C	-2.79989000	-0.00041500	-0.68805600
C	-0.33770300	0.00007000	0.71974800
C	-1.56851200	-0.00011400	1.43284100
C	-2.79989000	-0.00041500	0.68805600
C	-1.49628300	0.00004000	2.85254600
H	-2.42292400	-0.00013800	3.44587000
C	-0.25197300	0.00046100	3.47486000
C	0.92238600	0.00063400	2.69090600
H	-3.74886100	-0.00063300	-1.24430400
H	-0.16285700	0.00067100	-4.57020900
H	-2.42292400	-0.00013800	-3.44587000
H	1.91706000	0.00103800	-3.16218800
H	-3.74886100	-0.00063300	1.24430400
H	-0.16285700	0.00067100	4.57020900
H	1.91706000	0.00103800	3.16218800
N	0.88194300	0.00039200	-1.34964300
N	0.88194300	0.00039200	1.34964300
Ni	2.24541700	-0.00055200	0.00000000

Phen4:

Eel = -3275.200495
Zero-point correction = 0.328738
Thermal correction to Energy = 0.354932
Thermal correction to Enthalpy = 0.355877
Thermal correction to Gibbs Free Energy = 0.269173

C	-4.37889900	-2.54208100	-1.00335000
C	-5.27691200	-1.52047300	-0.69685200

C	-4.76713500	-0.24074500	-0.34868800
C	-3.35243300	-0.08798400	-0.33104100
C	-2.98982900	-2.29633100	-0.96343400
C	-5.58042900	0.90034700	-0.01923800
C	-2.76548500	1.17608100	0.00996000
C	-3.58766900	2.29537300	0.31821000
C	-5.01564000	2.11651300	0.29759600
C	-2.92924400	3.51703200	0.62342900
H	-3.51958500	4.41248900	0.86925100
C	-1.53501200	3.55631500	0.59799500
C	-0.79994200	2.39222300	0.28939400
H	-6.67488100	0.78271900	-0.02849100
H	-4.73631400	-3.54382100	-1.28226700
H	-6.36378700	-1.69016900	-0.72367700
H	-2.26402300	-3.08798300	-1.20454700
H	-5.65539700	2.97833700	0.54202400
H	-0.99259400	4.48706200	0.81829300
H	0.30101100	2.39503800	0.26042800
N	-2.47048100	-1.10111400	-0.62977500
N	-1.39215300	1.21544000	0.01437500
Ni	-0.60625400	-0.55315900	-0.28722300
C	1.22148100	-0.69325300	0.30461500
C	1.51753300	-0.49433100	1.77541100
C	0.76746400	-1.94780300	-0.23160300
C	0.80668900	-1.56154800	2.61759100
H	1.22954400	0.53101300	2.09411900
H	2.61536500	-0.57994000	1.93329100
C	0.57725600	-3.17515800	0.66057100
H	1.04143800	-2.14546100	-1.28565500
C	1.13093600	-2.96016800	2.07902400
H	-0.29352800	-1.39090200	2.58831800
H	1.11713000	-1.46813500	3.67881000
H	1.08126200	-4.05007000	0.19677800
H	-0.50028600	-3.44993200	0.72150500
H	2.23659900	-3.07606600	2.05628200
H	0.74116100	-3.74404400	2.76138700
O	3.99767100	-1.42881800	-0.13559800
S	3.57572800	-0.24347400	-0.89978500
O	1.99695400	0.17592800	-0.56358200
O	3.79146800	-0.10806800	-2.34442800
C	4.40786800	1.26863900	-0.11378900
F	5.71569100	1.23420800	-0.39036300
F	4.23718100	1.25419800	1.21546500
F	3.87451200	2.38886000	-0.61345600

R3:

Eel = -780.131635
Zero-point correction = 0.095897
Thermal correction to Energy = 0.103715
Thermal correction to Enthalpy = 0.104659
Thermal correction to Gibbs Free Energy = 0.062045

S	-1.71826500	0.00000000	-0.25931600
O	-2.20987200	-1.31931300	0.27066000
O	-2.20987300	1.31931200	0.27066300
C	0.09776400	0.00000100	-0.08349500
C	0.76948600	1.22989600	-0.05252200
C	0.76948700	-1.22989600	-0.05252600
C	2.16966000	1.21963900	0.03240100
H	0.19587500	2.16771300	-0.07424000
C	2.16965700	-1.21963900	0.03240700
H	0.19586900	-2.16770900	-0.07424400
C	2.86646700	0.00000000	0.07179400
H	2.71914500	2.17222500	0.07189600
H	2.71914700	-2.17222300	0.07190600
H	3.96503500	-0.00000400	0.13479900

R3-4TS:

Eel = -780.090438
Zero-point correction = 0.091882
Thermal correction to Energy = 0.100069
Thermal correction to Enthalpy = 0.101014
Thermal correction to Gibbs Free Energy = 0.053864

S	-2.70577200	-0.00020800	-0.35873000
O	-2.92962000	-1.27635300	0.36588600
O	-2.92841900	1.27696300	0.36445700
C	0.80596400	-0.00055000	-0.05880500
C	1.42037800	1.23654500	-0.03228200
C	1.42130500	-1.23719600	-0.03275200
C	2.83285200	1.22221500	0.02239300
H	0.85691000	2.18264900	-0.04859400
C	2.83376900	-1.22182100	0.02188900
H	0.85857900	-2.18373300	-0.04943700
C	3.52647700	0.00045200	0.04821400
H	3.38455100	2.17550700	0.04616400

H	3.38618800	-2.17470600	0.04528100
H	4.62596400	0.00085700	0.09157200

R4:

Eel = -231.460423
Zero-point correction = 0.085108
Thermal correction to Energy = 0.089601
Thermal correction to Enthalpy = 0.090545
Thermal correction to Gibbs Free Energy = 0.056993

C	-0.00000900	-1.40554300	0.00000100
C	1.23337100	-0.77708900	0.00001100
C	-1.23336900	-0.77709500	-0.00001200
C	1.22091700	0.63578700	-0.00001000
H	2.18392800	-1.33401600	0.00000800
C	-1.22090600	0.63580900	0.00001000
H	-2.18396000	-1.33396200	-0.00001000
C	-0.00000100	1.33133200	-0.00000100
H	2.17412800	1.18852200	-0.00001100
H	-2.17414100	1.18850100	0.00001800
H	0.00003000	2.43174900	-0.00000200

SO_2:

Eel = -548.626869
Zero-point correction = 0.006474
Thermal correction to Energy = 0.009582
Thermal correction to Enthalpy = 0.010526
Thermal correction to Gibbs Free Energy = -0.017799

S	0.00000000	0.00000000	0.37767300
O	0.00000000	1.27600800	-0.37767300
O	0.00000000	-1.27600800	-0.37767300
